# Inferring Drosophila gap gene regulatory network: a parameter sensitivity and perturbation analysis

**DOI:** 10.1186/1752-0509-3-94

**Published:** 2009-09-21

**Authors:** Yves Fomekong-Nanfack, Marten Postma, Jaap A Kaandorp

**Affiliations:** 1 Section Computational Science, Faculty of Science, University of Amsterdam, Kruislaan 403, 1098 SJ Amsterdam, the Netherlands

## Abstract

**Background:**

Inverse modelling of gene regulatory networks (GRNs) capable of simulating continuous spatio-temporal biological processes requires accurate data and a good description of the system. If quantitative relations between genes cannot be extracted from direct measurements, an efficient method to estimate the unknown parameters is mandatory. A model that has been proposed to simulate spatio-temporal gene expression patterns is the connectionist model. This method describes the quantitative dynamics of a regulatory network in space. The model parameters are estimated by means of model-fitting algorithms. The gene interactions are identified without making any prior assumptions concerning the network connectivity. As a result, the inverse modelling might lead to multiple circuits showing the same quantitative behaviour and it is not possible to identify one optimal circuit. Consequently, it is important to address the quality of the circuits in terms of model robustness.

**Results:**

Here we investigate the sensitivity and robustness of circuits obtained from reverse engineering a model capable of simulating measured gene expression patterns. As a case study we use the early gap gene segmentation mechanism in *Drosophila melanogaster*. We consider the limitations of the connectionist model used to describe GRN Inferred from spatio-temporal gene expression. We address the problem of circuit discrimination, where the selection criterion within the optimization technique is based of the least square minimization on the error between data and simulated results.

**Conclusion:**

Parameter sensitivity analysis allows one to discriminate between circuits having significant parameter and qualitative differences but exhibiting the same quantitative pattern. Furthermore, we show that using a stochastic model derived from a deterministic solution, one can introduce fluctuations within the model to analyze the circuits' robustness. Ultimately, we show that there is a close relation between circuit sensitivity and robustness to fluctuation, and that circuit robustness is rather modular than global. The current study shows that reverse engineering of GRNs should not only focus on estimating parameters by minimizing the difference between observation and simulation but also on other model properties. Our study suggests that multi-objective optimization based on robustness and sensitivity analysis has to be considered.

## Background

Gene regulatory networks (GRNs) play a fundamental role in body plan formation and development [1]. In most cases the published gene regulatory networks are based on experimental studies. Furthermore, various studies reveal that network dynamics depend on qualitative aspects (network structure) as well as the quantitative properties [2,3]. An additional option is to study gene regulatory networks in mathematical models and simulation studies. Qualitative and quantitative models may provide insights in the causal relationships between components of a network as well as the mechanisms behind the network dynamics [2]. Qualitative models mainly focus on the inference of the wiring diagram and the understanding of the connection between the dynamics of the systems and the network structure [4,5]. Understanding how the gene regulation leads to pattern formation requires the usage of quantitative models that allows inferring the network structure, but also simulating the spatio-temporal gene expression dynamics [6]. Many approaches have been used to model gene regulatory networks (see for a review [7]). In modelling processes from developmental biology especially the class of quantitative spatio-temporal, models of gene regulation is relevant. These type of models can potentially be linked with three-dimensional biomechanical models of morphogenesis and provide new insights into developmental biology.

Although there is a large variety of formalisms used to describe quantitative model of GRN, the most commonly used is based on differential equations. This deterministic modelling framework allows describing spatio-temporal continuous system, where protein products are continuously produced through the balance of activation or repression. Models can be used in reverse engineering studies to infer the network structure and dynamics of the system. Quantitative spatio-temporal models of gene regulation are typically characterized by a large set of unknown parameters. Without a suitable method for reverse engineering the network and estimating the unknown parameter values these models have no practical use. Because of incomplete data, imprecise information concerning the interaction mechanisms and the large set of unknown parameter values; reverse engineering of the gene regulatory network is by far a straightforward task [8].

Reverse engineering of GRN capable of simulating spatio-temporal gene expression mainly consists in revealing the GRN structure that leads to the observed pattern. Optimization is therefore important [9,10] and it is used to infer a gene network whether it is transcriptional or protein interaction. A system identification approach is used to select a model structure and by means of parameter estimation, the network topology is estimated from experimental data. The optimization problem consists in minimizing the difference between simulated expression profiles and available data in order to estimate the best circuit that predicts with very good accuracy the spatio-temporal gene expression. Based on the resulting parameter-set, the network diagram is extracted and one tries to establish causal-relation of the dynamic mechanism that governs the pattern formation, using further analysis. The prediction is clearly linked to the quality and amount of the data; even with sufficient data, it is not guaranteed that the inferred network corresponds to the appropriate gene network that leads to the observed pattern [11,12]. From a theoretical point of view, this question is a matter of the structural identifiability of the model [13]. Given a data set, is it possible to uniquely infer the network? Although there exist some theoretical methods [12] to investigate the identifiably before inferring the network, when confronted with a complex mechanism characterized by a multi-dimensional parameters space, the feasibility is still analytically complex. In many situations, one is confronted with an overfitting problem that leads to parameter-set with very different quantitative values and even worse, yielding different network topologies having similar good realistic patterns.

To draw conclusions, one has to differentiate between circuits that are more likely to be biologically realistic.

It was shown that for certain experimental data that it is not possible to confirm whether the inferred model is really valid [14]. As long as the model is not contradicted by the given data, it is necessary to extend the validation test further. Especially when the model does not only predict a unique network, parameter-set discrimination can be addressed using diverse models validation approaches. The quality of the circuits can for example be quantified by measuring the parameter reliability and sensitivity, the uniqueness of the predicted network, the model robustness and the predictability of the model. In this paper we focus on the model robustness against perturbation. In another paper (Fomekong-Nanfack et al., in press.) we focus on the other aspects that can be used to quantify the quality of the circuits: methods for exploring the solution space, parameter correlation and asymptotical stability analysis.

By analyzing the robustness of the inferred network, we can test the quality of the circuits. The term robustness has various meanings, but in the current study, robustness is addressed as the ability of a system to maintain its mechanical and dynamical behaviour under perturbation. From a biological view, robustness is related to stability, homeostasis, canalization, redundancy, and plasticity [15-18], and can also be applied to dynamical process in development [19]. In simulations of dynamical developmental processes such as pattern formation, one should also expect robust behaviour within a certain extent in the model [20]. In the current paper, model robustness is addressed in two different perspectives. First, we investigate the quantitative robustness of the model towards internal fluctuations in expression level. It is known that presence of noise in gene regulation can lead to phenotype variation [21,22]. There are some studies on the robustness of GRNs under the influence of molecular fluctuation [23,24] and show the importance of noise and stochastic events [25]. In most cases, deterministic models are used to infer GRNs from noisy data. It has been shown in several studies that the robustness to noise mainly depends on the network structure instead of the parameter setting [26-28]. Therefore, one way to discriminate between circuits having different gene network but exhibiting the same pattern is to analyze their behaviour under noisy conditions. Model-solutions showing more robustness or stability can be considered for further analysis. Second through simple parameter perturbation, we investigate how model-circuits behave distinctly. This analysis allows us to identify the parameters that have the most significant influence on the model and to distinguish circuits that are less sensitive to overall perturbation. The combination of these two analyses allows one to discriminate between sets of circuits that are more robust, although they are obtained from the same model description and quantitative data.

### Reverse-Engineering the gap gene network

In the current paper, the case study early development along the anteroposterior (A-P) of gap gene network of the *Drosophila melanogaster *is considered. In the gene regulatory network responsible for segmentation of the *Drosophila melanogaster *embryo [29-32] for most, if not all genes involved, experimental and bioinformatics studies are available. These studies not only give information about potential interactions between the different genes, but also provide spatio-temporal information about the gene expression patterns in the embryo. The segmentation genes leading to pattern formation, which occurs during the first stages of development after fertilization is controlled by a network of genetic interactions, with a cascade like modularity [33]. At the early stage, the maternal mRNA, located at the extremes of the egg will define the anterior-posterior axis of the embryo. Following fertilization, the process begins with the diffusion of maternal morphogen factors: Bicoid (Bcd), Nanos (Nos), Caudal (Cad) and maternal Hunchback (Hb), and the activation of the torso receptor on the poles. At cycle 13 (after 13 nuclear divisions), the gradient of these maternal gene products in the embryo promotes the transcription of the first zygotic genes during cycle 14: zygotic *hb, giant (gt), Krüppel (Kr), knirps (kni) *and *tailless (tll)*; each forming expression domains in specific regions of the embryo [34]. This set of genes; the so-called gap genes-are defined in broad domains along the anterior-posterior axis. Their transcription factors regulate the expression of another group of zygotic genes that comprises seven pair-rule genes, which form a pattern of narrow stripes along the anterior-posterior axis. Finally, the pair rule genes regulate the formation of fourteen stripes by segment polarity genes. For review about the detailed mechanism, see [35-37].

For modelling the segmentation mechanism of the early *Drosophila melanogaster *embryo, two main formalisms have been proposed: logical formalism (tackling qualitative aspects) proposed by Sánchez and Thieffry [38], and continuous models proposed by Mjolsness et al. [39] used to obtain quantitative dynamics of a system. Following this formalism, Reinitz and co-workers formulated the problem as an inverse problem [40]. Given a complete mathematical model and sufficient accurate quantitative data, the parameters in the model can be estimated by optimization techniques, i.e., by fitting the model to the data. Except for box constraints, only little experimental information is used to constrain the parameter values in the model. This inverse modelling formulation of the problem leads to different gene circuits describing different aspect of the segmentation gene mechanism [41-44].

Using the gene circuit method proposed by Reinitz *et al *[40], Jaeger et al. [42,45] have inferred a network by means of reverse engineering that can reproduce the measured spatial and temporal gene expression patterns. The gap gene model involves seven different genes, *bcd, cad, hb, gt, Kr, kni *and *tll*. The experimental data used to fit the model were obtained from the FlyEx database, where an extensive amount of accurate quantified spatio-temporal expression data for all genes is stored [46,47]. A connectionist description was used to model the gene regulatory network. The number of parameters in this framework mounts up to 66 different unknowns for a network of six genes. To estimate these parameters the model was fitted to detailed spatio-temporal data using parallel simulated annealing (PLSA) [48-50], which is a technique that performs a global parameter search. Ten different gene circuits were obtained using this reverse engineering approach. Compared to the variance in the experimental data, all solutions fitted the dataset spatially and temporally accurately. Furthermore, the model reproduced the experimentally observed dynamic shift of the expression profiles and it was shown that diffusion is not the cause of the shift. Compared with experimental knowledge, for most interactions in the gene regulatory network the sign, i.e. activation, repression or no-interaction was reasonably well reproduced. Analysis of the 10 circuits suggested asymmetric repression between the gap genes, which was hypothesized to be the cause of the observed domain shift. This result has also been obtained by Perkins et al. [43] where parameter estimation was performed using a three-step strategy. Following [42], Fomekong-Nanfack et al. [51] employed a different optimization technique to find parameter sets that accurately fitted the experimental dataset and lead to the similar networks as proposed by Jaeger et al. [45]. In search for a computational more efficient method, they developed a hybrid optimization algorithm [51]. Typically this approach first performs a global search based on evolutionary strategy followed by a local search. With this method, another 91 circuits were obtained that fitted the experimental dataset accurately. Recently, Ashyraliyev et al. [52] showed that the circuits obtained by Fomekong-Nanfack et al. can be further improved using Levenberg-Maquardt.

The gene circuit method previously used [40,42,43,51] does not make any assumption about the network structure. All gene-to-gene interactions are assumed to be plausible and minimizing the difference between observation and simulation drives the inference. The criterion for selecting acceptable circuits was based on a low root mean square error (RMS) and visual inspection of the simulated gene expression profiles (no major pattern defects noticeable). In the current case, a large set of circuits can be used to simulate the A-P patterning of the *Drosophila melanogater*. Using a local sensitivity analysis, determinability of some parameters were studied in [52], where it was suggested that one could confirm on the nature of some biological interactions for parameters that were shown to be identifiable. Using a large set of parameters as base value, it is possible to examine the robustness of the model. Zak et al. [23] showed how input perturbations and stochastic gene expression influence the identifiability of a specific regulatory network given gene expression profiles and prior structural knowledge. Using as starting point the parameters-set obtained in [42,51] as base value, we discuss how does input perturbation and stochastic simulation allow a model-based robustness analysis of a model that leads to multiple circuits.

## Results

### Gap gene circuits

**Figure 1:**
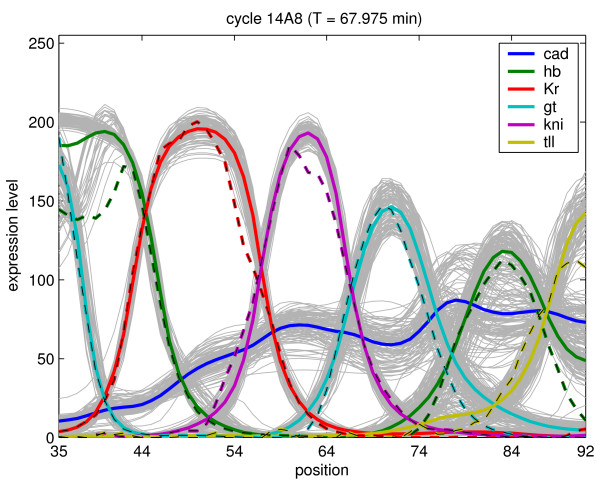
**Simulated expression profiles of the 101 gap gene circuits at the final time points**. The x-axis corresponds to 35-92% of the A-P position and the y-axis describes the expression level in fluorescence units. The experimentally measured expression profiles are plotted with coloured dashed lines. They represent average quantitative gene expression data obtained from fluorescent immunohistochemistry stained *Drosophila *blastoderm embryos [95] followed by successive image-processing operations [46,96]. The simulated profiles obtained from different circuits for each gene are shown in light grey and the average profiles at a specific time point are plotted using coloured solid lines.

**Figure 2:**
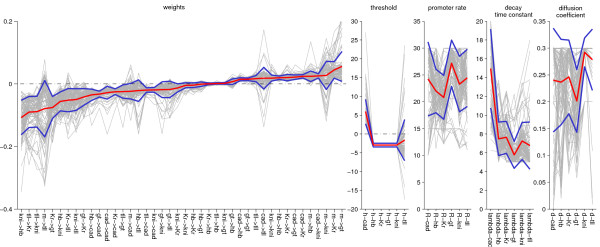
**Distribution of the 66 parameters obtained from the 101 gene circuit**. The solid red line represents the average value, the solid blue lines represent the standard deviations and the light grey lines are the different individual circuit parameters. Parameters are sorted according to their mean value. Most parameters show a strong tendency to cluster around a particular value, defining the type of interactions. However, some of them have a very broad distribution around their mean and in a few cases, they show all different types of interactions i.e. activation, repression or no interaction.

The analysis is based on 101 circuits each characterized by 66 parameters obtained from [42,51] using different optimization techniques. All circuits with a RMS value smaller than 12 (expression level measured in units of fluorescence level) were labeled as good fits and were selected for further analysis. In Fig. 1, the simulated expression patterns at gastrulation time are shown. The profiles (light grey) and the average profile (colour solid line) for each gene are shown together with the real data (dashed line). All circuits reproduce the gene expression patterns without any major defects (mainly amplitude variation). Although the profiles are well defined, the circuit-parameters show a rather different picture. In Fig. 2, the distribution of the 66 parameters is shown. The grey line represents the different 101 values of each circuits and the blue line their average. From this figure, we see that the distributions vary from parameter to parameter. None of the non-regulatory parameters such as the production rate, the diffusion coefficient or the decay time constant seems to be very consistent from circuit to circuit. In many cases, the promoter rate and the diffusion hit the upper boundary. Many parameters show a strong tendency to cluster at a specific value or location (specifically those around zero such as ,  or ). In some cases, we see a very broad distribution around the mean, especially for the parameters describing strong repression. In other cases regulatory parameters show both positive and negative interactions (*Bcd*_*tll*_, , *Bcd*_*kni*_), representing different network topologies.

### Robustness towards fluctuations

Gene transcription, degradation and diffusion inherently are stochastic processes, which lead to fluctuations in gene expression levels. If expression levels are high, these fluctuations generally do not affect the time evolution of the system. In these cases the deterministic model and stochastic model will yield a similar result. However if the system is non-linear and the fluctuations occur at an early time in development, where levels are still low, fluctuations may lead to different patterns. From a biological point of view this may not be favorable because gene pattern defects can lead to aberrations in development, hence development should be robust towards modest transient disturbances [53,54]. Here we investigate if the circuits obtained from the optimization, which are based on a deterministic model, are robust towards fluctuations.

Analysis of fluctuations in the bicoid gradient [55] suggests that the expression level of bicoid is about five fold higher than the fluorescence unit. In our analysis we assume that the expression level of all genes is five fold higher than the fluorescence unit. We have used the Gillespie algorithm [56] to introduce fluctuations in the gene expression levels.

For each circuit we performed 100 stochastic simulations (see methods) and analyzed the quality of the final expression pattern at *T *= 68.1 min. In each run, by comparing the deterministic model with the stochastic model using a RMS cut-off criterion, we counted how many of domains *cad, hb*-anterior (*hba*), *hb*-posterior (*hbp*), *Kr*, *gt*-anterior (*gta*), *gt*-posterior (*gtp*), *kni *and *tll *were considered to be correct. For each circuit we also calculated an overall score by counting the runs where all expression domains formed correctly [see Tab.1 in Additional file 1, and Additional files 2, 3 w]. On average the domains Cad (99%) and Hb-anterior (93%) show the best scores. The domains of *kni *(86%), *Kr *(80%) and *gt*-posterior (81%) have an intermediate score. The domains gt-anterior (66%), *hb*-posterior (70%) and particularly *tll *(47%) have a low score. Most circuits have a very low overall score; only the top 25% of the circuits have an overall score higher than 35%. Most of the circuits have a score lower than 20%.

If all domains except one develop correctly the overall score is determined by that domain score, however if two or more independent domains have scores lower than 100%, the overall score will be lower than the individual domain scores. For example, if all domains would have a score of 99% and are independent the overall score would be 0.99^8 ^* 100% = 92%. However we also observe interactions between domains, which leads to a higher overall score than would be expected if they behave independently. In these cases, if one domain yields a low score for a particular run, it is likely that another domain also yields a lower score for the same run. This is typically observed between adjacent domains. Notable examples are *Kr *with anterior *gt*, anterior *hb *with anterior *gt*, *kni *with posterior *gt*, and posterior *gt *with posterior *hb *and *tll*.

**Figure 3:**
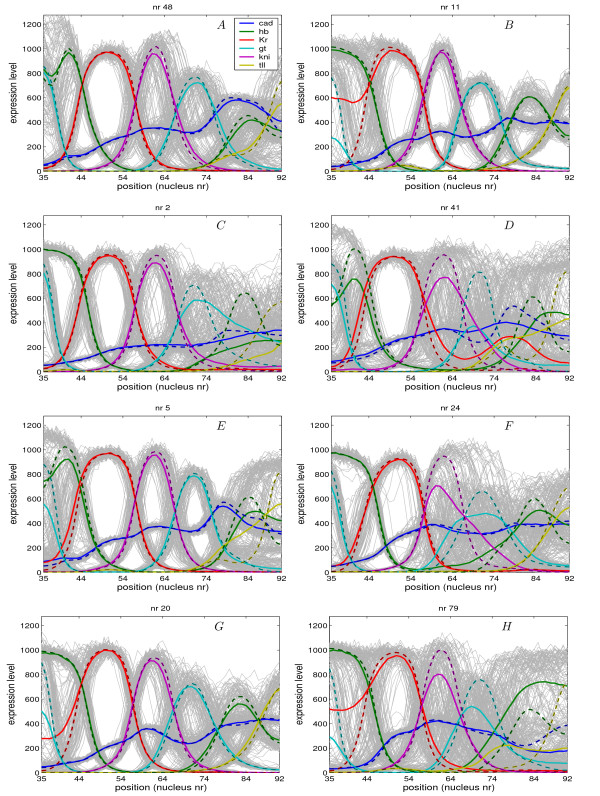
**Stochastic simulation of eight different circuits**. In these graphs the dashed lines represent the final profiles obtained from the deterministic model and the solid lines the average profiles obtained from the stochastic simulations calculated from the 100 individual stochastic runs, which are shown in grey.

**Figure 4:**
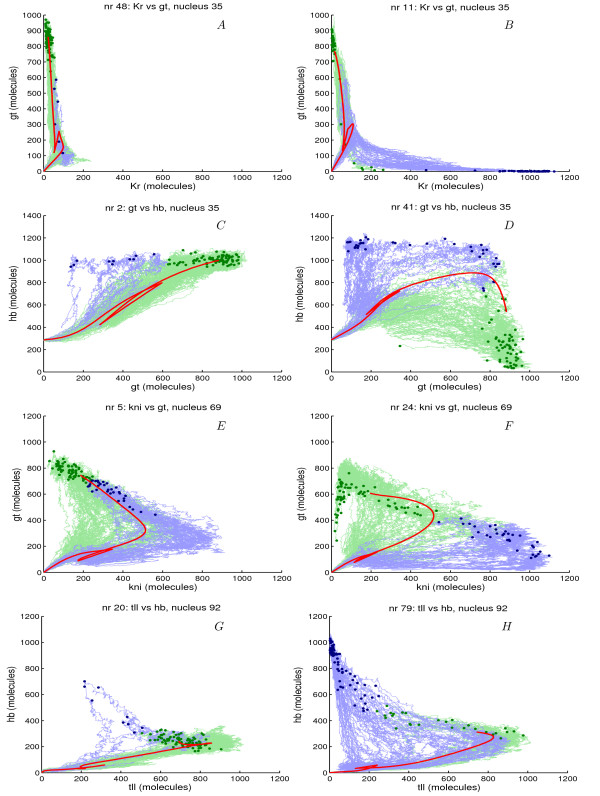
**Phase portrait of stochastic simulation at a particular nuclear position**. The time-evolution of the expression level of different gene combinations at a particular nuclear position is shown for the same circuits as in Fig.1. For comparison, the deterministic model is plotted using a solid red line and all stochastic runs (100) with light coloured lines. At the end of these lines a dot is plotted, which represents the final concentrations at T = 68.1 min. These end points were used to extract two clusters (with k-means clustering), which are represented by blue and green colours. The left panels show circuits with final points and pathways very close to deterministic model and the right panels show less well defined circuits.

In Fig. 3 the patterns of eight different circuits are shown. In these graphs the dashed lines represent the final profiles obtained from the deterministic model and the solid lines the average profiles obtained from the stochastic simulations calculated from the individual stochastic runs, which are shown in grey, [see movies in Additional file 4,5,6,7 where stochastic simulation of some circuits are shown]. In Fig. 4 the time-evolution of the expression level of different gene combinations at a particular nucleus position are shown for the same circuits as in Fig. 3. For comparison, the deterministic model is plotted using a solid red line and all stochastic runs (100) using light coloured lines. At the end of these lines a dot is shown, which represents the final concentration at *T *= 68.1 *min*. The end points were used to extract two clusters (with k-means clustering), which are represented by the blue and green colour.

In Fig. 3A, B the final pattern formed during the stochastic simulations are shown for circuit 48 and 11. Circuit 48 has an overall pattern score of 62% and nr 11 has an overall score of 34%. In circuit nr 11 all domains except *Kr *(44%) and anterior *gt *(35%) score 100%. *Kr *and anterior *gt *show a strong interaction, if anterior *gt *disappears then *Kr *expands into the anterior *gt *domain. In circuit 48 this interaction between Kr and Gt at the anterior of the embryo is not present. In this circuit especially *tll *is not well defined. Fig. 4A-B shows the trajectory of *Kr *and *gt *at nucleus 35, for circuit 48 in almost all runs *gt *and *Kr *develop correctly, however in circuit 11 there are two possible outcomes of the stochastic run. Here *gt *and *Kr *can develop correctly, however *gt *may also disappear and completely repressed by Kr. In this circuit there are two pathways for the system to evolve, which may lead to two stable points or a single stable point but two pathways. Jaeger et al. [45] suggested that non-overlapping gap gene are mutually exclusive and have mutual repression. This result was confirmed for *gt *and *Kr *by Ashyraliyev et al. [52]. Circuit 48 and 11 both show this strong mutual repression but we believe that the bad stochastic score of anterior *gt *and *Kr *might be caused by the weak repression of *gt *by Tll.

In Fig. 3C, D the final pattern formed during the stochastic simulations are shown for circuit 2 and 41. Both circuits have a very low overall score of 12% and 0% respectively. In circuit 2, a very low score for *hb*-p, *gt*-p and *tll *mainly causes this and for circuit 41 all domains except *cad *are not well defined. In Fig. 4C, D the trajectories at nucleus 35 of *hb *and *gt *are shown. For circuit 2 the pathway is well defined, in most runs both *gt *and *hb *evolve similar to the deterministic model. However in circuit 41 the pathways are not well defined at all and show variability. In circuit 2, we see that Hb represses *gt *while Gt weakly activates *hb*, and inversely for circuit 41. It is suggested that overlapping gap genes do not activate each other [45,52]. The poor robustness of these 2 circuits is therefore a consequence of the wrong connectivity of these 2 interactions.

In Fig. 3E, F the final pattern formed during the stochastic simulations are shown for circuit 5 and 24. Circuit 5 has a very low score of 10% and circuit 24 a score of 40%. Although circuit 5 has a low overall score both the *kni *and posterior *gt *domain score 100%, circuit 24 has a lower score for these domains. In Fig. 4E, F the trajectories are shown for *kni *and *gt *at nucleus 69. In circuit 5 the final points of the stochastic runs are very close to the final point of the deterministic run. Although the final points are well defined, the pathway, which is a shift of both domains, to these points is quite variable. In circuit 24 the same phenomenon is observed, only here Kni in some cases completely suppresses gt. Both circuits show *gt *activation by Kni (weak), which seems to be a wrong interaction [45,52]. Circuit 5 shows repression of *hb *by Gt and weak activation of *gt *by Hb while circuit 24 shows the inverse mechanism. Based on the identifiability analysis obtained by Ashyraliyev et al. [52], it is suggested that Gt does not repress *hb *and Hb does not regulate *gt*. However, the determinability of these parameters has a very poor confidence and qualitative conclusion on these interactions is still ambiguous.

In Fig. 3G, H the final pattern formed during the stochastic simulations are shown for circuit 20 and 79. Circuit 20 has an overall score of 50% and circuit 79 has a very low score of 2%. In circuit 20 all domains except *Kr *and anterior *gt *are well defined. In circuit 79 all domains except *cad *are not well defined. In Fig. 4G, H the trajectory of *hb *and *tll *at nucleus 92 are shown. In circuit 20 most final points are very close to the deterministic model, however in circuit 79 almost all points are far away from the deterministic model. In this circuit *tll *domain is repressed by Hb and completely disappears and the *hb *domain continuous to grow. In some runs in circuit 20 (blue trajectories) we see the same tendency of continuous *tll *repression combined wit *hb *increase. Circuit 79 shows very inconsistent regulatory mechanism with respect to available literature [31,32,57,58]. *gt *and *hb *show mutual activation, and Kni activates *kr*. These interactions seem to be the wrong regulatory mechanism, leading to stochastic instability and a very low pattern score.

### Correlation between robustness and parameters

**Figure 5:**
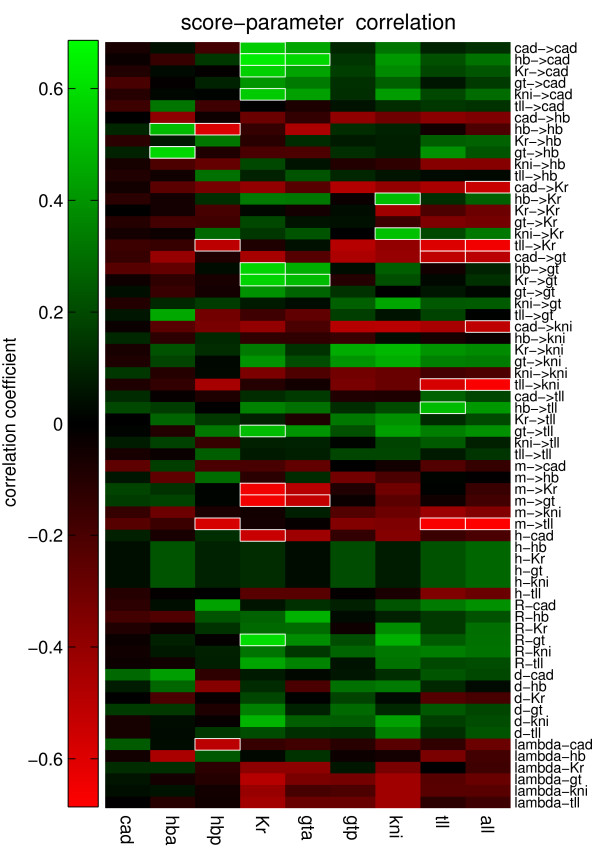
**Correlation between the score of the eight different expression domains obtained from stochastic simulations and all parameters**. Bright green represents a strong positive correlation and bright red a strong negative correlation. Squares in white borders are the most significant correlations.

In Fig. 5 the correlation between the eight different expression domains scores and all parameters are shown. The correlation pattern reveals that circuits with higher promoter rates, diffusion coefficients and higher degradation rates are more robust towards fluctuations. This suggests that higher rates tend to increase robustness of the circuits. Except for  all maternal inputs correlate negatively with pattern robustness. This suggests that strong maternal inputs tend to reduce the robustness of the circuit. All negative inputs on *cad *show a positive correlation, which suggests that strong Cad input weights tend to reduce robustness. Furthermore, the inputs of Cad on all genes except *tll *show a strong negative correlation, hence strong weights reduce robustness [In Fig. 1 Additional file 1, the correlations is shown separately for circuits with promoter threshold *H *= -2.5 and *H *= -3.5].

### Robustness towards parameter perturbation

If a parameter in one of the circuits (considered as a model input) is perturbed by a certain amount the formation of the gene expression pattern (considered as the model output) will be perturbed as well. The amount by which a parameter can be increased and decreased before a certain pattern error (RMS) is reached can be used as a measure for pattern sensitivity with respect to that parameter. In order to get a better understanding of the model's sensitivity, for each circuit and each parameter in that circuit the sensitivity interval (SI) was calculated (see methods). From a biological point view pattern formation should be robust towards small perturbations in the parameters [59].

**Figure 6:**
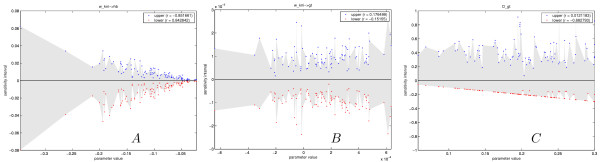
**Distribution of sensitivity interval versus parameter value**. (A) Plot of  sensitivity interval. Here both the upper and lower sensitivity interval scale with absolute parameter values. Hence the parameter becomes more sensitive when it is smaller in magnitude. (B)  sensitivity shows non-scaling behaviour, which is also observed for most other parameters. Furthermore, circuits with very similar parameter values can have very different sensitivities; this was also observed for most parameters. (C) Diffusion sensitivity illustrated by *D*_*gt*_. For most circuits the lower value for the diffusion coefficient reaches zero, hence the lower sensitivity interval is equal to the parameter value, meaning that without diffusion the pattern still forms correctly.

In Fig. 6C SI versus parameter value for *D*_*gt*_,  and  are plotted. For most circuits the lower value for the diffusion coefficient reaches the value zero, hence the lower bound of SI is equal to the parameter value, meaning that without diffusion the pattern still forms correctly. This phenomenon is also observed for all other diffusion coefficients. Furthermore, the upper bound of SI does not scale with the parameter value; hence circuits with very similar diffusion coefficients can have very different SIs. Non-scaling behavior is observed for most parameters, another example is  shown in Fig. 6B. For this parameter, both the upper and lower bound do not scale with parameter value, also here we observe that circuits with very similar parameter values can have very different SIs; this was observed for most parameters. For some parameters the SI does however scale with parameter value. For example all decay time constants show scaling, but also some weights. Fig. 6A shows the plot of , here both the upper and lower bound of the SI decreases with smaller absolute parameter values. Hence the circuits become more sensitive towards perturbations when the parameter is smaller in magnitude [see Additional file 8 where all parameters SIs are shown].

**Figure 7:**
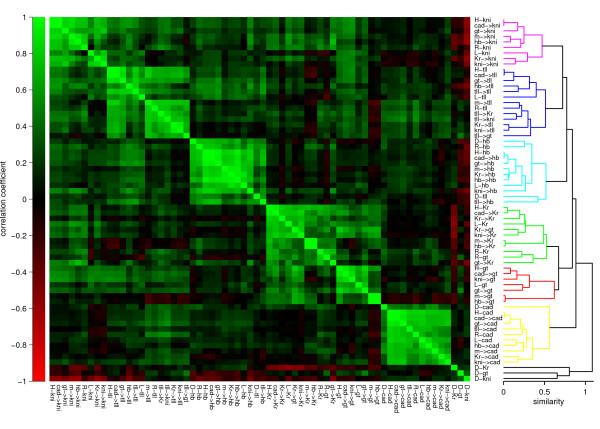
**Parameter sensitivity intervals correlation**. The correlation pattern is calculated on the basis of sensitivity intervals. The correlation matrix shows blocks on the diagonal, where circuit parameters regulating one particular gene tend to cluster together.

In Fig. 7 the correlation matrix, calculated on the basis of the SIs is shown. The correlation pattern shows blocks on the diagonal, where circuit parameters that regulate one particular gene tend to cluster together. For example, in the case of *cad*, if in a particular circuit a parameter that regulates *cad *has a large SI, it is likely that the other parameters that regulate *cad *also have a larger SI. This suggests that sensitivity appears to behave in a modular fashion, where the genes represent modules. One exception is *Kr *and *gt*, which both are found in the same cluster, suggesting these two genes have a strong correlation. This correlation is caused by their strong mutual repression [52], forcing them to act in similar way to perturbation.

### Circuit sensitivity

**Figure 8:**
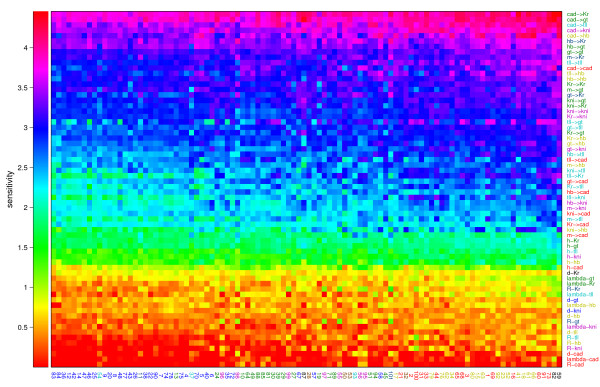
**Relation between parameter sensitivity intervals and circuit sensitivity**. Intensity plot of the sensitivity of all parameters and all circuits sorted according to the average parameter sensitivity and average circuit sensitivity. The most sensitive parameter and most sensitive circuit are shown at the top right.

**Figure 9:**
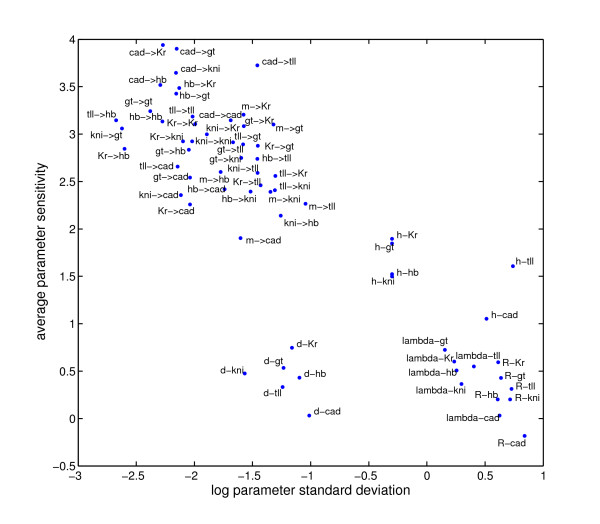
**Relationship between average sensitivity interval and parameter standard deviation**. On the y-axis,-log *SIs *is given and on the x-axis parameter standard deviation. The figure shows that there is no obvious correlation between SI and STD. The parameter identifiability is not necessarily linked to SI.

To investigate which parameters and which circuits are more sensitive than others we have calculated the average sensitivity on a logarithmic scale for each parameter and each circuit (see methods). Then the SI of each parameter value in each circuit was plotted using an intensity plot, where the colour corresponds to:-log *SI*. The parameters and circuits were ordered in Fig. 8 according to their average sensitivity, showing the parameters with smallest SI and most sensitive circuit at the top right corner. From Fig. 8 it can be seen that the difference between the smallest and largest SIs are 4 log units. The circuits are less sensitive with respect to diffusion coefficients, promoter rates, decay time constants and promoter threshold. Circuits are most sensitive with respect to  weights followed by the auto-regulation weights . Because the different parameter types (diffusion, decay, promoter rate, thresholds and weights) are not on the same scale we also calculated the average relative sensitivities of the circuit parameters [see Tab. 2 in Additional file 1]. This approach can however be problematic, if the parameter values are close to zero, possibly yielding a very high relative sensitivity. Therefore these outliers were removed from the analysis. Using this measure, the least sensitive parameters are the diffusion coefficients with a relative sensitivity of about 100-200%. The thresholds are now more comparable with the weights and have a relative sensitivity of about 1%. Furthermore the  weights are still amongst the most sensitive parameters with a relative sensitivity of about 0.5-1%. From Fig. 9 we see that the average sensitivity of the circuits varies from one to another. Certain parameters in the least sensitive circuits, notably weights related to *tll *and *cad *tend to have lower sensitivities. By comparing the least sensitive circuits with the more sensitive circuits using a t-test several parameters where found that are significantly different in these groups. These are in fact the parameters that show scaling between sensitivity and parameter value , , , , , ,  and .

The standard deviation of the circuit parameters can be quite large compared to the average parameter value shown in Fig. 2. And furthermore, average parameter value and standard deviation strongly correlate (*r *= 0.81). In Fig. 9 the average -log *SIs *versus standard deviation is plotted. Amongst similar parameter types (weights and non-weights) no correlation is observed between SI and standard deviation. This suggests that if a parameter is very variable across the circuits, i.e. it is not well-defined [60], it is not necessary less sensitive. A notable example is  and , which both have a similar standard deviation but differ in sensitivity by 2 log units. Furthermore, we also observe that the standard deviation on average is much higher than the sensitivity interval.

### Model sensitivity vs. pattern robustness

**Figure 10:**
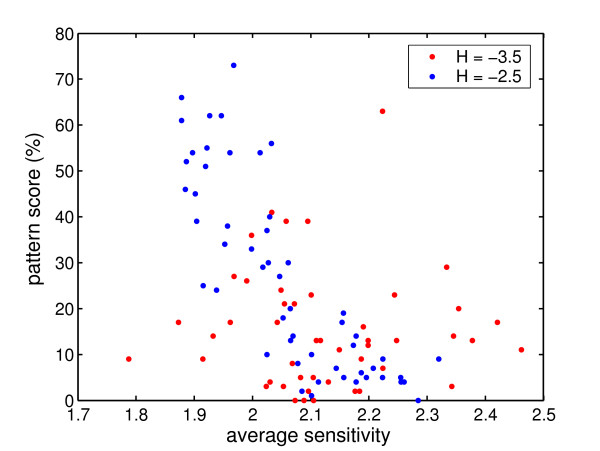
**Scatter plot of pattern scores versus average circuit sensitivity**. On the x-axis, average sensitivity of a circuit is plotted and on the y-axis the overall pattern score as percentage, which was obtained from 100 stochastic simulation runs. This figure shows that sensitive circuits are not robust towards fluctuations.

In Fig. 10 we have plotted the average sensitivity versus the overall score of the circuit. In this figure the circuits with promoter threshold *H*_*hb*,*Kr*,*gt*,*kni *_= -2.5 are shown in blue and the circuits with *H*_*hb*,*Kr*,*gt*,*kni *_= -3.5 in red. The circuits with an average sensitivity higher than 2.05 are significantly less robust towards fluctuations than the circuits with an average sensitivity lower than 2.05. The circuits with *H *= -3.5 are more sensitive than the circuits with *H *= -2.5. Furthermore the robust circuits have a better overall pattern score on average; however lower sensitivity does not necessarily yield a good pattern score. This suggests that the average sensitivity is not the only determinant for robustness towards fluctuations. In Fig. 2 of Additional file 1, the correlations between parameter SIs and stochastic simulation of individual gene domain robustness is shown separately for circuits with promoter threshold *H *= -2.5 and *H *= -3.5.

## Discussion

The 101 gap gene circuits were previously obtained with inverse modelling [42,51] of a seven gene (*bcd, cad, hb, Kr, gt, kni *and *tll*) connectionist model using detailed spatio-temporal gene expression data [61]. All circuits were selected only on the basis of a low RMS value (*RMS *≤ 12), and in all cases the circuits were able to reproduce the dynamics and the patterns accurately. However, the parameter values of the circuits appeared not to be well defined, for some parameters multiple clusters were found, which represented multiple circuit topologies. Furthermore many parameters showed a single cluster with a high degree of scattering around the mean as shown in Fig. 2. Before any further tests, this result insinuates that for some parameters such as the non-regulatory parameters (showing major scattering), the notion of a global optimal parameter set makes no biological sense as suggest by Von Dassow et al. [27]. Also, the scatterings of most of the regulatory parameters are almost in a precise region of the search space, describing a precise qualitative behavior that can be linked to a potential interaction (repression, no-interaction or activation). The model always performs well and leads to very good pattern suggesting that the model is robust to the network topology as well as variation in individual parameters. Robustness to network topology is a behavior already observed by Von Dassow et al. [27] in the case of the segment polarity of Drosophila melanogaster.

To further analyze the properties of the model and to classify the quality of the circuits we conducted a perturbation analysis. We analyzed the robustness of 101 *Drosophila melanogaster *gap gene circuits using two different approaches, first the robustness of pattern formation towards intrinsic fluctuations in gene expression levels was investigated using a stochastic model and secondly the sensitivity of the circuits with respect to the parameters in the model was investigated using a simple parameter perturbation technique.

### Robustness towards fluctuations

Robustness of the regulatory network is expected to be a fundamental property of the network, pattern formation should therefore not be compromised by small intrinsic fluctuations in gene expression levels. Therefore, the patterns obtained from stochastic simulations should statistically be similar to the pattern obtained from the deterministic simulation for robust circuits.

### Robustness of domain formation

**Figure 11:**
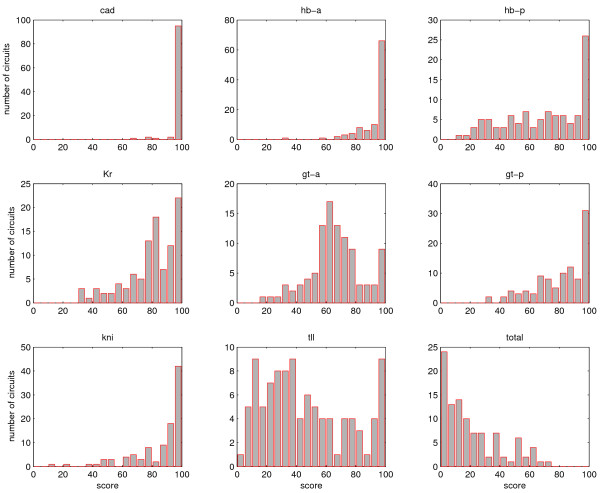
**Robustness of overall patterns and domain formation**. Each panel beside the last one shows the distribution of the circuits' robustness to gene expression fluctuation. Genes that are expressed in more than one domain (such as *hb *and *gt*) have more than one score. The score determines how precise is the stochastic simulation to the deterministic simulation. For each circuit, 100 stochastic simulations were run and score for individual gene and total pattern was calculated. On the x-axis the score is given and the y-axis determines the number of circuits with a given score. Cad and anterior Hb (hb-a) show very good score while Tll shows a very bad score.

Introducing fluctuations in the model had a profound effect on the formation of the expression patterns. Although some of the circuits showed robust formation for most domains, none of the circuits were sufficiently robust when looking at the formation of all the domains. We observe that fluctuations can lead to an increase or decrease of domain amplitudes (see Fig. 3). Furthermore we also observe posterior and anterior boundary shifts, which leads to domain expansion, domain contraction or domain shifts. We also observe that domains completely disappear or that domains appear in other regions, where they repress other domains. Especially in a significant number of circuits the anterior domains Gt and Kr did not form robustly during the simulations. The least robust was the Tll domain at the posterior end, this domain interacted with Gt and Hb, which all showed defects in many circuits. We believe that Tll weak robustness is caused by an incomplete model where gap gene are regulating *tll *which is inconsistent with experimental evidence [45,62]. However, we see that although the overall robustness to fluctuation is poor, the robustness tends to behave in a modular way, where, for a given circuit, all genes but one can have very good score. The gene with a bad stochastic simulation can be linked to a bad or wrong interaction, feature that cannot be seen from the deterministic simulation. This modular behavior shown in Fig. 11 is strongly related to the network topology as well as the interaction weights.

Looking at the evolution of the system in phase space, we found that the pathways in many cases were not well defined, which appeared to be linked to the existence of multiple attractors. The fluctuations allowed the system to jump from one attractor to another, causing the system to evolve into a pattern very different compared to the deterministic model. This suggests that by using the reverse engineering approach many circuits can be found, which are well defined for the deterministic case but not for the stochastic case. Multiple attractors can easily occur in a non-linear model with many parameters, where overfitting may lead to many connections in the circuits. Also the type of feedback loops may be crucial, e.g. cad has a negative auto-regulation, which reduces the effect of fluctuations, however all other genes have strong positive auto-activation [63,64]; in these cases fluctuations are amplified if there are no negative feedback loops associated with the increase of the expression level.

### The effect of certain parameters on robustness

The connectionist model contains a promoter threshold for each gene, which determines if gene production is on or off when there are no other inputs. In the current gap gene model the production of *hb, Kr, gt *and *kni *can only be turned on by maternal inputs or by auto-activation, therefore the promoter threshold are set to a fixed negative value, which was either *H *-3.5 or *H *= -2.5. Looking at the robustness of individual circuits, we find that circuits with promoter threshold set to *H *= -2.5 are more robust towards fluctuations compared to circuits with promoter threshold set to *H *= -3.5, and also their parameters are on average less sensitive. In general we find that weaker maternal inputs from bicoid and caudal increase the robustness with respect to fluctuations. Furthermore higher promoter rates, decay rates and diffusion coefficients improve the robustness towards fluctuations.

We have seen that circuits are relatively less sensitive with respect to diffusion coefficients, and with some extent, to decay time constant. Regarding the diffusion coefficient, this result confirms observations made elsewhere where it was suggested that the diffusion is not essential to explain precise gene expression pattern formation [42,65], but the diffusion term does account for the boundary nuclei effect [66]. It was also shown by Nusslein-Volhard et al. [67] that the effect of diffusion is reduced with the exponential increase of the number of nuclei. Also, Gregor et al. [68] showed that diffusion coefficient does not scale with varying embryos length. The rest of the parameters have a mixed sensitivity behavior and do not fall into a specific category [see Tab. 2 in Additional file 1]. Some of the weights are very sensitive and some are not, idem for the production rate. Although we do not have precise biological explanations regarding the difference of the sensitivity behavior of the parameters, it might be interested to experimentally investigate if such difference is a property of the connectionist model or a characteristic of the regulatory mechanism. The sensitivity information can then guide the selection of the optimal mutation targets and thereby reduce the experimental effort. This validation could be done for example by measuring mRNA degradation rate of zygotic Hb in embryos with over expressed maternal *hb*, or by measuring the binding affinity in mutants. Also one could consider inducing genetic mutation to control kinetic parameters that can be measured [69].

We also find that certain parameters that regulate posterior *tll *and also some parameters that regulate anterior *gt *and *Kr *affect robustness of the corresponding domains. Furthermore we find that robustness and parameter sensitivity are linked. Especially for the group with a lower promoter threshold we find that circuits with a lower average sensitivity are more robust towards fluctuations. These results show that in the current gap gene model not just the network topology but to a large extent the precise value of the parameters determines robustness. We noticed that inputs of *cad *to other genes correlate negatively with robustness, these parameters are amongst the most sensitive in the model, with a - log *SI *in the order of 4. If we assume that fluctuations are proportional to the square root of the concentration level (this is a reasonable assumption for steady production) the relative fluctuation level is about , where *n *is the number of molecules. For *n *= 100, *n *= 1000 and *n *= 10000 the relative fluctuation level then is 10%, 3.2% and 1%. The most sensitive parameters in the model have a relative sensitivity of about 0.5 - 2% [see Tab. 2, Additional file 1]. This holds assuming that 20% of the circuit RMS is a critical cut-off.

### Possible reasons for weak robustness

We conclude that the current connectionist model describing the gap gene segmentation obtained from reverse engineering techniques is not robust. Although some circuits appear more robust than others, most of the circuits are extremely sensitive towards gene expression level fluctuations and also pattern formation is very sensitive with respect to perturbation of a large number of parameters. In most cases the parameters causing this are linked to: Tll regulation, in some cases are linked to anterior Gt and Kr regulation and finally the promoter threshold (see Fig. 5). There may be a multiple reasons for weak robustness. First, the incompleteness of the model may be the cause. In the real system it is known that the terminal pathway with Tll, huckebein [70] and Torso also regulate gap genes, the latter two are missing in the current model. Furthermore, hb regulation is not only zygotic but also has a maternal component at the anterior end of the embryo that is also regulated by nanos, nanos and a maternal description *hb *mRNA are missing in the model. Furthermore, only a part of the embryo is considered, which may lead to boundary effects at the anterior end. Secondly, the current reverse engineering approach using a simple RMS optimization may lead to sensitive circuits, not only some time points are missing in the data also features in the data that are not represented in the model may be over fitted. Finally, the rather phenomenological approach of the connectionist model may also be a source of sensitivity because promoter threshold has a profound effect on robustness.

### How to improve the network inference

In modelling processes from developmental biology especially the class of quantitative spatio-temporal models of gene regulation is relevant. This type of models can potentially linked with three-dimensional biomechanical models of morphogenesis and provides new insights into developmental biology. Especially the quantitative spatio-temporal models of gene regulation are characterized by a large number of unknown parameters and an (infinite) class of potential solutions. So far, very few model-based analysis method have been proposed to validate or invalidate models, especially for nonlinear and spatially distributed models [6,71,72].

The analysis presented in this paper shows the effect of missing genes, suggesting that it might be necessary to include more genes known to be involved in gap gene segmentation. Although data might not be available, one could consider combining synthetic data with observation. The hierarchical modular structure of the system should be considered within the model description [27]. Phenomenological models such as the connectionist model can generate realistic pattern, but they still fail at establishing the clear role of genetic perturbation. These models can be improved by considering using Hill type functions [27] and/or more detailed rate equations [65]

Another improvement would be to infer the network using stochastic models that translate the fluctuations in the system. It is computationally expensive to numerically solve such systems and it will require efficient optimizations methods [73].

To prevent overfitting, one might consider reducing the number of free parameters by adding more constraints known from previous experiments. In general the inverse problem that is only based on minimizing the RMS leads to over-fitting, allowing for many solutions that all fit the dataset equally well but may not show correct behavior or properties beyond the dataset. It is therefore difficult to know, which solution is most similar to the real system. The different post-optimization analyses presented in this paper and in an accompanying manuscript ([74]) revealed some simple but efficient invalidation tests. Ideally, we would like to funnel all circuits into a pipeline of tests where the circuits are filtered and those passing all the tests are the robust and stable solutions. It may however turn out that none of the solutions will pass all the tests. Therefore we propose not only to minimize the RMS but also to incorporate other objectives into the optimization strategy. By introducing multiple objectives the solutions obtained should possess better properties and also have better defined parameters. The possible additional objectives for gene regulatory networks are:

• **Sensitivity constraints **should lead to networks that are robust towards parameter or input perturbation. This could simply be addressed by incorporating a sensitivity analysis within the optimization procedure. Using the Levenberg-marquardt method for parameter estimation, Ashryalyev et al. [52] have investigated simultaneously while estimating the parameters, their identifiability. Rodriguez-Fernandez et al. [75] have presented a hybrid method that can handle the sensitivity analysis while optimization. Using such method, we suggest attributing to each circuit a sensitivity value that determines the overall sensitivity of its parameter.

• **Noise robustness constraint **is another improvement that should lead to circuits robust towards fluctuations (internal or external). This improvement can be achieved by inferring the network using stochastic models that include the fluctuations in the system. It is computationally expensive to numerically solve such systems and it will require efficient optimizations methods [73,76]. Furthermore, It is known that patterning is insensitive to external fluctuation such as maternal gene expression. Consequently, an efficient model should also be able to simulate gap-gene expression given noisy external Bcd expression. An efficient way to distinguish all the solutions obtained from the parameter estimation would then be to change the Bcd dosage during the optimization It was shown by Manu et al. [77] that Bcd variation is not the cause of the precise canalization of the gap genes but its a consequence of the cross regulation between zygotic gap genes [29]. In an optimization procedure, good circuits should be able to reproduce the patterns as well as the shift without showing any major fluctuation of the gap gene expression while varying the Bcd concentration.

• **Reducing network connectivity **In our accompanying manuscript [74], we show that the long term dynamic revealed that in the presence of certain motifs, the circuits pattern converge to oscillatory attractors. This behavior is not desirable as we expect the patterns to converge to a stationary point being the steady state. Therefore, we would ideally ignore or penalized the circuits having this behavior. Also, we have shown that circuits with realistic topologies do not have these motifs and converge to the desired attractors. This could be obtained by either running the circuits beyond the data set to force asymptotic stability [66], or by adding an entropy function to prioritize circuits with the minimal connectivity. It was shown by Isalan et al. [78] that circuits where reduced connectivity are more robust to parameter variation in comparison to circuits with less connected members.

These different additional objectives would be integrated in a multi-objective optimization framework, which is an extension of the classic optimization of a single-objective function (see Handl et al. for interesting review on Multi-objective optimization [79]). Many multi-objective algorithms exist [80,81] such as method where multi objectives are transformed into one single objective [82-84], Pareto [85,86] and non-Pareto dominance approaches. To our knowledge, very few have used Multi-objective optimization for GRN inference [87,88]. Based on recent reviews [9,10], stochastic ranking evolutionary strategy (SRES) is one of the best methods for parameter estimation of biological problems. This method has been used to obtain some of the circuits analyzed in this article and obtained elsewhere [51]. The implementation of the SRES is suitable for multi-penalties where the weighted sum of penalties is used [9] as a multi-objective constraints. The strength of the ES is its intrinsic parallel nature [89]. One alternative would be to use an island based ES where the number of island is determined by the different objectives. Each island minimizes the multi-objectives, but the distribution of the weight is different from island to island. Migration between islands will spread individuals with a particular strong property in the other islands. The main objective being the least square difference between the simulation and the data, it might be more practical to start with one population where the penalties have equal weights and later on, switch to islands once the LSE is acceptable. This would guarantee that in all islands, the individuals have at least a good RMS.

## Conclusion

In this paper, we have provided a robust analysis of a model used to infer the gap gene regulatory network of *Drosophila *melanogaster. The model has been extensively used elsewhere [42,43,51] to simulate and provide some explanations concerning the regulatory mechanism that leads to precise pattern formation. Unfortunately, many assumptions were based on a limited number of circuits obtained using simulated annealing and previous researchers assumed that the model was correct with a correct topology. In this article, we have shown that the mathematical model leads to different circuits all capable of reproducing the quantitative spatio-temporal gene expression pattern. Consequently, it is difficult to decide based solely on the architecture which circuit is the correct one.

Robustness towards fluctuation has revealed that the overall gap gene domain tends to be poorly resistant to perturbation and this weak property could be related to some particular interactions predicted by the circuits. Furthermore, parameter perturbation analysis has shown that the circuits with lower sensitivity do not necessarily yield to robustness to fluctuation. The reason for these few exceptions is related to the promotor threshold and to local domain robustness, which can considerably affect the overall global robustness. Overall, the analysis shows that the network possesses modular robustness and some local properties may affect the robustness of a gene expression locally as show in Fig. 11. This feature is strongly related to the network topology as well as the interaction weights.

From a biological point of view, this paper has shown that it is difficult to relate the connectionist model with biological evidence. The model ability to simulate the gene expression does not necessarily provide meaningful information since alternative networks are predicted. Therefore, it would be interesting to test some of the different alternatives, especially when there is not yet any experimental evidence to invalidate. However, it was recently demonstrated that it is still possible to draw qualitative conclusions on the regulatory topology of the gap gene network [52]. The overall analysis has shown that based on the robustness toward gene expression fluctuations and parameter perturbations, it is possible to identify robust circuits as well as the parameters that are identifiable according to their sensitivity intervals. For a computational/system biologist, this shows that it is essential to further analyze a model prediction, when results are obtained from reverse engineering based on parameter estimation, since some of its properties may or may not invalidate the results. We have also provided some preliminary suggestions to efficiently improve the GRN inference to avoid reverse engineering that leads to circuits with different topologies, by controlling the optimization by means of multi-objectives minimization.

## Methods

### Inference of the Gap Gene model

The gap gene circuits analyzed in this paper were presented by Jaeger et al. [42] and Fomekong-Nanfack et al. [51]. In both cases, the inference was performed using the same quantitative data, the same model description but different parameter estimation methods.

**Quantitative data **used are available online in the FlyEx database  or . The database presents a collection of spatio-temporal gene expression data obtained from fluorescently stained wild-type embryos for Eve protein and two other genes. Data were obtained by applying different image processing strategies [46]. The embryos are for different time ranging from cycle 7 to cycle 14A. In the simulation, data obtained at cycle 12 were used as initial conditions. For the genes *Kr, gt, kni, Tll *these are very close to zero and set to 0 in the simulations.

**Mathematical model **of gap gene considers the 35% to 92% of the A-P axis of an embryo. It is reduced to a one-dimensional discrete model where nuclei are aligned horizontally. The model focuses on the development between cycle 13 and cycle 14A8, before gastrulation (71.1 min). Three rules describe the mechanism during that phase: interphase, mitosis and division [90]. Interphase and mitosis are continuous stages describing the dynamic of protein variation of a gene within a nucleus. The division is a discrete process describing the cleavage of a nucleus in two. Mitosis, arising before division, differs from interphase by the absence of protein synthesis. The resulting model is a system of 180 equations before division and 348 equations after, with a total of 66 unknown parameters written as:

(1)

where *N*_*g *_denotes the number of genes or gene products involved and Φ is a sigmoid function with range (0,1). (*t*) represents the concentration level at time *t *of gene *a *in nucleus *i *with 1 ≤ *i *≤ *N *and *N *the number of nuclei during a cleavage cycle. The concentration, , of the maternal gene *bicoid *is taken from experimental observations and is kept constant in time during the simulation. The parameters are: the regulatory weight matrix , describing the influence of gene *b *on gene *a*, the production rate *R*_*a*_, the activation threshold *h*_*a *_for Φ, the decay rate *λ*_*a*_, the diffusion coefficient *D*_*a*_, and the regulatory influence *bcd*_*a*_.

**Parameter estimation **was performed by two different strategies. Jaeger et al. [42] have used a parallel simulated annealing (PLSA) algorithm as described in [48], originally proposed by Lam [49]. The expensive computational time required by PLSA could only lead to 10 gap gene circuits with good solution's quality and patterns behavior. Later on, Fomekong-Nanfack et al. [51] proposed 101 gap gene circuits obtained using hybrid methods composed of a stochastic ranking evolution strategy [91] followed by direct search [92]. Evolution strategy is a evolution based algorithm like genetic algorithm [93] and direct search is a local search strategy suitable to solve a variety of optimization problems that are not well suited for standard optimization algorithms, including problems in which the objective function is discontinuous, non-differentiable, stochastic, or highly nonlinear. This number of solution could be obtained because of the reasonably low computational time of their method (8 h on single processor) compare to PLSA (1 to 5 days on 10 parallel CPU), but leading to the same quality of solution. In both cases, the optimization goal is to estimate the unknown parameters that minimize a scalar valued *cost-function*, by exploring the set of possible values in an allowed search space. The chosen cost-function is the least squares of the difference of the simulated and the observed data:

(2)

with *θ *the parameter vector to which a constraint or penalty function is added. An explicit search-space constraint is given for parameters *R*_*a*_, *λ*_*a *_and *D*_*a*_. For the parameters , *bcd*_*a *_and *h*_*a *_a collective penalty function is used ([40]) to restrict the function value of Φ to the domain [Λ, 1-Λ] with Λ a small parameter (in this study taken to be 0.001). The root mean square (RMS) described by Reinitz et al. is used ([40]) as a measure of the quality of a model solution for a given set of parameters:

(3)

where *E*(*θ*) is given by Equation (2) and *N*_*d *_is the number of data points.

### Sensitivity analysis and confidence interval

**Figure 12:**
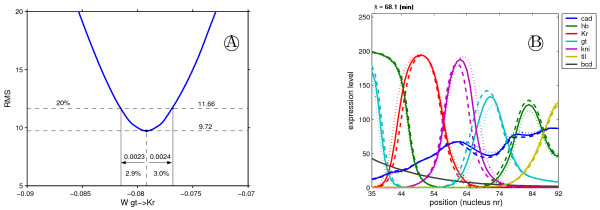
**Parameter perturbation**. For each parameter within each circuit, lower and upper parameters bound are calculated by tweaking the original value until the RMS is increased by 20%. (A) perturbation of the parameter  and in (B) simulated gene expression after parameter perturbation. The solid lines represent the original circuit's gene expression. The small dashed lines and big dashed lines are the profiles corresponding respectively to the lower and lower parameter bounds.

If a single parameter is slightly decreased or increased, the RMS of the simulated pattern will increase because the quality of the fit to the gene expression data reduces. The amount, or range, by which a parameter can be changed before the fit becomes significantly bad, is a measure for the sensitivity of the parameter for a particular solution. For each parameter within each solution we computed the lower and upper value of a parameter where the RMS increases by 20%, which corresponds to a situation where the gene pattern becomes significantly bad as shown in Fig. 12. Hence, the lower value *θ*_*n*,*i *_- Δ*L*_*n*,*i *_is computed from *f*(*θ*_*n*,*i *_- Δ*L*_*n*,*i*_) = 1.2 * *RMS*_*n *_and the upper value *θ*_*n*,*i *_+ Δ*U*_*n*,*i *_is computed from *f*(*θ *_*n*,*i *_+ Δ*U*_*n*,*i*_) = 1.2 * *RMS*_*n*_, where RMS is the original RMS value of the fit obtained from the optimization and *f *denotes the cost function, *n *denotes the solution number and *i *the parameter index. We define the sensitivity interval as [*θ*_*n*,*i *_- Δ*L*_*n*,*i*_, *θ*_*n*,*i *_+ Δ*U*_*n*,*i*_] and the sensitivity as . The average sensitivity of a solution and a parameter are defined as  where *np *is the number of parameters and respectively  where *ns *is the number of circuit. The lower relative sensitivity is defined as *θ*_*n*,*i*_/*ΔL*_, i _* 100% and the upper relative sensitivity is defined as *θ*_*n*,*i*_/*ΔU*,_i _* 100%.

### Stochastic behavior of the model

We implemented a nonlinear stochastic deterministic differential equation model of the gap gene where the dynamic is driven by Gillespie stochastic simulation algorithm [56]. The master equation is based on the differential equation describing the concentration change of a gene expression in a particular nucleus. Each equation describes three different reactions: protein production, protein decay and diffusion. Only one reaction can occur at a specific time. The Gillespie algorithm simulates the system by choosing first in a probabilistic manner which reaction occurs, and then estimates when does the next reaction will be realized. To speed the process, we have used a method [94] that only calculates and updates reactions that have changed. The probabilistic nature of the algorithm imposed us to run 100 simulations for each of the 101 gap gene circuits. All simulations used the same initial condition and the number of molecules is 5 times the deterministic case (based on [55]). For simplicity, analyses are only made on the final pattern (gastrulation time) by comparing the deterministic simulation with the stochastic one.

## Authors' contributions

All authors participated in the design of the study, and wrote the manuscript together. YF-N and MP conducted the analysis of data and simulations, and made the figures. All authors read and approved the final manuscript.

## Supplementary Material

Additional file 1 **Additional statistics**. This file (GapGeneModelRobustnessAddFile1.pdf) contains the material, which is not given in the paper due to the space limitation. It mainly gives additional statistical results for the stochastic simulations and the perturbation analysis. Click here for file

Additional file 2 **Stochastic simulation of all circuits**. The document (GapGeneModelRobustnessAddFile2.pdf) provided gives the simulation obtained from the 100 stochastic runs of each circuit obtained deterministically. Each page corresponds to a circuit and on each page; individual panels correspond to individual run. The panels with a grey background represent runs that have a defect in one or more expression domains. The runs shown in the panels with a white background are considered to be correct. Click here for file

Additional file 3 **Robustness of all circuits**. In this document (GapGeneModelRobustnessAddFile3.pdf), each figure shows the deterministic stochastic simulation of a circuit. In these graphs the dashed lines represent the final profiles obtained from the deterministic model and the solid lines the average profiles obtained from the stochastic simulations calculated from the 100 individual stochastic runs, which are shown in grey. Click here for file

Additional file 4 **stochastic simulation of circuit number 5 run 52**. The movie shows the spatio-temporal simulation of circuit number 5 for the 52 stochastic run. Click here for file

Additional file 5 **stochastic simulation of circuit number 5 run 83**. The movie shows the spatio-temporal simulation of circuit number 5 for the 83 stochastic run. Click here for file

Additional file 6 **stochastic simulation of circuit number 11 run 64**. The movie shows the spatio-temporal simulation of circuit number 11 for the 64 stochastic run. Click here for file

Additional file 7 **stochastic simulation of circuit number 11 run 98**. The movie shows the spatio-temporal simulation of circuit number 11 for the 98 stochastic run. Click here for file

Additional file 8 **Absolute parameters sensitivity intervals of all parameters**. The data provided in this document (GapGeneModelRobustnessAddFile8.pdf) represent the sensitivity interval of all the 66 parameters obtained from the 101 circuits. Each figure gives the upper and lower bound of the SI. Click here for file
