# Supplementary material for: Inferring Drosophila gap gene regulatory network: a parameter sensitivity and perturbation analysis
Source: BMC Syst Biol. 2009 Sep 21;3:94. doi: 10.1186/1752-0509-3-94 (PMC2761871; doi:10.1186/1752-0509-3-94)

Supplementary material for the paper

Inferring *Drosophila* gap gene regulatory network: a parameter sensitivity and perturbation analysis

Yves Fomekong-Nanfack [1], Marten Postma[1], Jaap Kaandorp [1,a]
[1]Section Computational Science, Faculty of Science University of Amsterdam.
Science Park 107 ,1078 XJ Amsterdam The Netherlands.
[a]corresponding author E-mail: J.A.Kaandorp@uva.nl

Additional statistical results

Table 1: Statistics on stochastic simulations of all circuits. The data provided represent the statistical analysis for each circuits,100 stochastic simulations were run. The score is calculated based on the overall pattern, but also the individual gene score. Genes expressed in two domains (such as hb) have two different scores. Circuits are ordered based on their global score.

nr	cad	hb-a	hb-p	Kr	gt-a	gt-p	kni	tll	all	nr	cad	hb-a	hb-p	Kr	gt-a	gt-p	kni	tll	all
7	100	99	94	93	88	92	96	94	73	60	97	87	100	96	78	77	89	30	14
12	100	100	98	84	67	100	100	100	66	31	100	99	78	69	61	82	64	22	14
80	100	100	98	94	72	99	100	83	63	27	97	100	57	91	56	89	54	71	14
48	100	95	100	100	98	96	100	67	62	95	100	98	98	46	39	88	92	27	13
26	100	100	95	87	77	97	100	80	62	94	98	92	72	81	48	65	91	48	13
36	100	100	99	76	61	100	99	95	61	92	100	98	46	90	69	74	94	31	13
87	100	100	97	97	60	100	100	100	59	73	100	86	77	83	92	66	73	21	13
35	100	98	100	95	97	82	92	75	56	68	100	97	33	93	76	83	94	33	13
8	100	100	92	82	71	96	98	92	55	29	100	98	58	89	63	87	99	25	13
47	100	100	93	78	70	80	99	98	54	65	100	95	28	96	83	65	98	51	12
22	100	100	95	80	56	100	98	99	54	2	100	100	37	98	98	94	70	20	12
4	100	99	96	79	65	88	97	96	54	101	100	100	85	54	68	100	100	76	11
14	100	100	89	84	68	100	100	80	52	59	100	70	99	77	96	71	76	27	11
20	100	100	100	80	52	100	100	95	51	50	100	99	69	84	67	58	85	38	10
42	99	99	89	84	60	99	97	79	46	5	100	90	82	96	64	100	100	20	10
46	100	68	100	100	97	100	100	65	45	83	100	97	78	59	61	34	62	64	9
72	100	99	92	68	61	77	87	100	41	76	100	98	100	70	100	46	72	21	9
24	100	97	83	96	92	64	93	71	40	54	100	83	86	76	83	53	78	33	9
81	100	99	84	81	72	85	95	75	39	18	99	88	68	99	75	89	97	15	9
71	100	100	62	81	67	100	98	97	39	1	100	94	72	68	50	91	70	32	9
25	100	100	78	84	62	83	96	86	39	99	100	82	66	33	42	49	49	42	8
38	100	94	74	87	78	59	95	100	38	28	99	99	46	63	57	69	79	34	8
40	100	100	96	81	47	97	90	81	37	63	99	73	42	99	65	87	95	13	7
75	100	100	63	78	72	100	98	58	36	45	100	100	42	93	58	90	79	33	7
11	100	100	100	44	35	100	100	100	34	3	69	97	59	63	72	66	85	13	7
13	100	97	70	91	80	89	93	59	33	44	100	98	26	85	71	84	70	46	6
30	100	99	76	99	94	75	97	56	30	93	100	99	29	70	43	68	80	17	5
6	100	95	100	75	89	42	50	82	30	57	100	99	40	76	68	73	86	26	5
61	100	71	100	100	99	98	99	39	29	34	84	80	100	100	72	94	96	12	5
37	99	99	74	82	62	90	100	51	29	32	100	99	33	96	58	73	90	10	5
74	100	100	70	78	64	97	99	46	27	23	99	75	38	83	62	75	94	12	5
10	100	100	63	96	67	98	100	43	27	15	99	97	25	83	54	81	88	20	5
78	100	100	82	95	66	99	100	46	26	96	100	85	60	45	52	42	38	37	4
9	100	99	82	64	47	89	95	55	25	58	98	83	97	80	71	56	76	14	4
64	100	100	75	89	62	70	88	39	24	52	100	94	98	32	28	69	67	26	4
43	100	100	50	99	78	98	62	65	24	51	100	99	74	58	37	97	94	23	4
69	100	100	55	81	63	100	97	45	23	33	99	80	26	72	73	33	54	18	4
55	94	80	99	100	79	98	99	40	23	16	98	91	33	56	54	74	92	24	4
85	76	82	100	99	100	86	92	30	21	88	100	95	24	91	56	94	65	57	3
67	100	83	88	92	59	99	100	46	21	70	100	98	34	79	61	78	87	7	3
91	93	84	100	100	100	92	95	27	20	66	99	97	15	86	63	66	80	32	3
39	100	98	50	93	86	52	93	61	20	100	100	99	57	67	84	52	22	13	2
21	100	99	46	85	66	92	99	34	19	89	100	83	49	76	61	72	71	24	2
49	100	97	58	97	59	97	100	36	18	79	78	100	30	45	33	85	86	6	2
98	100	100	100	54	41	91	98	41	17	19	100	100	31	90	70	90	44	38	2
90	100	100	73	46	34	100	100	52	17	53	100	99	78	32	24	85	52	7	1
84	100	100	58	96	75	83	97	52	17	97	100	86	25	37	19	50	13	12	0
82	100	100	51	82	56	100	100	36	17	86	97	32	99	72	60	59	76	4	0
17	100	89	96	100	78	99	99	27	17	56	100	59	16	71	60	46	67	14	0
62	100	100	41	92	69	69	97	50	16	41	100	78	55	59	80	67	48	10	0
77	100	93	54	73	55	79	91	38	14	**avg**	**99**	**93**	**70**	**80**	**66**	**81**	**86**	**47**	**22**

Table 2: Parameters sensitivity intervals. Table shows the relative and absolute parameter sensitivity intervals. Colors indicate the level of sensitivity where green are the least sensitive (such as the diffusion coefficient) yellow intermediates and red the most sensitive's.

parameter	mean parameter value	average lower value	average upper value	relative lower (%)	relative upper (%)
D_{Kr}	0.246	0.173	0.196	70.39	79.52
D_{cad}	0.242	0.242	2.174	100	898.07
D_{gt}	0.201	0.192	0.416	95.23	206.54
D_{hb}	0.238	0.23	0.548	96.8	230.29
D_{kni}	0.292	0.258	0.436	88.43	149.35
D_{tll}	0.278	0.278	0.698	100	250.82
H_{Kr}	-2.97	0.014	0.013	0.49	0.44
H_{cad}	5.961	0.112	0.098	1.87	1.64
H_{gt}	-2.97	0.015	0.016	0.51	0.52
H_{hb}	-2.97	0.041	0.036	1.38	1.21
H_{kni}	-2.97	0.033	0.033	1.13	1.13
H_{tll}	-1.613	0.028	0.03	1.76	1.89
L_{Kr}	7.649	0.275	0.284	3.6	3.72
L_{cad}	14.912	1.121	1.012	7.52	6.78
L_{gt}	5.768	0.2	0.212	3.47	3.67
L_{hb}	7.472	0.348	0.374	4.66	5
L_{kni}	7.252	0.457	0.522	6.31	7.2
L_{tll}	6.745	0.341	0.359	5.05	5.32
R_{Kr}	20.756	0.297	0.281	1.43	1.36
R_{cad}	24.235	2.13	2.154	8.79	8.89
R_{gt}	27.199	0.419	0.429	1.54	1.58
R_{hb}	21.926	0.731	0.696	3.33	3.18
R_{kni}	23.302	0.766	0.762	3.29	3.27
R_{tll}	24.464	0.58	0.607	2.37	2.48
bcd_{Kr}	4.49E-02	8.05E-04	5.57E-04	1.79	1.24
bcd_{cad}	-2.37E-02	1.39E-02	1.42E-02	58.53	59.76
bcd_{gt}	5.61E-02	7.09E-04	1.13E-03	1.26	2.02
bcd_{hb}	2.41E-02	2.68E-03	3.23E-03	11.12	13.38
bcd_{kni}	2.70E-02	4.48E-03	4.41E-03	16.59	16.34
bcd_{tll}	-8.01E-02	7.42E-03	7.08E-03	9.26	8.83
Kr→Kr	2.00E-02	9.30E-04	8.32E-04	4.64	4.16
Kr→cad	-2.51E-02	6.71E-03	5.17E-03	26.68	20.55
Kr→gt	-7.54E-02	1.49E-03	1.63E-03	1.98	2.17
Kr→hb	4.23E-05	1.61E-03	1.46E-03	3793.97	3440
Kr→kni	-4.51E-03	1.28E-03	1.25E-03	28.32	27.82
Kr→tll	-5.14E-02	4.83E-03	4.66E-03	9.39	9.07
cad→Kr	2.38E-02	1.27E-04	1.25E-04	0.53	0.53
cad→cad	-2.81E-02	9.13E-04	8.00E-04	3.25	2.85
cad→gt	2.12E-02	1.41E-04	1.34E-04	0.66	0.63
cad→hb	1.46E-02	4.25E-04	3.51E-04	2.91	2.4
cad→kni	2.52E-02	2.52E-04	2.55E-04	1	1.01
cad→tll	1.79E-02	2.17E-04	2.36E-04	1.21	1.32
gt→Kr	-4.19E-02	1.05E-03	9.60E-04	2.51	2.29
gt→cad	-3.25E-02	3.71E-03	2.84E-03	11.42	8.75
gt→gt	1.47E-02	6.33E-04	5.80E-04	4.32	3.96
gt→hb	6.95E-03	1.91E-03	1.62E-03	27.49	23.27
gt→kni	-1.85E-02	2.12E-03	1.98E-03	11.42	10.65
gt→tll	-1.80E-02	1.41E-03	1.54E-03	7.81	8.55
hb→Kr	-4.23E-03	4.22E-04	2.88E-04	9.99	6.8
hb→cad	-3.52E-02	4.83E-03	3.75E-03	13.73	10.68
hb→gt	-1.21E-03	3.27E-04	5.06E-04	26.95	41.74
hb→hb	1.98E-02	8.63E-04	7.94E-04	4.35	4
hb→kni	-5.61E-02	4.91E-03	4.79E-03	8.77	8.54
hb→tll	-2.15E-02	2.20E-03	2.29E-03	10.19	10.63
kni→Kr	-1.30E-02	1.20E-03	1.14E-03	9.29	8.82
kni→cad	-1.92E-02	4.96E-03	4.50E-03	25.81	23.41
kni→gt	1.51E-03	9.37E-04	9.81E-04	62.21	65.13
kni→hb	-1.08E-01	1.26E-02	9.90E-03	11.72	9.2
kni→kni	1.83E-02	1.29E-03	1.33E-03	7.08	7.3
kni→tll	-5.02E-02	3.41E-03	3.63E-03	6.79	7.23
tll→Kr	-9.06E-02	5.62E-03	5.19E-03	6.21	5.74
tll→cad	-1.97E-02	3.59E-03	2.09E-03	18.22	10.6
tll→gt	-2.44E-02	2.32E-03	2.05E-03	9.52	8.39
tll→hb	1.25E-03	1.23E-03	5.94E-04	98.45	47.61
tll→kni	-8.91E-02	6.32E-03	6.18E-03	7.1	6.93
tll→tll	1.67E-02	7.74E-04	7.85E-04	4.63	4.7

Figure 1: Stochastic domain score vs. parameters. Correlation between the score of the eight different expression domains scores and all parameters obtained for solution with respectively $H = -2.5$ on the left panel A, $H = -3.5$ on the right panel B. Light green expresses a strong positive correlation and light red a strong negative correlation. Squares in white borders are the most significant correlations.

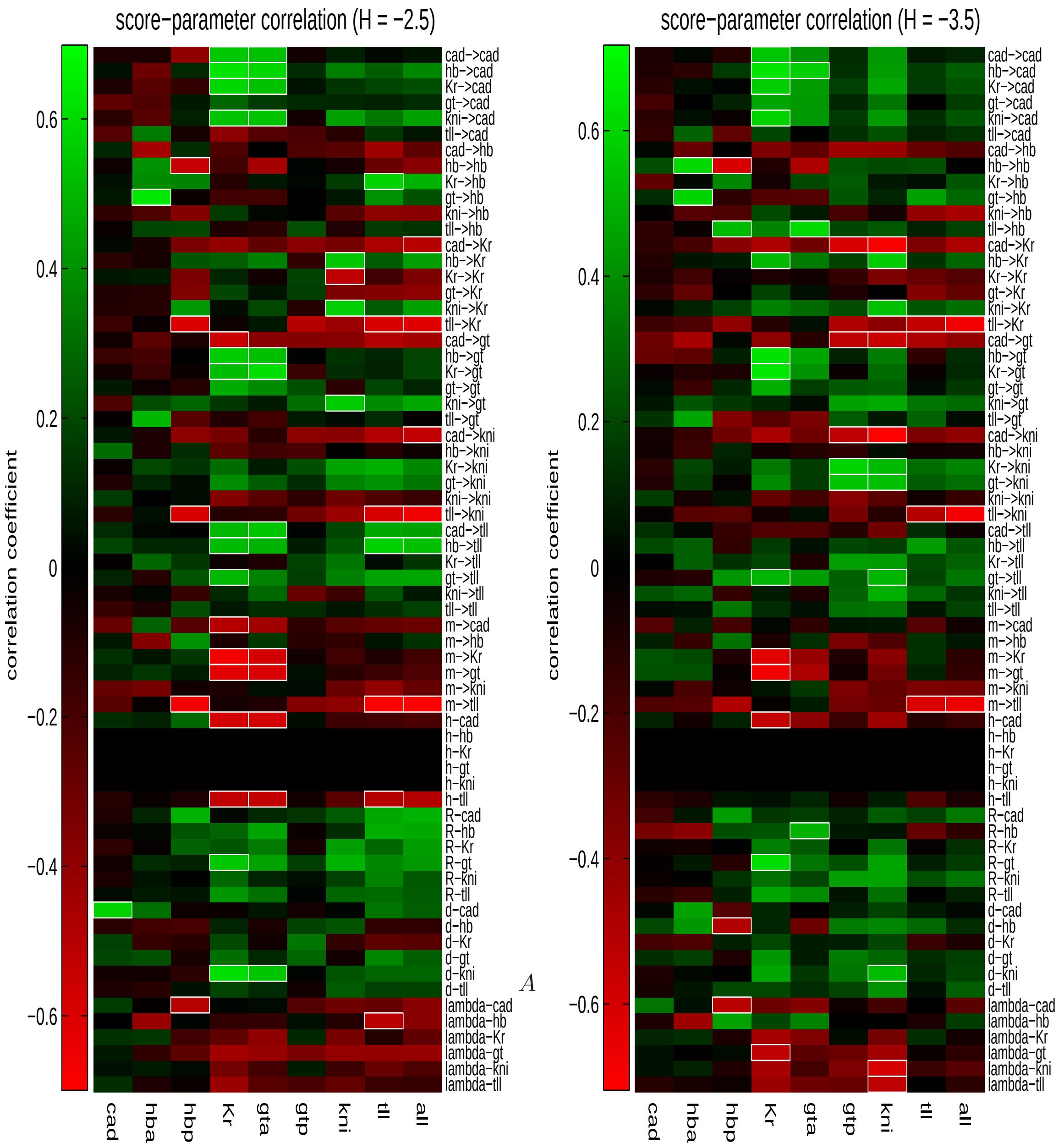

Figure 2: Stochastic domain score vs. parameters sensitivity. Correlation between the score of the eight different expression domains scores and all the parameter sensitivity intervals obtained for solution with respectively $H = -2.5$ on the left panel A, $H = -3.5$ on the right panel B. Light green expresses a strong positive correlation and light red a strong negative correlation. Squares in white borders are the most significant correlations.

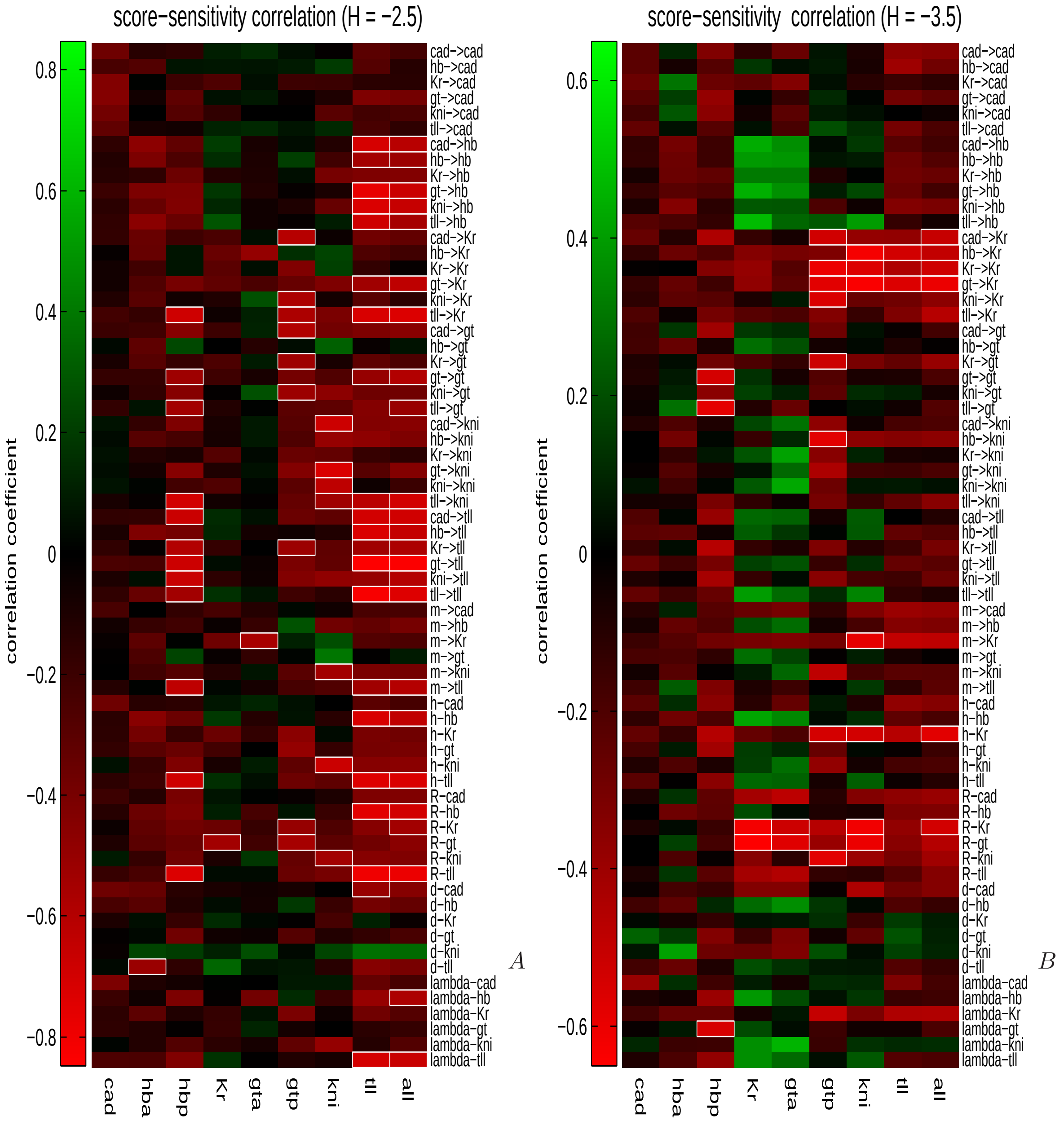

Supplement: Additional file 1 — Additional statistics. This file (GapGeneModelRobustnessAddFile1.pdf) contains the material, which is not given in the paper due to the space limitation. It mainly gives additional statistical results for the stochastic simulations and the perturbation analysis. [file 1752-0509-3-94-S1.PDF]
