# Supplementary figures and images for: Inferring Drosophila gap gene regulatory network: a parameter sensitivity and perturbation analysis
Source: BMC Syst Biol. 2009 Sep 21;3:94. doi: 10.1186/1752-0509-3-94 (PMC2761871; doi:10.1186/1752-0509-3-94)

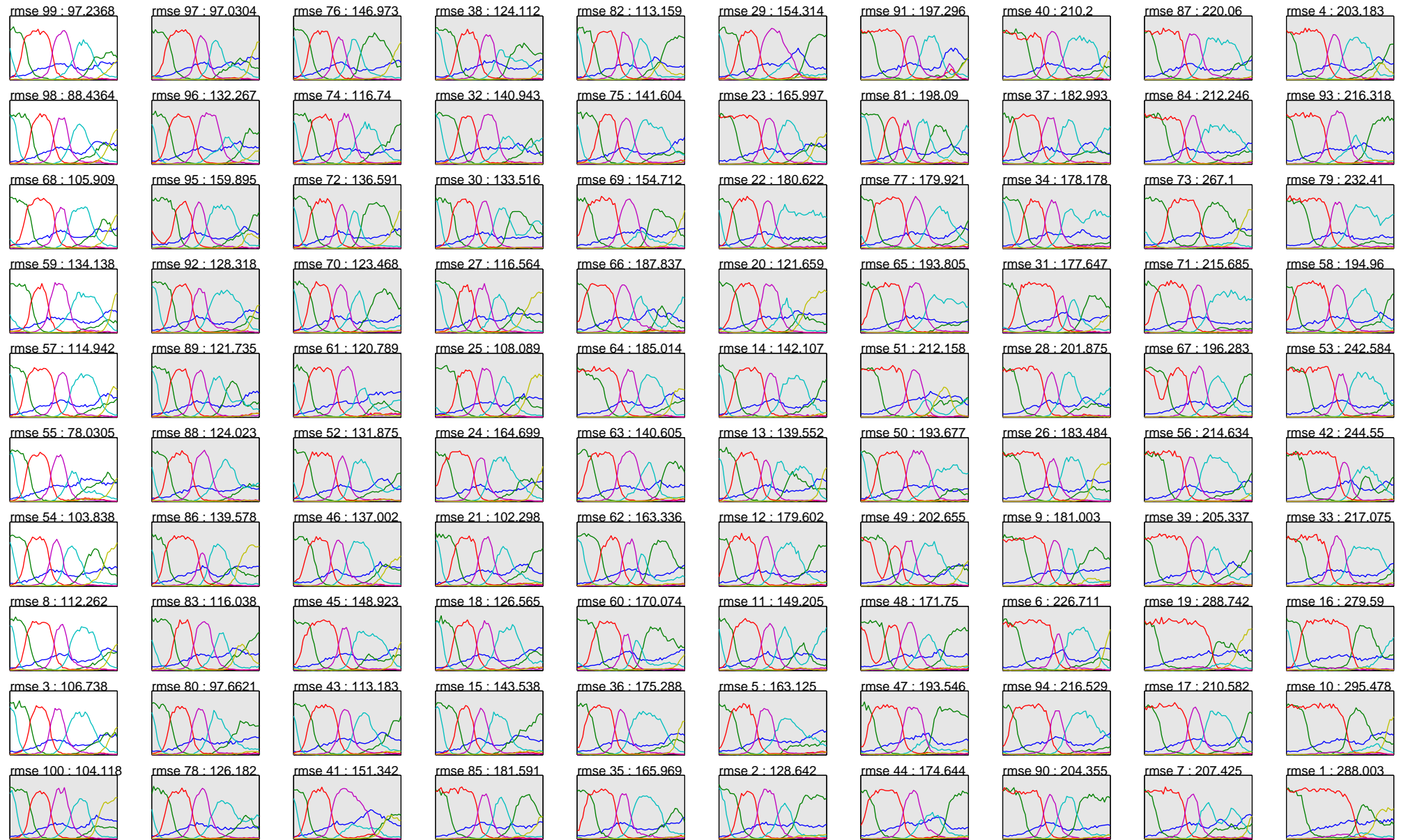

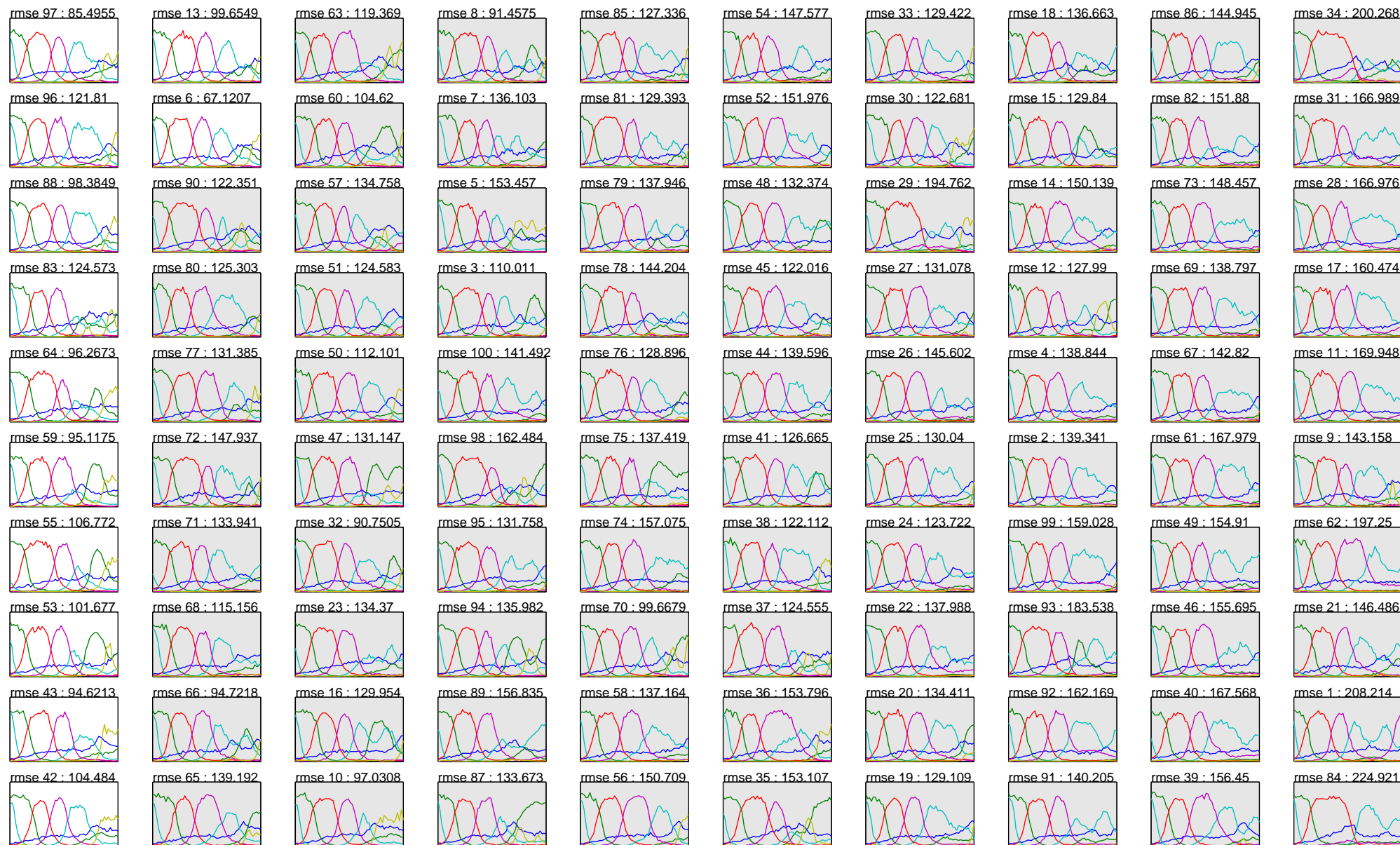

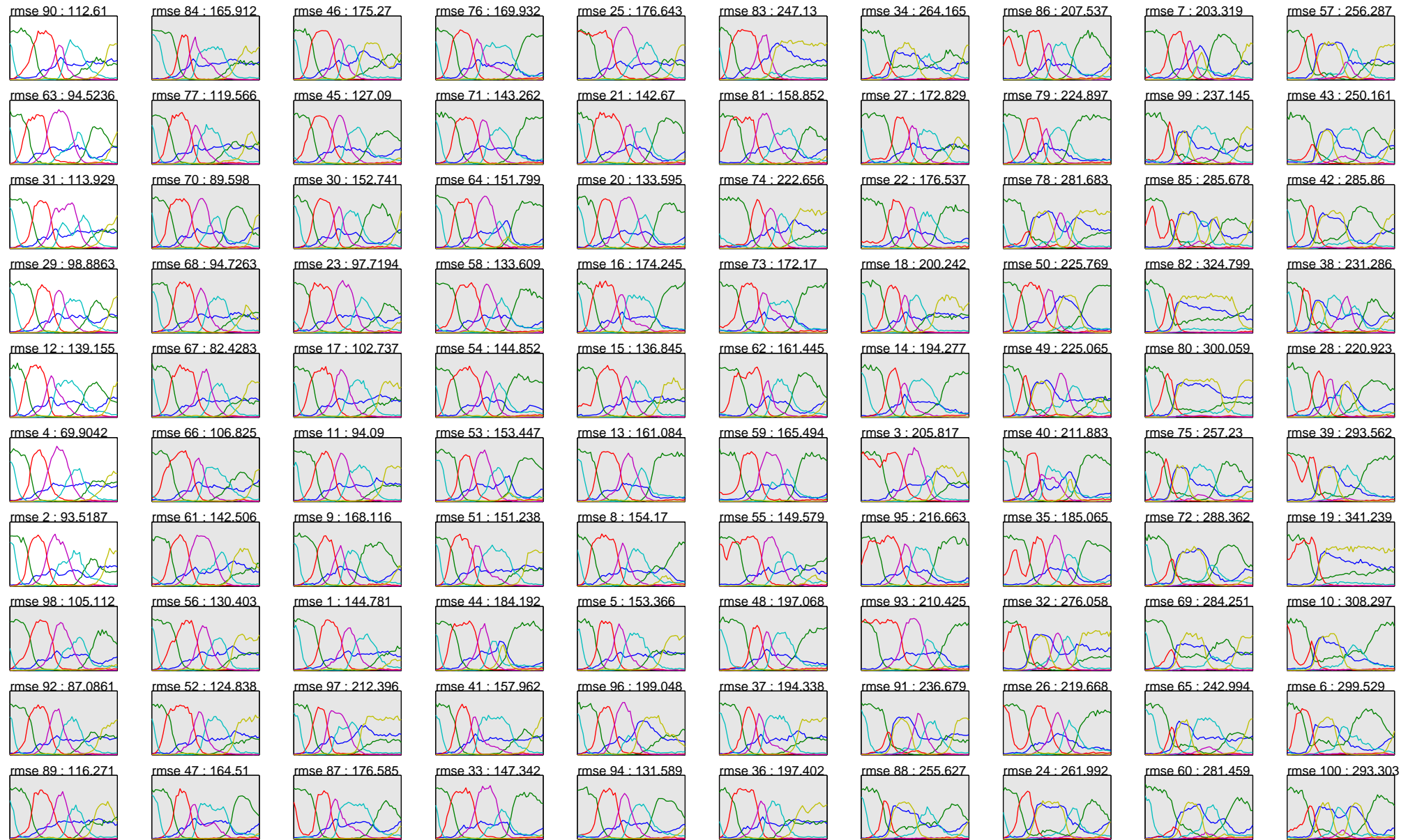

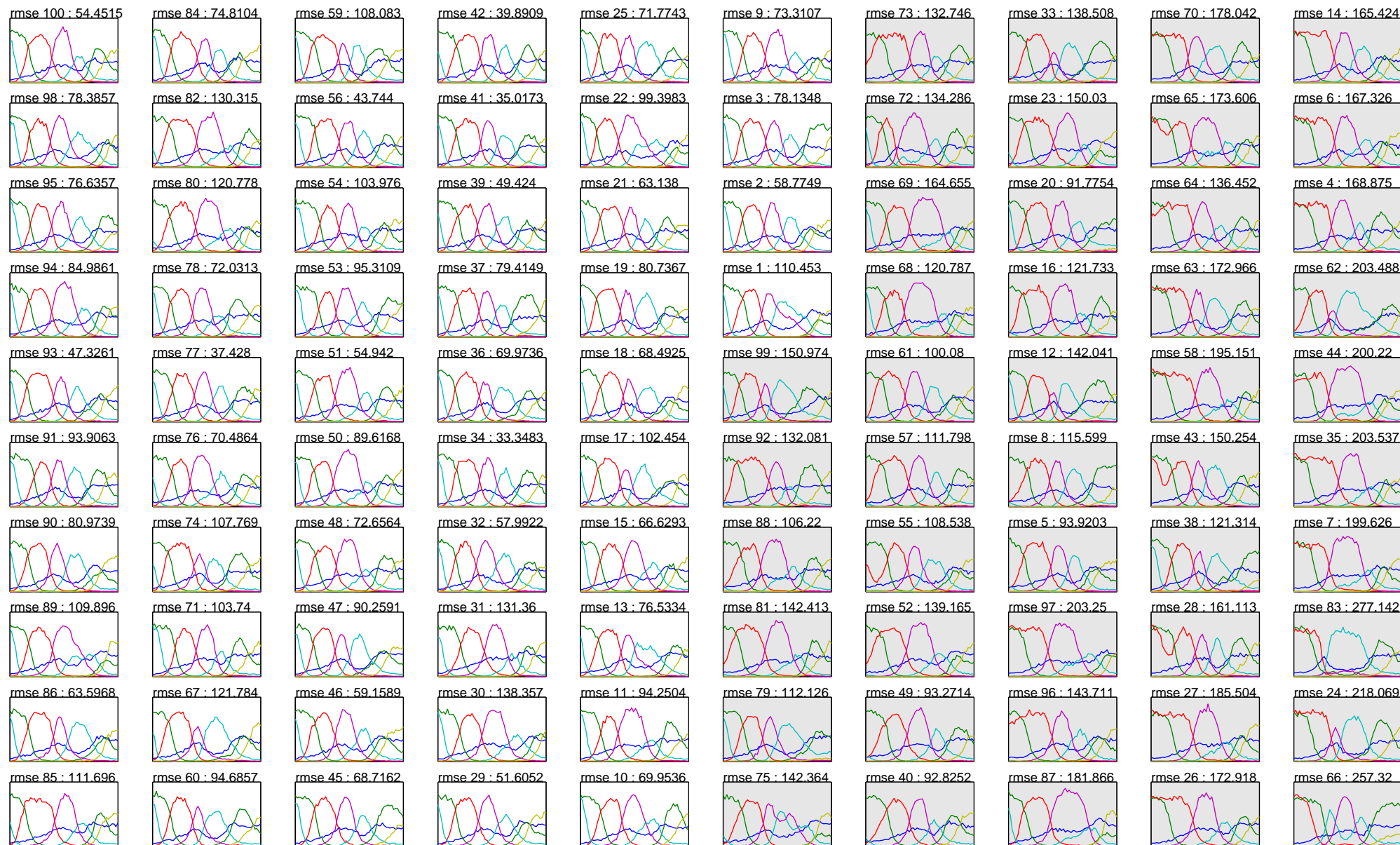

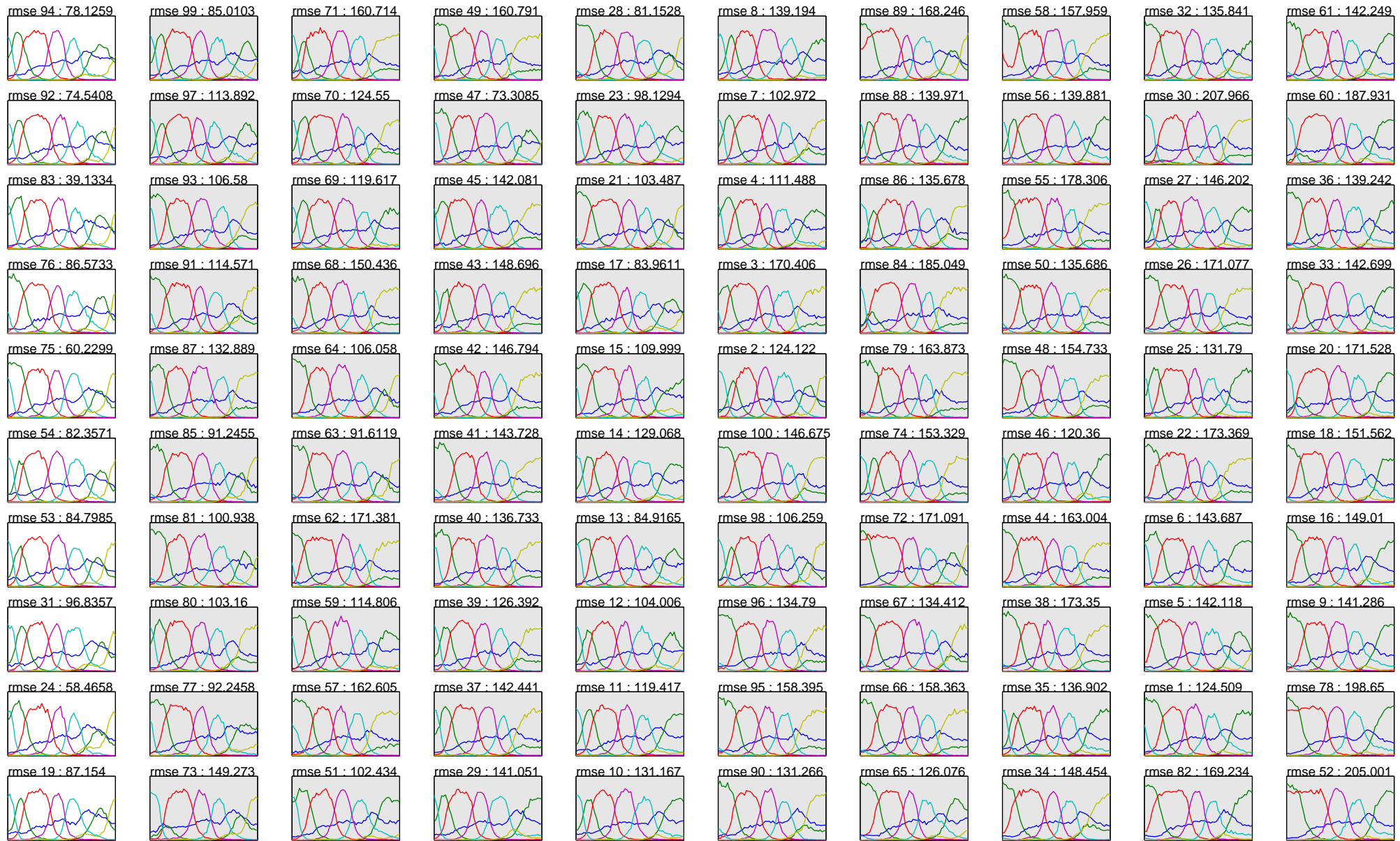

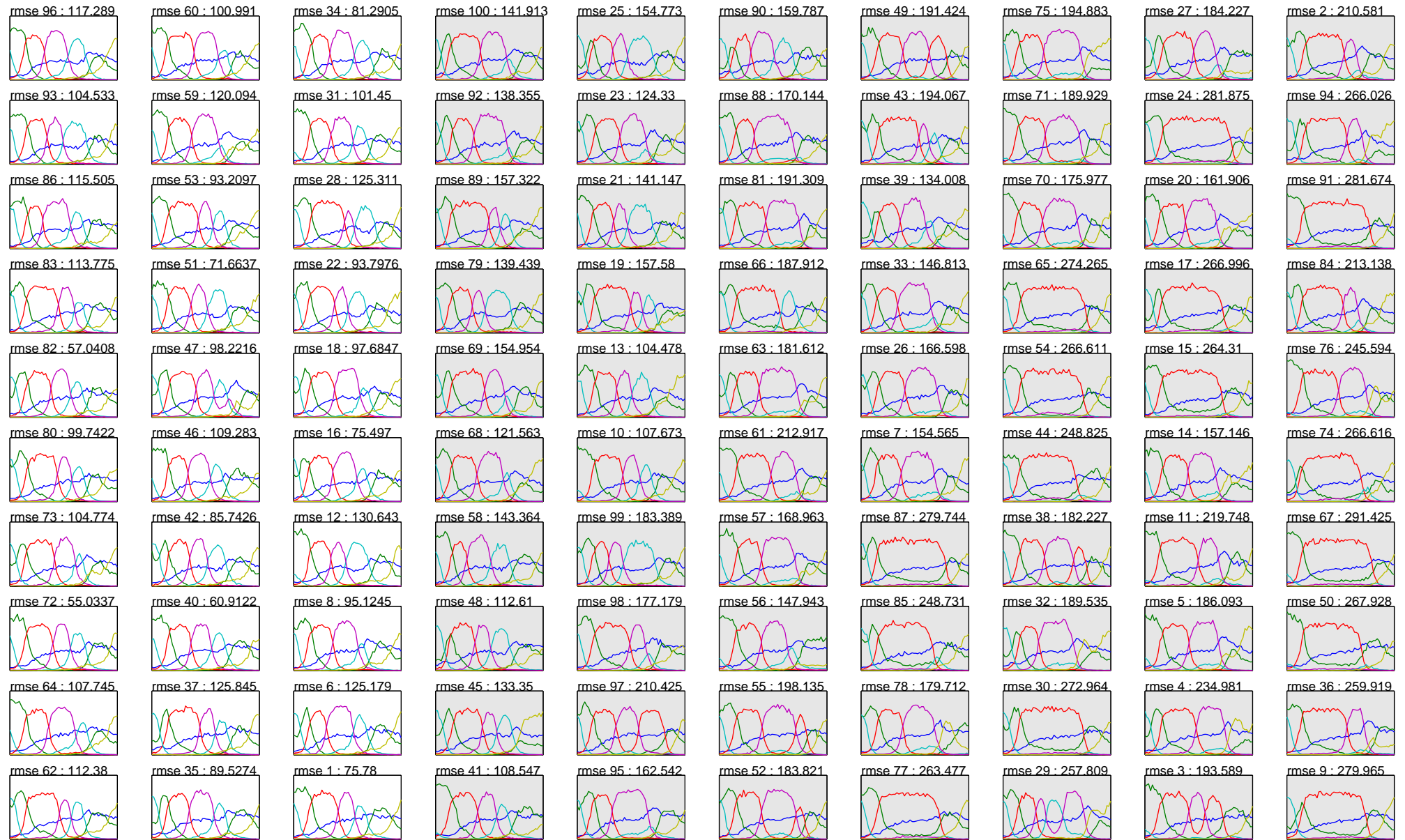

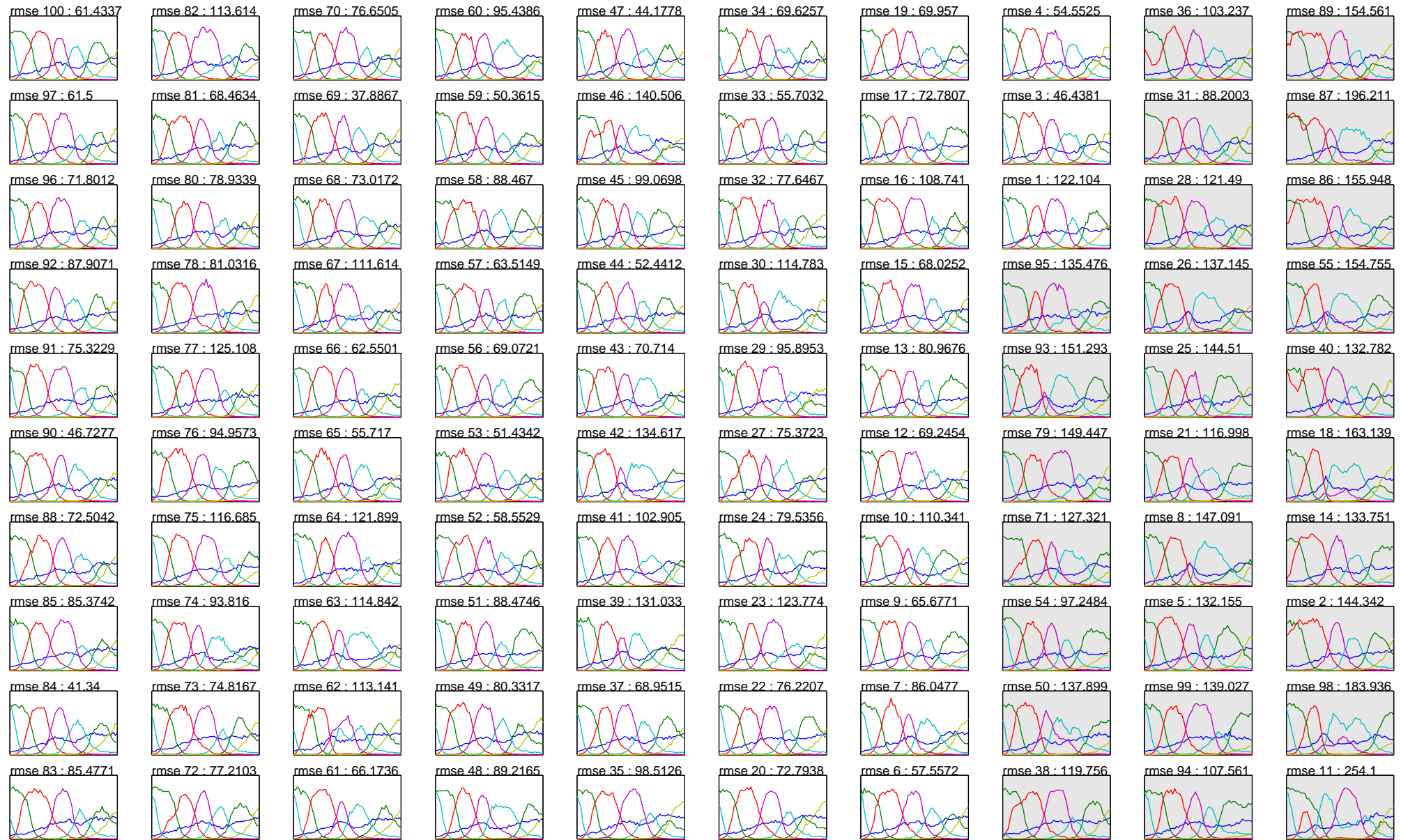

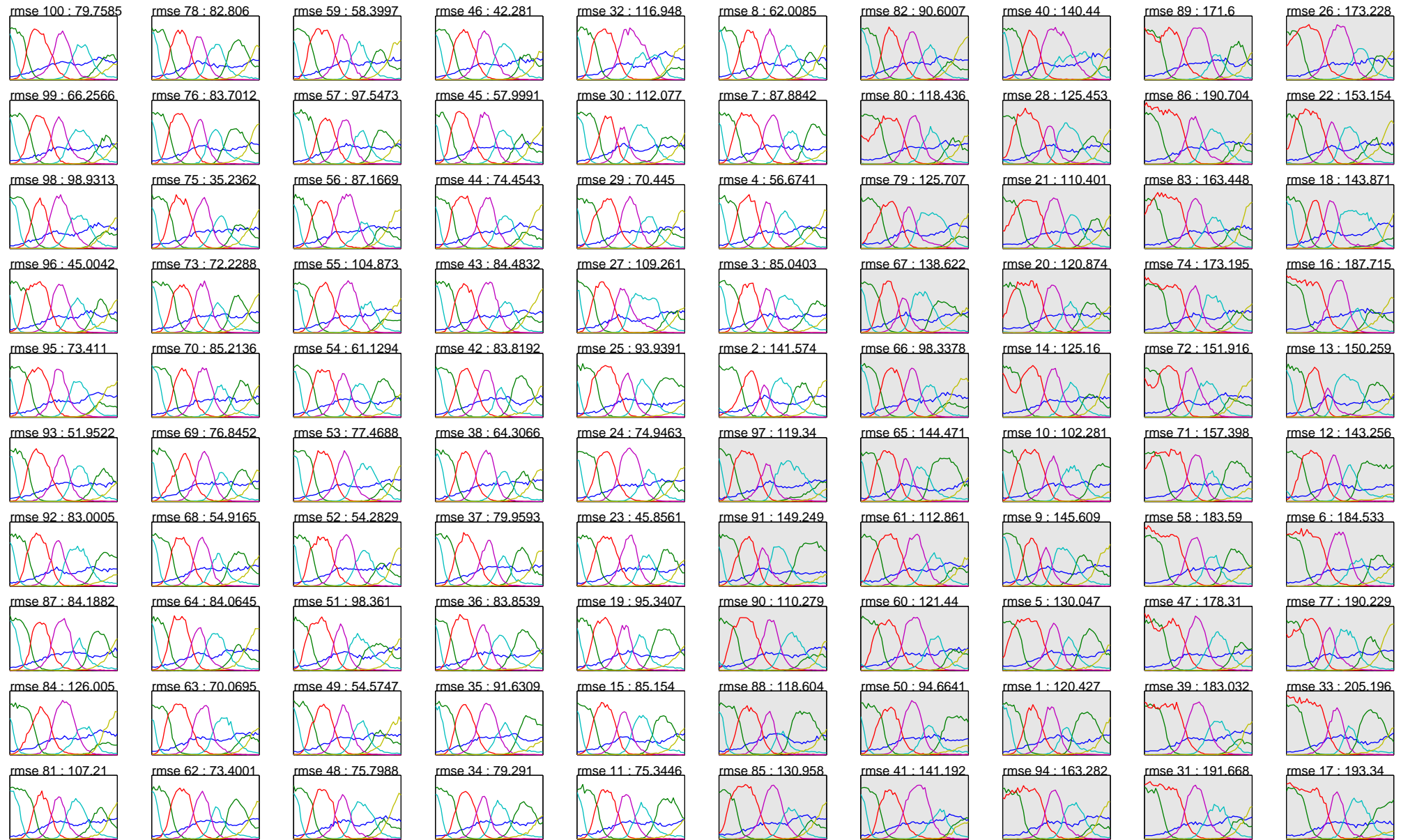

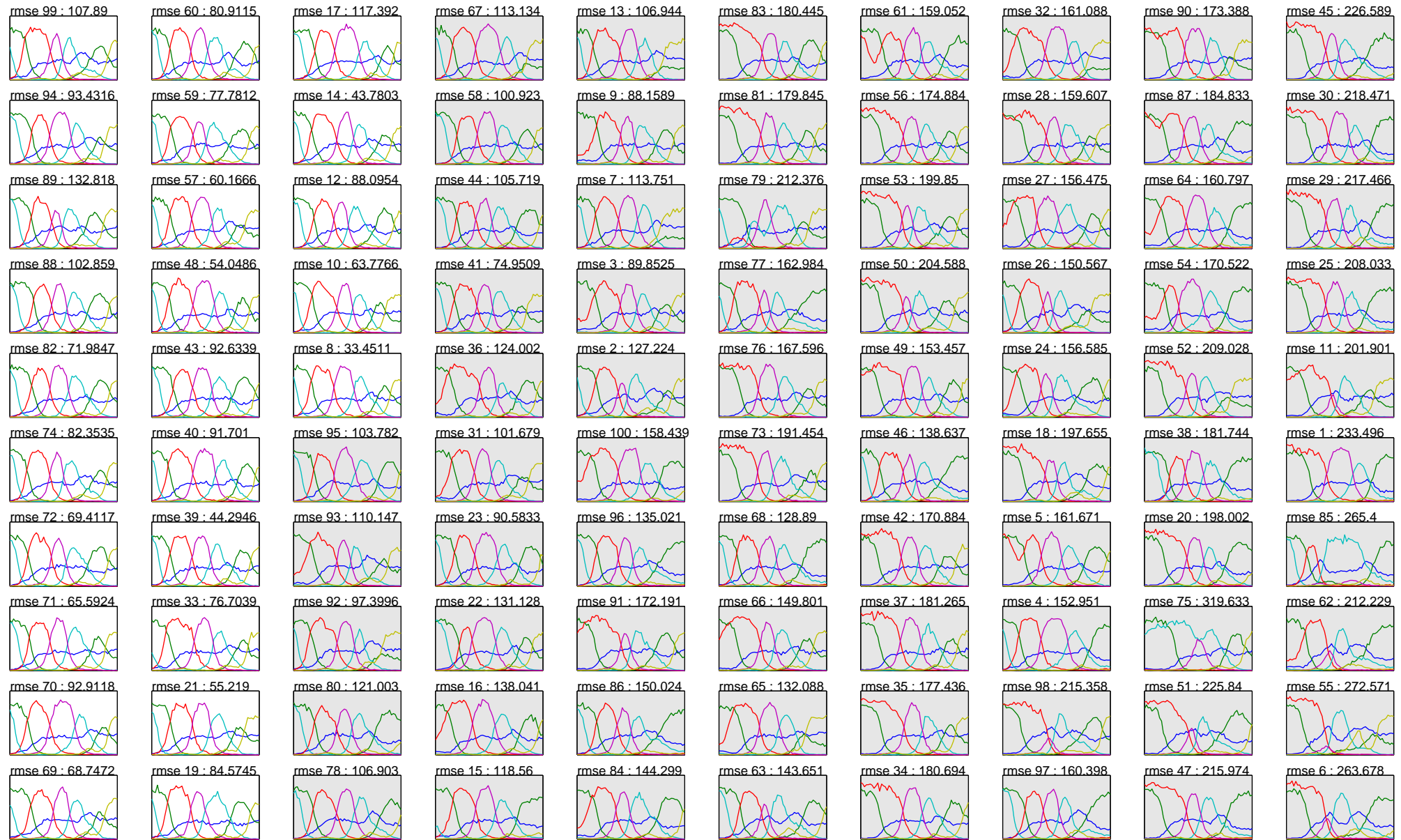

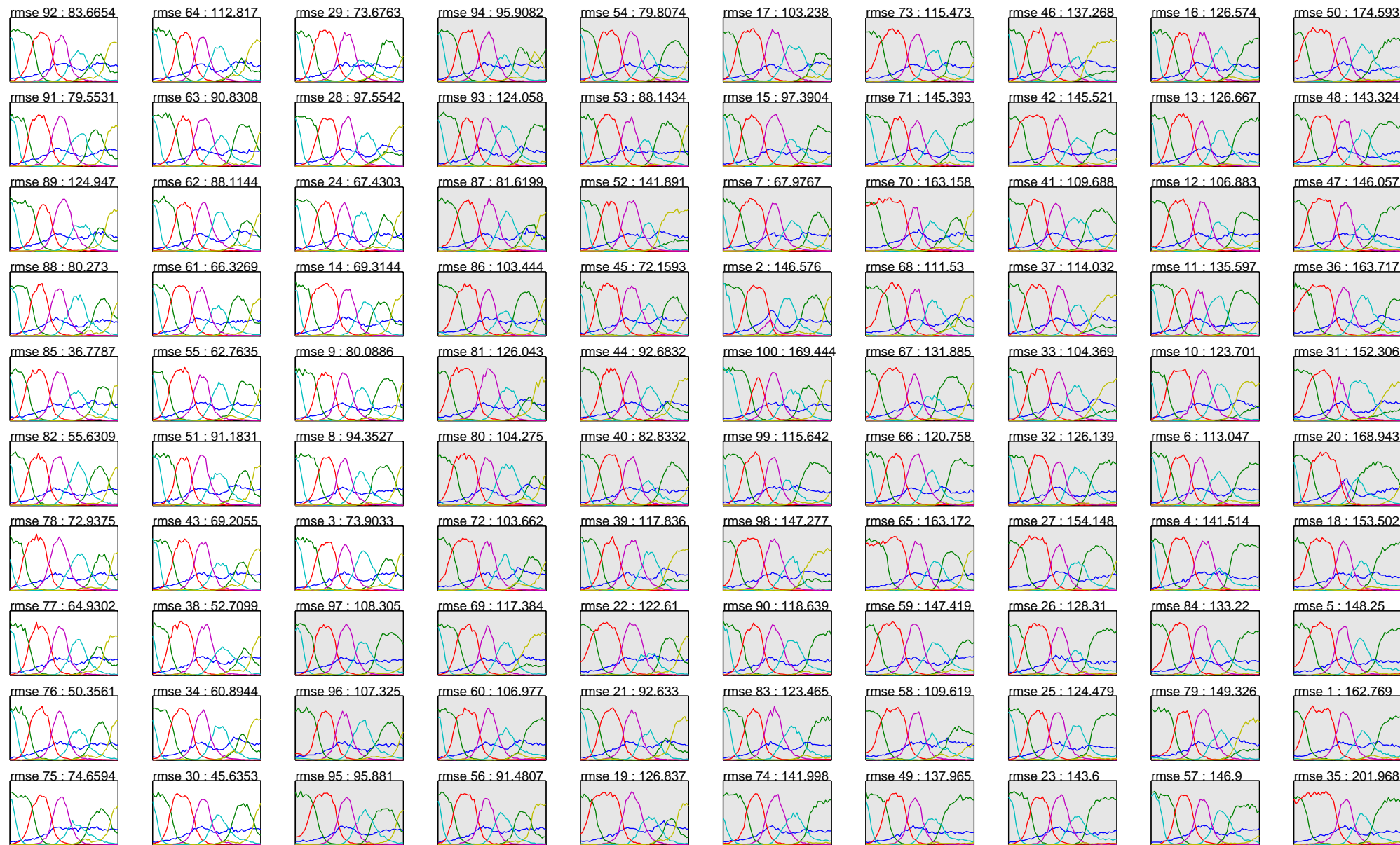

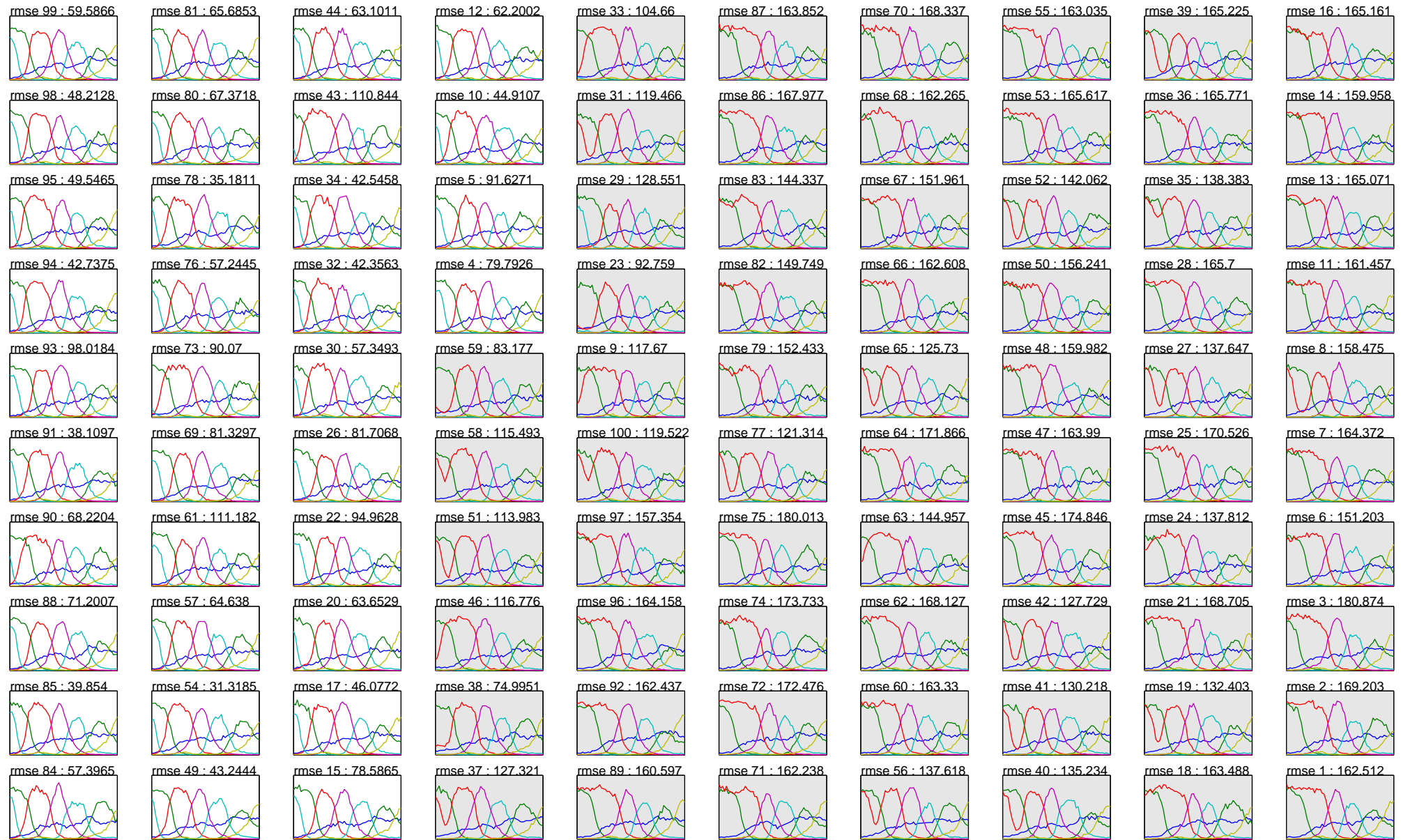

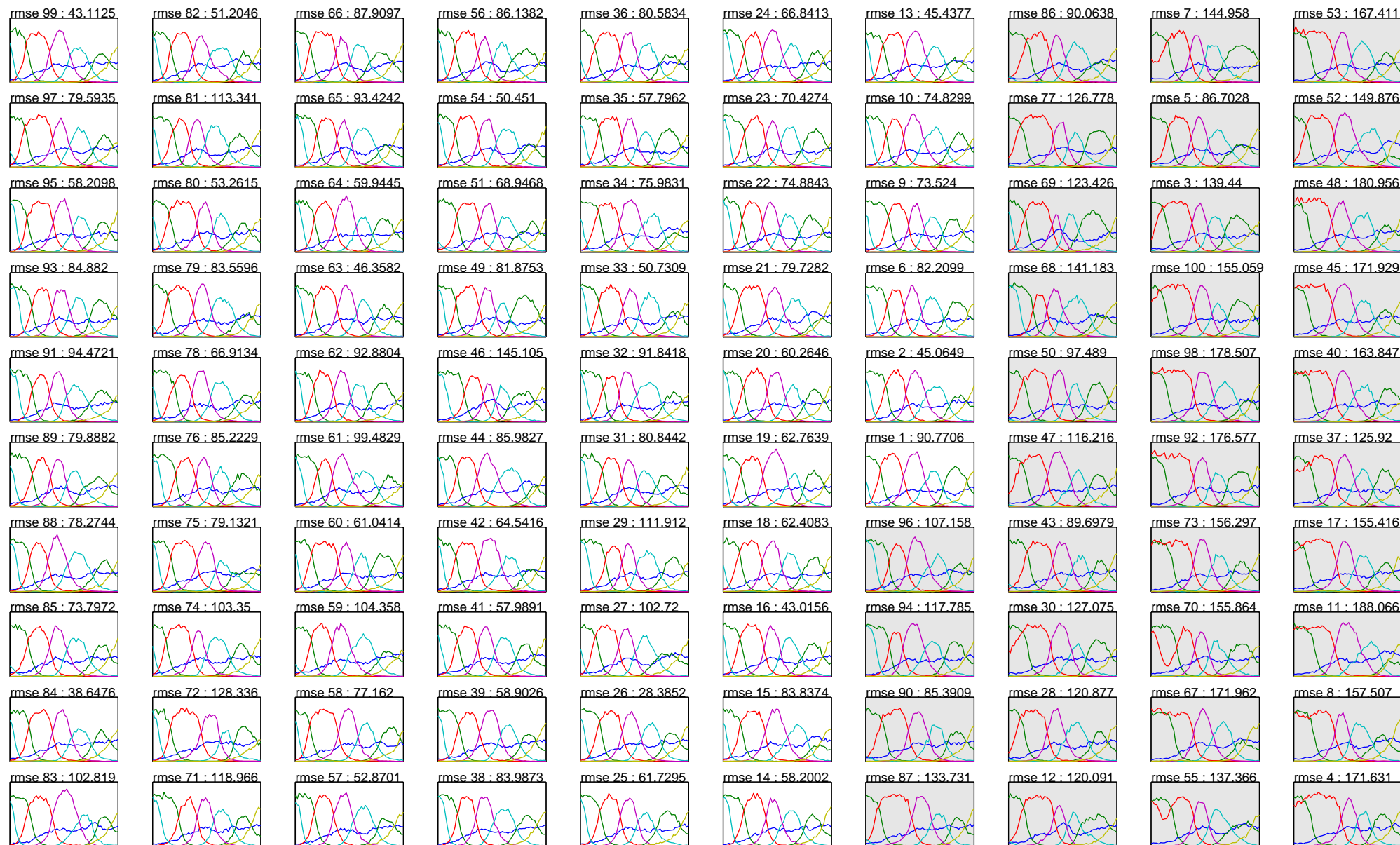

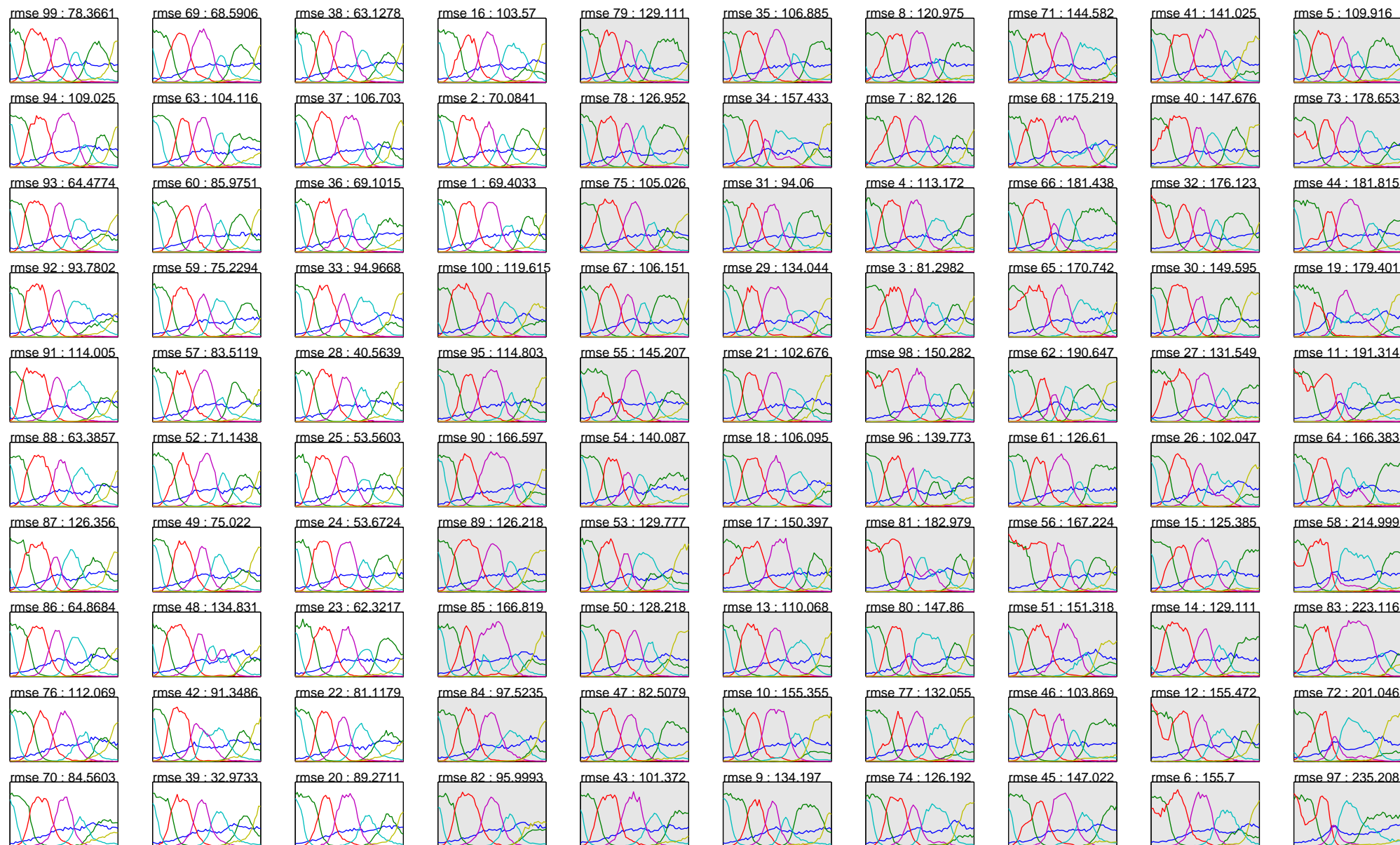

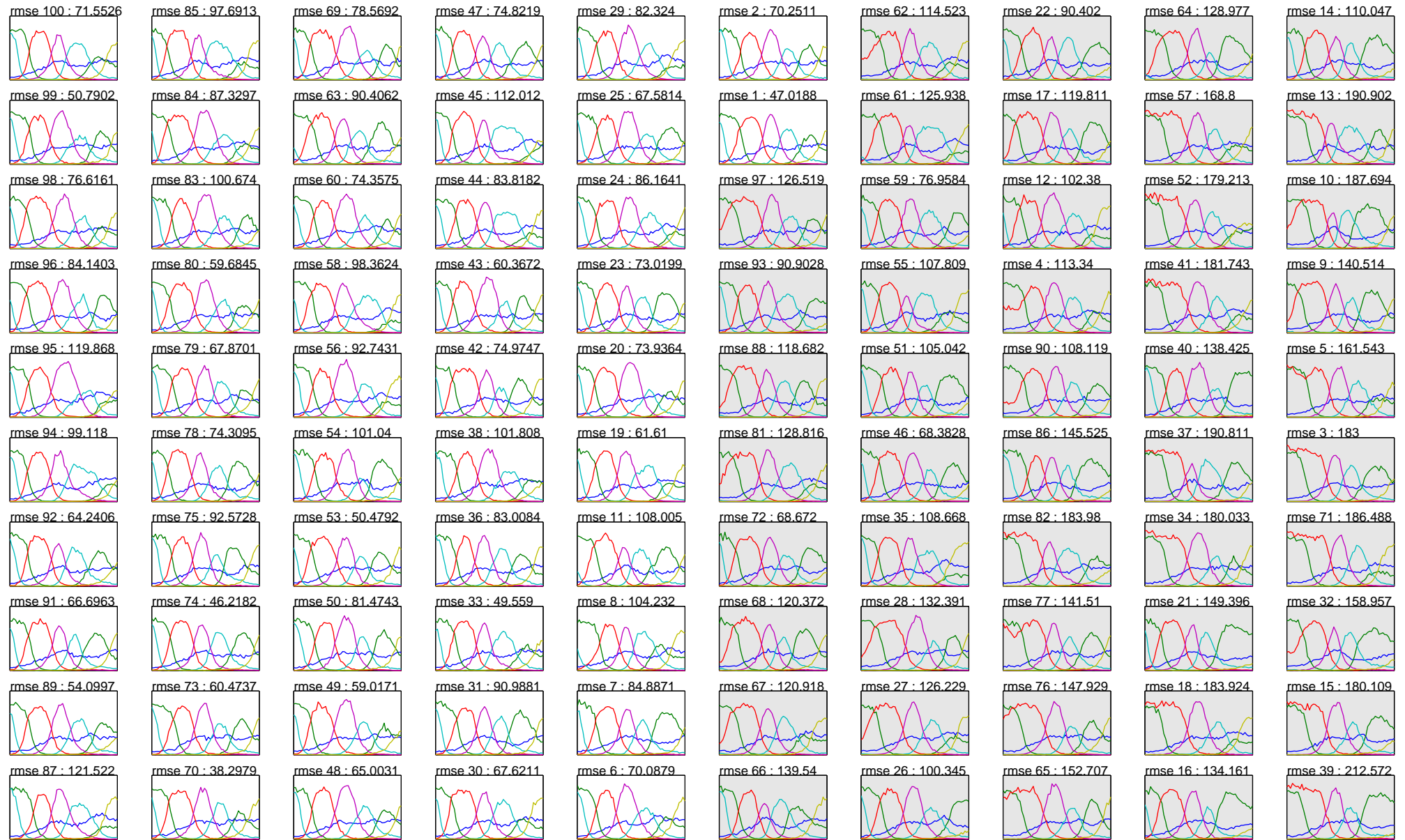

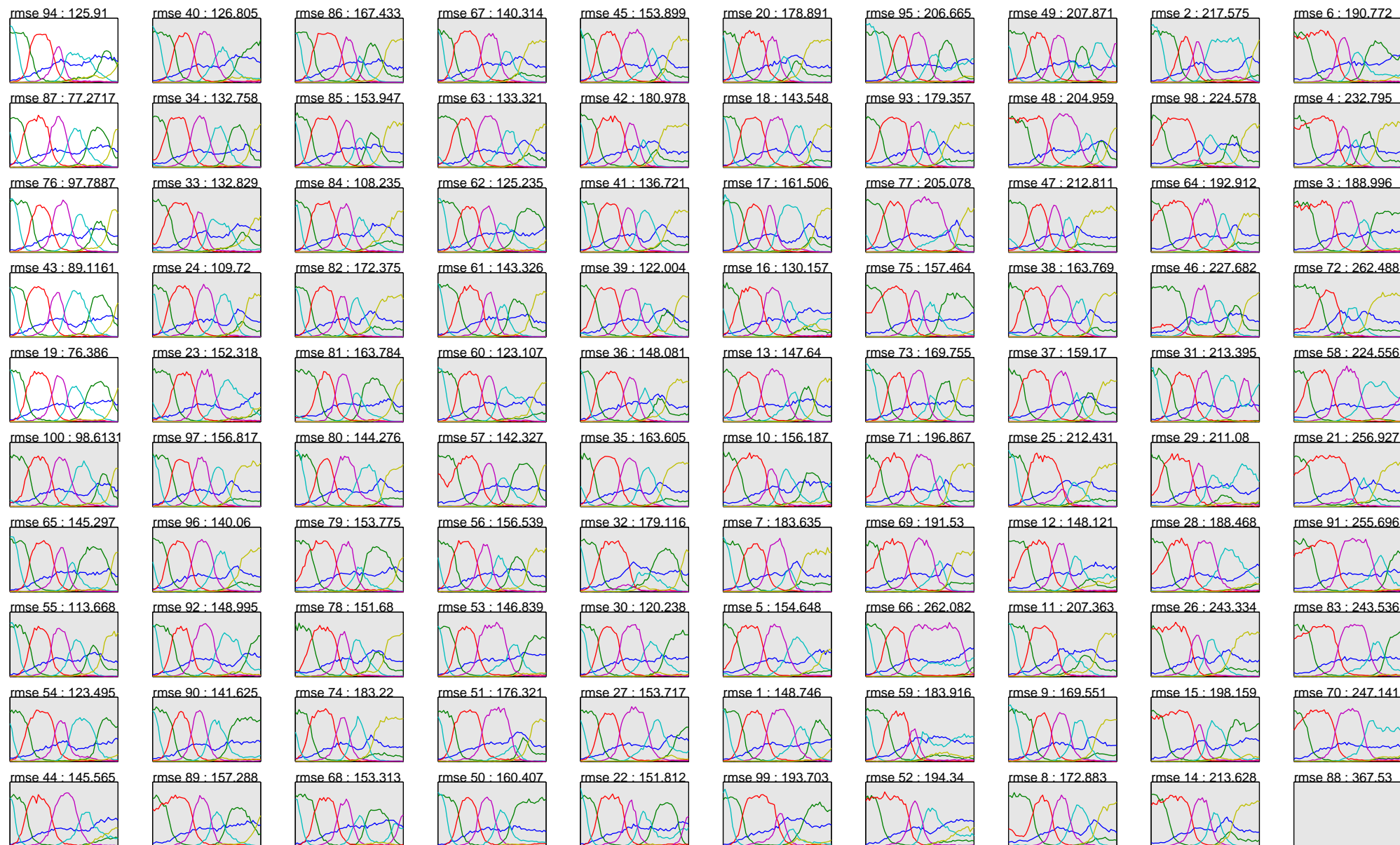

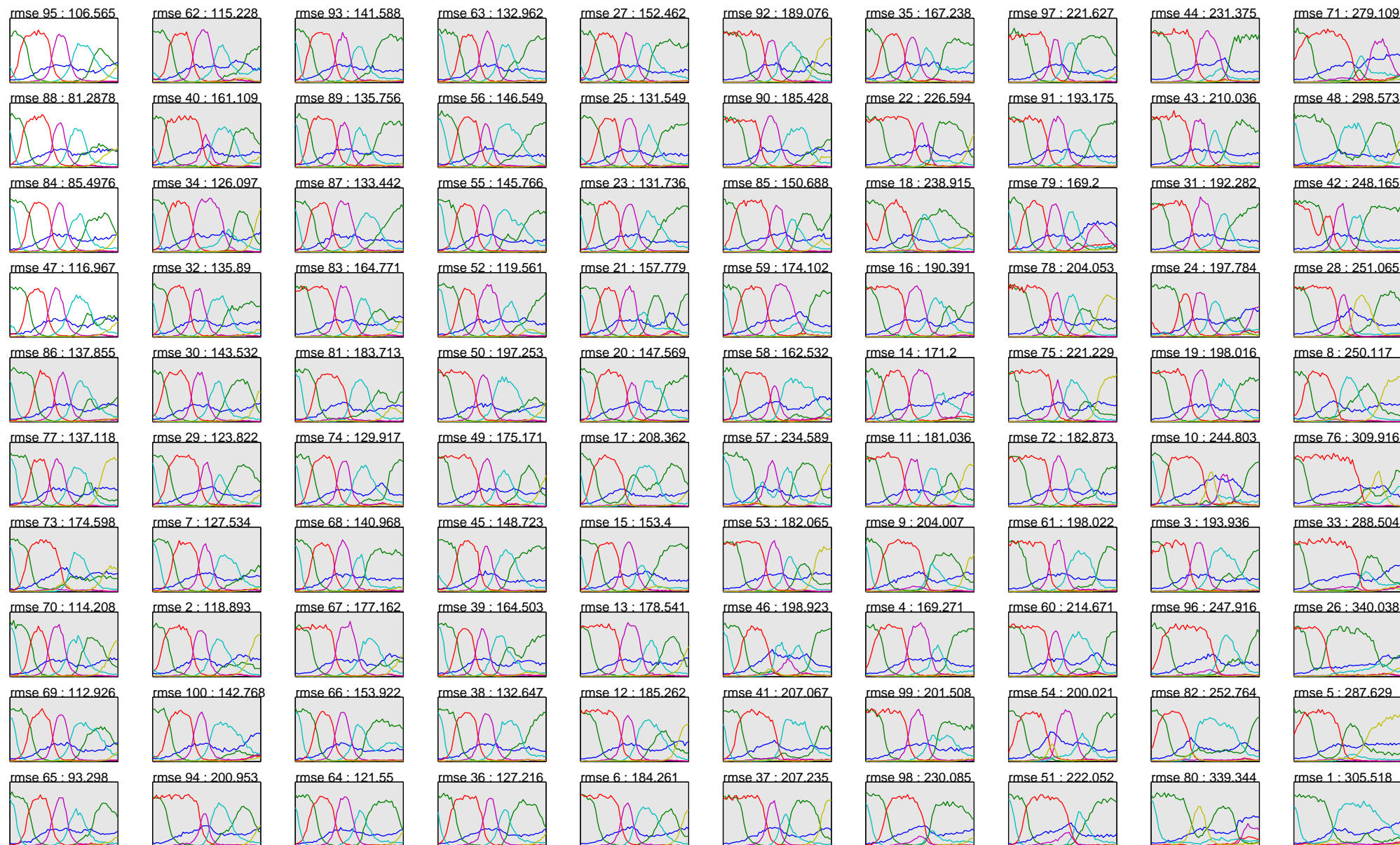

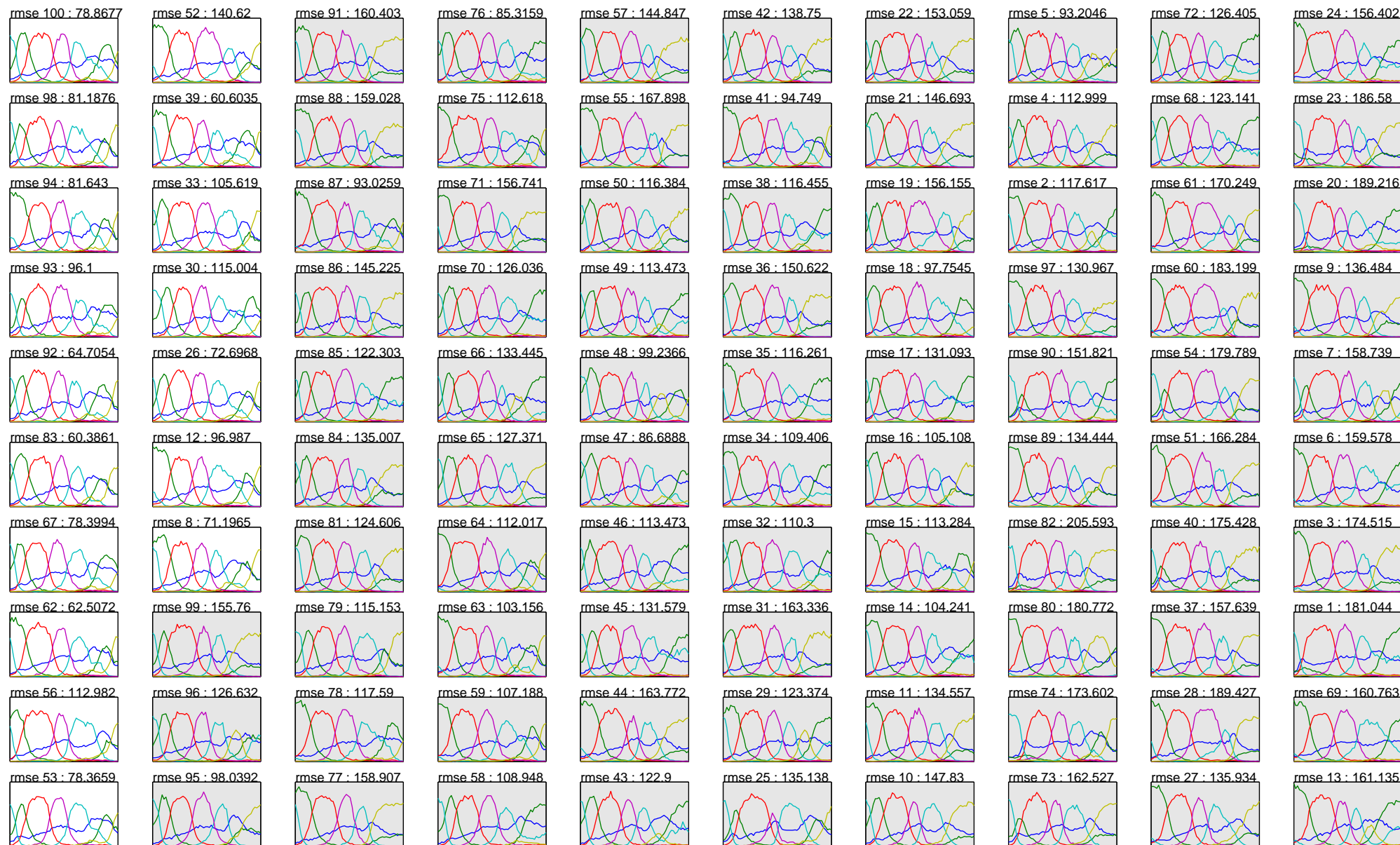

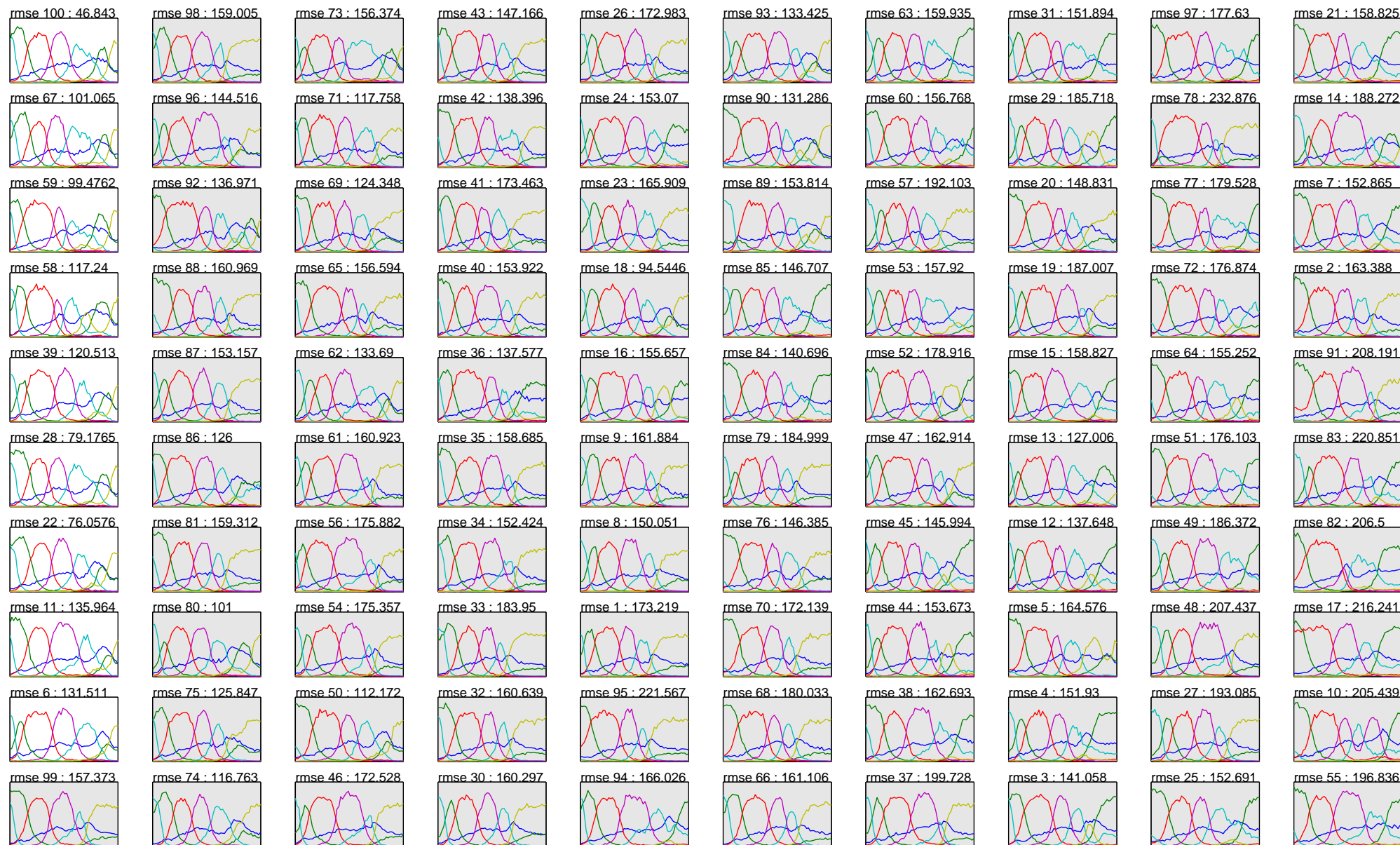

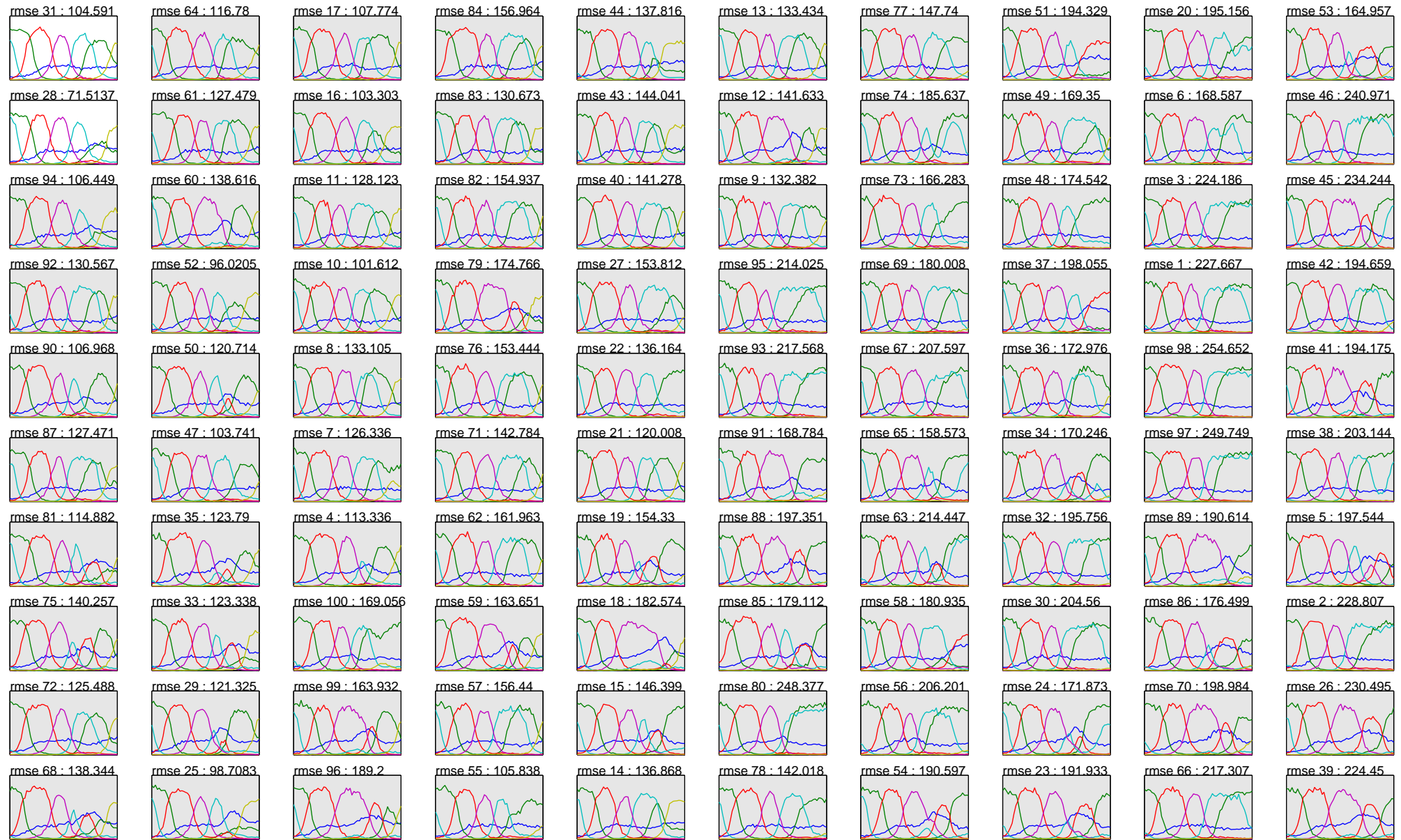

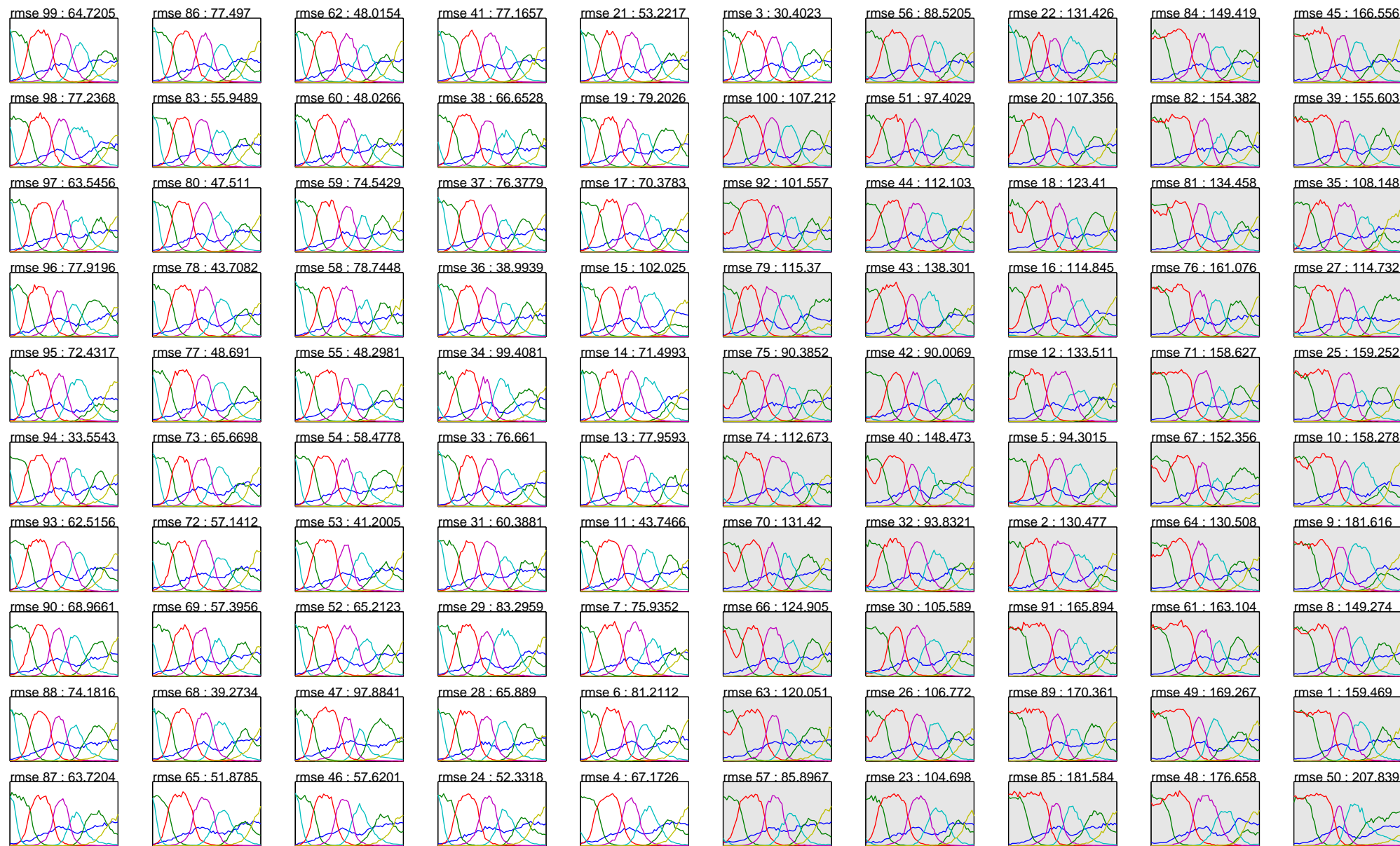

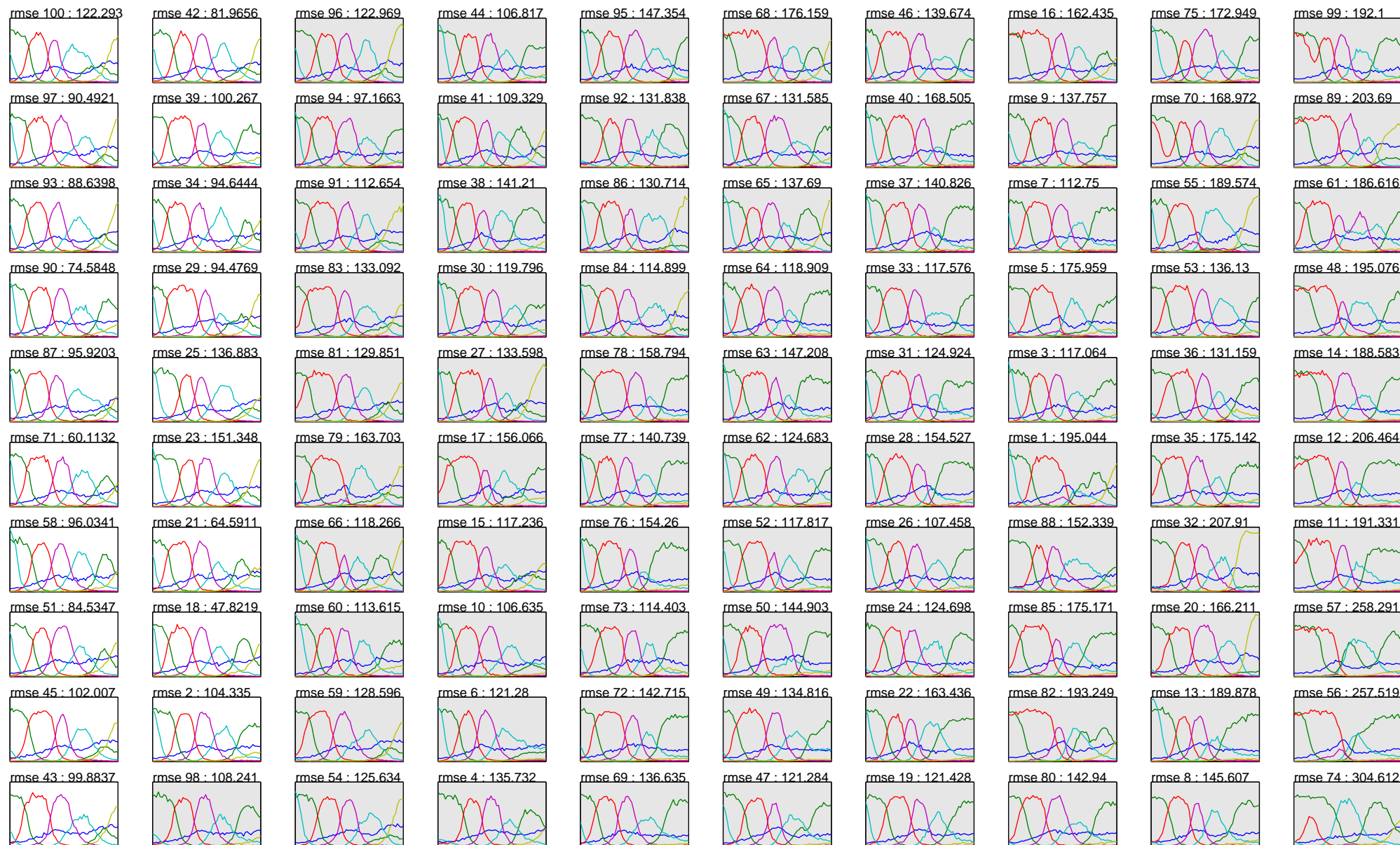

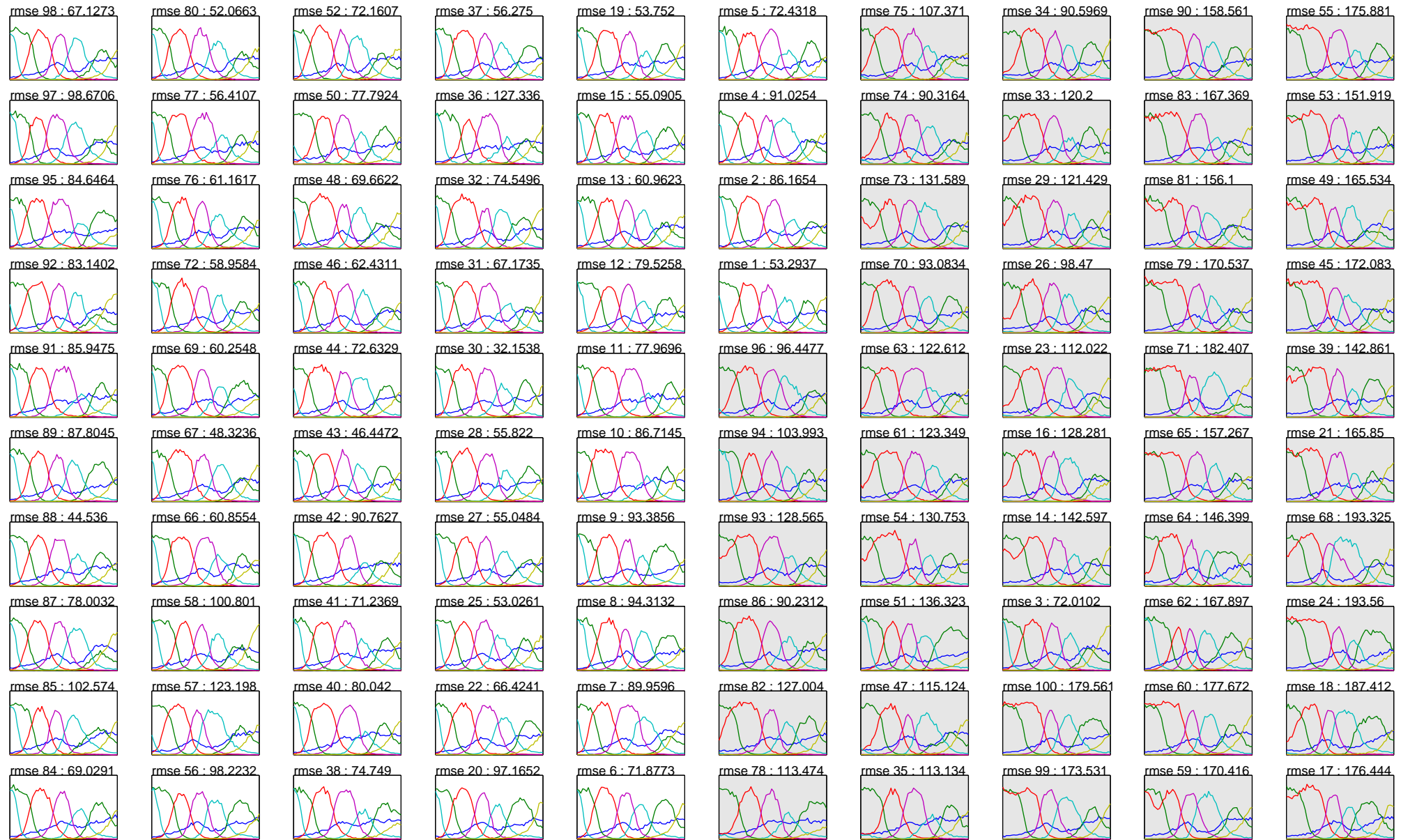

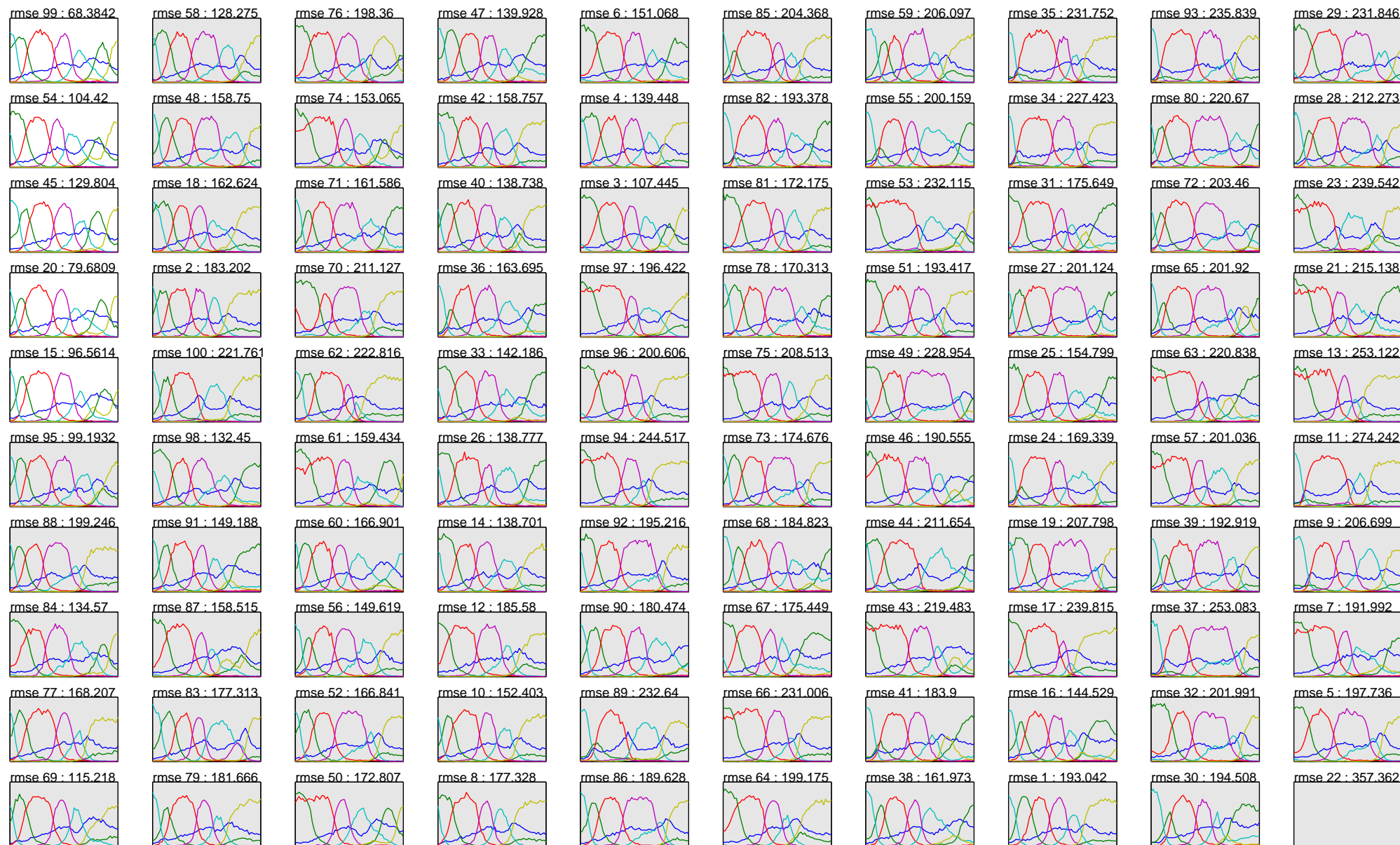

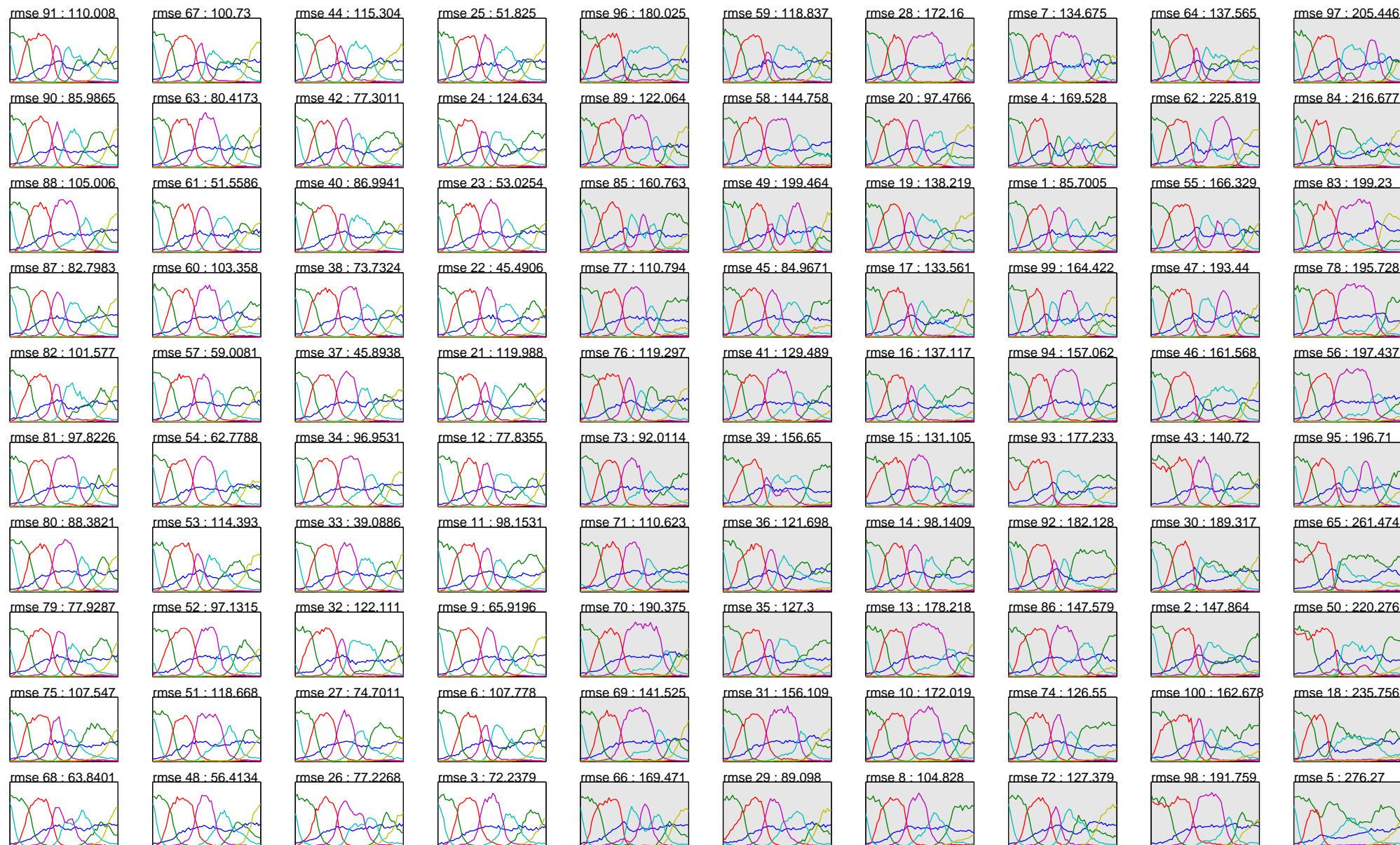

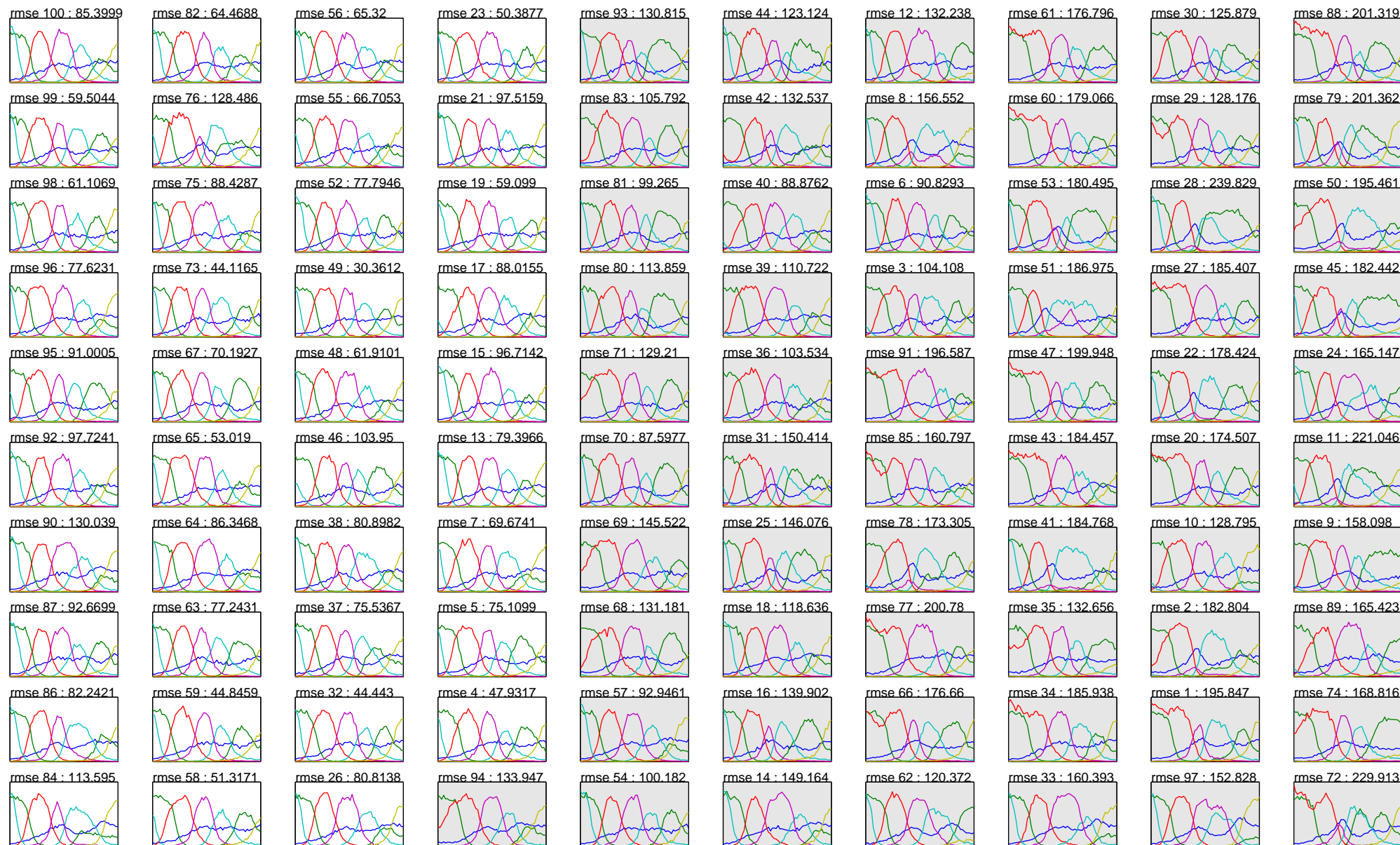

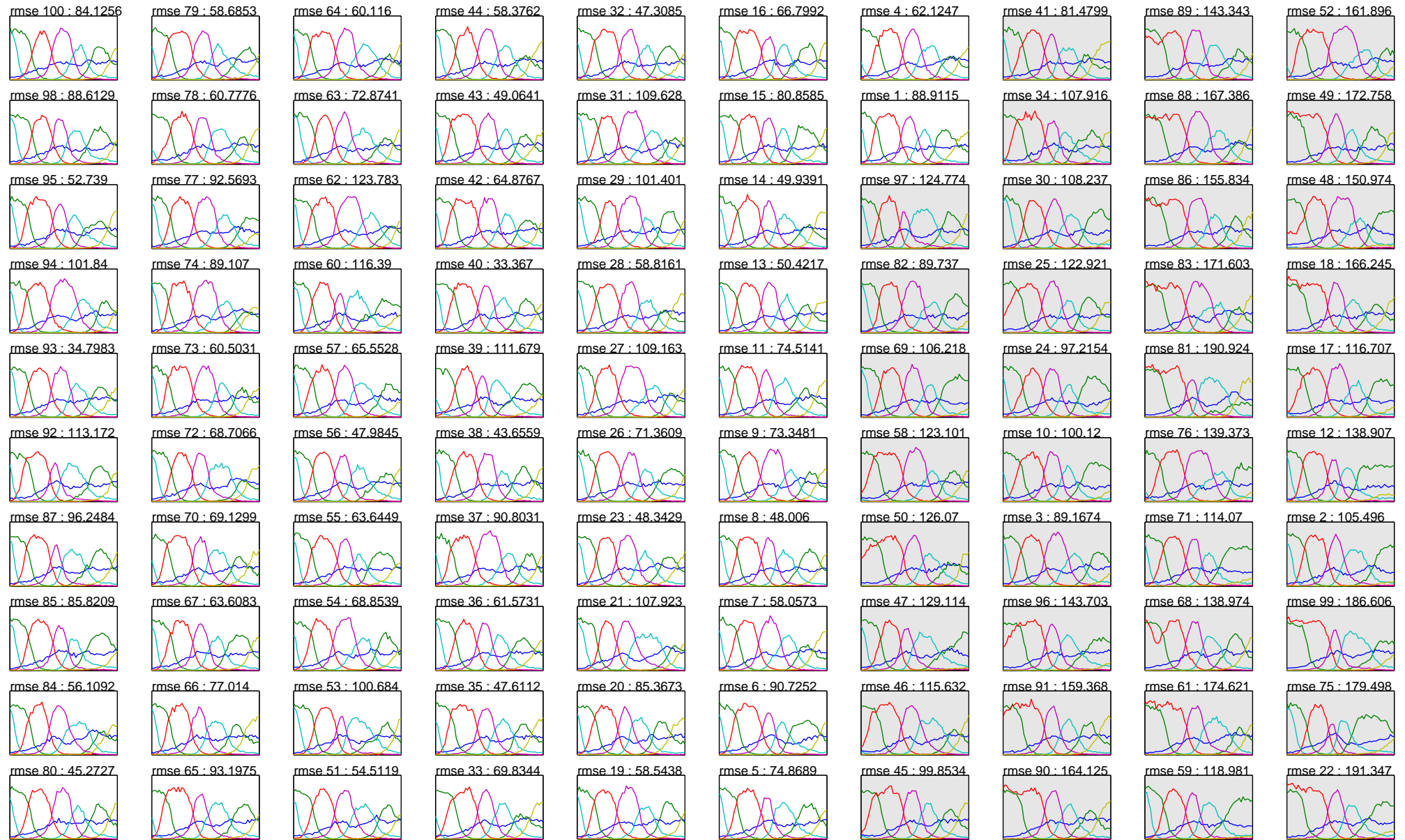

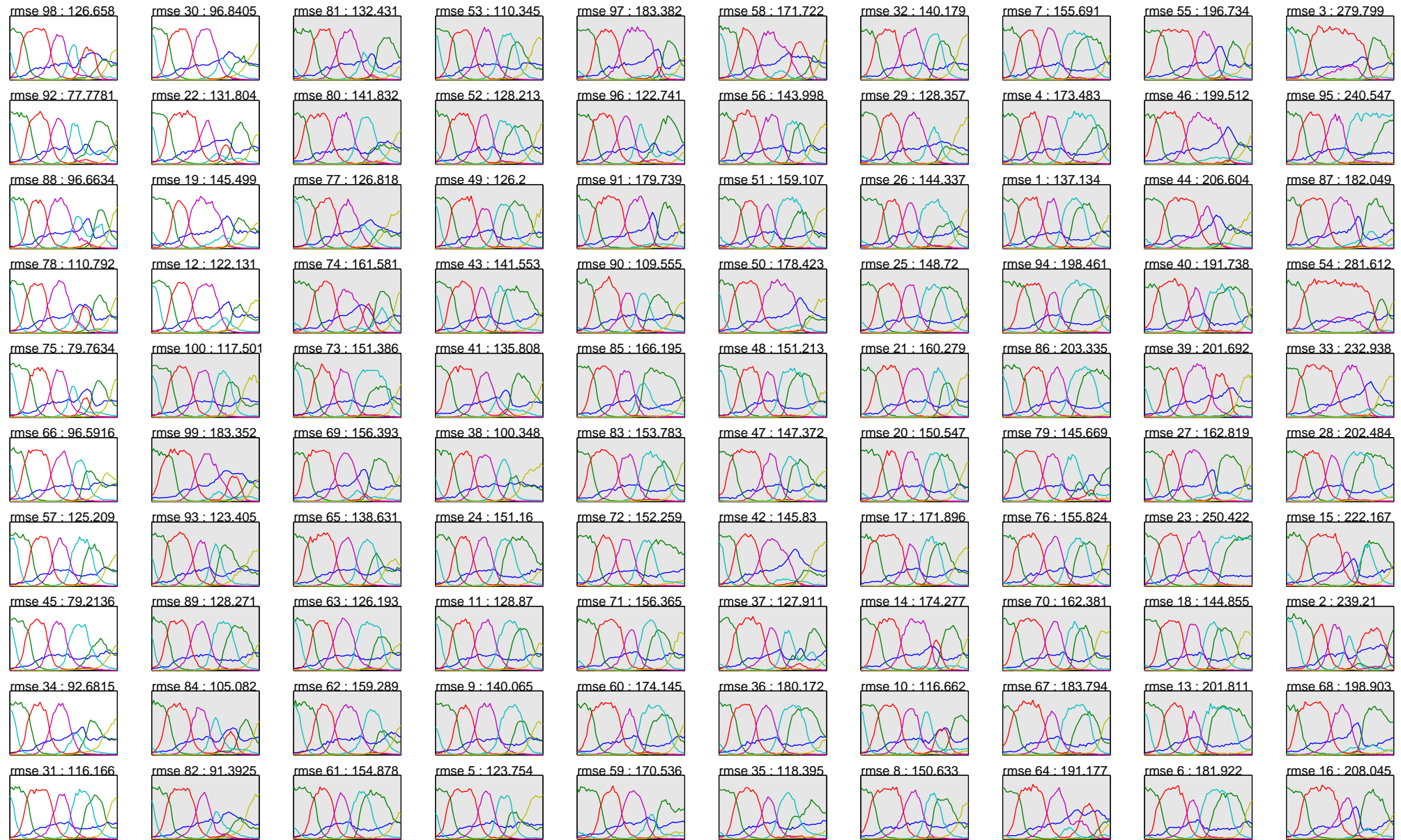

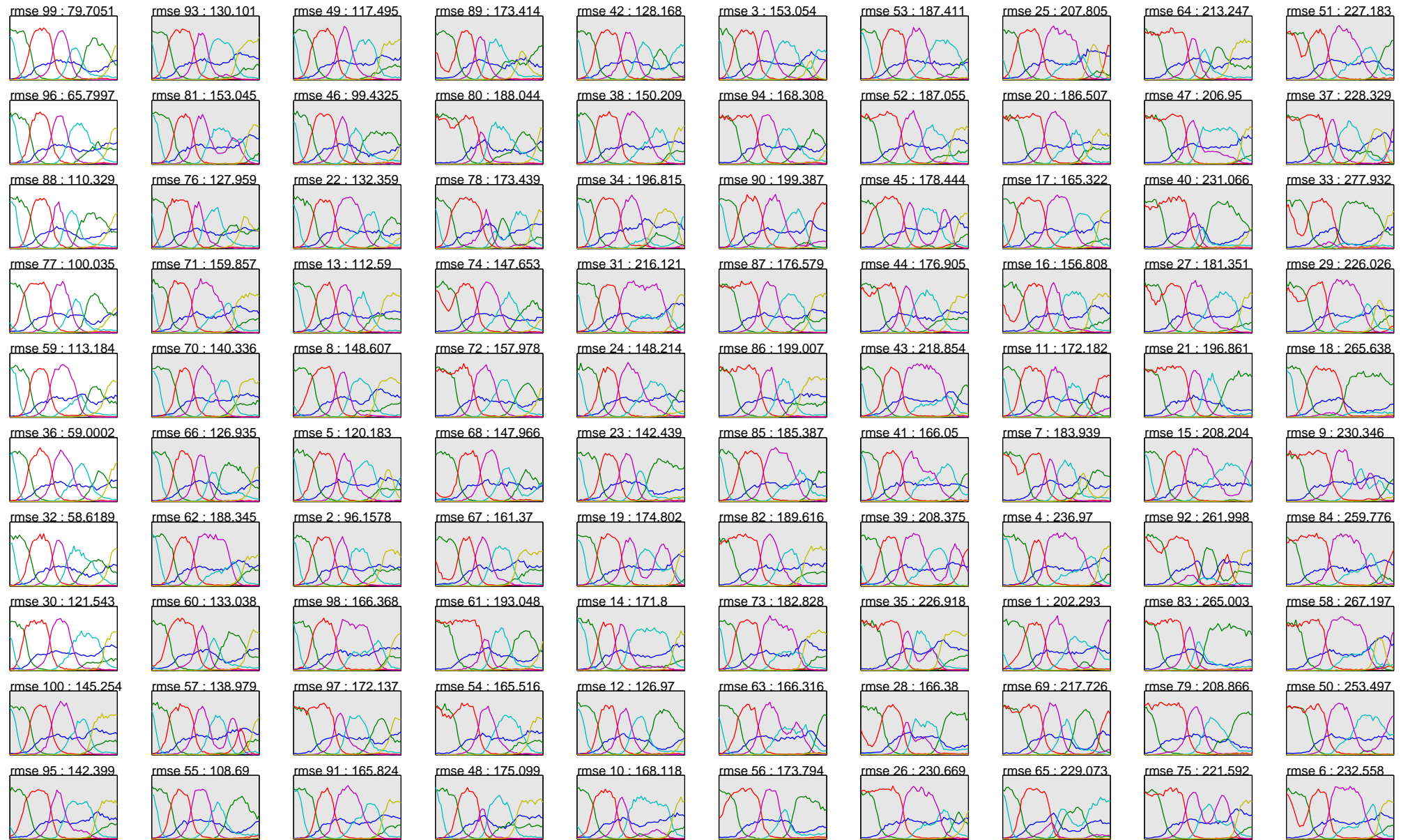

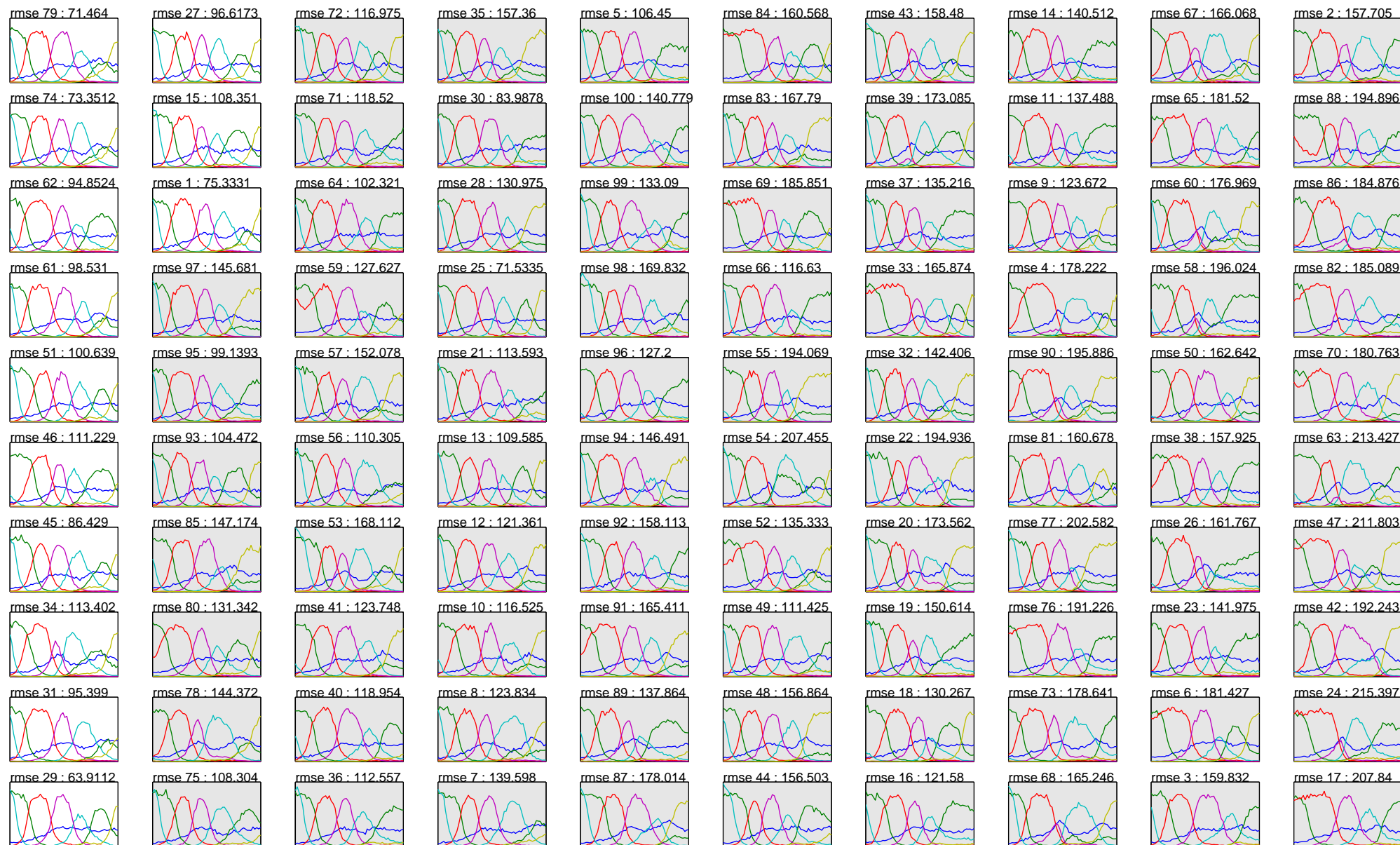

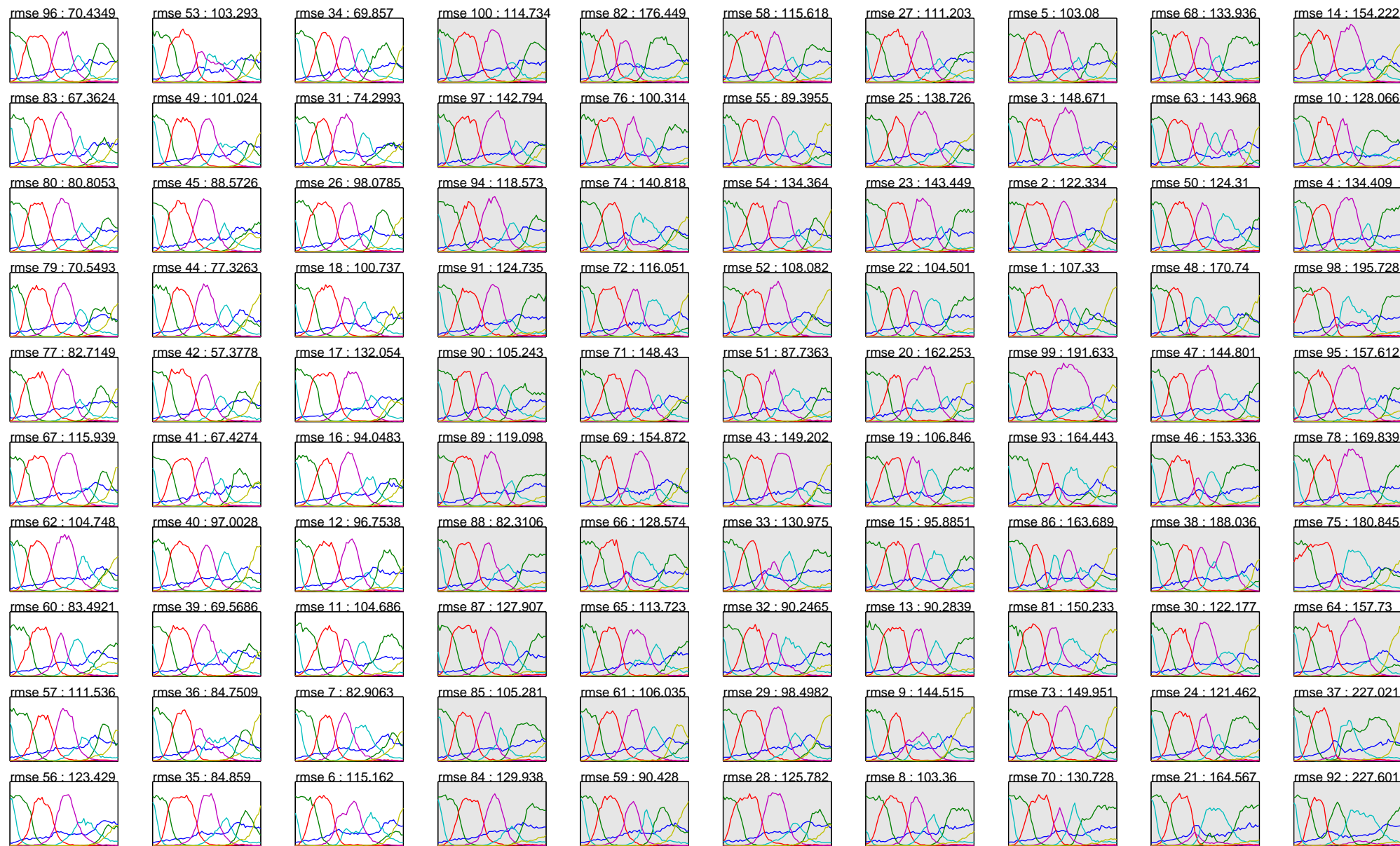

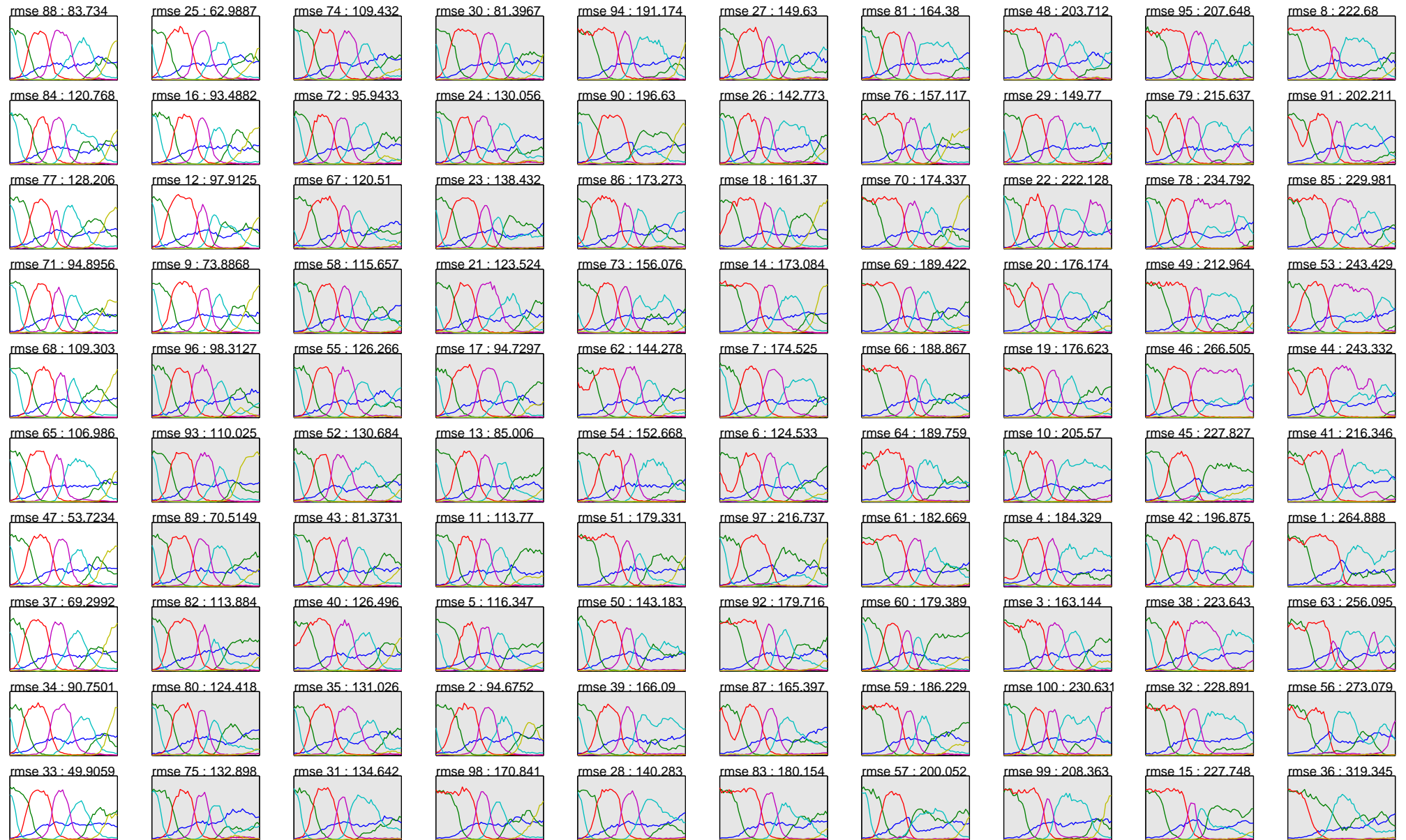

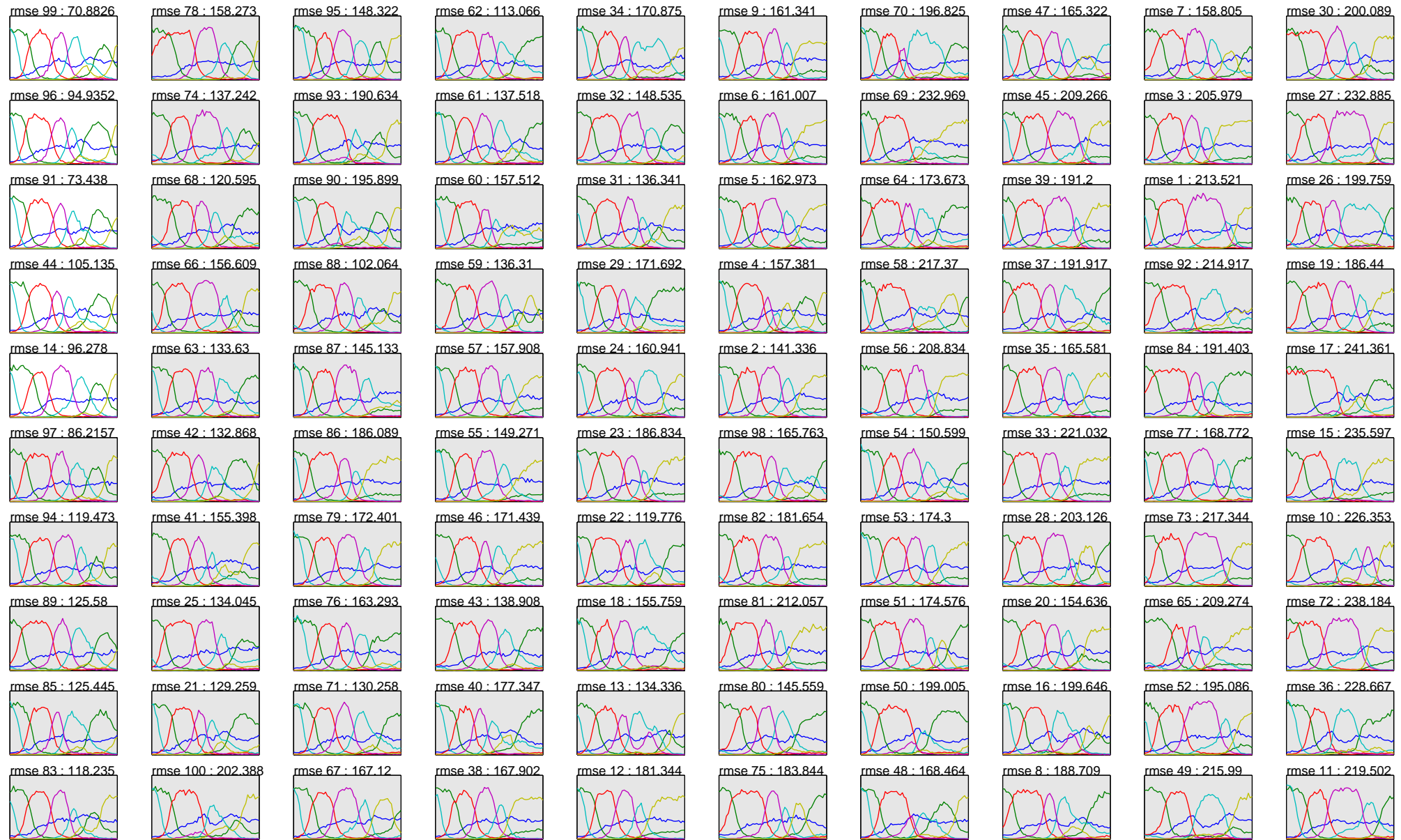

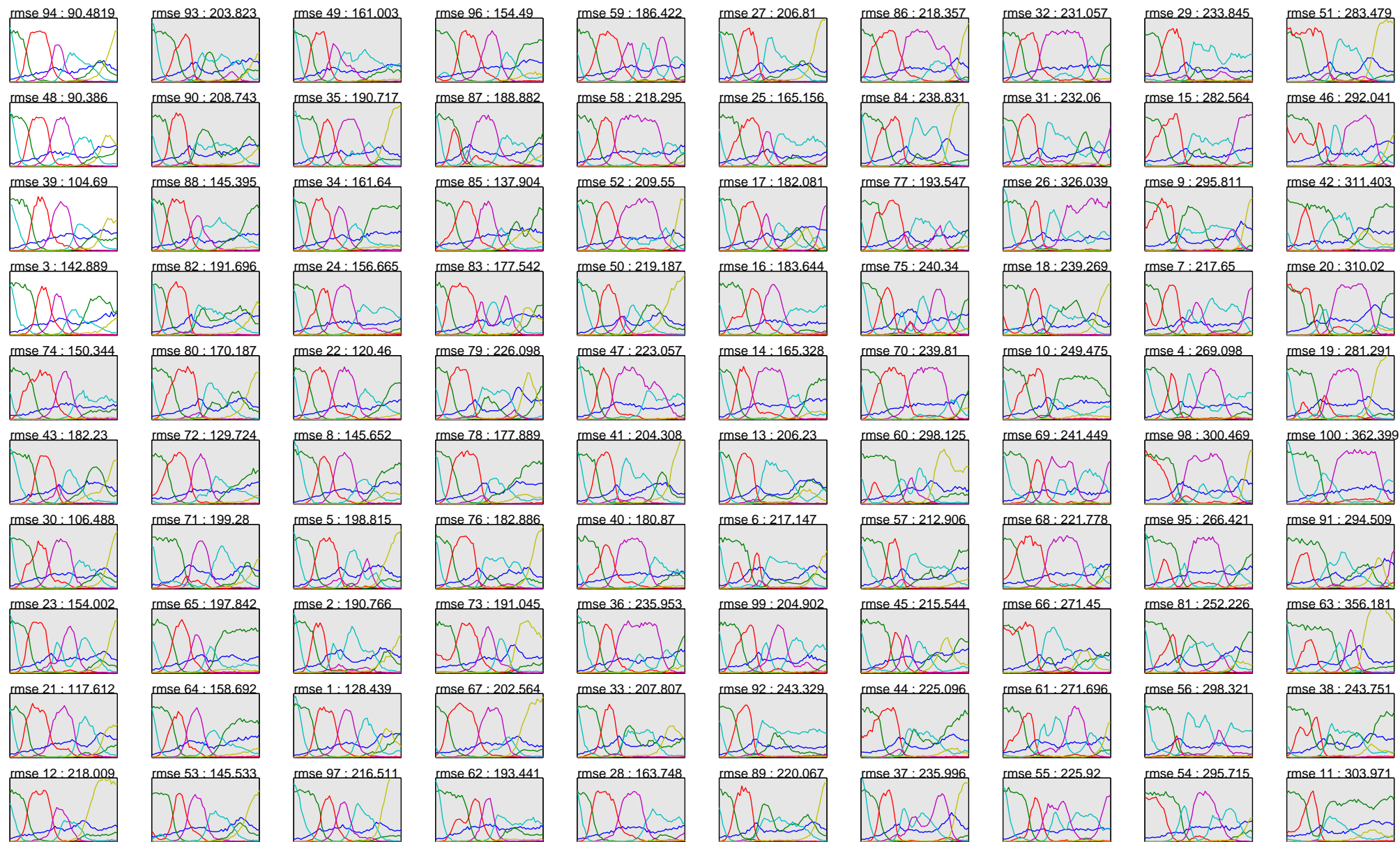

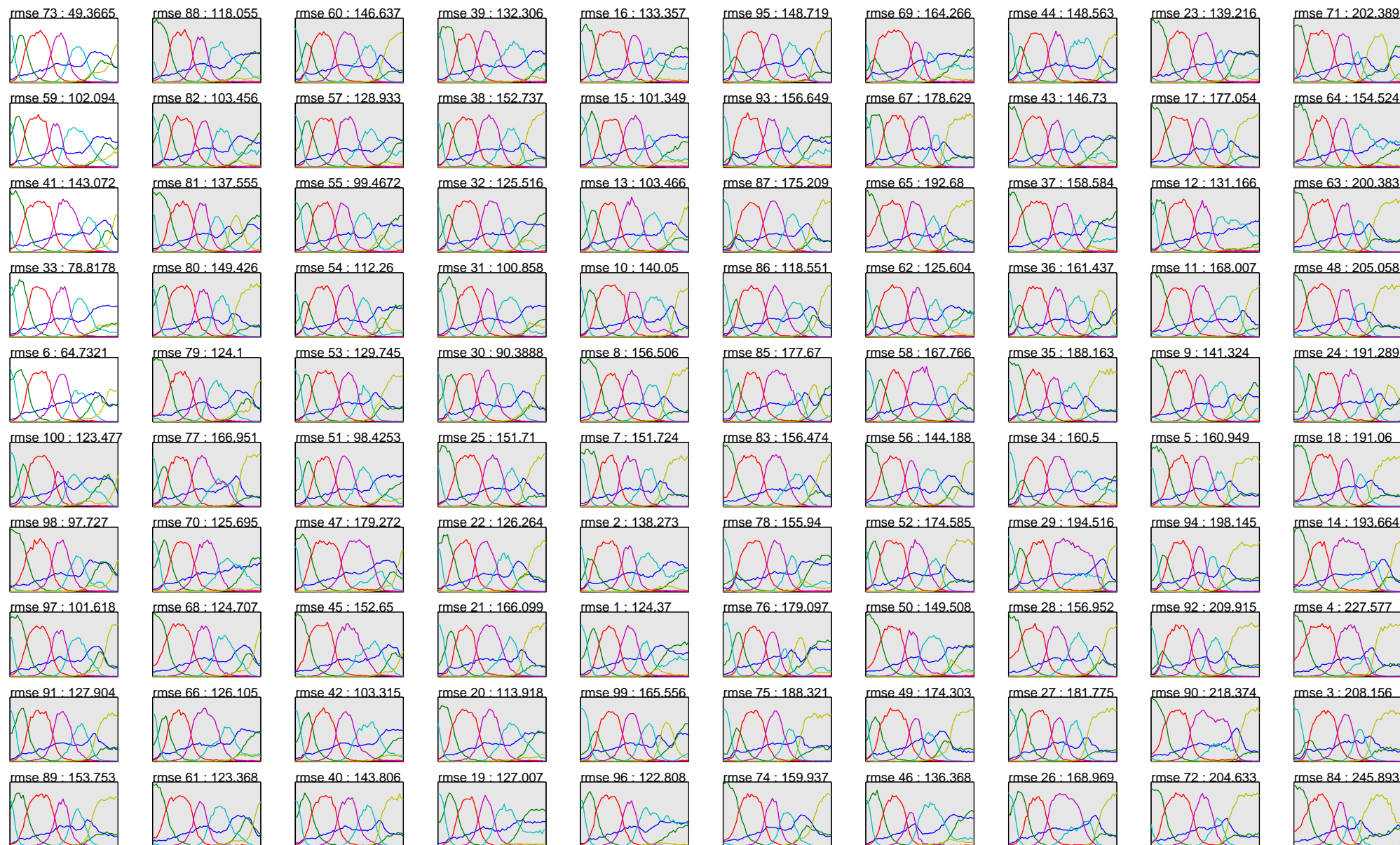

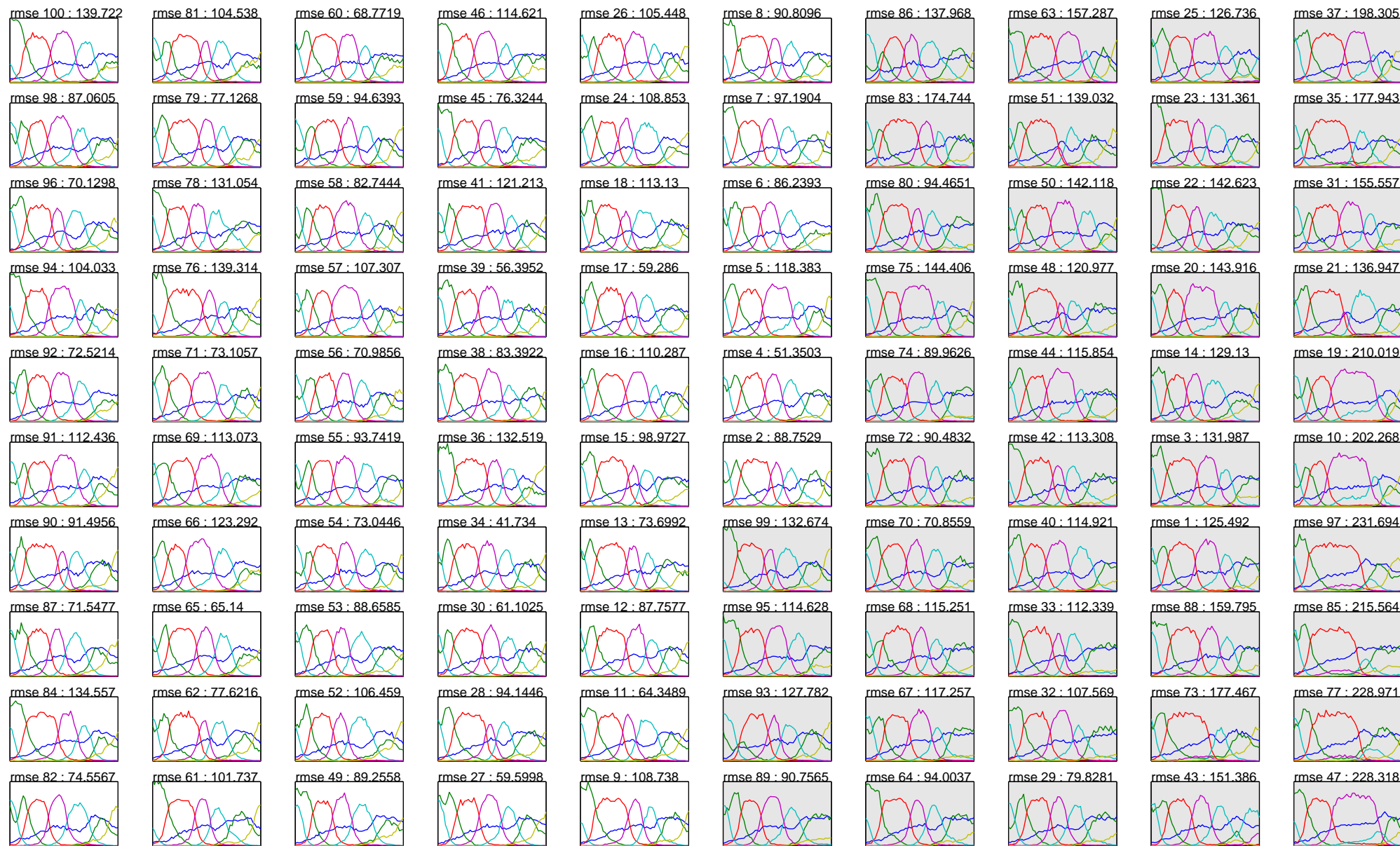

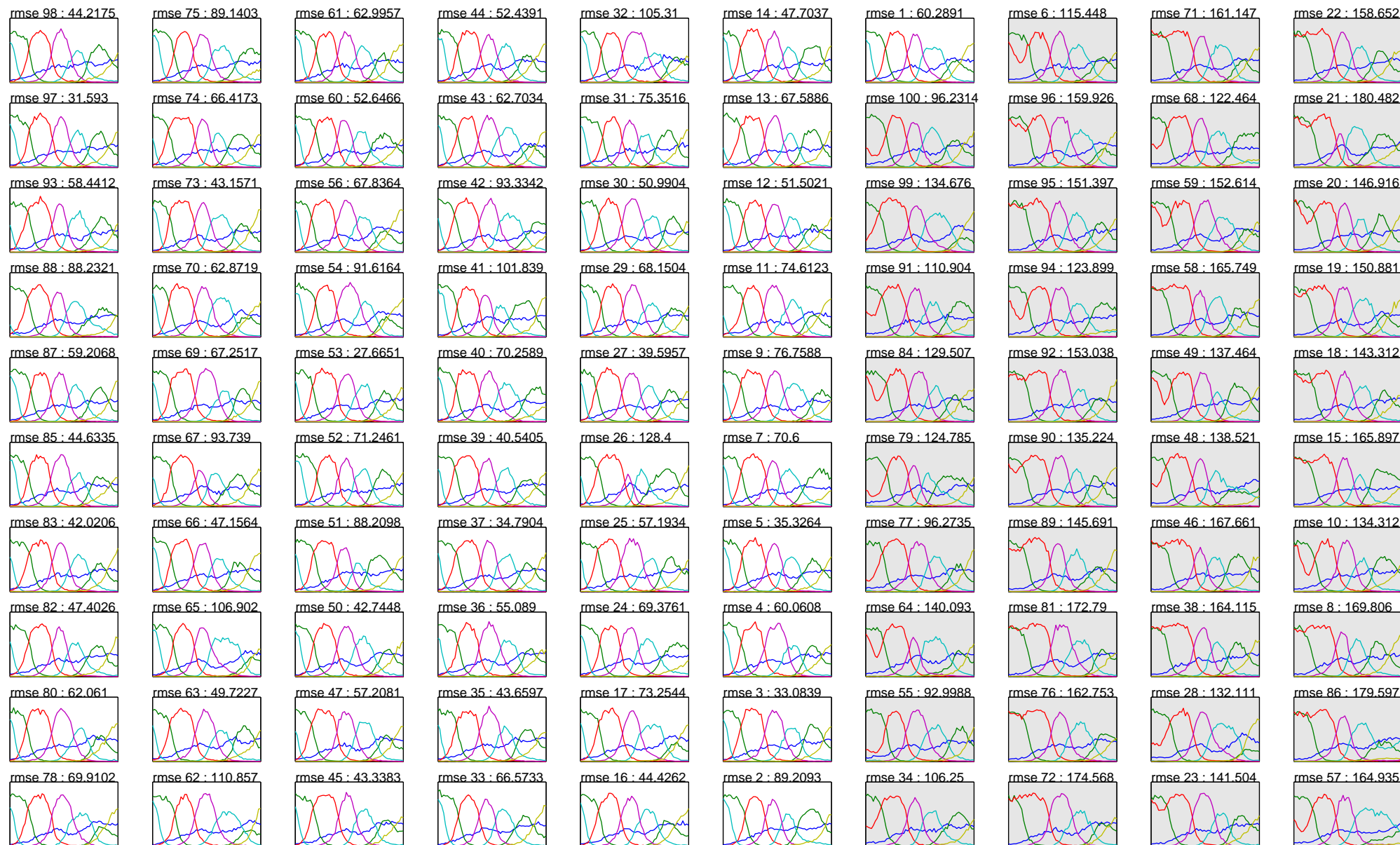

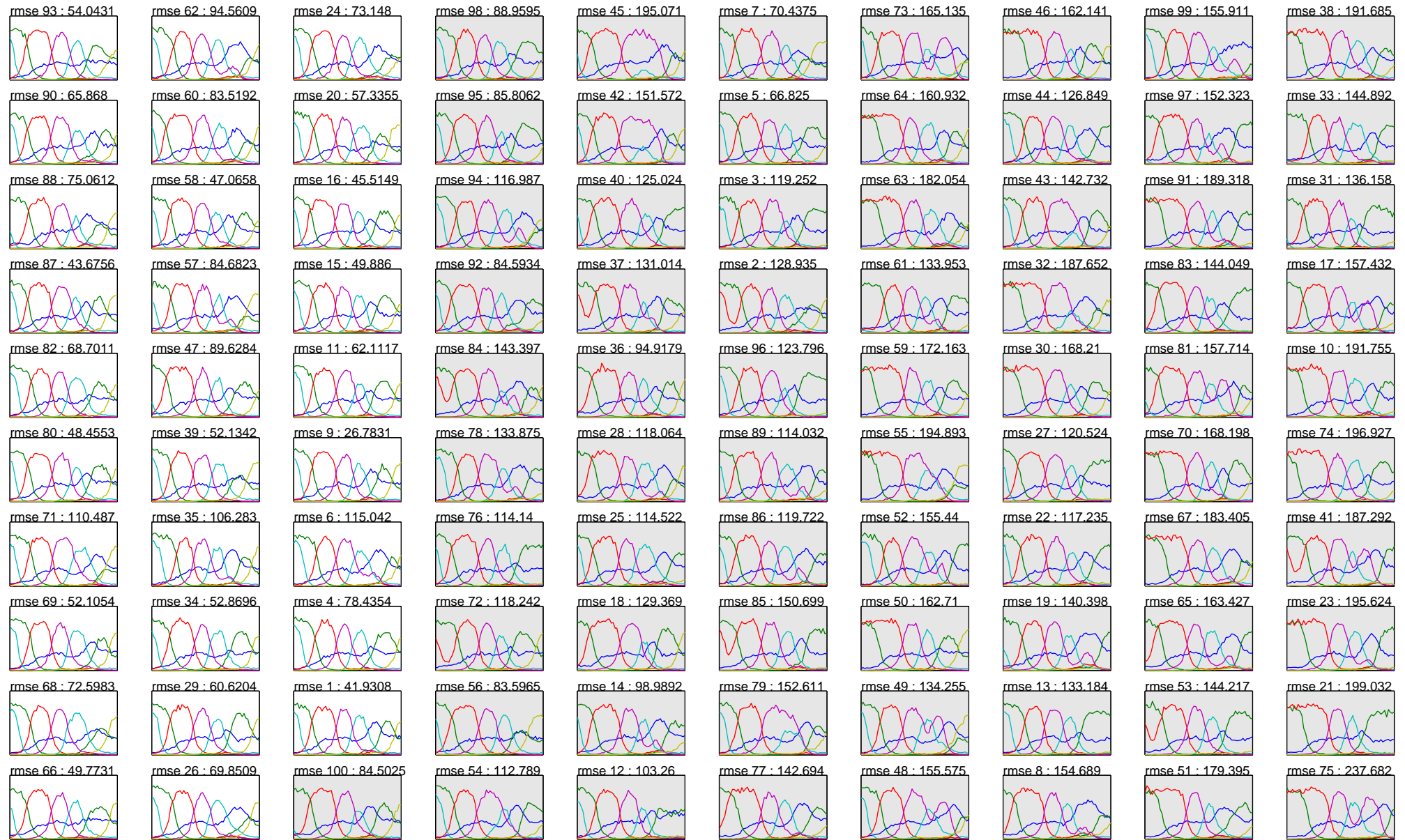

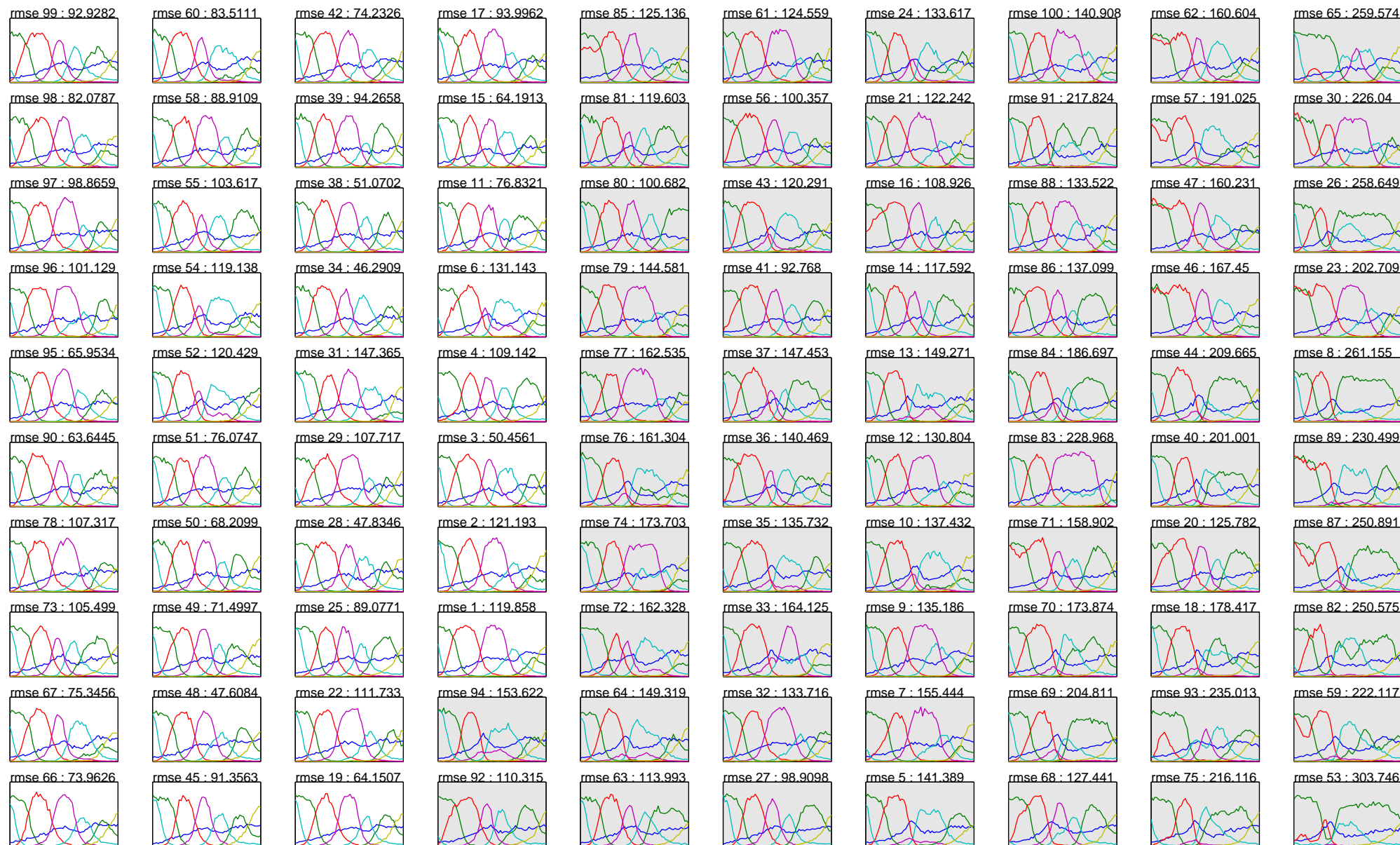

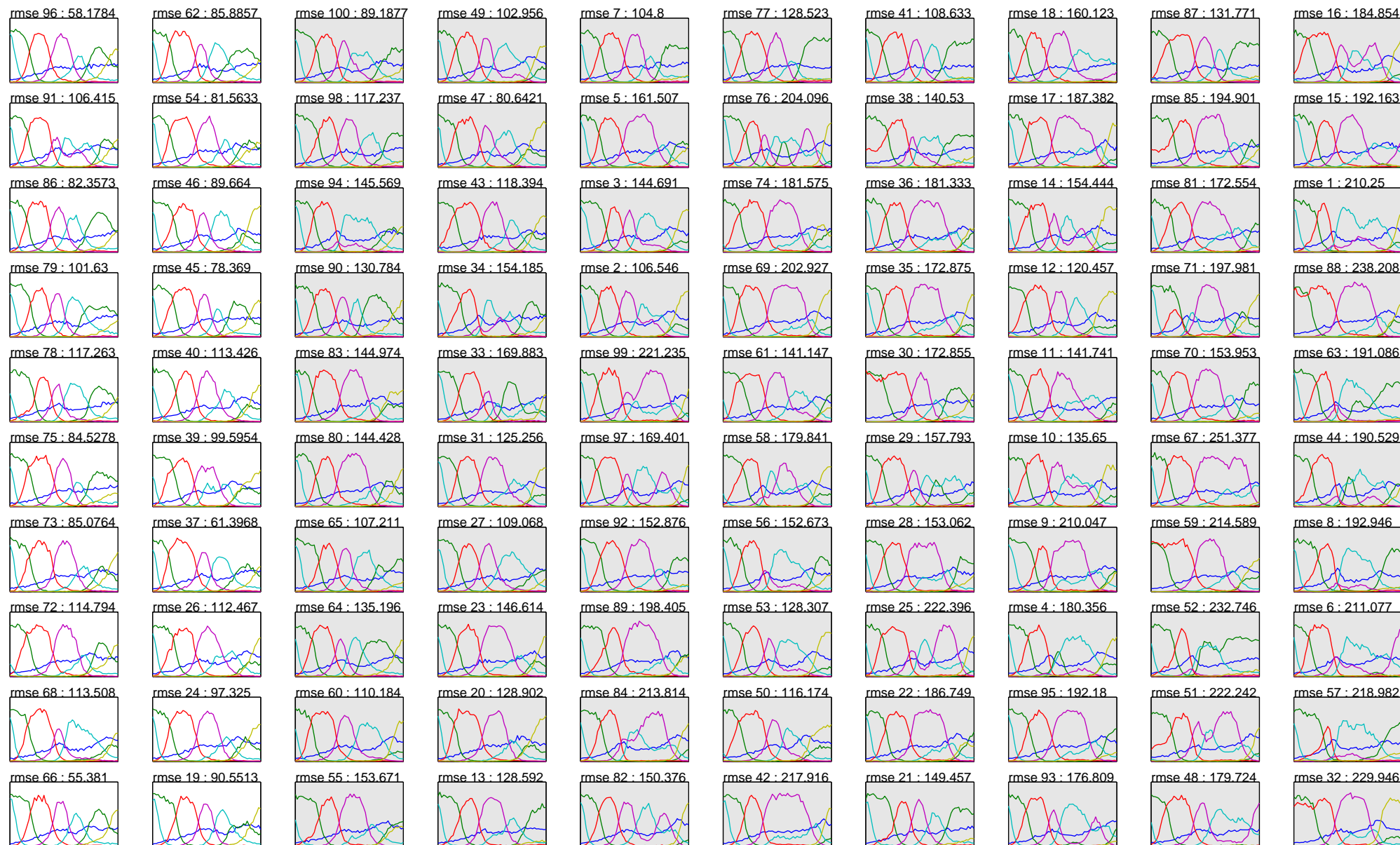

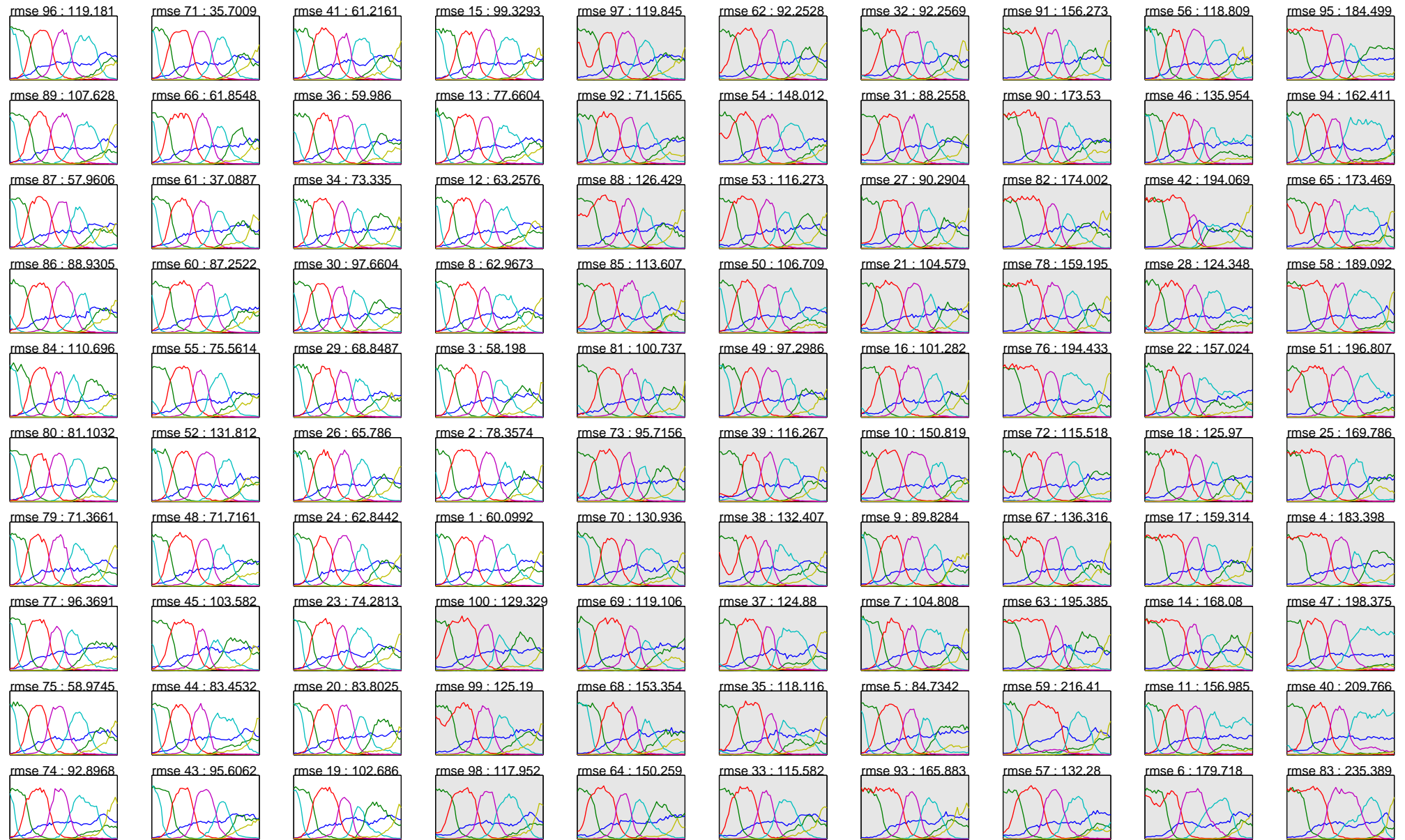

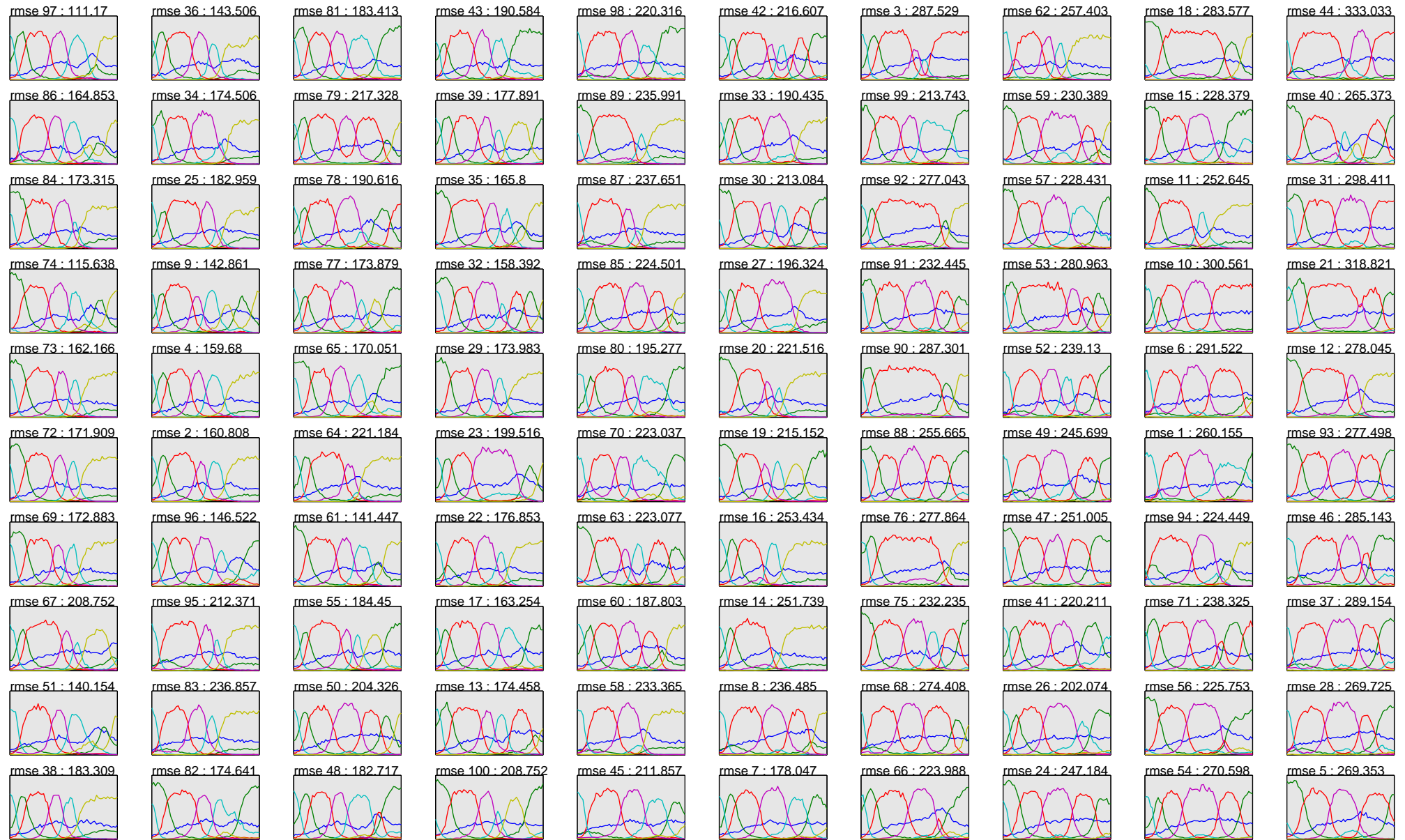

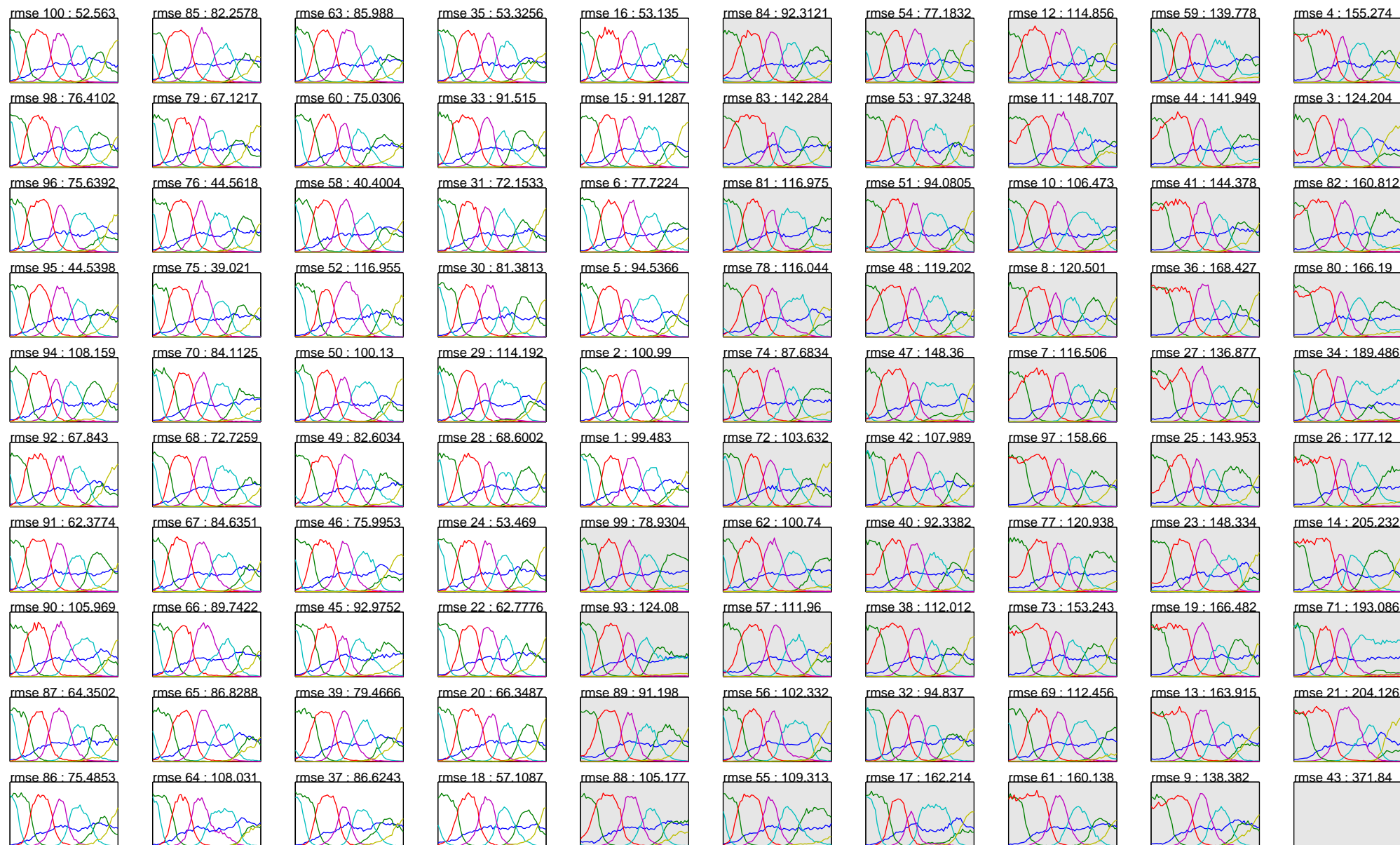

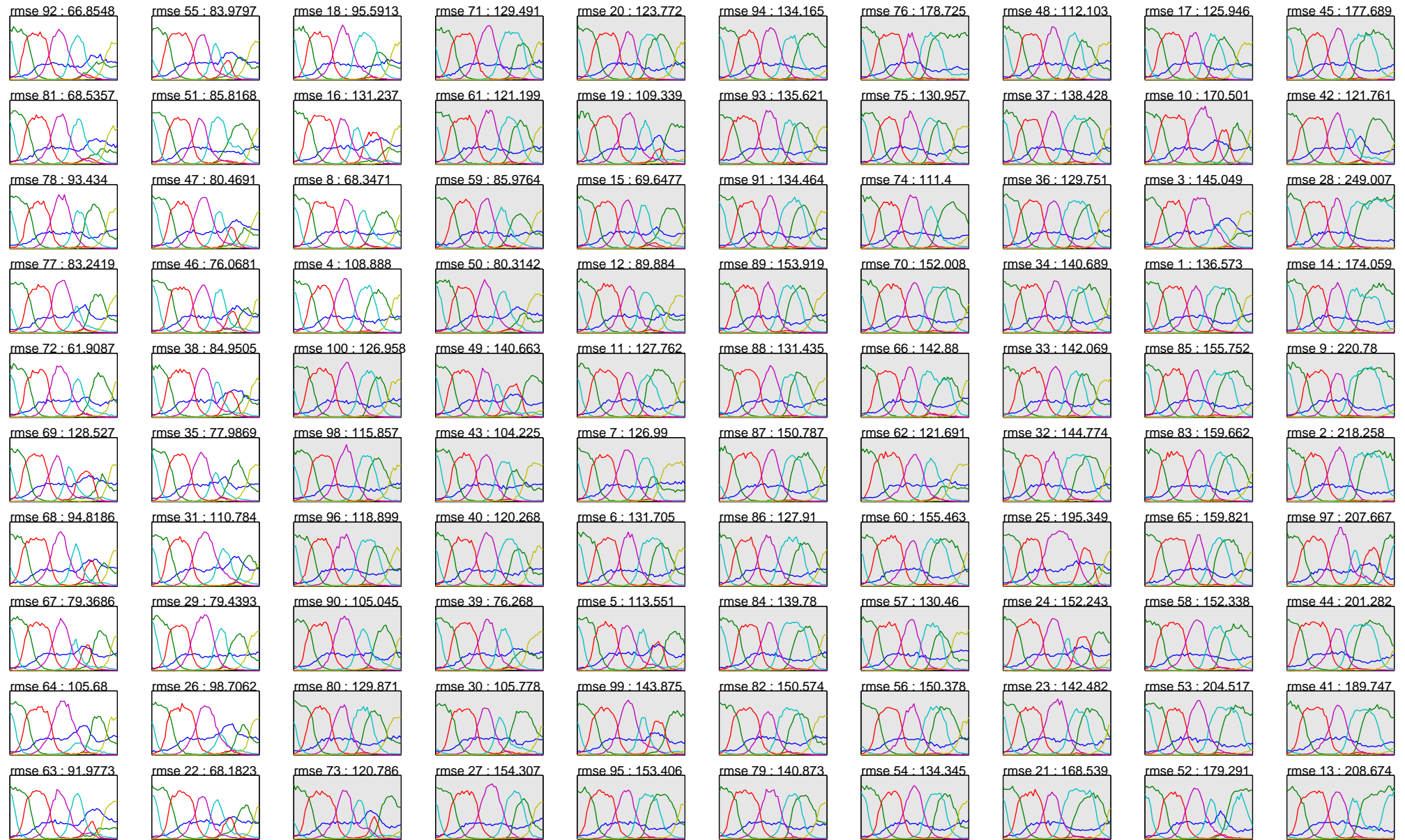

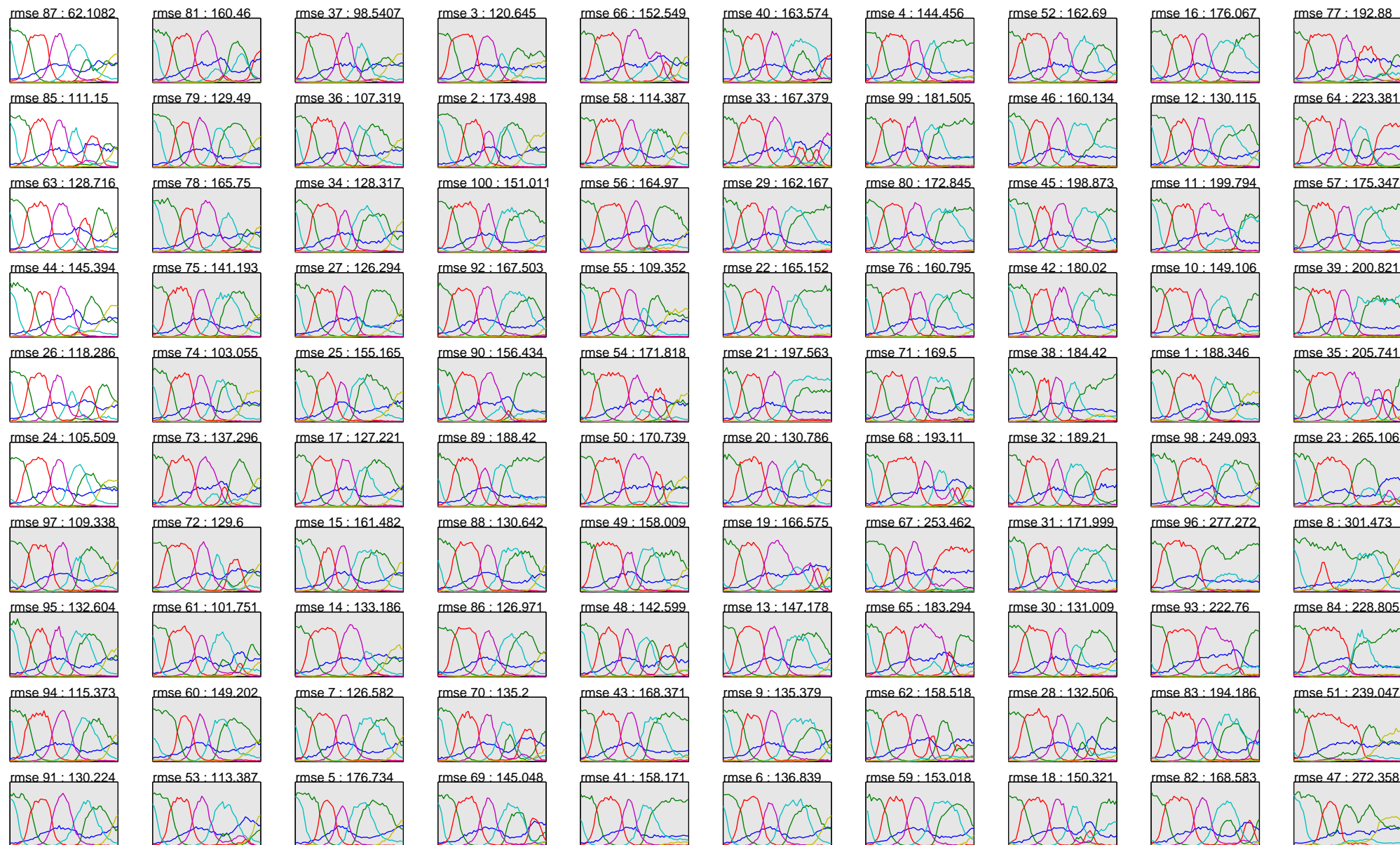

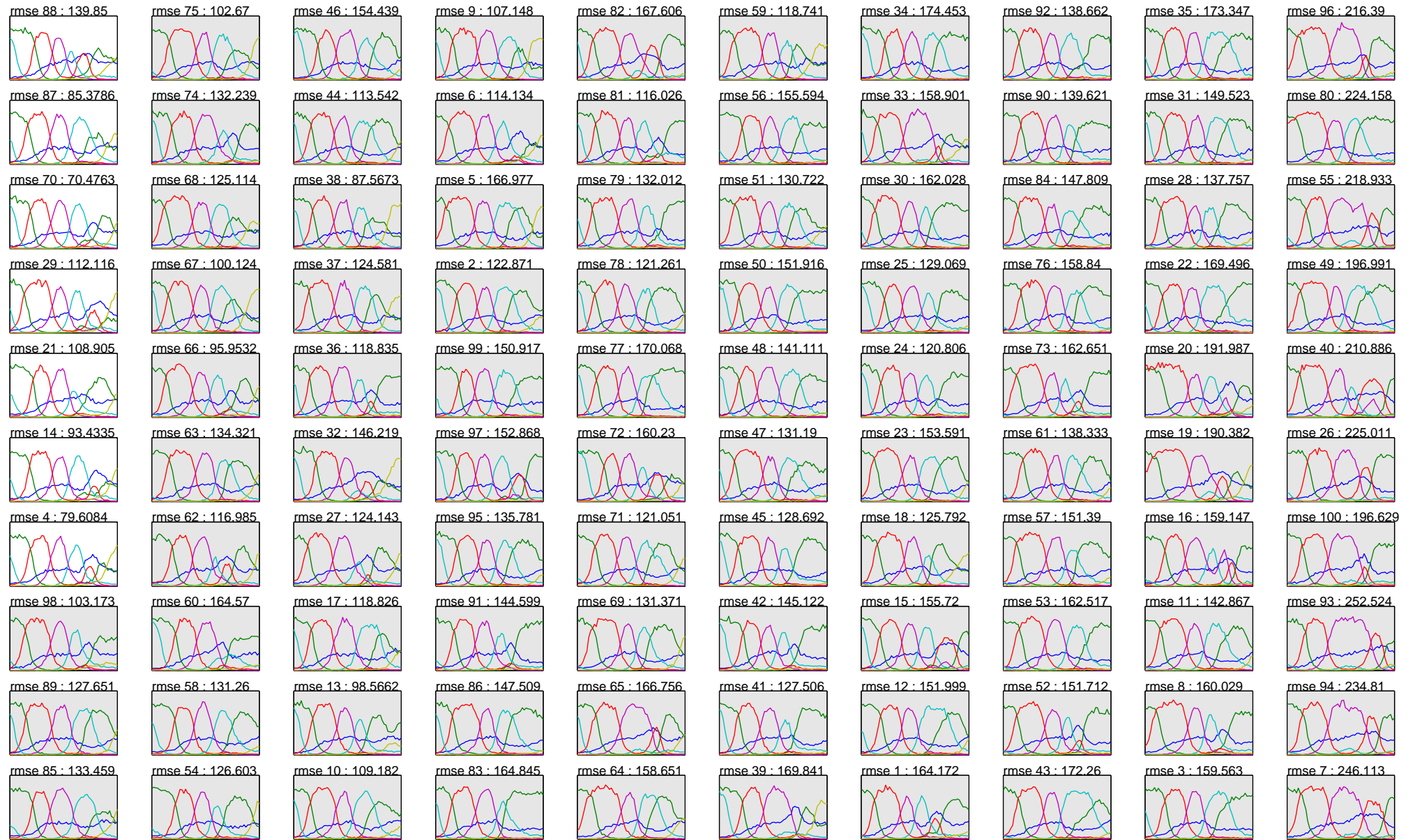

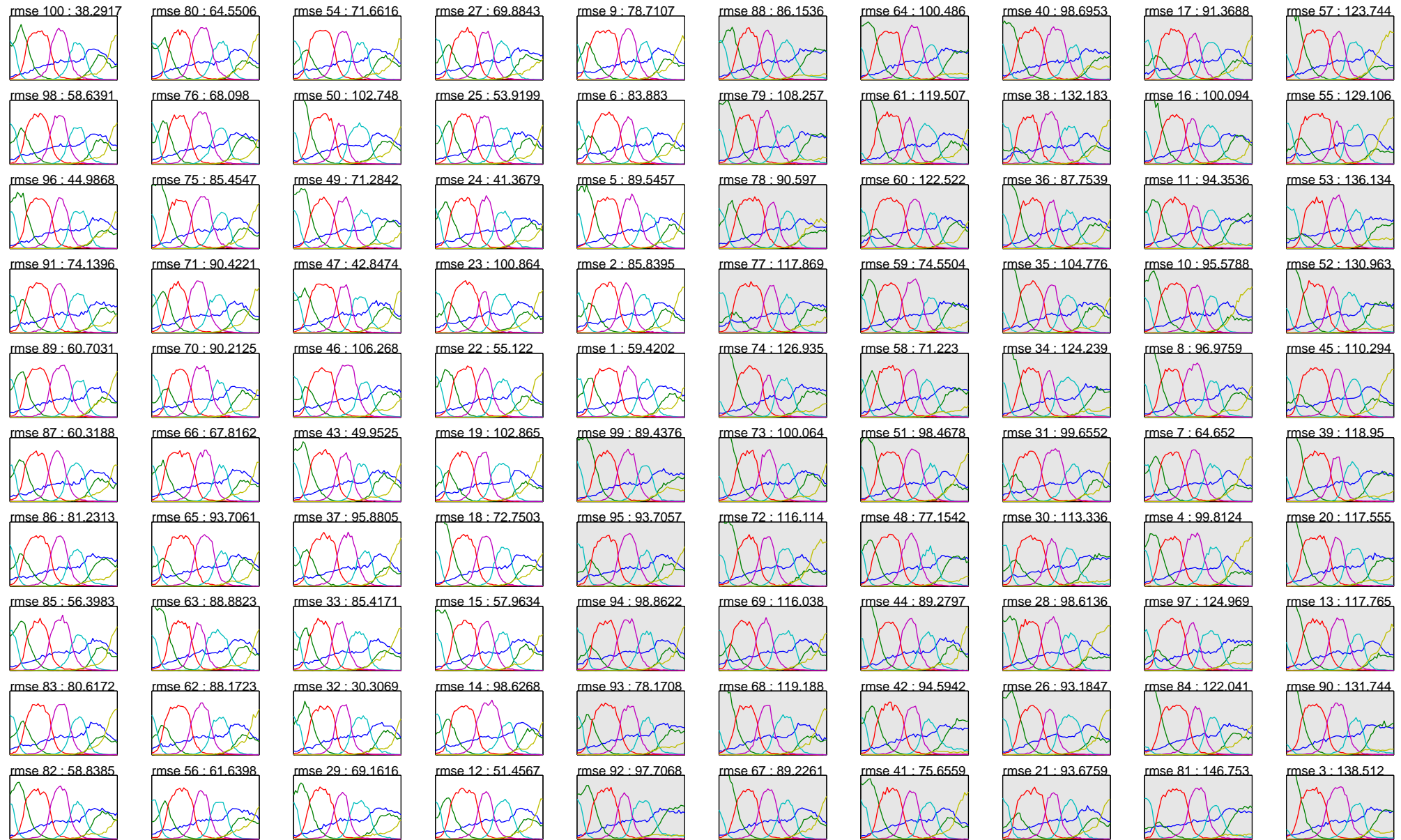

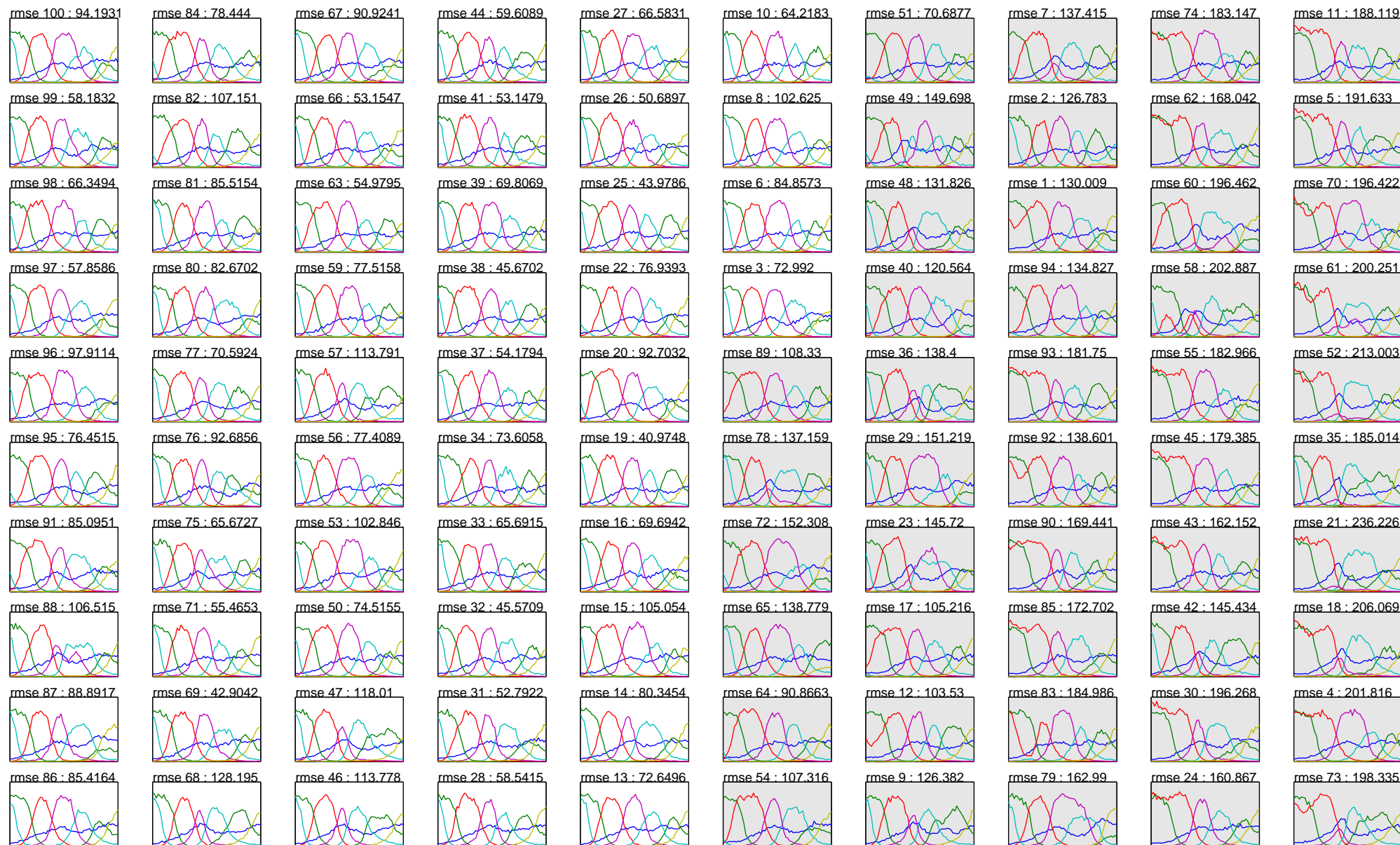

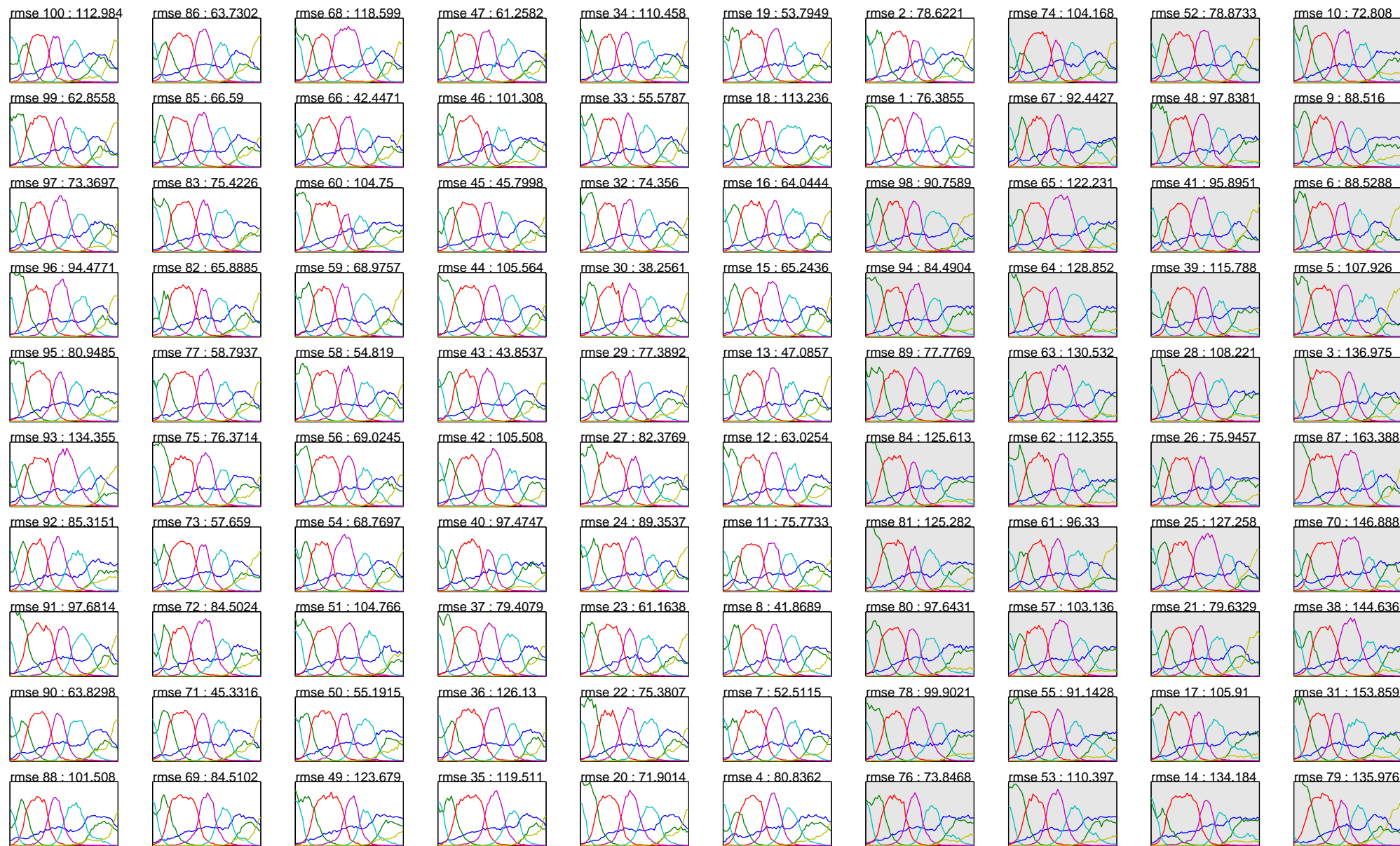

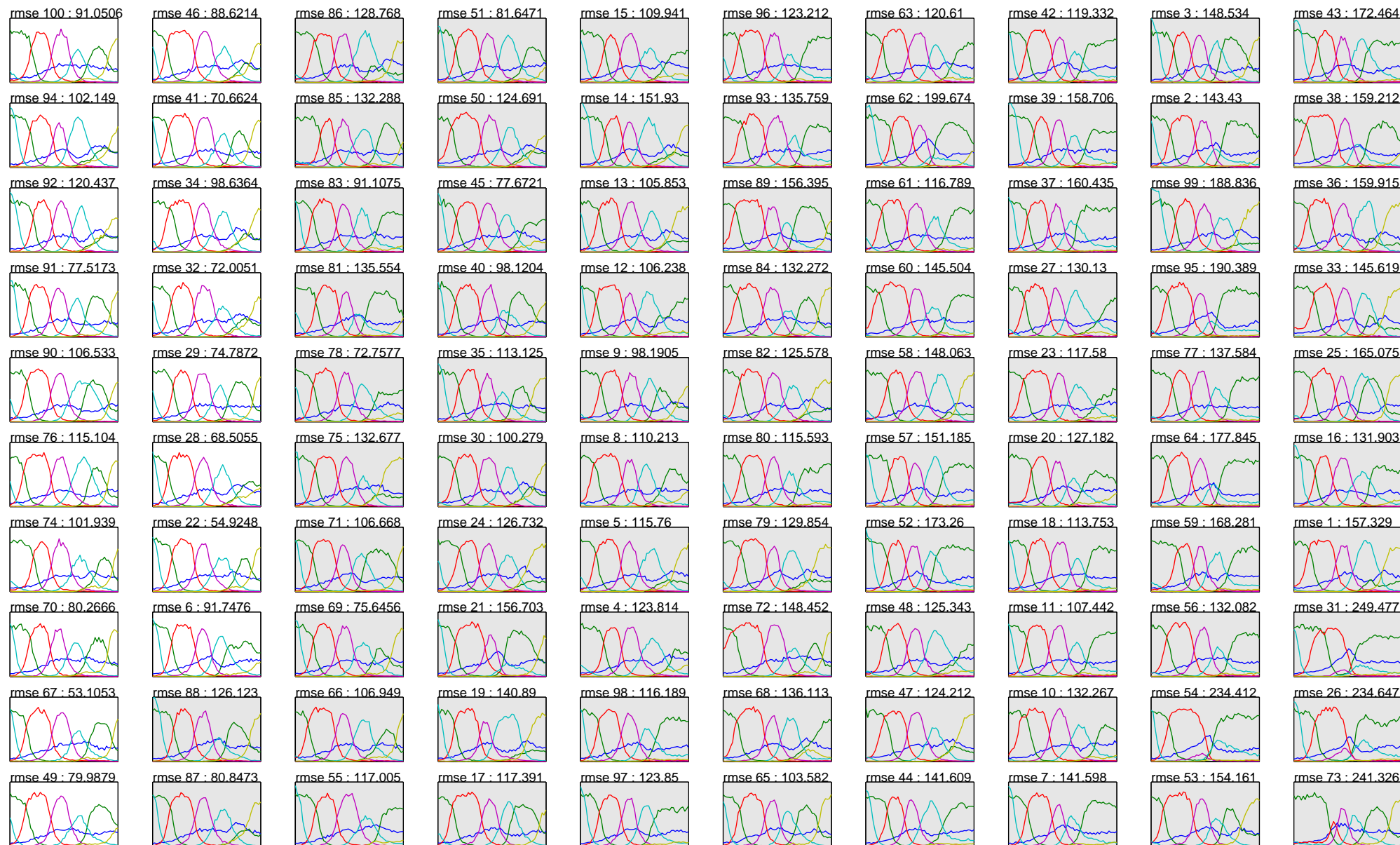

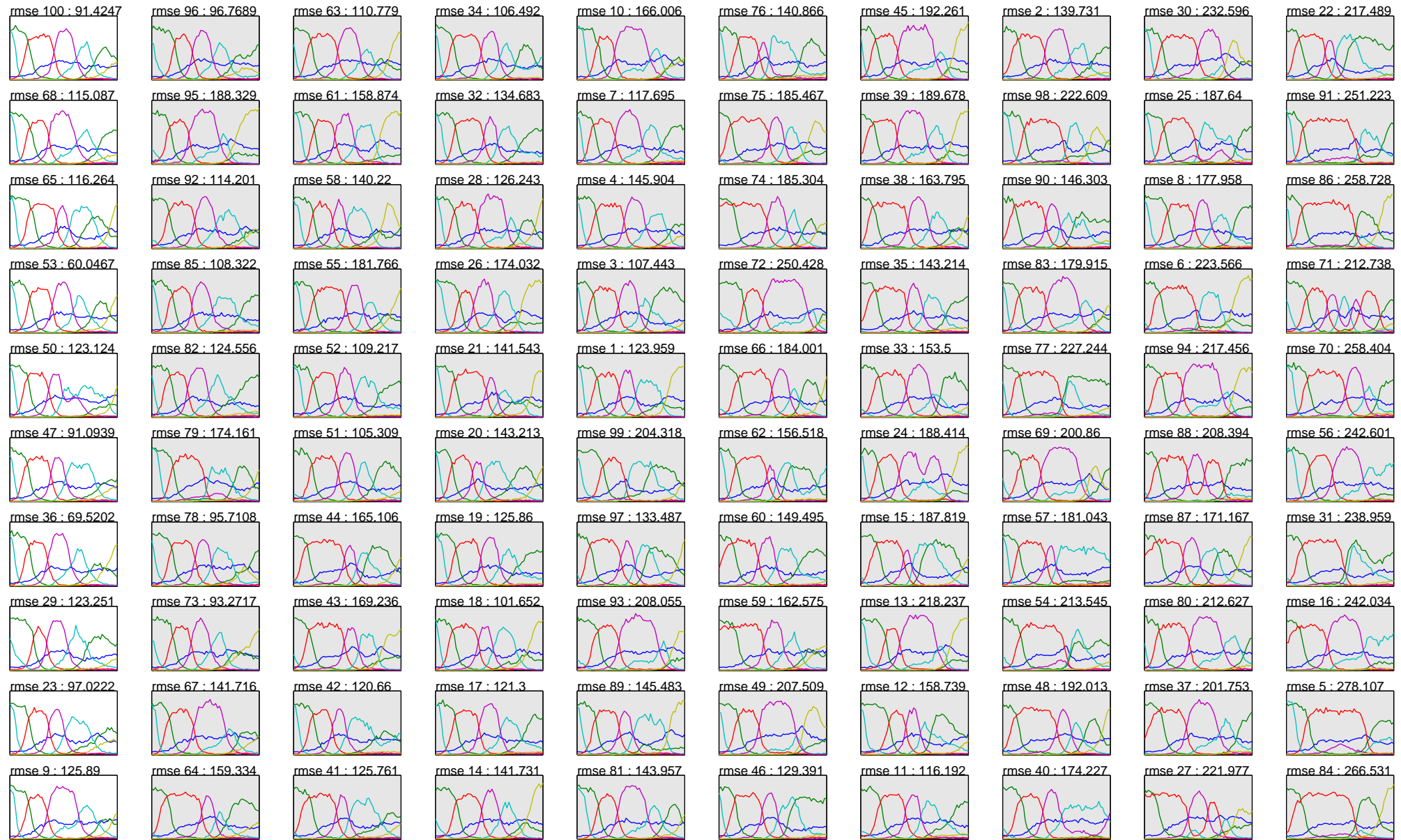

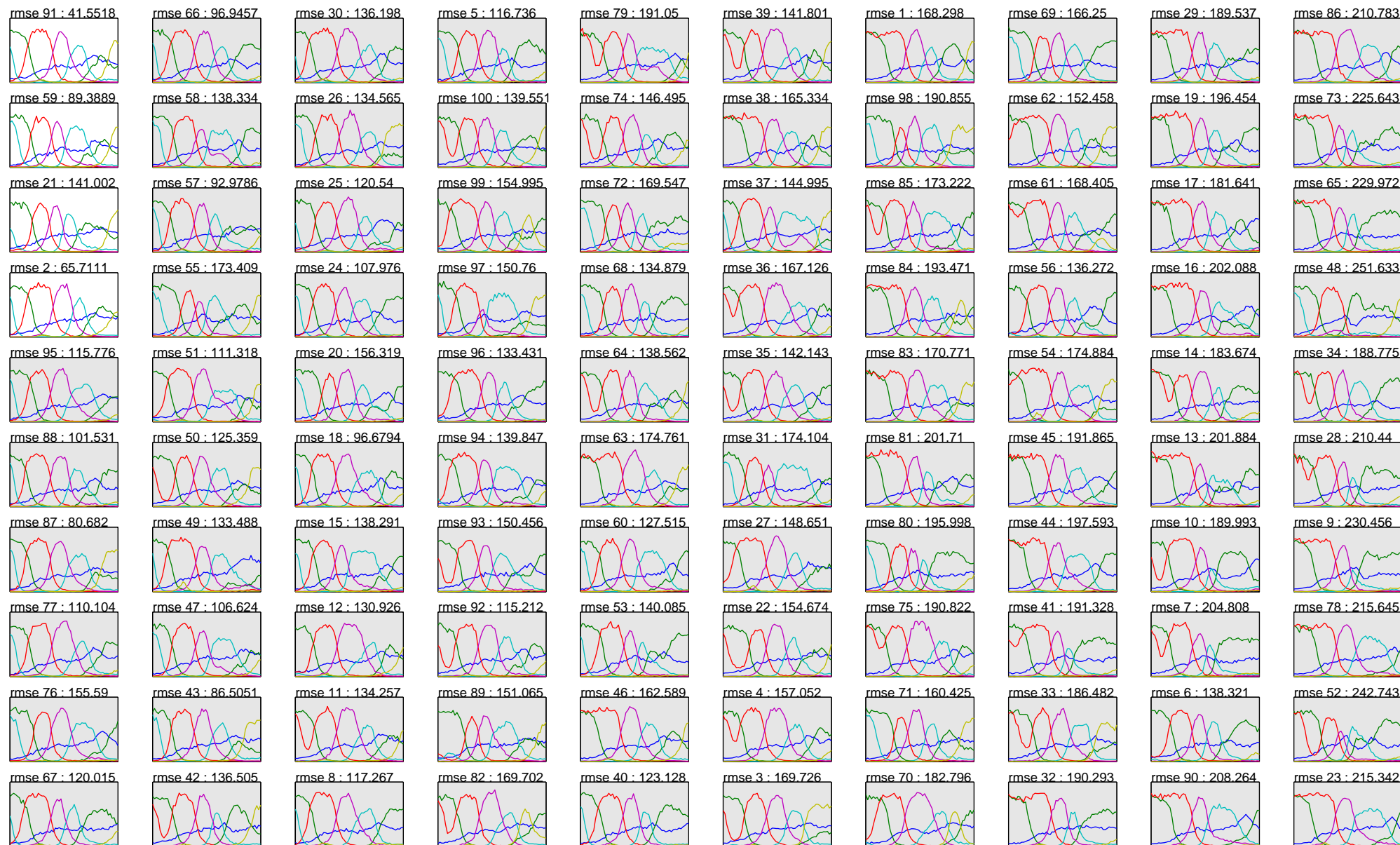

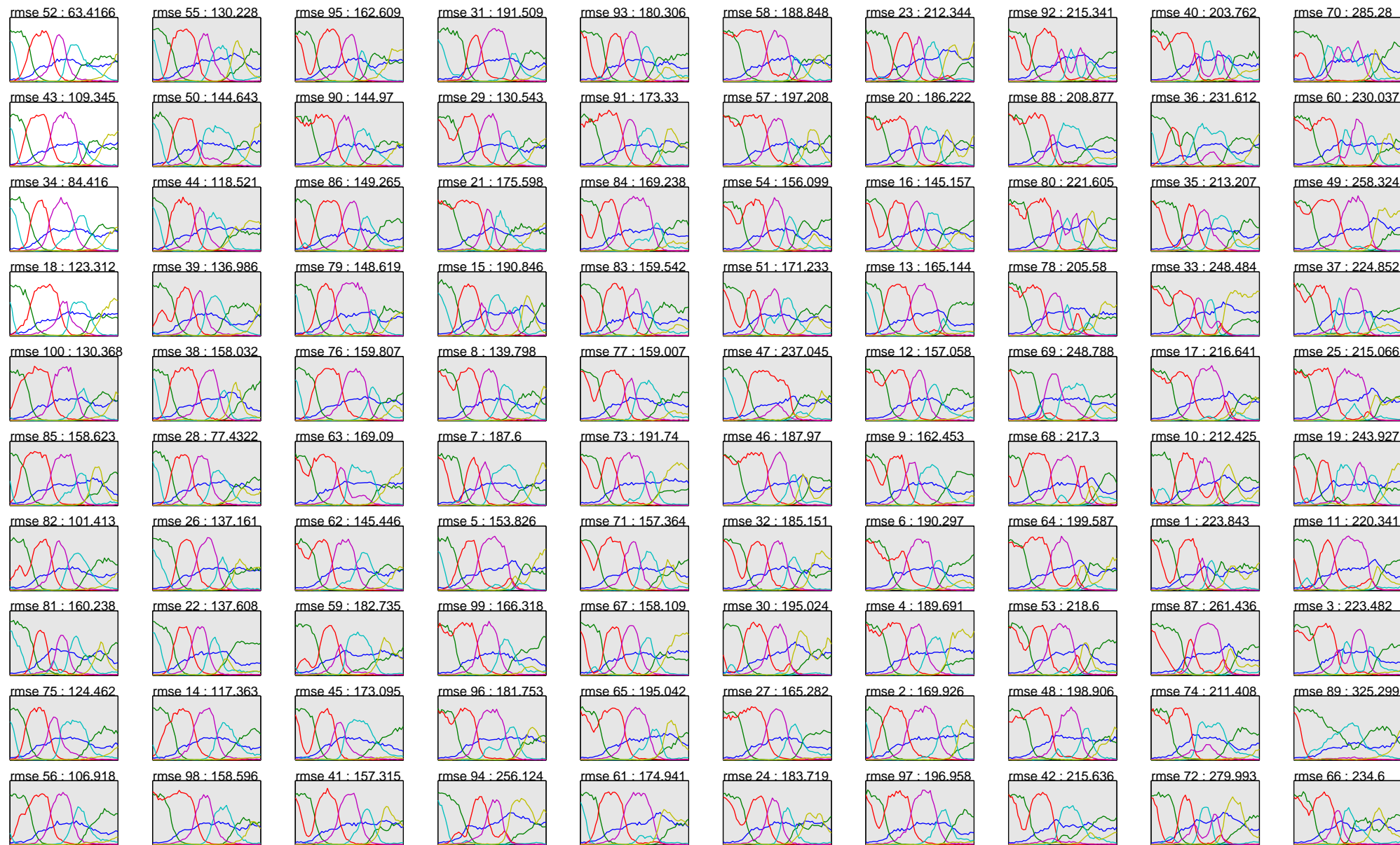

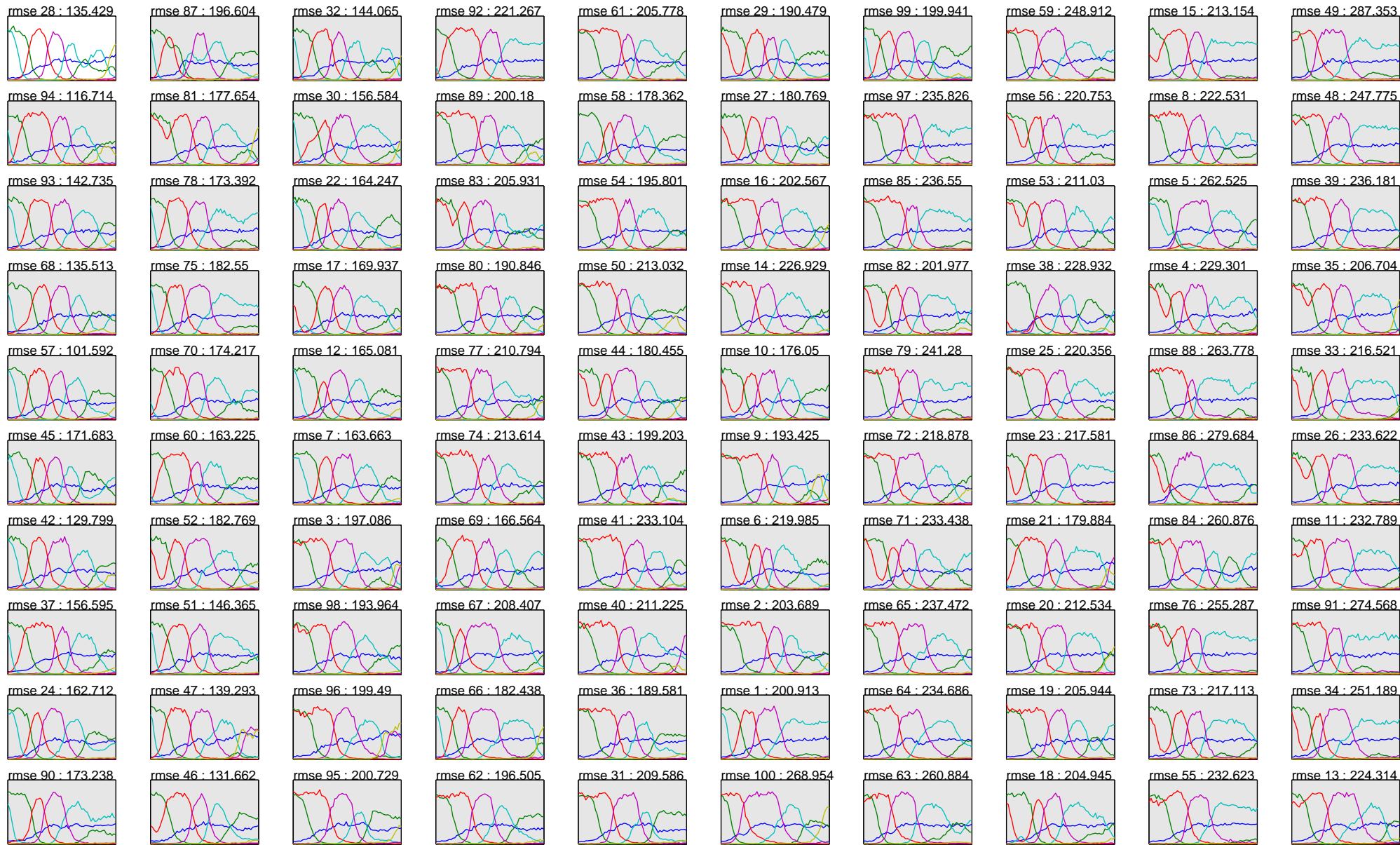

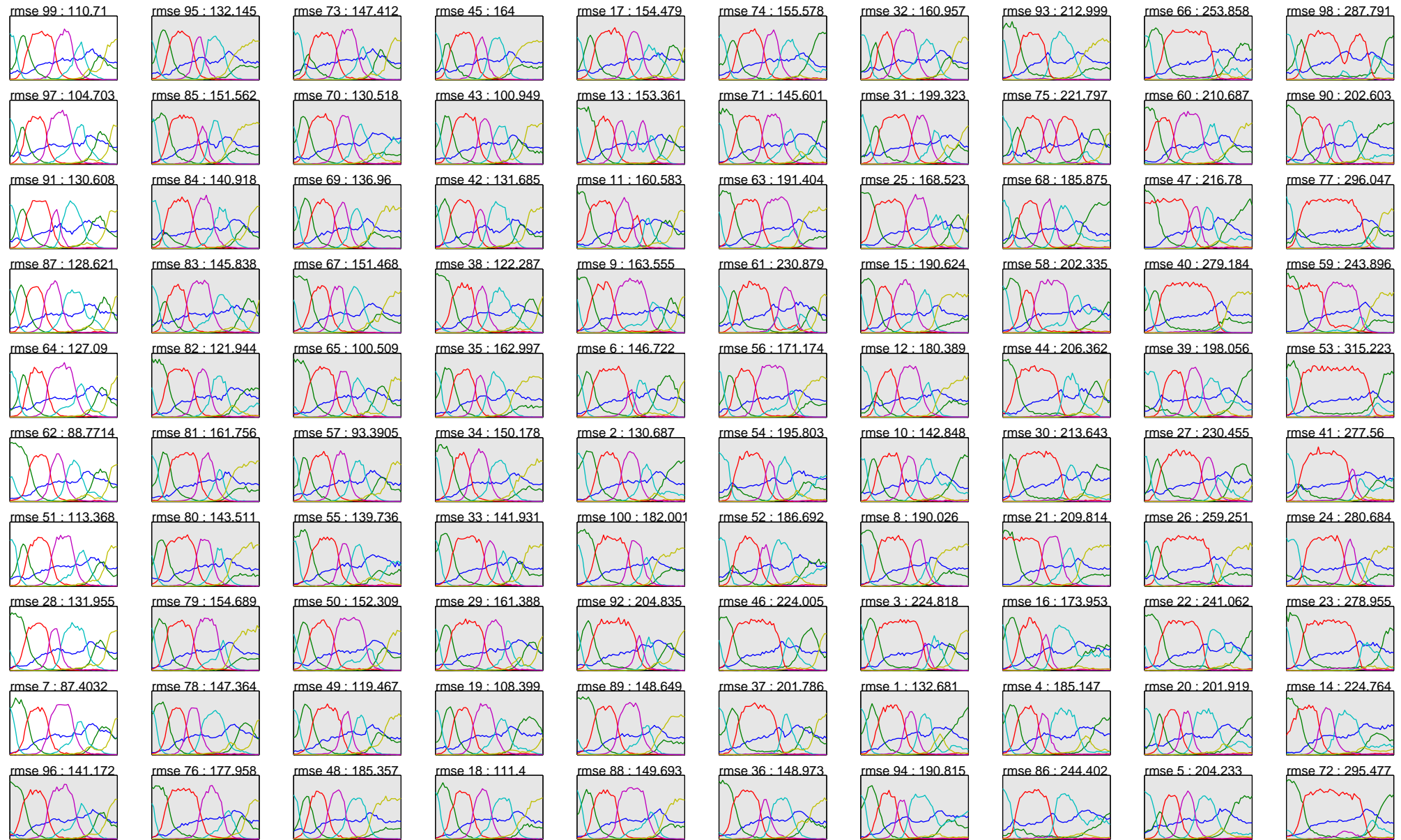

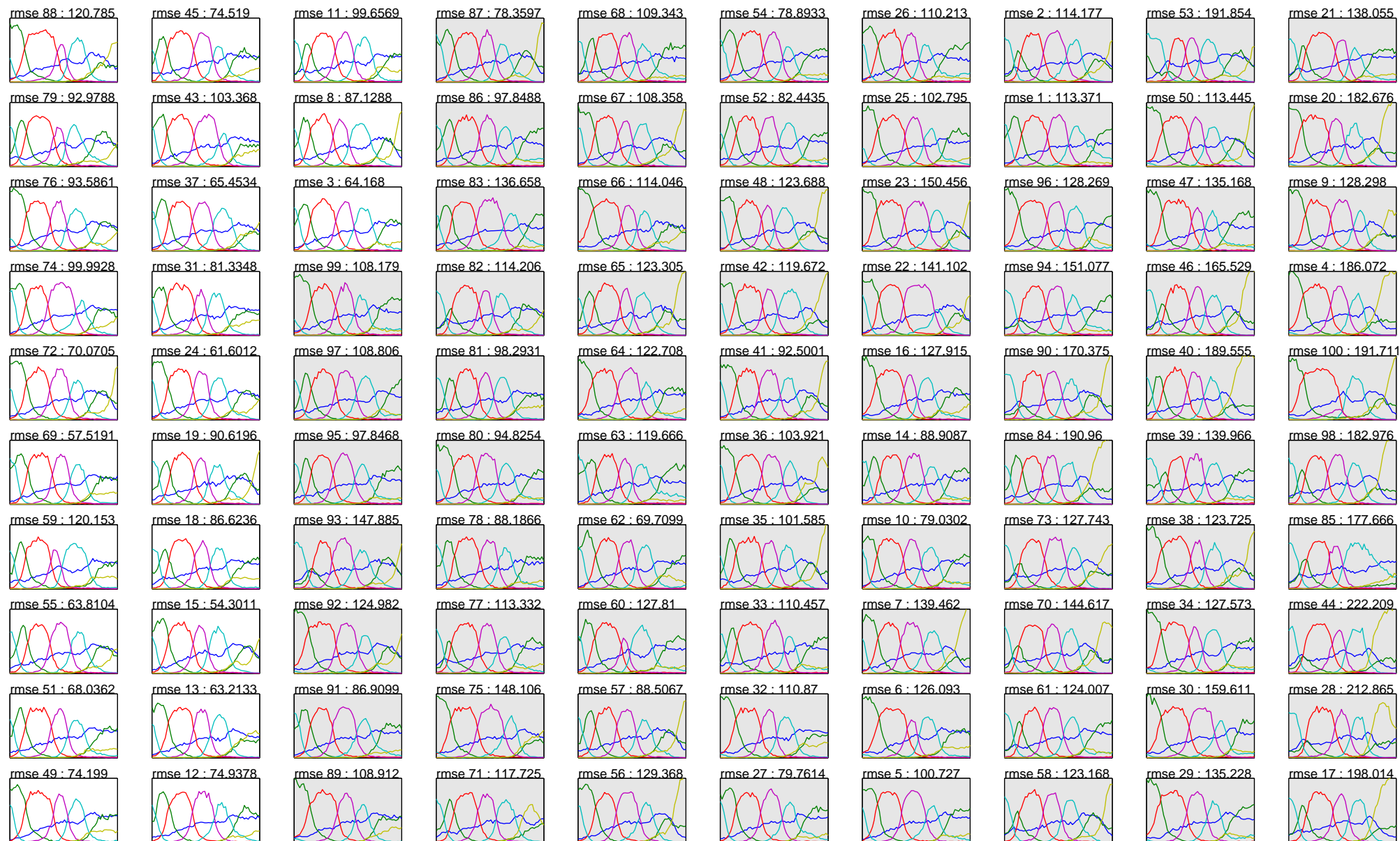

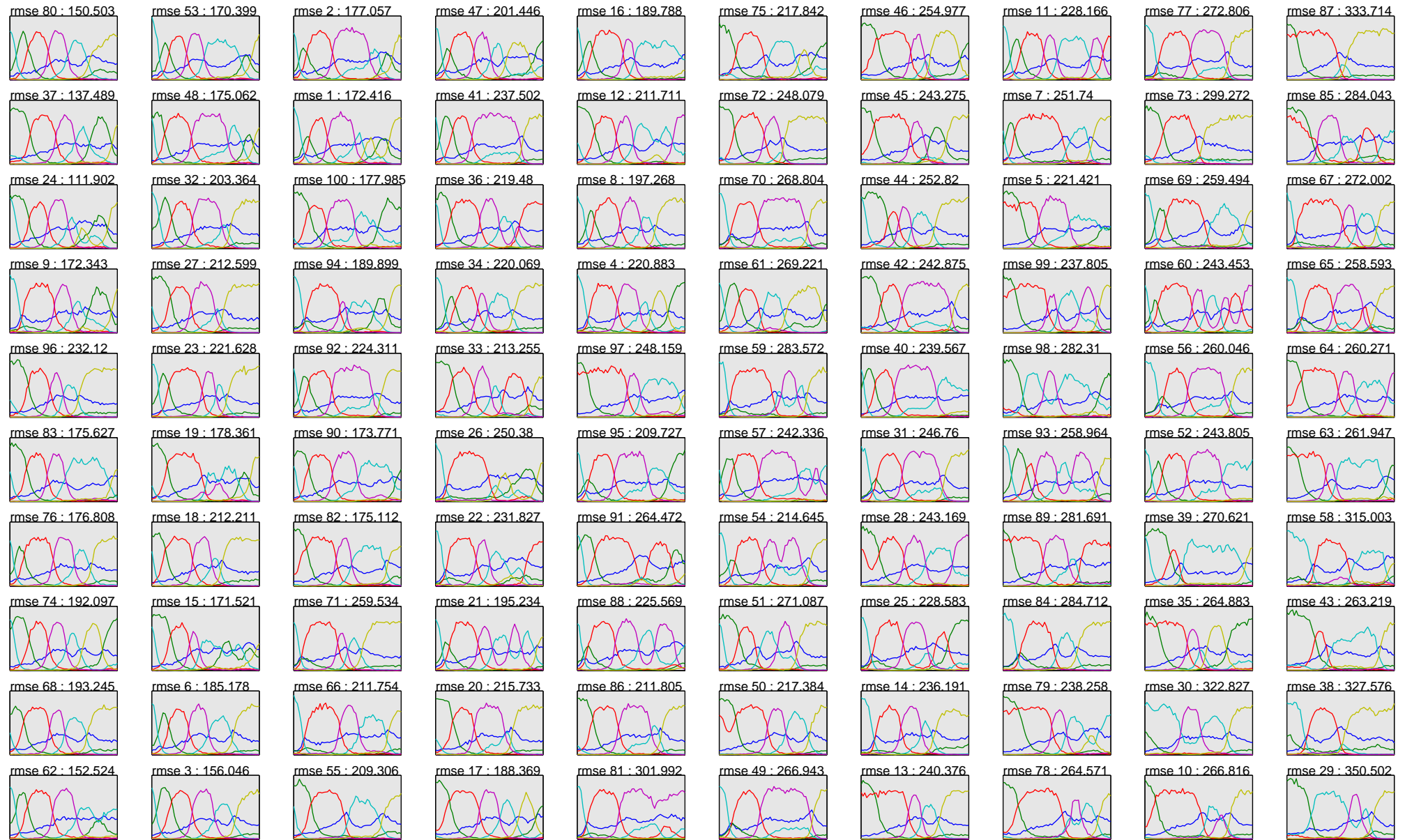

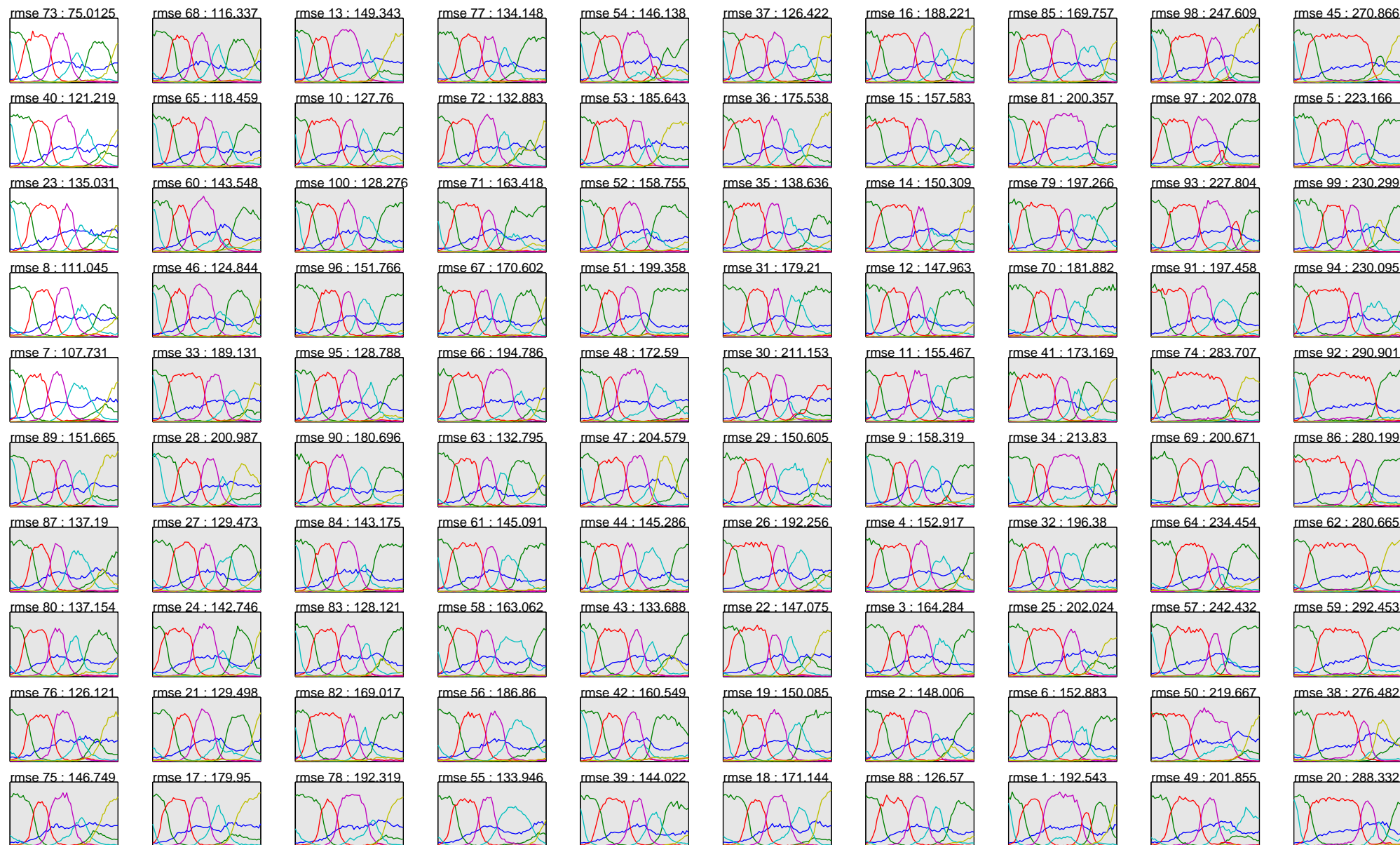

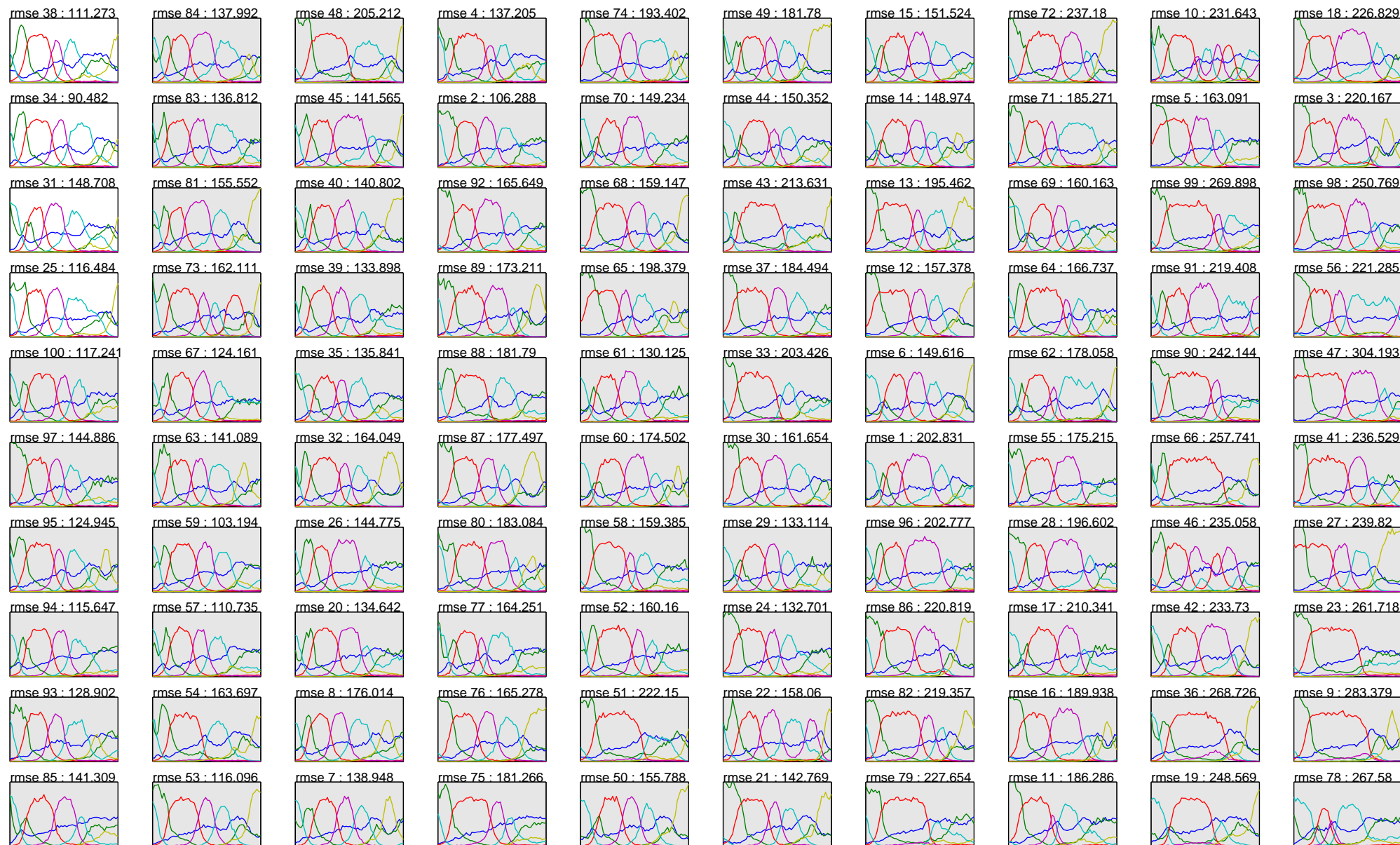

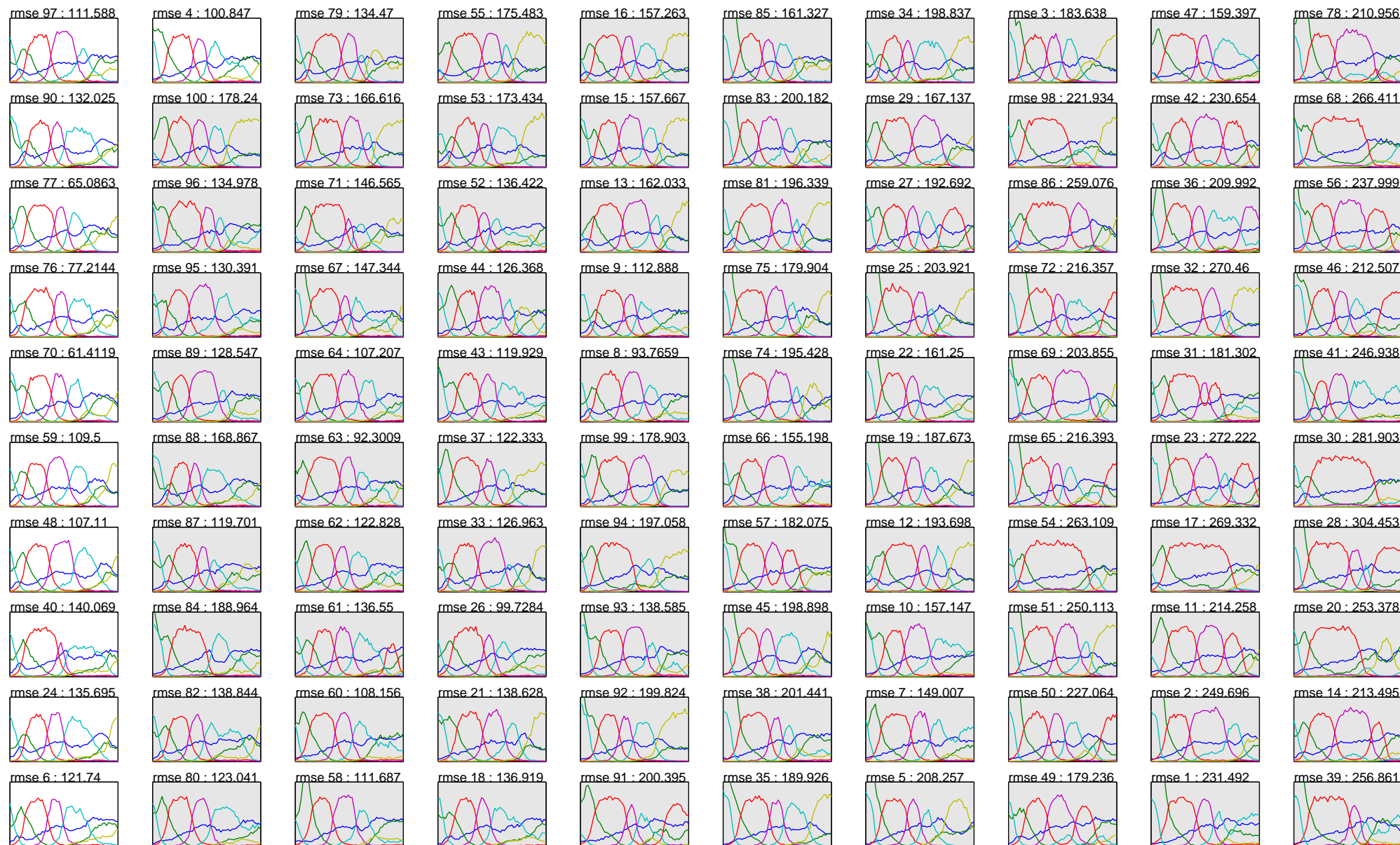

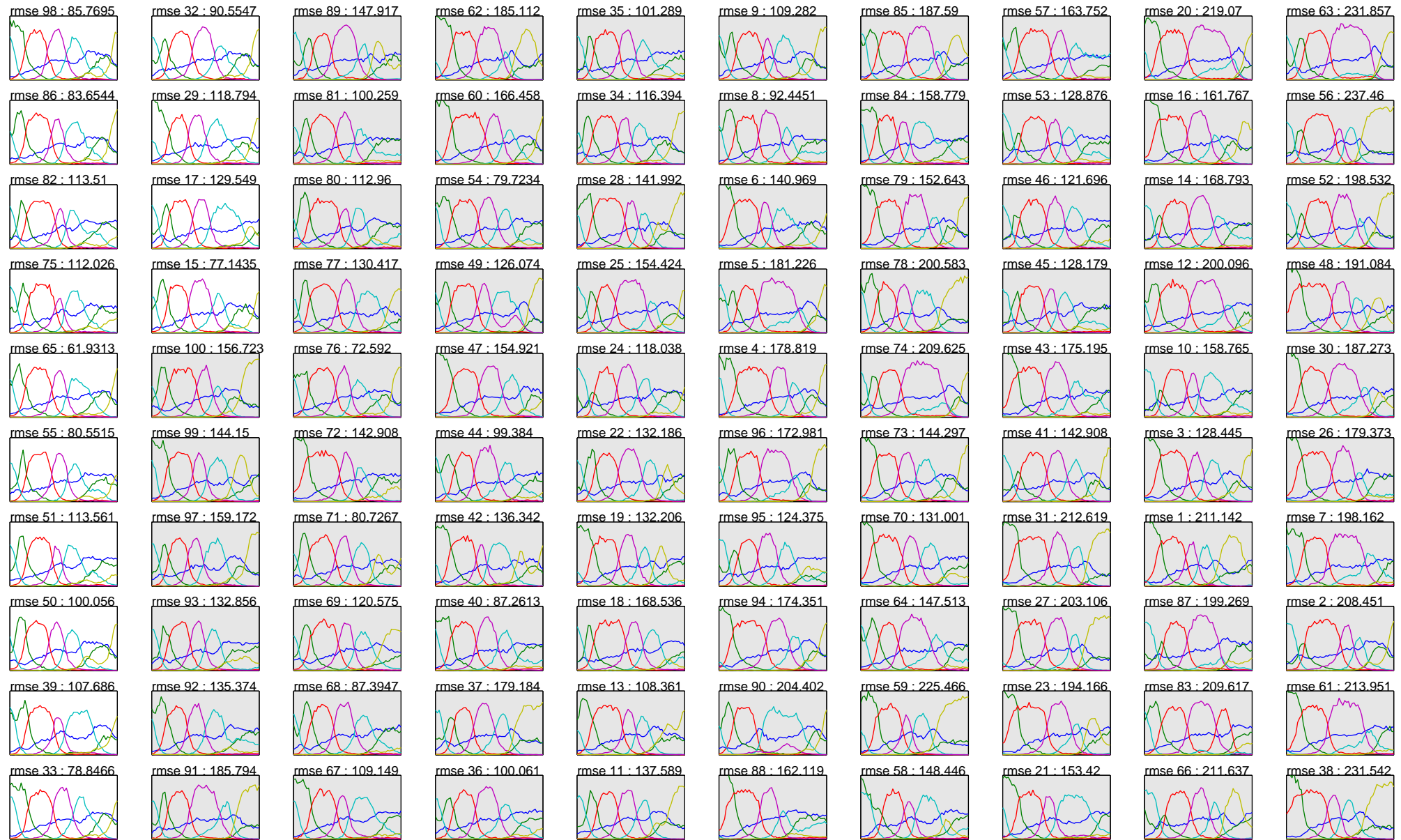

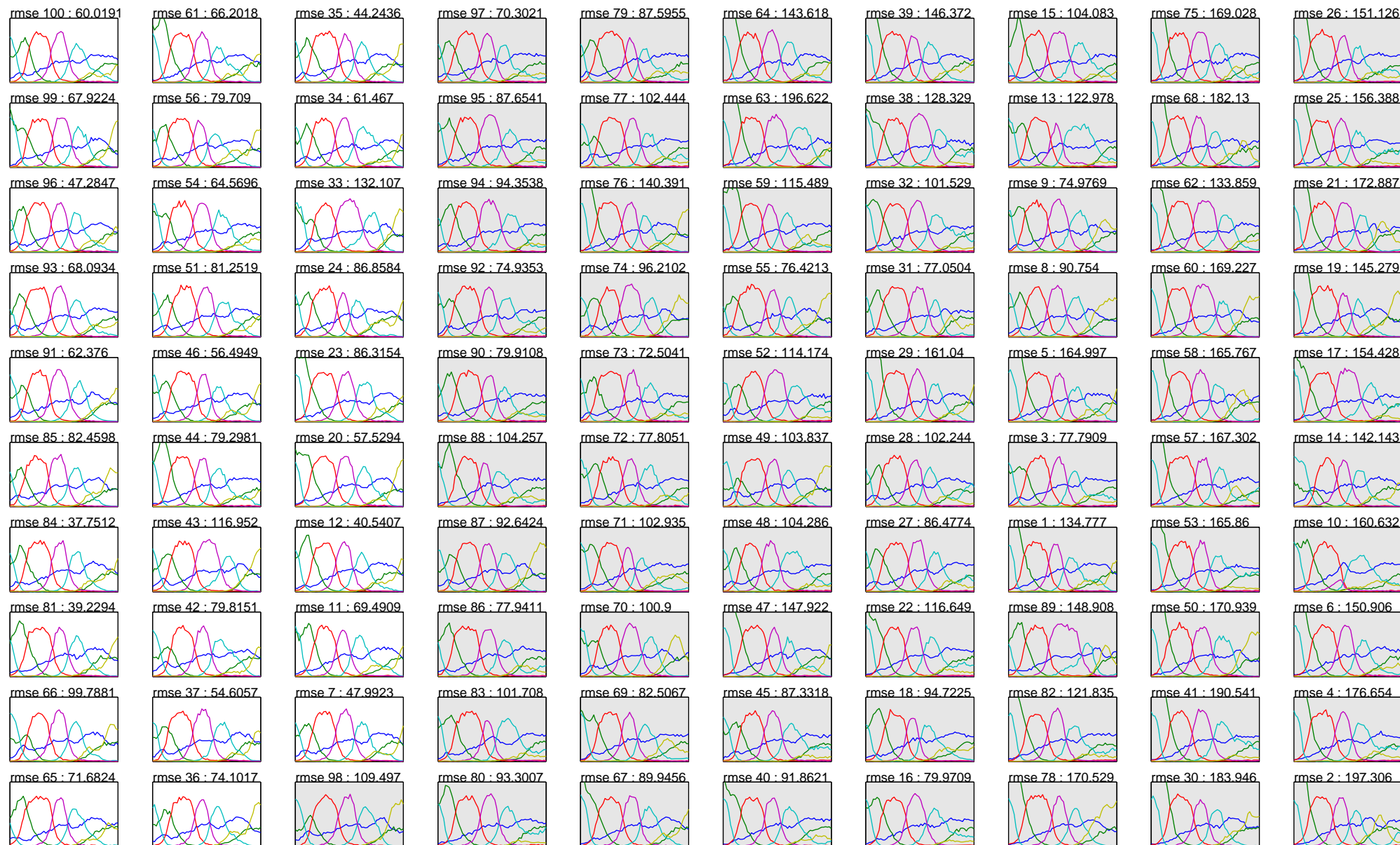

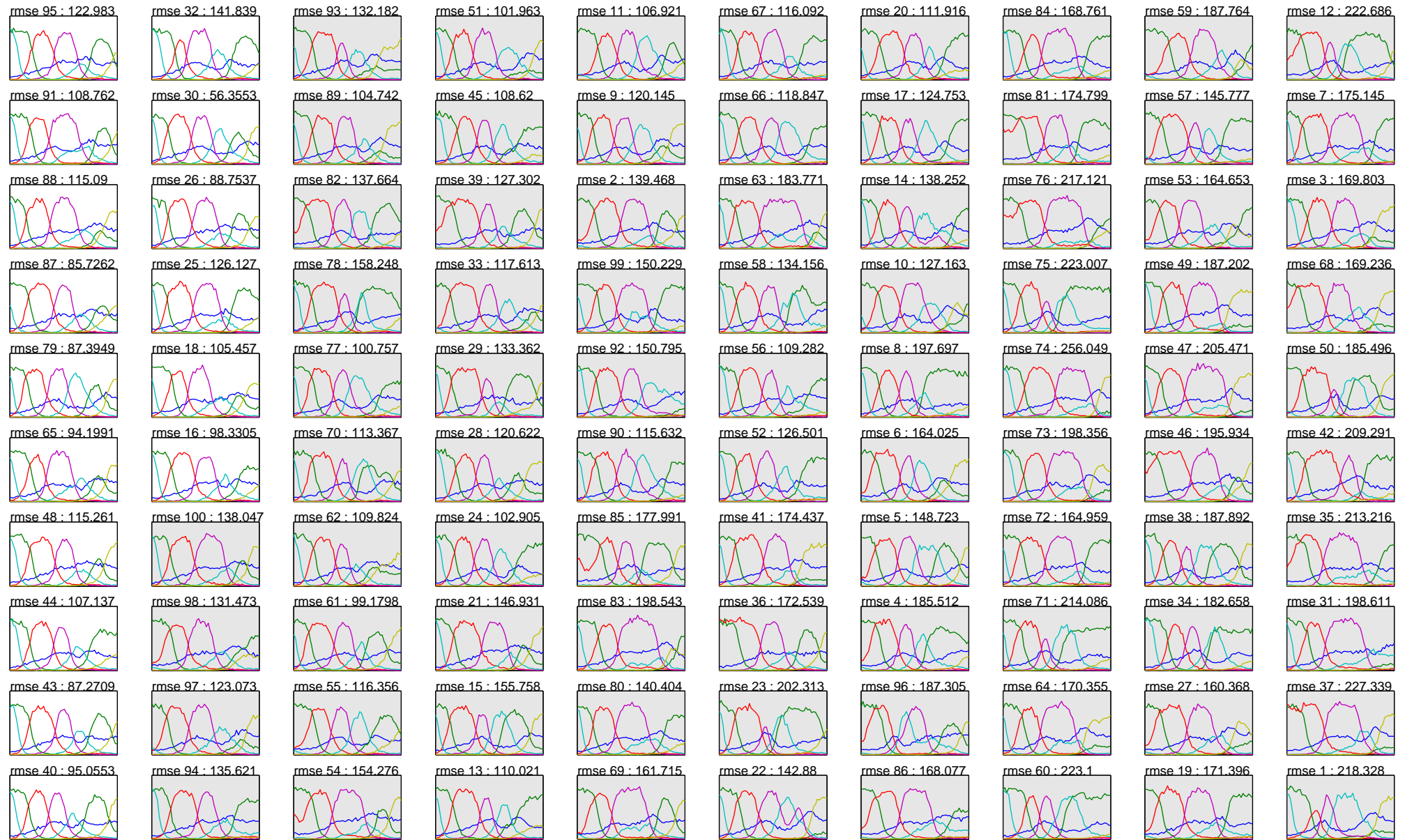

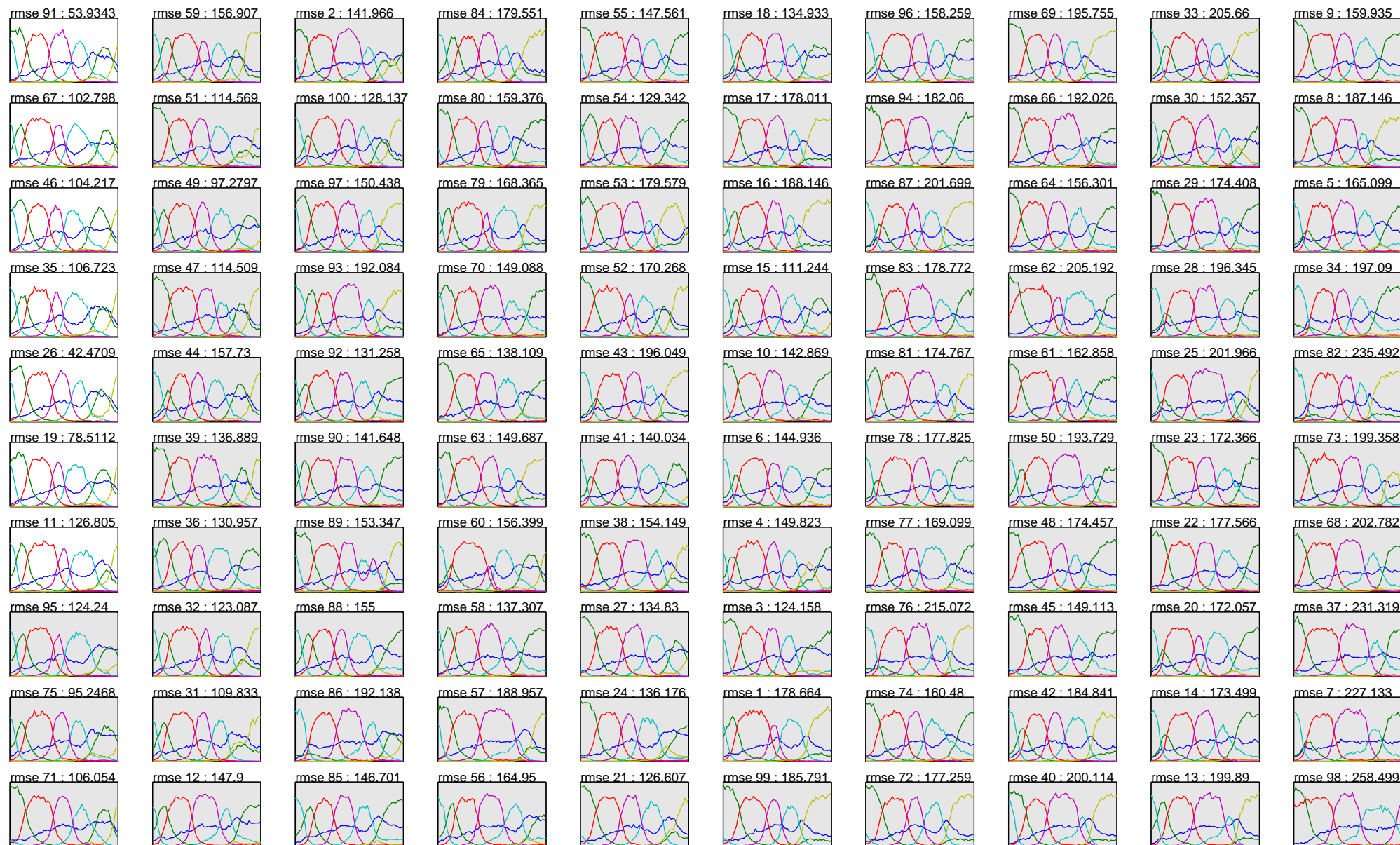

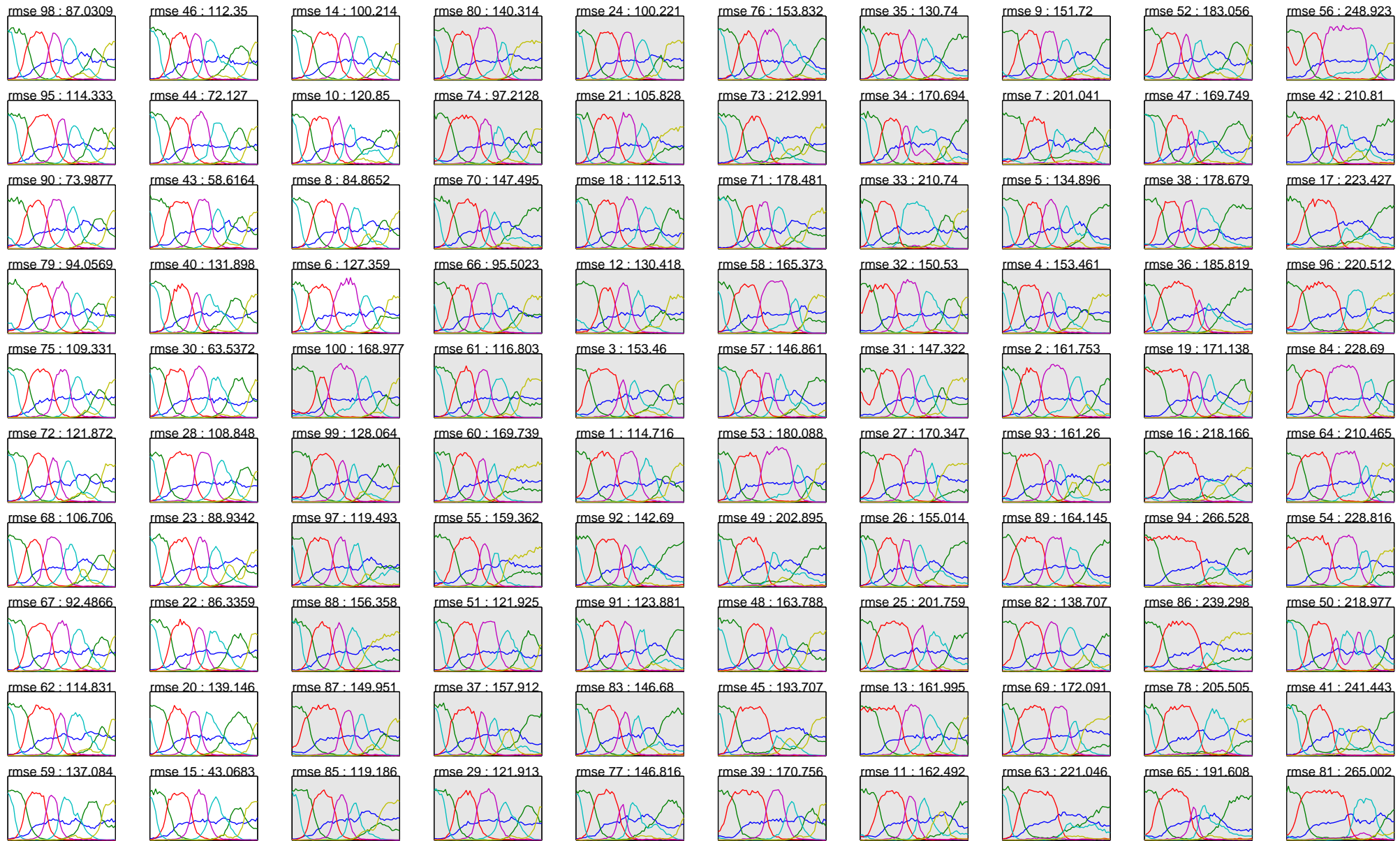

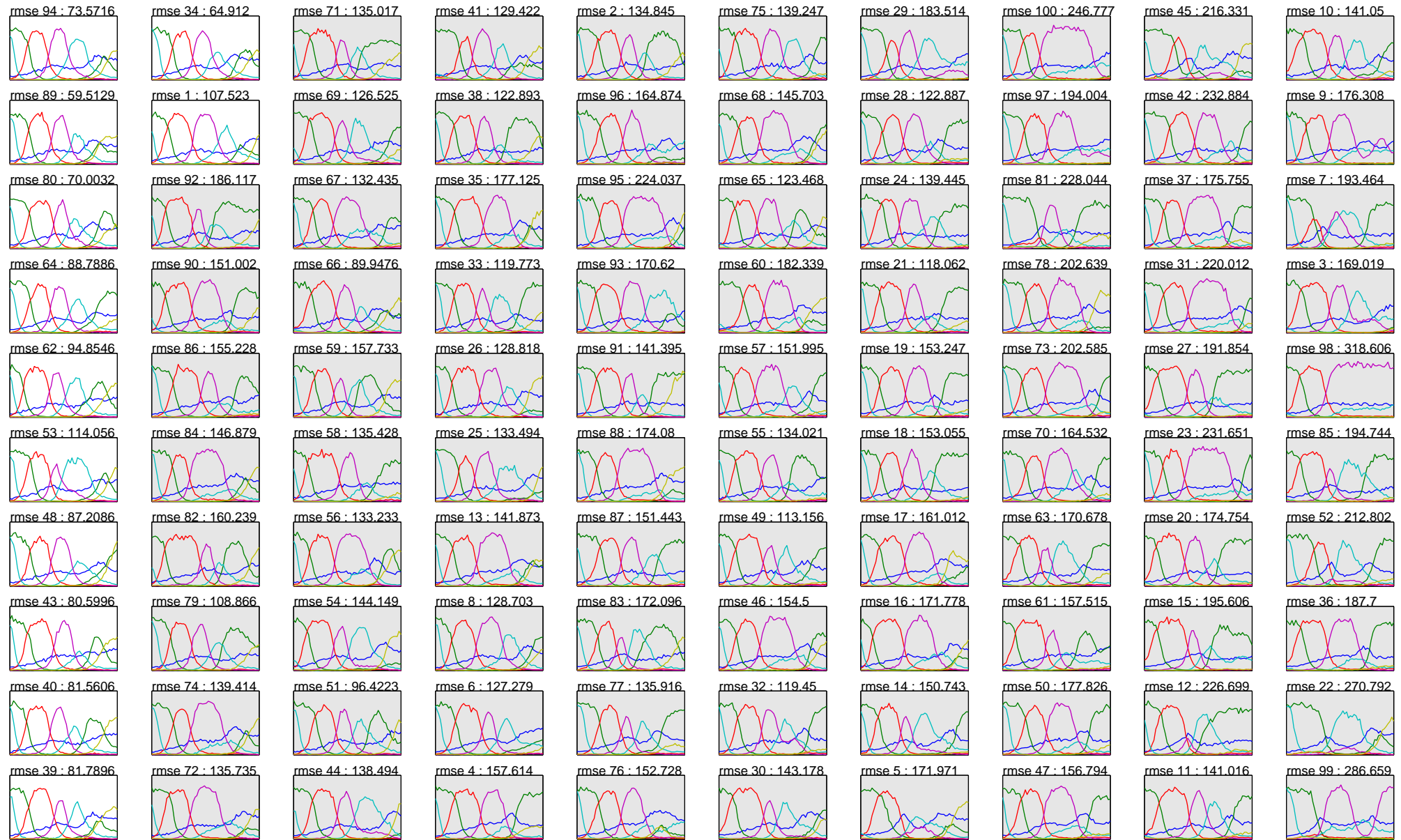

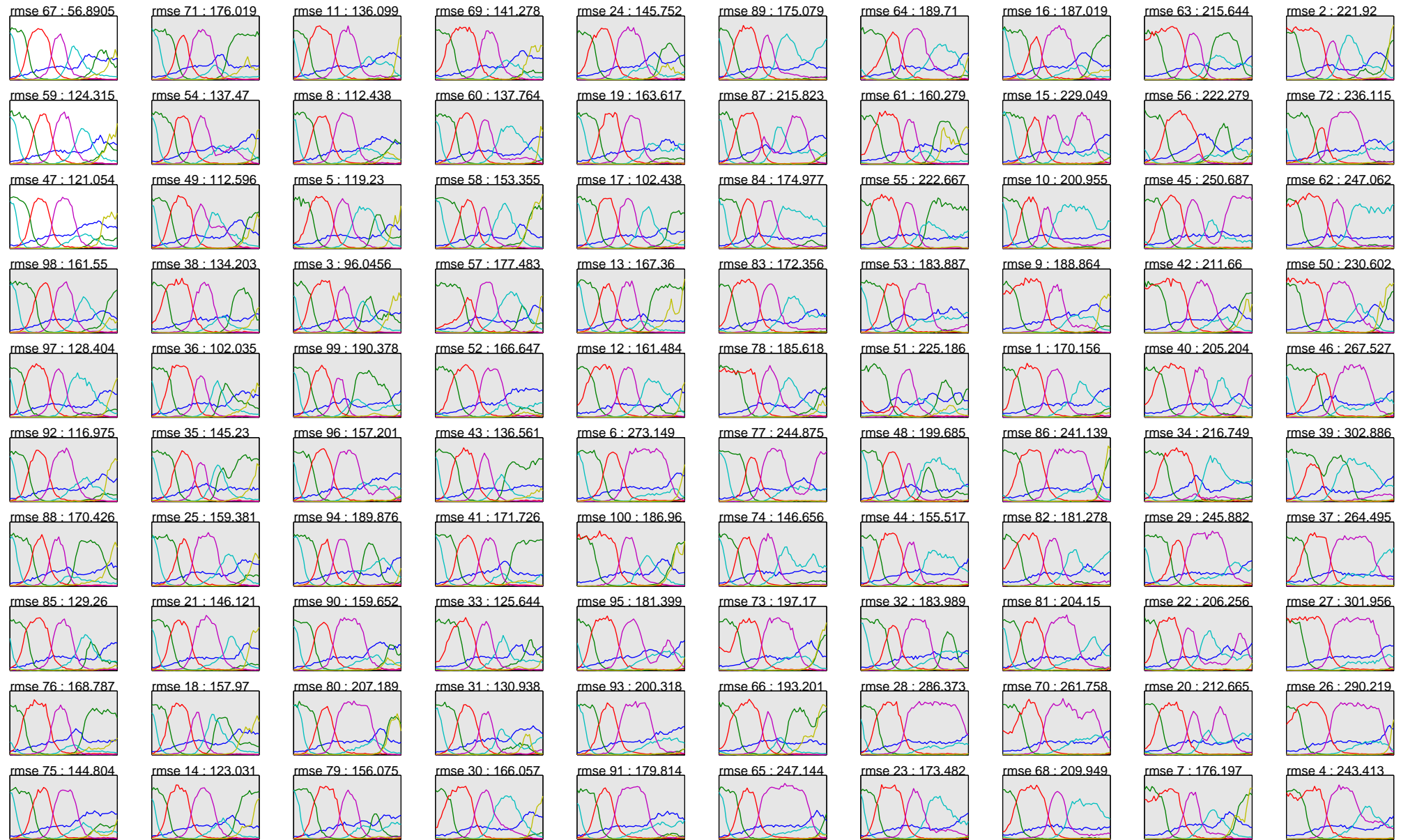

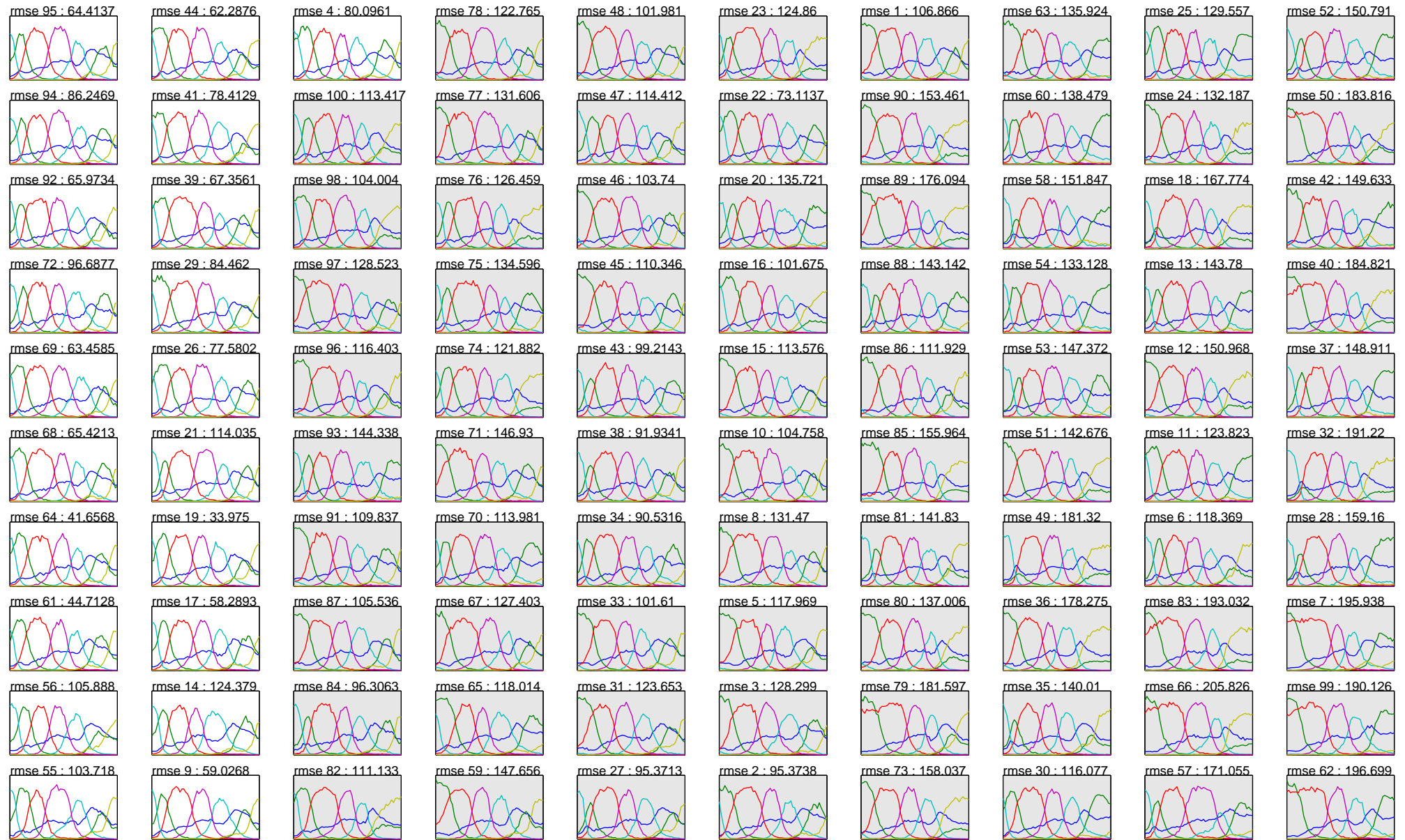

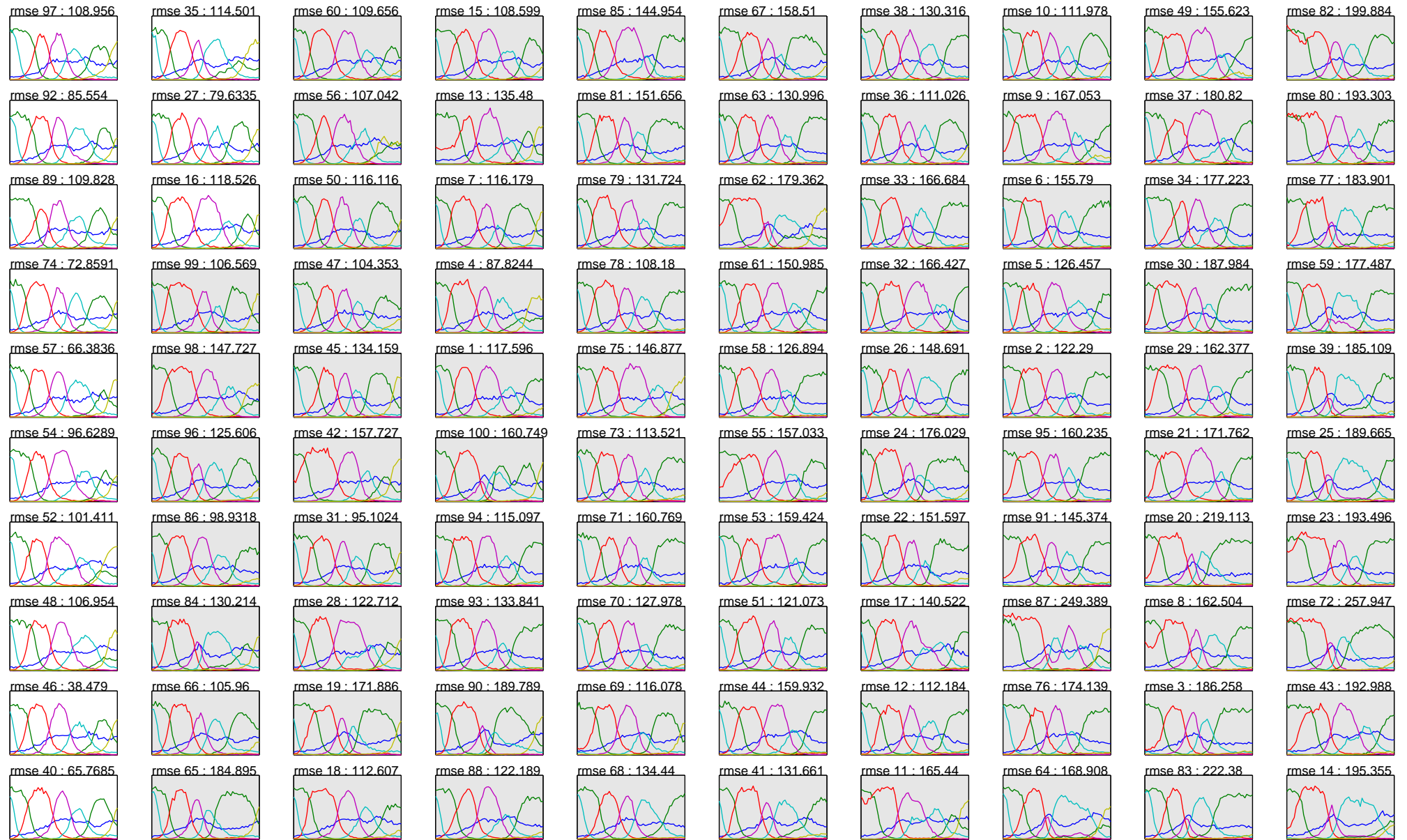

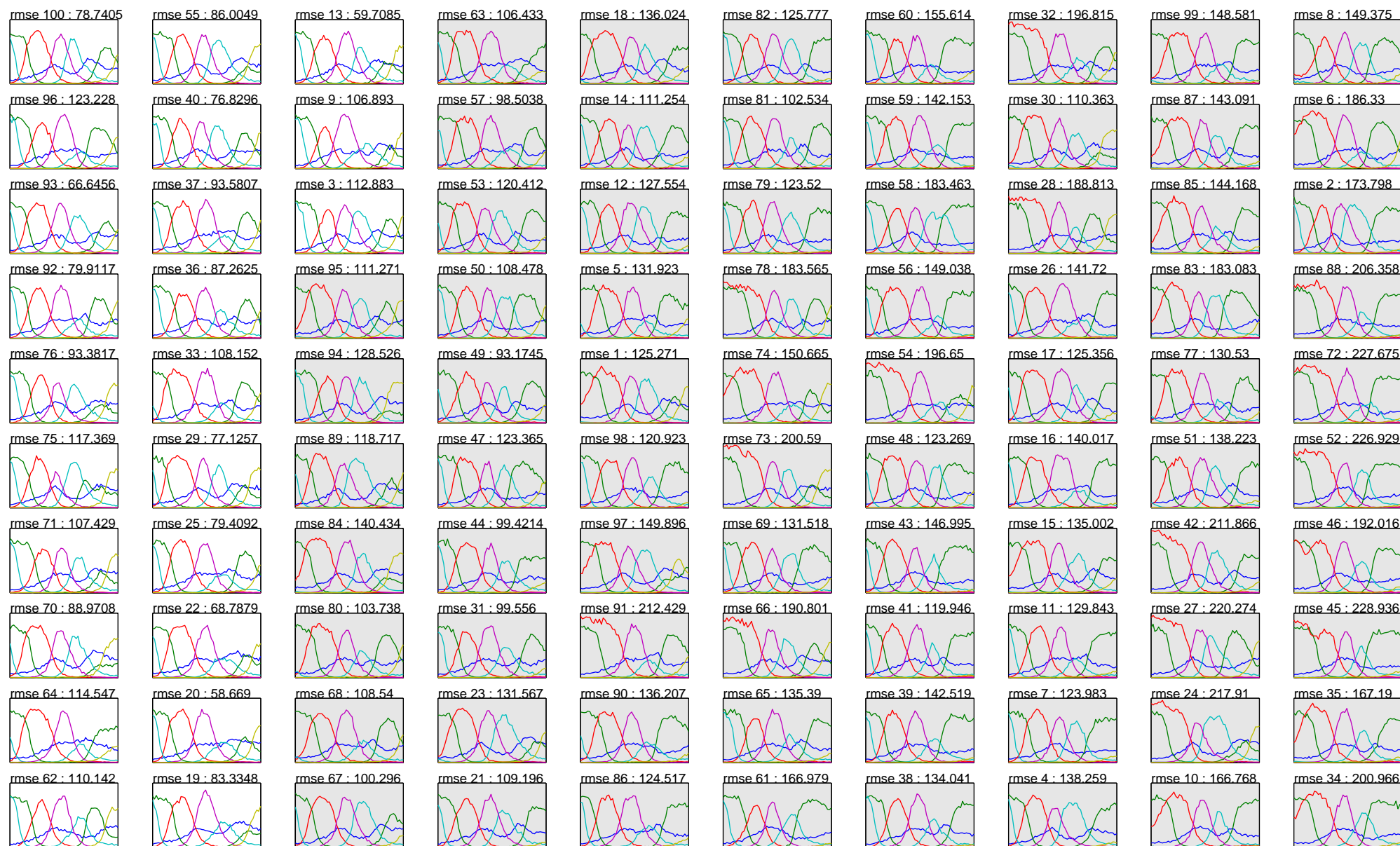

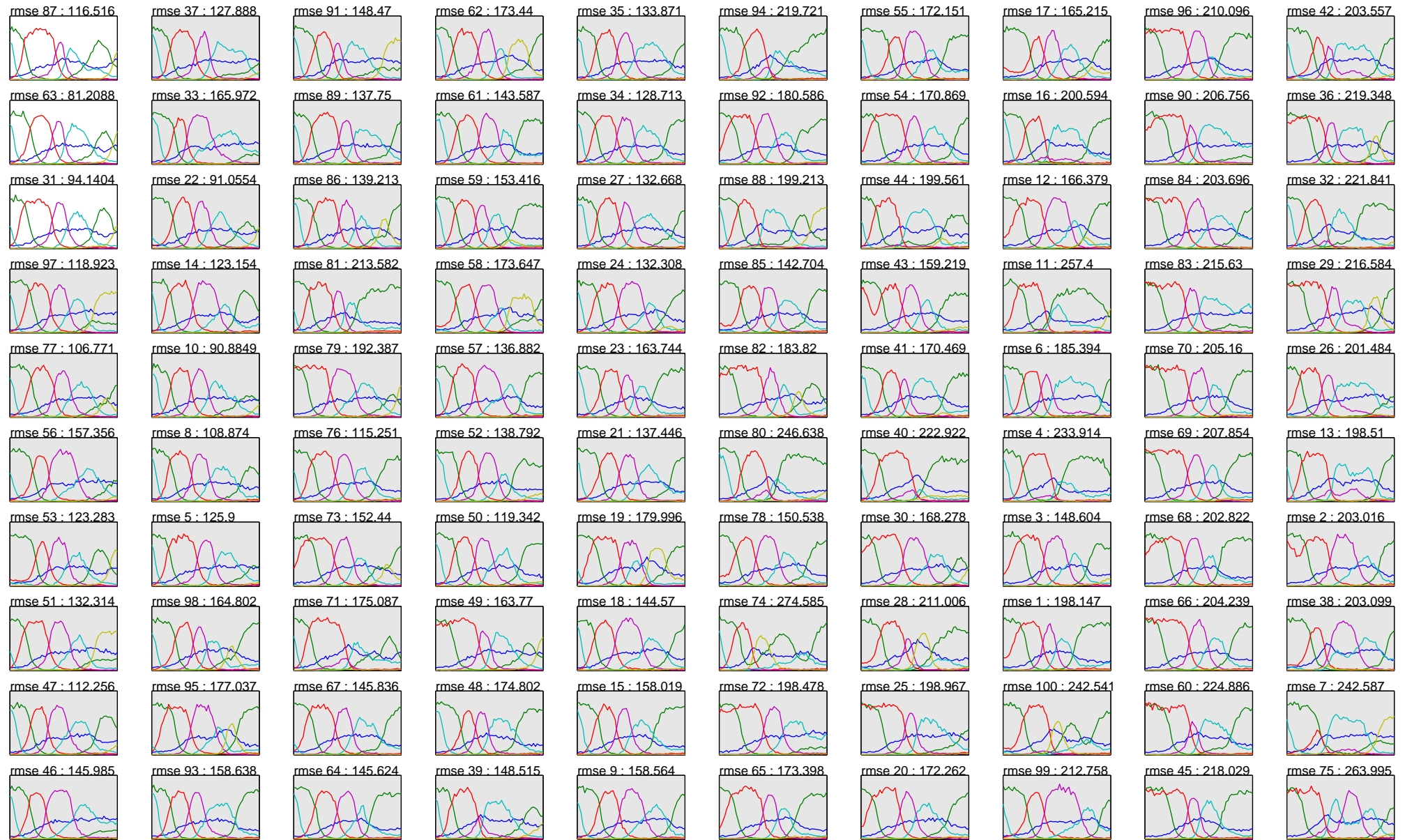

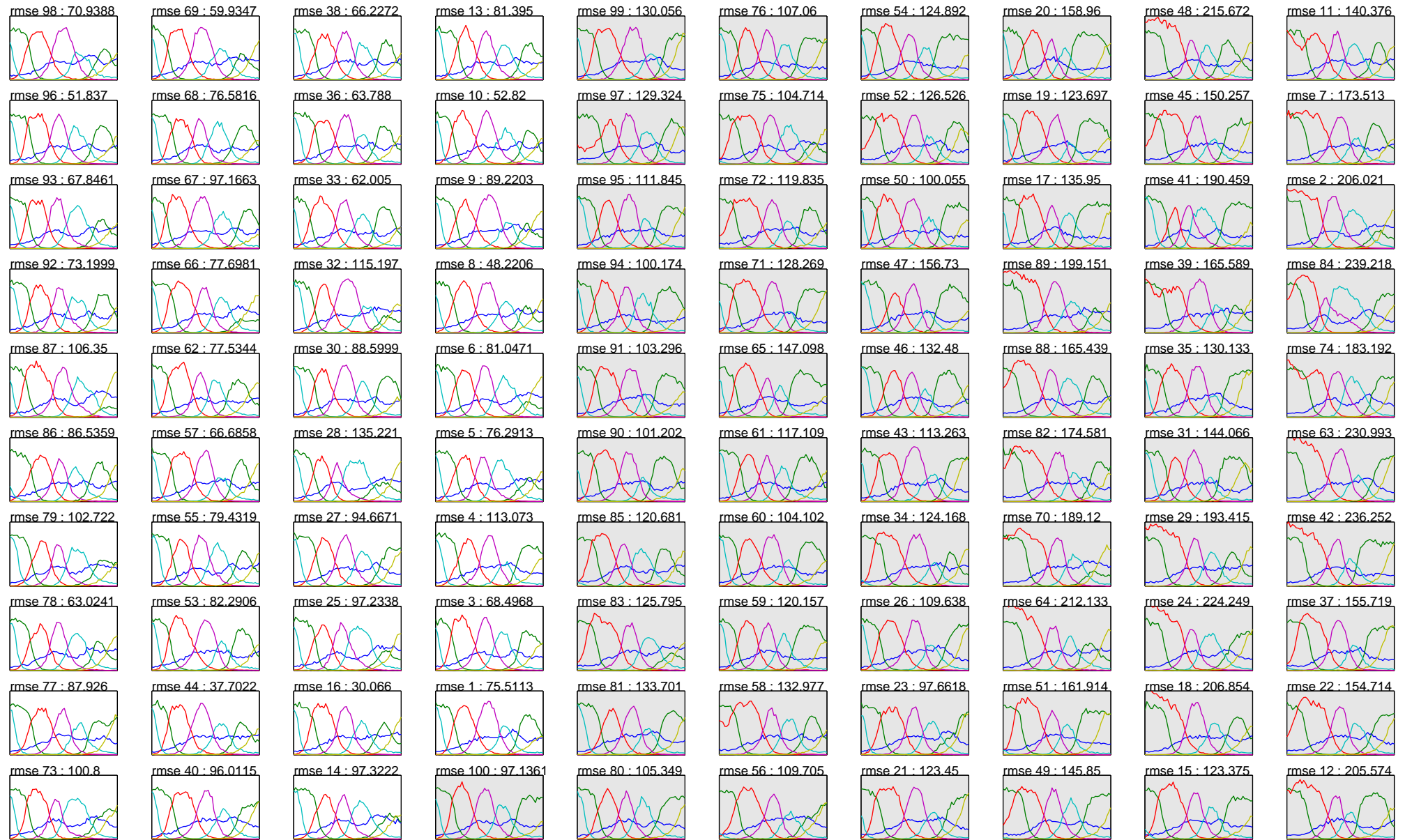

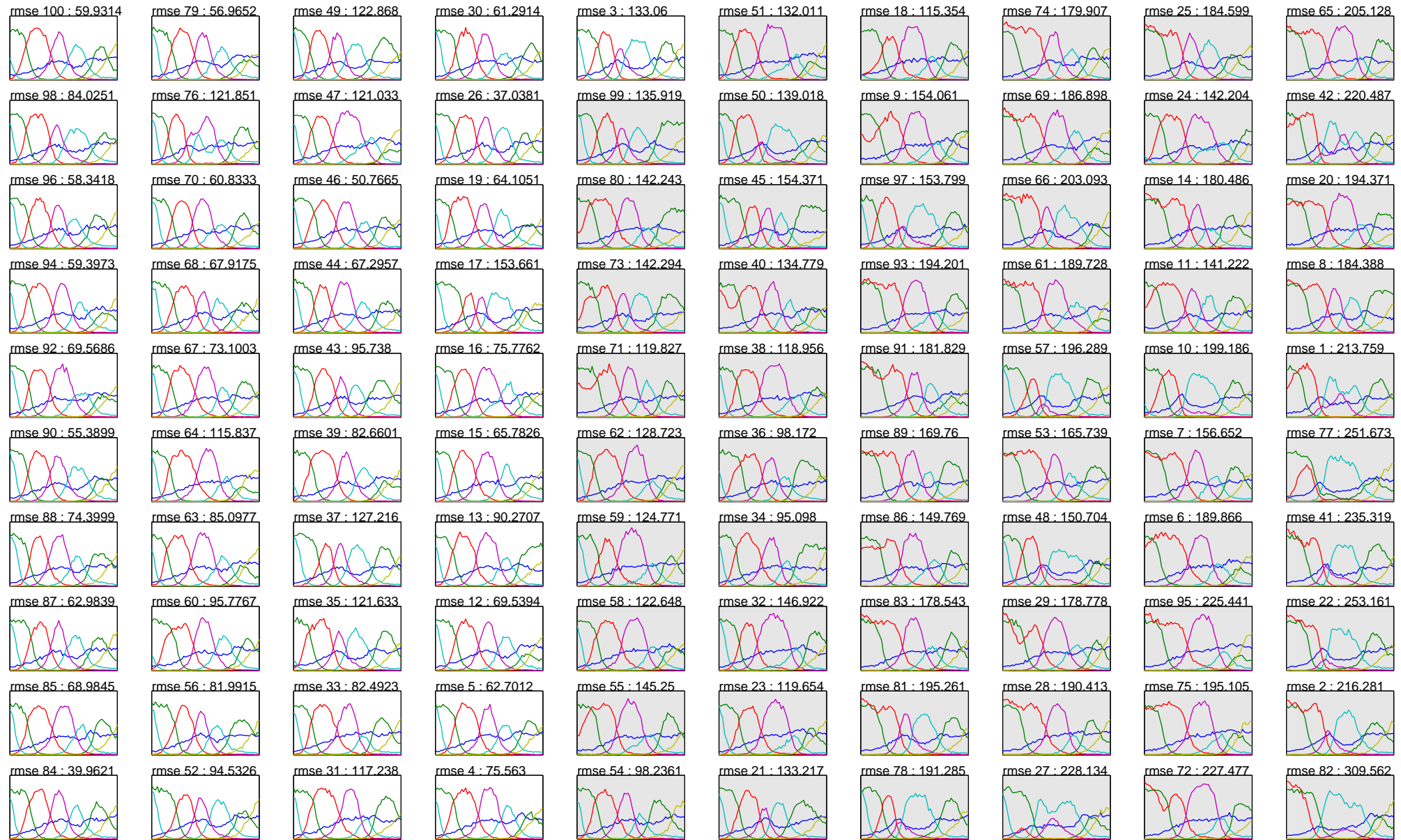

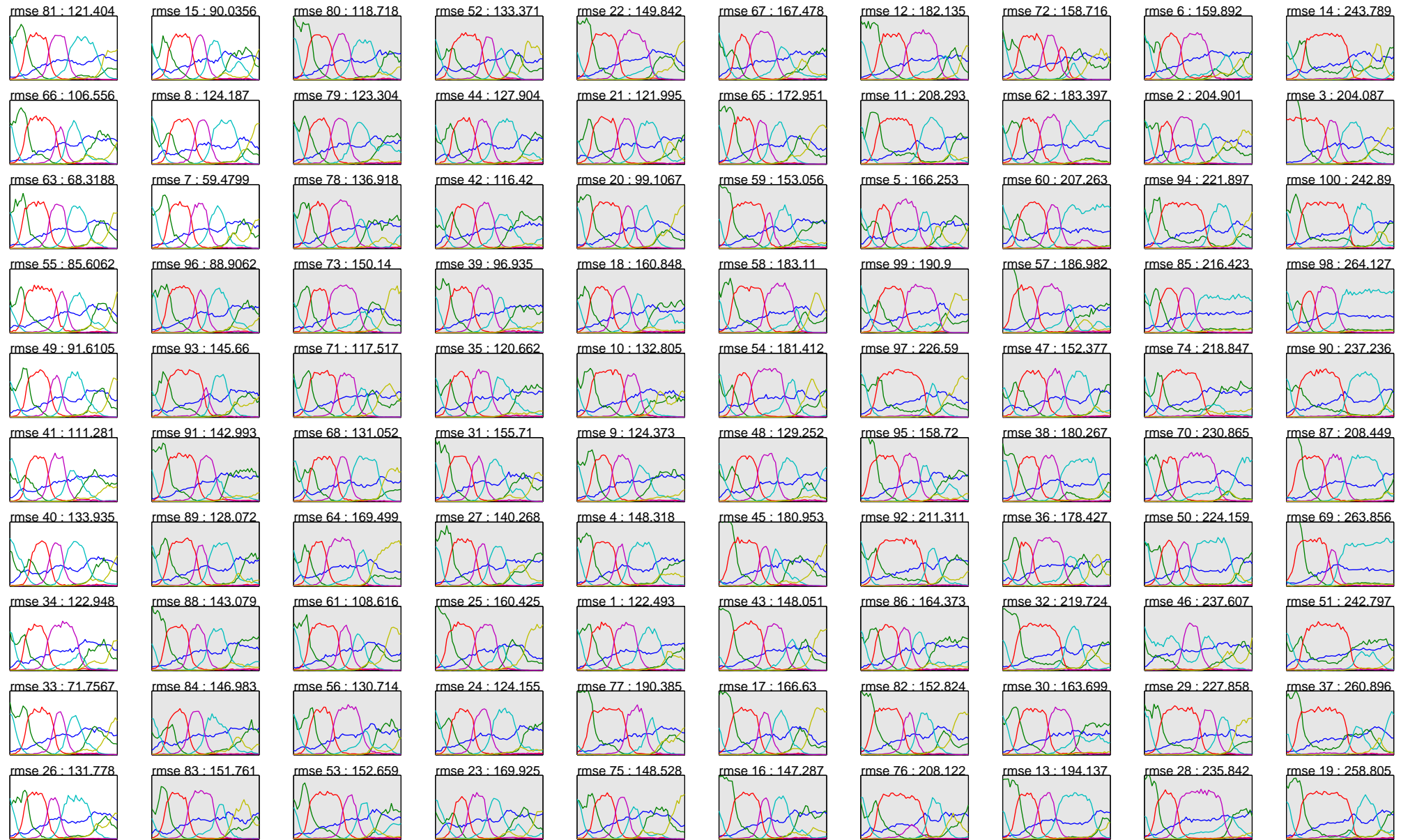

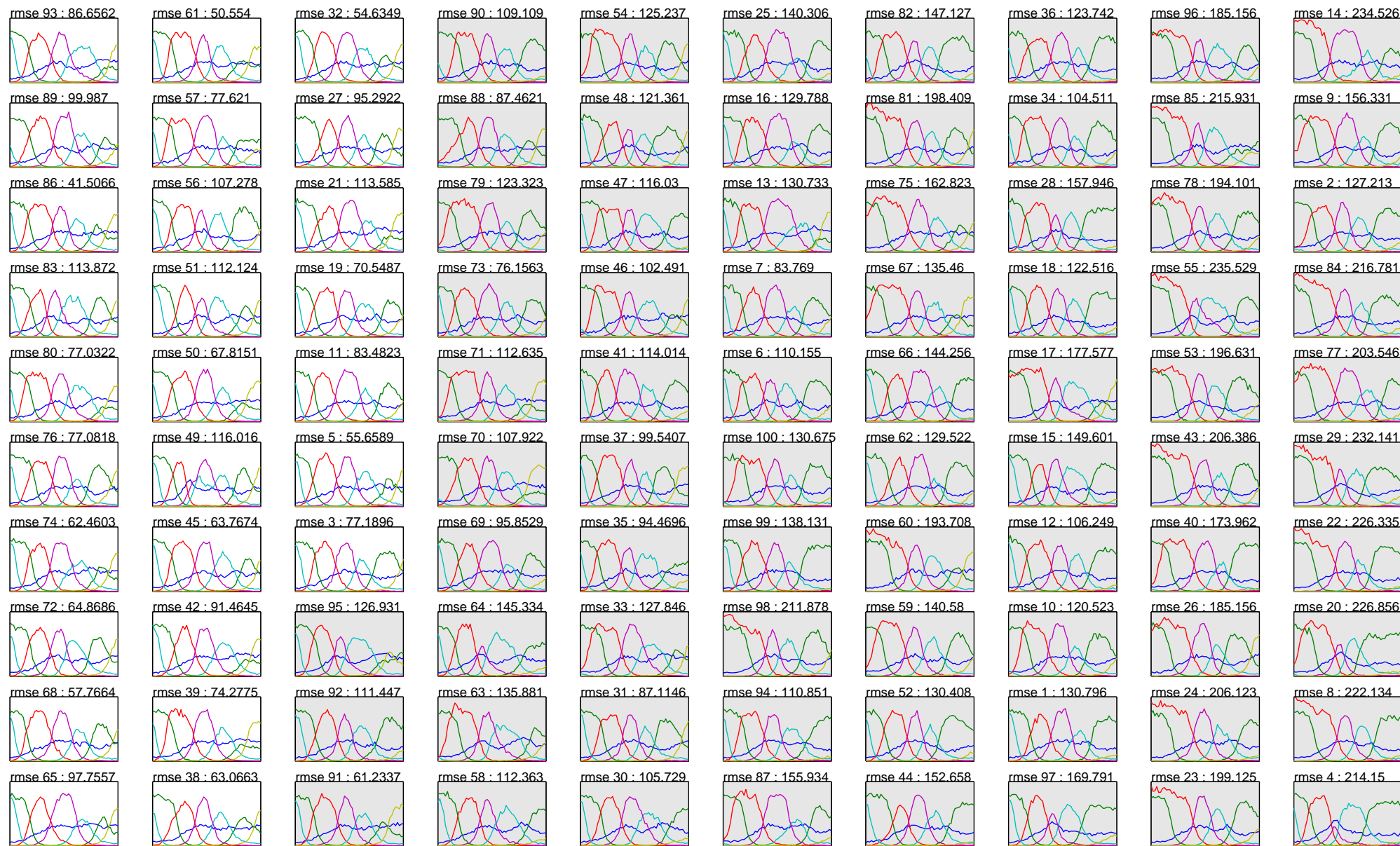

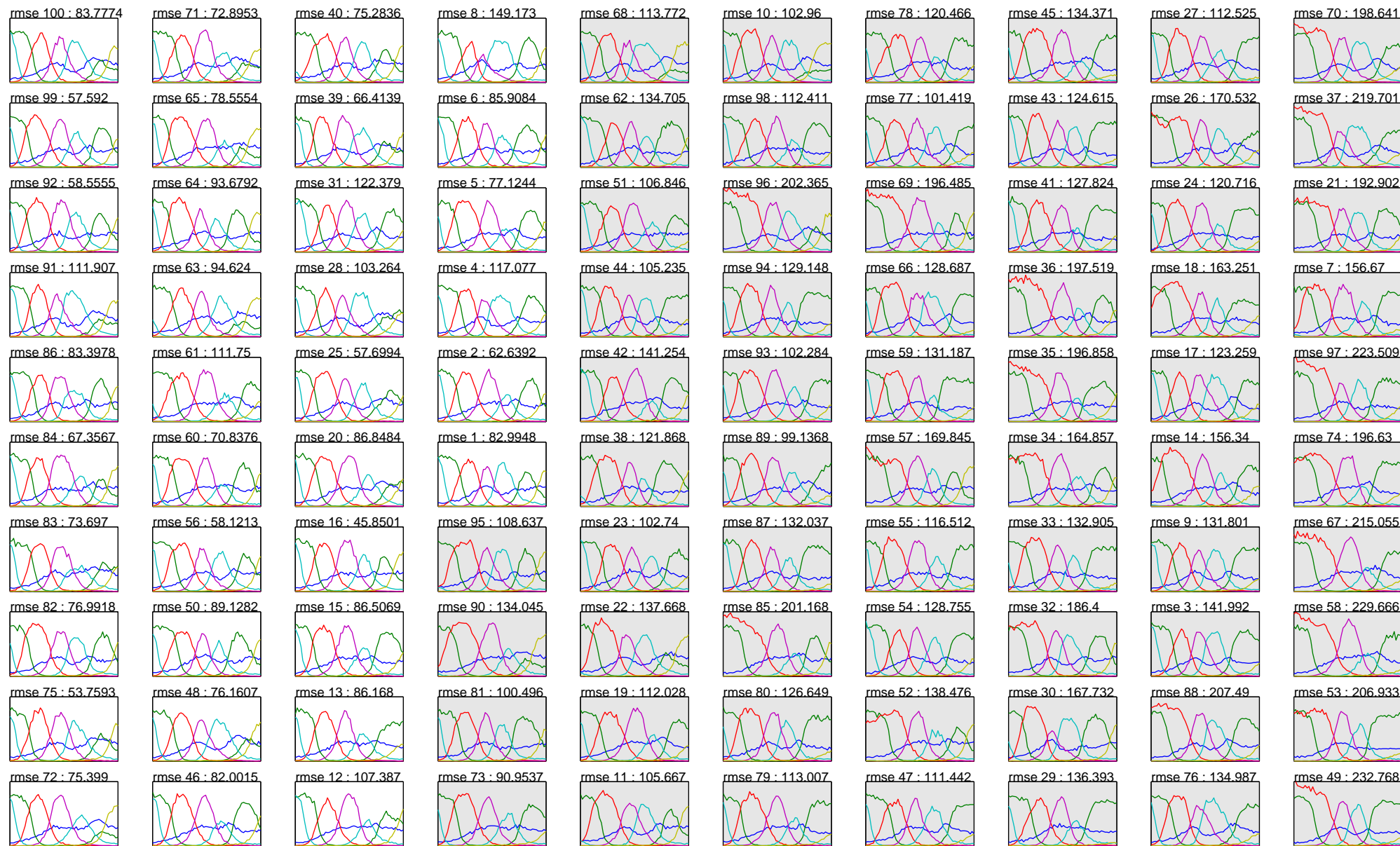

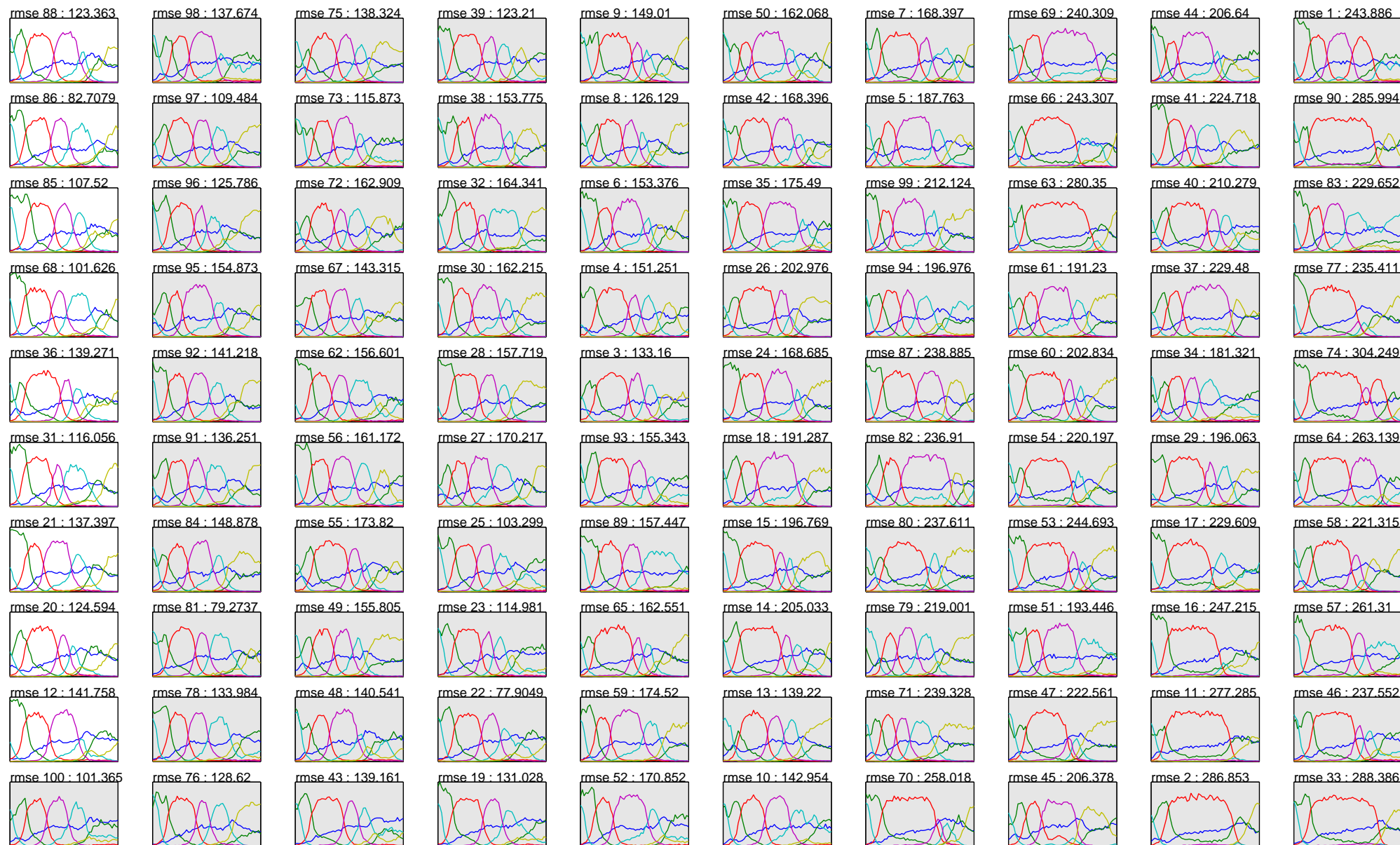

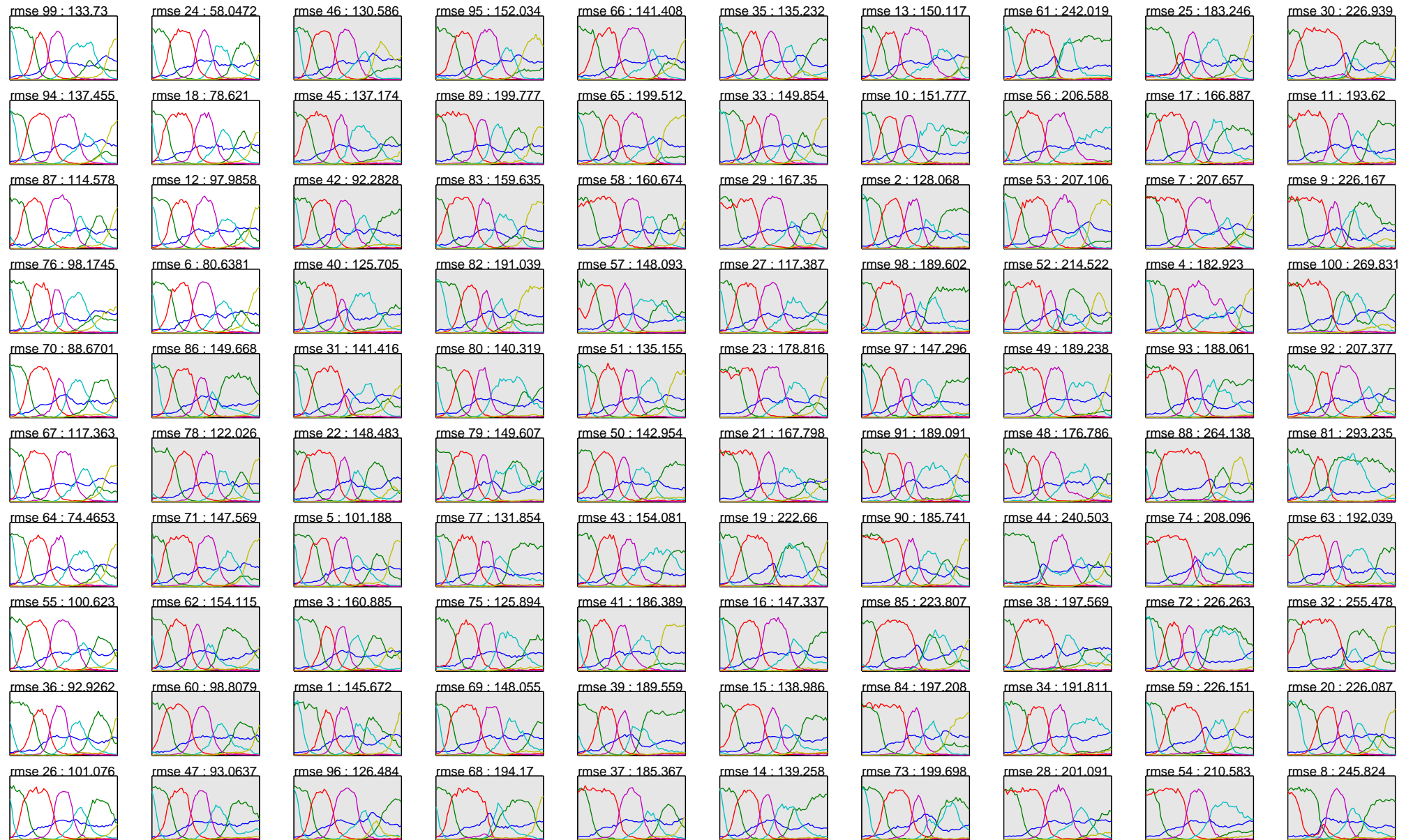

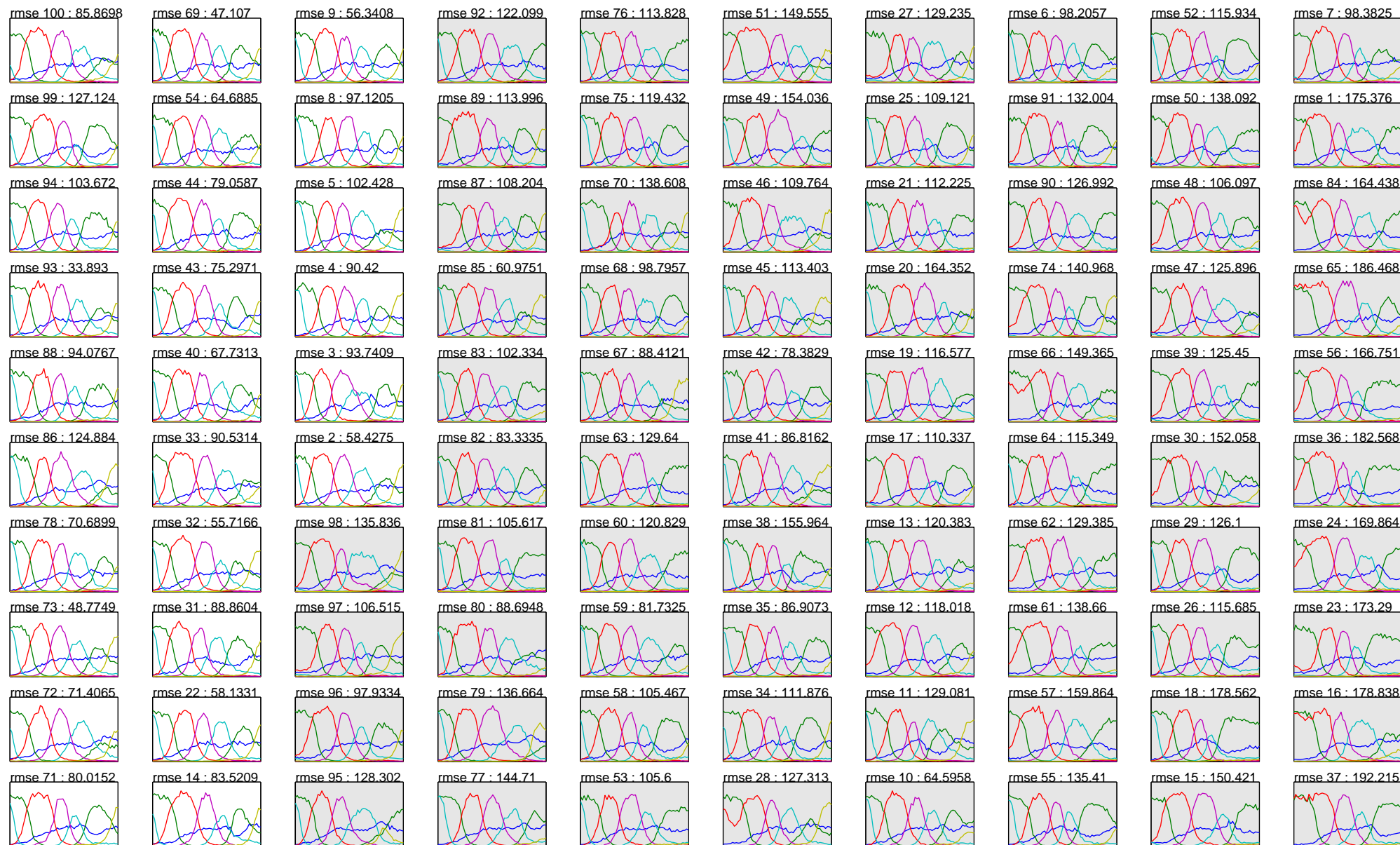

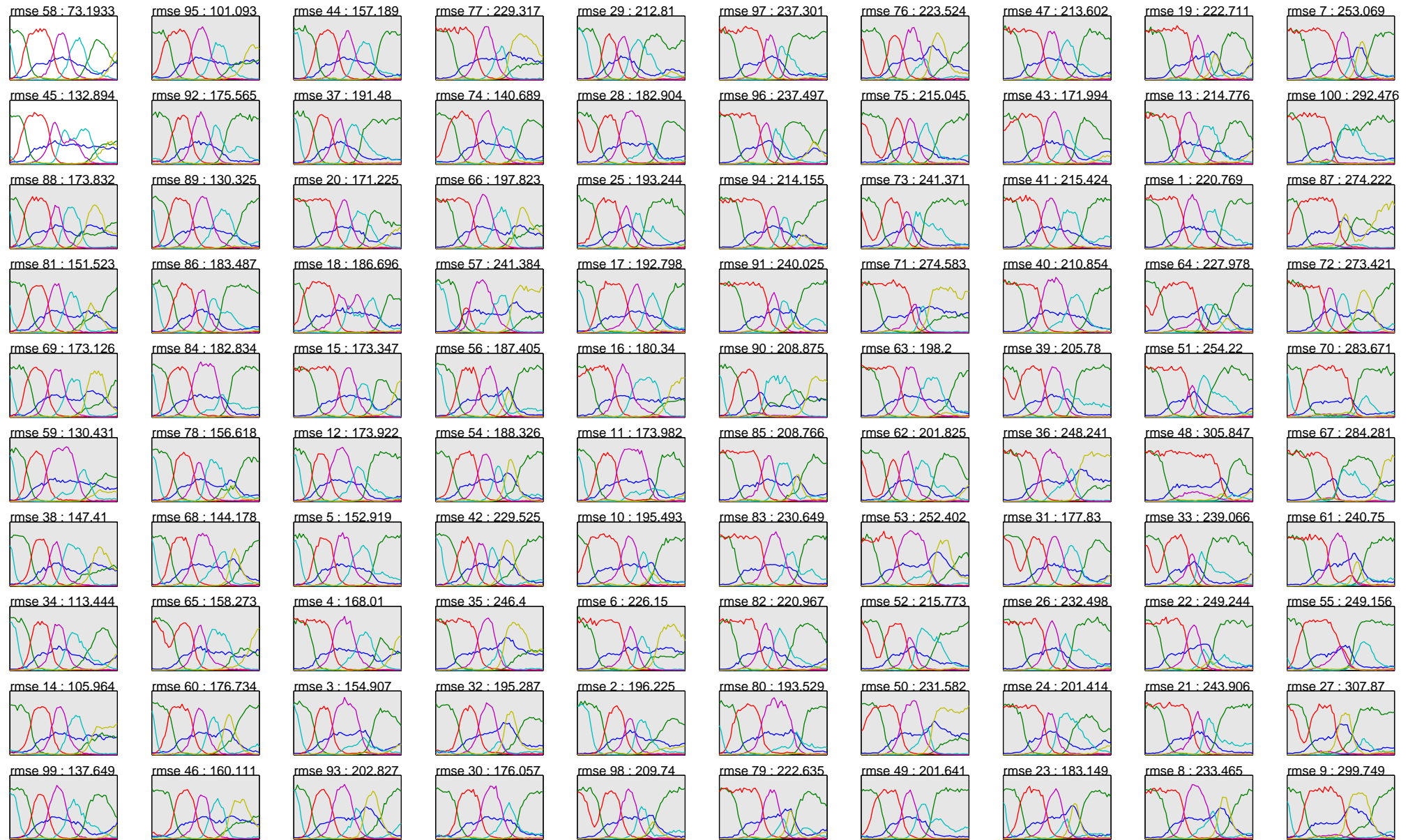

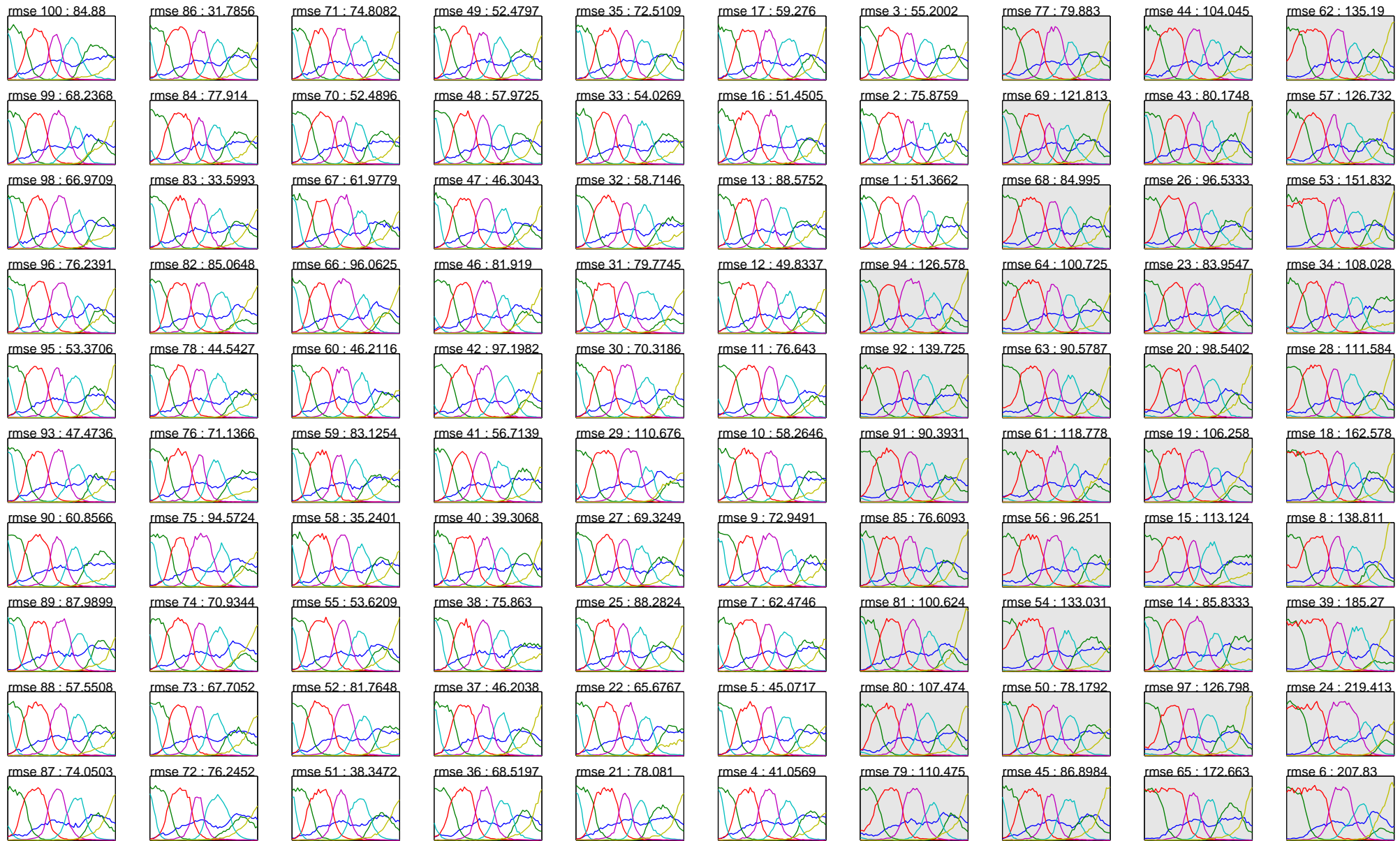

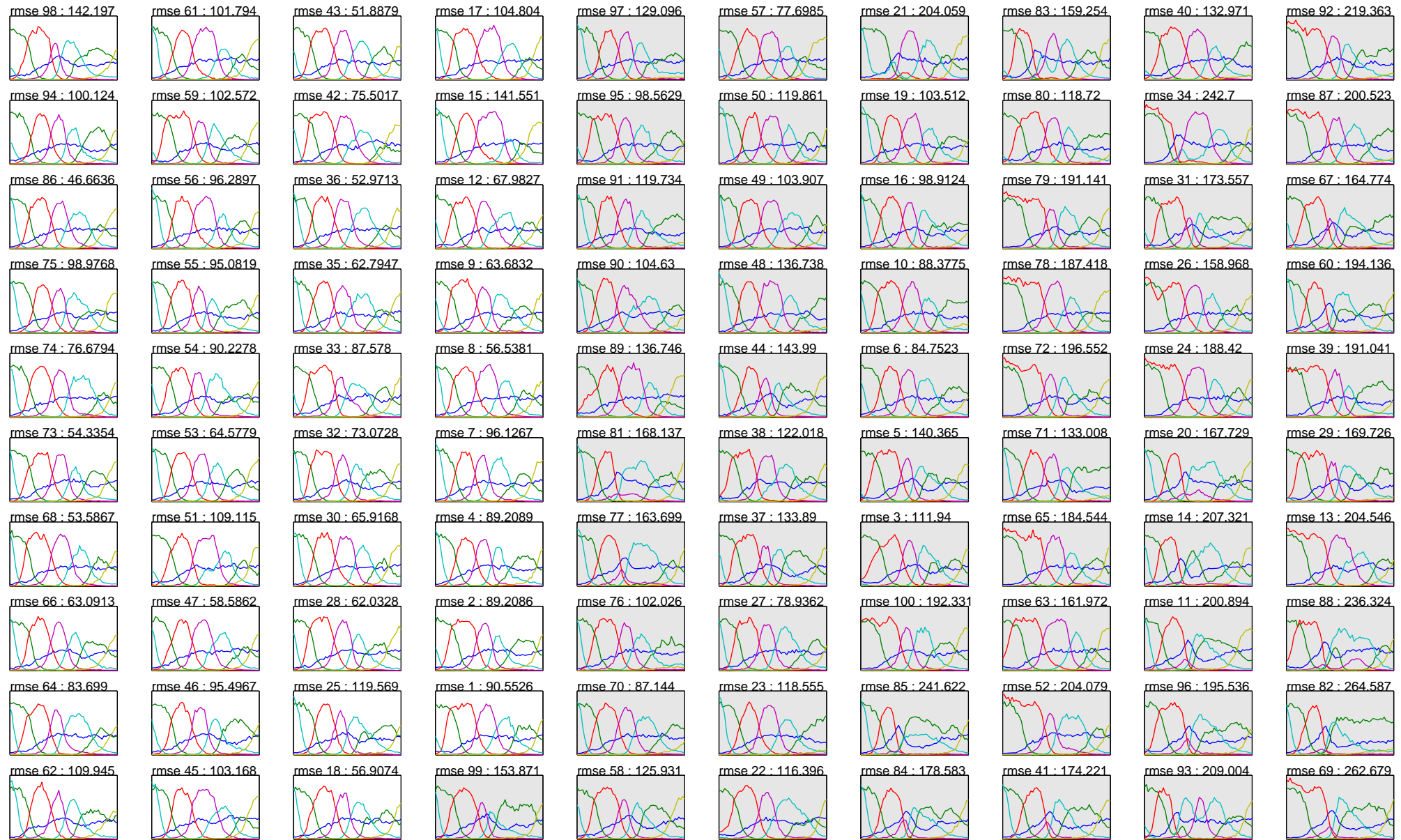

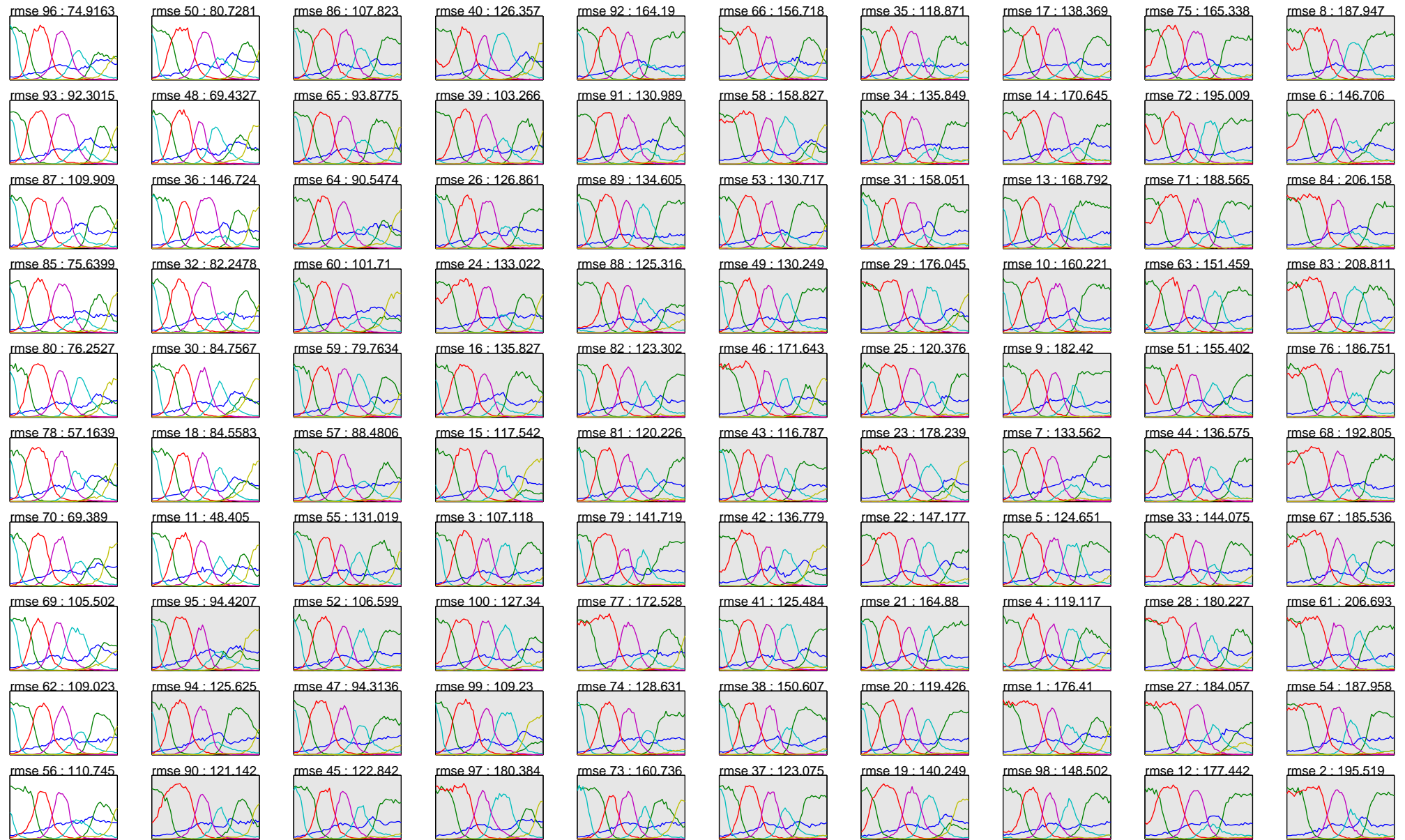

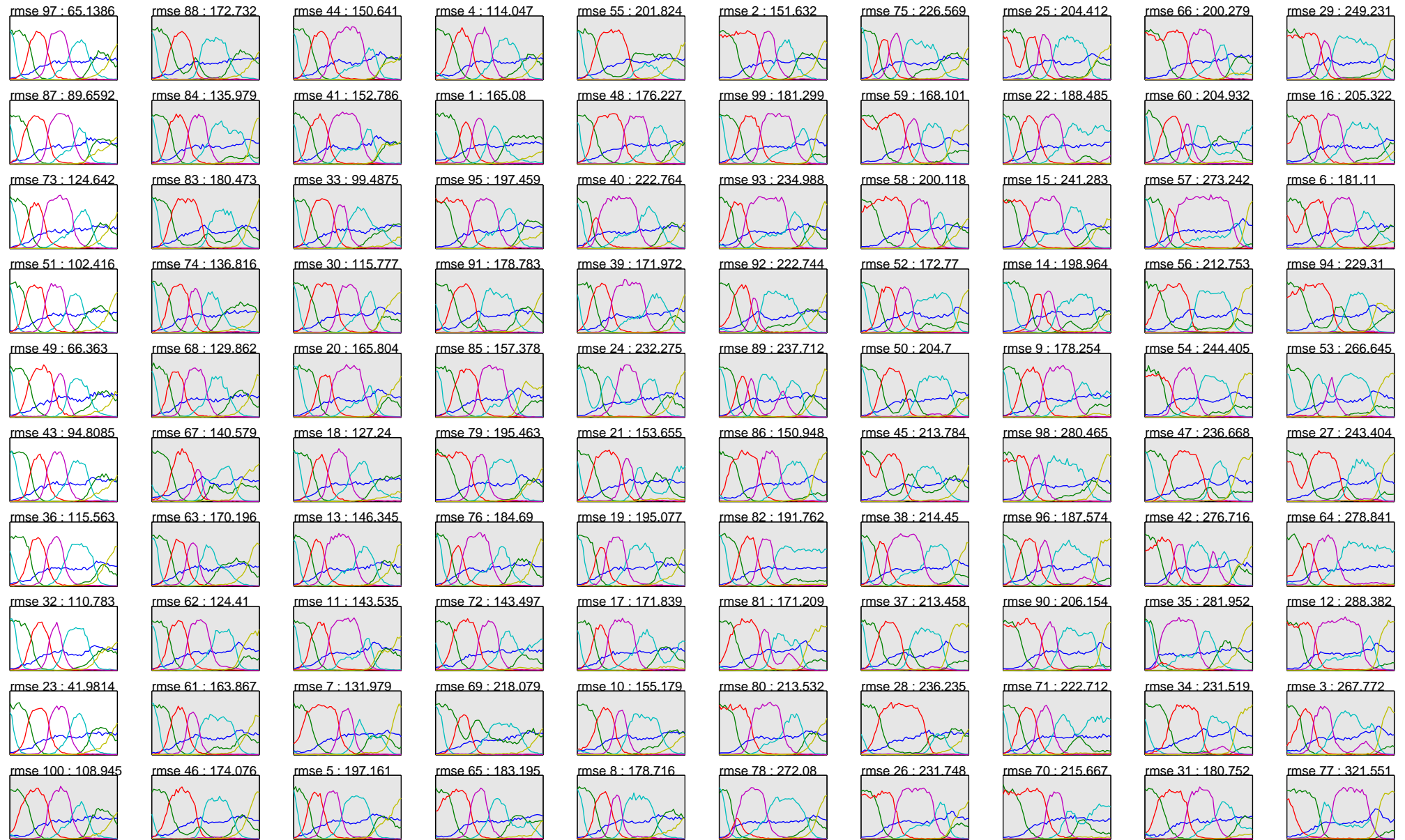

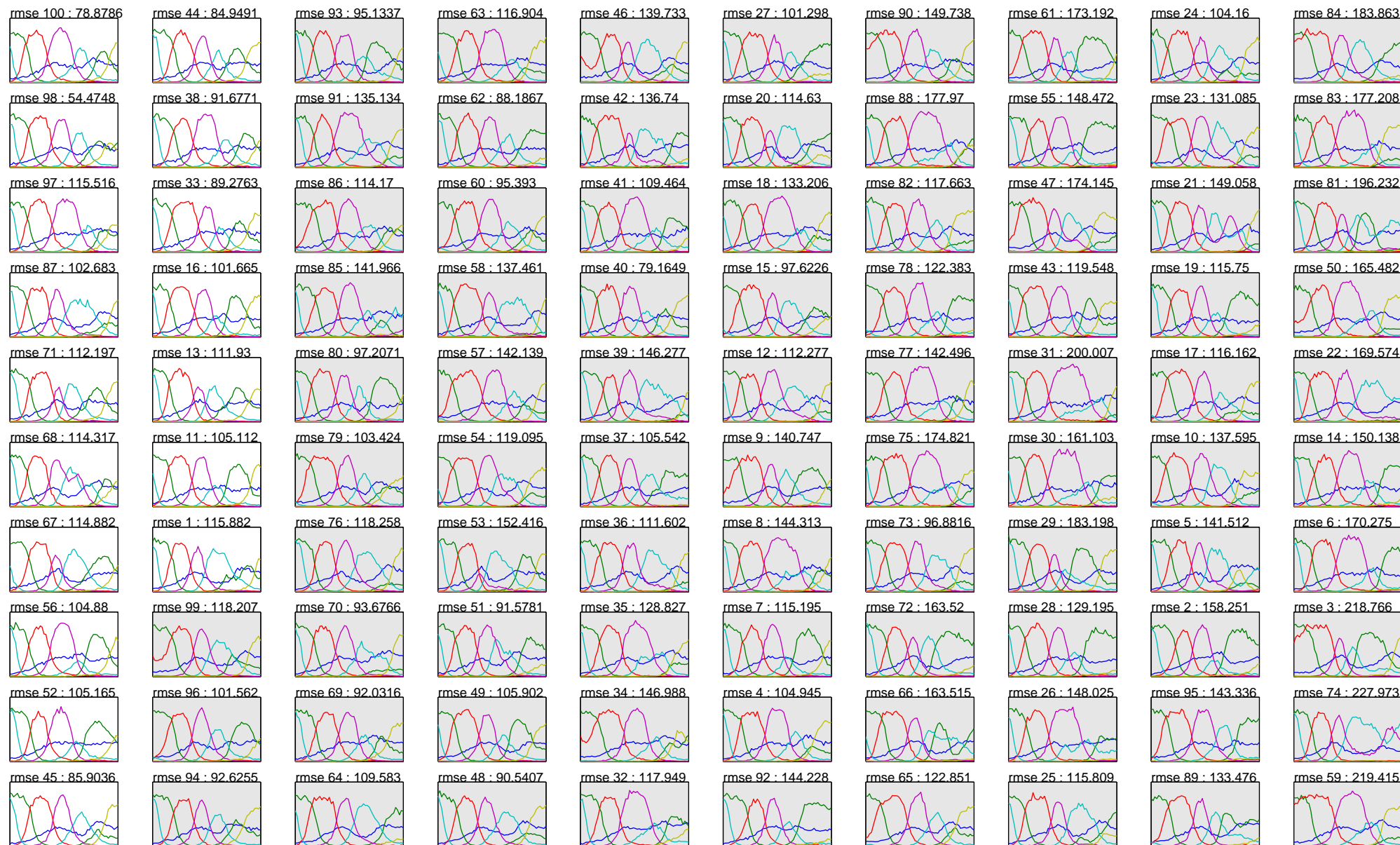

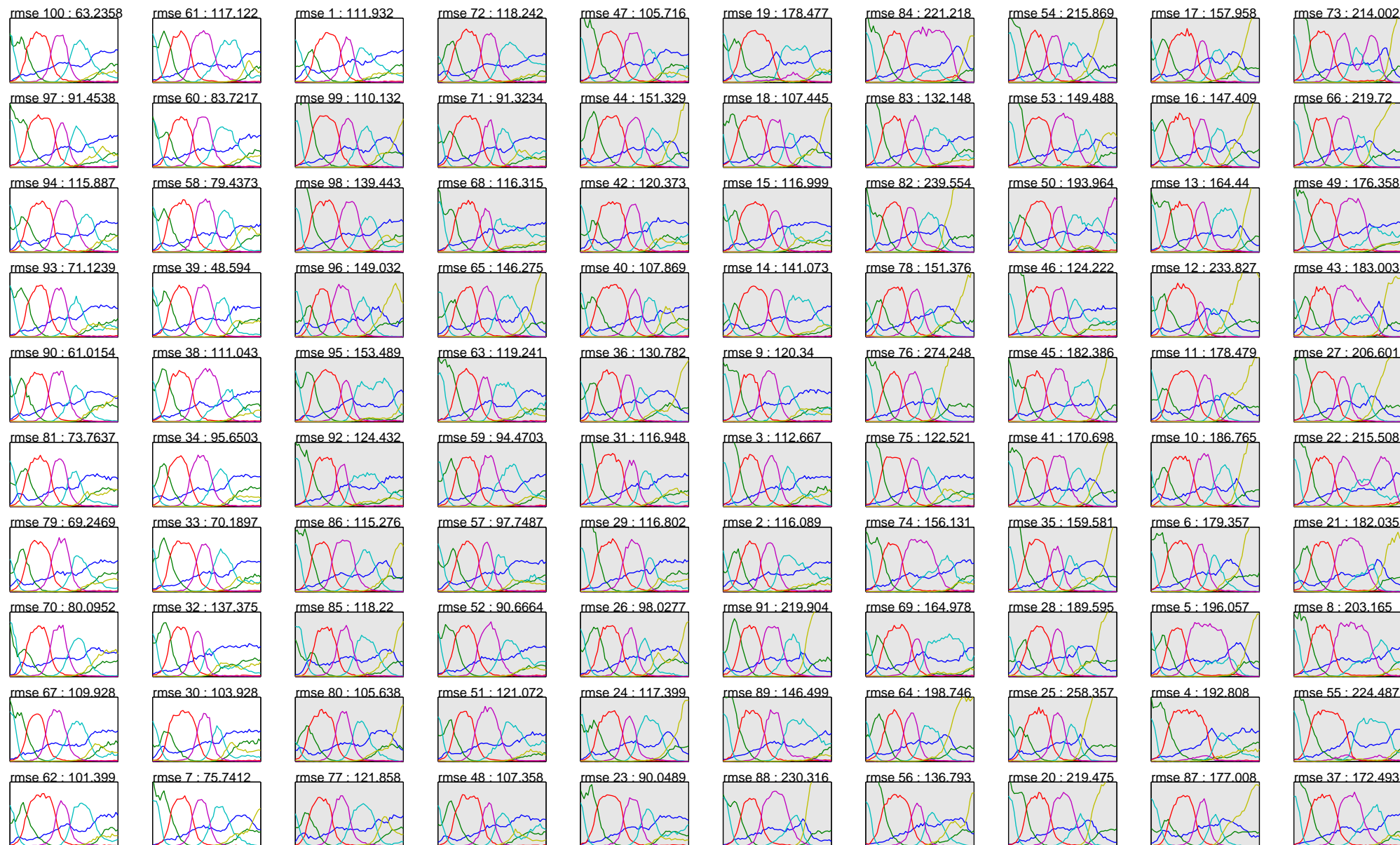

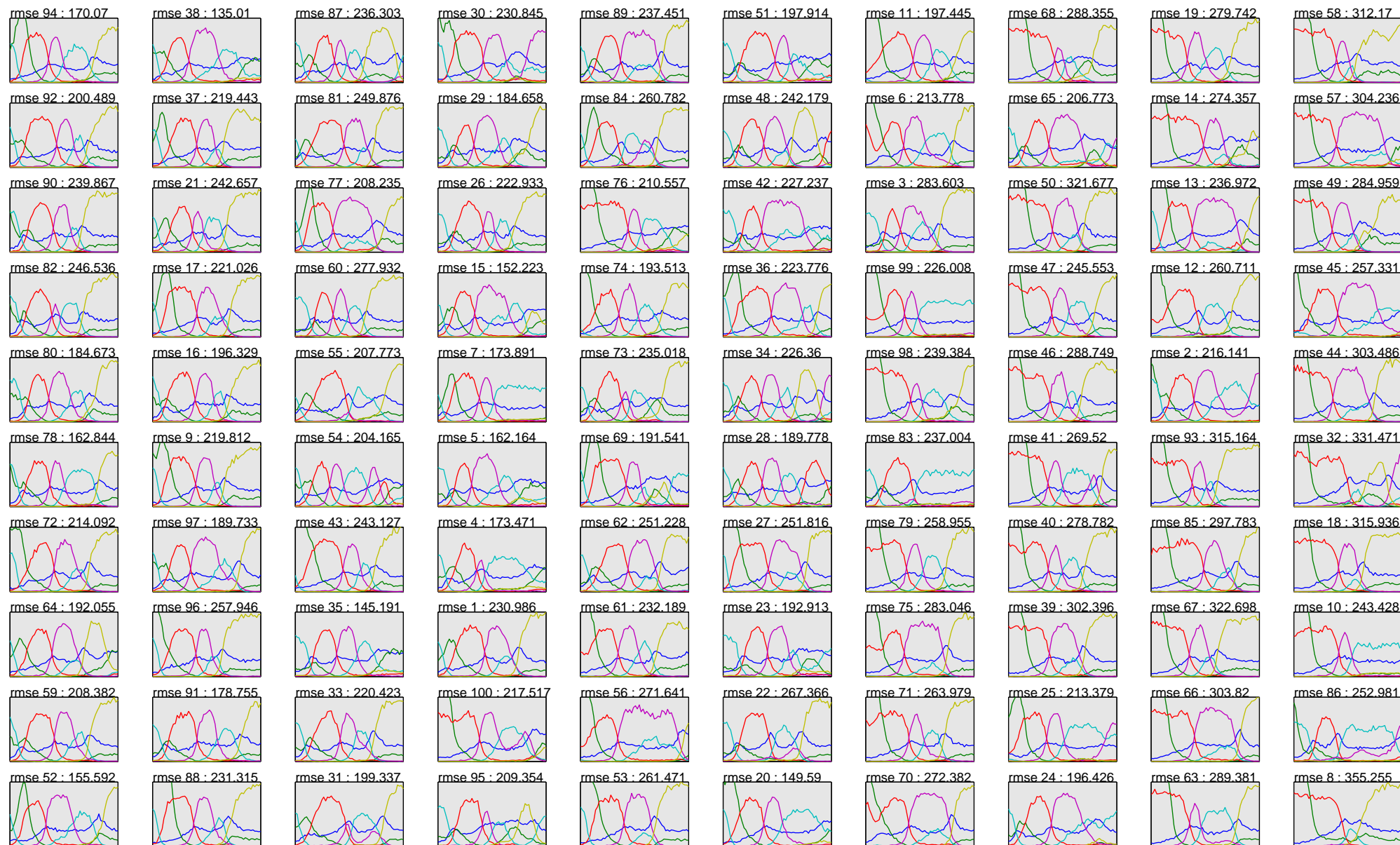

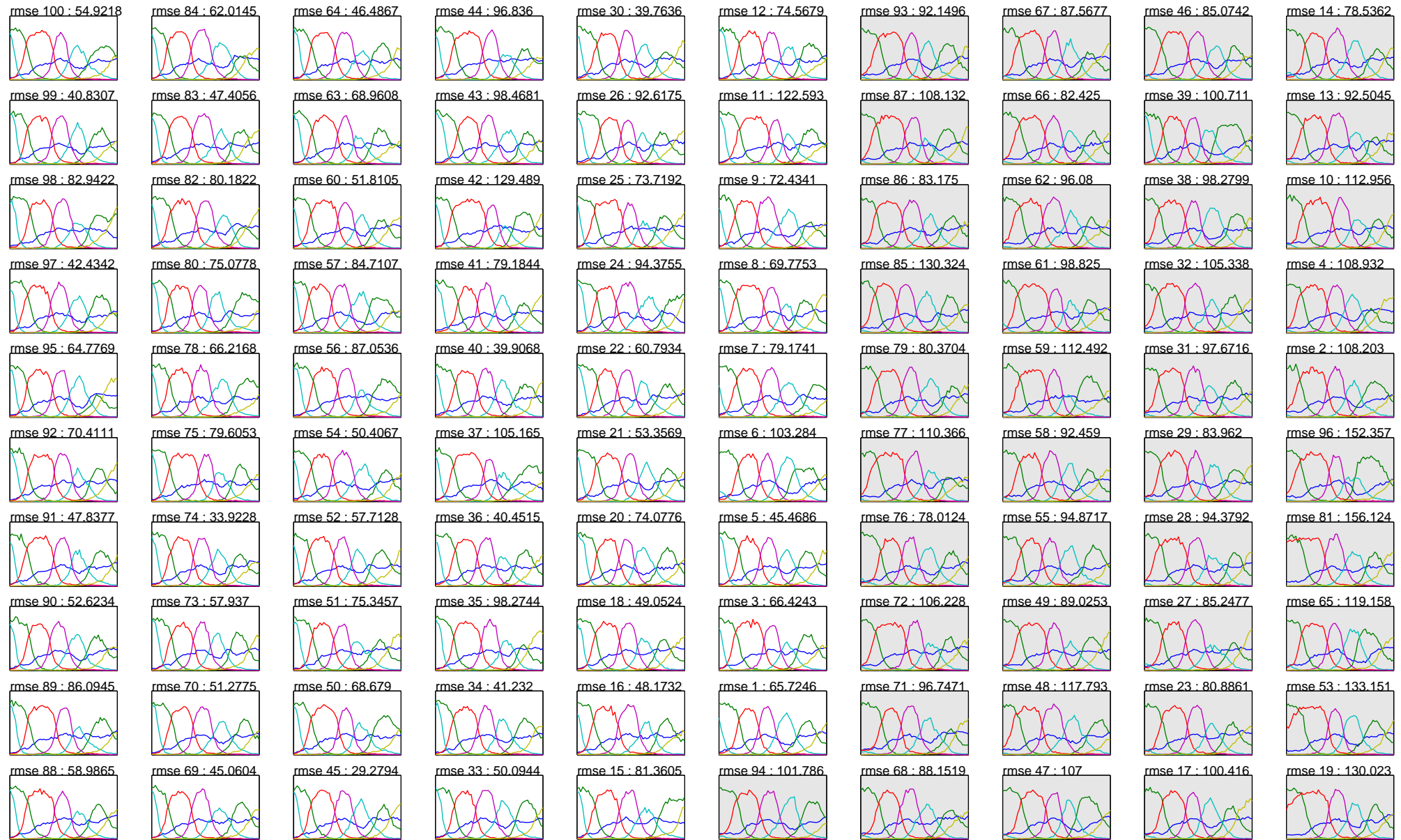

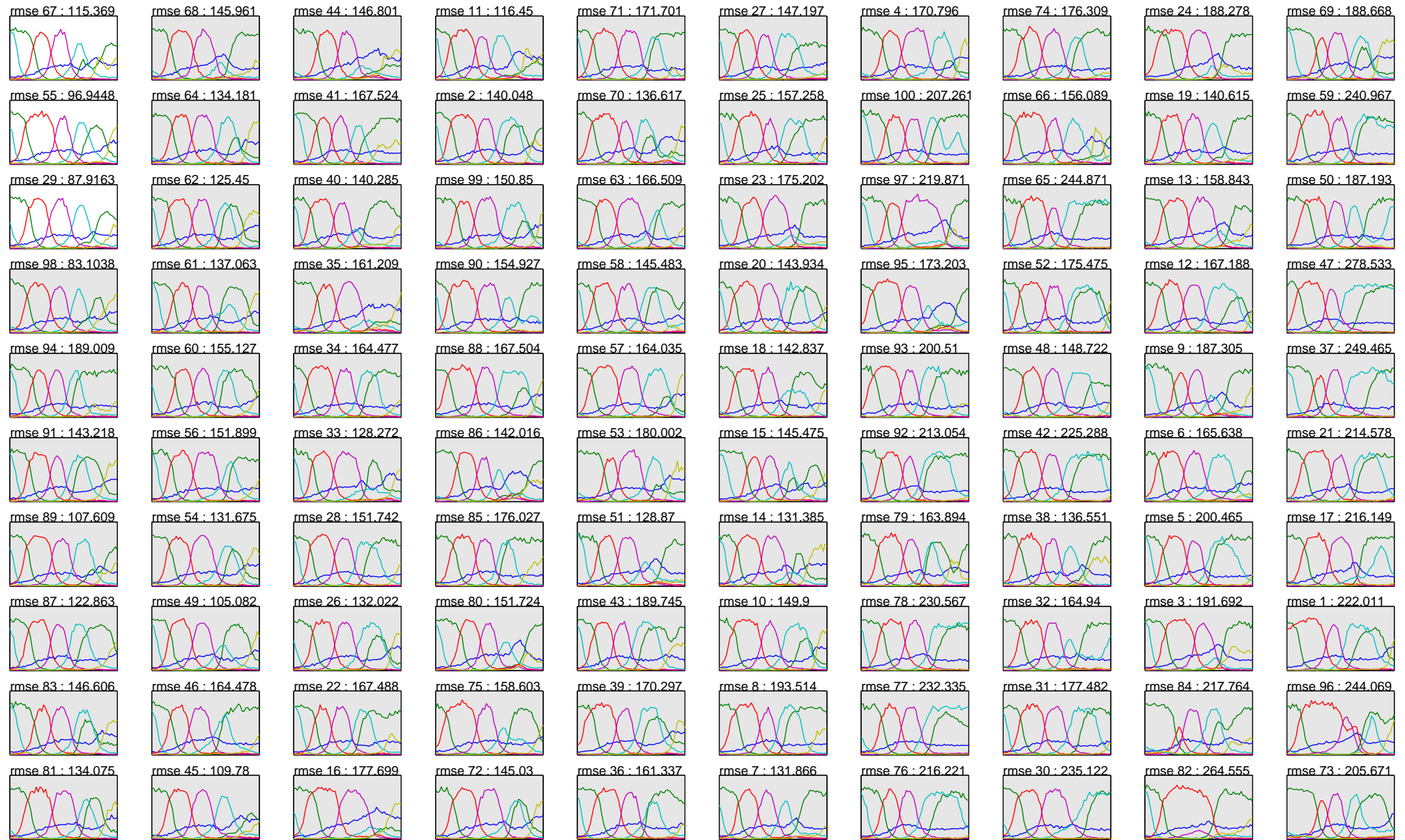

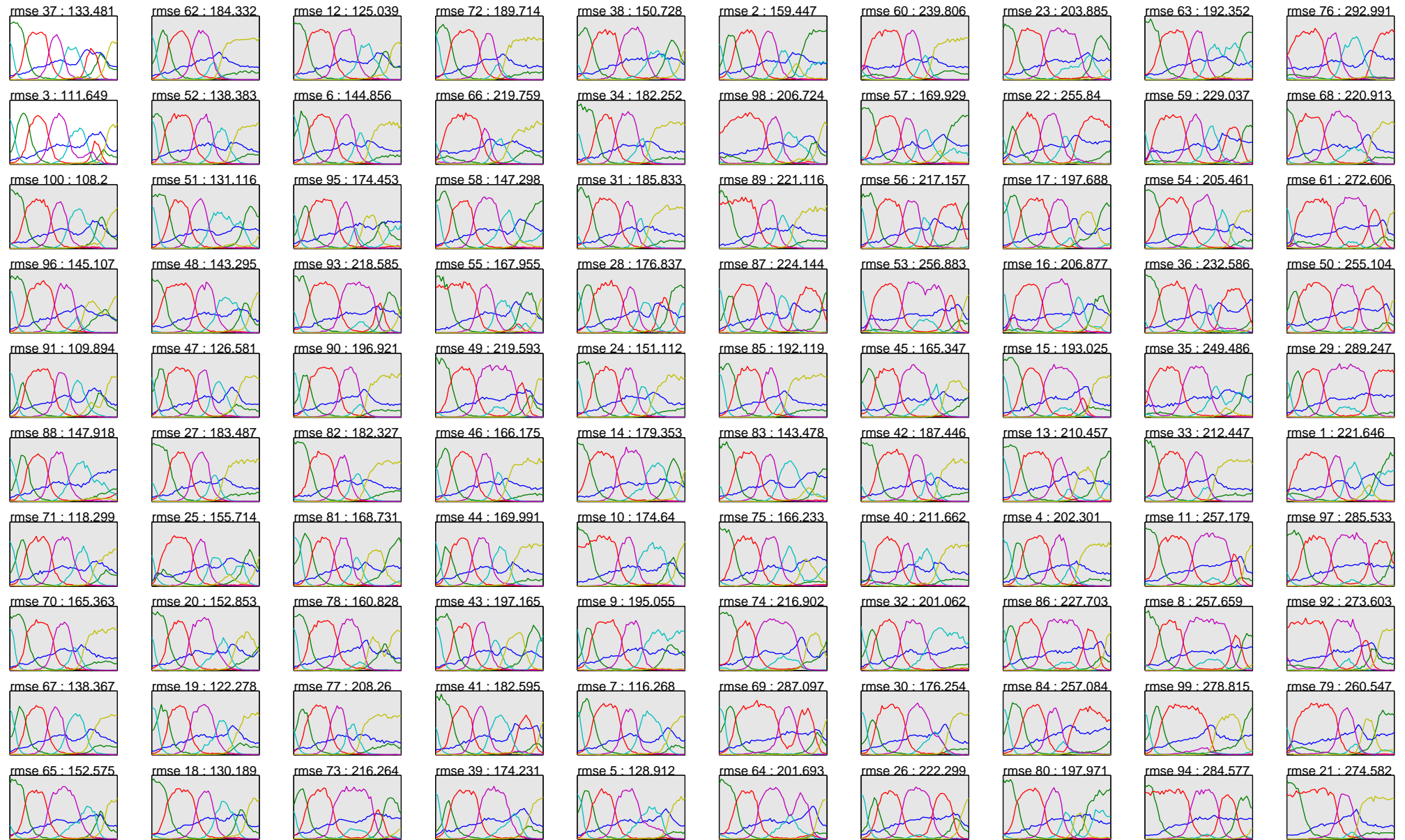

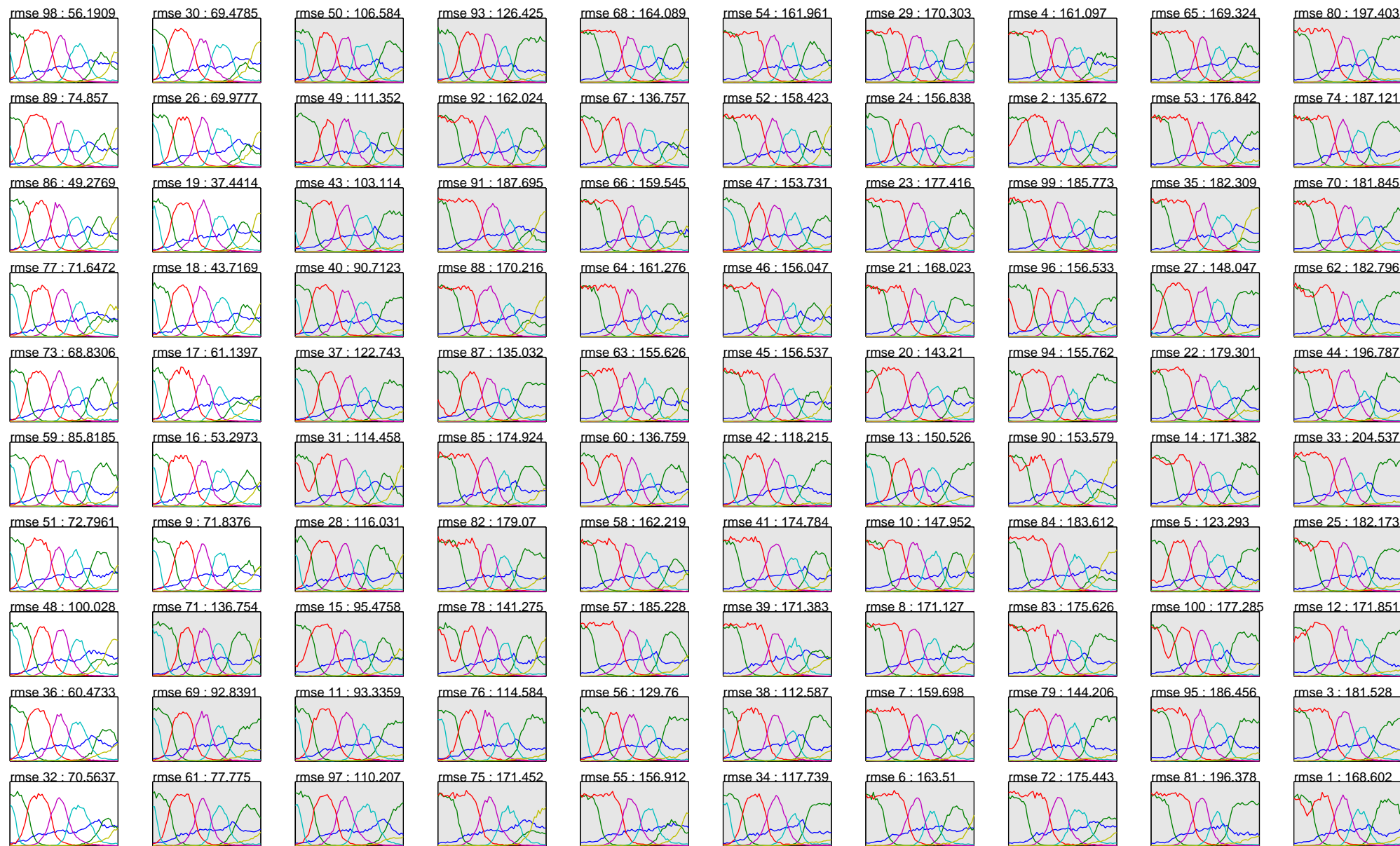

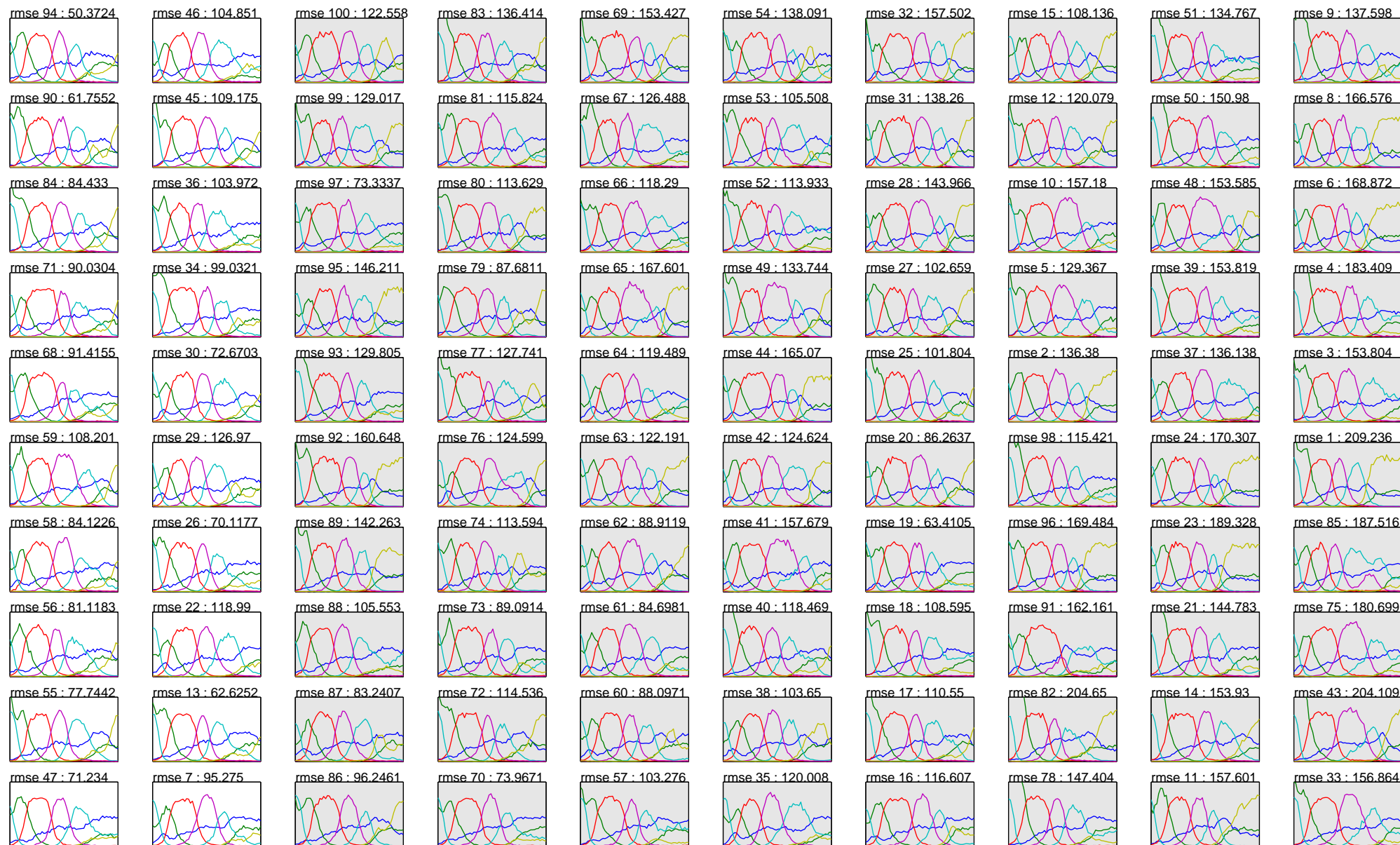

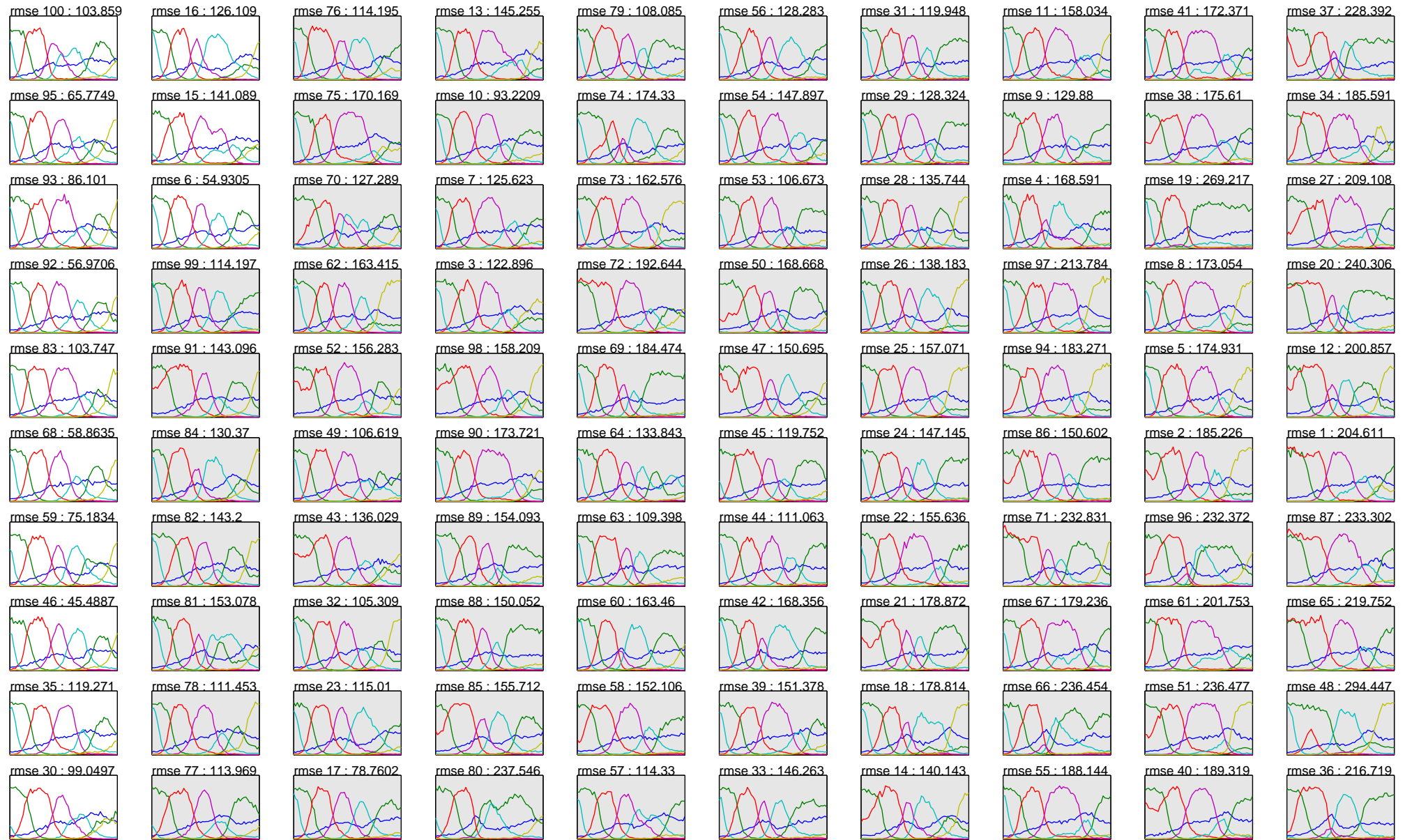

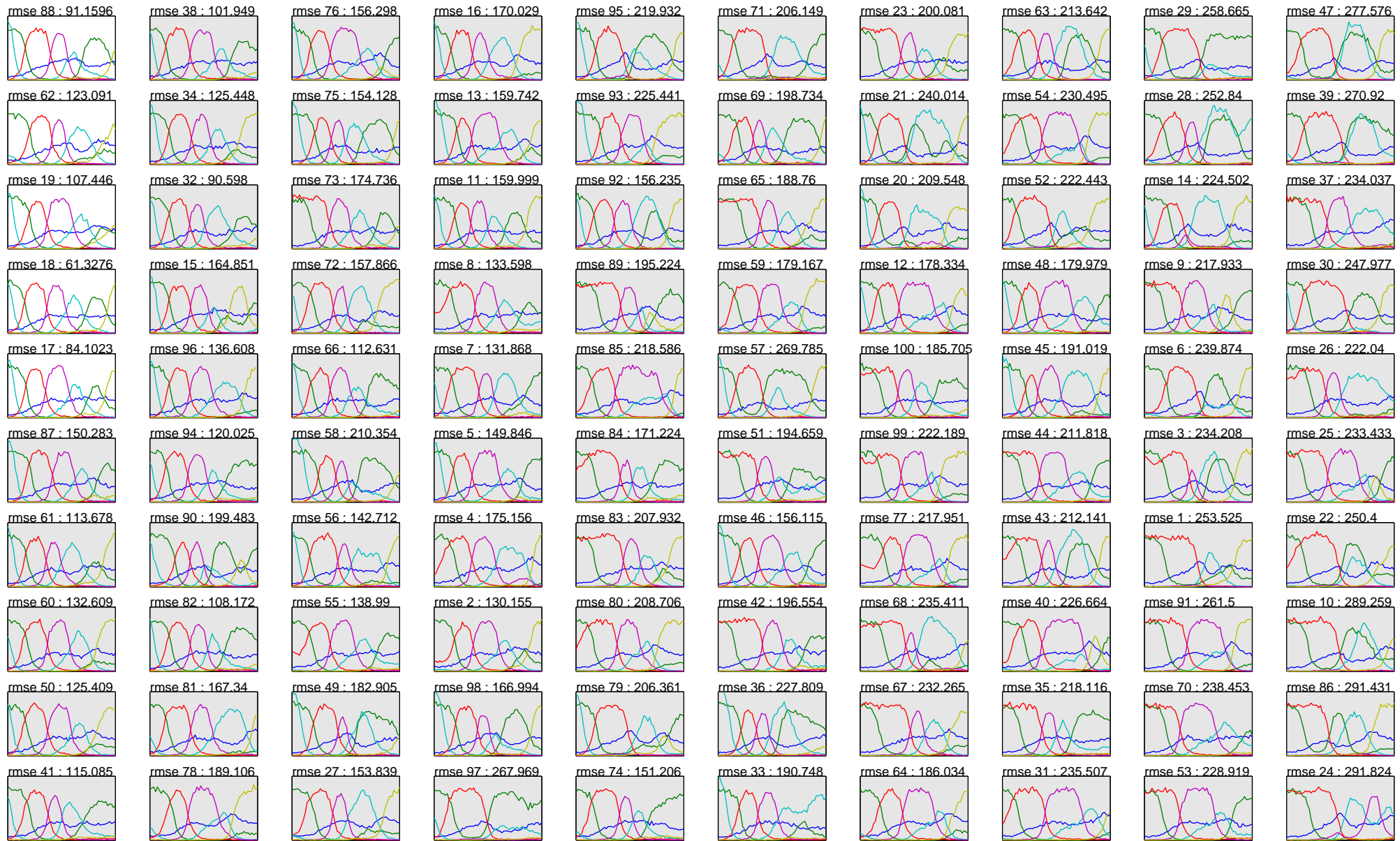

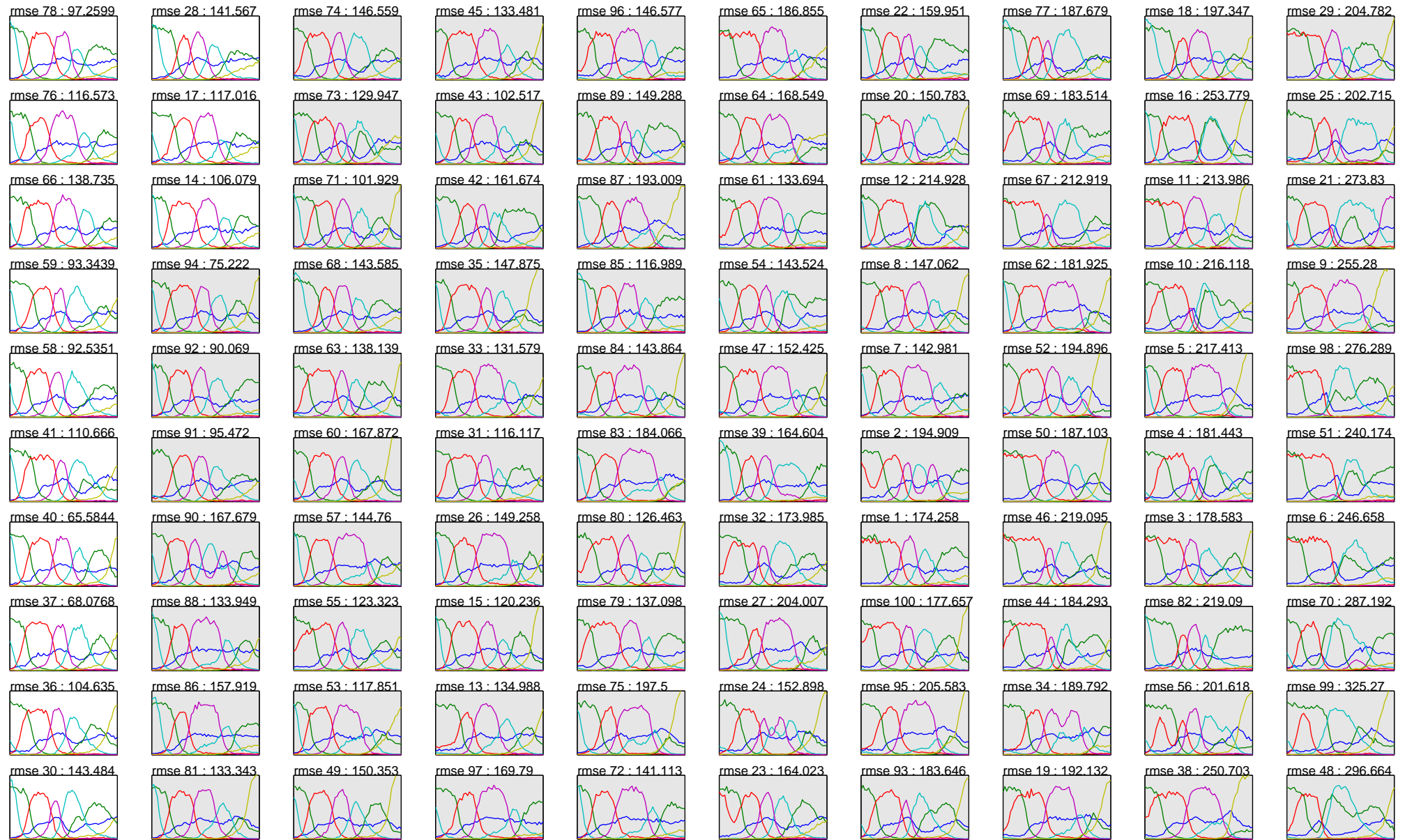

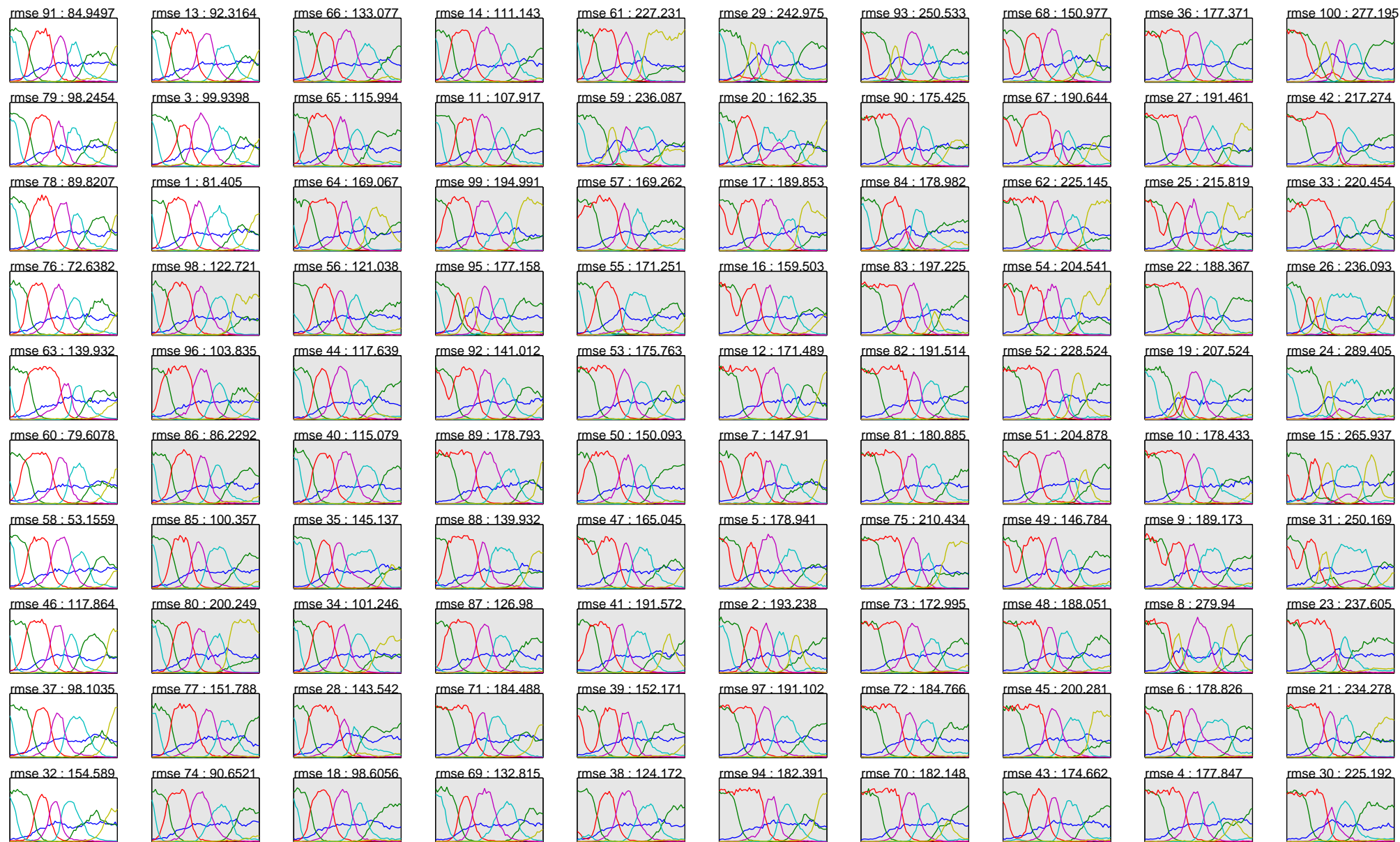

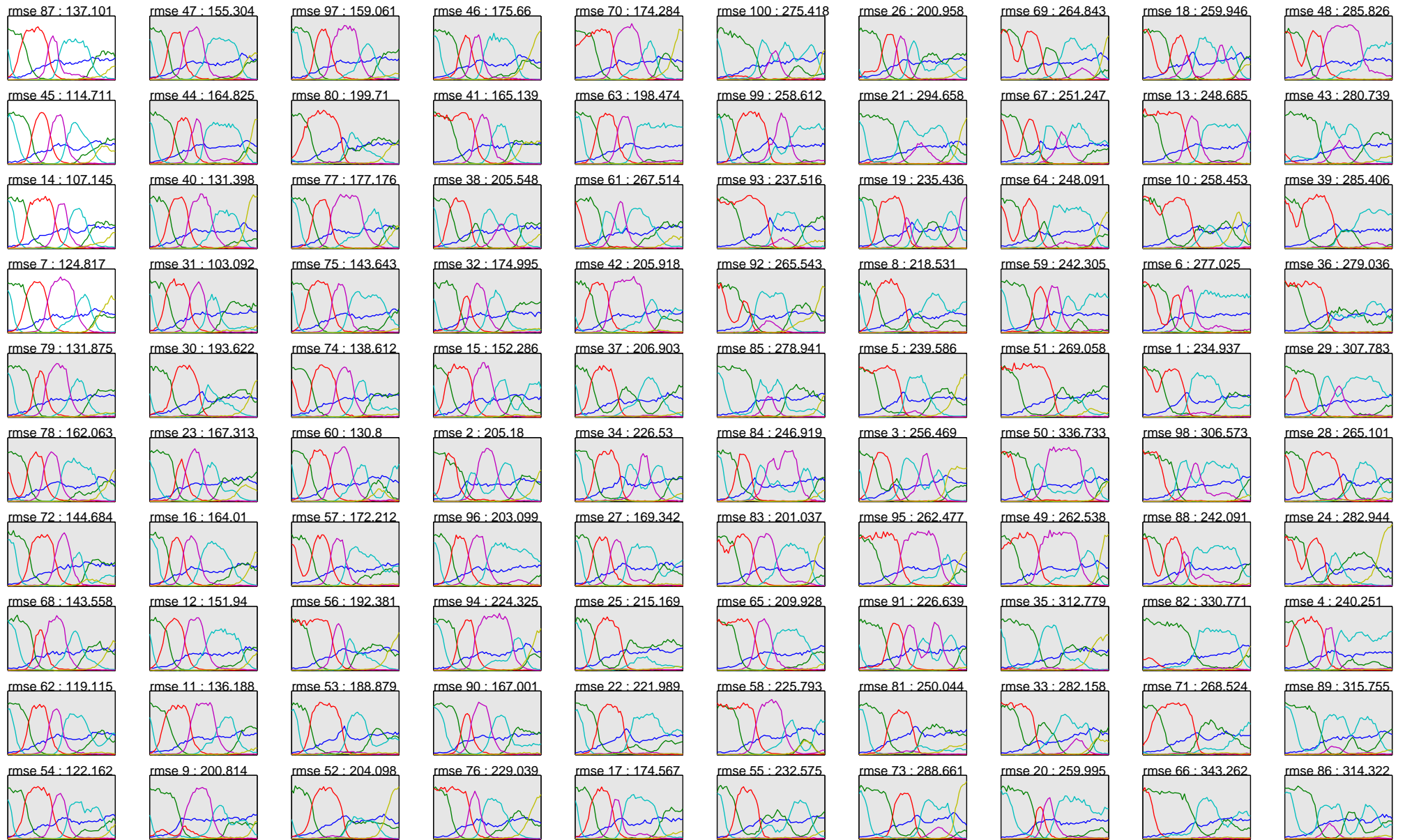

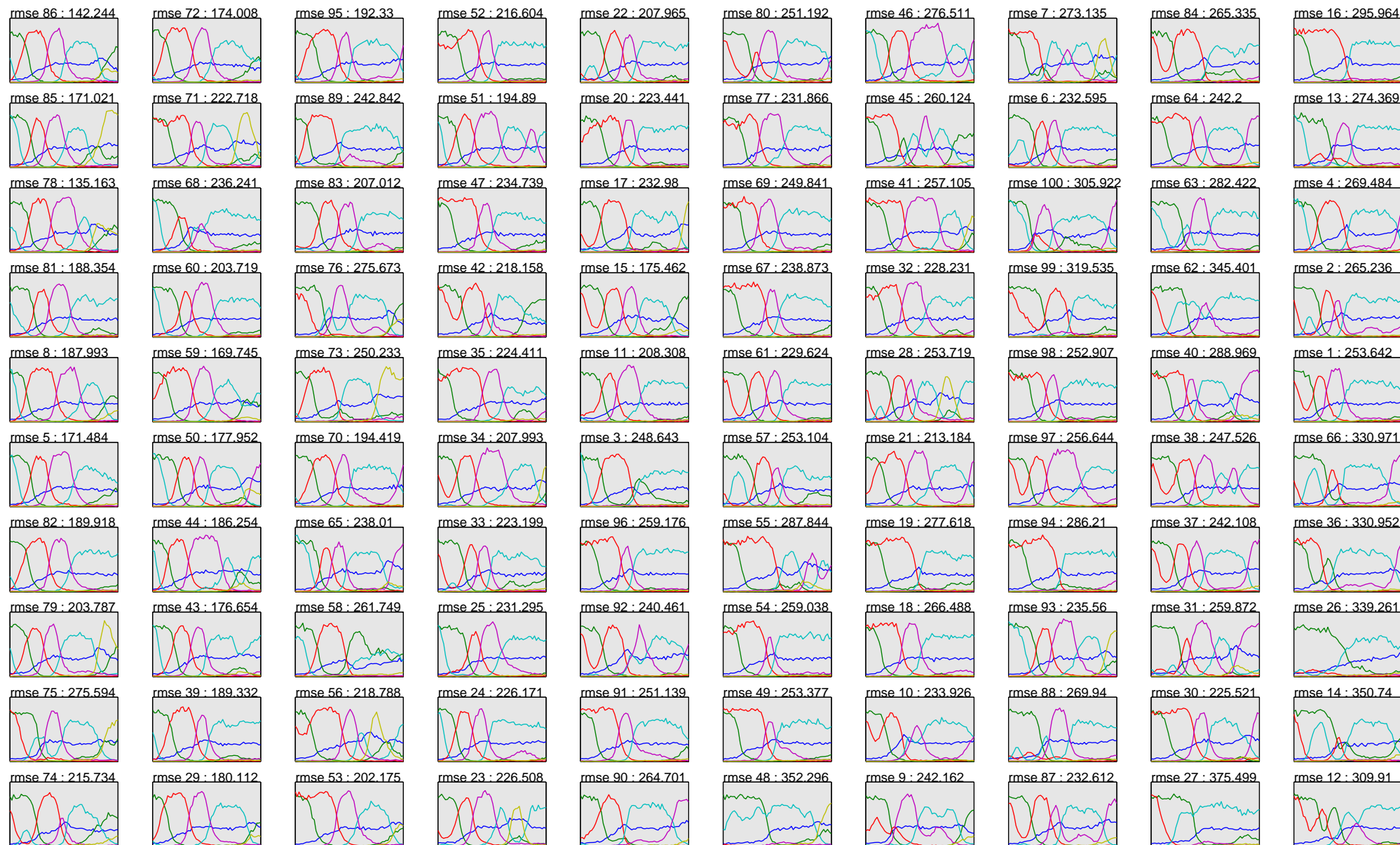

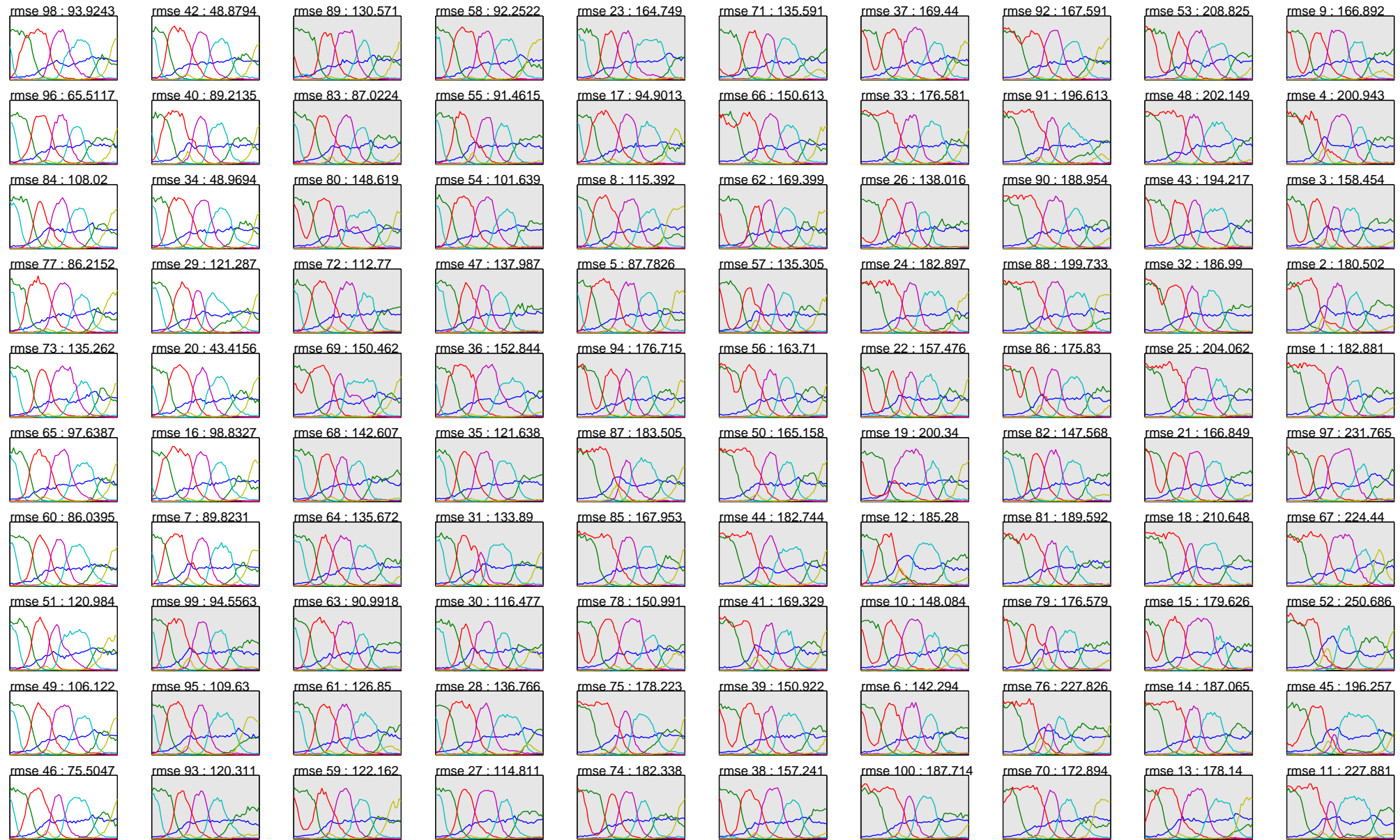

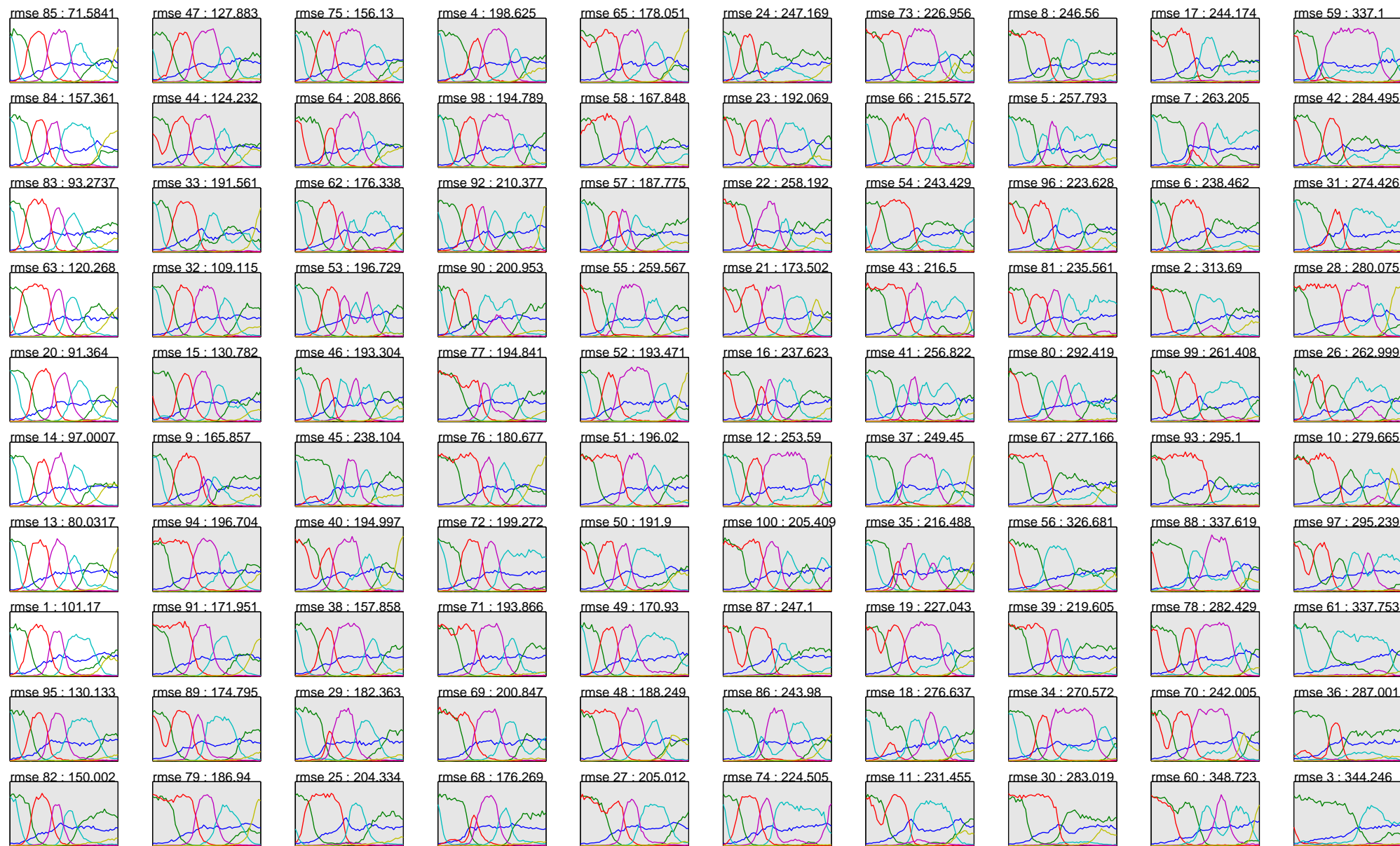

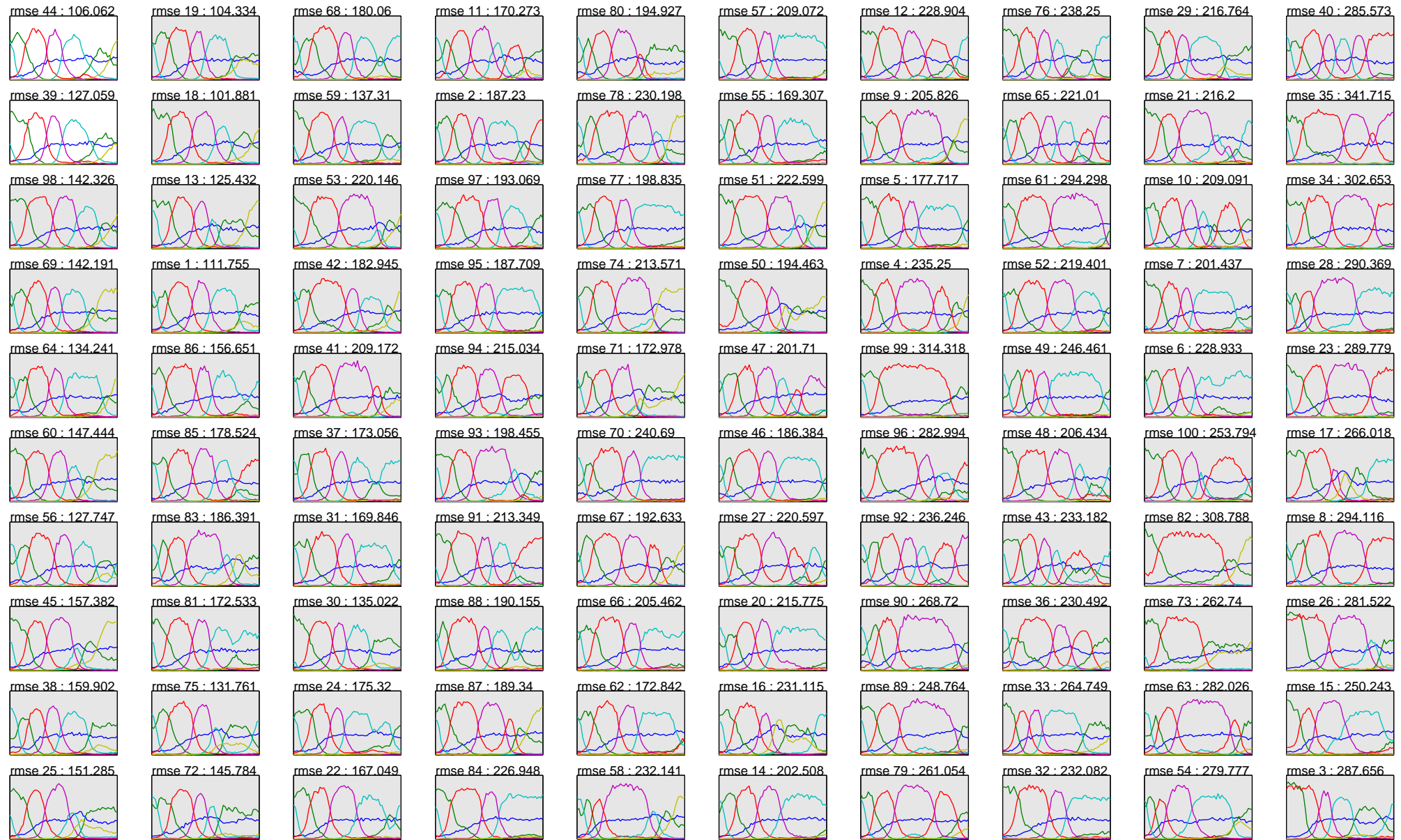

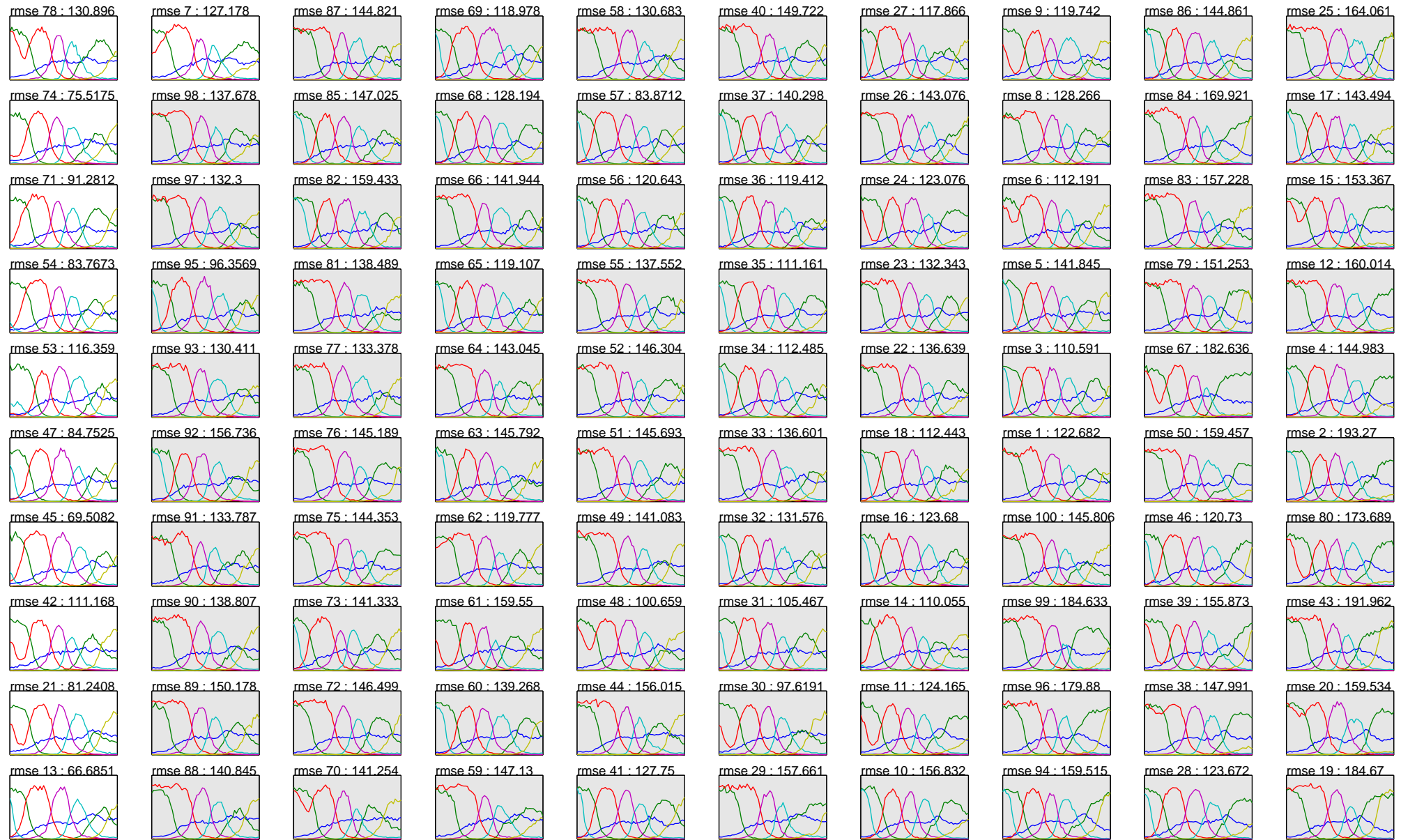

Supplement: Additional file 2 — Stochastic simulation of all circuits. The document (GapGeneModelRobustnessAddFile2.pdf) provided gives the simulation obtained from the 100 stochastic runs of each circuit obtained deterministically. Each page corresponds to a circuit and on each page; individual panels correspond to individual run. The panels with a grey background represent runs that have a defect in one or more expression domains. The runs shown in the panels with a white background are considered to be correct. [file 1752-0509-3-94-S2.PDF]
