# Supplementary material for: Inferring Drosophila gap gene regulatory network: a parameter sensitivity and perturbation analysis
Source: BMC Syst Biol. 2009 Sep 21;3:94. doi: 10.1186/1752-0509-3-94 (PMC2761871; doi:10.1186/1752-0509-3-94)
Supplement: Additional file 3 — Robustness of all circuits. In this document (GapGeneModelRobustnessAddFile3.pdf), each figure shows the deterministic stochastic simulation of a circuit. In these graphs the dashed lines represent the final profiles obtained from the deterministic model and the solid lines the average profiles obtained from the stochastic simulations calculated from the 100 individual stochastic runs, which are shown in grey. [file 1752-0509-3-94-S3.PDF]

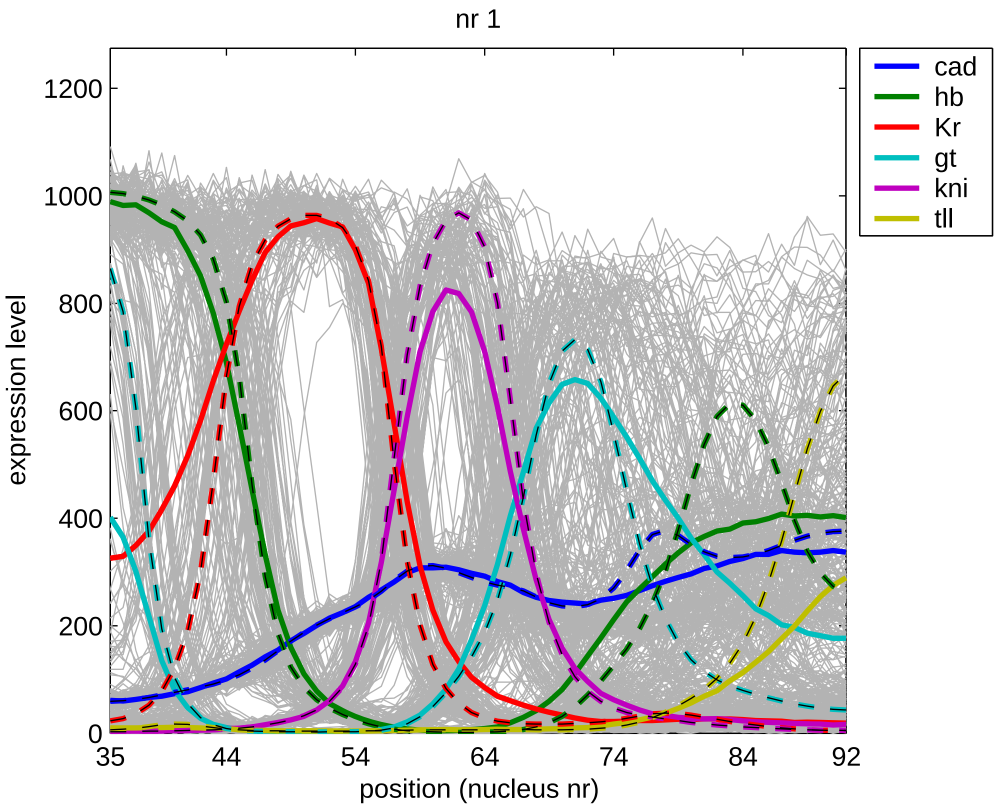

nr 1
expression level
position (nucleus nr)
0
200
400
600
800
1000
1200
35
44
54
64
74
84
92
cad
hb
Kr
gt
kni
tll

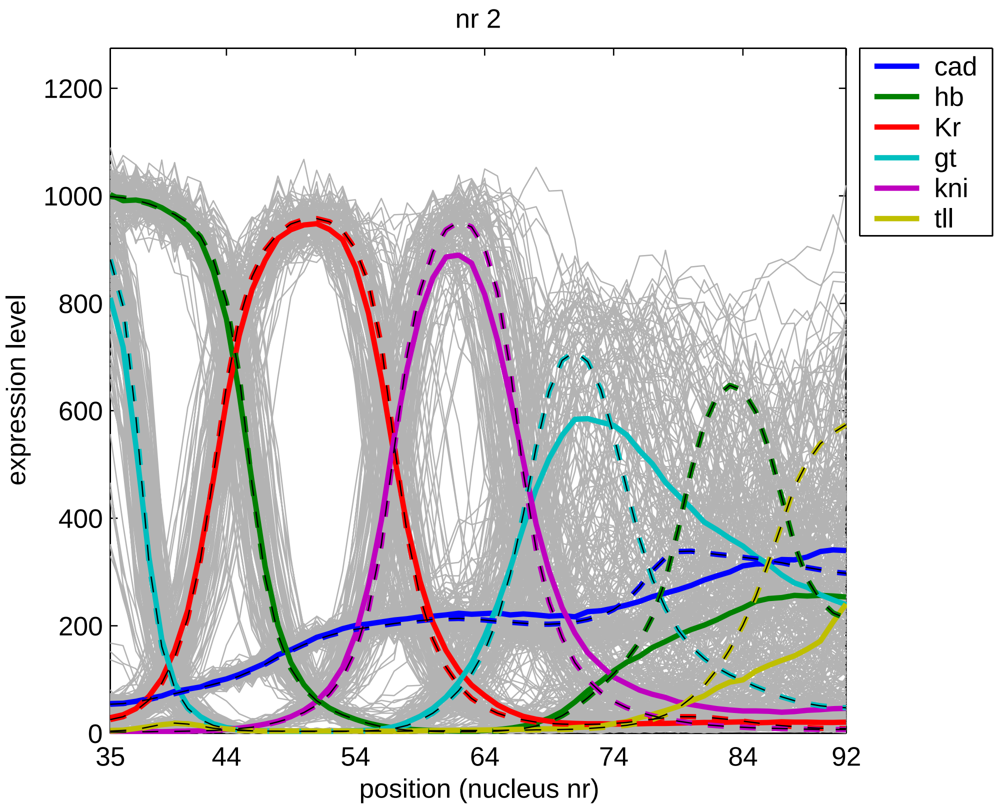
nr 2
expression level
position (nucleus nr)
0
200
400
600
800
1000
1200
35
44
54
64
74
84
92
cad
hb
Kr
gt
kni
tll

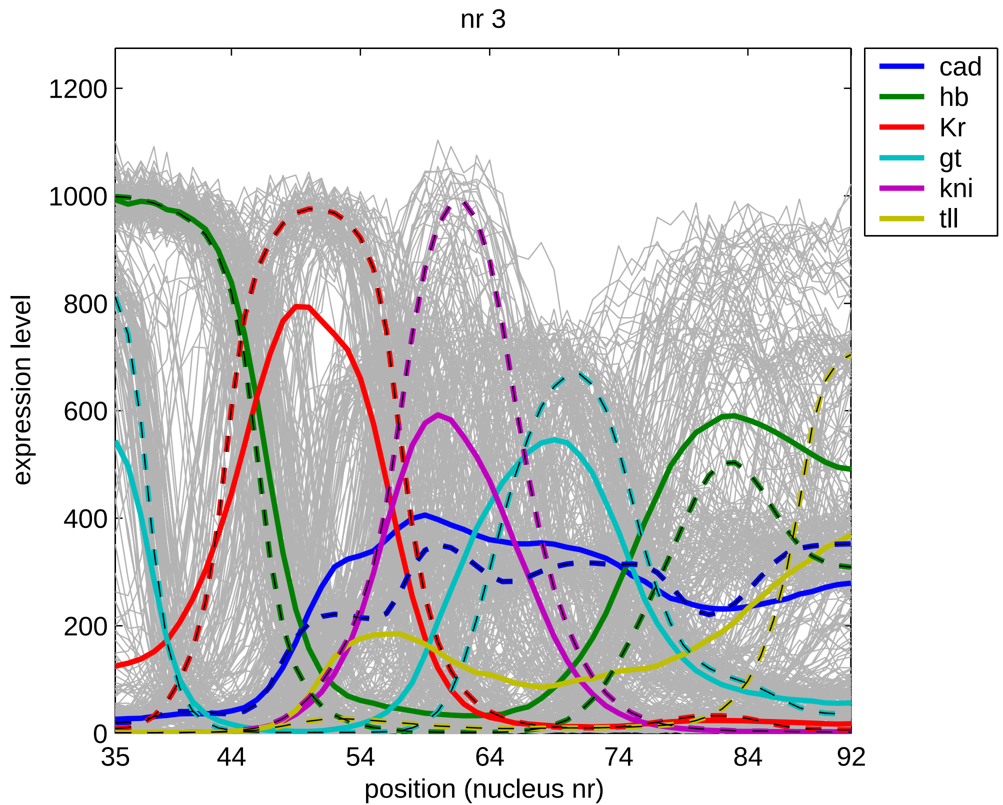
nr 3
expression level
position (nucleus nr)
0
200
400
600
800
1000
1200
35
44
54
64
74
84
92
cad
hb
Kr
gt
kni
tll

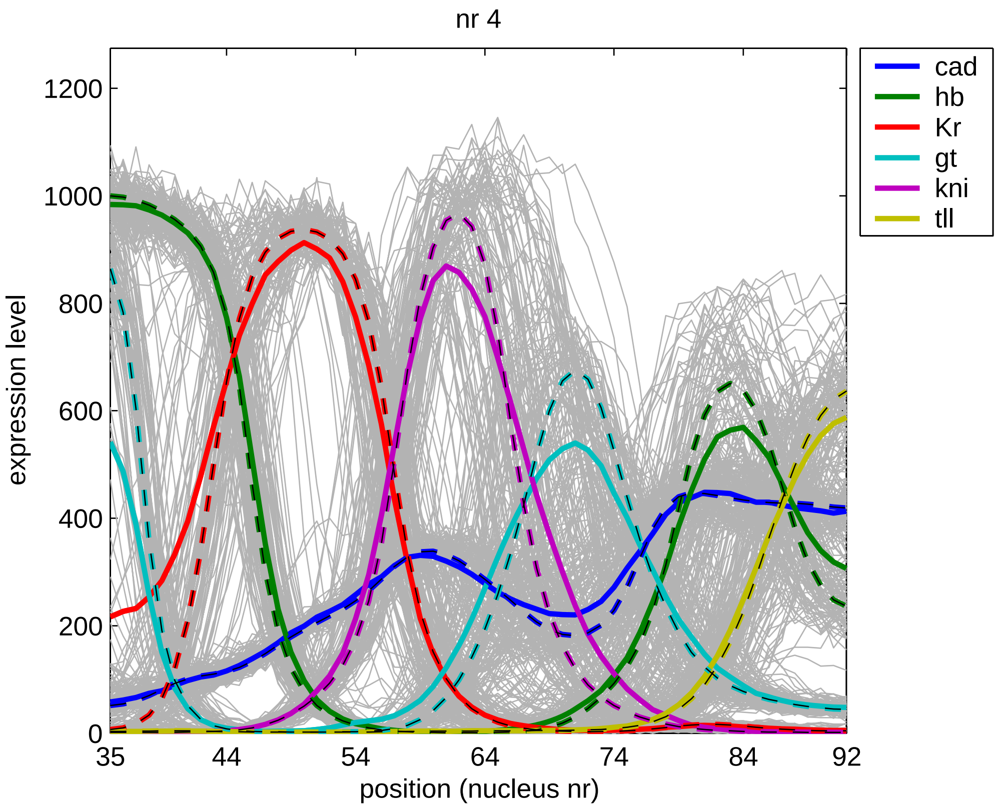
nr 4
cad
hb
Kr
gt
kni
tll
expression level
0
200
400
600
800
1000
1200
35
44
54
64
74
84
92
position (nucleus nr)

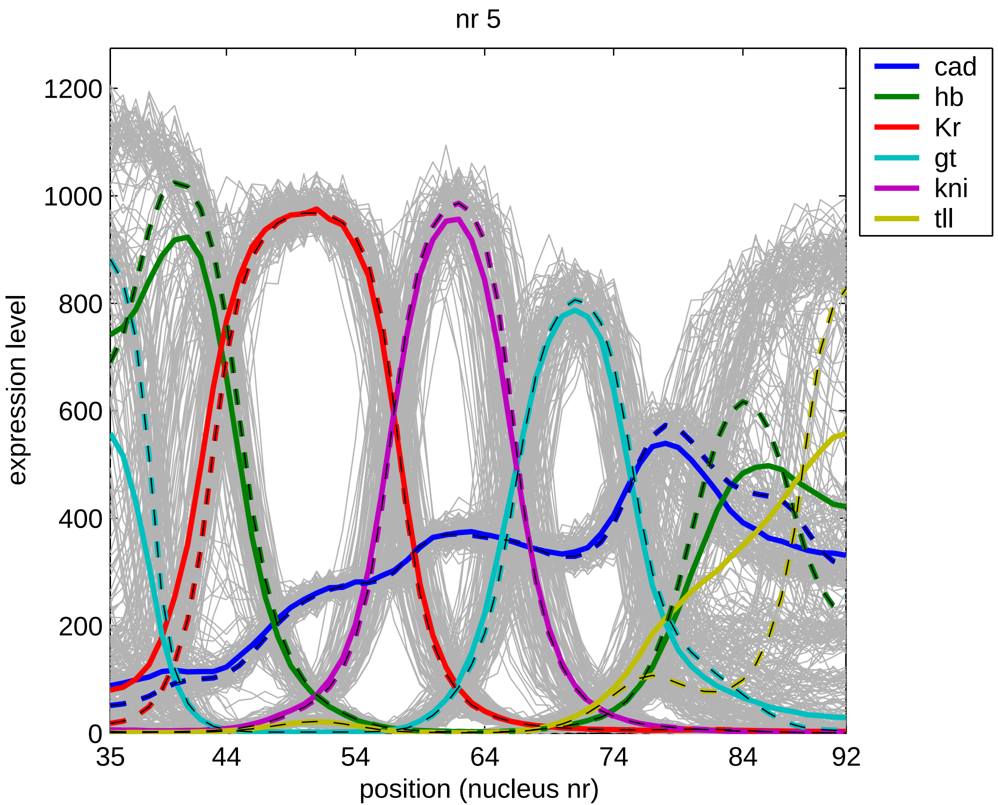

nr 5
expression level
position (nucleus nr)
0
200
400
600
800
1000
1200
35
44
54
64
74
84
92
cad
hb
Kr
gt
kni
tll

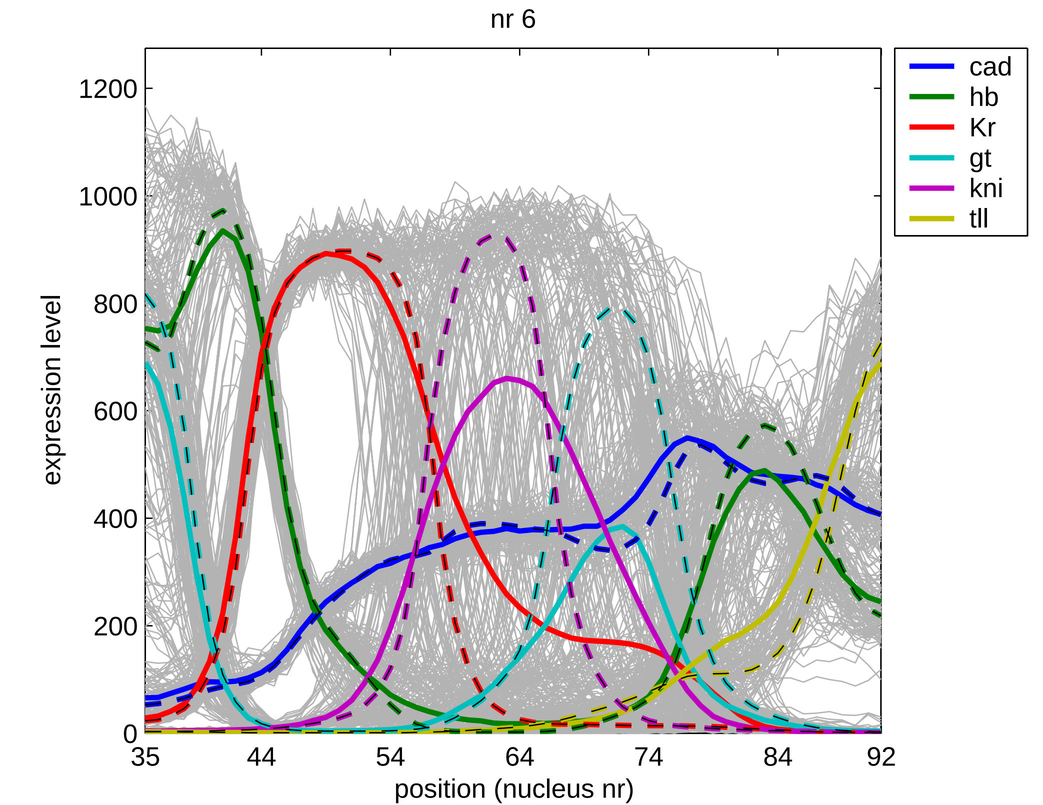
nr 6
expression level
position (nucleus nr)
0
200
400
600
800
1000
1200
35
44
54
64
74
84
92
cad
hb
Kr
gt
kni
tll

nr 7

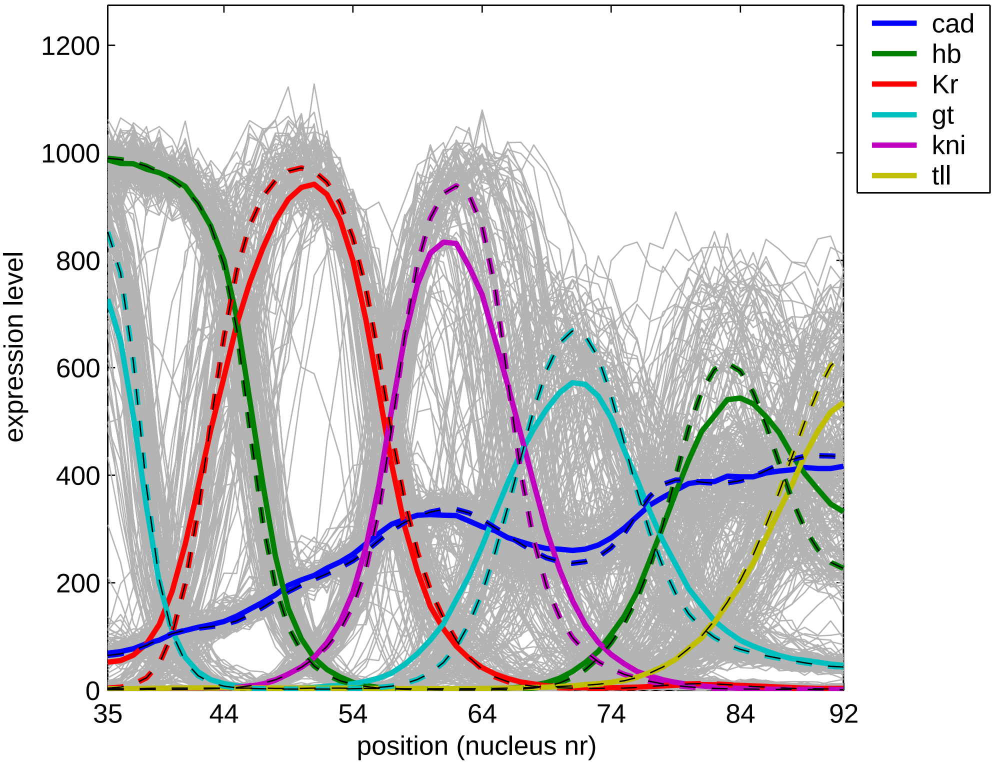

expression level
position (nucleus nr)
0
200
400
600
800
1000
1200
35
44
54
64
74
84
92
cad
hb
Kr
gt
kni
tll

nr 8

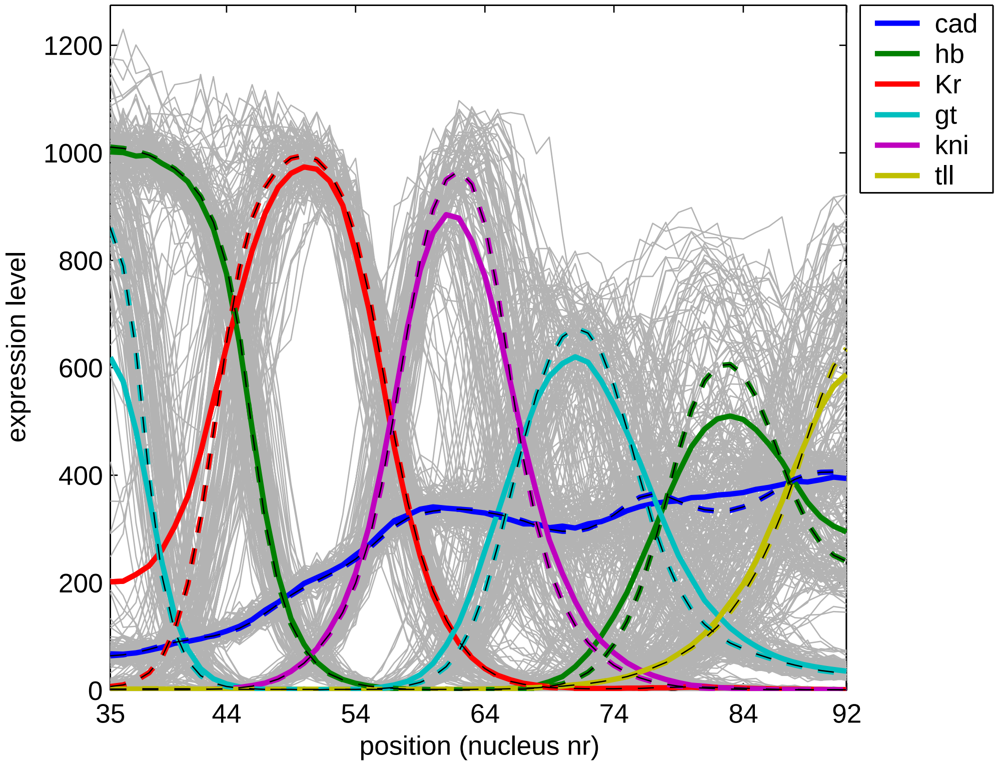

0
200
400
600
800
1000
1200
expression level
35
44
54
64
74
84
92
position (nucleus nr)
cad
hb
Kr
gt
kni
tll

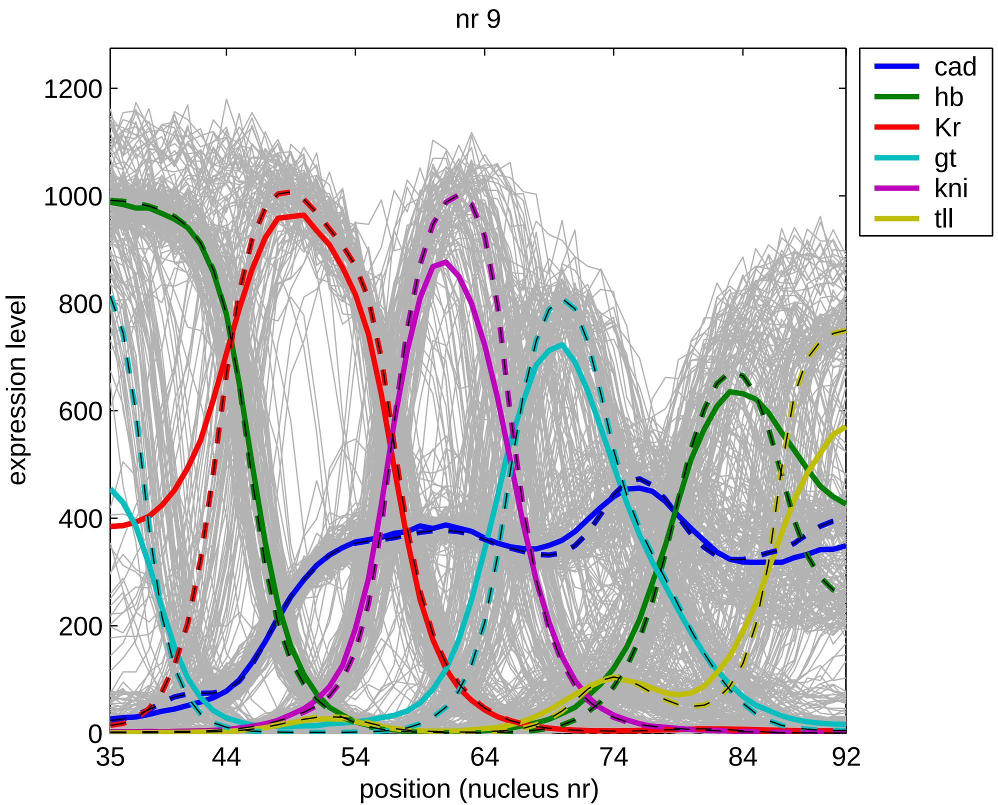

nr 9
expression level
position (nucleus nr)
0
200
400
600
800
1000
1200
35
44
54
64
74
84
92
cad
hb
Kr
gt
kni
tll

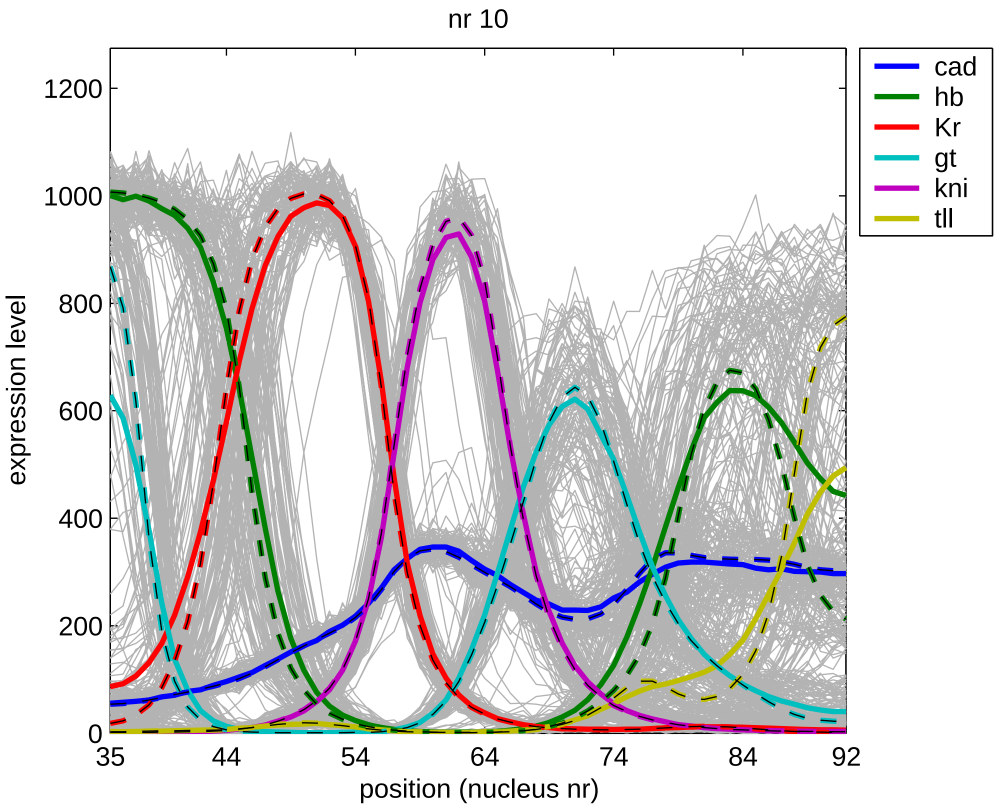

nr 10
expression level
0
200
400
600
800
1000
1200
35
44
54
64
74
84
92
position (nucleus nr)
cad
hb
Kr
gt
kni
tll

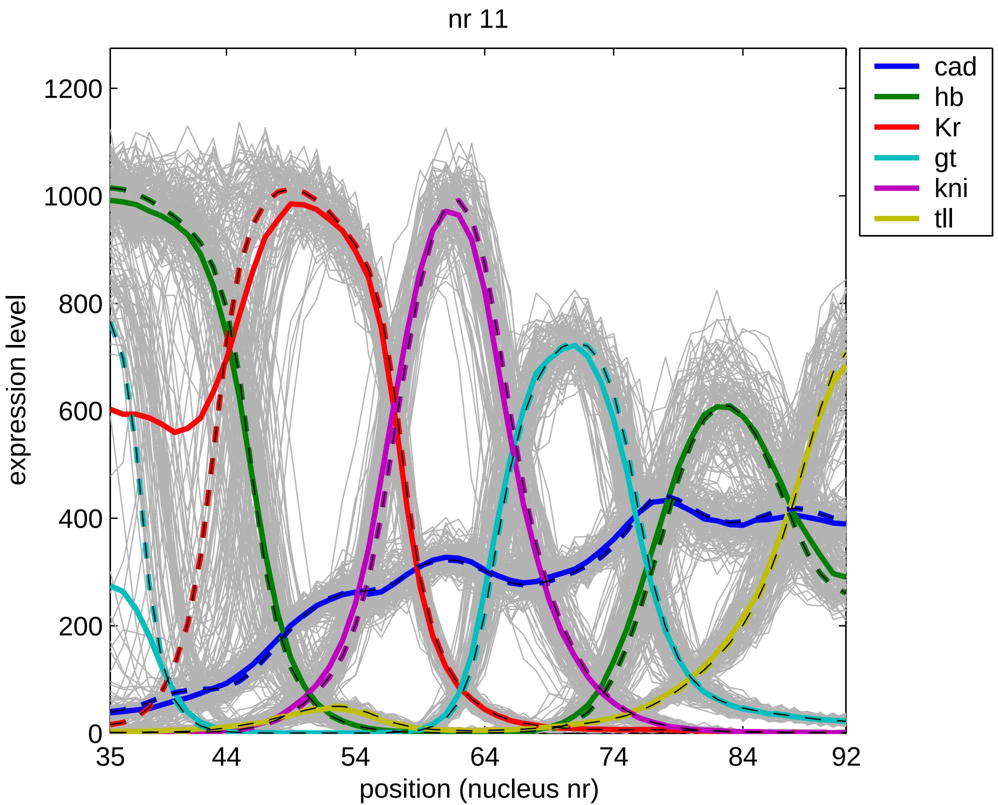
nr 11
expression level
position (nucleus nr)
0
200
400
600
800
1000
1200
35
44
54
64
74
84
92
cad
hb
Kr
gt
kni
tll

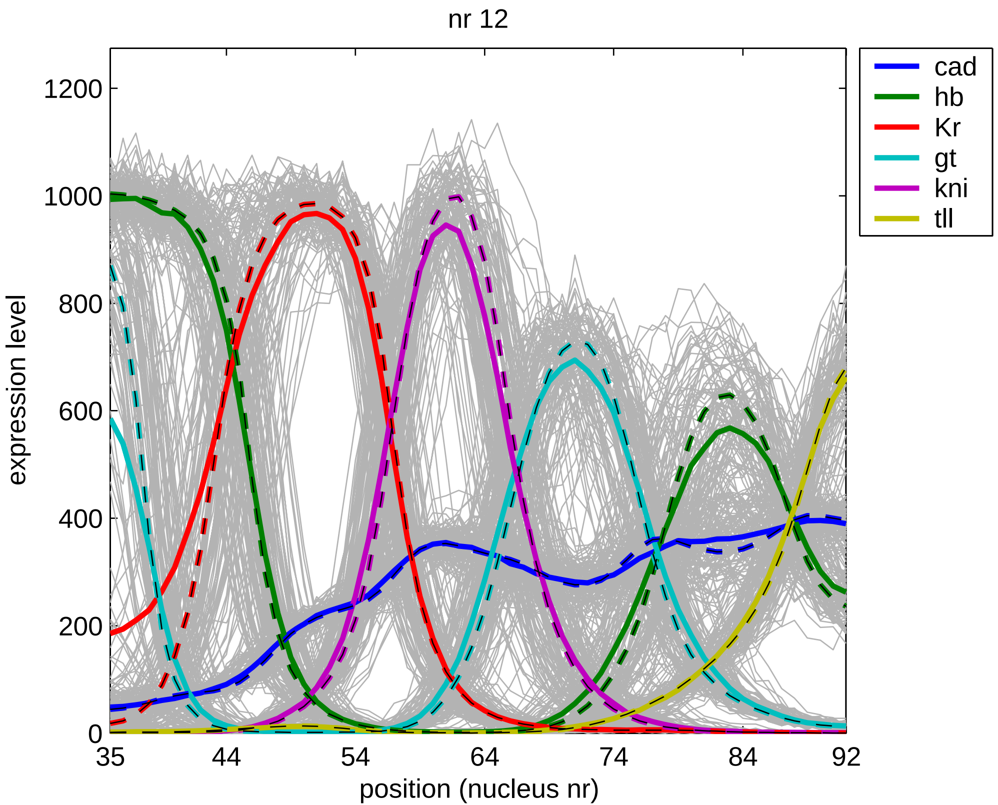

nr 12
cad
hb
Kr
gt
kni
tll
expression level
0
200
400
600
800
1000
1200
35
44
54
64
74
84
92
position (nucleus nr)

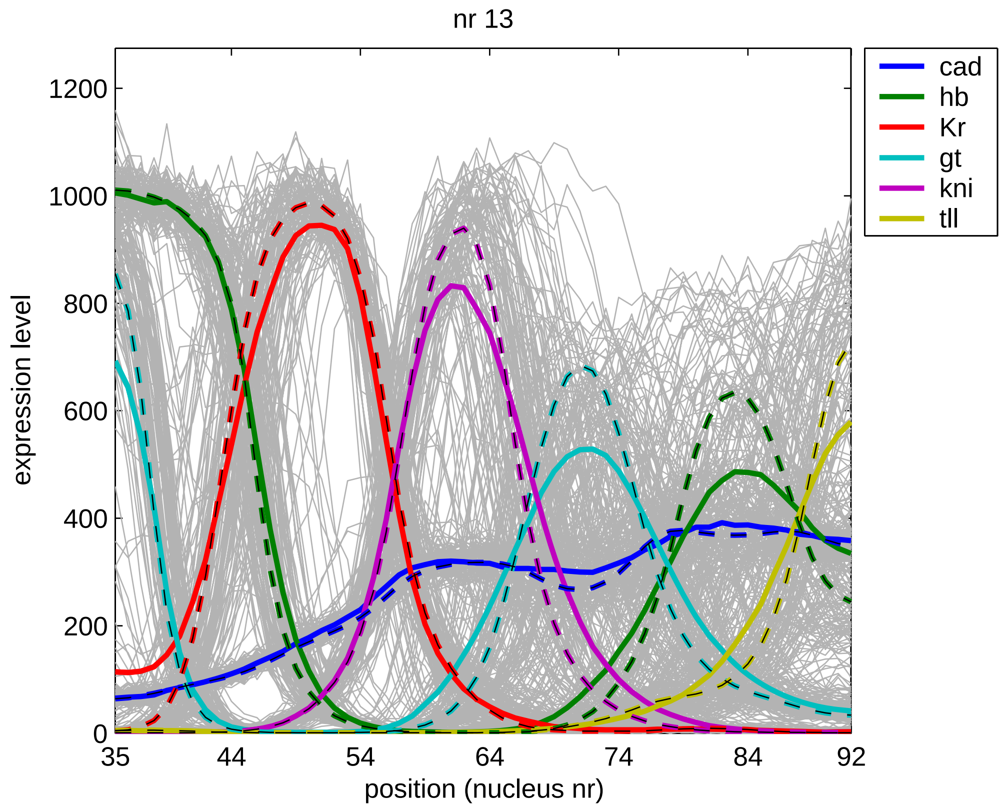
nr 13
expression level
position (nucleus nr)
0
200
400
600
800
1000
1200
35
44
54
64
74
84
92
cad
hb
Kr
gt
kni
tll

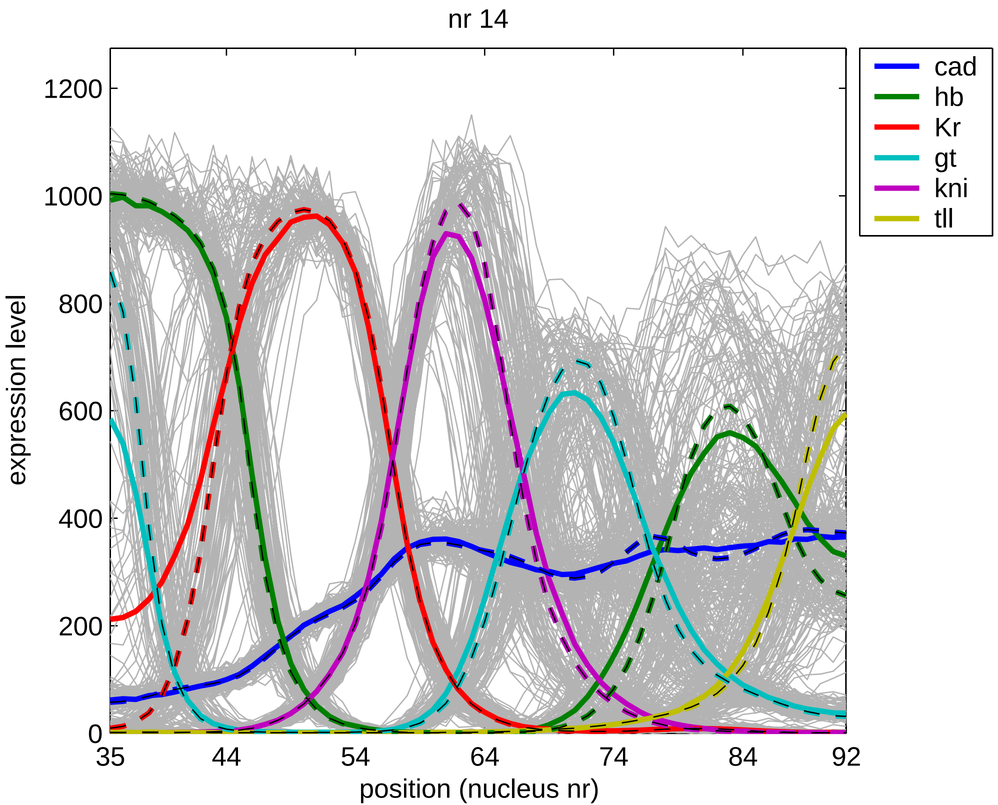

nr 14
expression level
position (nucleus nr)
0
200
400
600
800
1000
1200
35
44
54
64
74
84
92
cad
hb
Kr
gt
kni
tll

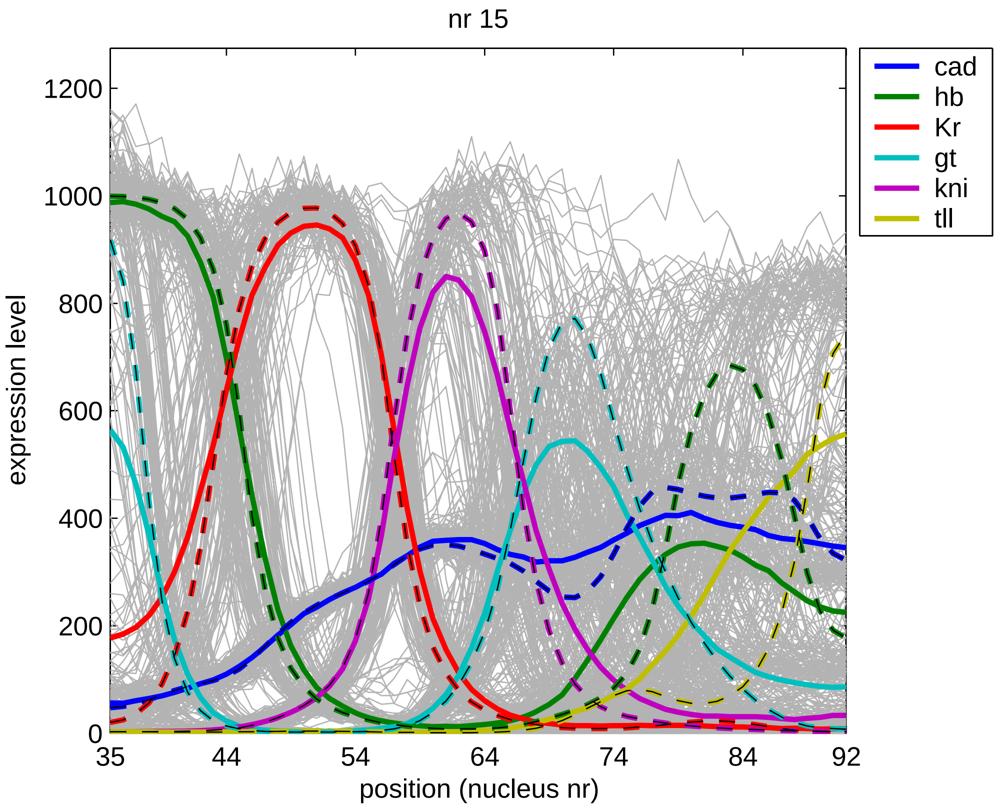

nr 15
cad
hb
Kr
gt
kni
tll
expression level
0
200
400
600
800
1000
1200
35
44
54
64
74
84
92
position (nucleus nr)

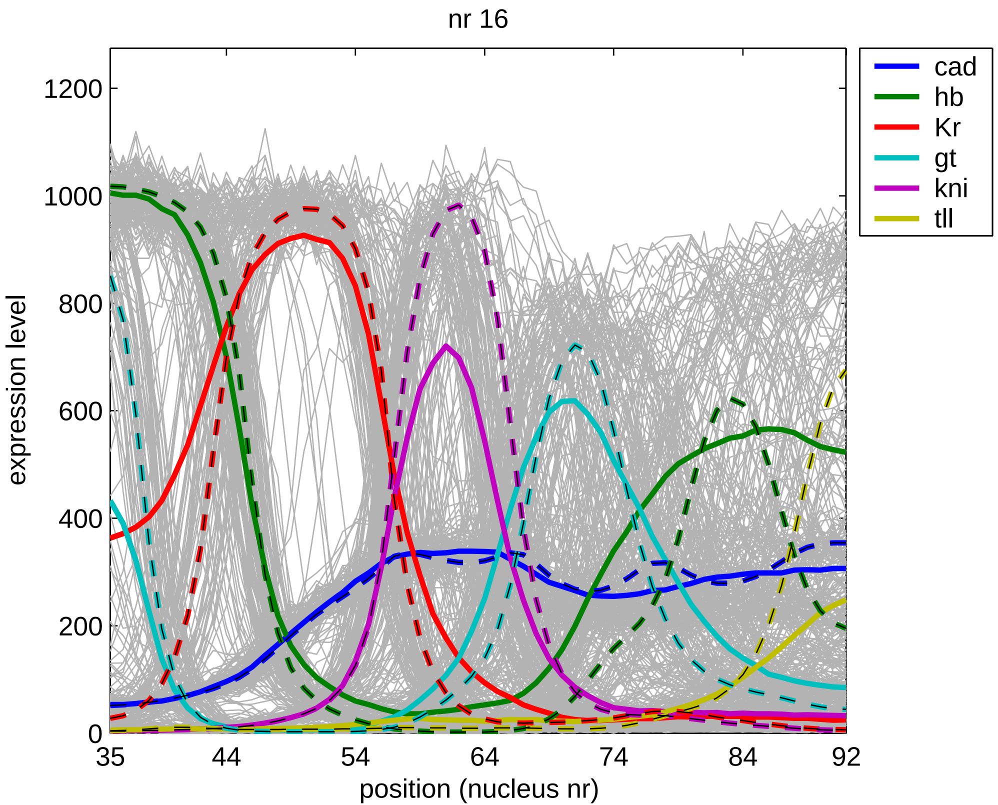

nr 16
cad
hb
Kr
gt
kni
tll
expression level
1200
1000
800
600
400
200
0
35
44
54
64
74
84
92
position (nucleus nr)

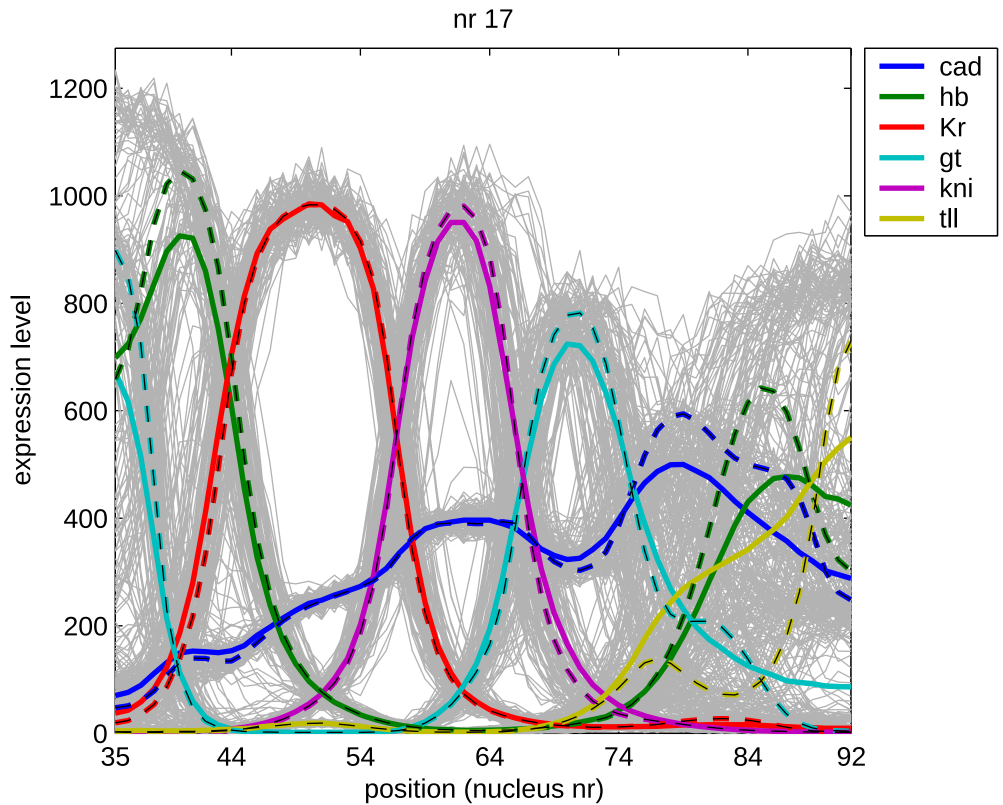
nr 17
expression level
position (nucleus nr)
0
200
400
600
800
1000
1200
35
44
54
64
74
84
92
cad
hb
Kr
gt
kni
tll

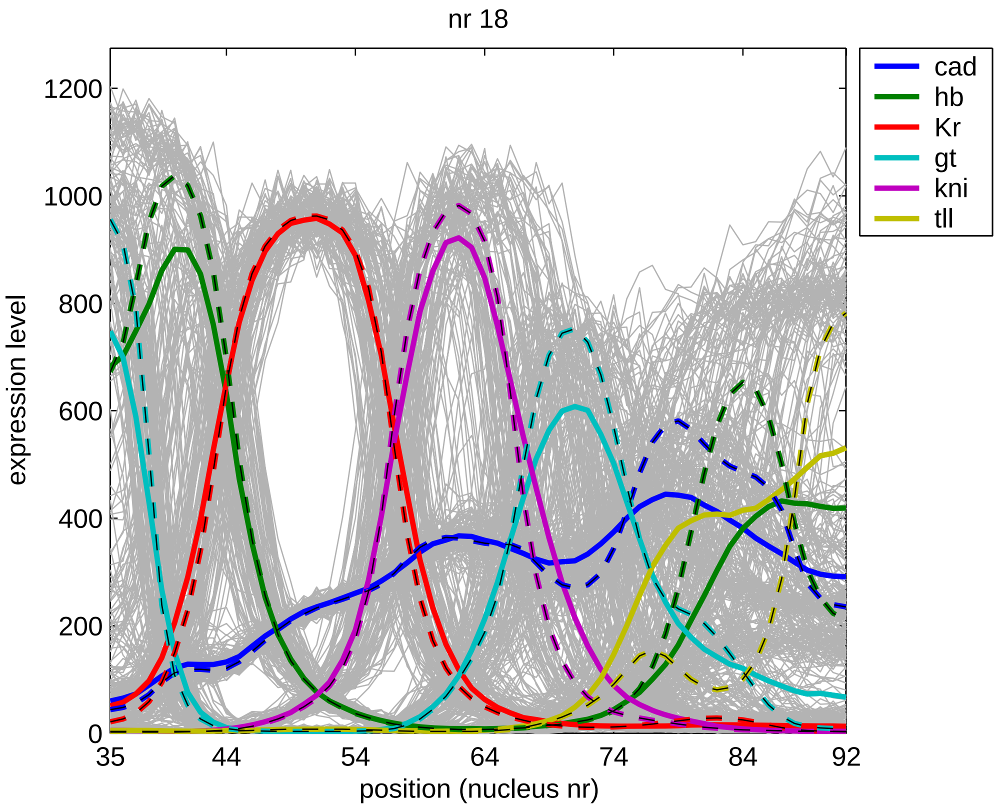

nr 18
1200
1000
800
600
400
200
0
35
44
54
64
74
84
92
expression level
position (nucleus nr)
cad
hb
Kr
gt
kni
tll

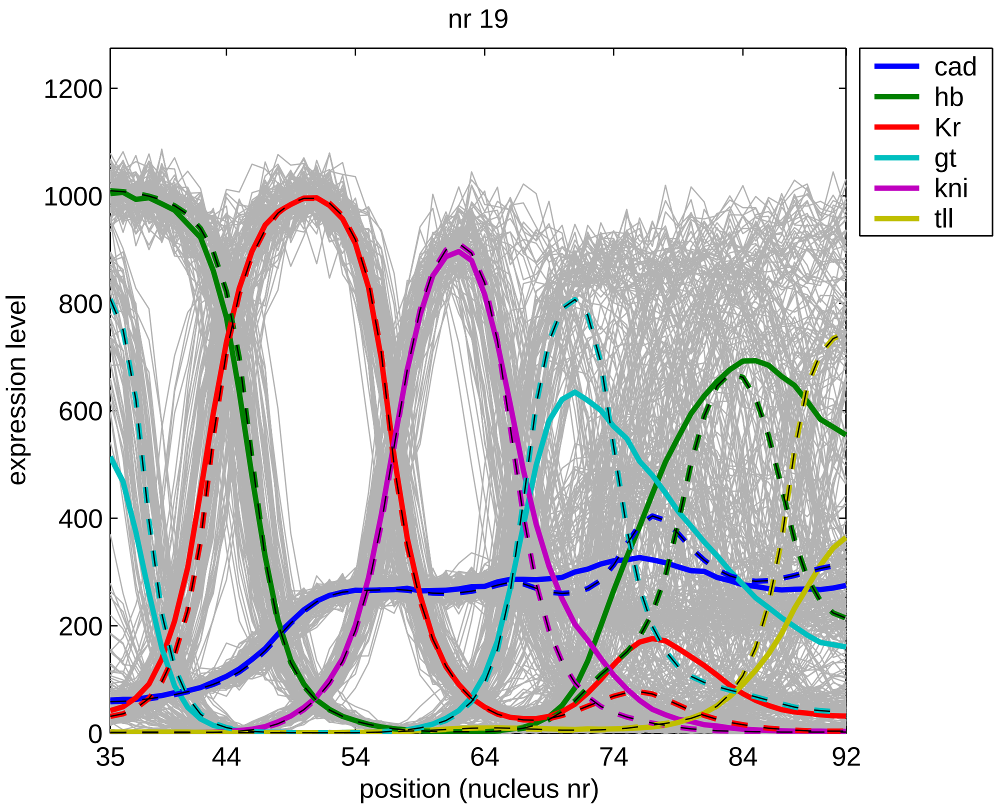

nr 19
expression level
position (nucleus nr)
0
200
400
600
800
1000
1200
35
44
54
64
74
84
92
cad
hb
Kr
gt
kni
tll

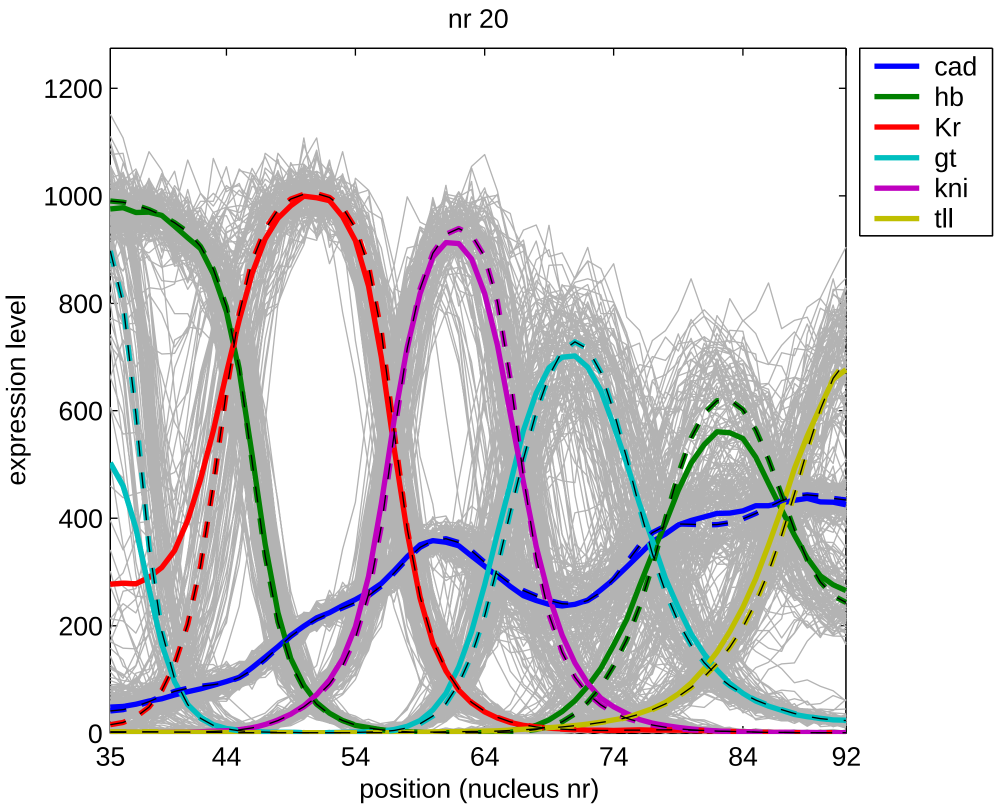
nr 20
expression level
position (nucleus nr)
0
200
400
600
800
1000
1200
35
44
54
64
74
84
92
cad
hb
Kr
gt
kni
tll

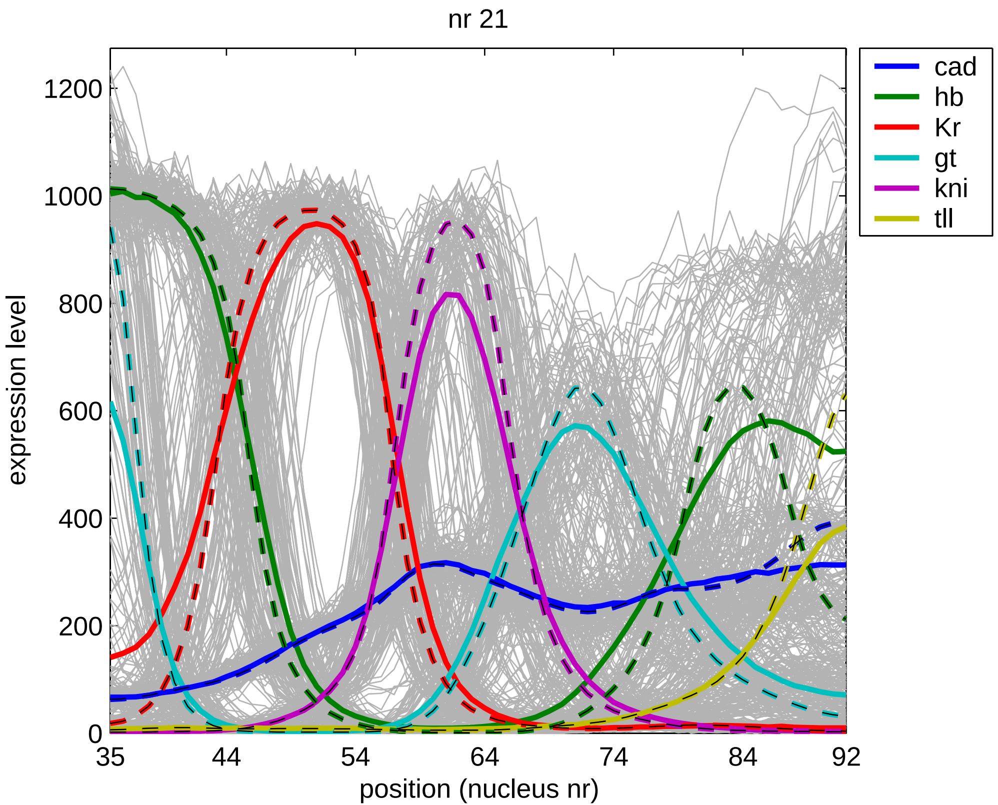

nr 21
expression level
position (nucleus nr)
0
200
400
600
800
1000
1200
35
44
54
64
74
84
92
cad
hb
Kr
gt
kni
tll

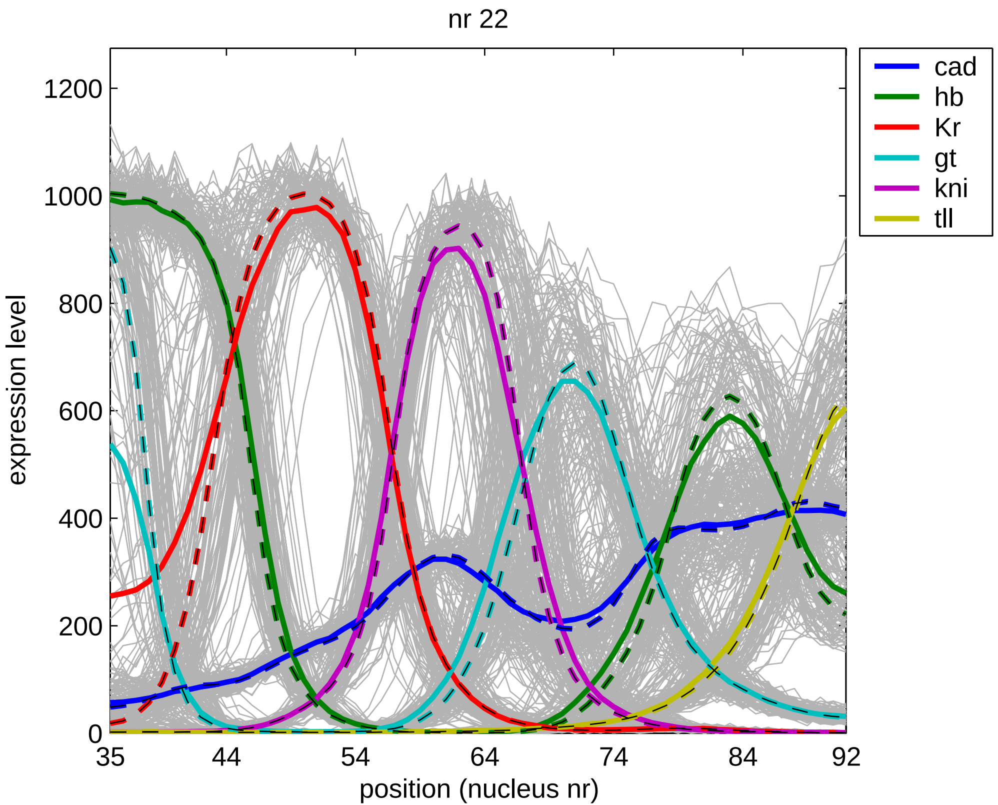
nr 22
expression level
1200
1000
800
600
400
200
0
35
44
54
64
74
84
92
position (nucleus nr)
cad
hb
Kr
gt
kni
tll

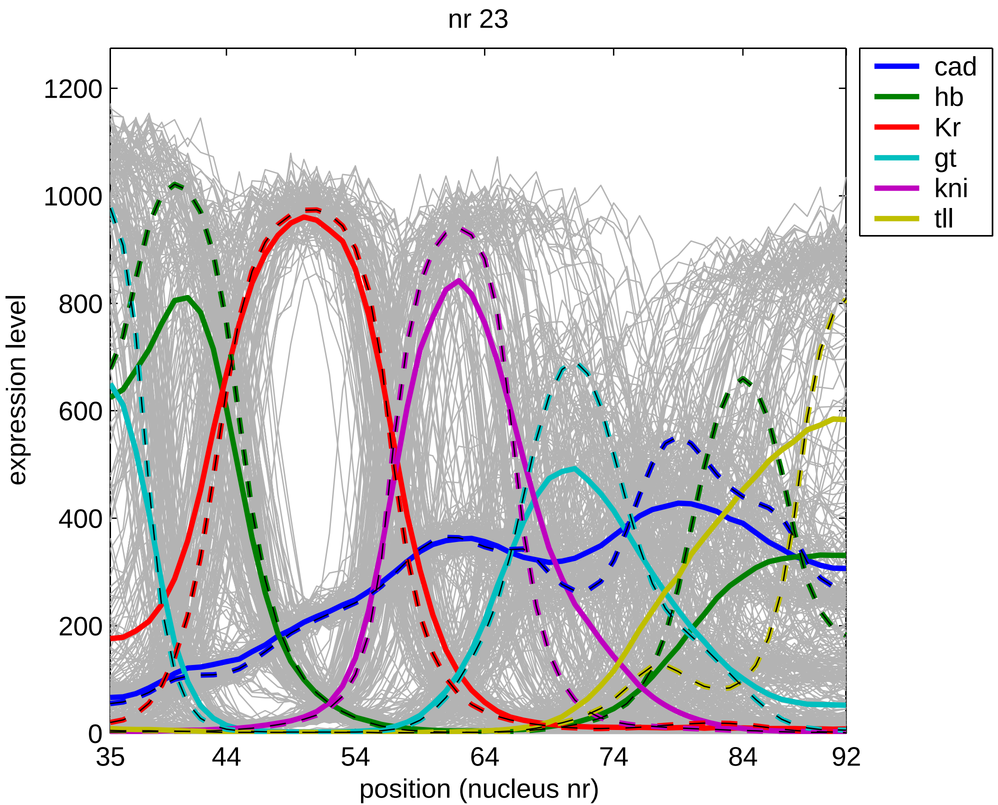

nr 23
cad
hb
Kr
gt
kni
tll
expression level
position (nucleus nr)
0
200
400
600
800
1000
1200
35
44
54
64
74
84
92

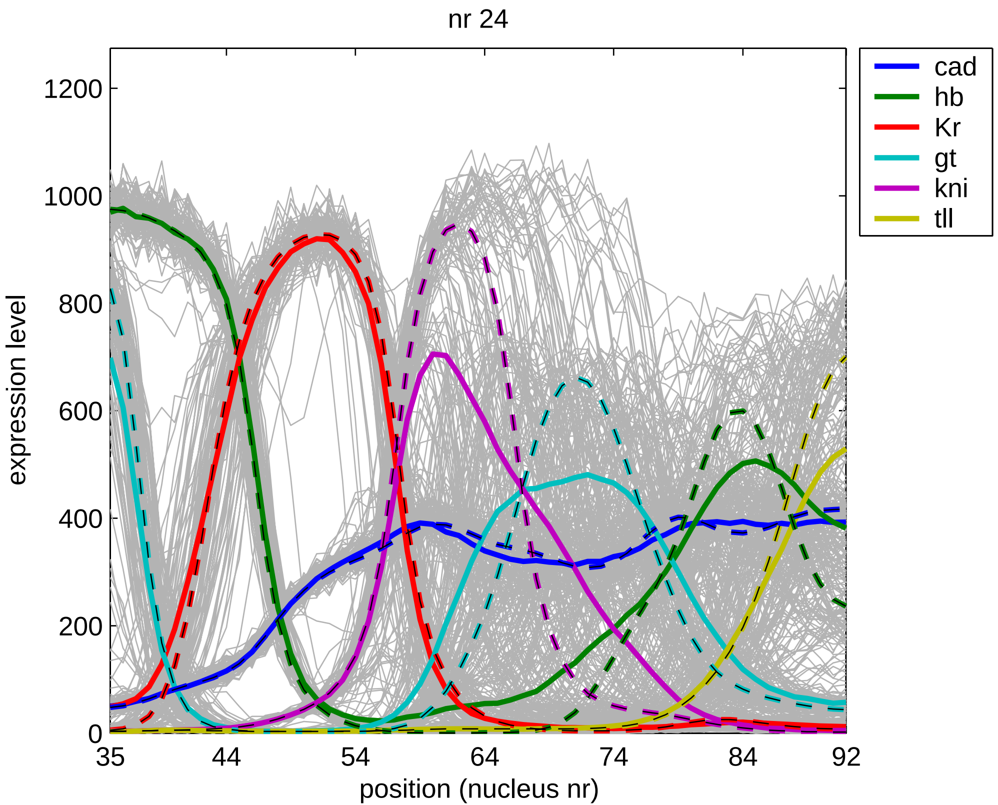

nr 24
expression level
1200
1000
800
600
400
200
0
35
44
54
64
74
84
92
position (nucleus nr)
cad
hb
Kr
gt
kni
tll

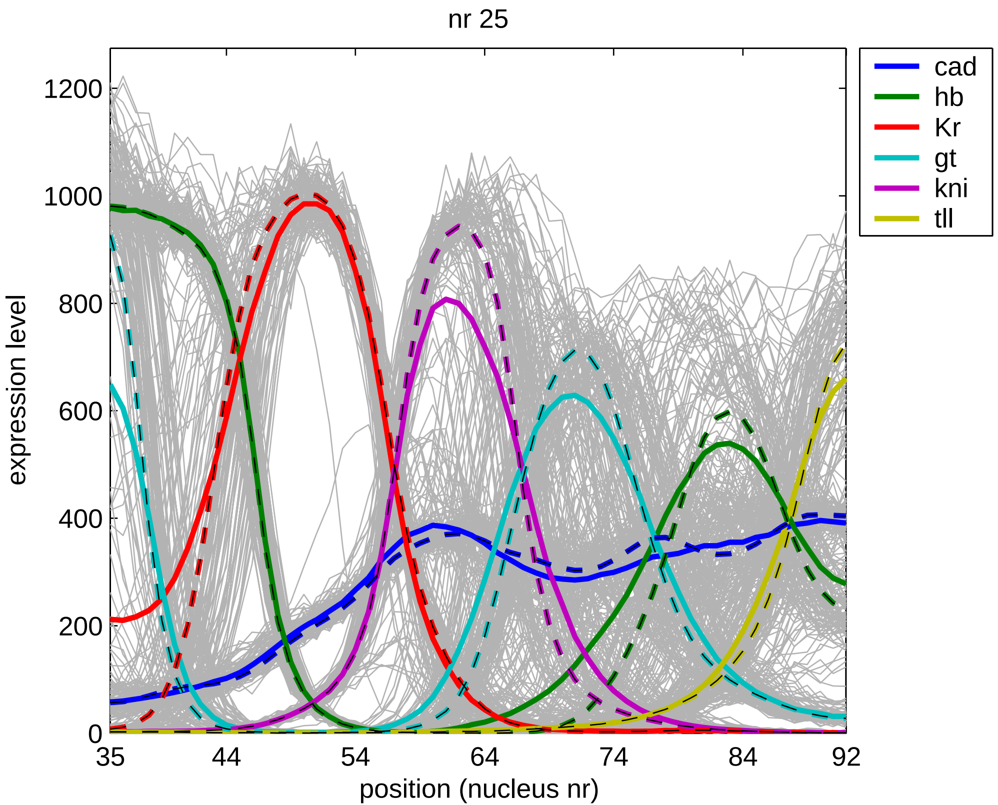
nr 25
expression level
position (nucleus nr)
0
200
400
600
800
1000
1200
35
44
54
64
74
84
92
cad
hb
Kr
gt
kni
tll

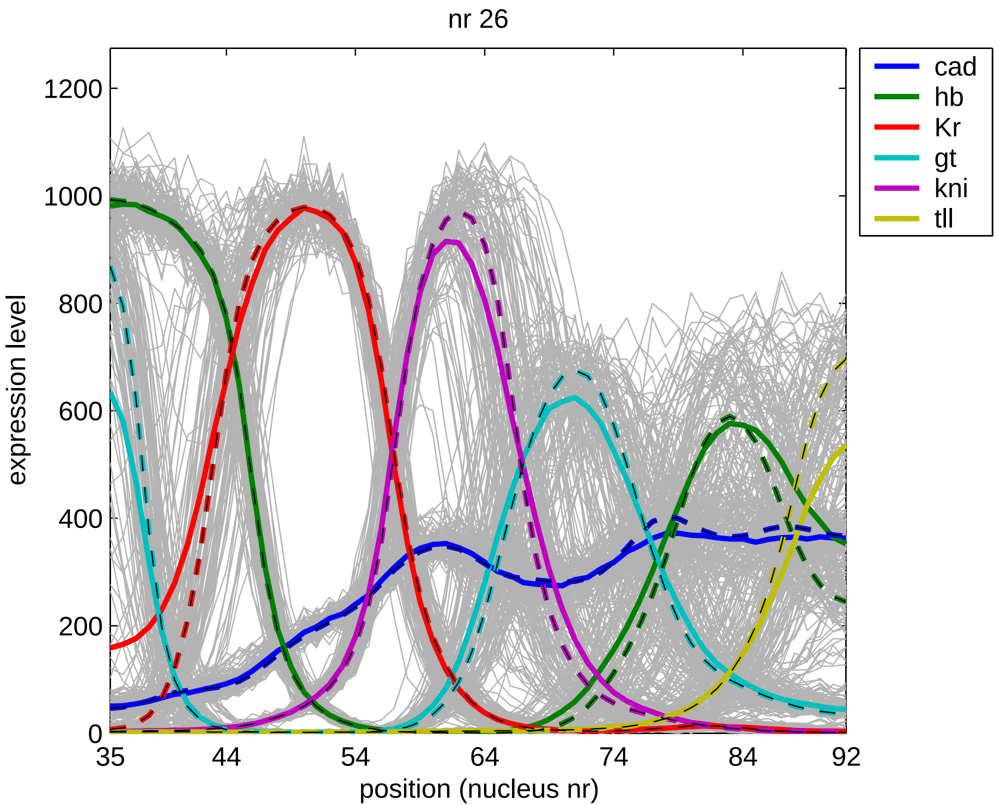

nr 26
expression level
position (nucleus nr)
0
200
400
600
800
1000
1200
35
44
54
64
74
84
92
cad
hb
Kr
gt
kni
tll

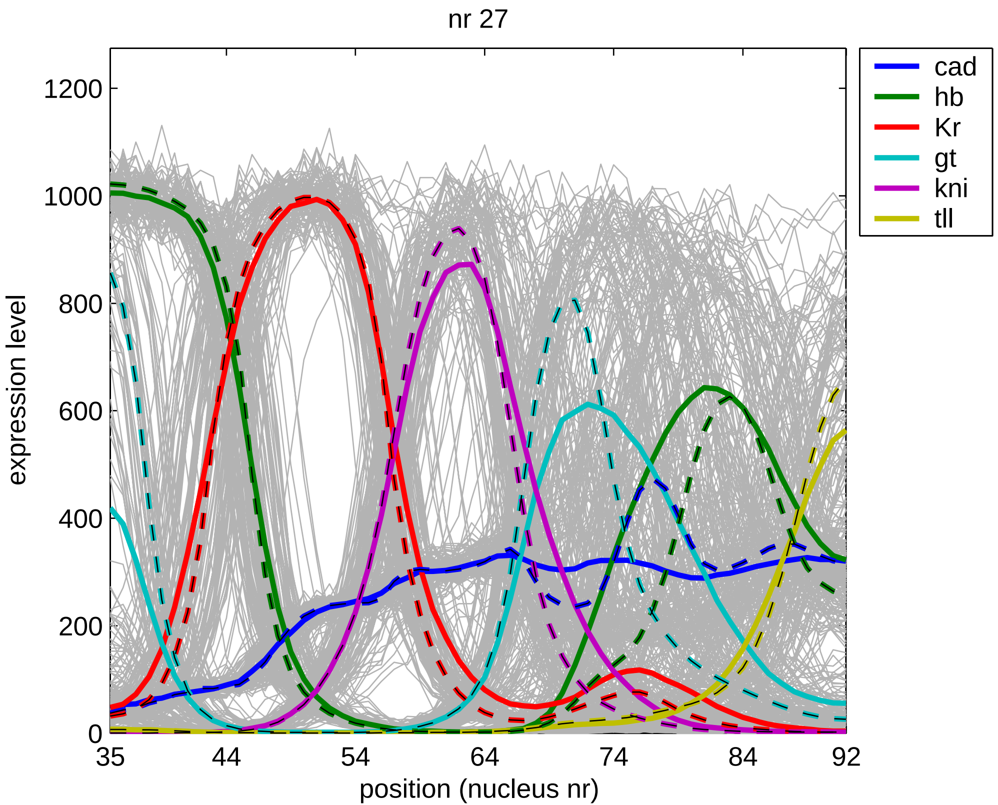

nr 27
expression level
position (nucleus nr)
0
200
400
600
800
1000
1200
35
44
54
64
74
84
92
cad
hb
Kr
gt
kni
tll

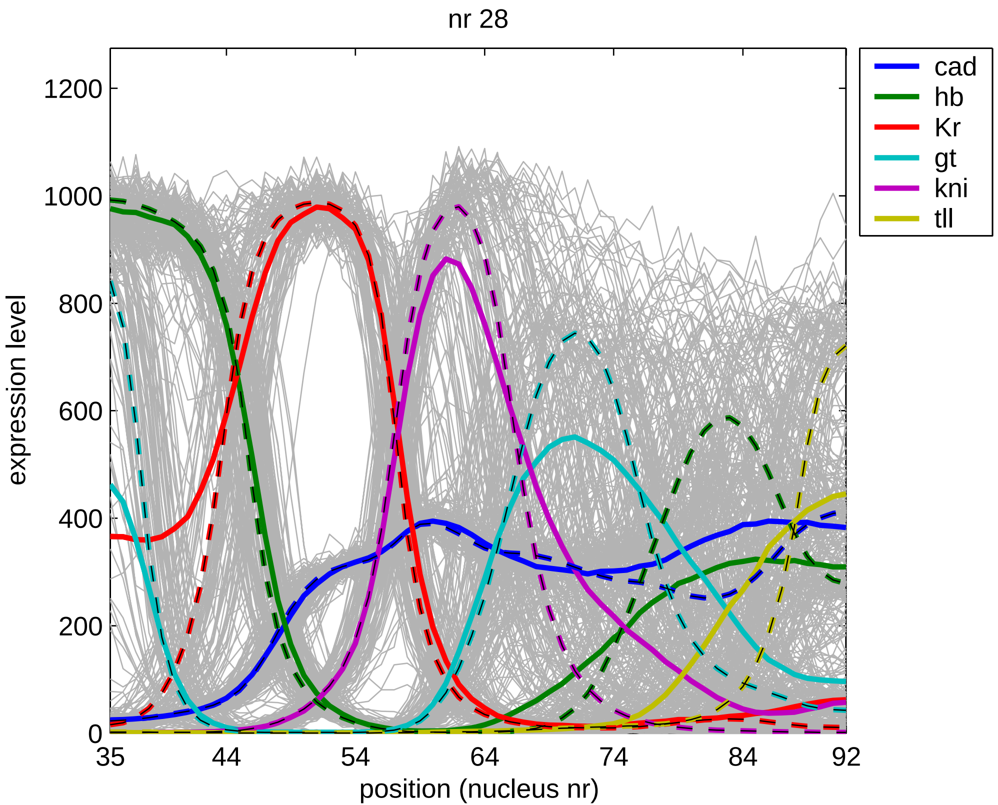

nr 28
expression level
position (nucleus nr)
0
200
400
600
800
1000
1200
35
44
54
64
74
84
92
cad
hb
Kr
gt
kni
tll

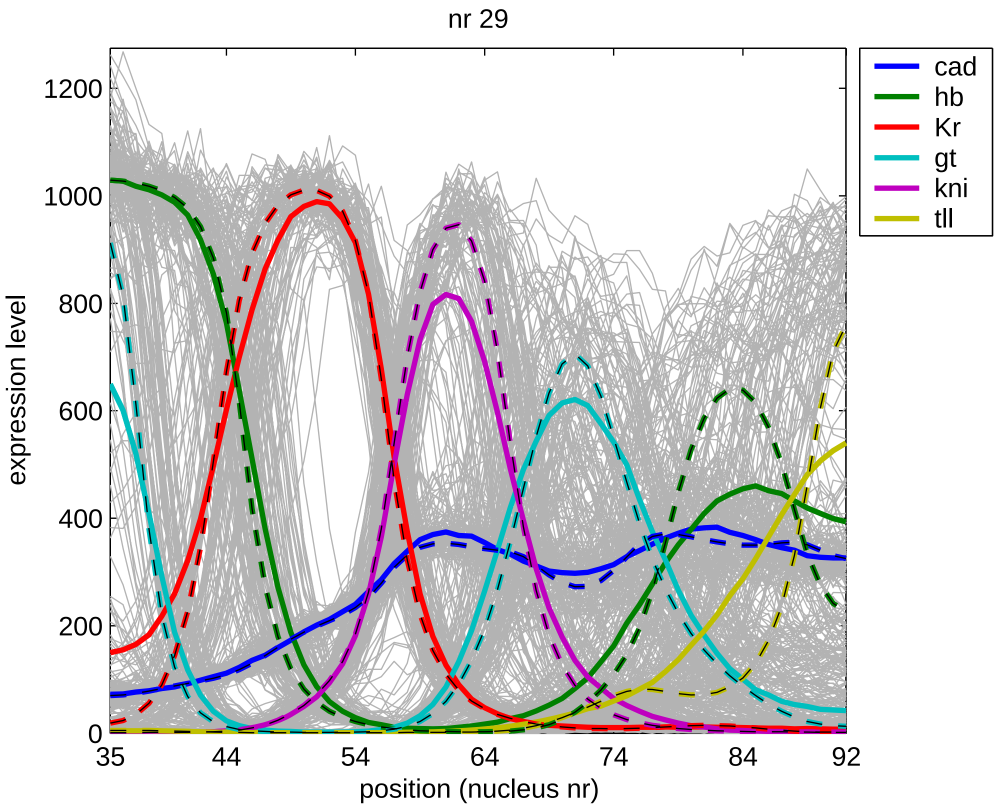

nr 29
cad
hb
Kr
gt
kni
tll
expression level
0
200
400
600
800
1000
1200
35
44
54
64
74
84
92
position (nucleus nr)

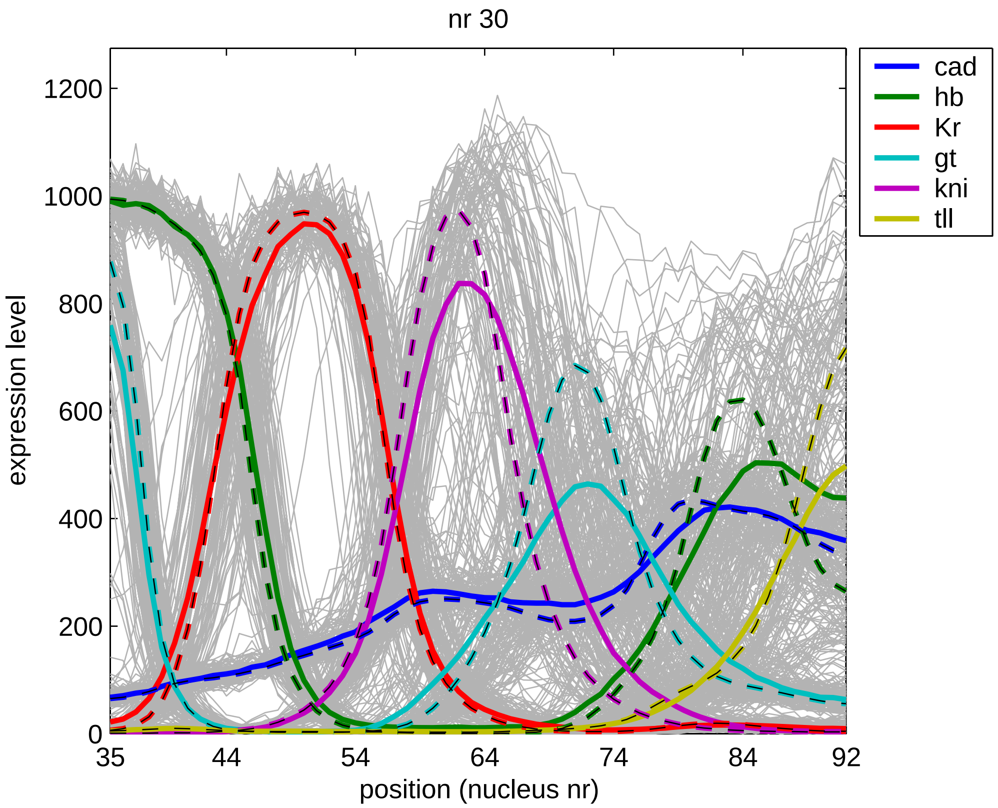
nr 30
expression level
position (nucleus nr)
0
200
400
600
800
1000
1200
35
44
54
64
74
84
92
cad
hb
Kr
gt
kni
tll

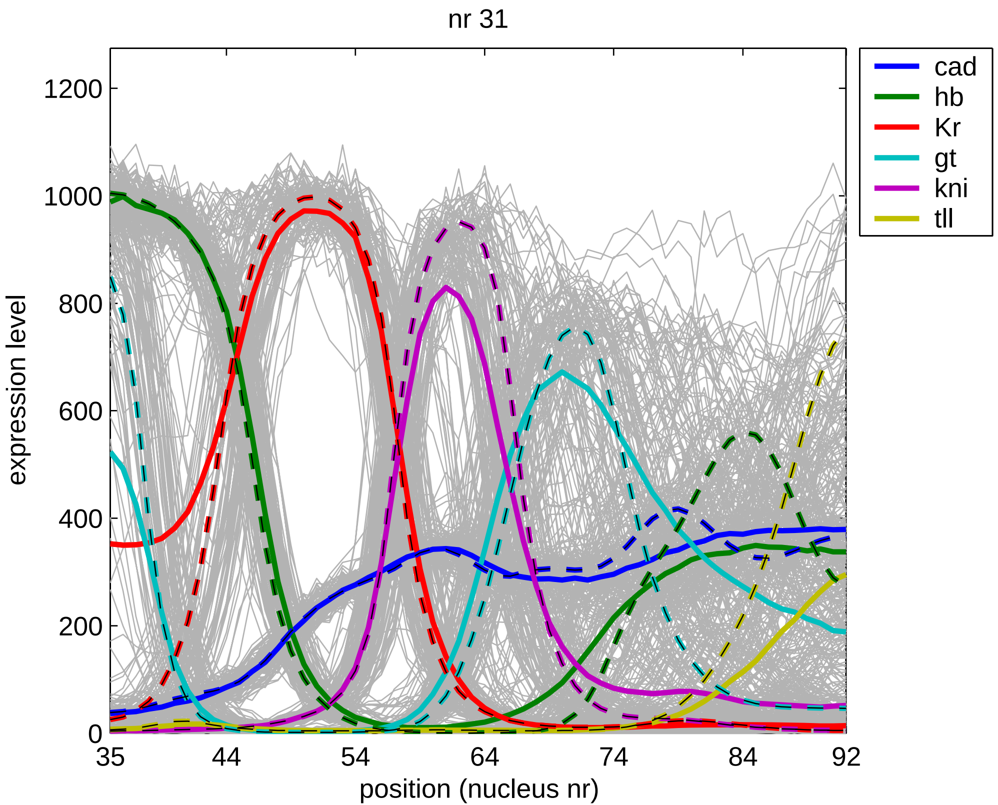
nr 31
expression level
position (nucleus nr)
0
200
400
600
800
1000
1200
35
44
54
64
74
84
92
cad
hb
Kr
gt
kni
tll

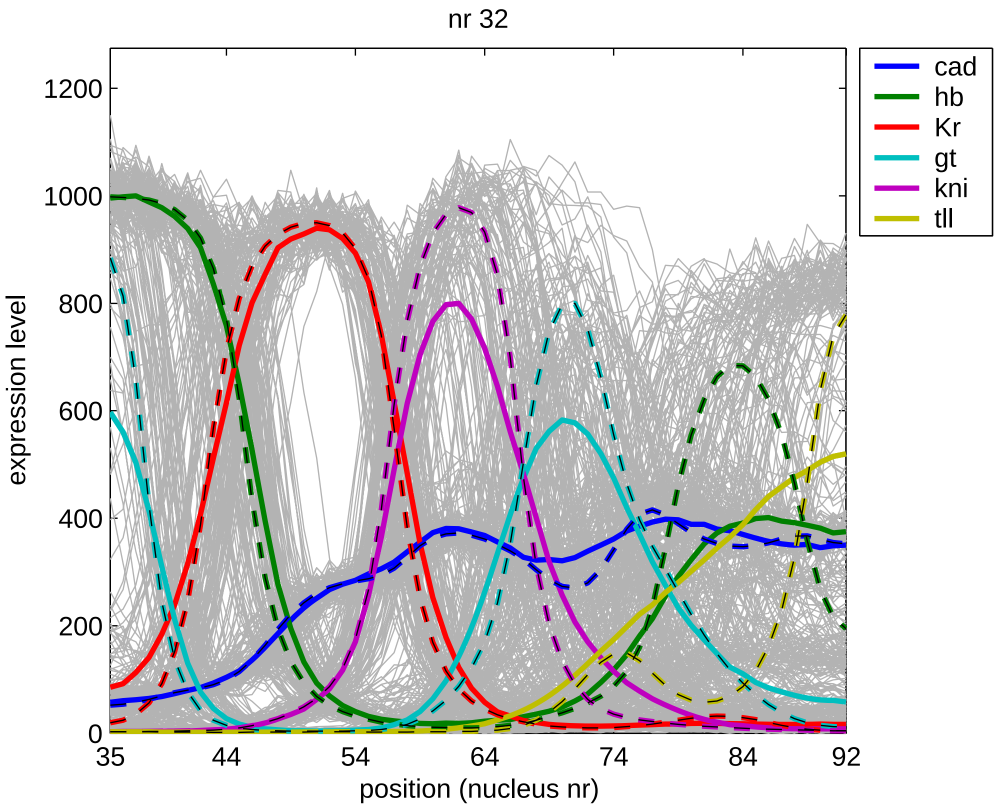
nr 32
expression level
1200
1000
800
600
400
200
0
35
44
54
64
74
84
92
position (nucleus nr)
cad
hb
Kr
gt
kni
tll

nr 33

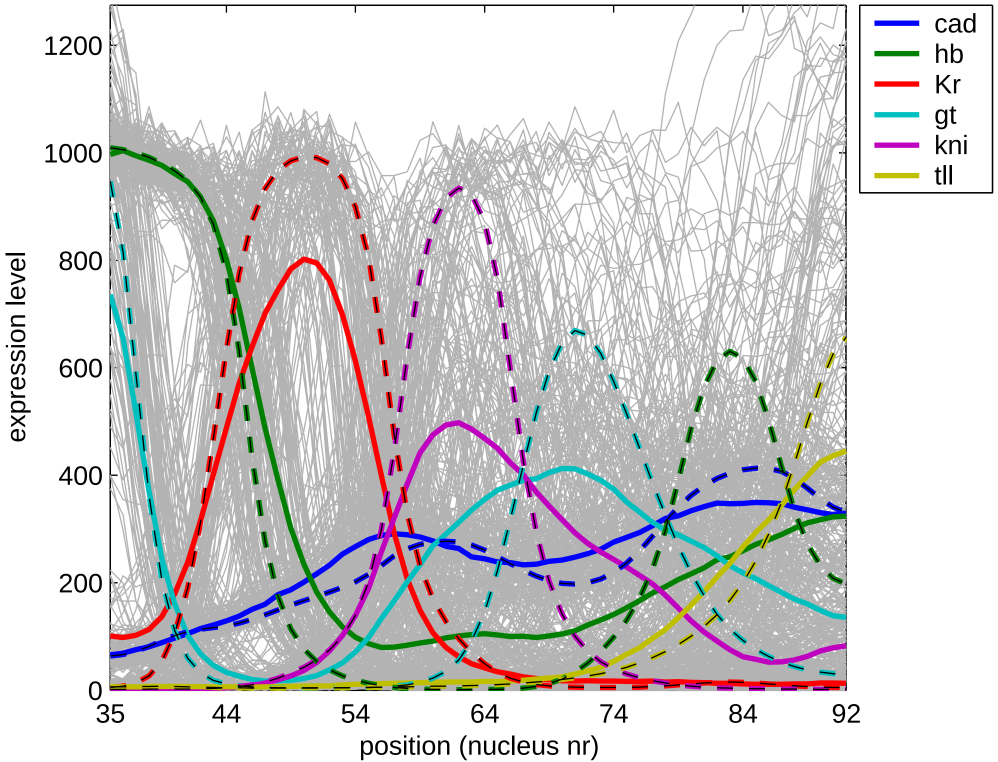

cad
hb
Kr
gt
kni
tll
expression level
position (nucleus nr)
0
200
400
600
800
1000
1200
35
44
54
64
74
84
92

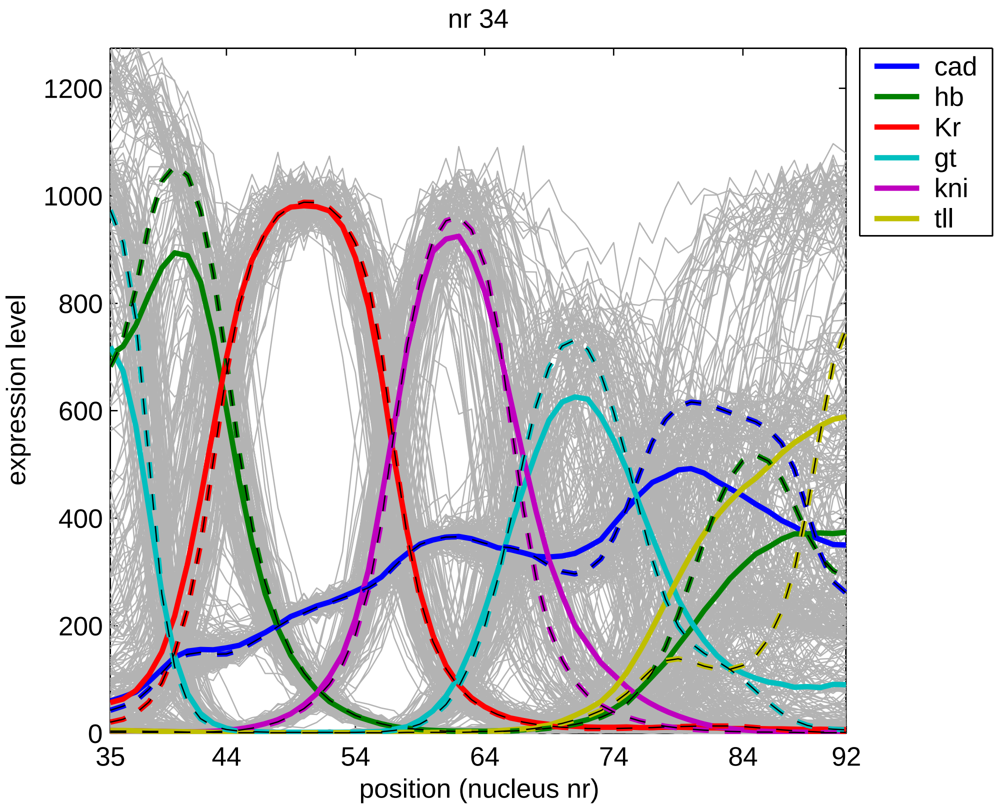

nr 34
expression level
0
200
400
600
800
1000
1200
35
44
54
64
74
84
92
position (nucleus nr)
cad
hb
Kr
gt
kni
tll

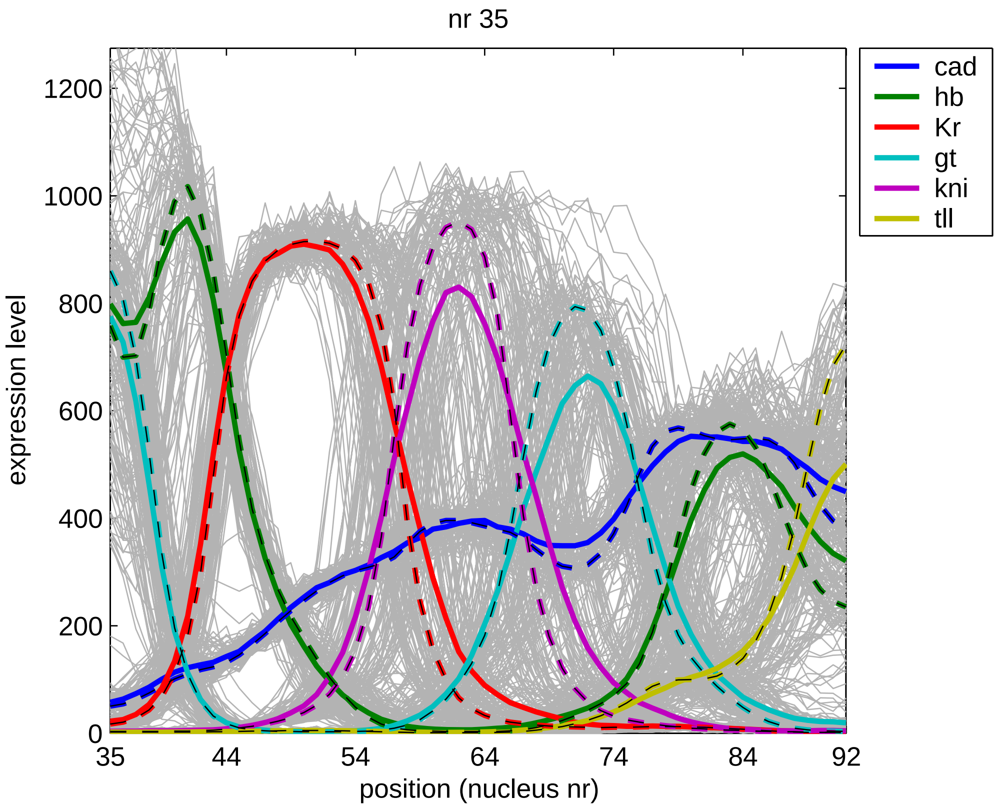

nr 35
expression level
position (nucleus nr)
0
200
400
600
800
1000
1200
35
44
54
64
74
84
92
cad
hb
Kr
gt
kni
tll

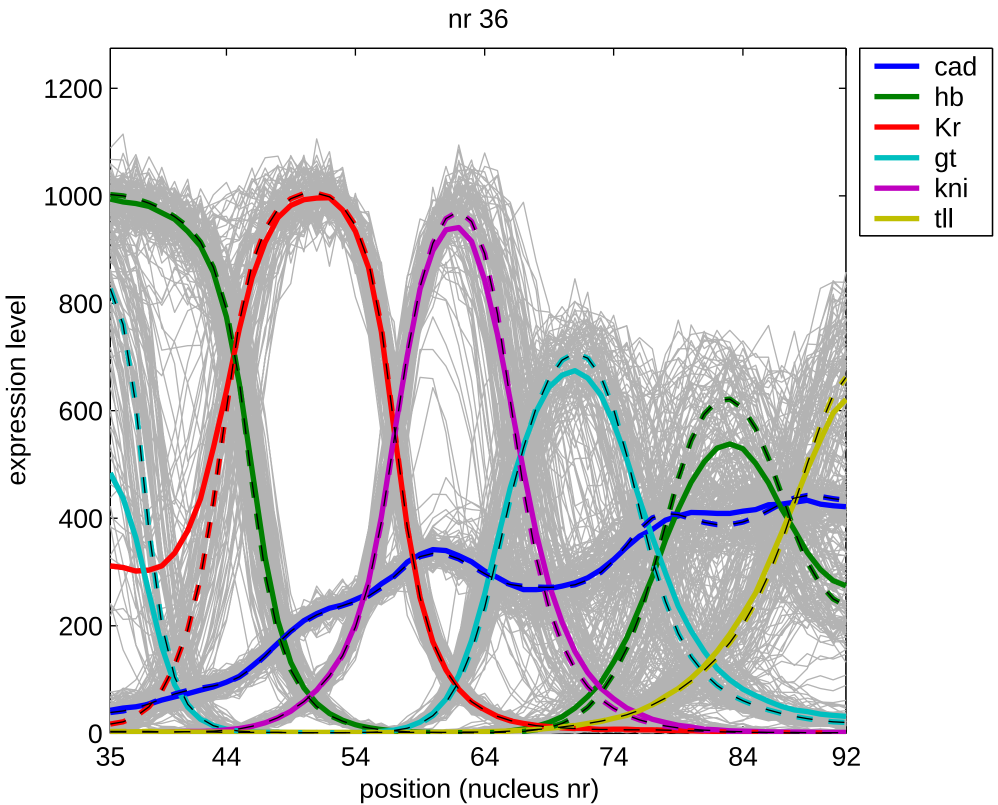
nr 36
expression level
position (nucleus nr)
0
200
400
600
800
1000
1200
35
44
54
64
74
84
92
cad
hb
Kr
gt
kni
tll

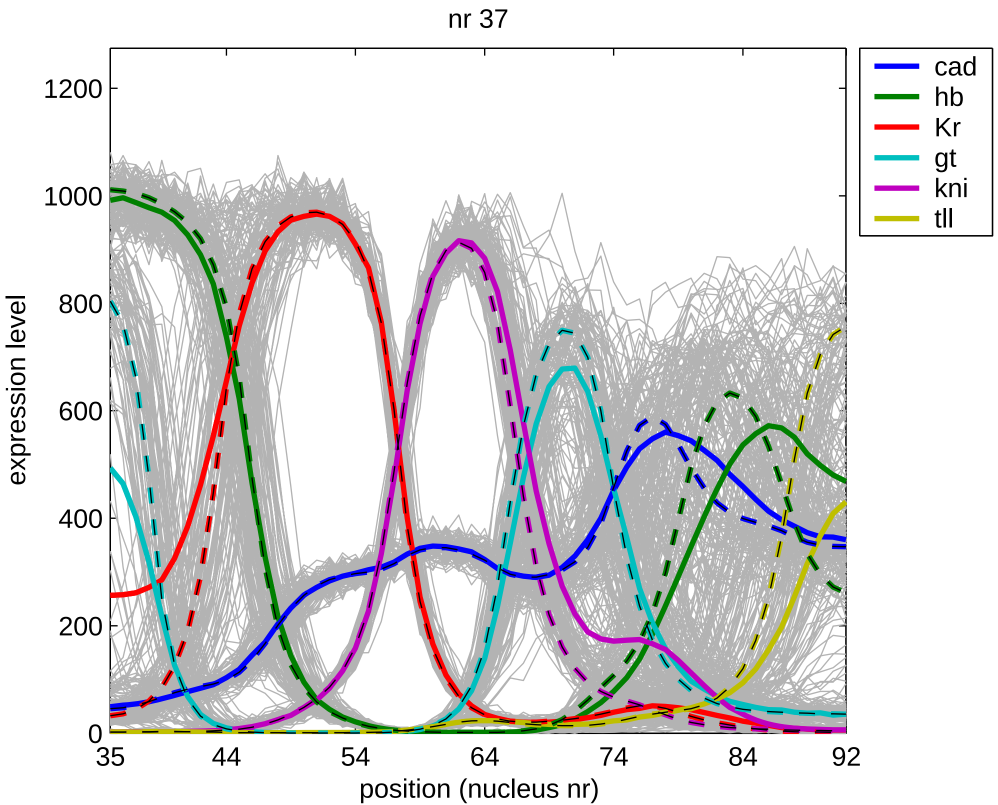
nr 37
expression level
position (nucleus nr)
0
200
400
600
800
1000
1200
35
44
54
64
74
84
92
cad
hb
Kr
gt
kni
tll

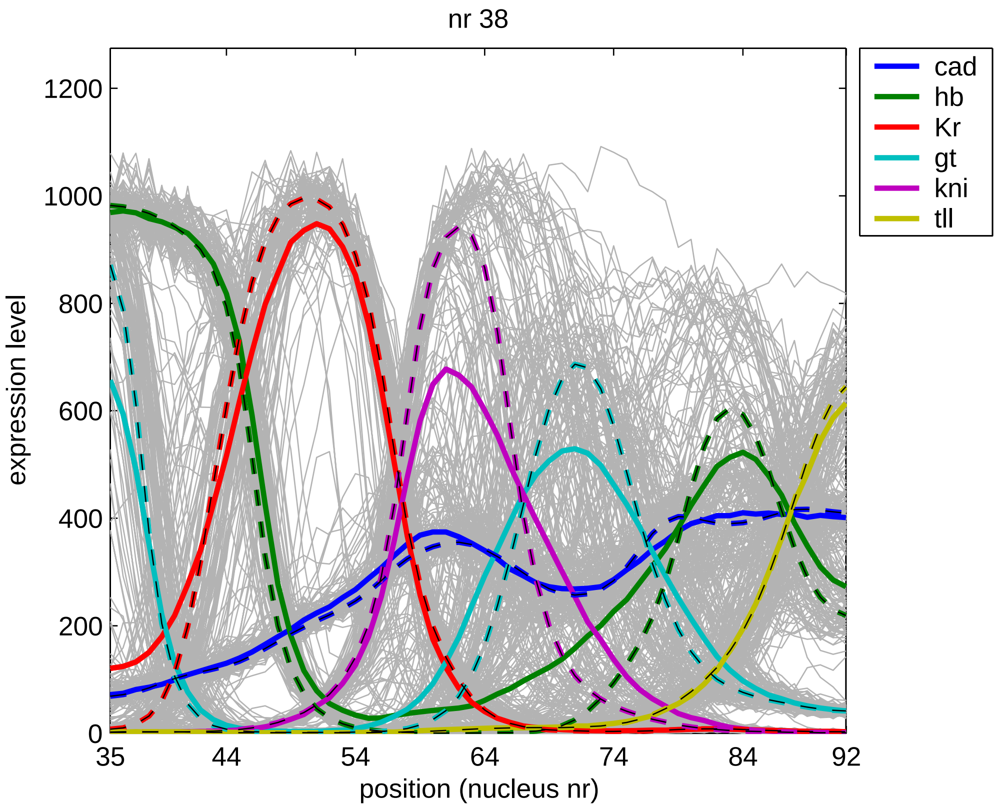

nr 38
expression level
position (nucleus nr)
0
200
400
600
800
1000
1200
35
44
54
64
74
84
92
cad
hb
Kr
gt
kni
tll

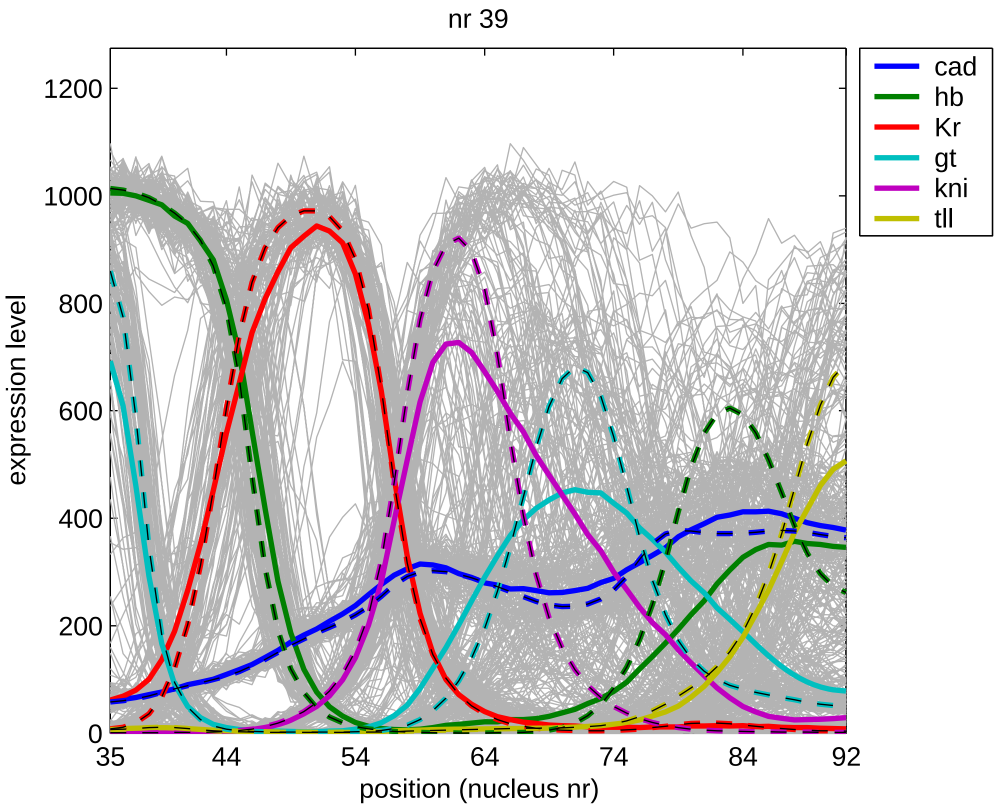
nr 39
expression level
position (nucleus nr)
0
200
400
600
800
1000
1200
35
44
54
64
74
84
92
cad
hb
Kr
gt
kni
tll

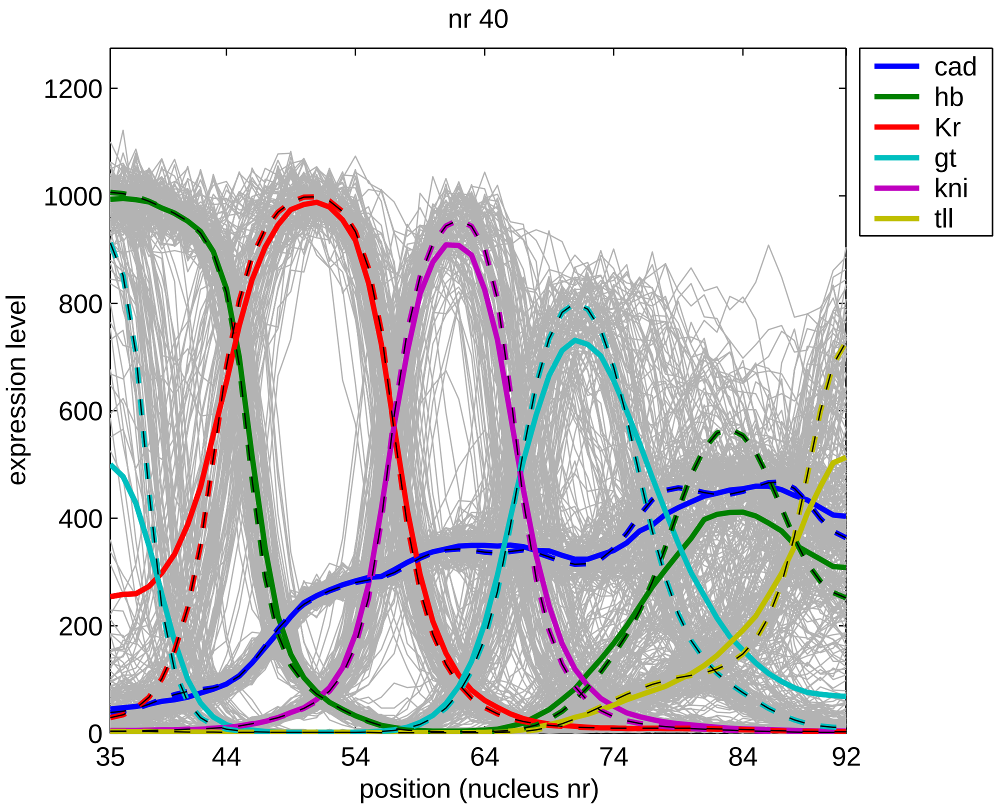
nr 40
expression level
0
200
400
600
800
1000
1200
35
44
54
64
74
84
92
position (nucleus nr)
cad
hb
Kr
gt
kni
tll

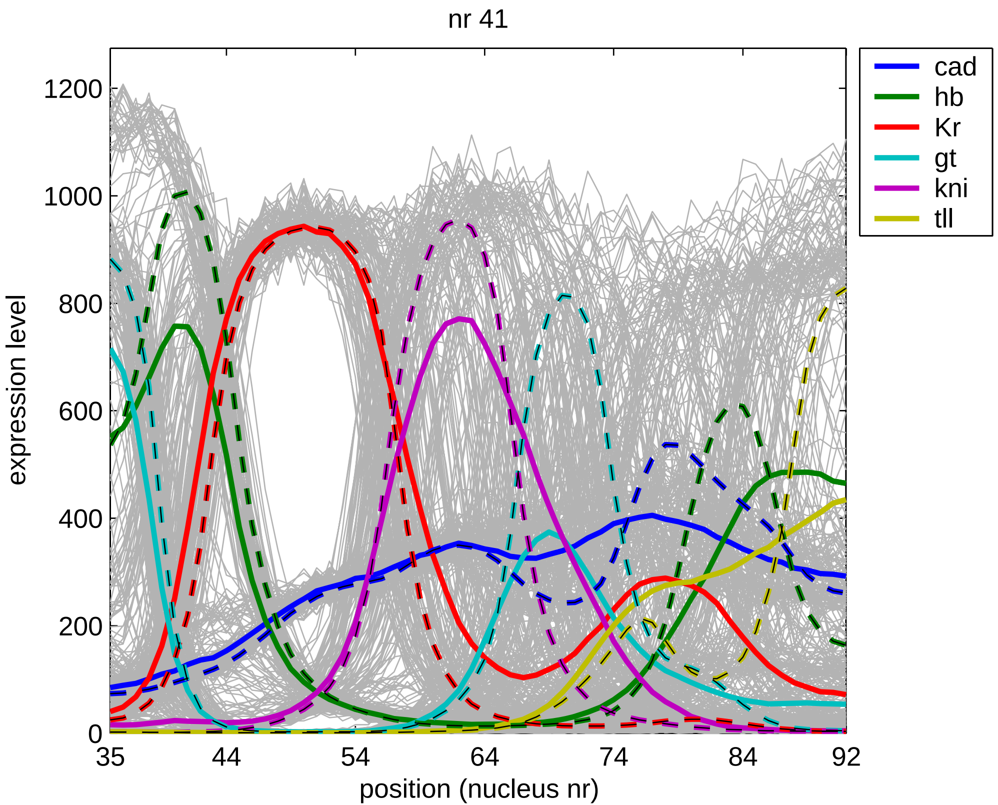

nr 41
expression level
position (nucleus nr)
0
200
400
600
800
1000
1200
35
44
54
64
74
84
92
cad
hb
Kr
gt
kni
tll

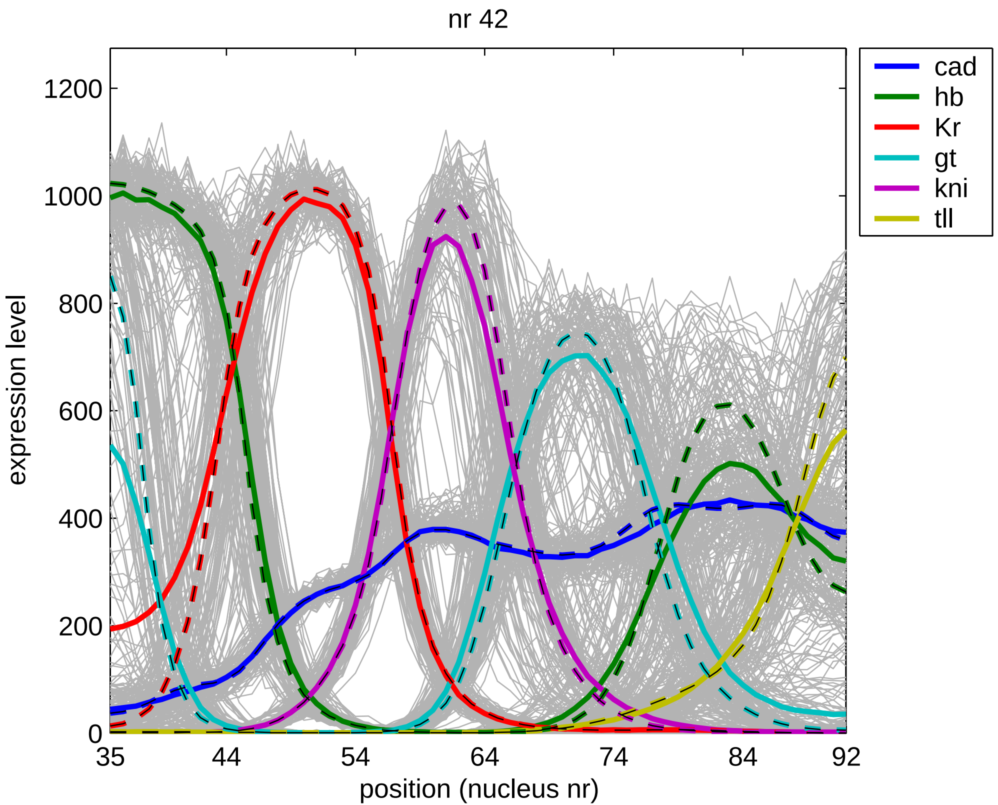
nr 42
expression level
1200
1000
800
600
400
200
0
35
44
54
64
74
84
92
position (nucleus nr)
cad
hb
Kr
gt
kni
tll

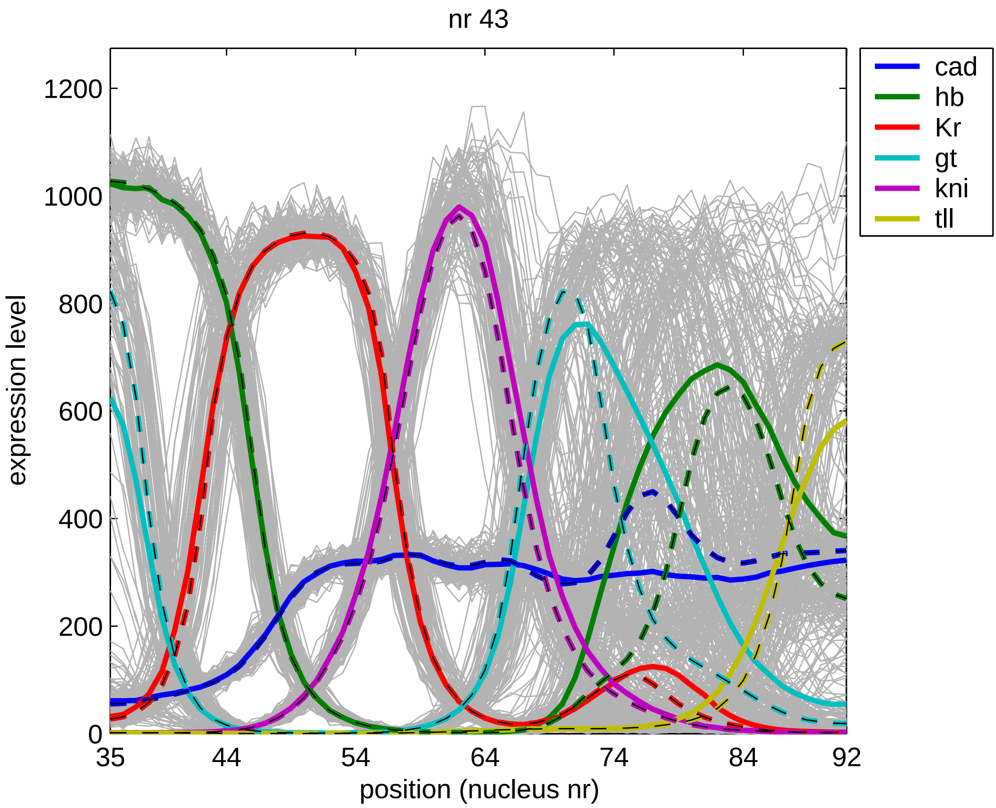

nr 43
1200
1000
800
600
400
200
0
expression level
35
44
54
64
74
84
92
position (nucleus nr)
cad
hb
Kr
gt
kni
tll

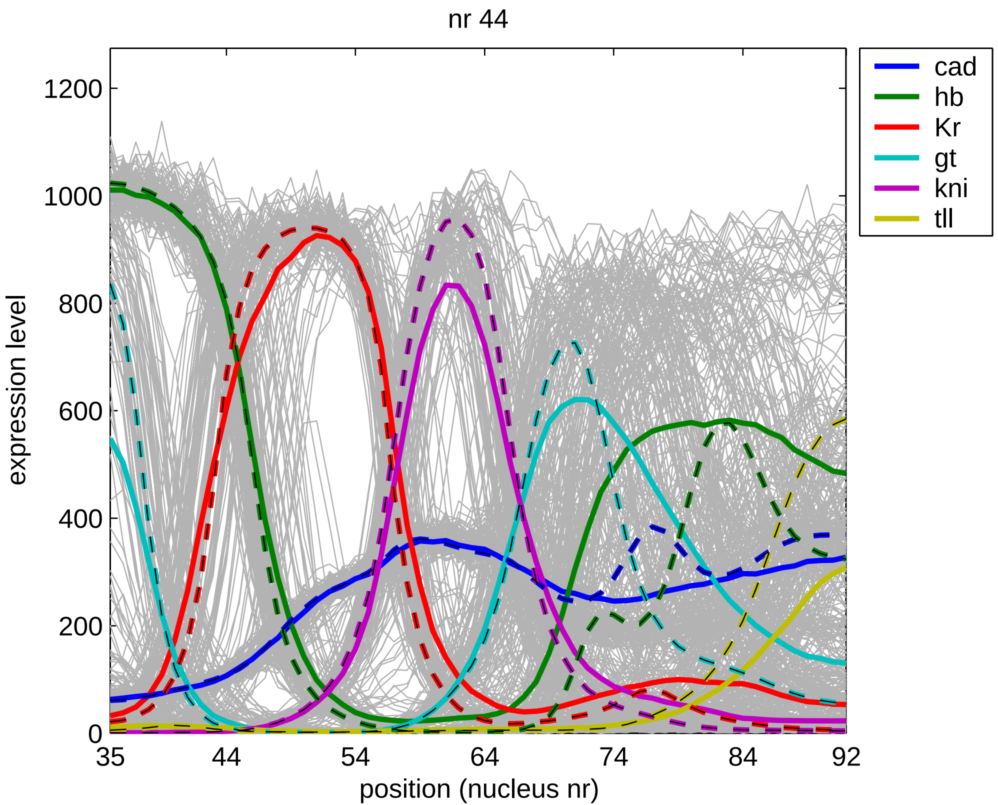

nr 44
expression level
position (nucleus nr)
0
200
400
600
800
1000
1200
35
44
54
64
74
84
92
cad
hb
Kr
gt
kni
tll

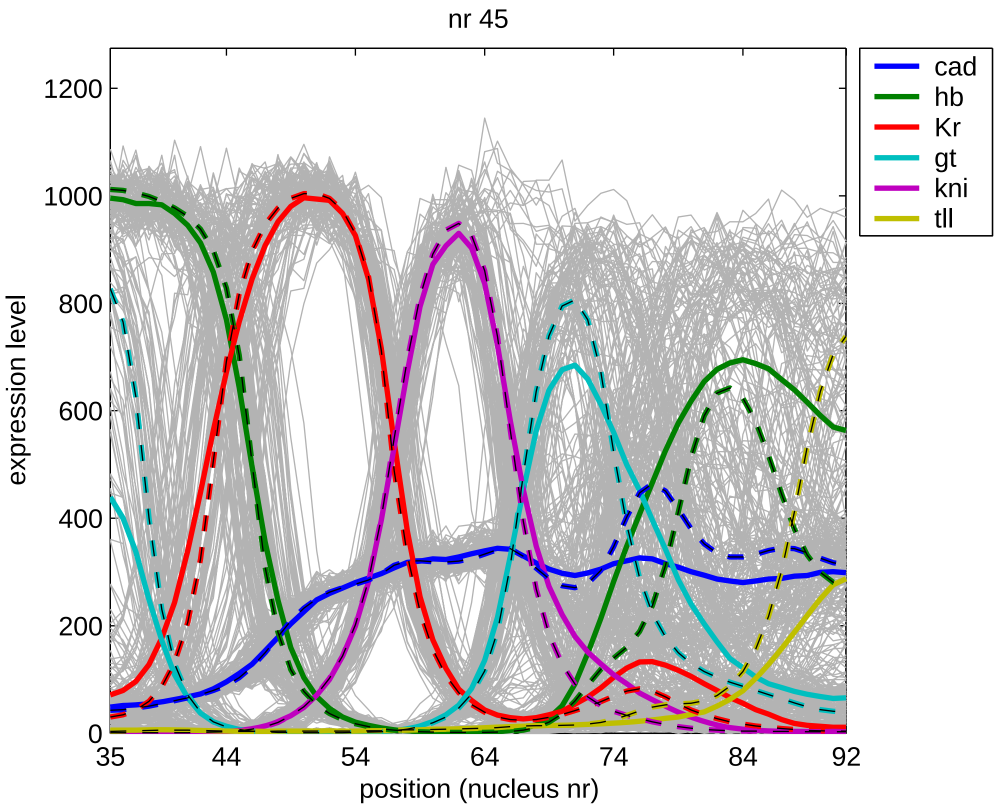
nr 45
expression level
position (nucleus nr)
0
200
400
600
800
1000
1200
35
44
54
64
74
84
92
cad
hb
Kr
gt
kni
tll

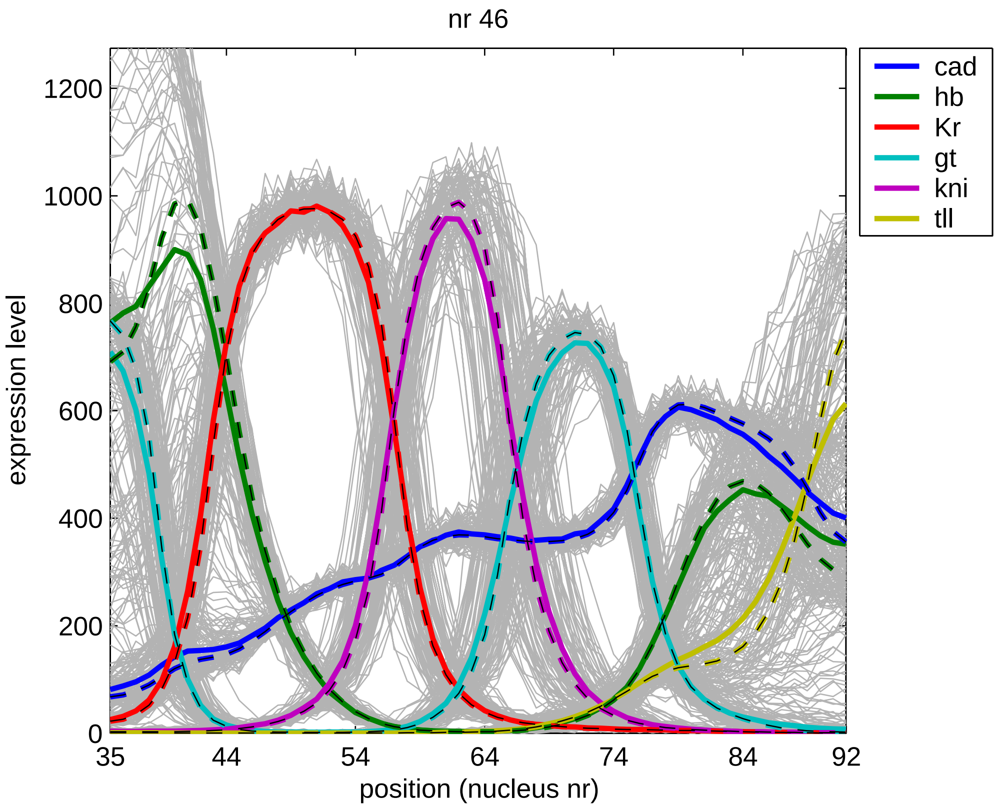
nr 46
expression level
position (nucleus nr)
0
200
400
600
800
1000
1200
35
44
54
64
74
84
92
cad
hb
Kr
gt
kni
tll

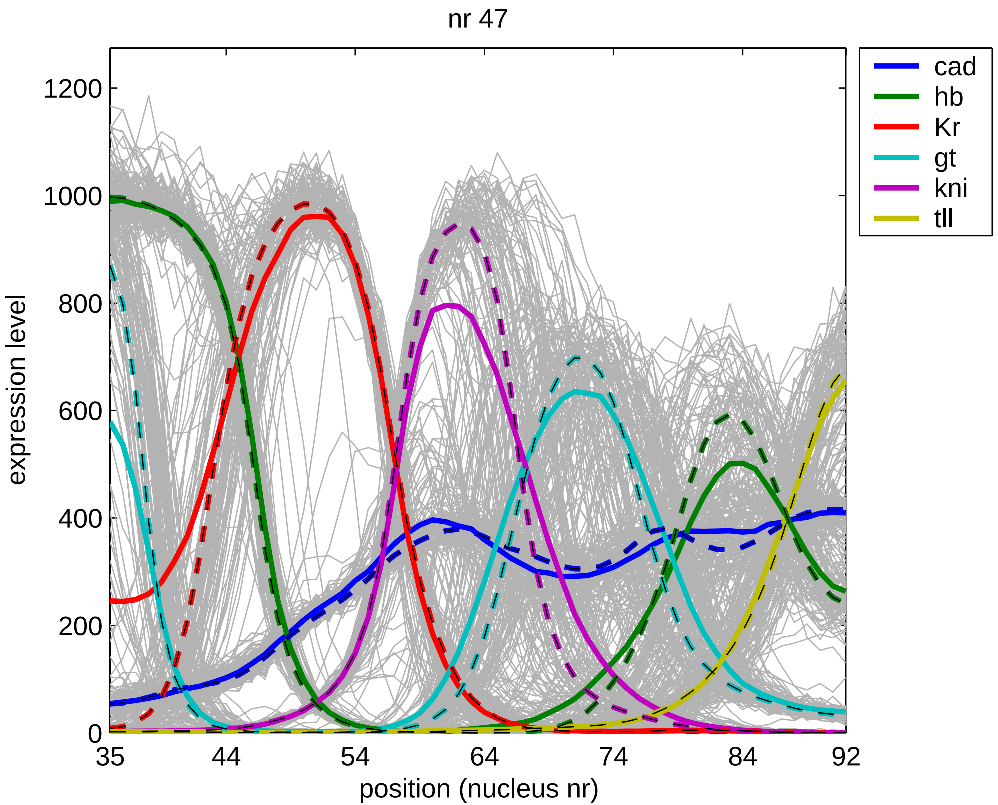
nr 47
expression level
position (nucleus nr)
0
200
400
600
800
1000
1200
35
44
54
64
74
84
92
cad
hb
Kr
gt
kni
tll

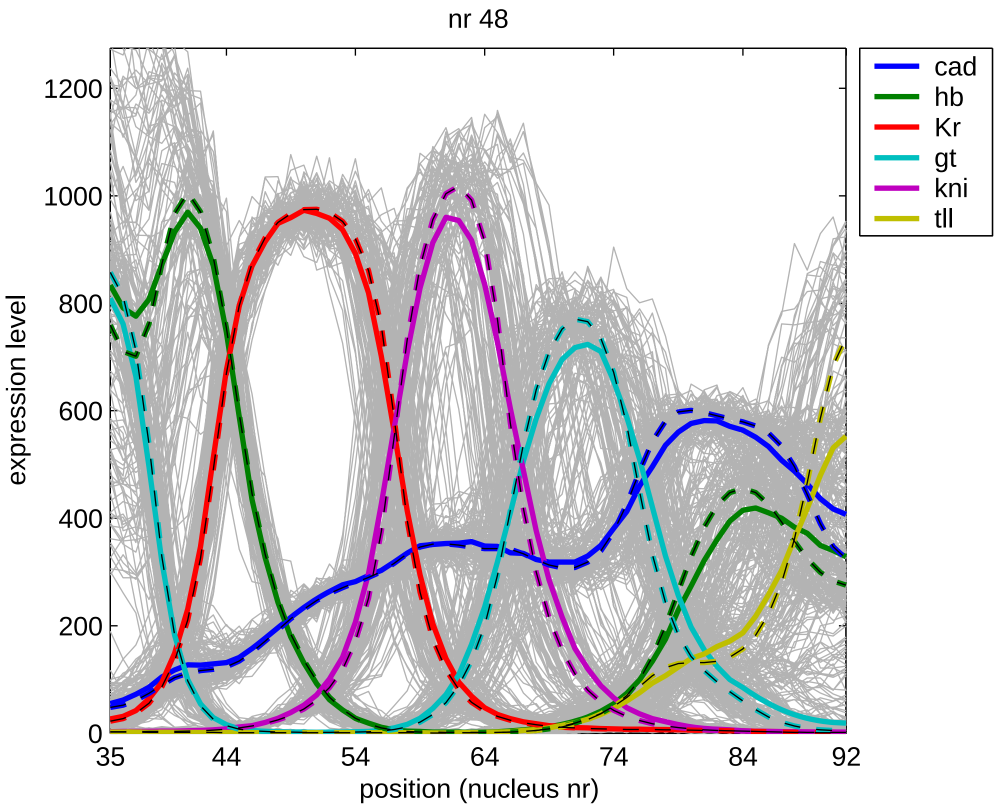

nr 48
cad
hb
Kr
gt
kni
tll
expression level
0
200
400
600
800
1000
1200
35
44
54
64
74
84
92
position (nucleus nr)

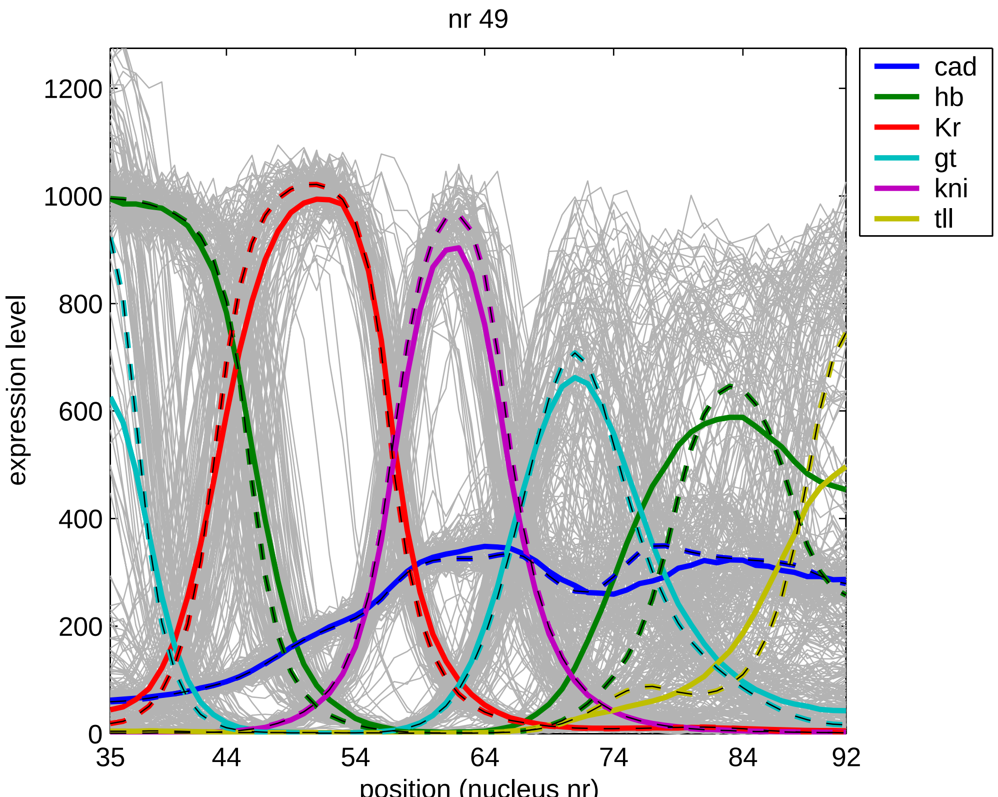

nr 49
expression level
position (nucleus nr)
0
200
400
600
800
1000
1200
35
44
54
64
74
84
92
cad
hb
Kr
gt
kni
tll

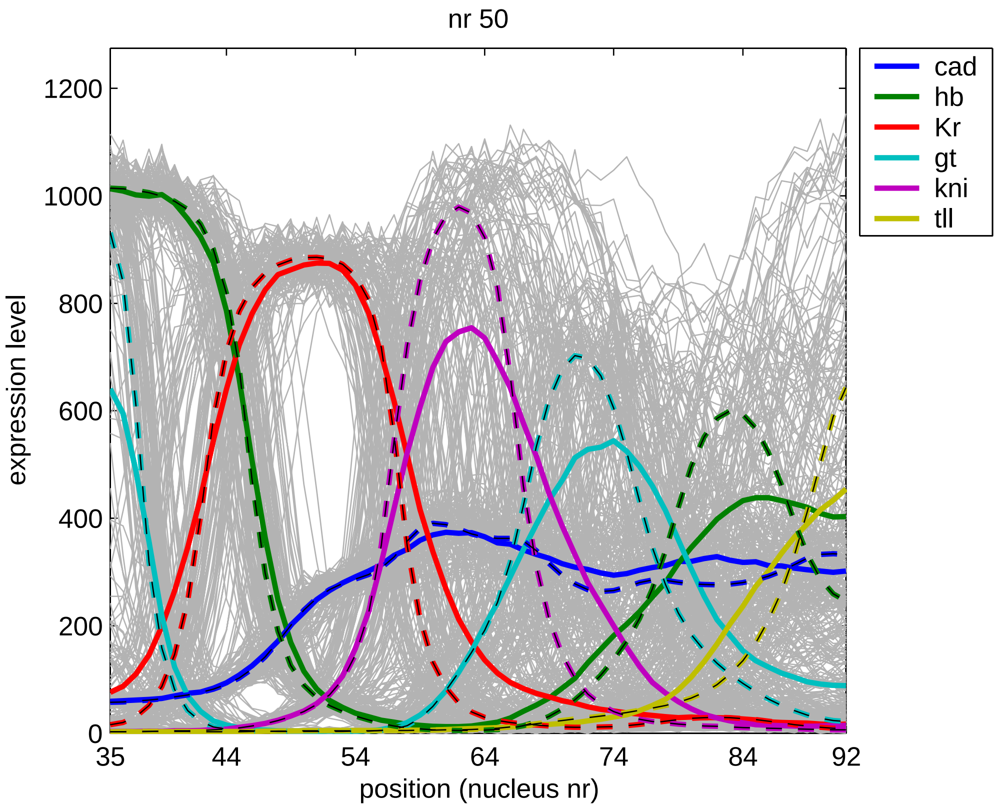

nr 50
expression level
position (nucleus nr)
1200
1000
800
600
400
200
0
35
44
54
64
74
84
92
cad
hb
Kr
gt
kni
tll

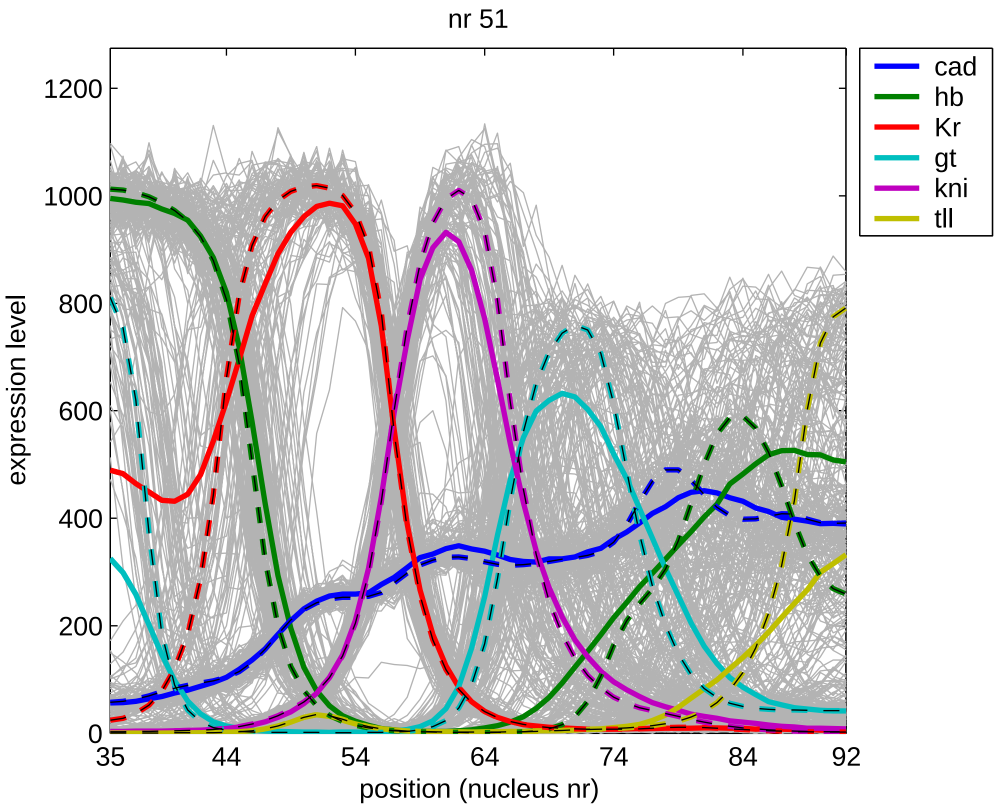
nr 51
expression level
position (nucleus nr)
0
200
400
600
800
1000
1200
35
44
54
64
74
84
92
cad
hb
Kr
gt
kni
tll

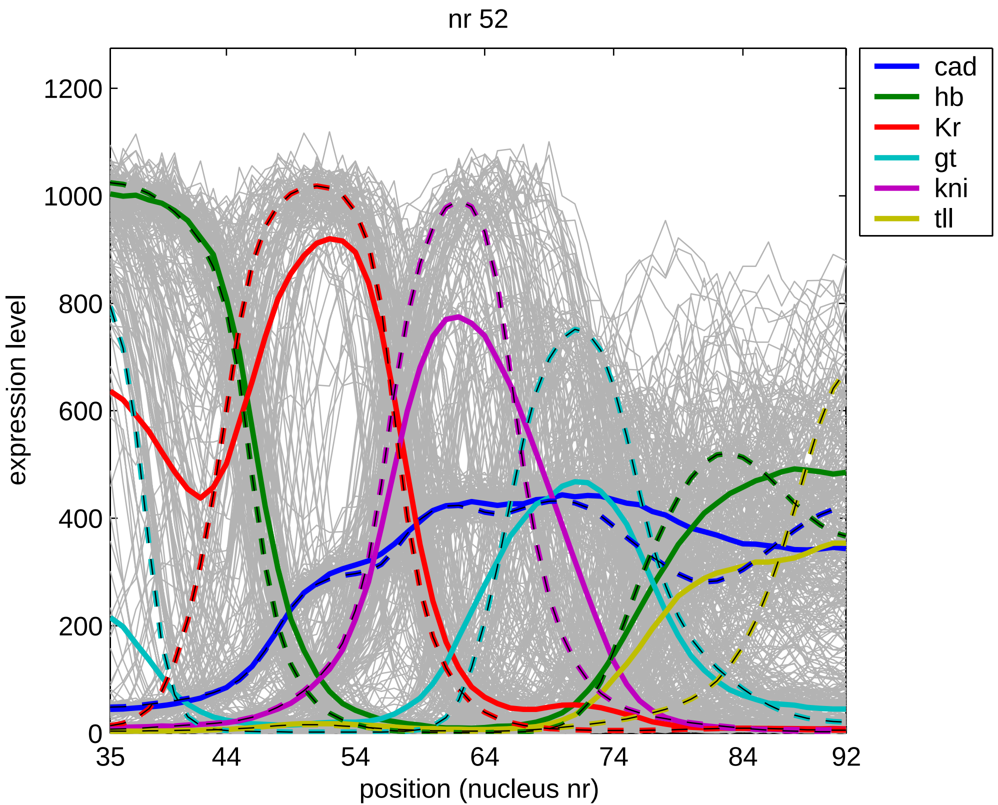

nr 52
1200
1000
800
600
400
200
0
expression level
35
44
54
64
74
84
92
position (nucleus nr)
cad
hb
Kr
gt
kni
tll

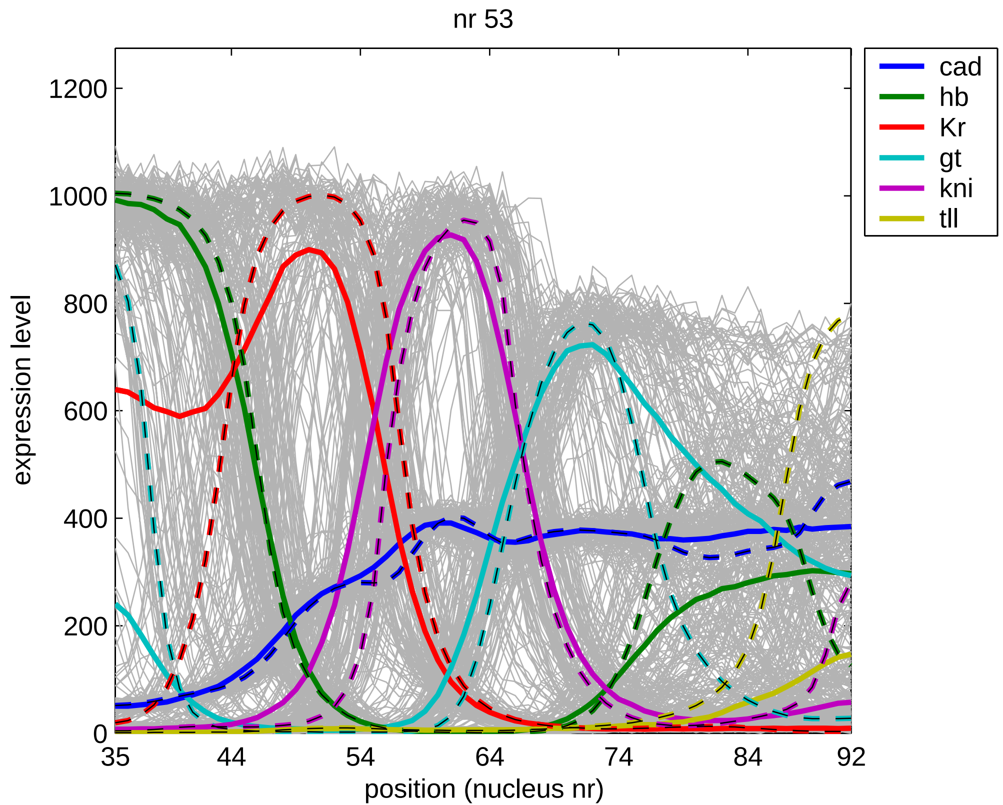

nr 53
cad
hb
Kr
gt
kni
tll
expression level
0
200
400
600
800
1000
1200
35
44
54
64
74
84
92
position (nucleus nr)

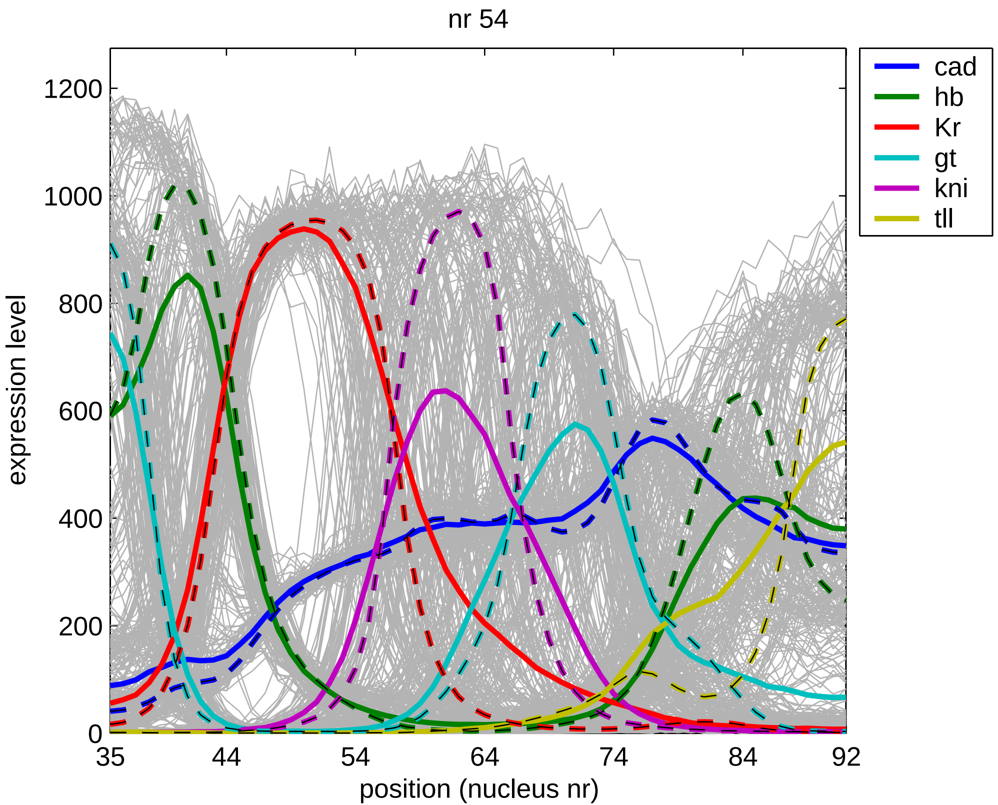

nr 54
expression level
position (nucleus nr)
0
200
400
600
800
1000
1200
35
44
54
64
74
84
92
cad
hb
Kr
gt
kni
tll

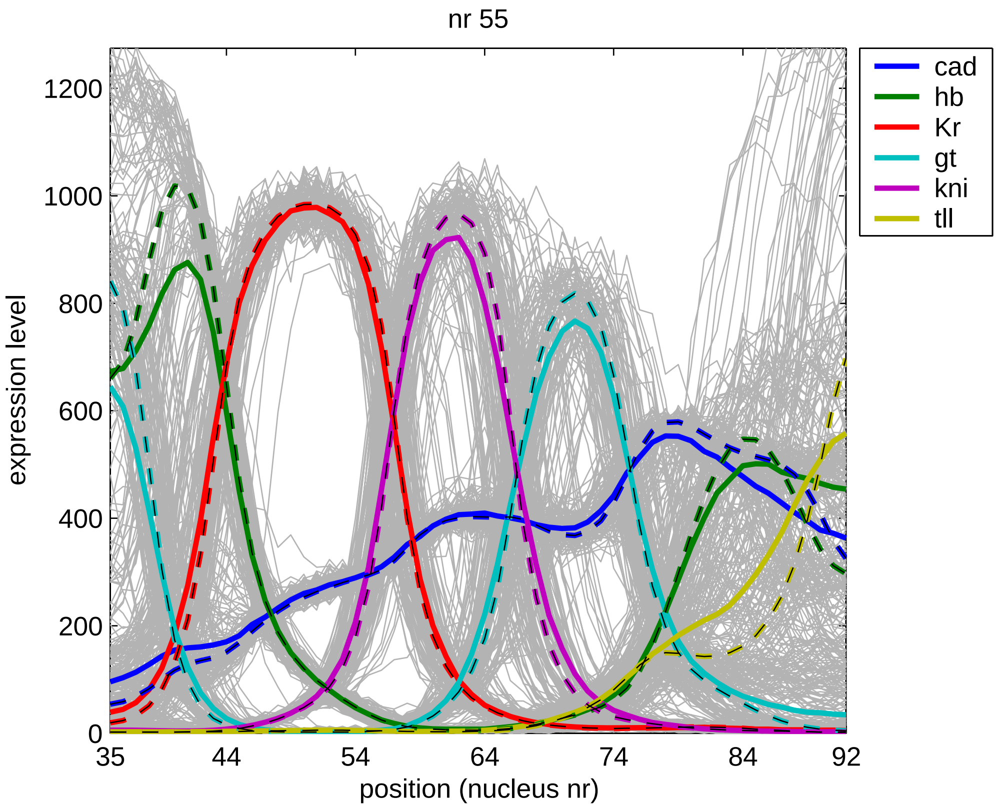
nr 55
expression level
position (nucleus nr)
0
200
400
600
800
1000
1200
35
44
54
64
74
84
92
cad
hb
Kr
gt
kni
tll

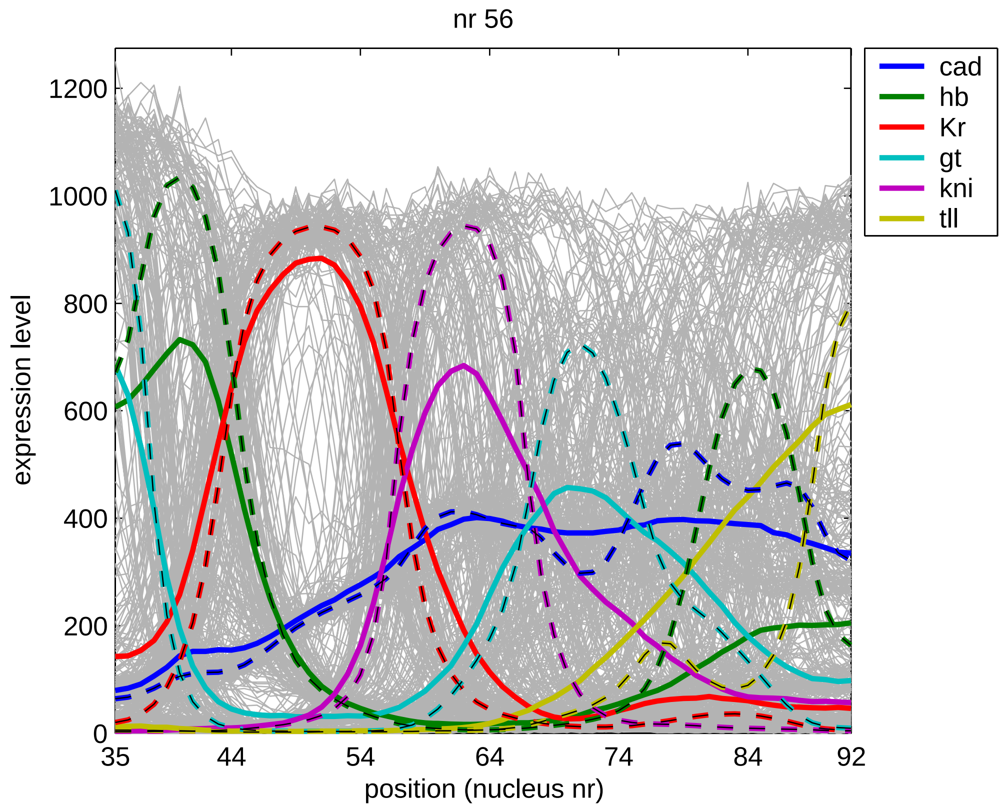

nr 56
cad
hb
Kr
gt
kni
tll
expression level
0
200
400
600
800
1000
1200
35
44
54
64
74
84
92
position (nucleus nr)

nr 57

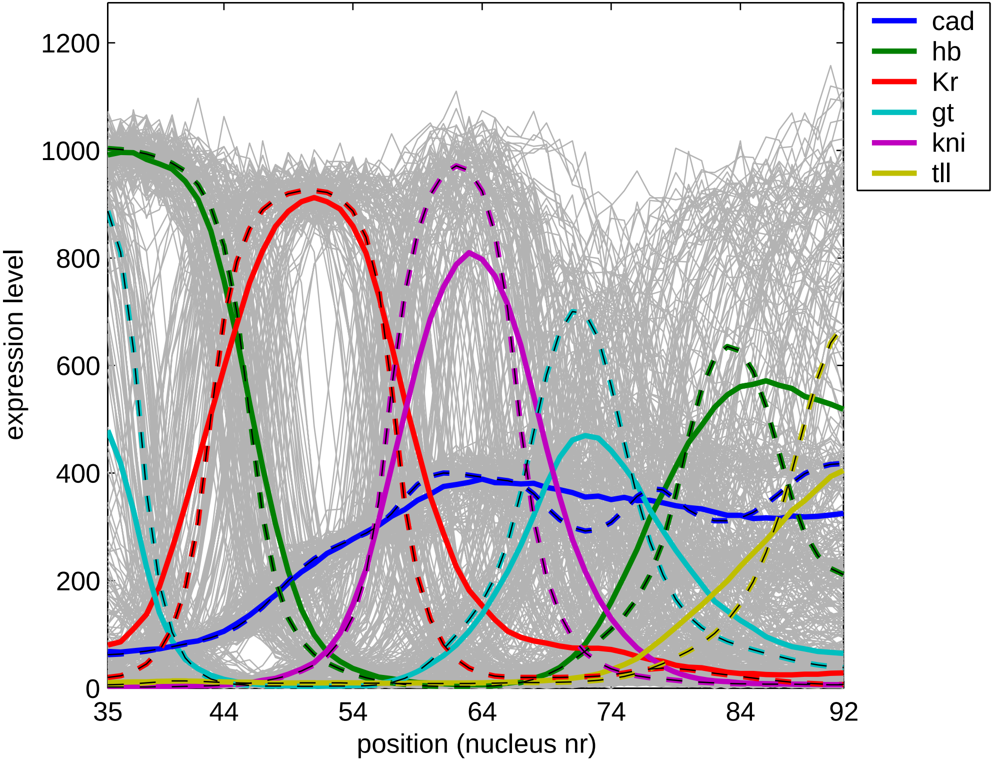

cad
hb
Kr
gt
kni
tll
expression level
position (nucleus nr)
0
200
400
600
800
1000
1200
35
44
54
64
74
84
92

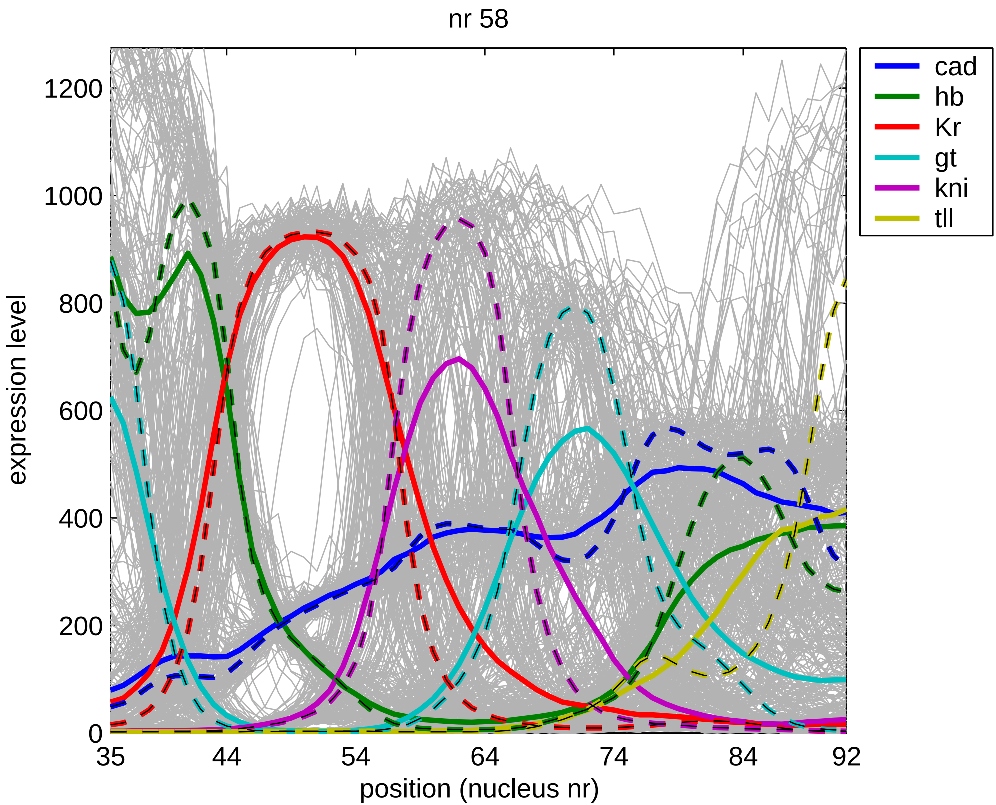
nr 58
expression level
0
200
400
600
800
1000
1200
35
44
54
64
74
84
92
position (nucleus nr)
cad
hb
Kr
gt
kni
tll

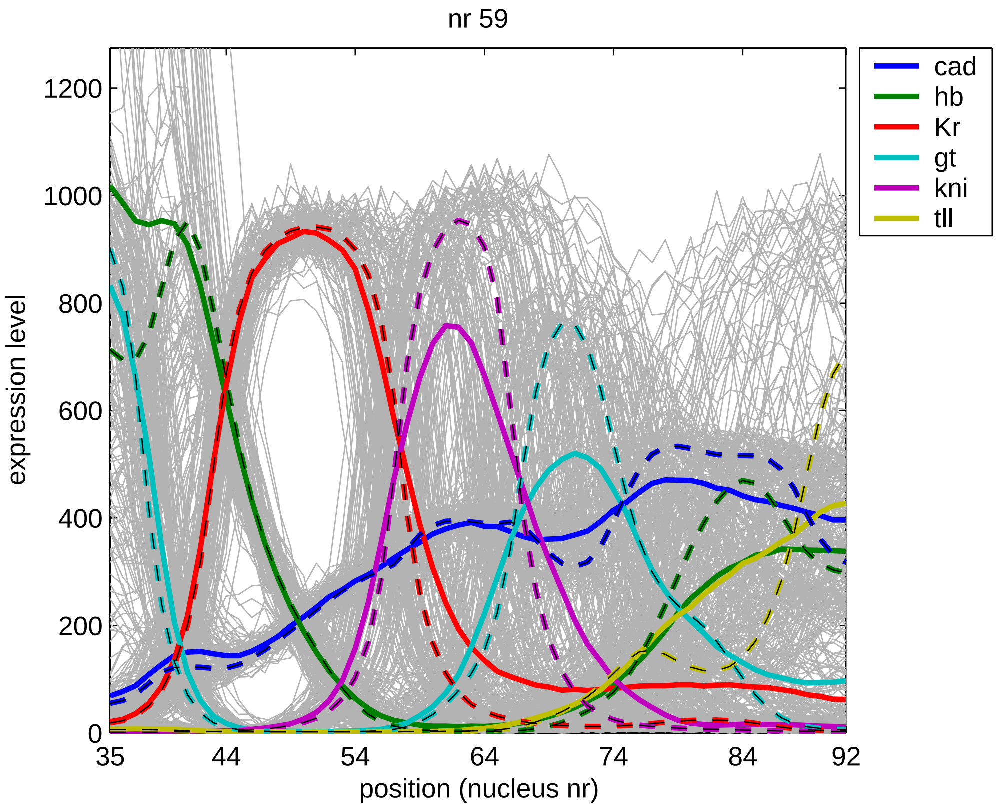
nr 59
expression level
position (nucleus nr)
0
200
400
600
800
1000
1200
35
44
54
64
74
84
92
cad
hb
Kr
gt
kni
tll

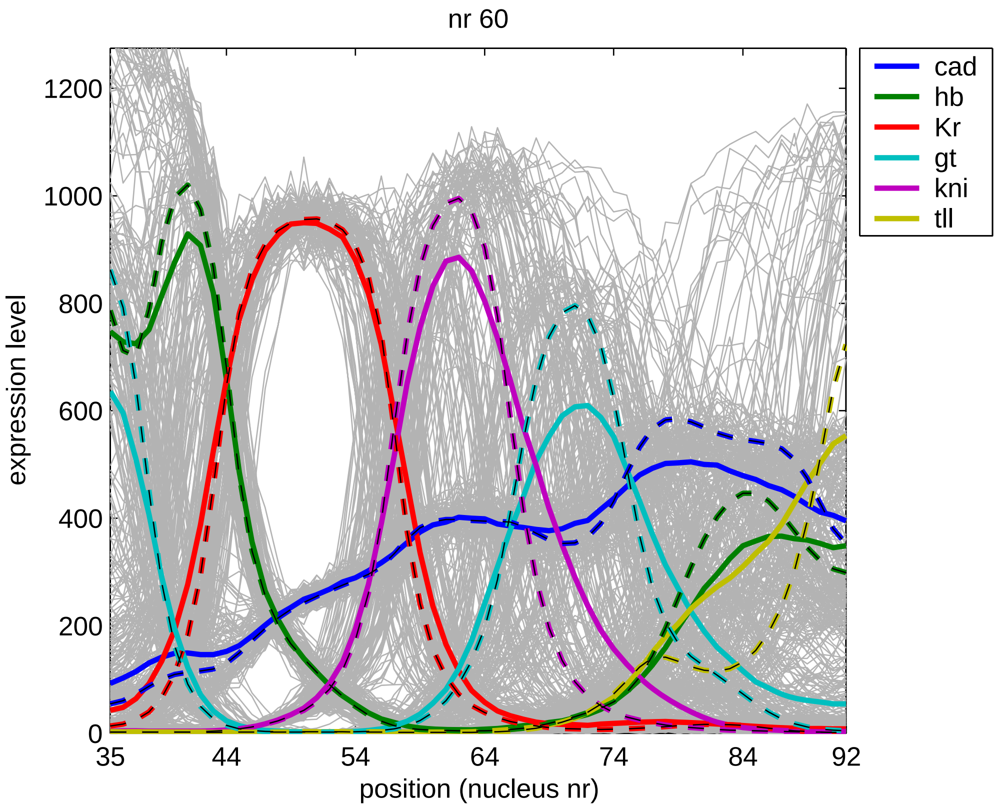

nr 60
expression level
position (nucleus nr)
0
200
400
600
800
1000
1200
35
44
54
64
74
84
92
cad
hb
Kr
gt
kni
tll

nr 61

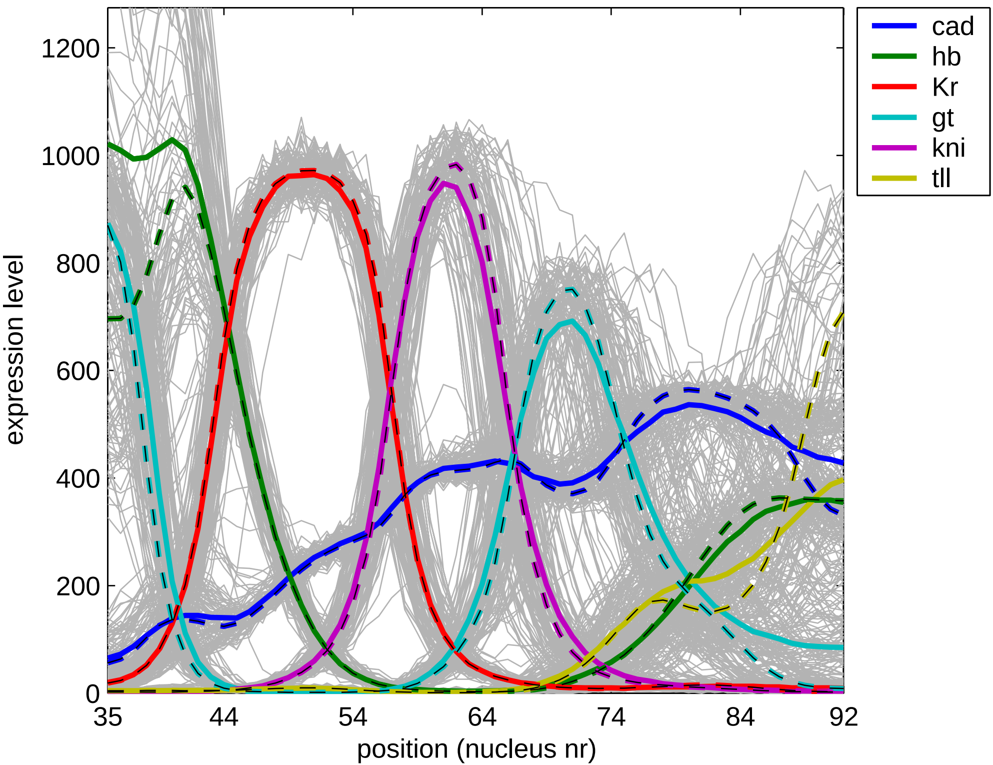

1200
1000
800
600
400
200
0
expression level
35
44
54
64
74
84
92
position (nucleus nr)
cad
hb
Kr
gt
kni
tll

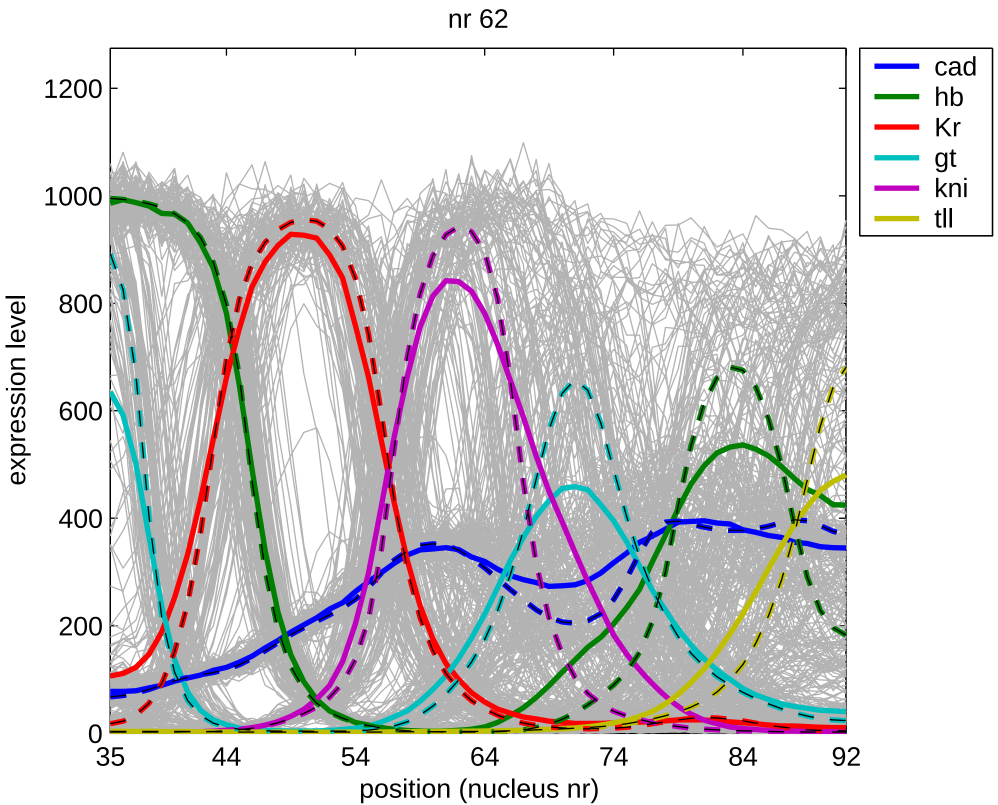
nr 62
cad
hb
Kr
gt
kni
tll
expression level
0
200
400
600
800
1000
1200
35
44
54
64
74
84
92
position (nucleus nr)

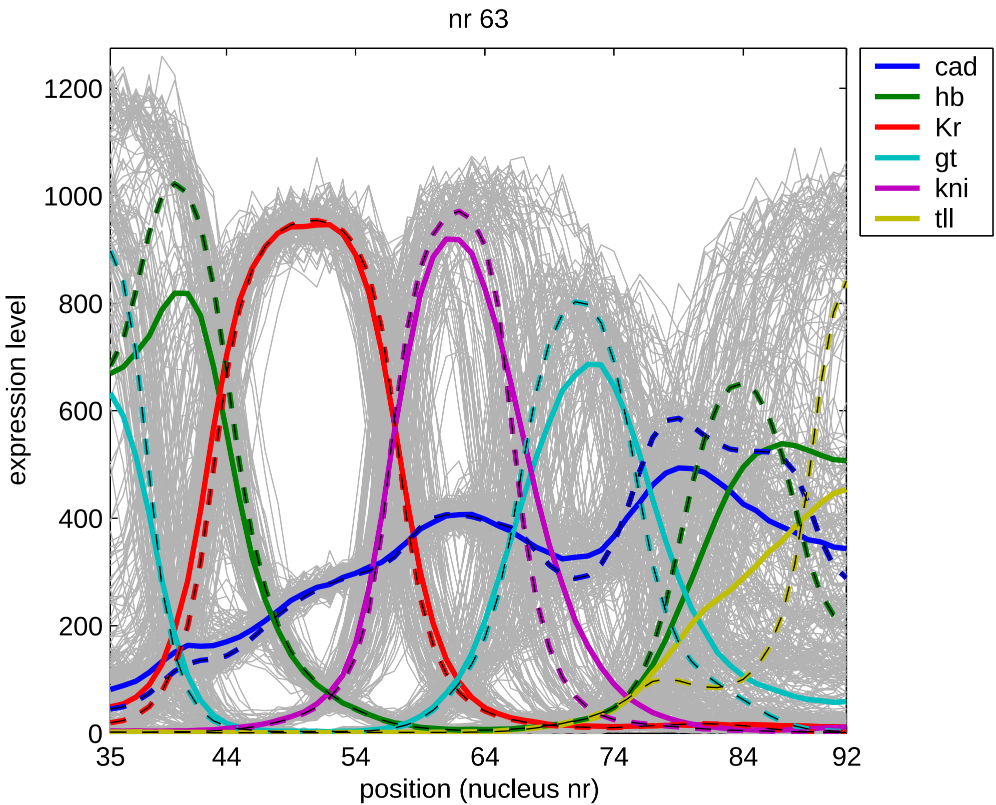

nr 63
1200
1000
800
600
400
200
0
35
44
54
64
74
84
92
expression level
position (nucleus nr)
cad
hb
Kr
gt
kni
tll

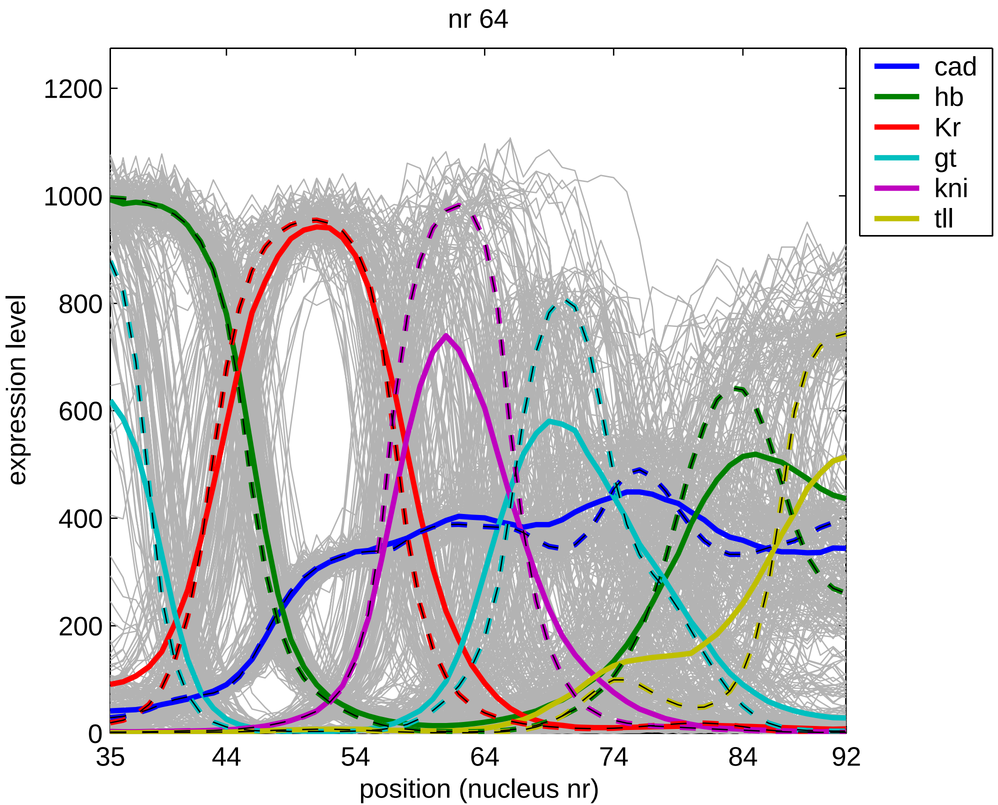
nr 64
expression level
position (nucleus nr)
0
200
400
600
800
1000
1200
35
44
54
64
74
84
92
cad
hb
Kr
gt
kni
tll

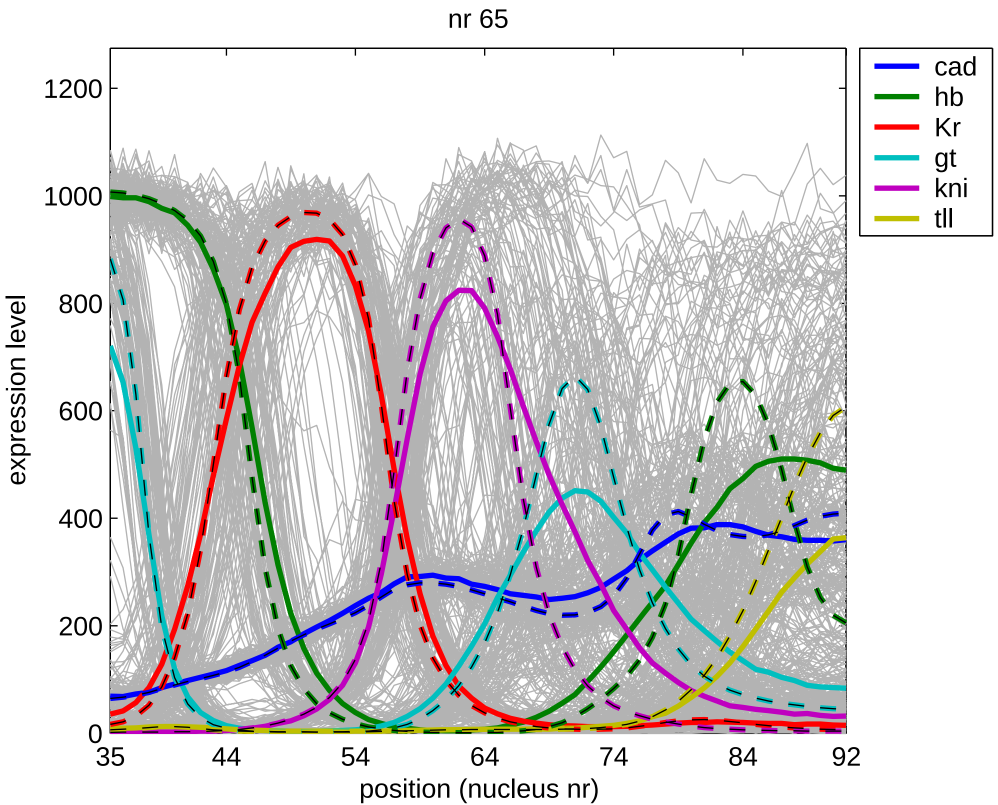

nr 65
expression level
1200
1000
800
600
400
200
0
35
44
54
64
74
84
92
position (nucleus nr)
cad
hb
Kr
gt
kni
tll

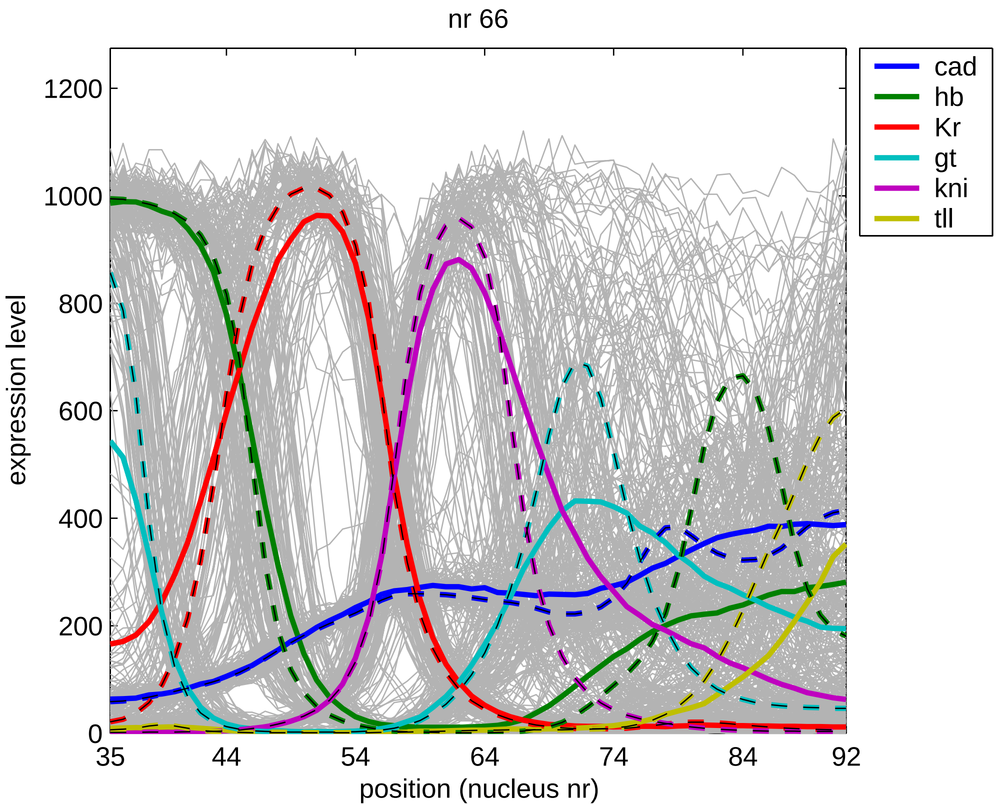

nr 66
expression level
0
200
400
600
800
1000
1200
35
44
54
64
74
84
92
position (nucleus nr)
cad
hb
Kr
gt
kni
tll

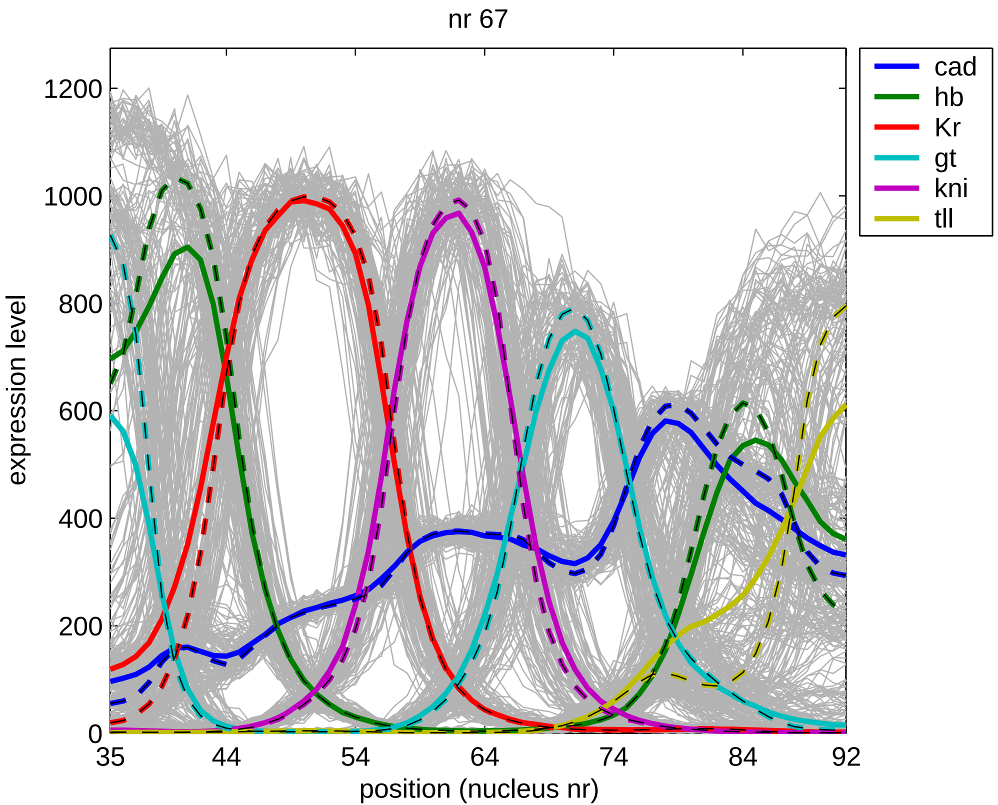

nr 67
expression level
position (nucleus nr)
0
200
400
600
800
1000
1200
35
44
54
64
74
84
92
cad
hb
Kr
gt
kni
tll

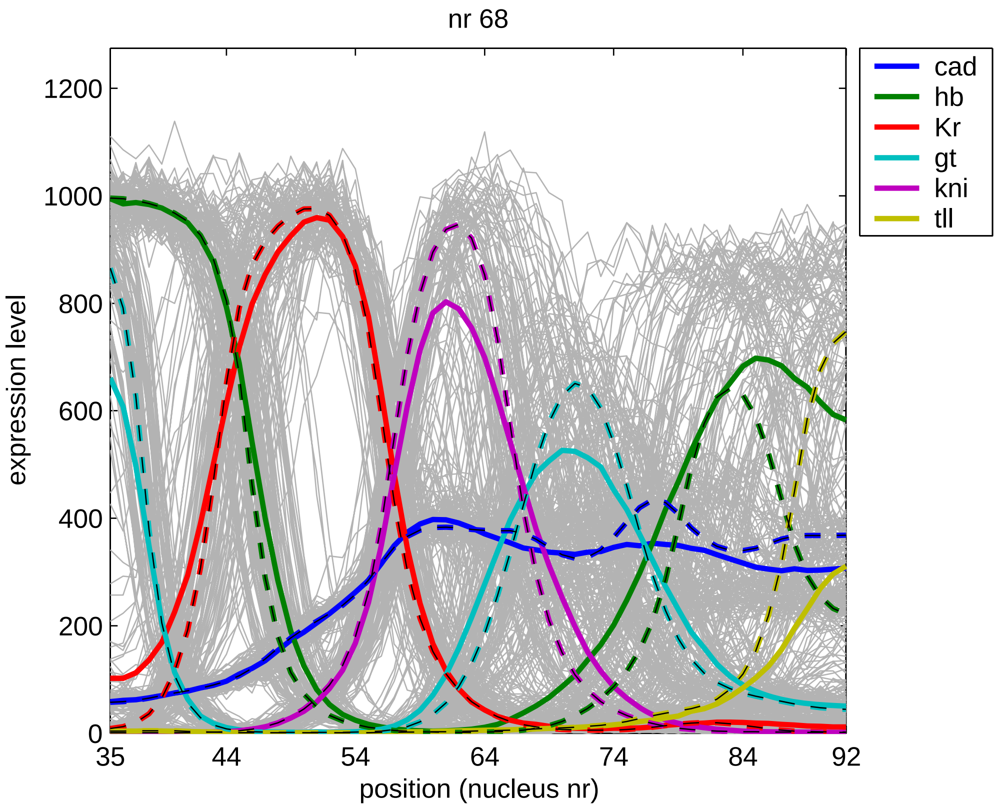
nr 68
expression level
position (nucleus nr)
0
200
400
600
800
1000
1200
35
44
54
64
74
84
92
cad
hb
Kr
gt
kni
tll

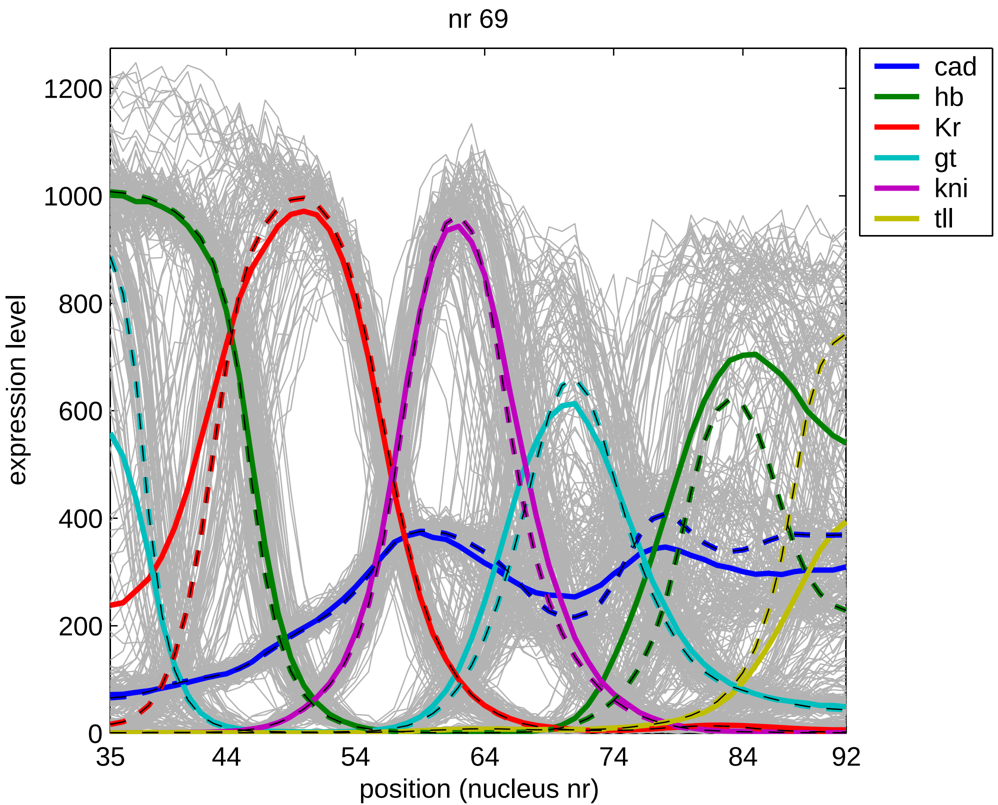
nr 69
expression level
1200
1000
800
600
400
200
0
35
44
54
64
74
84
92
position (nucleus nr)
cad
hb
Kr
gt
kni
tll

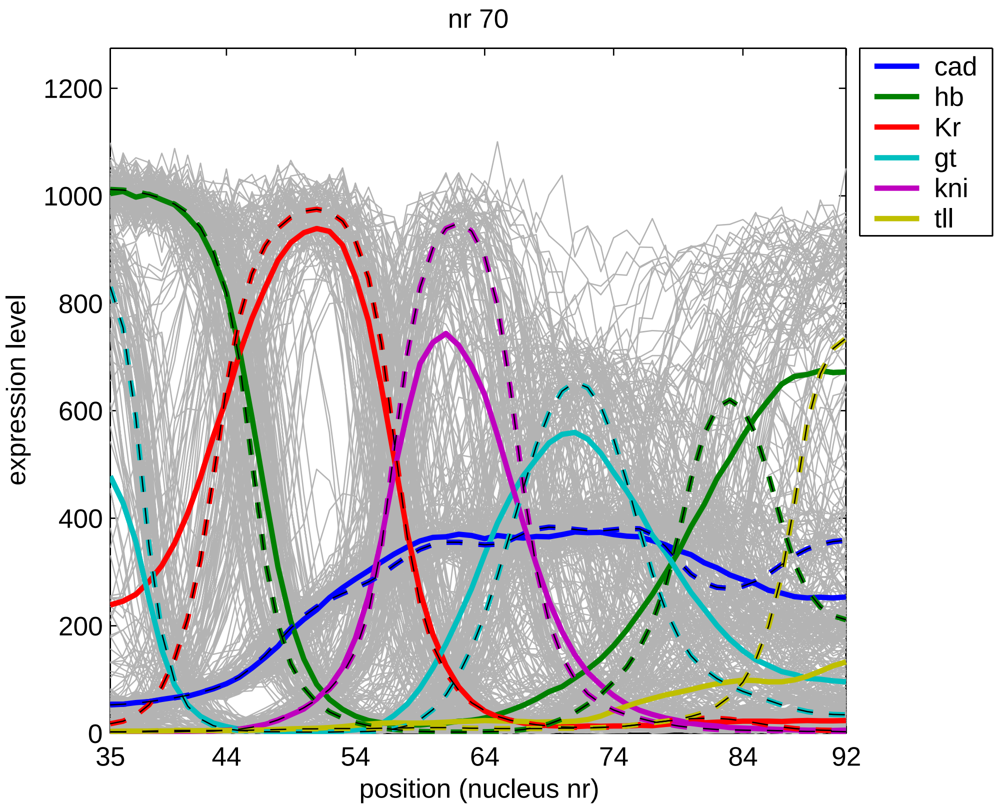
nr 70
expression level
0
200
400
600
800
1000
1200
35
44
54
64
74
84
92
position (nucleus nr)
cad
hb
Kr
gt
kni
tll

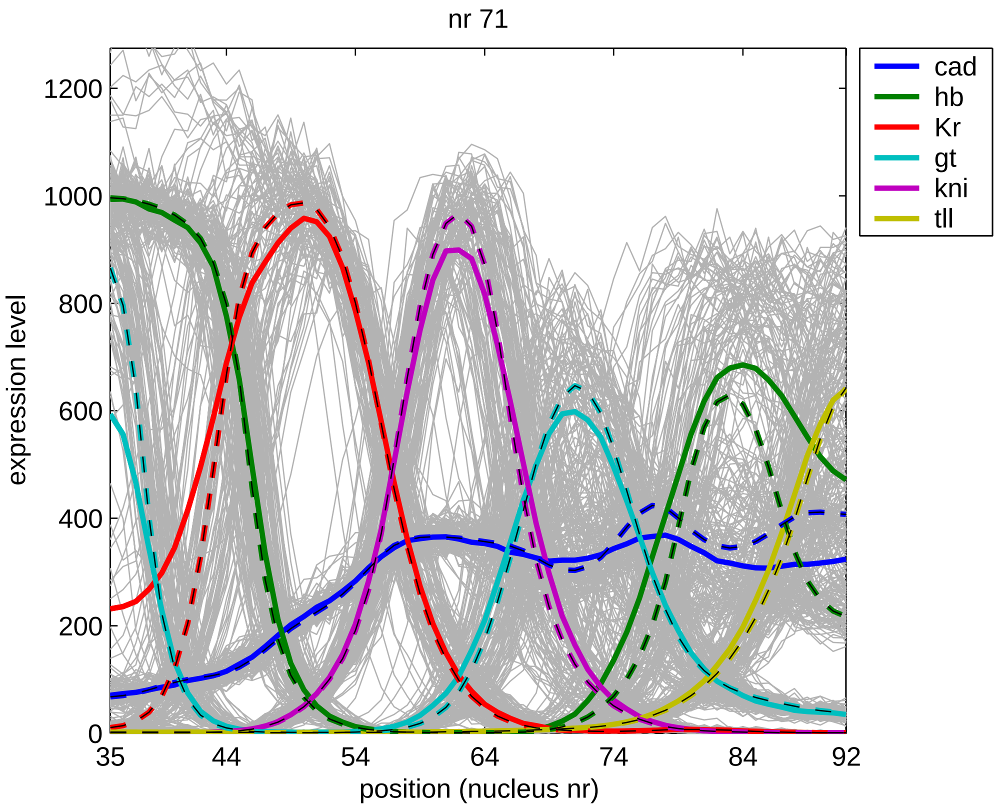
nr 71
expression level
position (nucleus nr)
0
200
400
600
800
1000
1200
35
44
54
64
74
84
92
cad
hb
Kr
gt
kni
tll

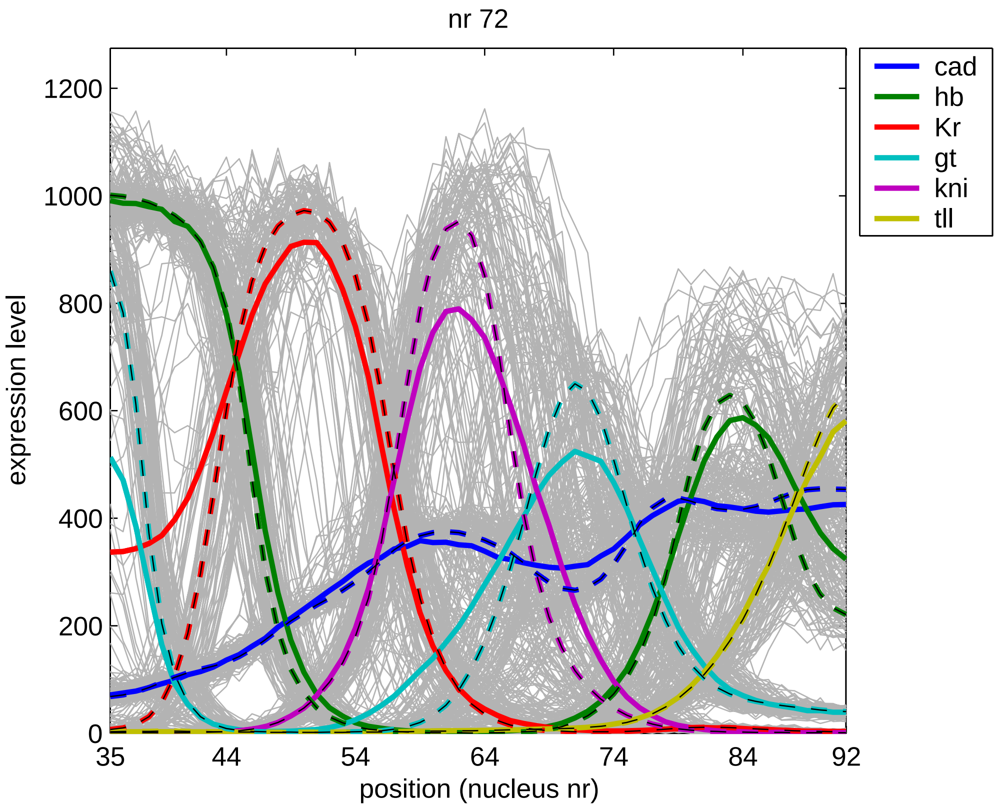
nr 72
expression level
position (nucleus nr)
0
200
400
600
800
1000
1200
35
44
54
64
74
84
92
cad
hb
Kr
gt
kni
tll

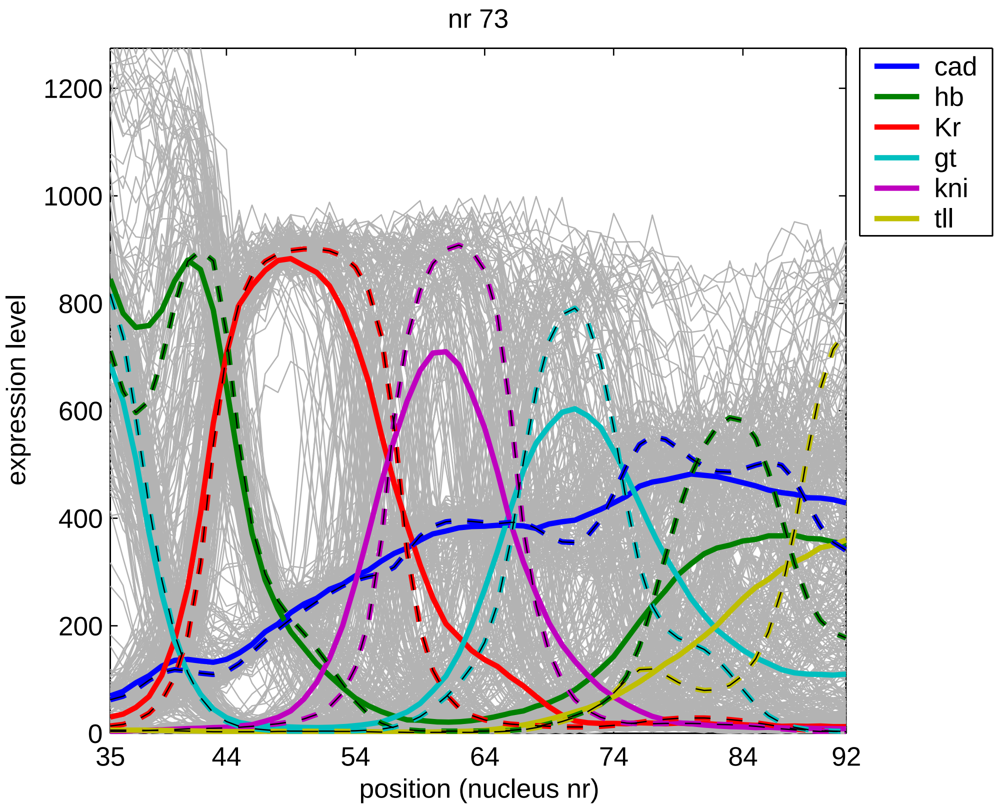

nr 73
expression level
position (nucleus nr)
0
200
400
600
800
1000
1200
35
44
54
64
74
84
92
cad
hb
Kr
gt
kni
tll

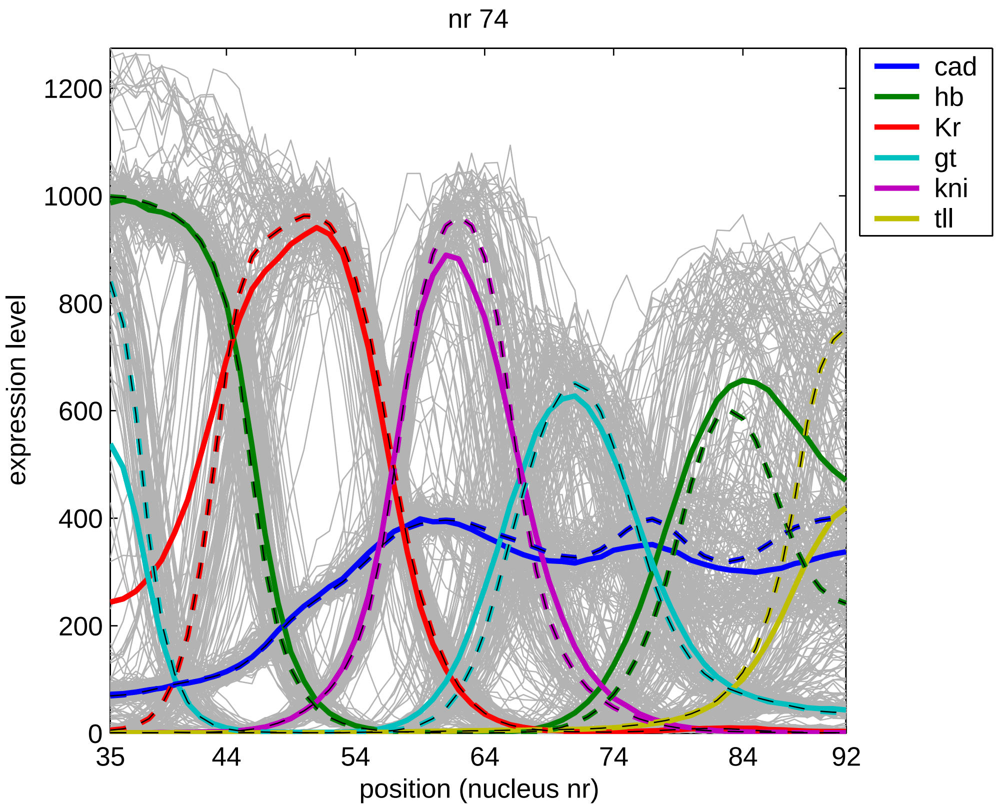
nr 74
expression level
position (nucleus nr)
0
200
400
600
800
1000
1200
35
44
54
64
74
84
92
cad
hb
Kr
gt
kni
tll

nr 75

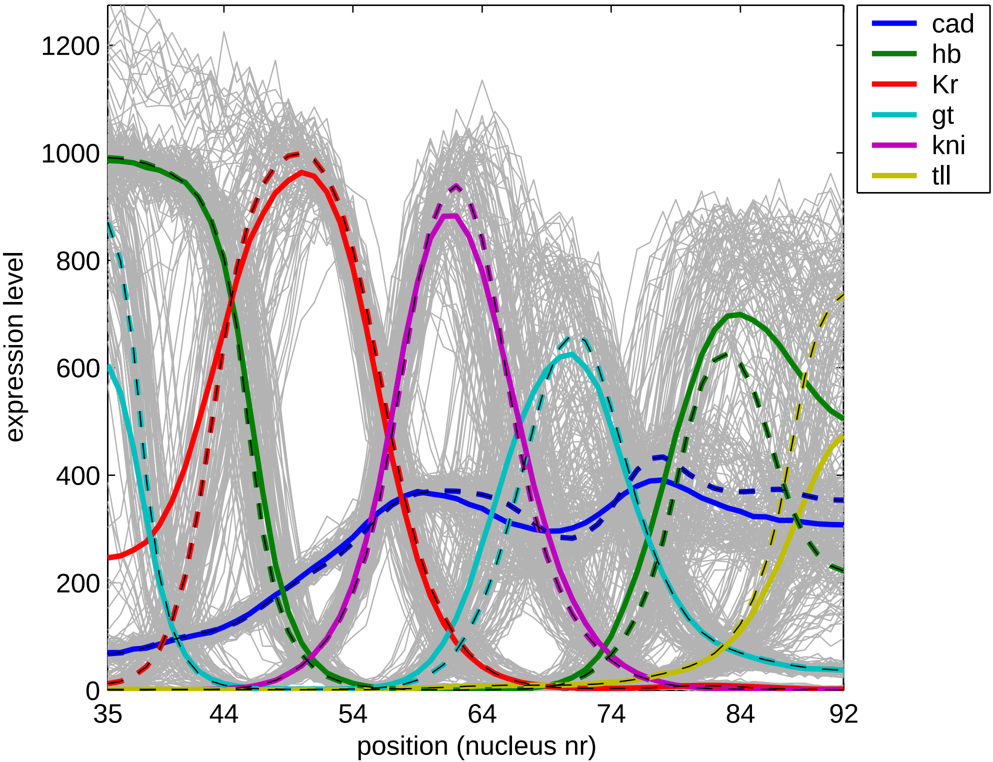

1200
1000
800
600
400
200
0
35
44
54
64
74
84
92
expression level
position (nucleus nr)
cad
hb
Kr
gt
kni
tll

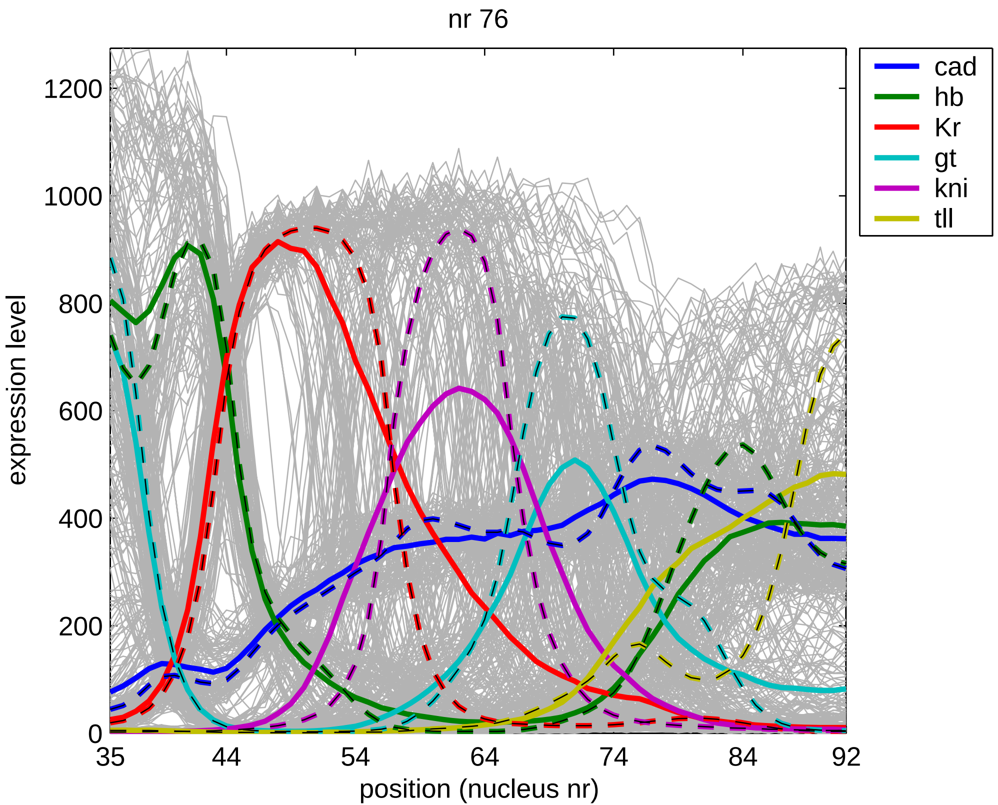

nr 76
cad
hb
Kr
gt
kni
tll
expression level
position (nucleus nr)
0
200
400
600
800
1000
1200
35
44
54
64
74
84
92

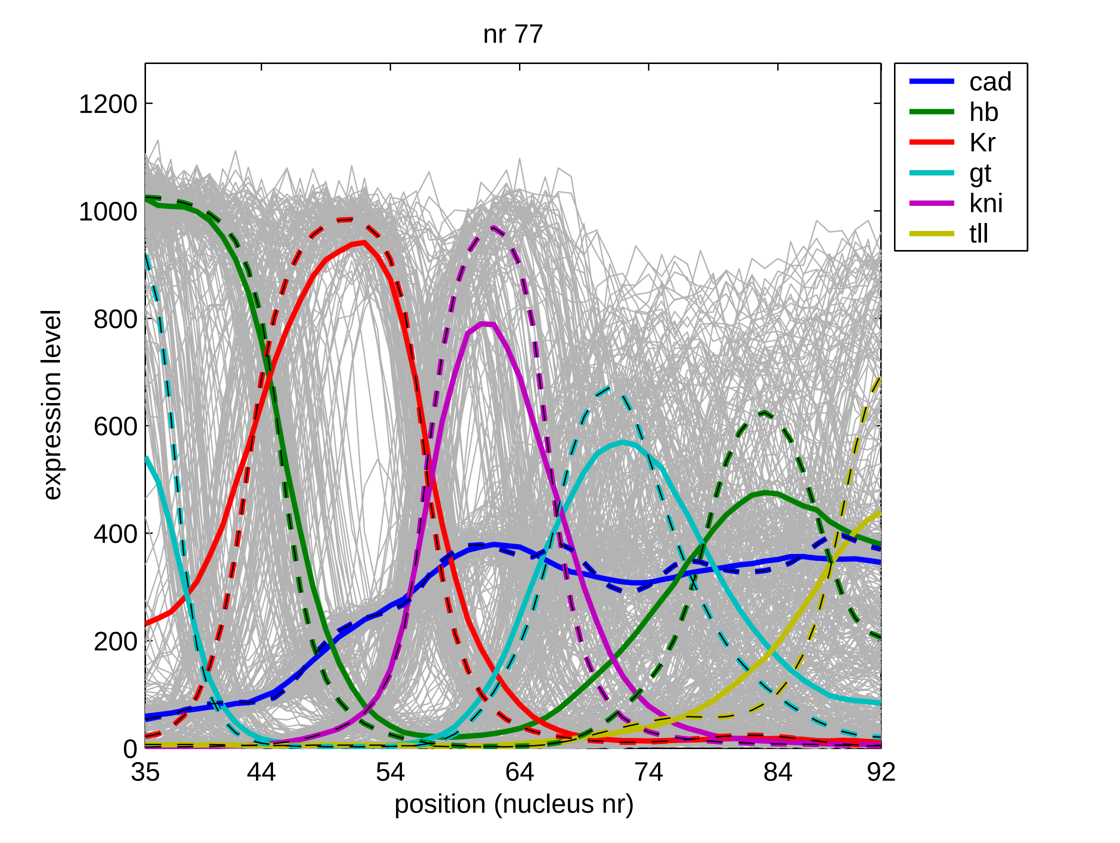
nr 77
expression level
position (nucleus nr)
0
200
400
600
800
1000
1200
35
44
54
64
74
84
92
cad
hb
Kr
gt
kni
tll

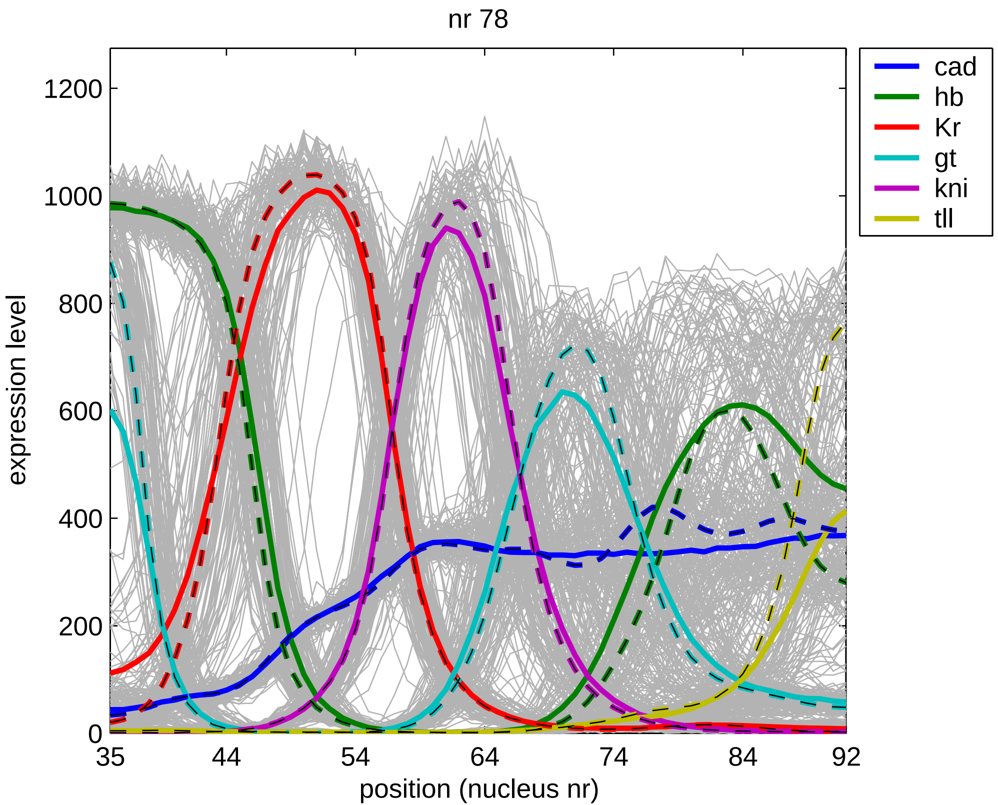

nr 78
expression level
1200
1000
800
600
400
200
0
35
44
54
64
74
84
92
position (nucleus nr)
cad
hb
Kr
gt
kni
tll

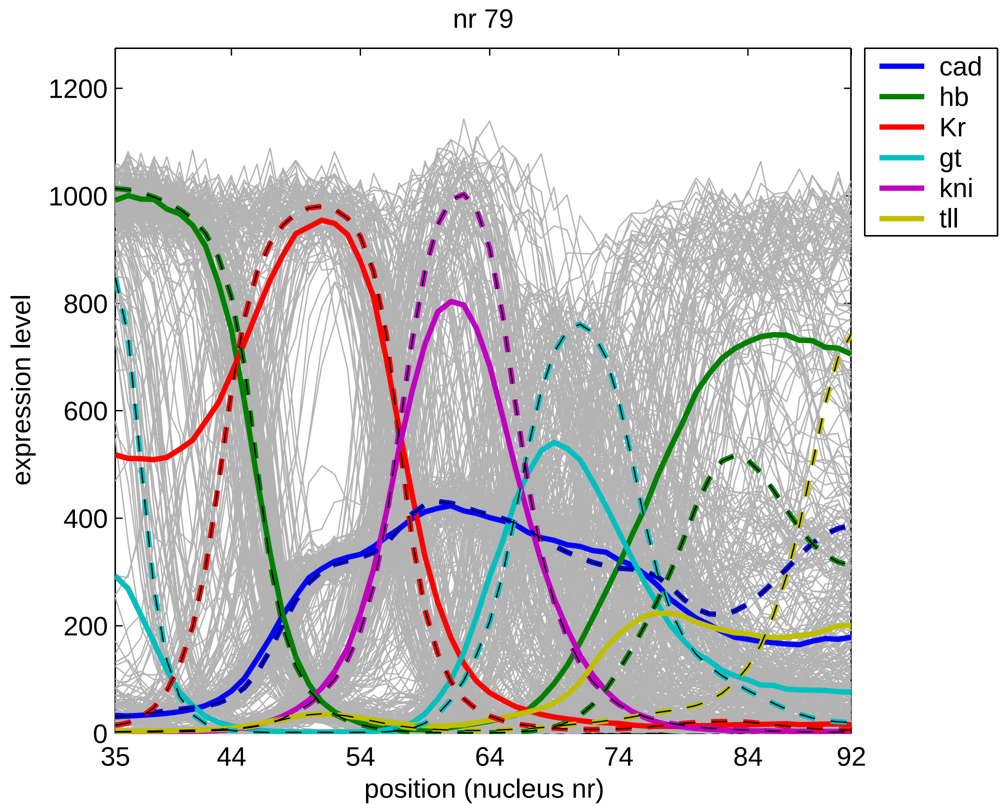

nr 79
1200
1000
800
600
400
200
0
expression level
35
44
54
64
74
84
92
position (nucleus nr)
cad
hb
Kr
gt
kni
tll

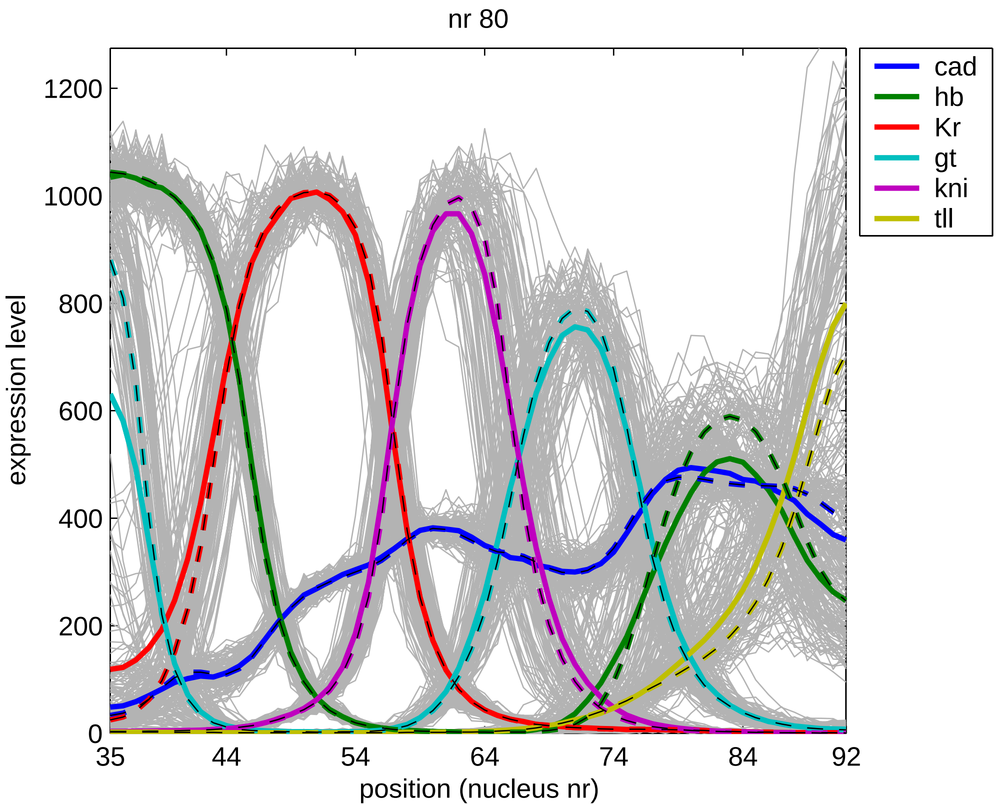
nr 80
expression level
position (nucleus nr)
0
200
400
600
800
1000
1200
35
44
54
64
74
84
92
cad
hb
Kr
gt
kni
tll

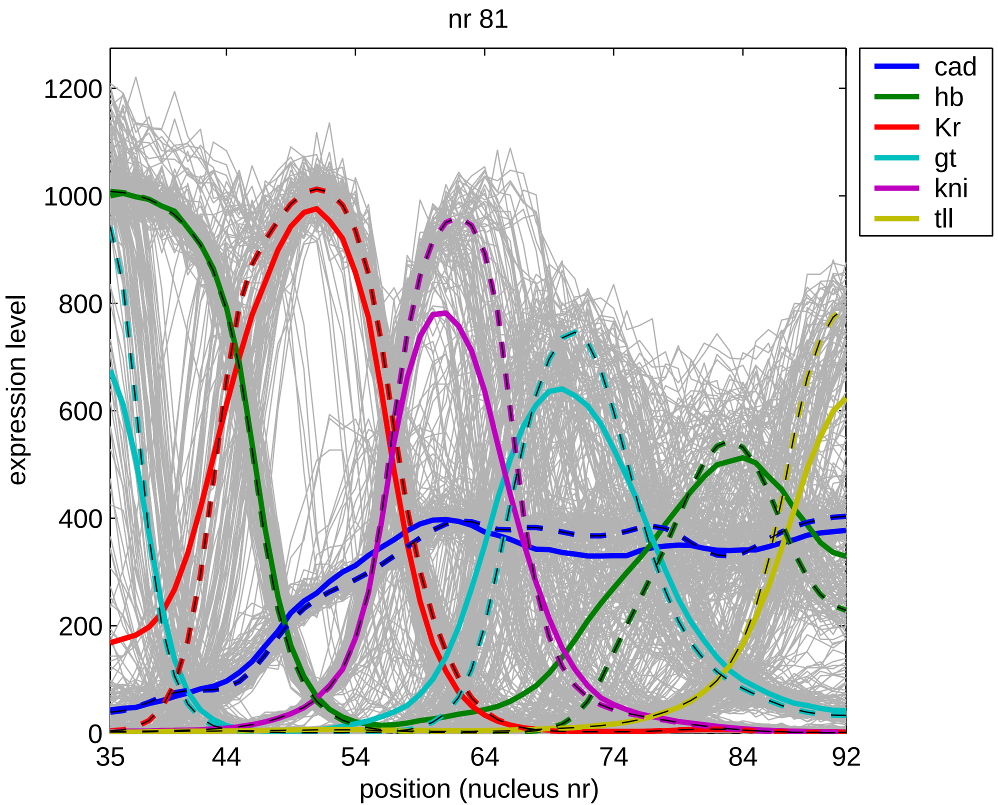
nr 81
expression level
0
200
400
600
800
1000
1200
35
44
54
64
74
84
92
position (nucleus nr)
cad
hb
Kr
gt
kni
tll

nr 82

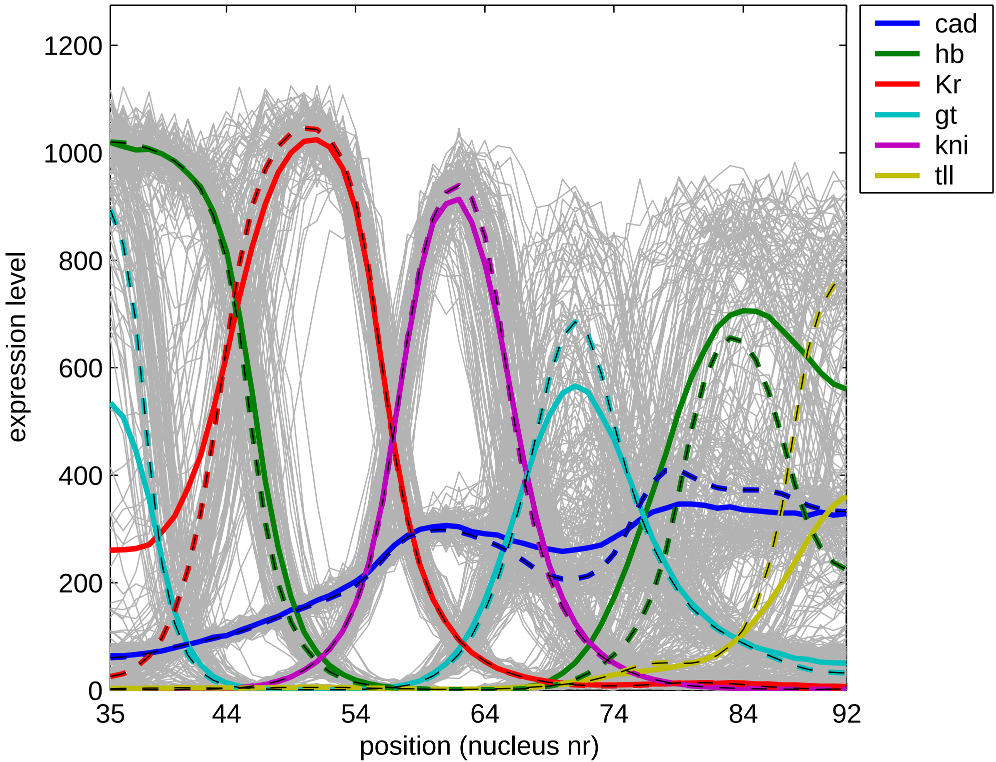

1200
1000
800
600
400
200
0
expression level
35
44
54
64
74
84
92
position (nucleus nr)
cad
hb
Kr
gt
kni
tll

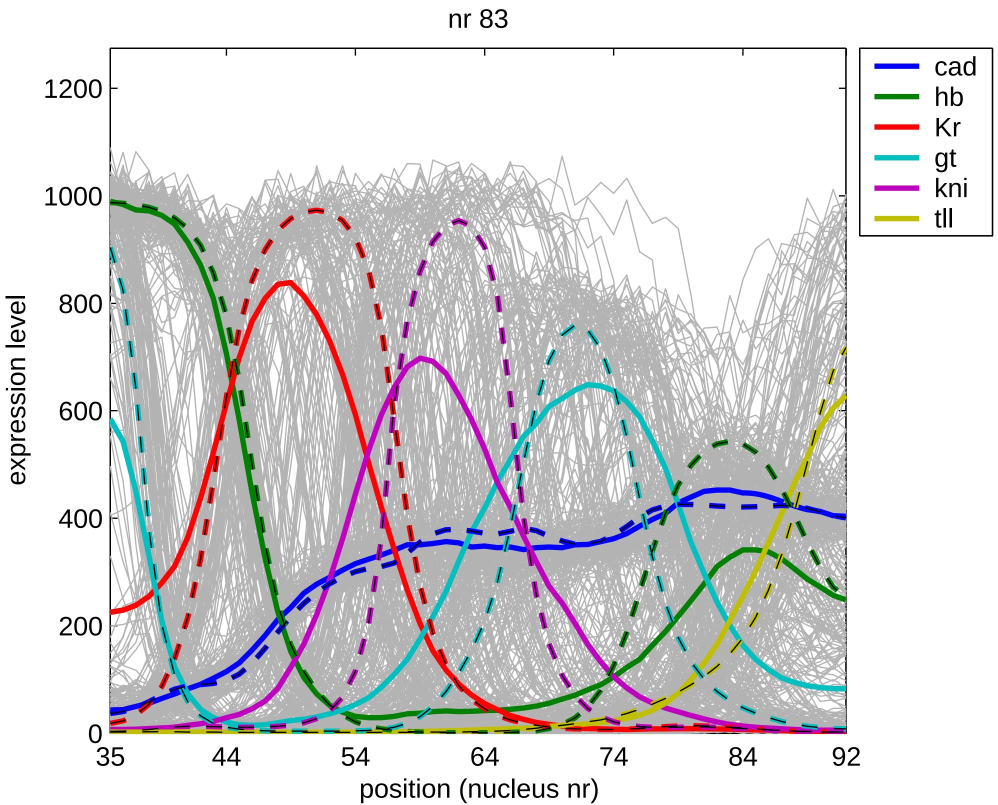

nr 83
expression level
position (nucleus nr)
0
200
400
600
800
1000
1200
35
44
54
64
74
84
92
cad
hb
Kr
gt
kni
tll

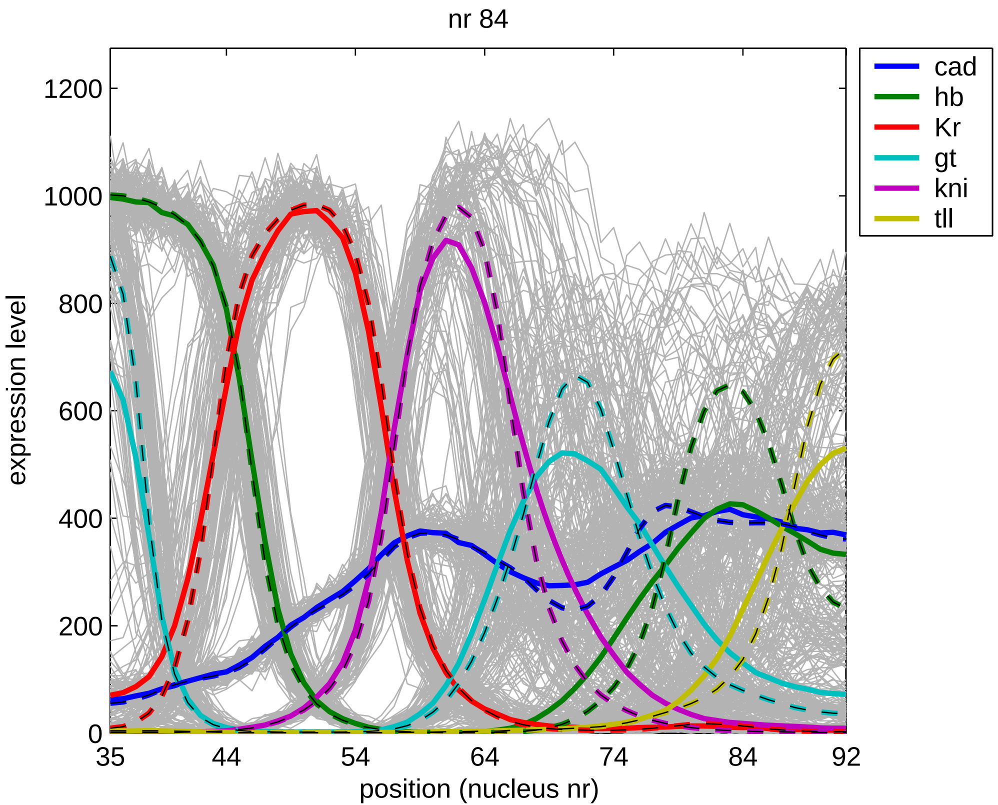

nr 84
expression level
position (nucleus nr)
0
200
400
600
800
1000
1200
35
44
54
64
74
84
92
cad
hb
Kr
gt
kni
tll

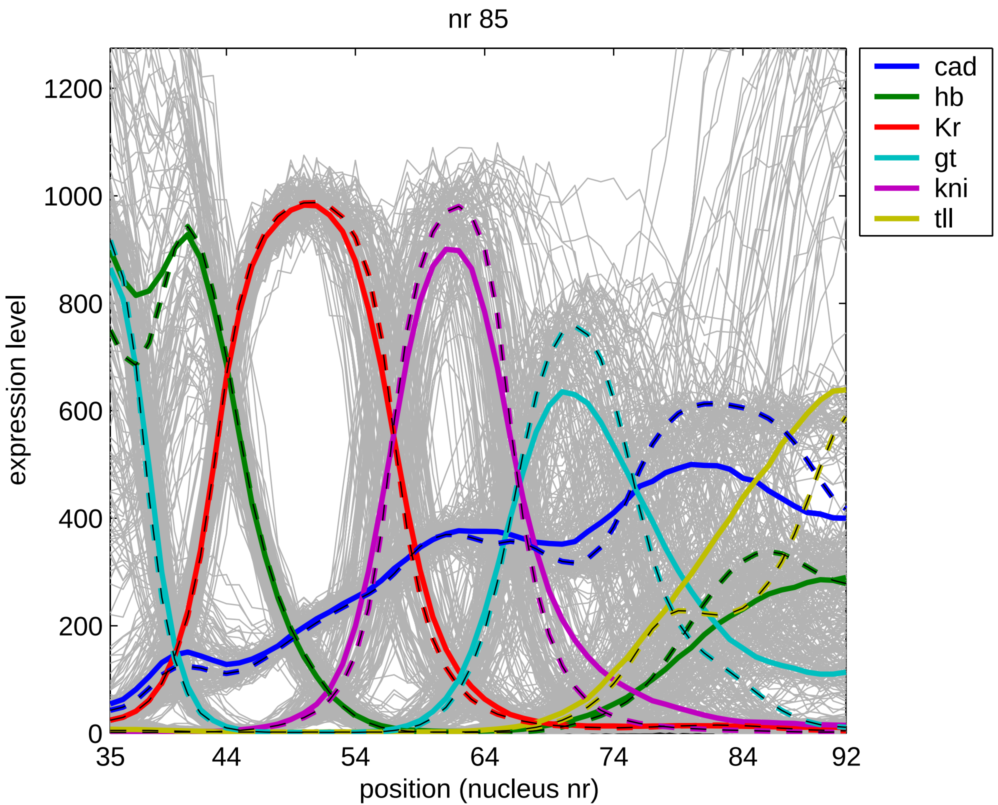

nr 85
expression level
position (nucleus nr)
0
200
400
600
800
1000
1200
35
44
54
64
74
84
92
cad
hb
Kr
gt
kni
tll

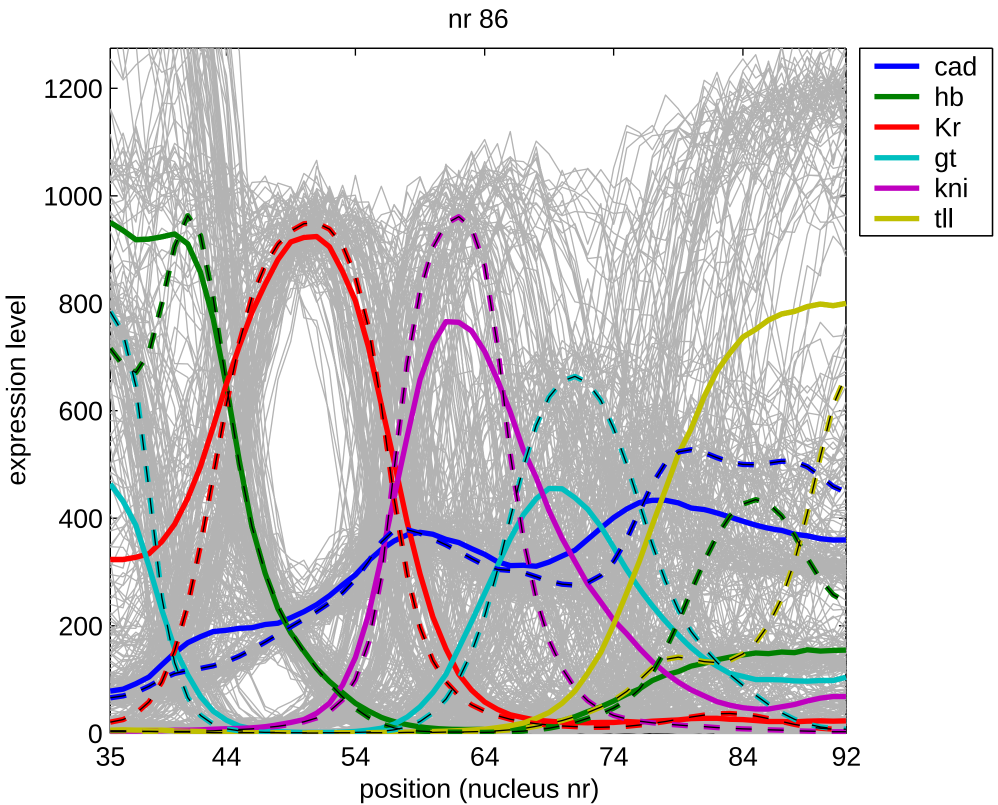

nr 86
expression level
position (nucleus nr)
0
200
400
600
800
1000
1200
35
44
54
64
74
84
92
cad
hb
Kr
gt
kni
tll

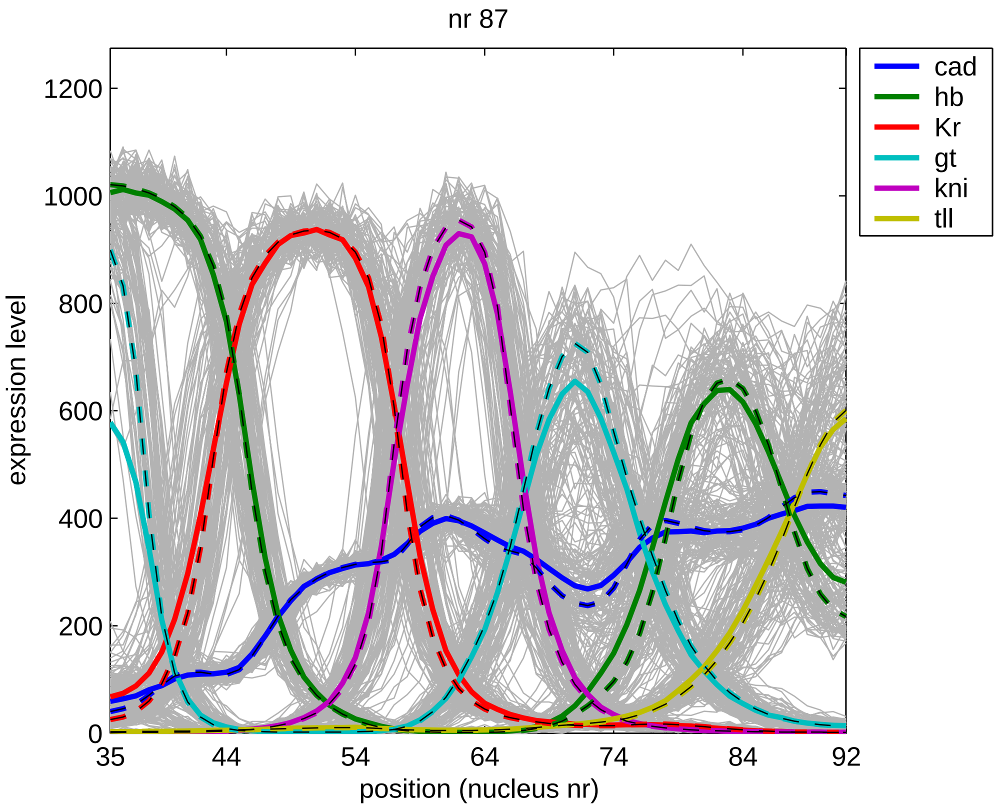

nr 87
1200
1000
800
600
400
200
0
expression level
35
44
54
64
74
84
92
position (nucleus nr)
cad
hb
Kr
gt
kni
tll

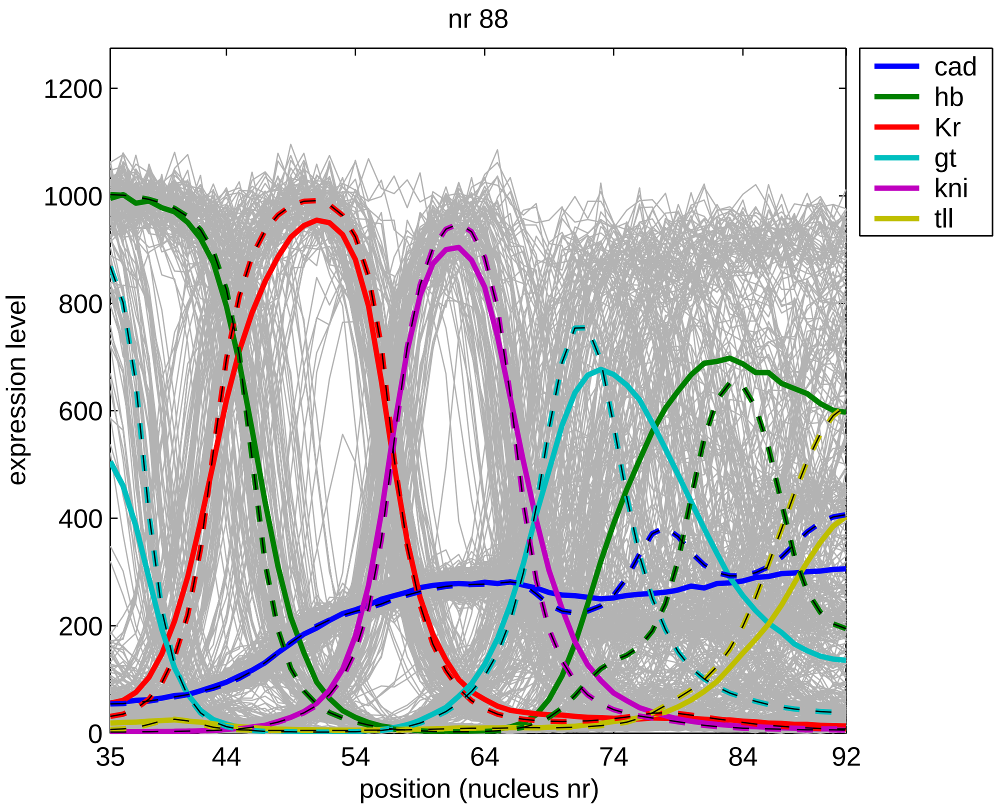

nr 88
1200
1000
800
600
400
200
0
expression level
35
44
54
64
74
84
92
position (nucleus nr)
cad
hb
Kr
gt
kni
tll

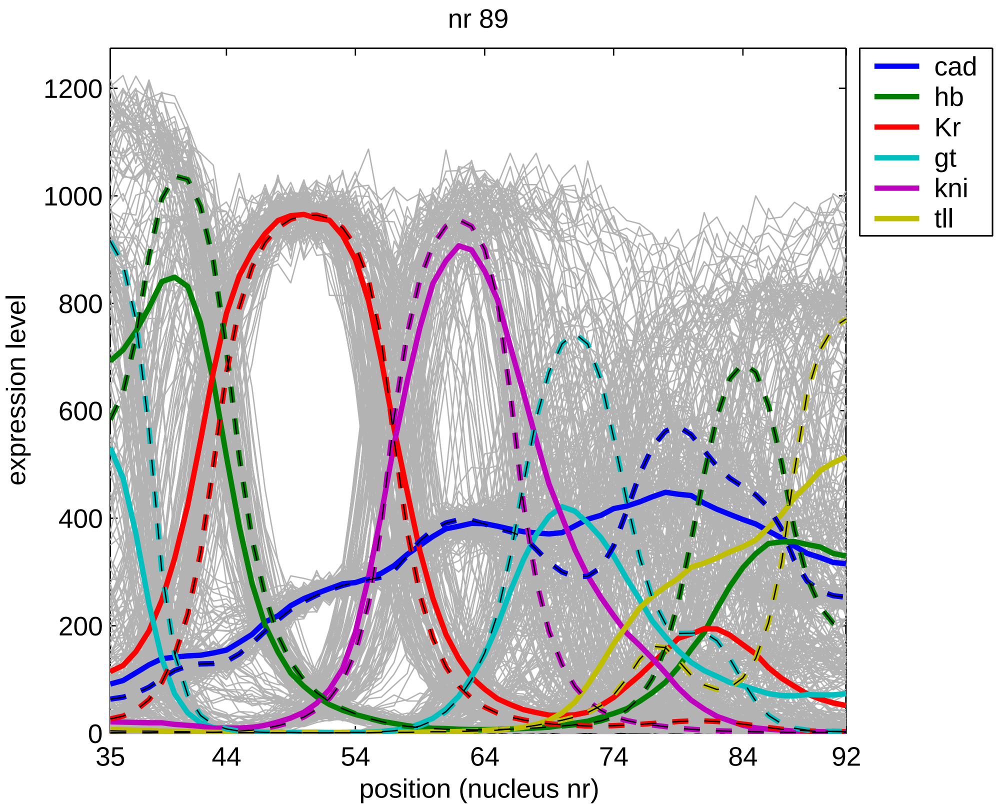
nr 89
expression level
position (nucleus nr)
0
200
400
600
800
1000
1200
35
44
54
64
74
84
92
cad
hb
Kr
gt
kni
tll

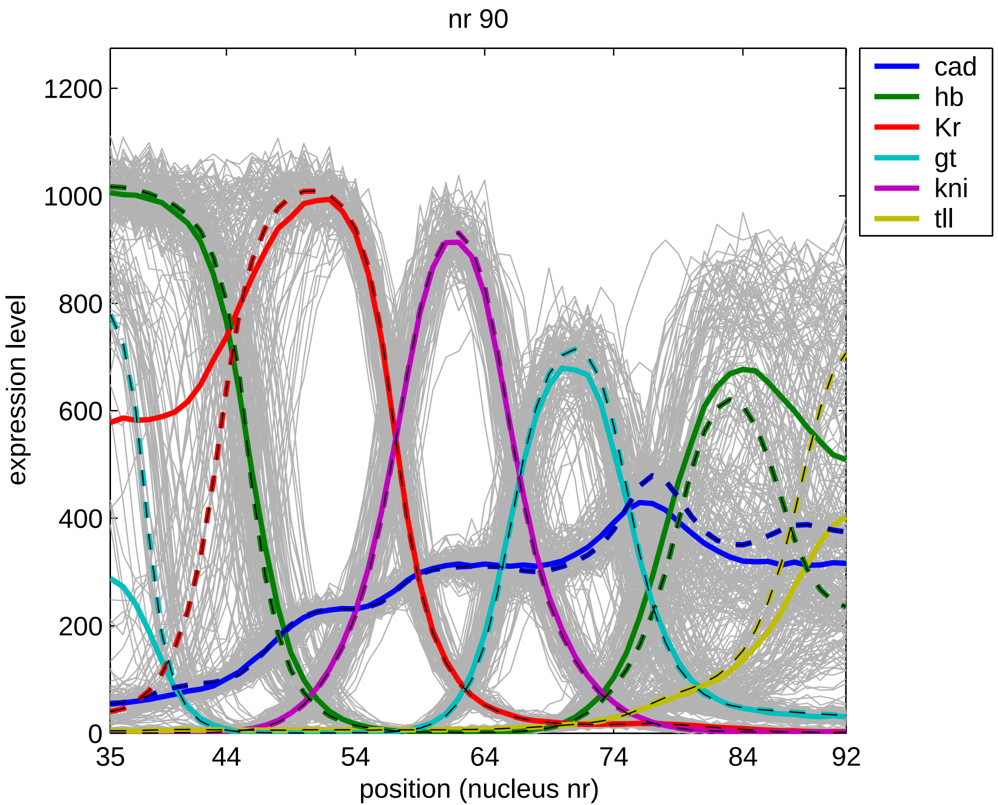

nr 90
expression level
position (nucleus nr)
0
200
400
600
800
1000
1200
35
44
54
64
74
84
92
cad
hb
Kr
gt
kni
tll

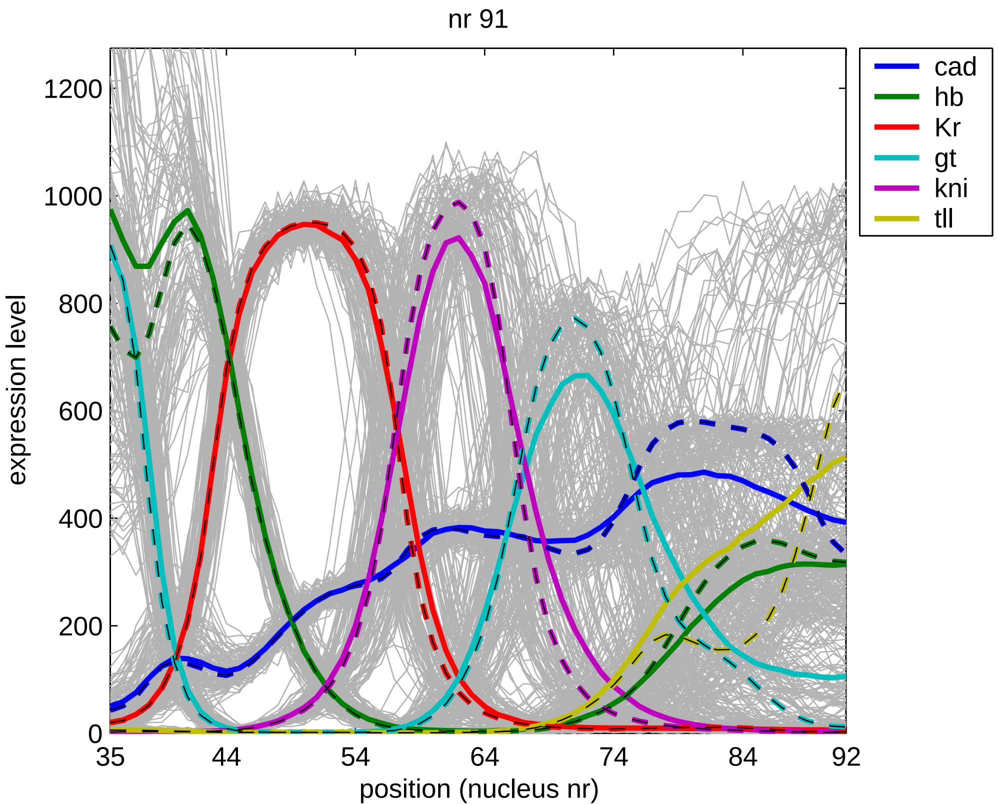

nr 91
expression level
position (nucleus nr)
0
200
400
600
800
1000
1200
35
44
54
64
74
84
92
cad
hb
Kr
gt
kni
tll

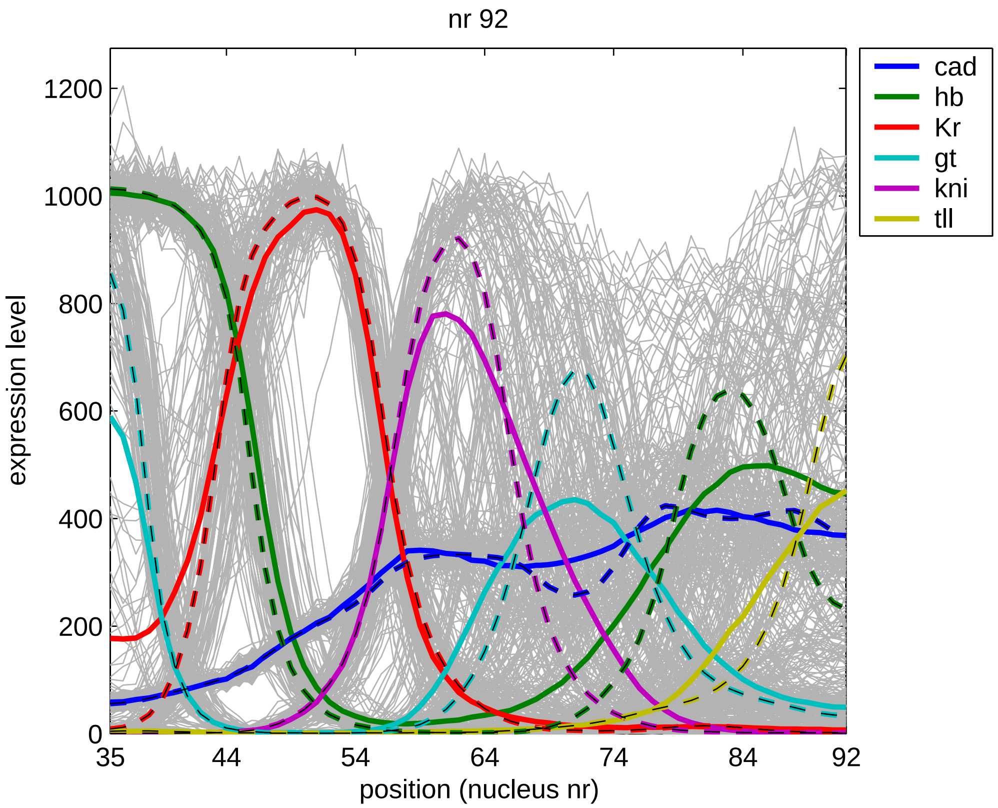
nr 92
expression level
0
200
400
600
800
1000
1200
35
44
54
64
74
84
92
position (nucleus nr)
cad
hb
Kr
gt
kni
tll

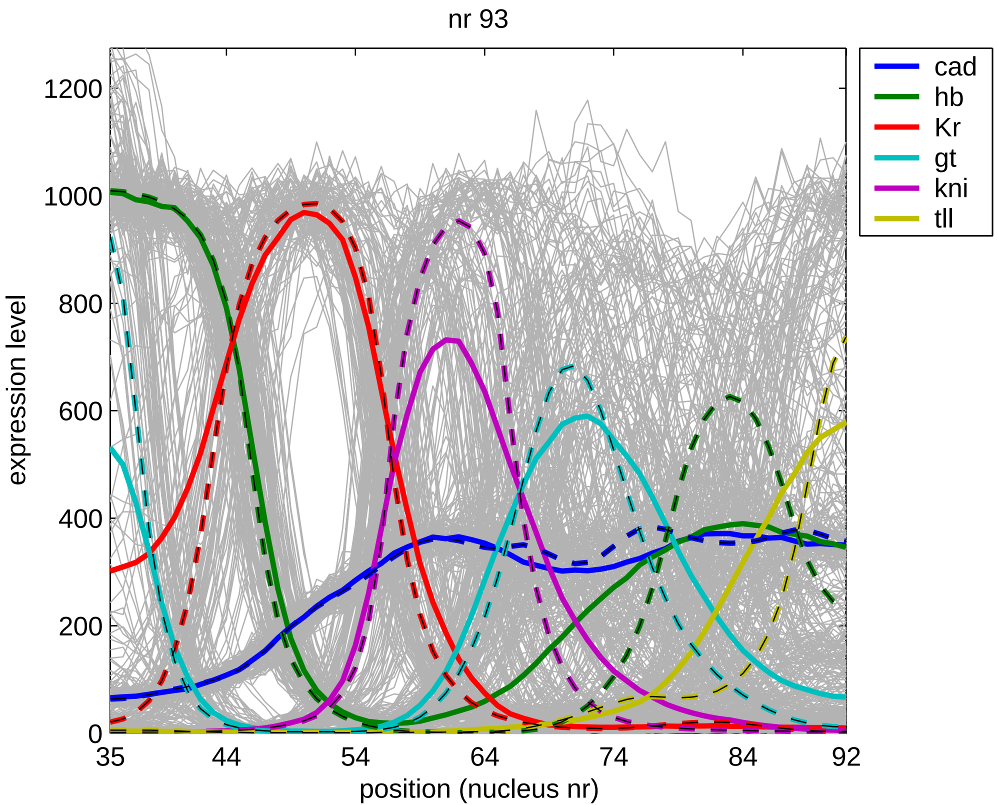
nr 93
expression level
position (nucleus nr)
0
200
400
600
800
1000
1200
35
44
54
64
74
84
92
cad
hb
Kr
gt
kni
tll

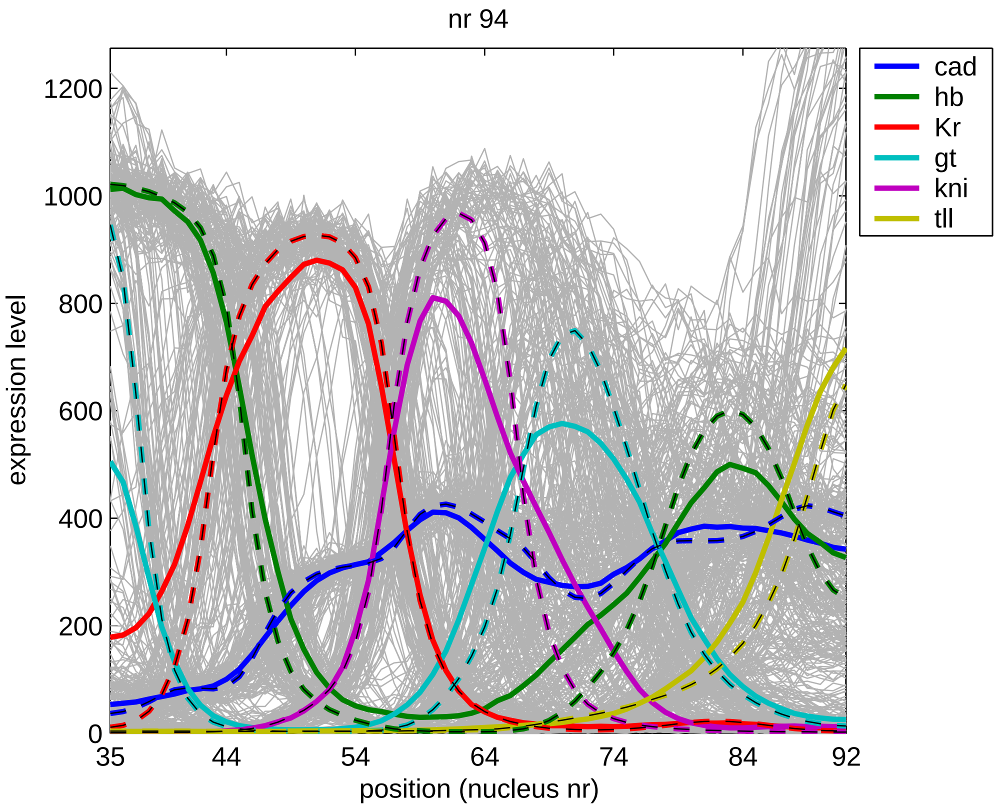

nr 94
expression level
position (nucleus nr)
0
200
400
600
800
1000
1200
35
44
54
64
74
84
92
cad
hb
Kr
gt
kni
tll

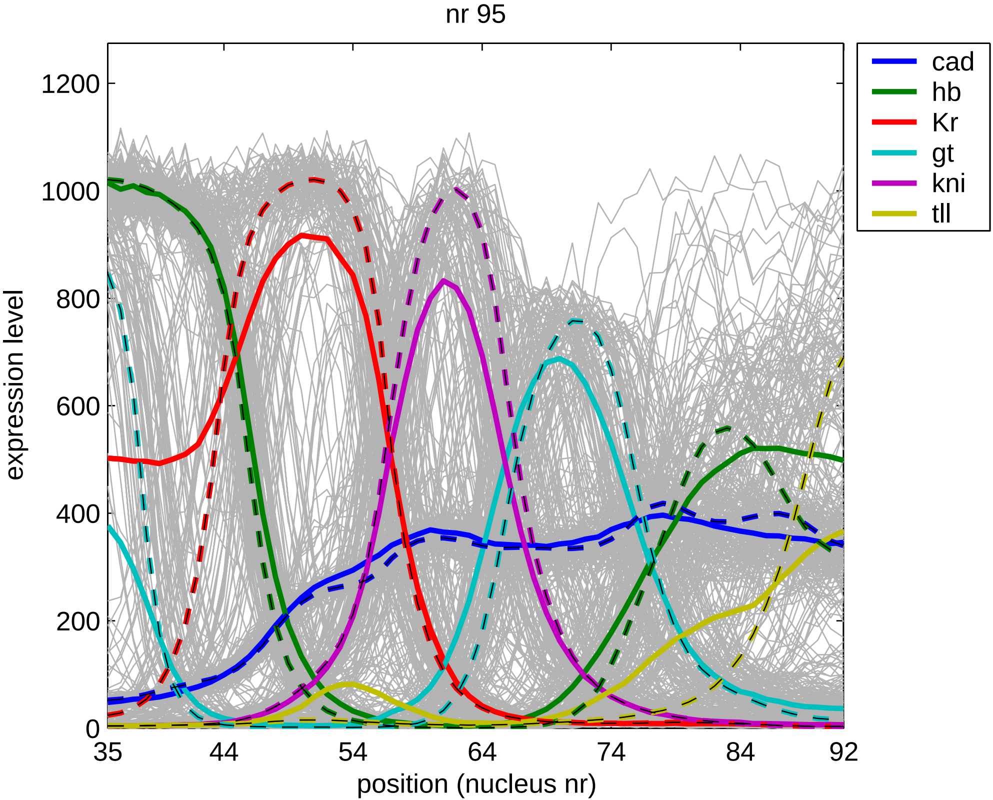
nr 95
cad
hb
Kr
gt
kni
tll
expression level
0
200
400
600
800
1000
1200
35
44
54
64
74
84
92
position (nucleus nr)

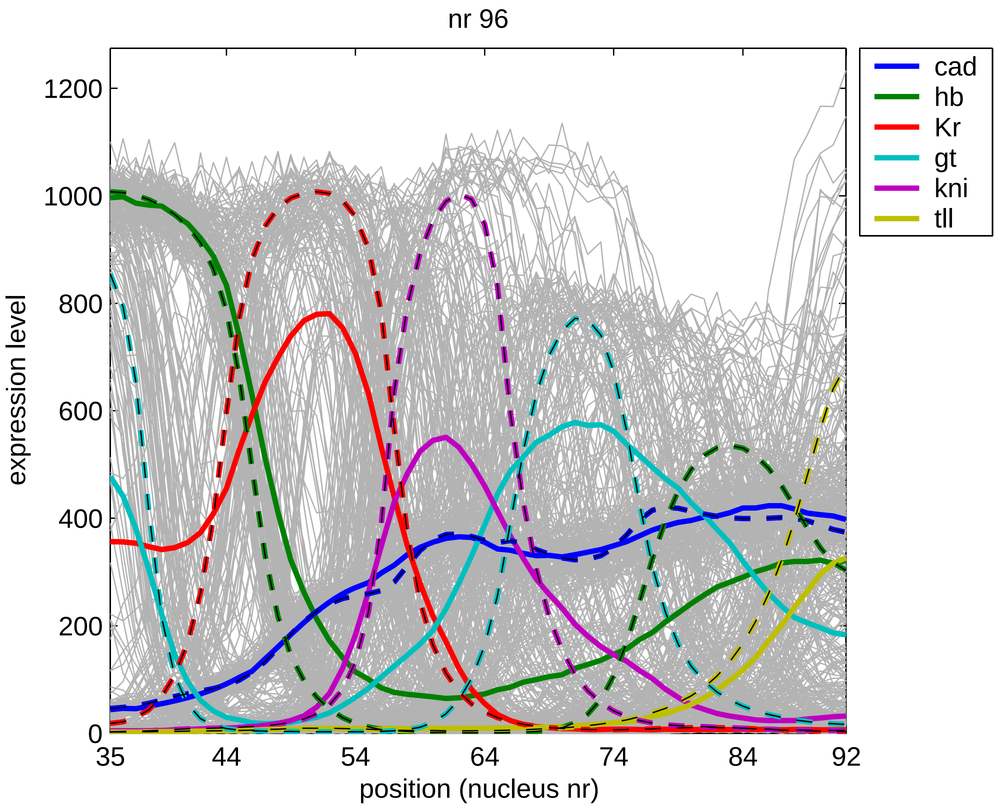

nr 96
expression level
position (nucleus nr)
0
200
400
600
800
1000
1200
35
44
54
64
74
84
92
cad
hb
Kr
gt
kni
tll

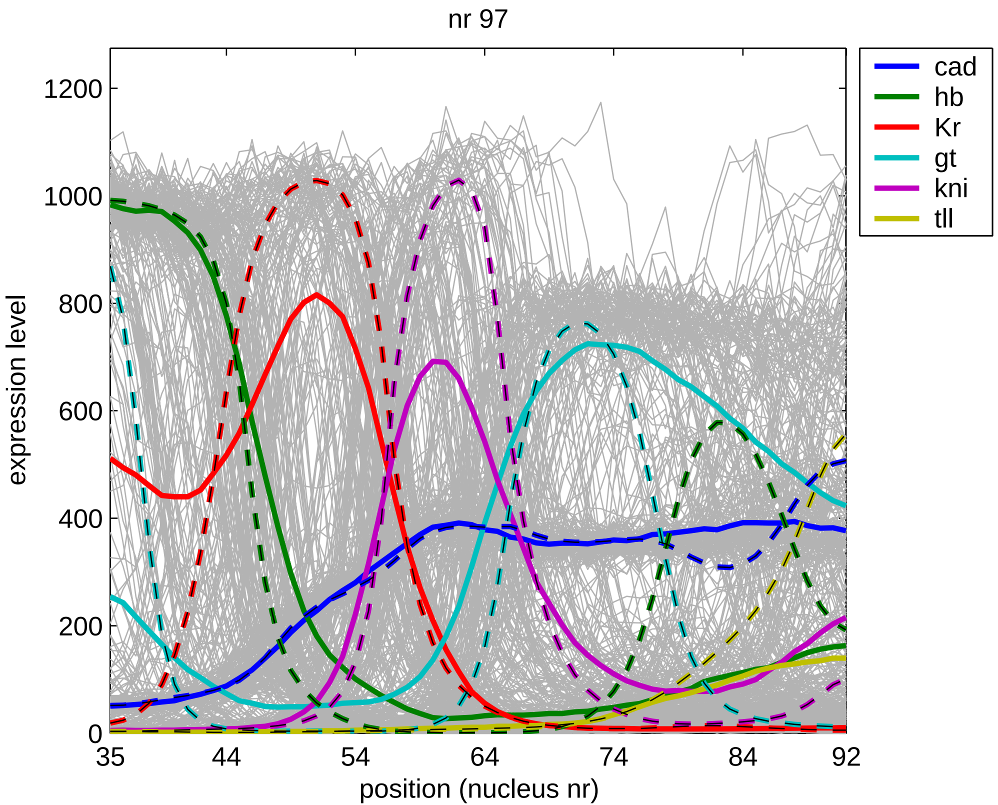

nr 97
expression level
position (nucleus nr)
0
200
400
600
800
1000
1200
35
44
54
64
74
84
92
cad
hb
Kr
gt
kni
tll

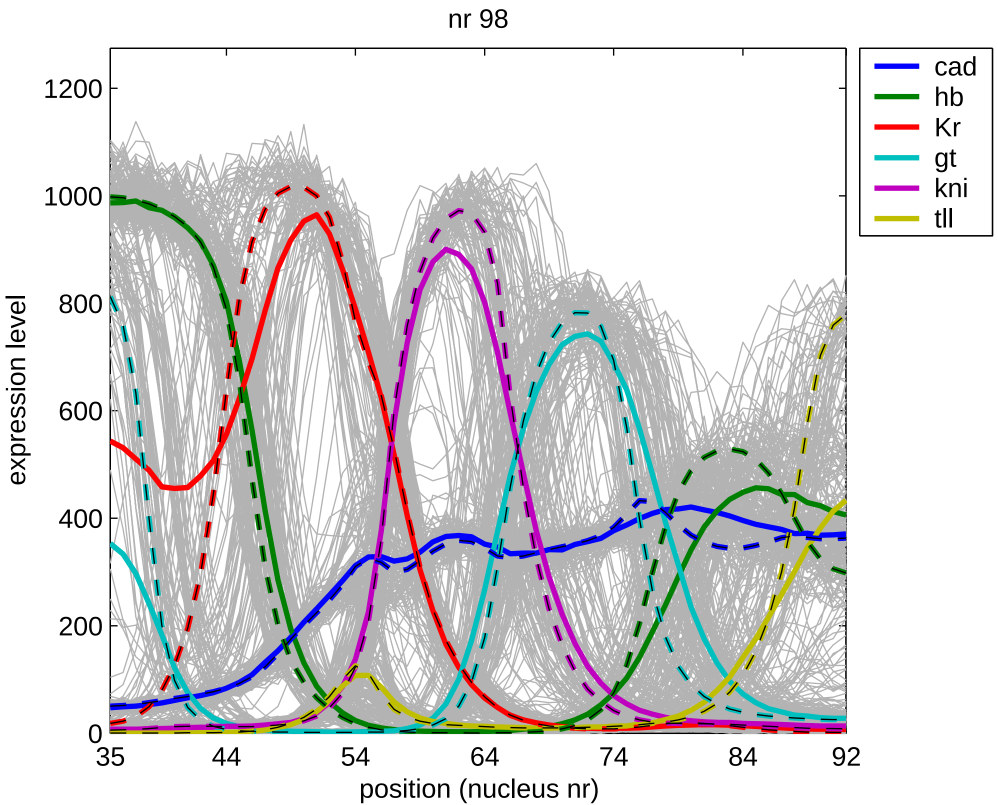

nr 98
expression level
position (nucleus nr)
1200
1000
800
600
400
200
0
35
44
54
64
74
84
92
cad
hb
Kr
gt
kni
tll

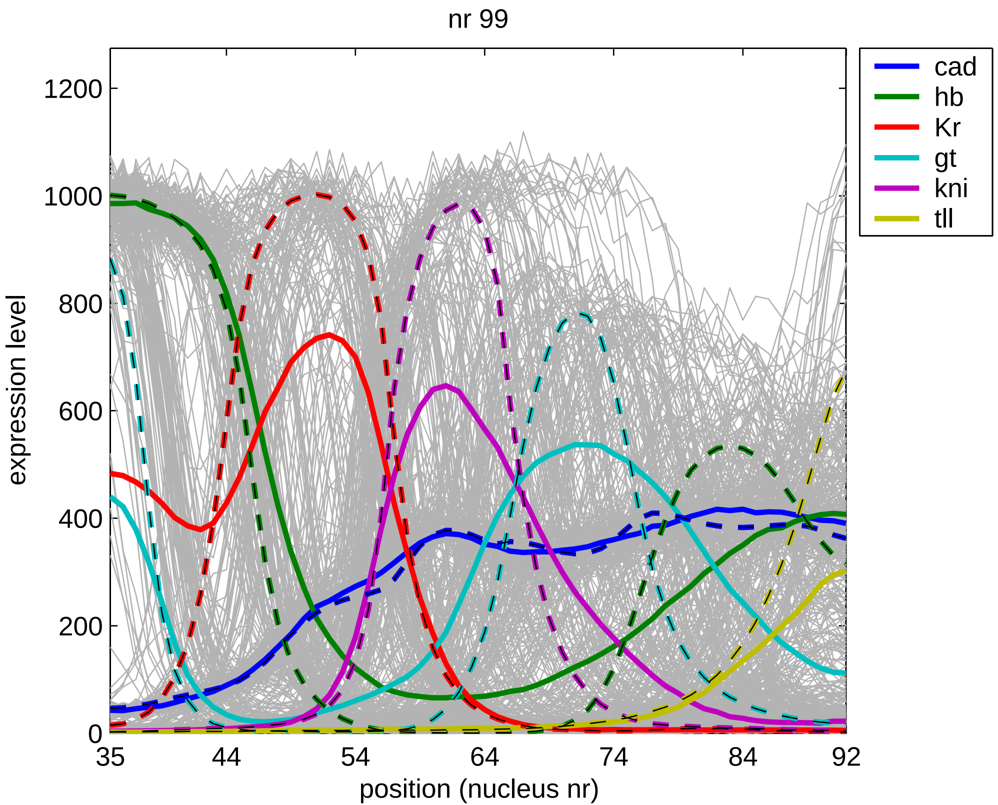

nr 99
expression level
position (nucleus nr)
0
200
400
600
800
1000
1200
35
44
54
64
74
84
92
cad
hb
Kr
gt
kni
tll

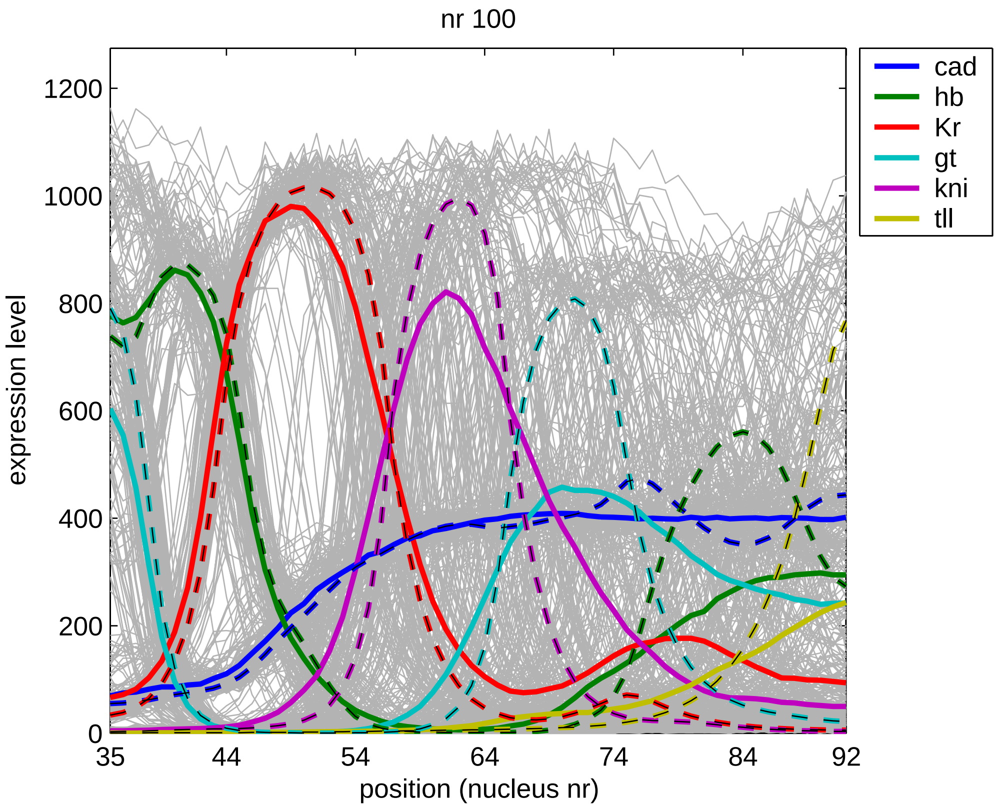
nr 100
expression level
position (nucleus nr)
0
200
400
600
800
1000
1200
35
44
54
64
74
84
92
cad
hb
Kr
gt
kni
tll

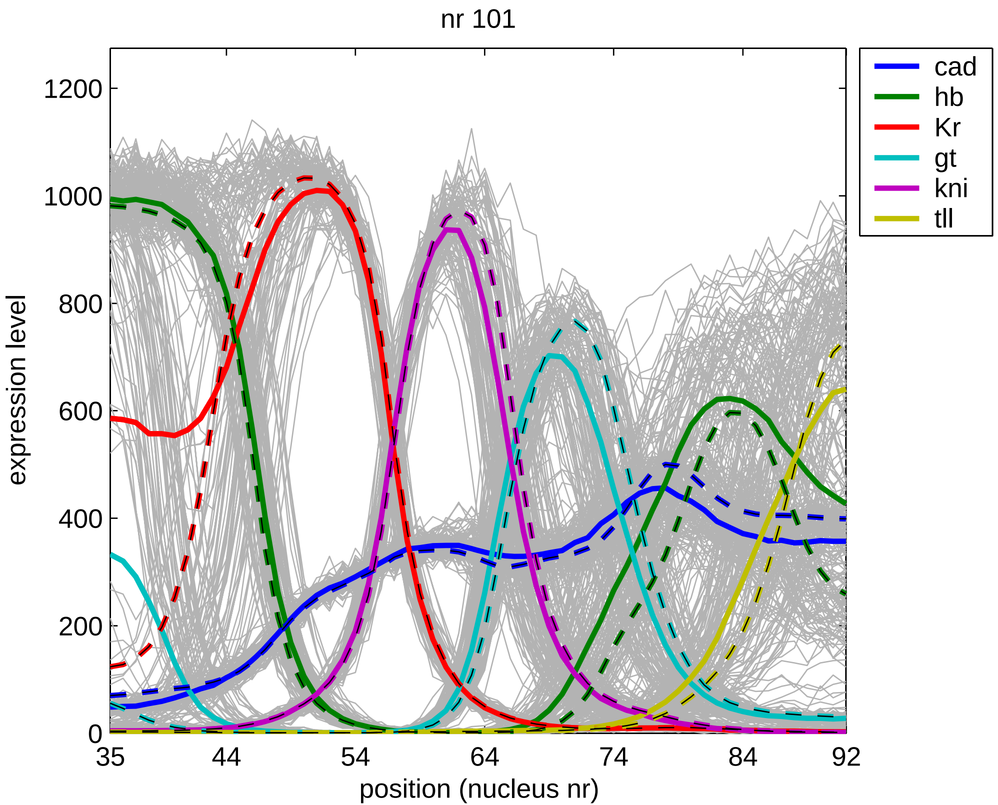

nr 101
expression level
1200
1000
800
600
400
200
0
35
44
54
64
74
84
92
position (nucleus nr)
cad
hb
Kr
gt
kni
tll
