# Supplementary material for: Inferring Drosophila gap gene regulatory network: a parameter sensitivity and perturbation analysis
Source: BMC Syst Biol. 2009 Sep 21;3:94. doi: 10.1186/1752-0509-3-94 (PMC2761871; doi:10.1186/1752-0509-3-94)
Supplement: Additional file 8 — Absolute parameters sensitivity intervals of all parameters. The data provided in this document (GapGeneModelRobustnessAddFile8.pdf) represent the sensitivity interval of all the 66 parameters obtained from the 101 circuits. Each figure gives the upper and lower bound of the SI. [file 1752-0509-3-94-S8.PDF]

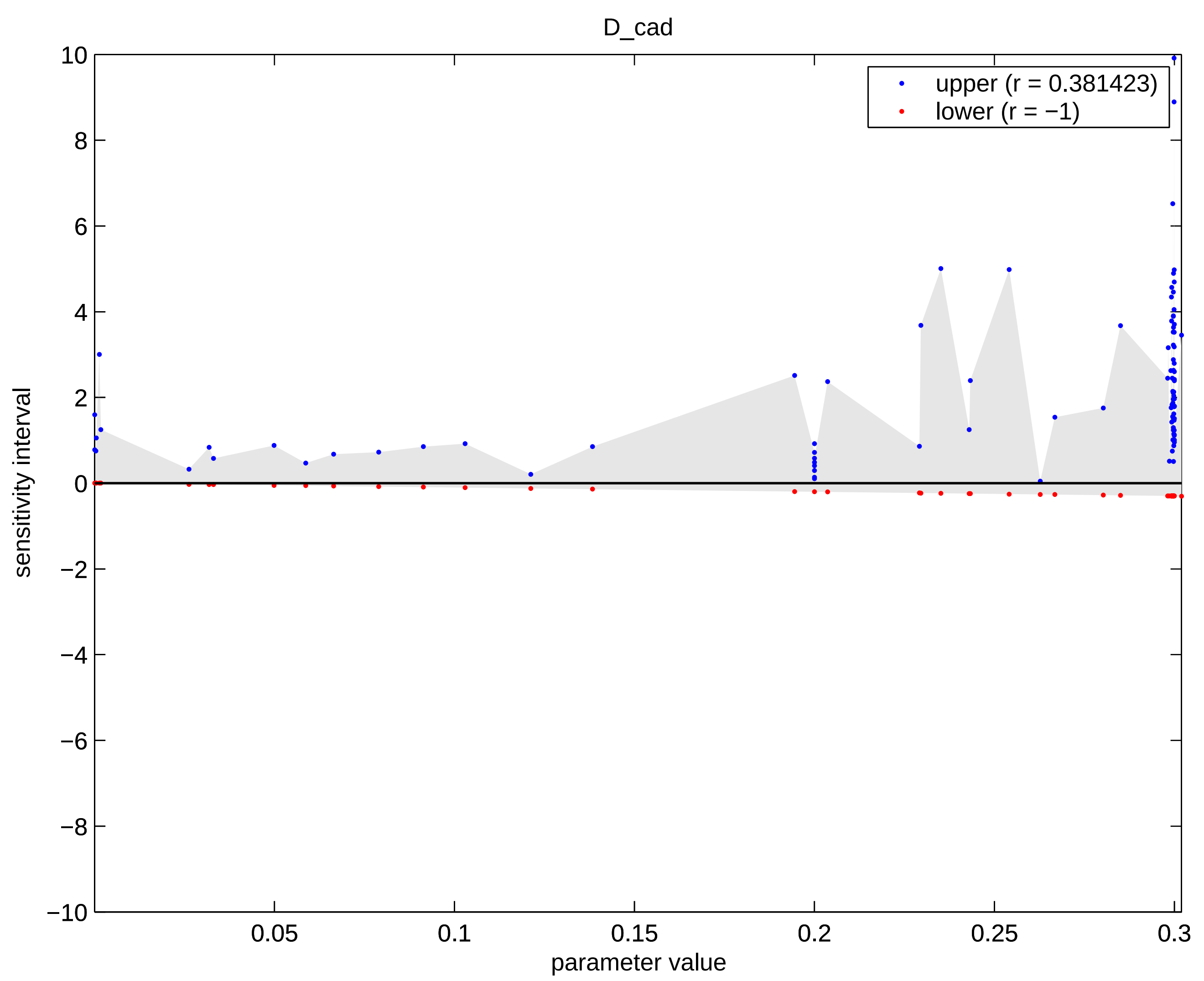

D_cad
upper (r = 0.381423)
lower (r = −1)
sensitivity interval
parameter value
10
8
6
4
2
0
−2
−4
−6
−8
−10
0.05
0.1
0.15
0.2
0.25
0.3

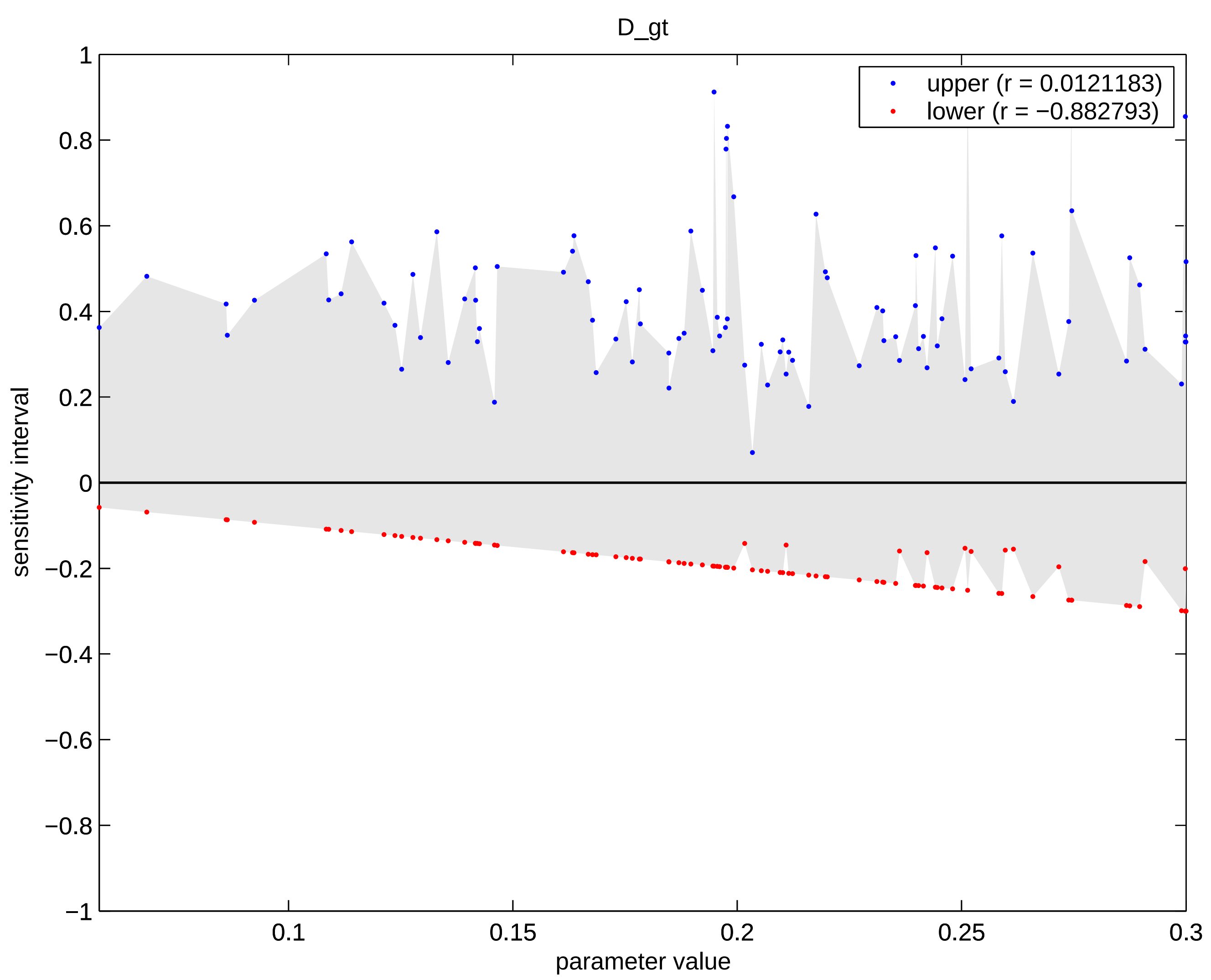
D_gt
upper (r = 0.0121183)
lower (r = −0.882793)
sensitivity interval
parameter value
1
0.8
0.6
0.4
0.2
0
−0.2
−0.4
−0.6
−0.8
−1
0.1
0.15
0.2
0.25
0.3

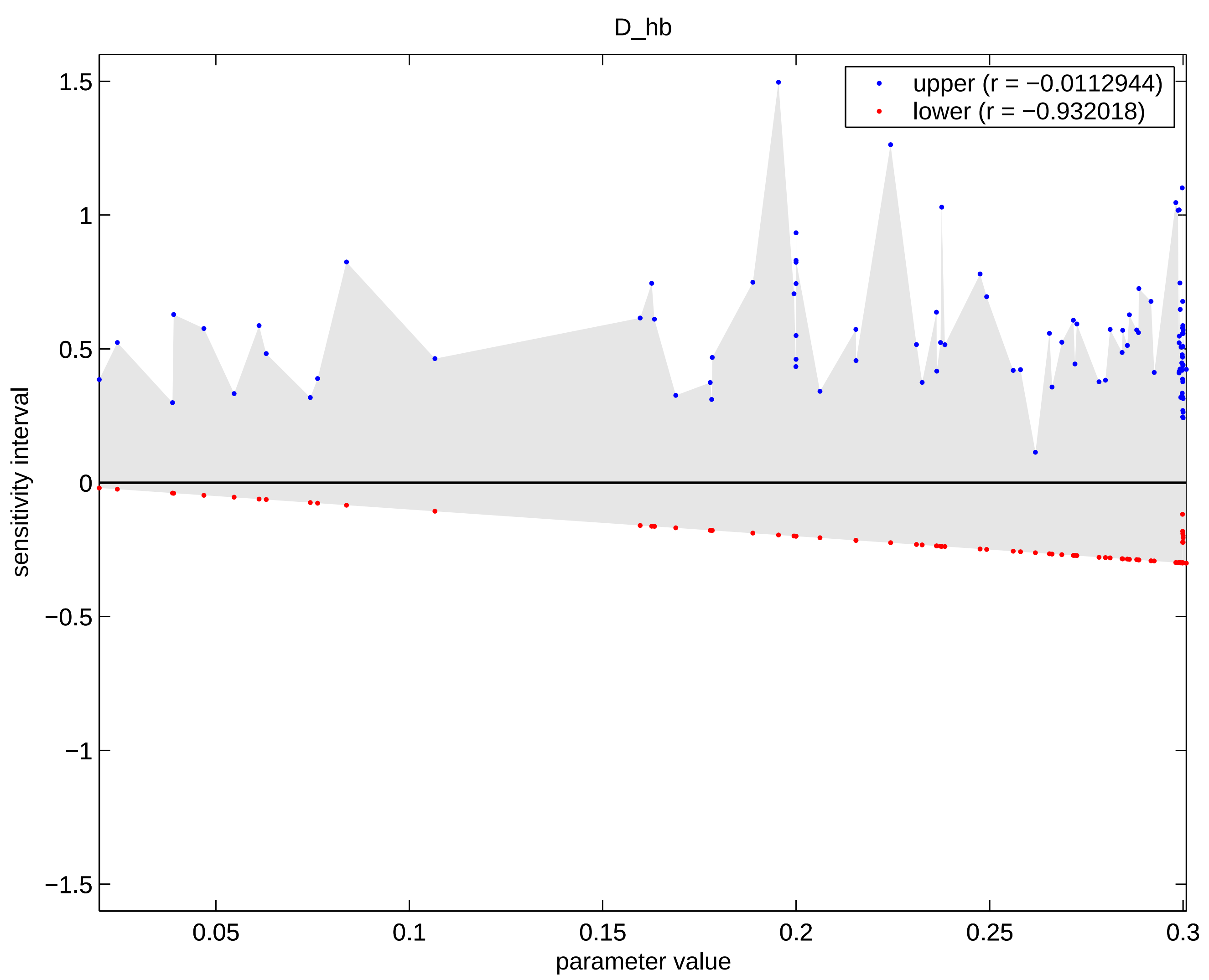
D_hb
upper (r = −0.0112944)
lower (r = −0.932018)
sensitivity interval
parameter value
1.5
1
0.5
0
−0.5
−1
−1.5
0.05
0.1
0.15
0.2
0.25
0.3

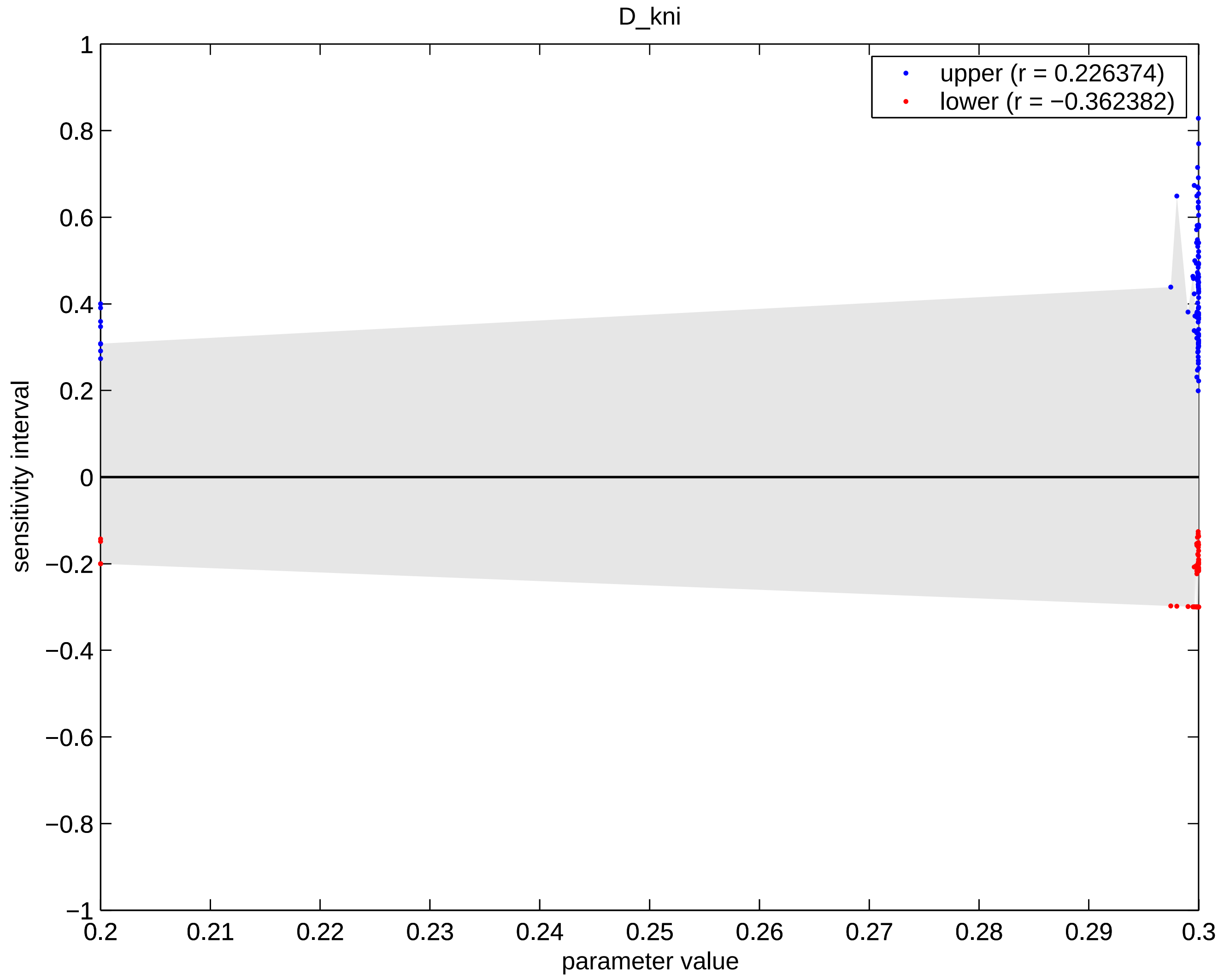

D_kni
upper (r = 0.226374)
lower (r = −0.362382)
sensitivity interval
parameter value
1
0.8
0.6
0.4
0.2
0
−0.2
−0.4
−0.6
−0.8
−1
0.2
0.21
0.22
0.23
0.24
0.25
0.26
0.27
0.28
0.29
0.3

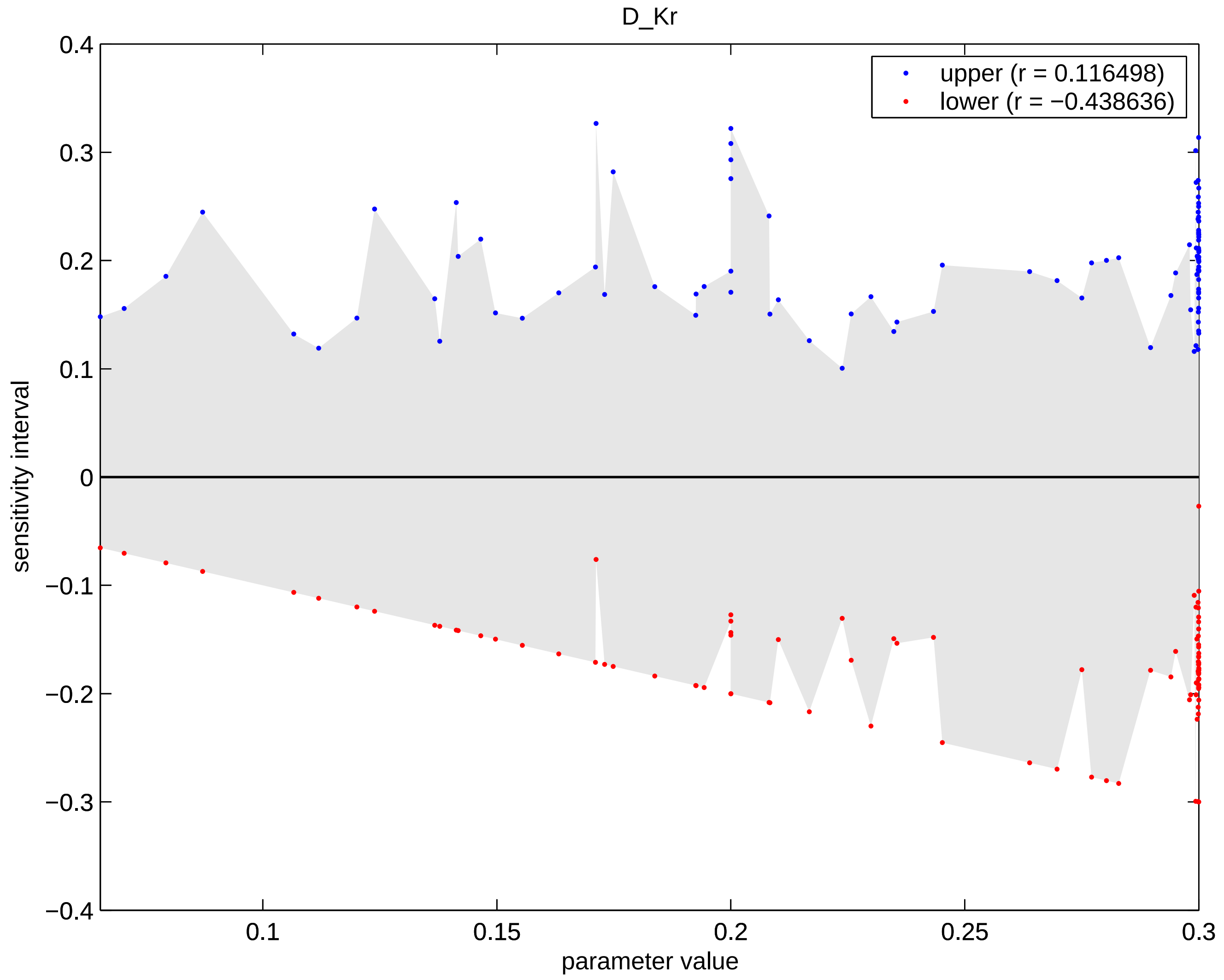

D_Kr
upper (r = 0.116498)
lower (r = −0.438636)
sensitivity interval
parameter value
0.4
0.3
0.2
0.1
0
−0.1
−0.2
−0.3
−0.4
0.1
0.15
0.2
0.25
0.3

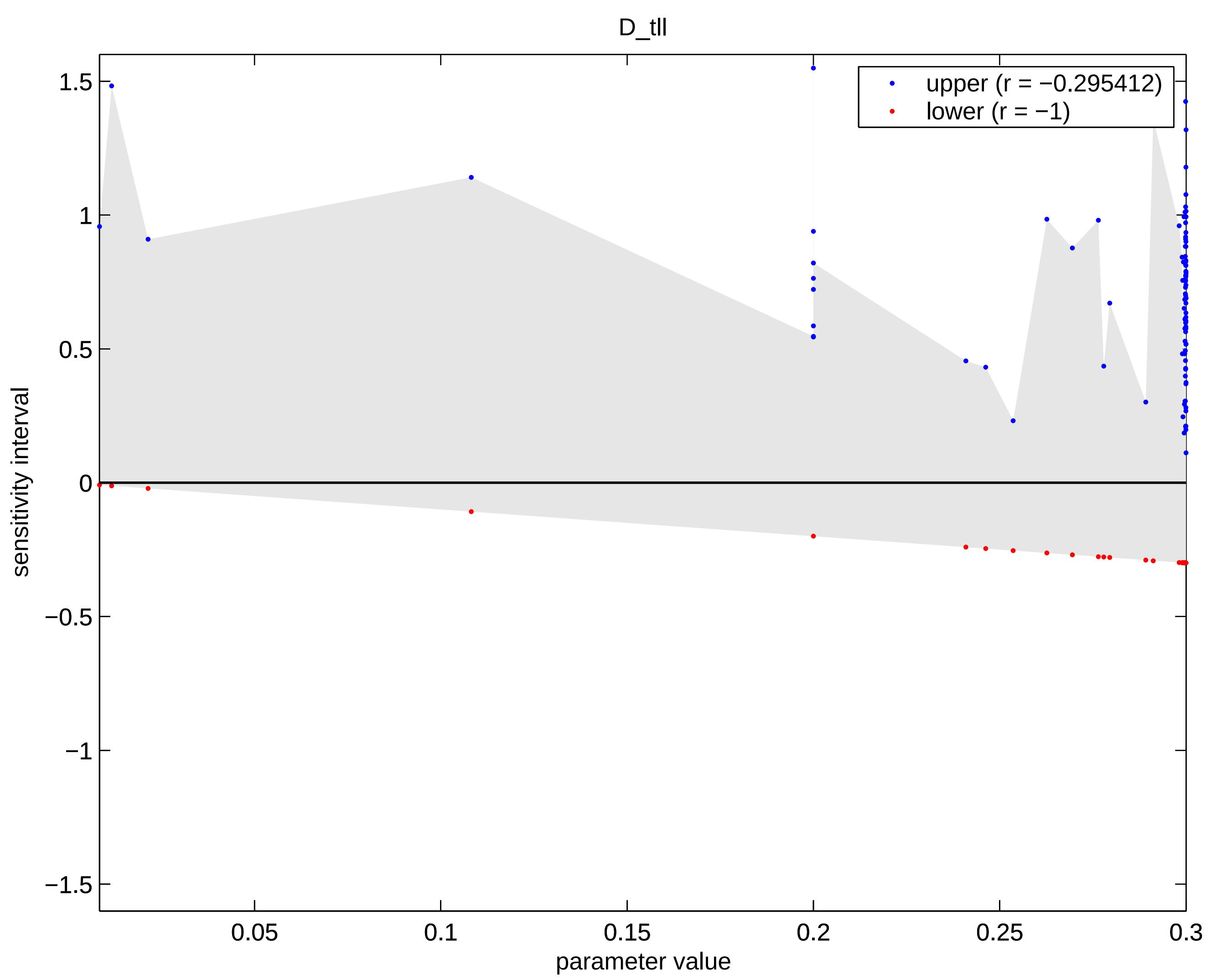
D_tll
upper (r = −0.295412)
lower (r = −1)
sensitivity interval
parameter value
1.5
1
0.5
0
−0.5
−1
−1.5
0.05
0.1
0.15
0.2
0.25
0.3

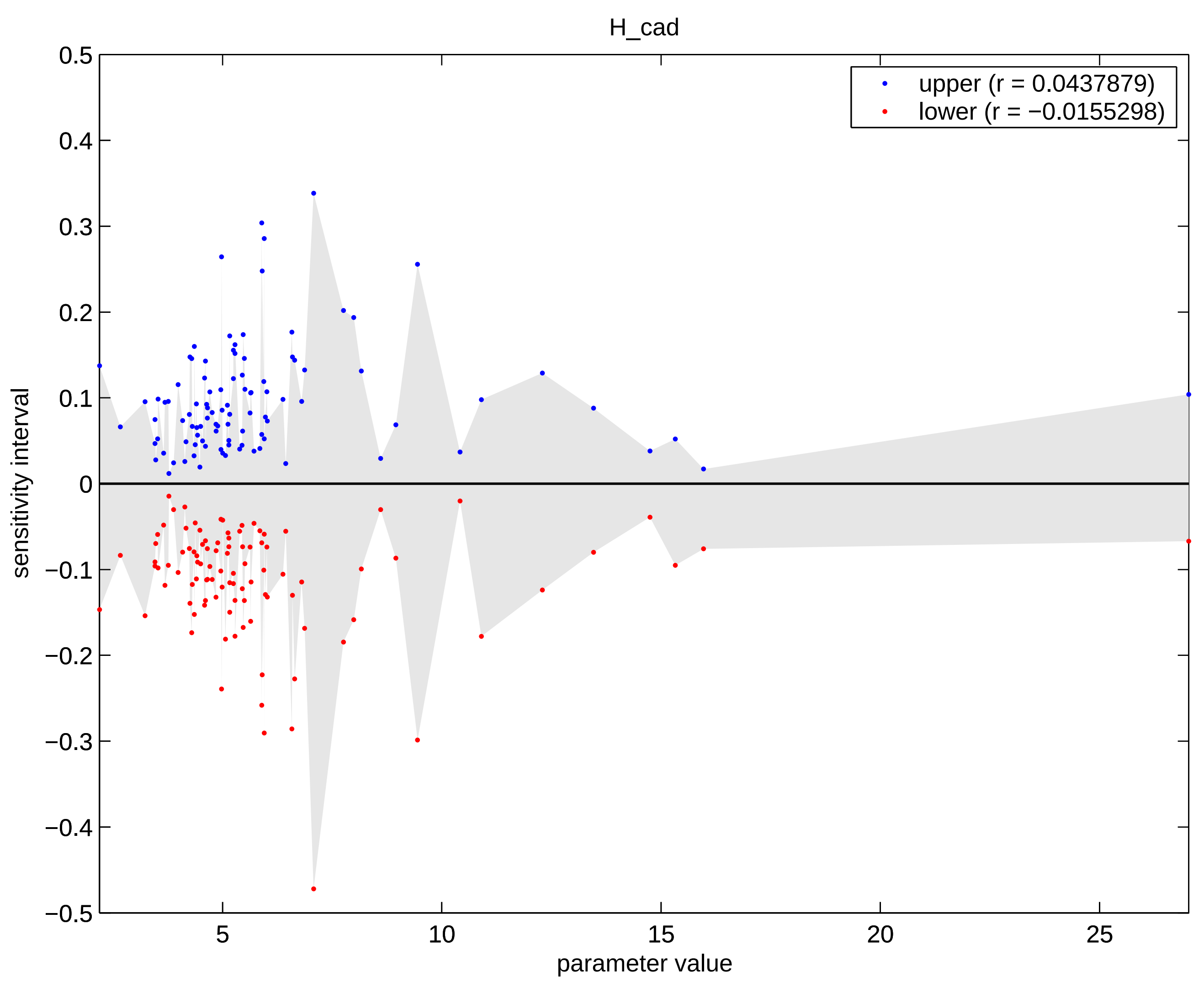

H_cad
upper (r = 0.0437879)
lower (r = −0.0155298)
sensitivity interval
parameter value
0.5
0.4
0.3
0.2
0.1
0
−0.1
−0.2
−0.3
−0.4
−0.5
5
10
15
20
25

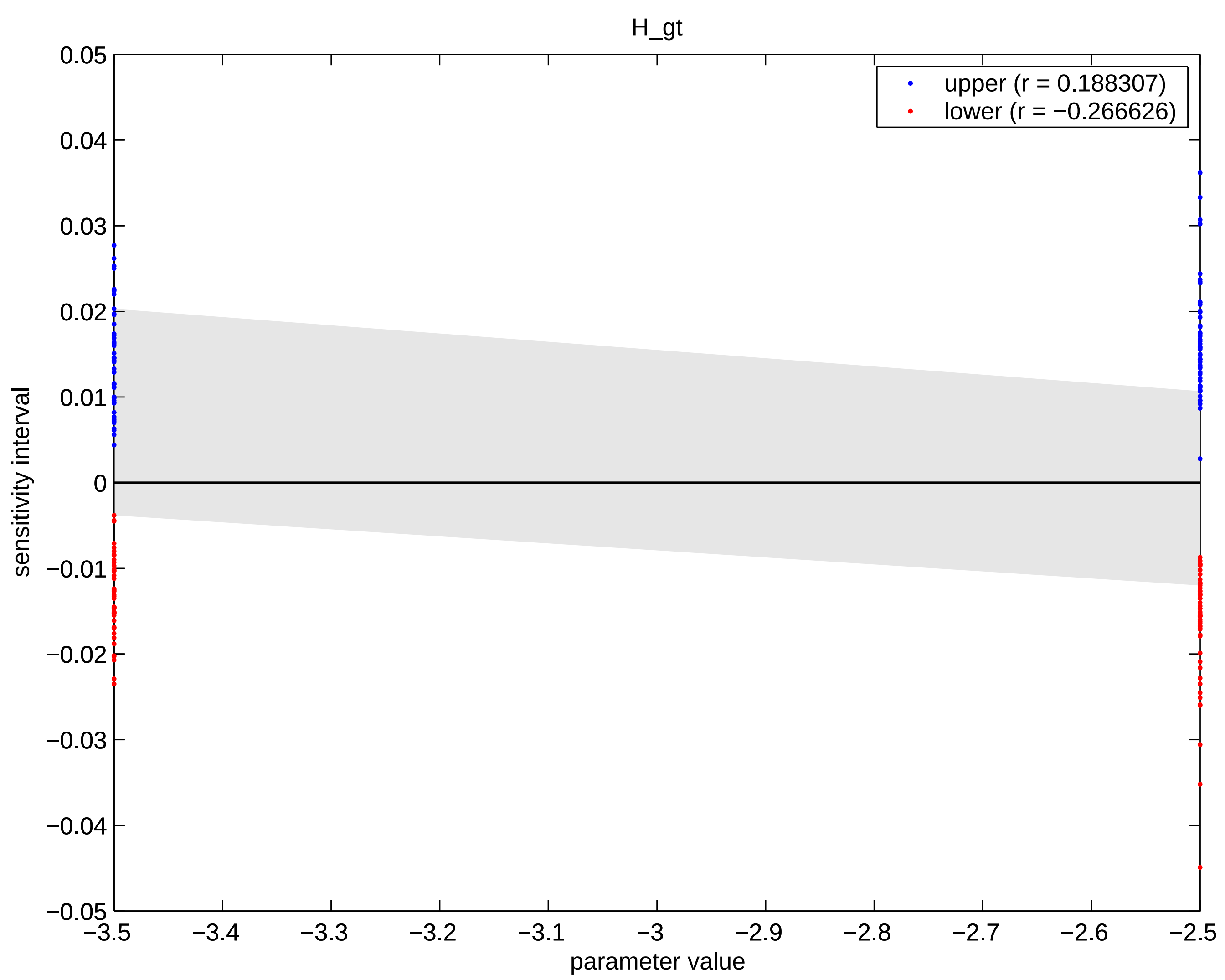

H_gt
upper (r = 0.188307)
lower (r = −0.266626)
0.05
0.04
0.03
0.02
0.01
0
−0.01
−0.02
−0.03
−0.04
−0.05
sensitivity interval
−3.5
−3.4
−3.3
−3.2
−3.1
−3
−2.9
−2.8
−2.7
−2.6
−2.5
parameter value

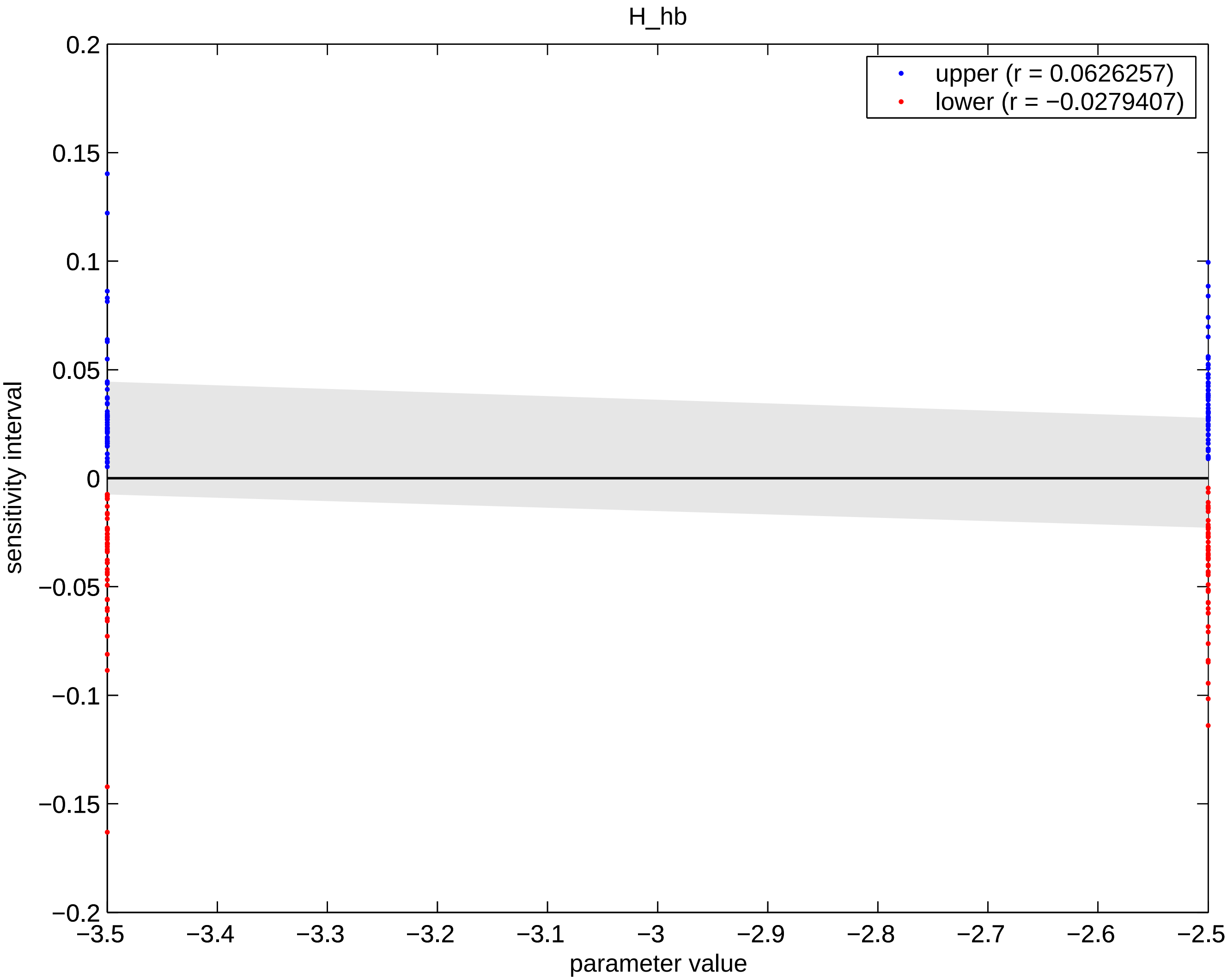
H_hb
upper (r = 0.0626257)
lower (r = −0.0279407)
sensitivity interval
parameter value
0.2
0.15
0.1
0.05
0
−0.05
−0.1
−0.15
−0.2
−3.5
−3.4
−3.3
−3.2
−3.1
−3
−2.9
−2.8
−2.7
−2.6
−2.5

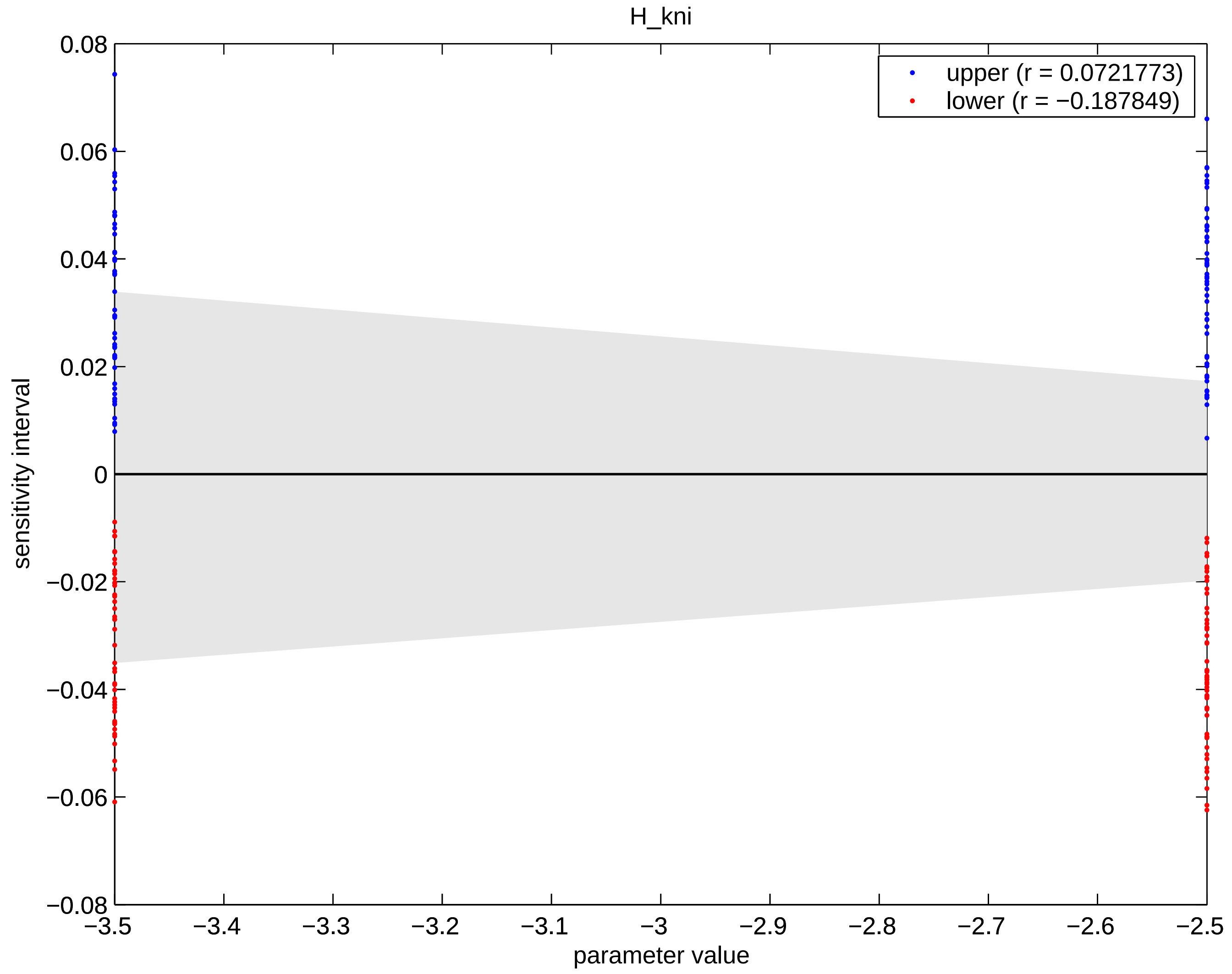

H_kni
upper (r = 0.0721773)
lower (r = −0.187849)
sensitivity interval
parameter value
0.08
0.06
0.04
0.02
0
−0.02
−0.04
−0.06
−0.08
−3.5
−3.4
−3.3
−3.2
−3.1
−3
−2.9
−2.8
−2.7
−2.6
−2.5

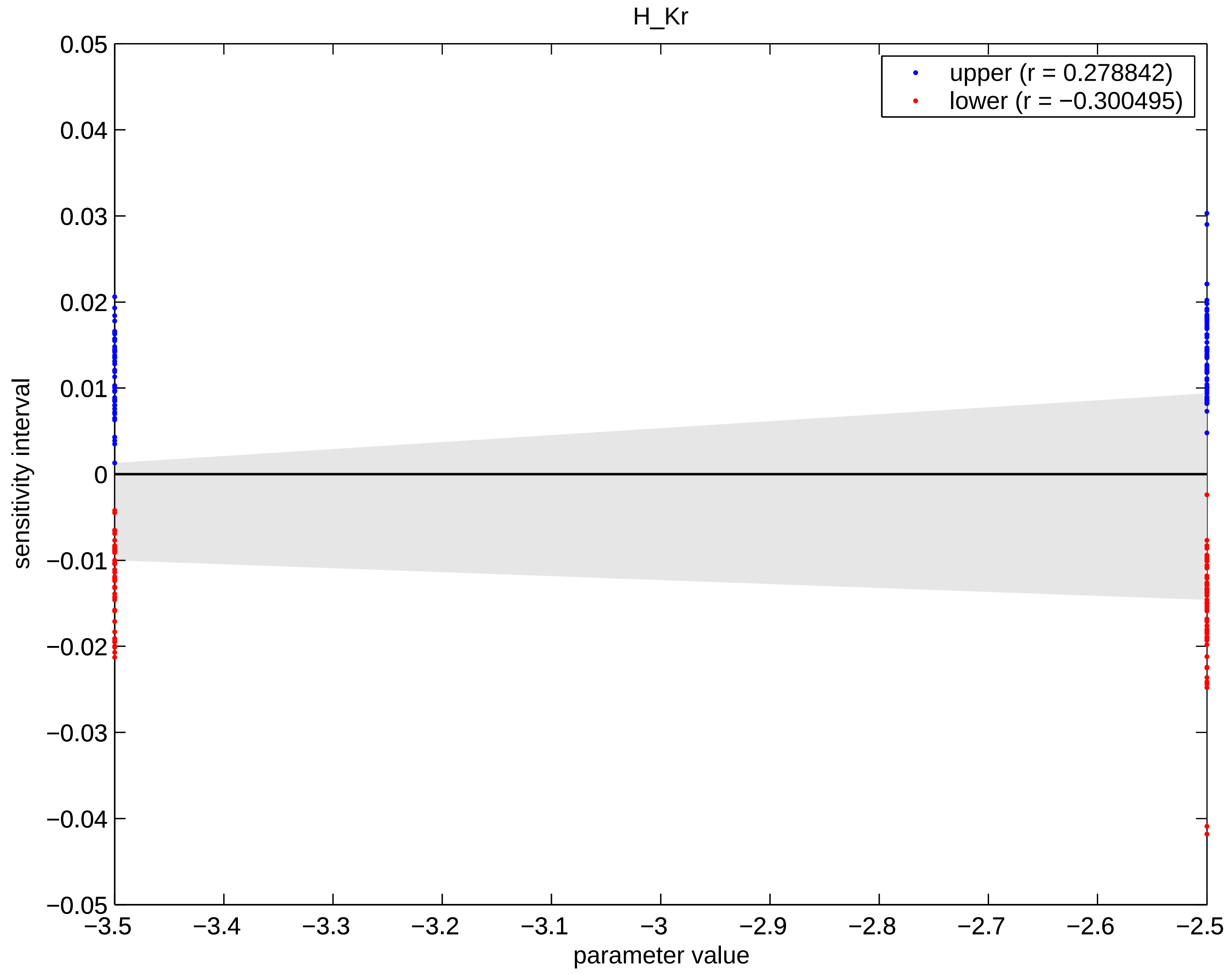

H_Kr
upper (r = 0.278842)
lower (r = −0.300495)
0.05
0.04
0.03
0.02
0.01
0
−0.01
−0.02
−0.03
−0.04
−0.05
sensitivity interval
−3.5
−3.4
−3.3
−3.2
−3.1
−3
−2.9
−2.8
−2.7
−2.6
−2.5
parameter value

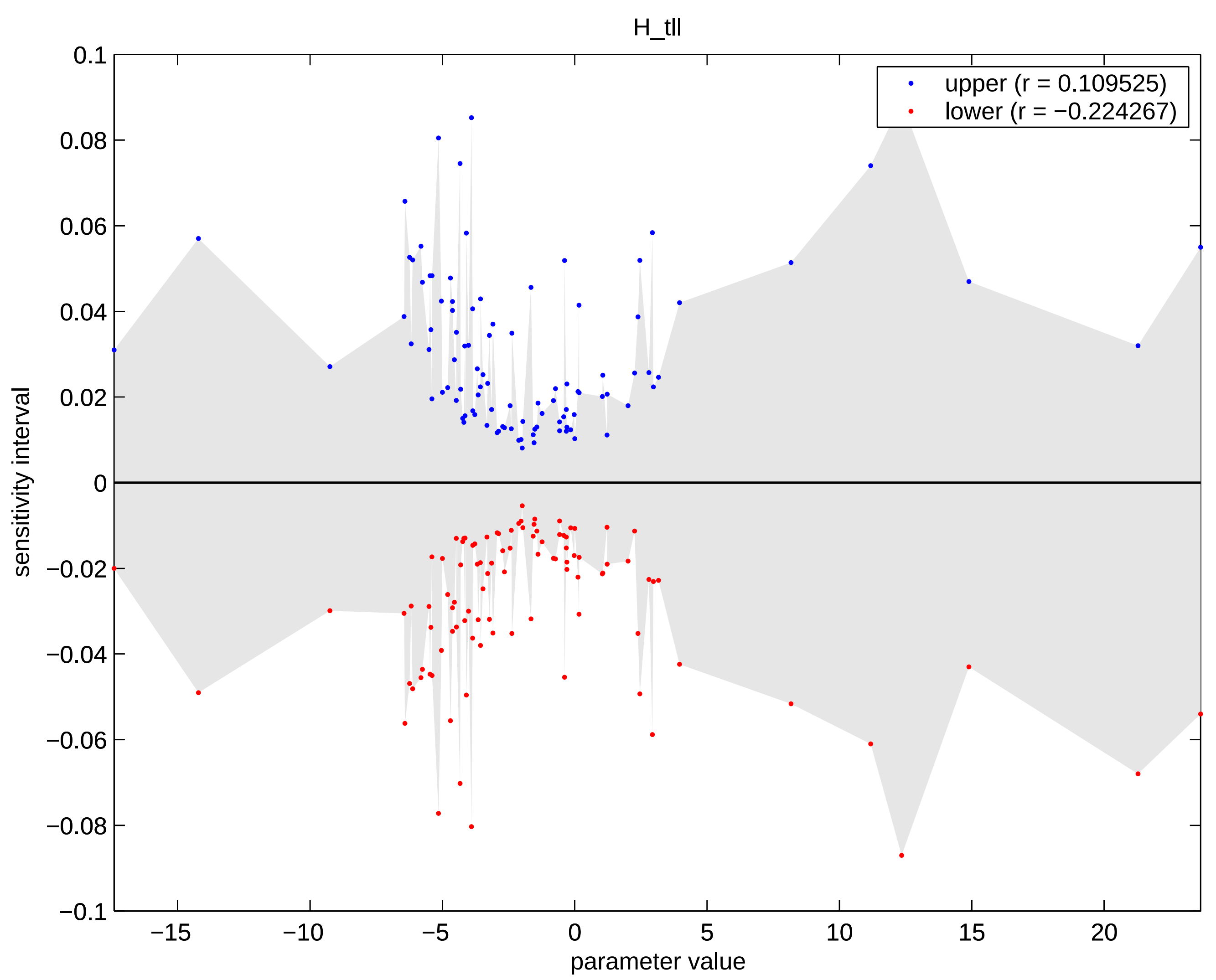
H_tll
upper (r = 0.109525)
lower (r = −0.224267)
sensitivity interval
parameter value
0.1
0.08
0.06
0.04
0.02
0
−0.02
−0.04
−0.06
−0.08
−0.1
−15
−10
−5
0
5
10
15
20

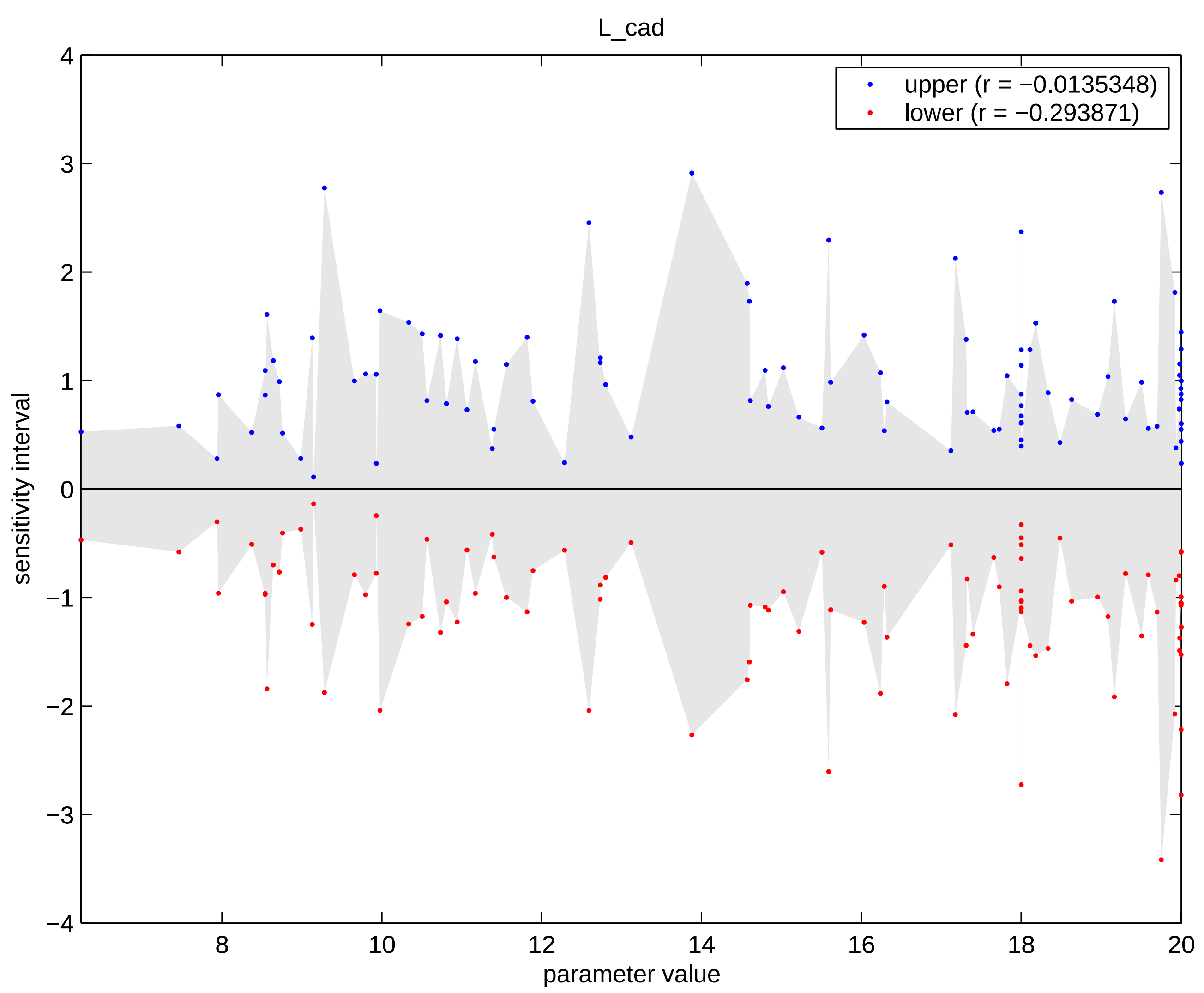

L_cad
upper (r = −0.0135348)
lower (r = −0.293871)
sensitivity interval
parameter value
4
3
2
1
0
−1
−2
−3
−4
8
10
12
14
16
18
20

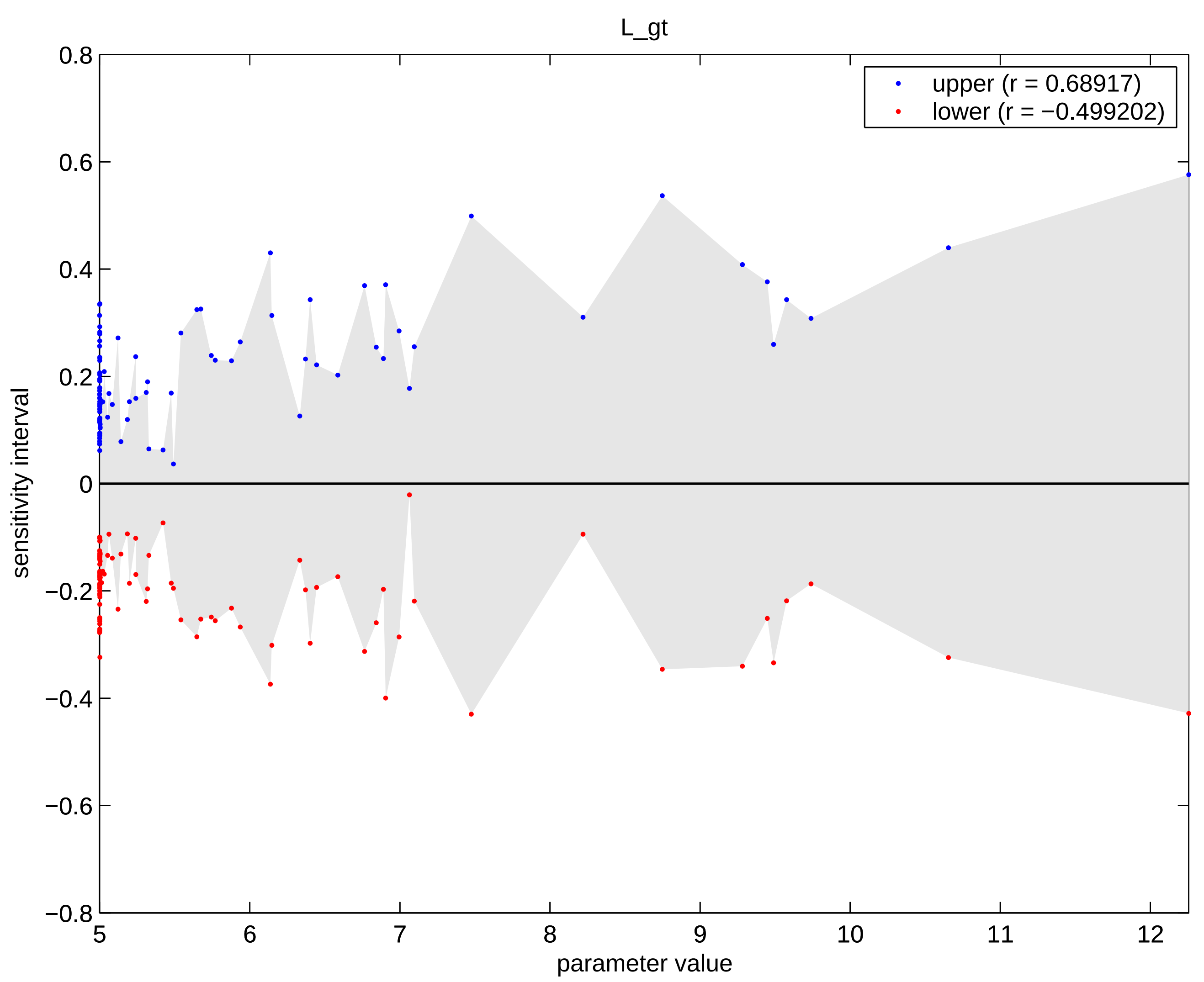
L_gt
upper (r = 0.68917)
lower (r = −0.499202)
sensitivity interval
parameter value
0.8
0.6
0.4
0.2
0
−0.2
−0.4
−0.6
−0.8
5
6
7
8
9
10
11
12

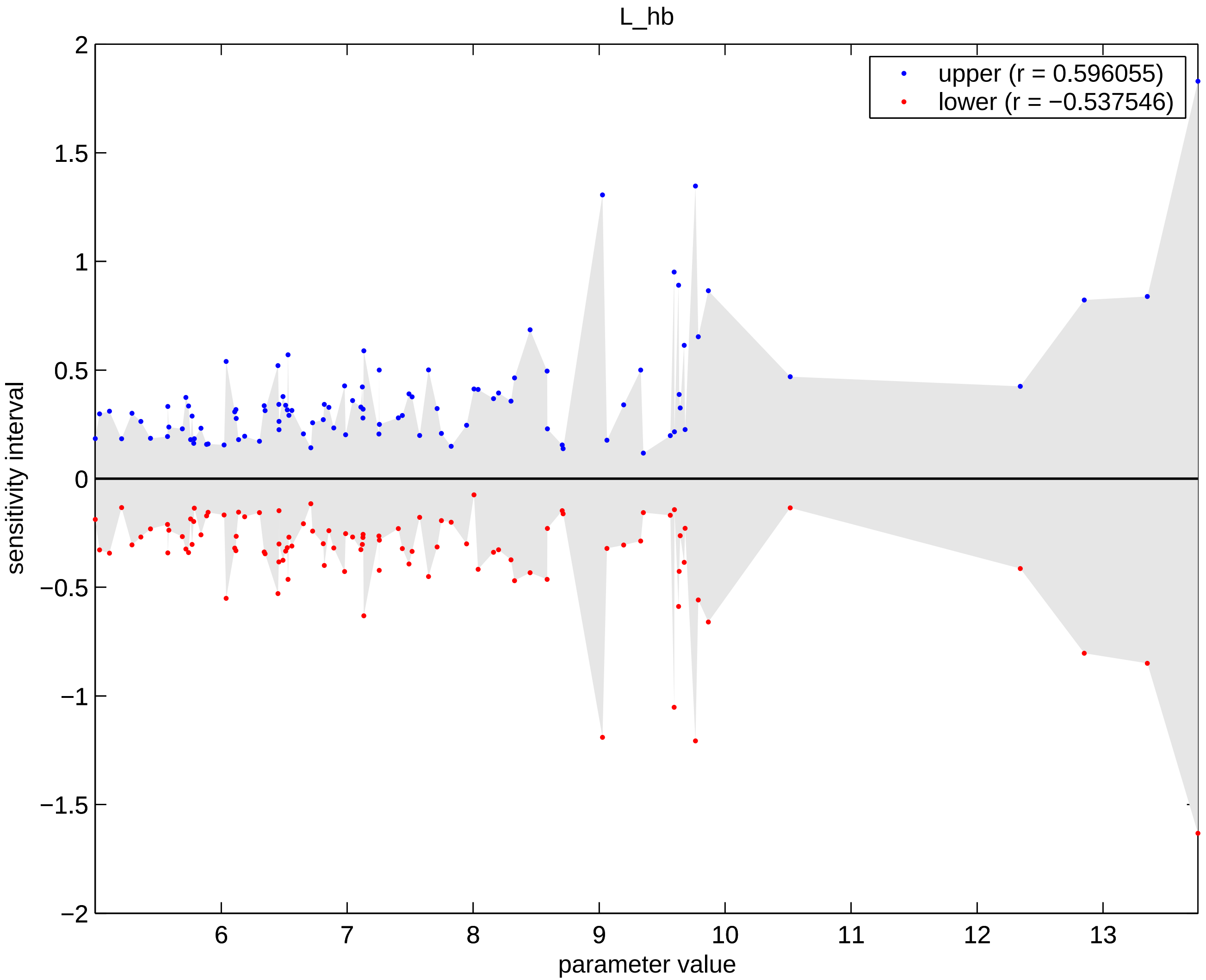

L_hb
upper (r = 0.596055)
lower (r = −0.537546)
sensitivity interval
parameter value
2
1.5
1
0.5
0
−0.5
−1
−1.5
−2
6
7
8
9
10
11
12
13

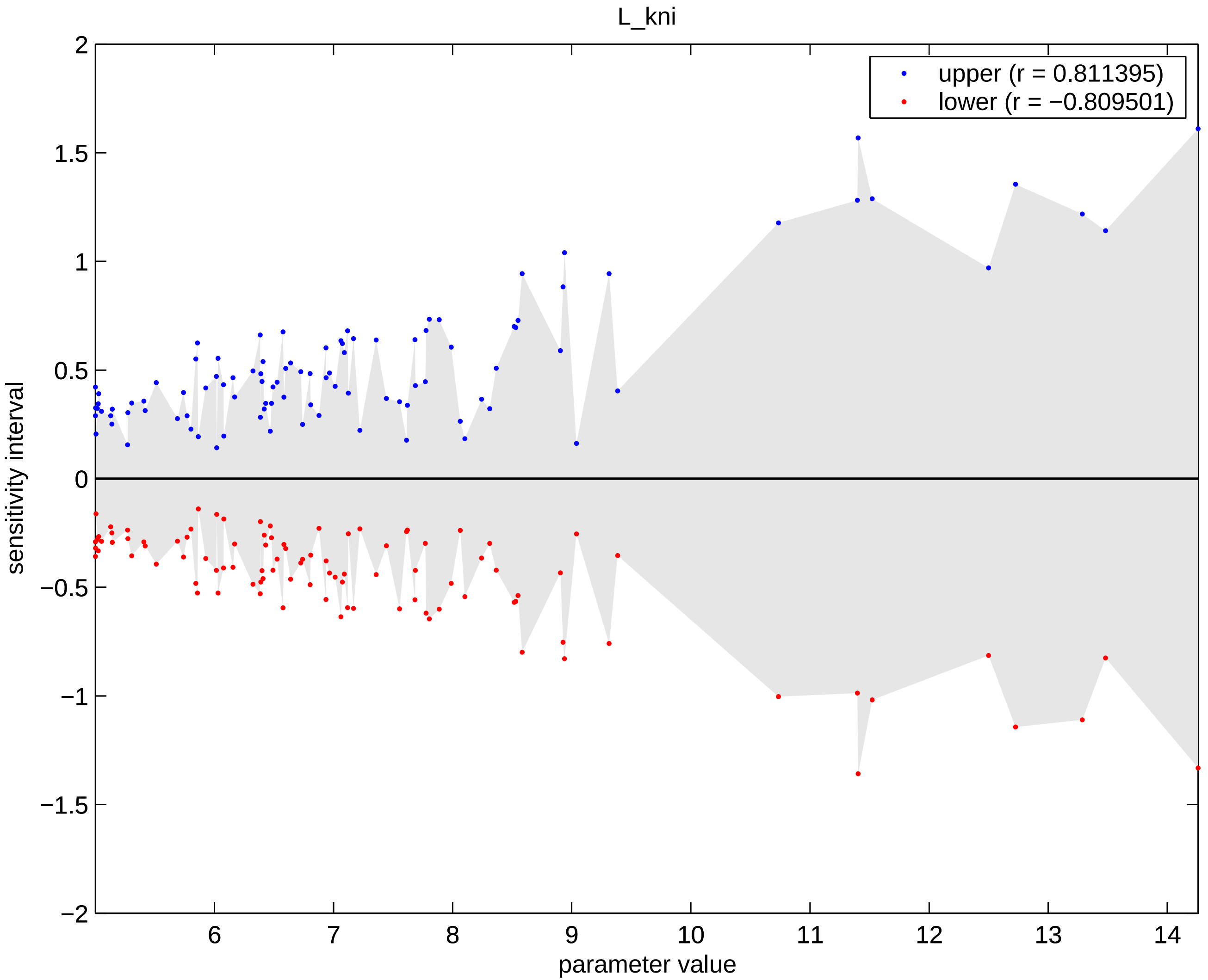

L_kni
upper (r = 0.811395)
lower (r = −0.809501)
sensitivity interval
parameter value
2
1.5
1
0.5
0
−0.5
−1
−1.5
−2
6
7
8
9
10
11
12
13
14

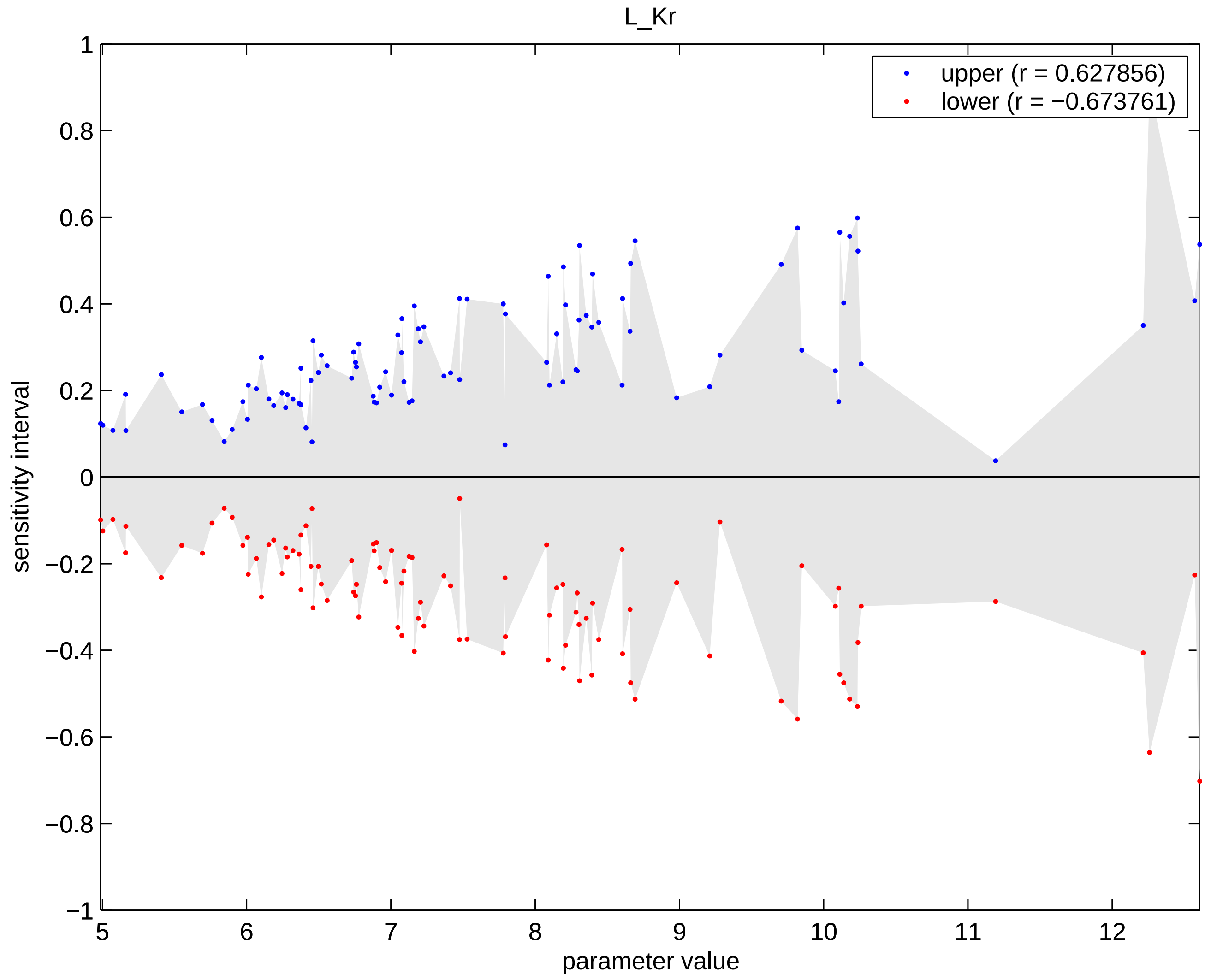
L_Kr
upper (r = 0.627856)
lower (r = −0.673761)
sensitivity interval
parameter value
1
0.8
0.6
0.4
0.2
0
−0.2
−0.4
−0.6
−0.8
−1
5
6
7
8
9
10
11
12

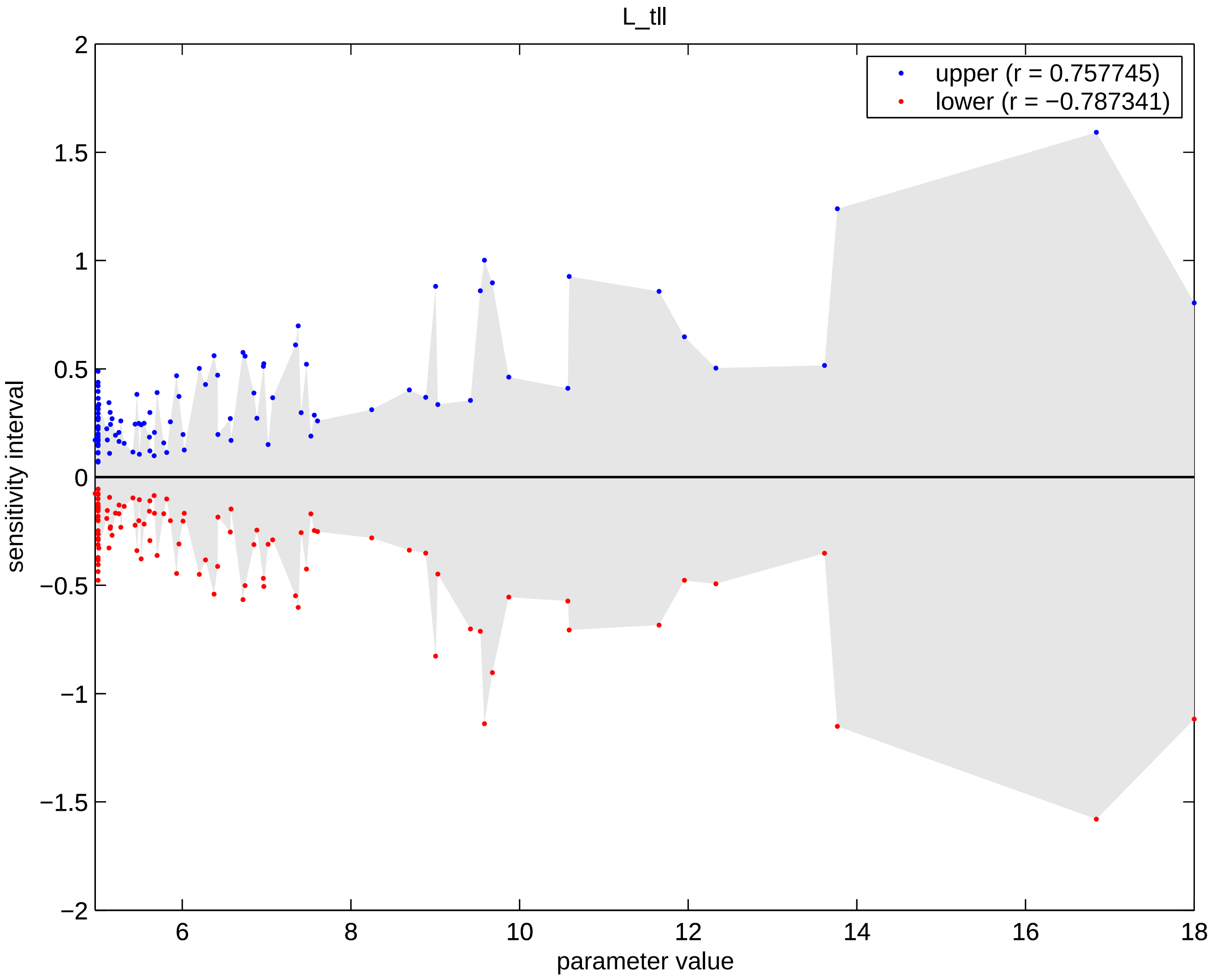

L_tll
upper (r = 0.757745)
lower (r = −0.787341)
sensitivity interval
parameter value
2
1.5
1
0.5
0
−0.5
−1
−1.5
−2
6
8
10
12
14
16
18

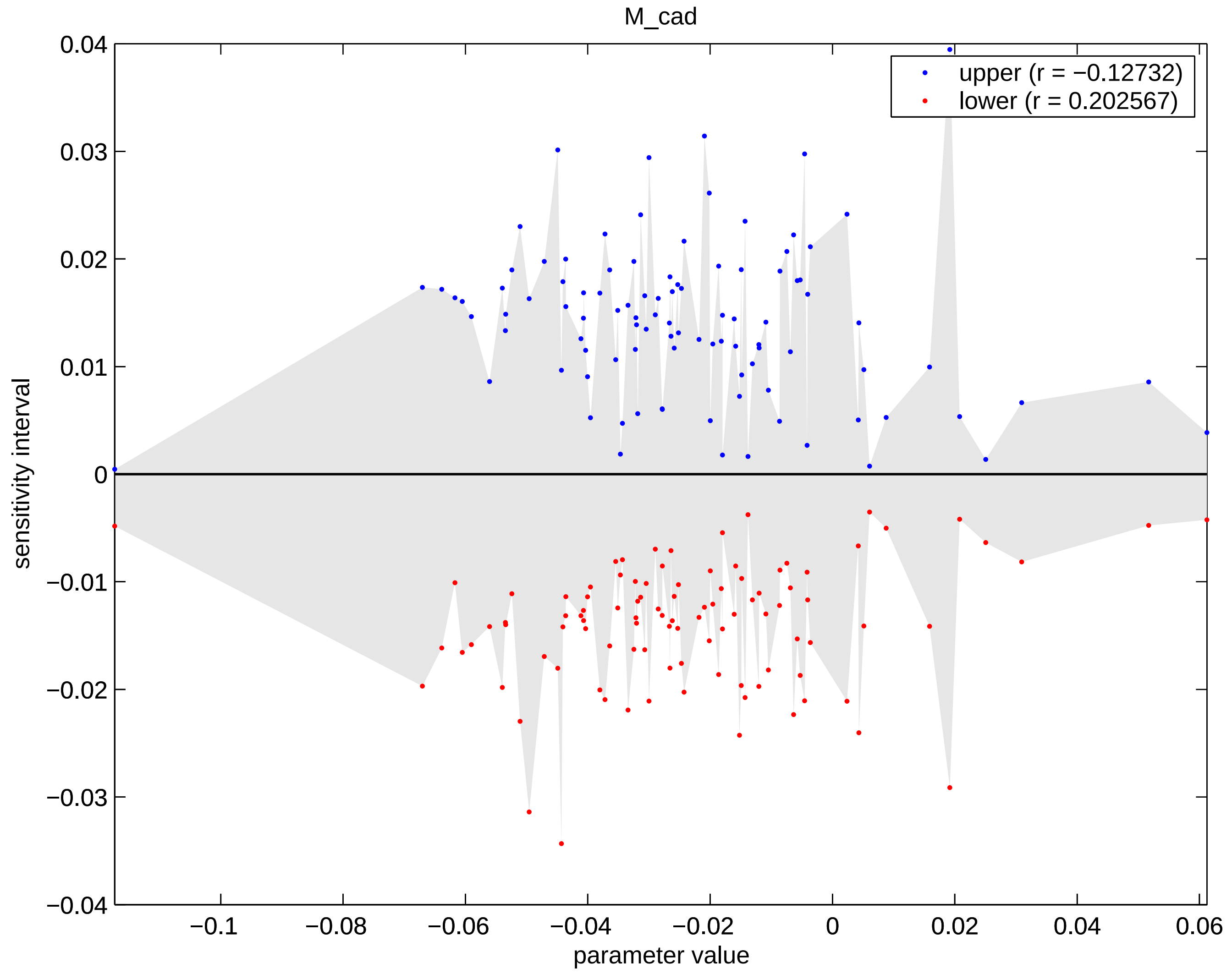
M_cad
upper (r = −0.12732)
lower (r = 0.202567)
sensitivity interval
parameter value
0.04
0.03
0.02
0.01
0
−0.01
−0.02
−0.03
−0.04
−0.1
−0.08
−0.06
−0.04
−0.02
0
0.02
0.04
0.06

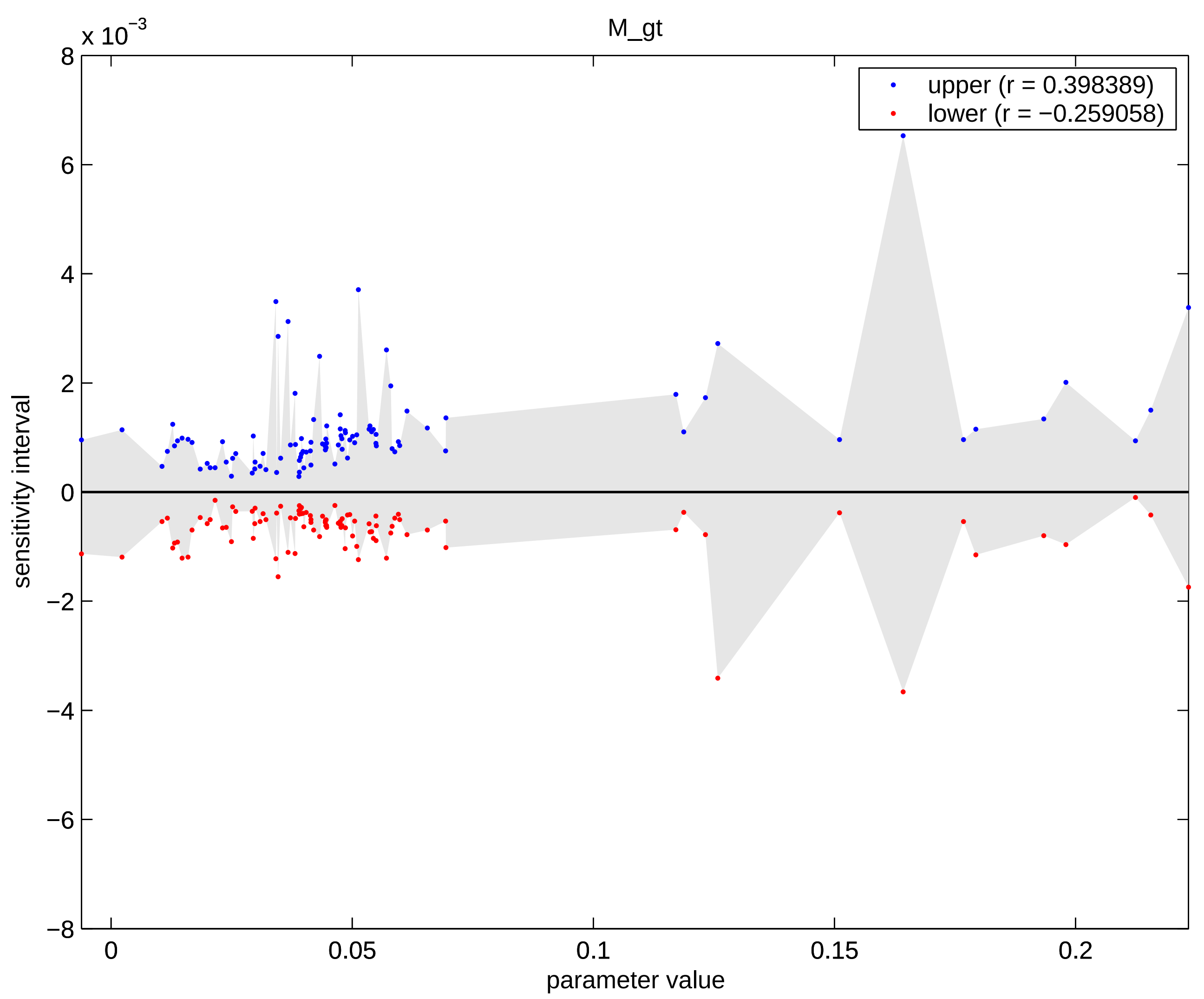

M_gt
x 10^−3
8
6
4
2
0
−2
−4
−6
−8
sensitivity interval
0
0.05
0.1
0.15
0.2
parameter value
upper (r = 0.398389)
lower (r = −0.259058)

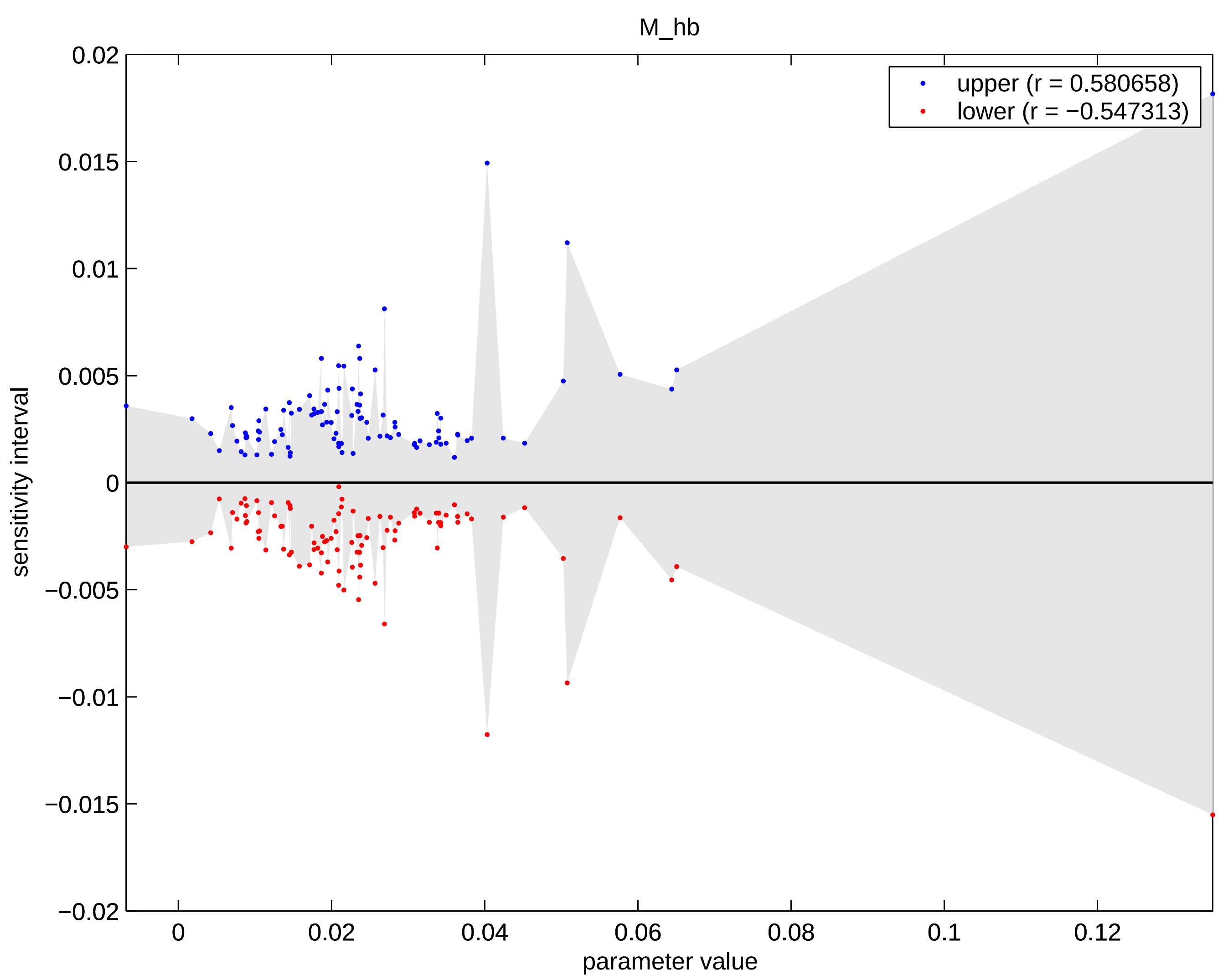
M_hb
upper (r = 0.580658)
lower (r = −0.547313)
sensitivity interval
parameter value
0.02
0.015
0.01
0.005
0
−0.005
−0.01
−0.015
−0.02
0
0.02
0.04
0.06
0.08
0.1
0.12

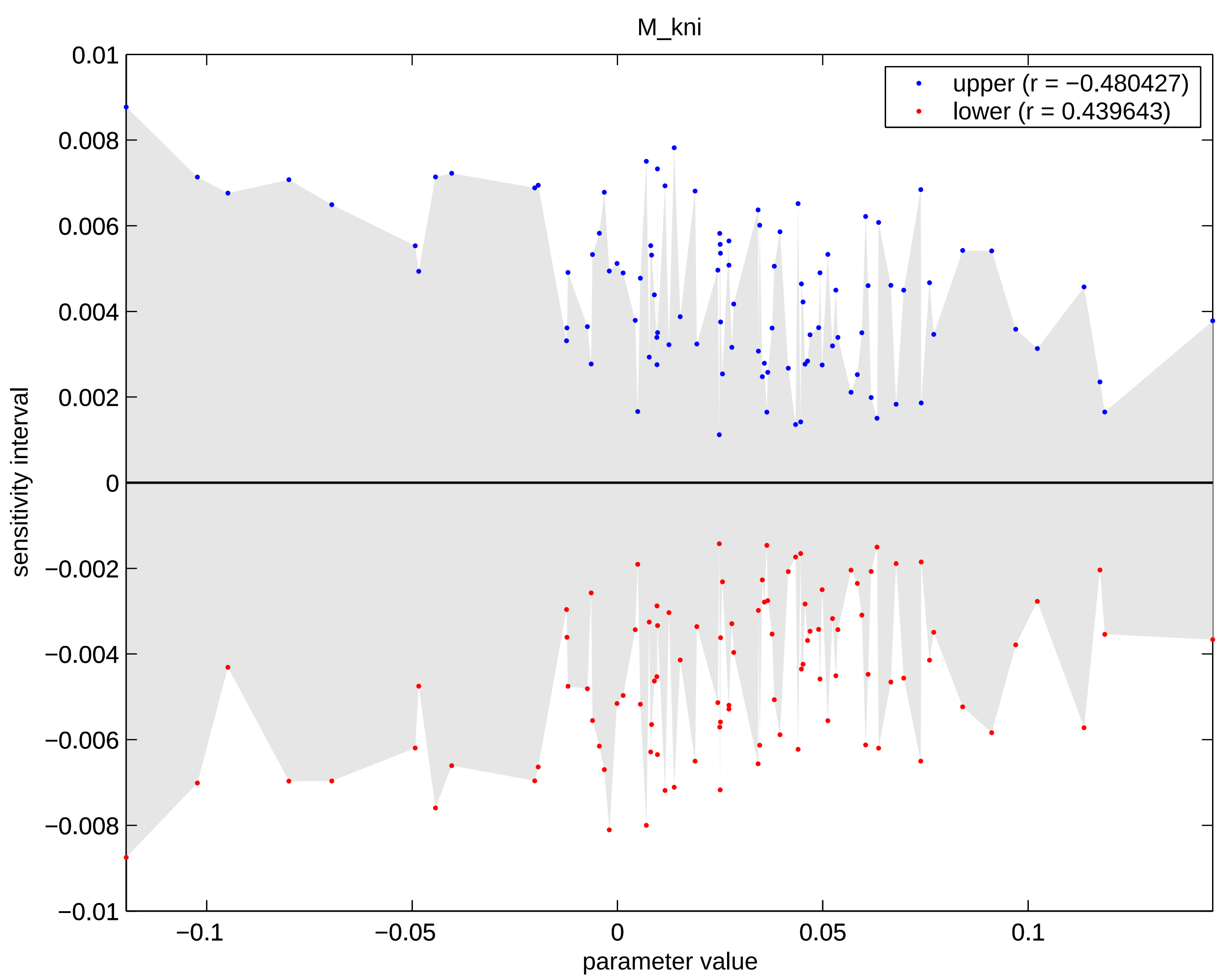
M_kni
upper (r = −0.480427)
lower (r = 0.439643)
sensitivity interval
parameter value
0.01
0.008
0.006
0.004
0.002
0
−0.002
−0.004
−0.006
−0.008
−0.01
−0.1
−0.05
0
0.05
0.1

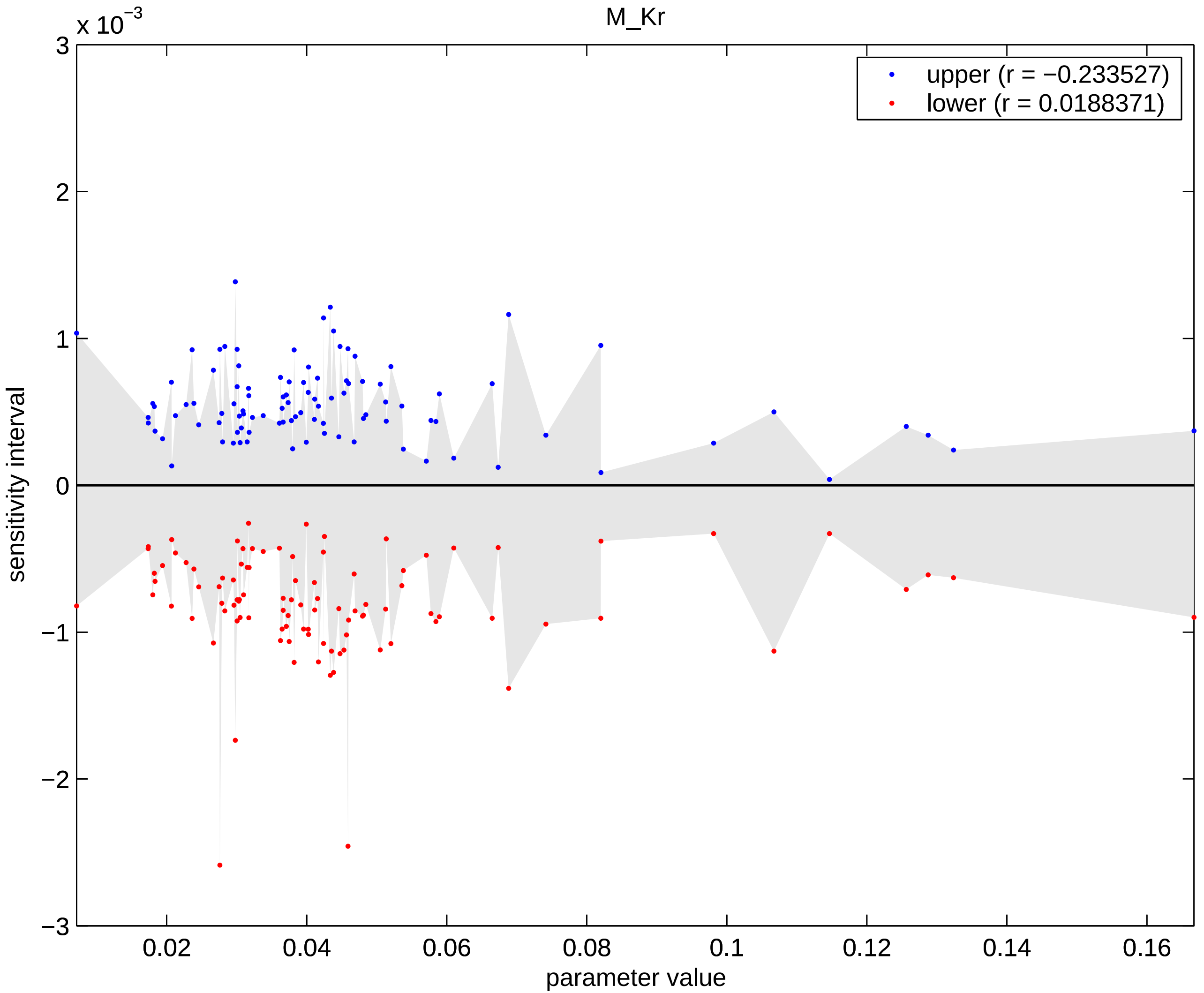

M_Kr
x 10^−3
upper (r = −0.233527)
lower (r = 0.0188371)
sensitivity interval
parameter value
3
2
1
0
−1
−2
−3
0.02
0.04
0.06
0.08
0.1
0.12
0.14
0.16

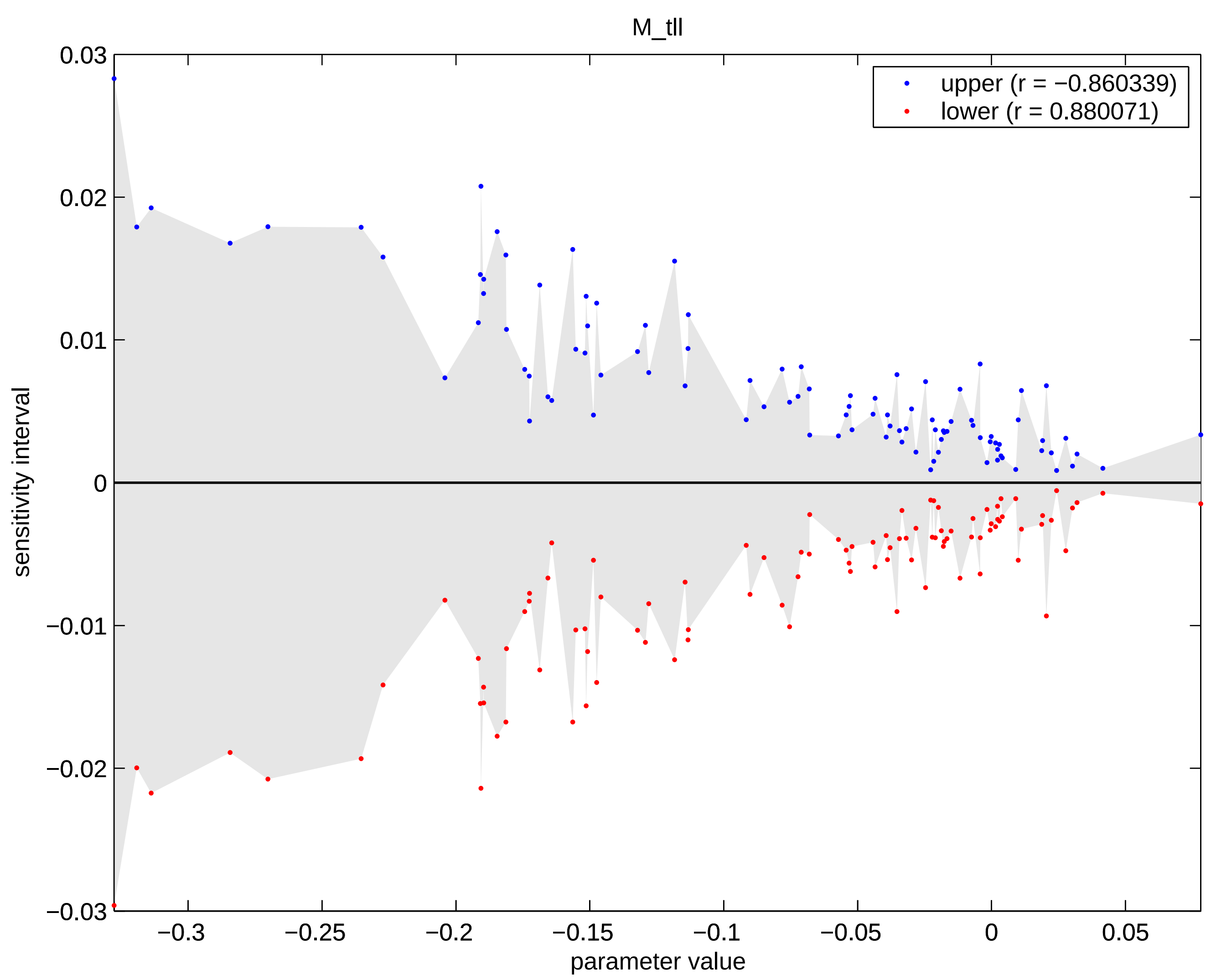
M_tll
upper (r = −0.860339)
lower (r = 0.880071)
sensitivity interval
parameter value
0.03
0.02
0.01
0
−0.01
−0.02
−0.03
−0.3
−0.25
−0.2
−0.15
−0.1
−0.05
0
0.05

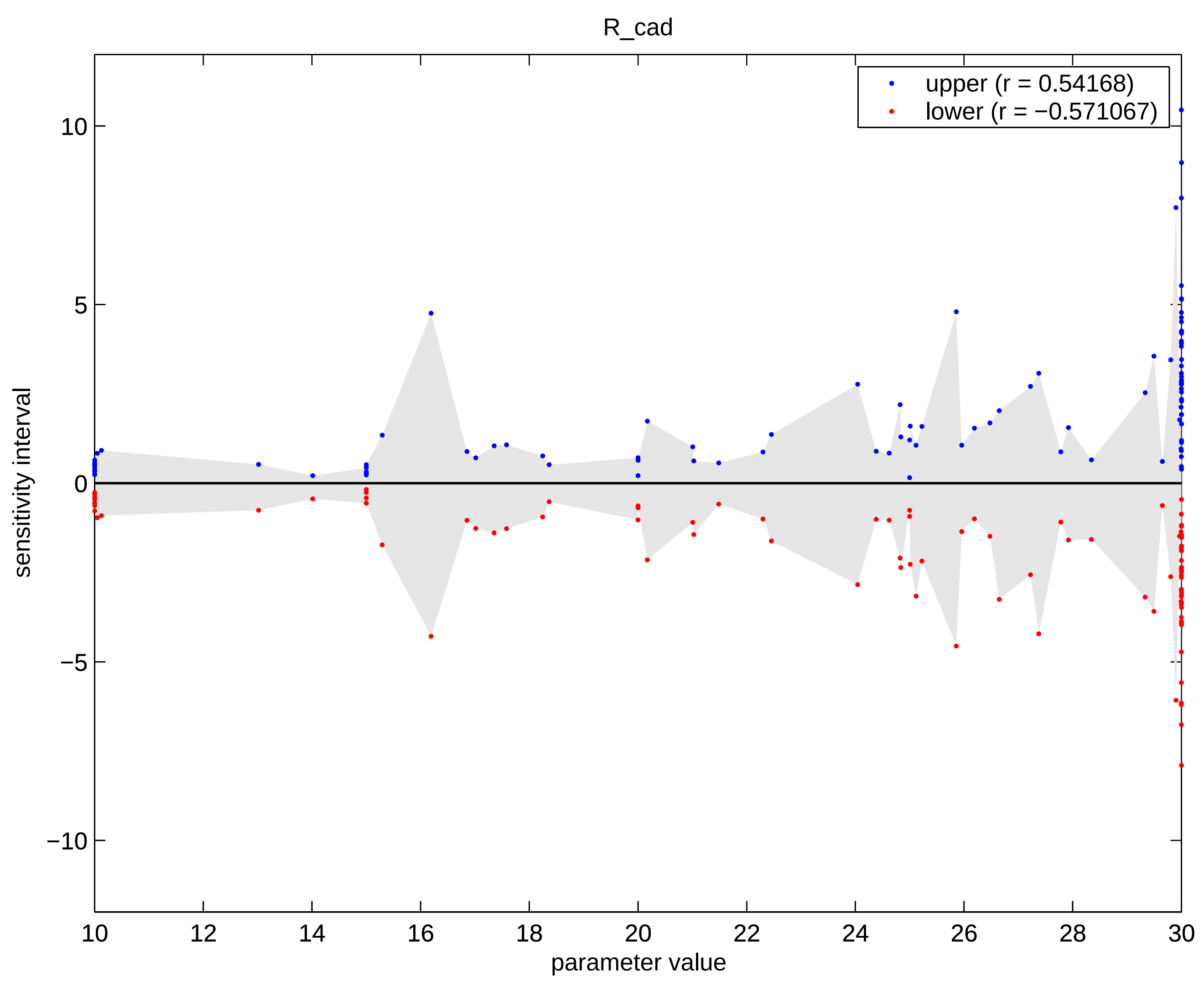
R_cad
upper (r = 0.54168)
lower (r = −0.571067)
sensitivity interval
parameter value
10
5
0
−5
−10
10
12
14
16
18
20
22
24
26
28
30

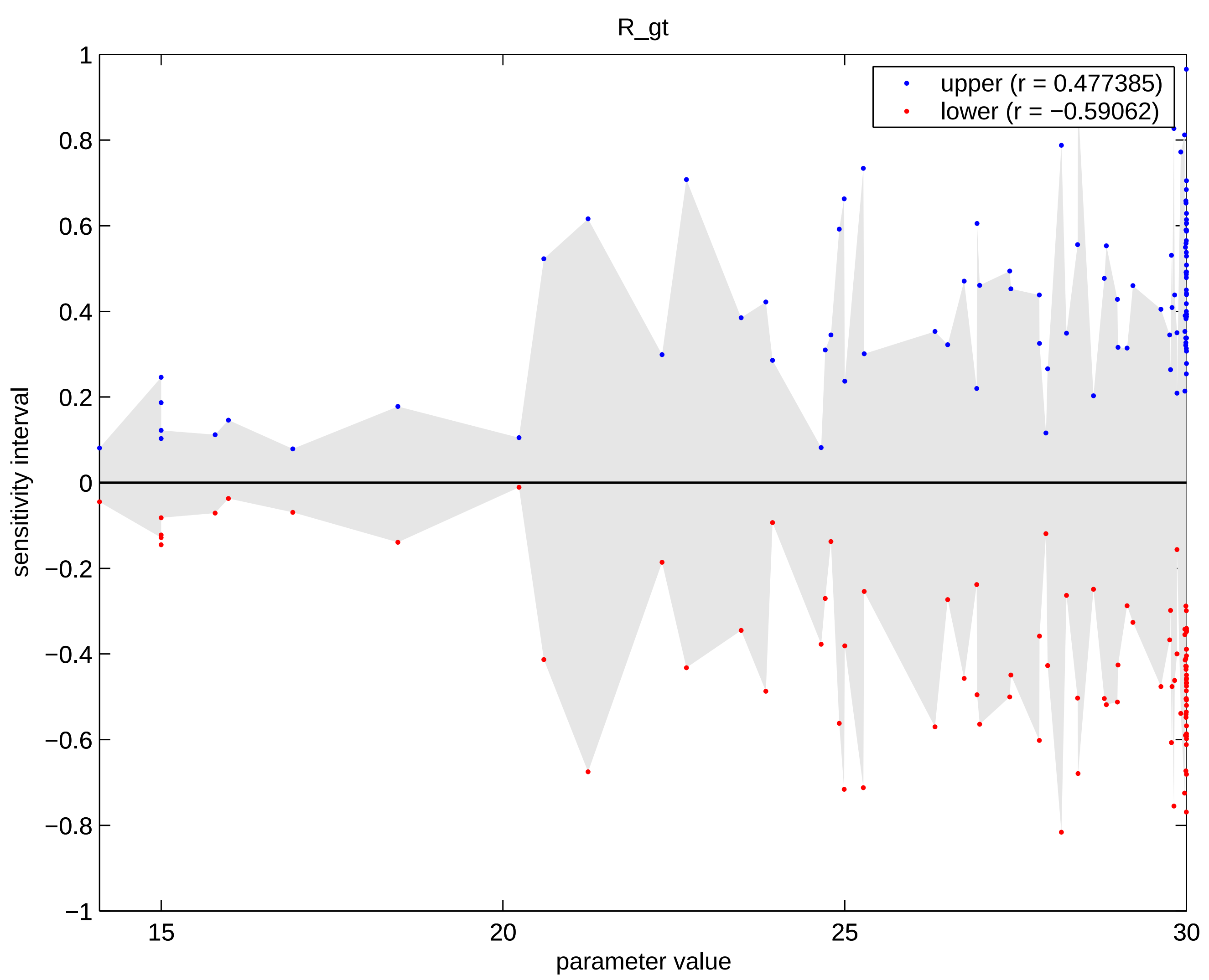

R_gt
upper (r = 0.477385)
lower (r = −0.59062)
sensitivity interval
parameter value
1
0.8
0.6
0.4
0.2
0
−0.2
−0.4
−0.6
−0.8
−1
15
20
25
30

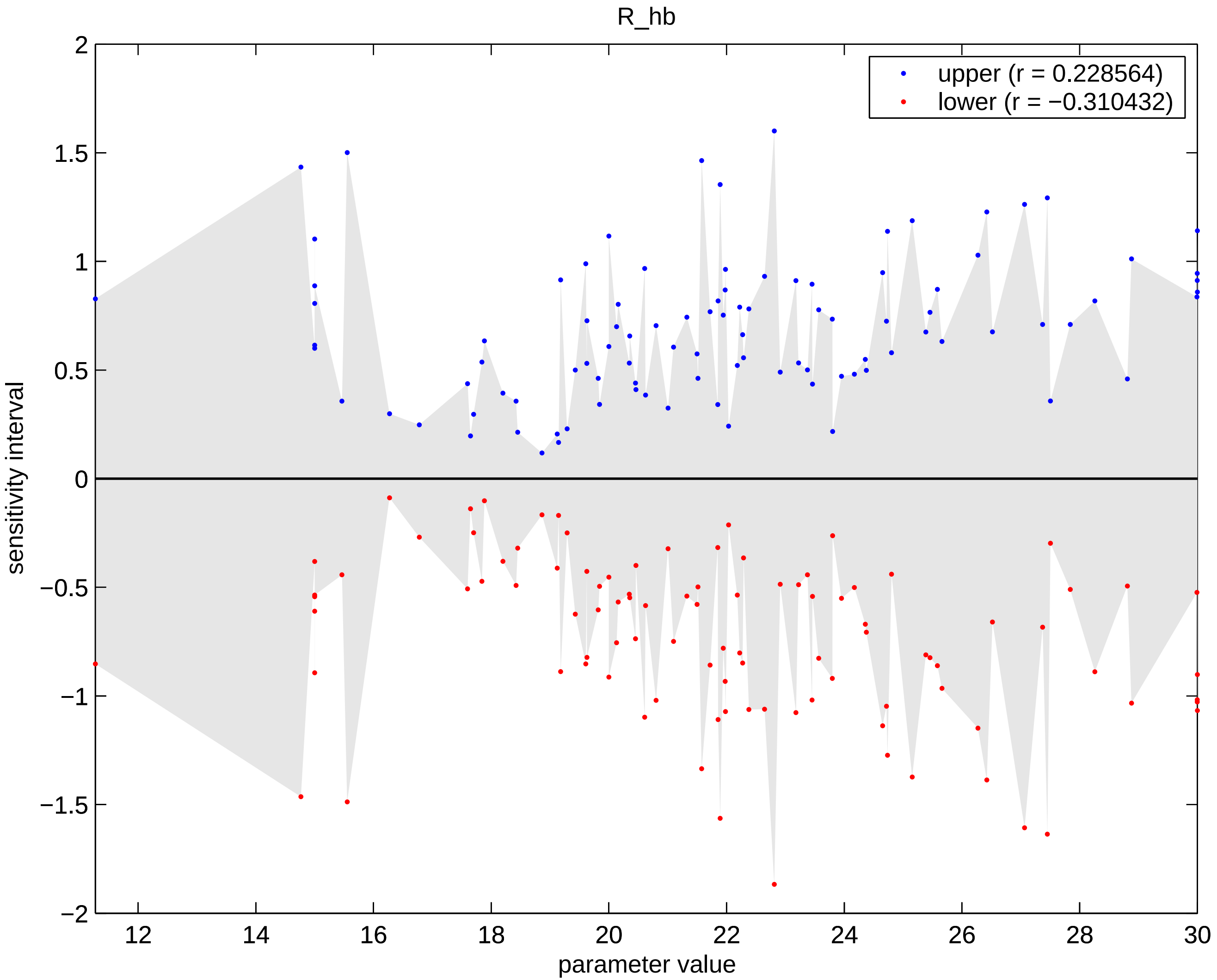
R_hb
upper (r = 0.228564)
lower (r = −0.310432)
sensitivity interval
parameter value
2
1.5
1
0.5
0
−0.5
−1
−1.5
−2
12
14
16
18
20
22
24
26
28
30

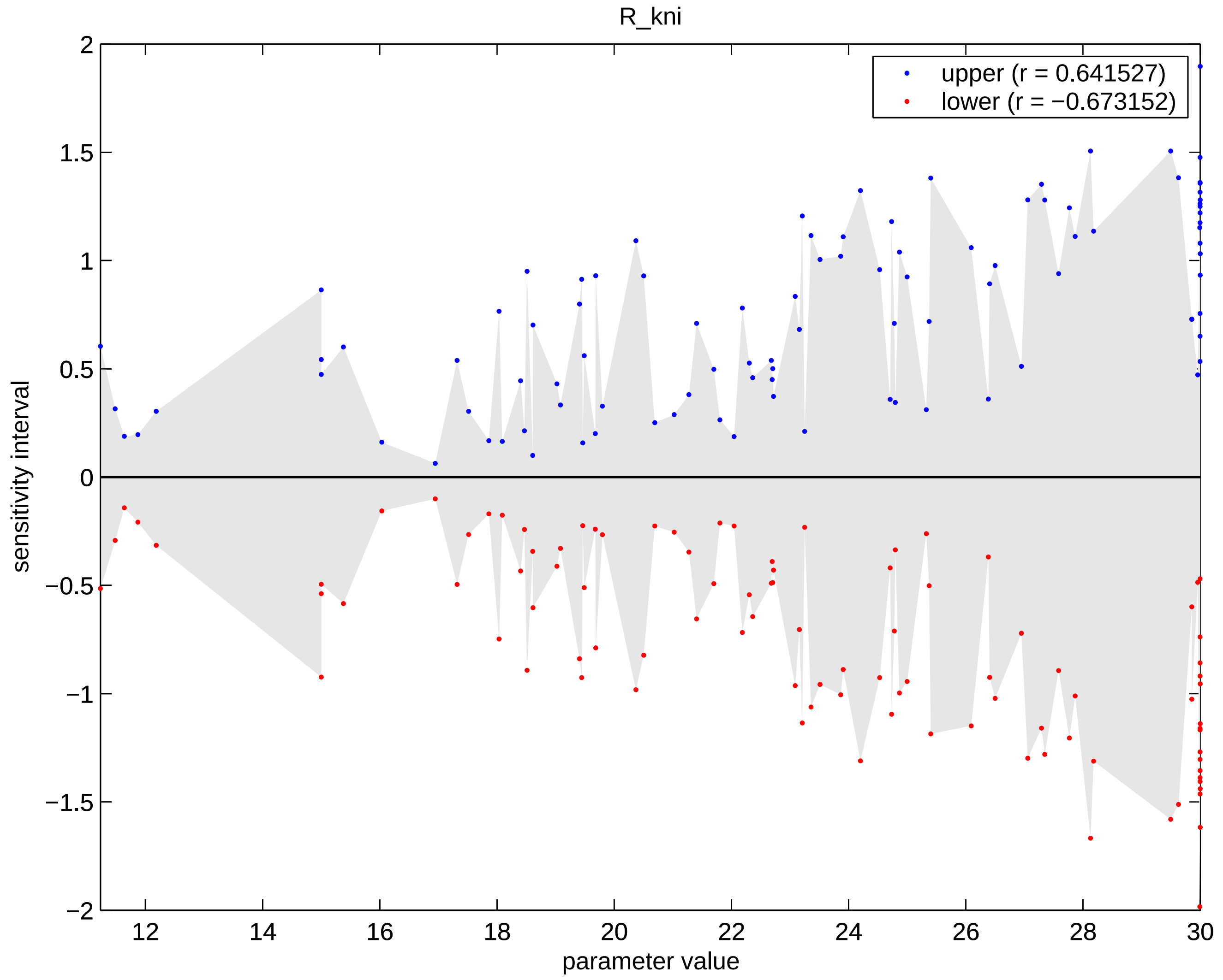
R_kni
upper (r = 0.641527)
lower (r = −0.673152)
sensitivity interval
parameter value
2
1.5
1
0.5
0
−0.5
−1
−1.5
−2
12
14
16
18
20
22
24
26
28
30

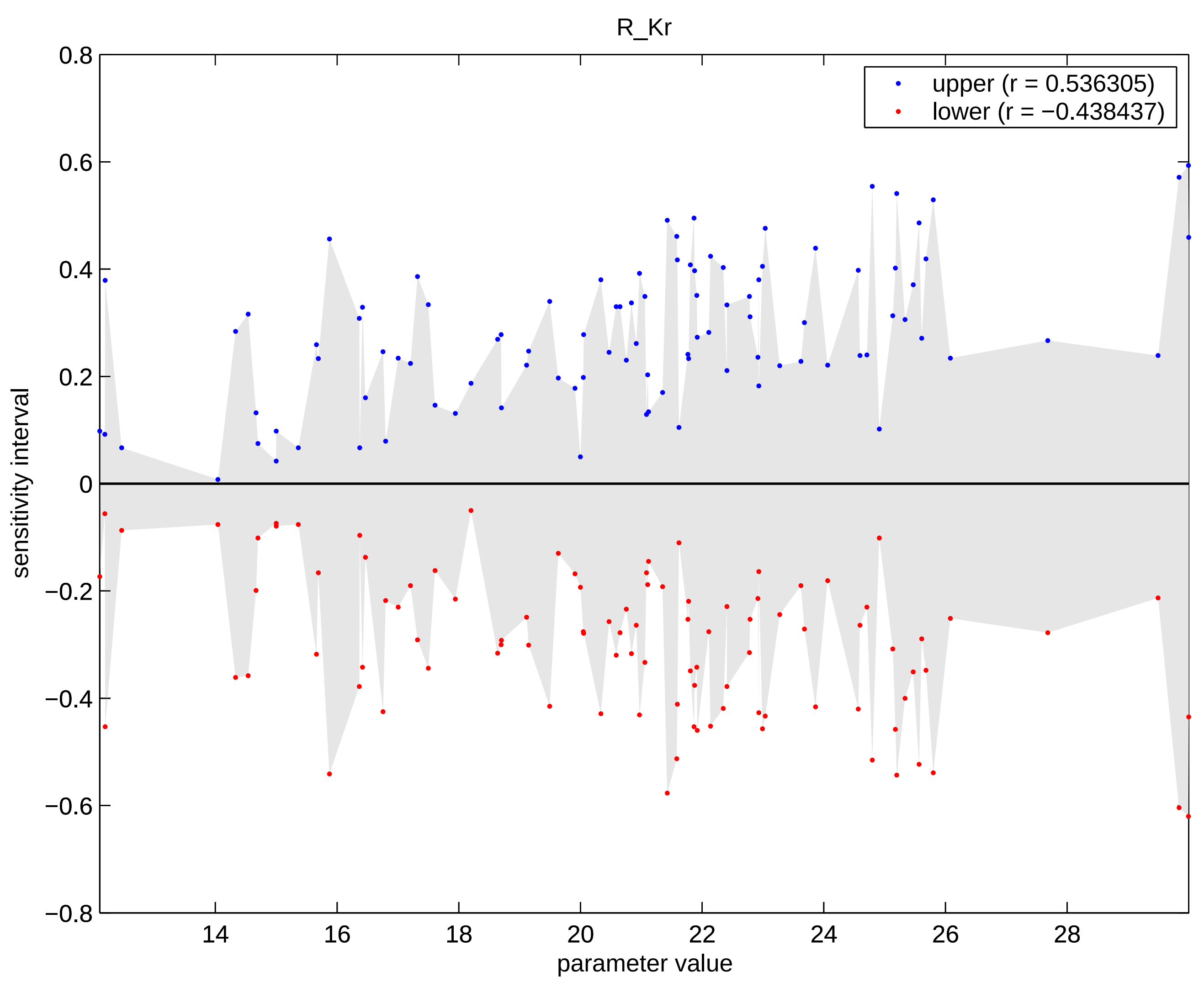
R_Kr
upper (r = 0.536305)
lower (r = −0.438437)
sensitivity interval
parameter value
0.8
0.6
0.4
0.2
0
−0.2
−0.4
−0.6
−0.8
14
16
18
20
22
24
26
28

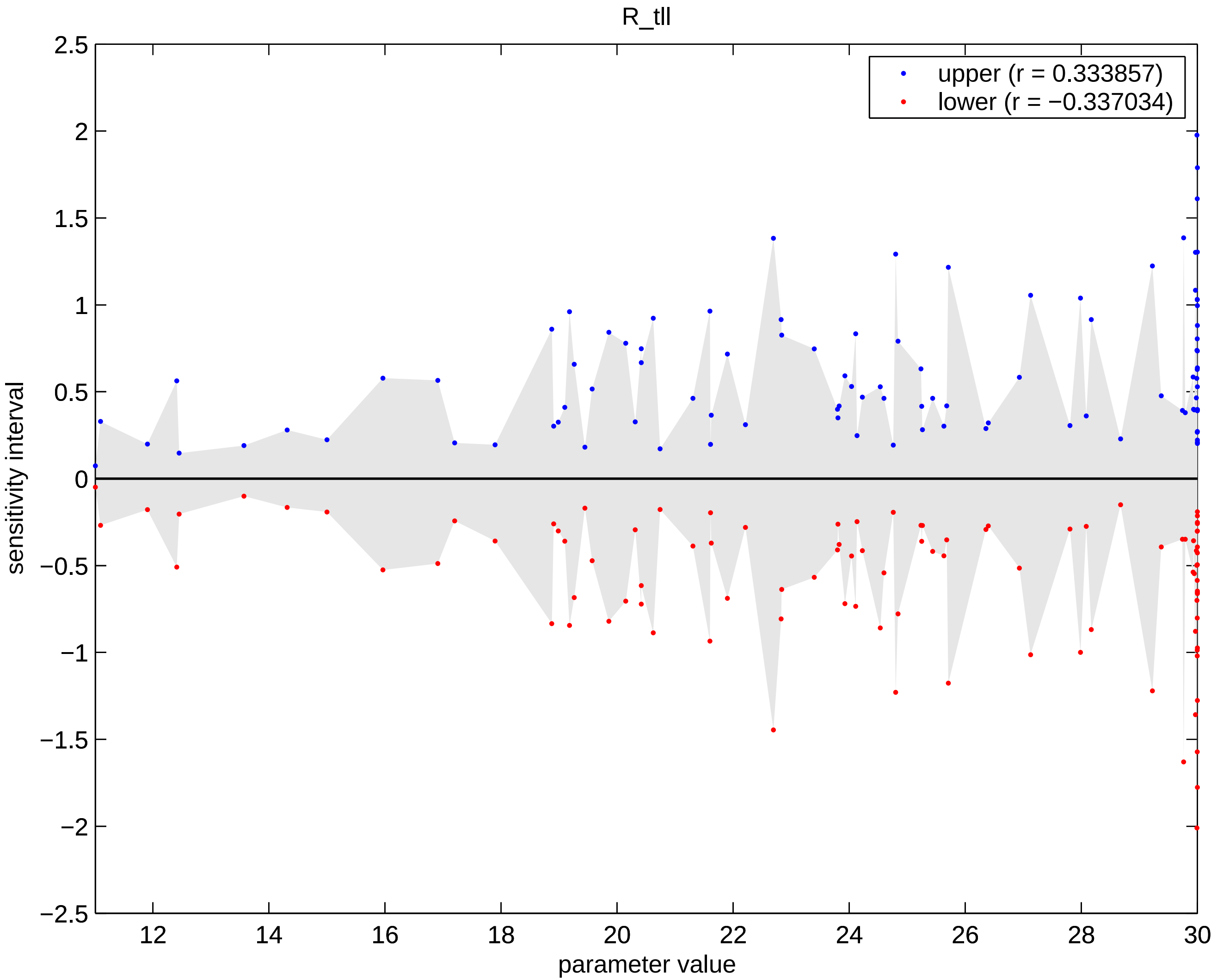

R_tll
upper (r = 0.333857)
lower (r = −0.337034)
sensitivity interval
parameter value
2.5
2
1.5
1
0.5
0
−0.5
−1
−1.5
−2
−2.5
12
14
16
18
20
22
24
26
28
30

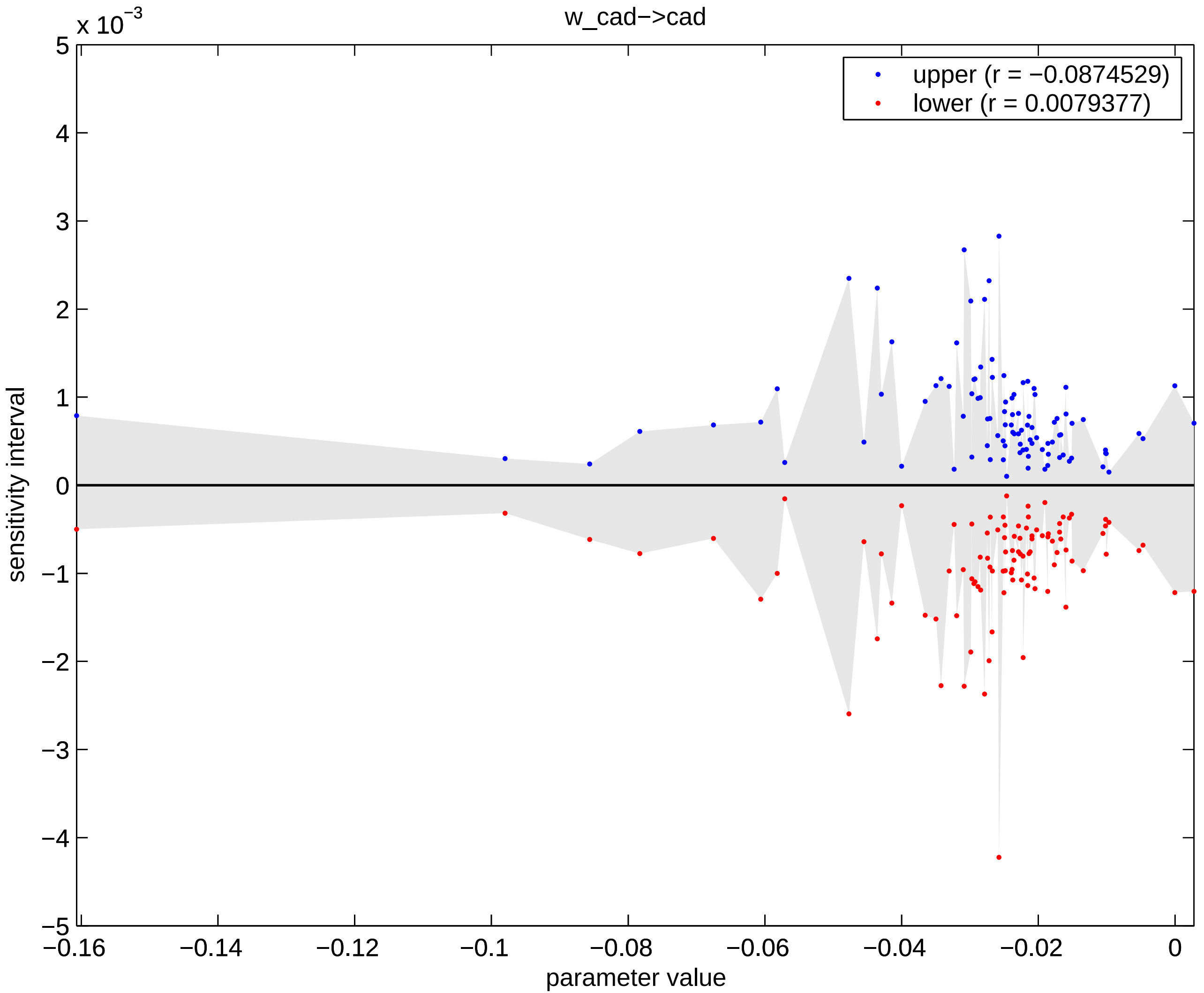

w_cad−>cad
x 10^−3
upper (r = −0.0874529)
lower (r = 0.0079377)
sensitivity interval
parameter value
5
4
3
2
1
0
−1
−2
−3
−4
−5
−0.16
−0.14
−0.12
−0.1
−0.08
−0.06
−0.04
−0.02
0

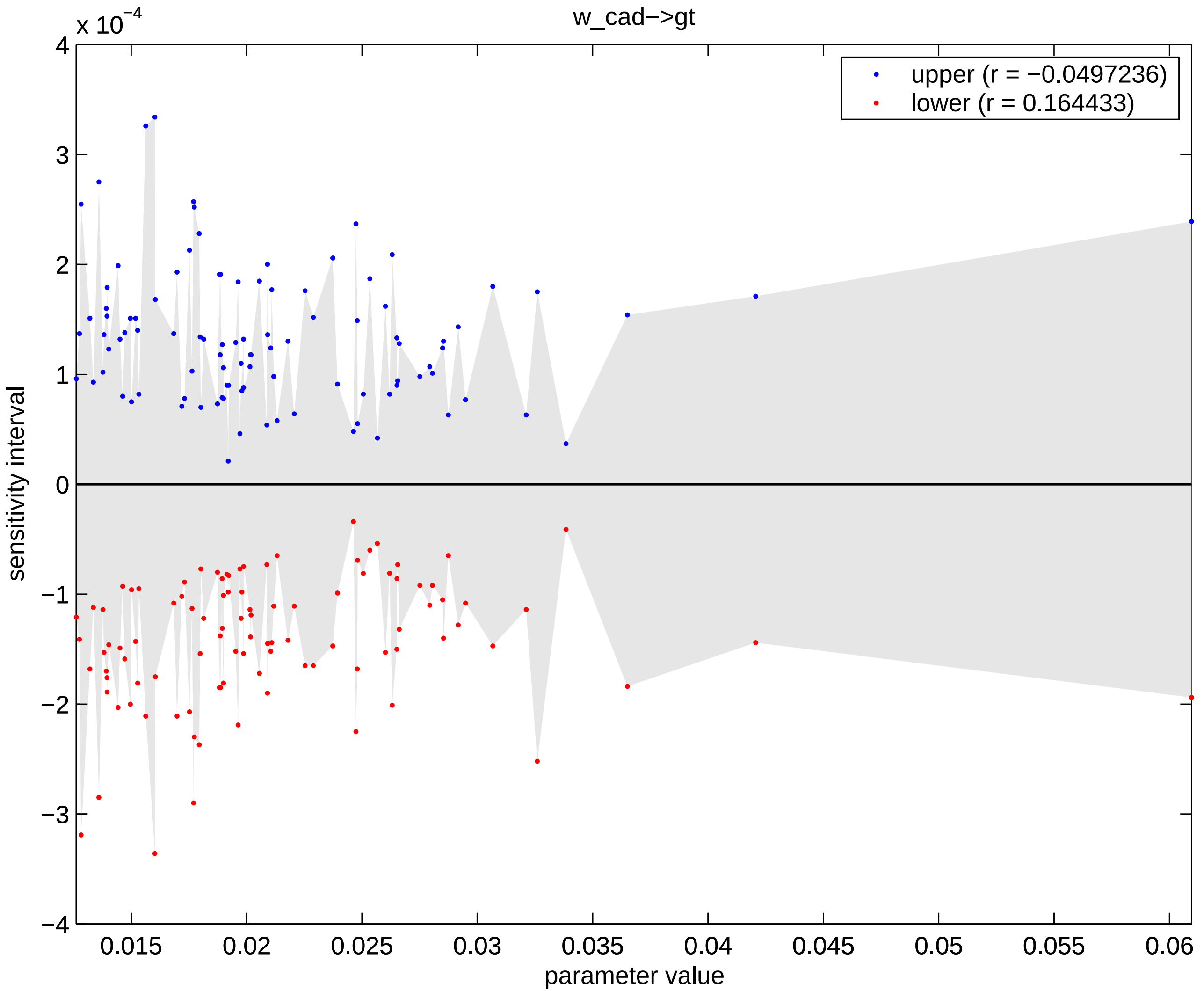

w_cad−>gt
x 10^−4
upper (r = −0.0497236)
lower (r = 0.164433)
sensitivity interval
parameter value
4
3
2
1
0
−1
−2
−3
−4
0.015
0.02
0.025
0.03
0.035
0.04
0.045
0.05
0.055
0.06

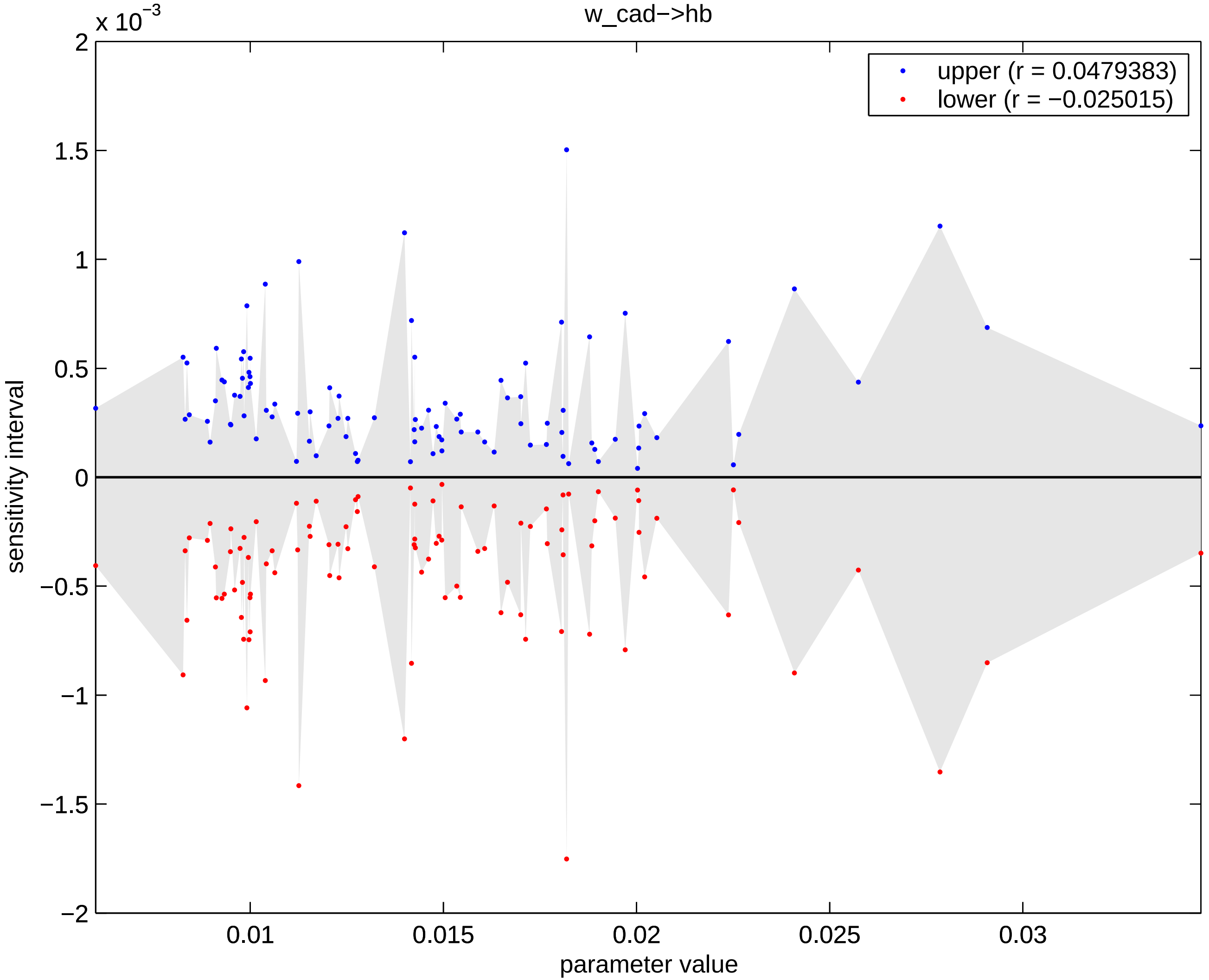
w_cad−>hb
x 10^−3
upper (r = 0.0479383)
lower (r = −0.025015)
sensitivity interval
parameter value
2
1.5
1
0.5
0
−0.5
−1
−1.5
−2
0.01
0.015
0.02
0.025
0.03

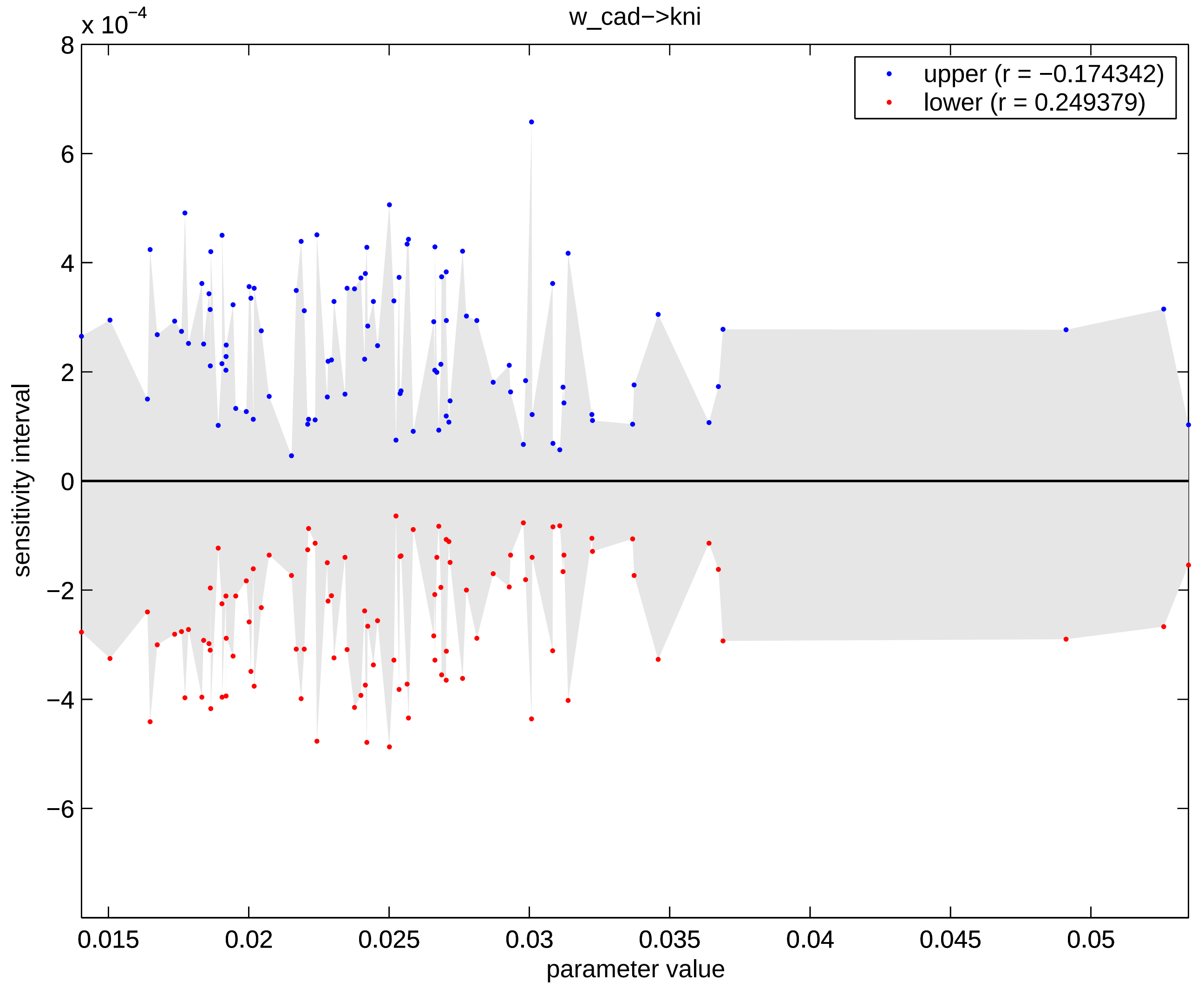

w_cad−>kni
x 10^−4
8
6
4
2
0
−2
−4
−6
sensitivity interval
upper (r = −0.174342)
lower (r = 0.249379)
0.015
0.02
0.025
0.03
0.035
0.04
0.045
0.05
parameter value

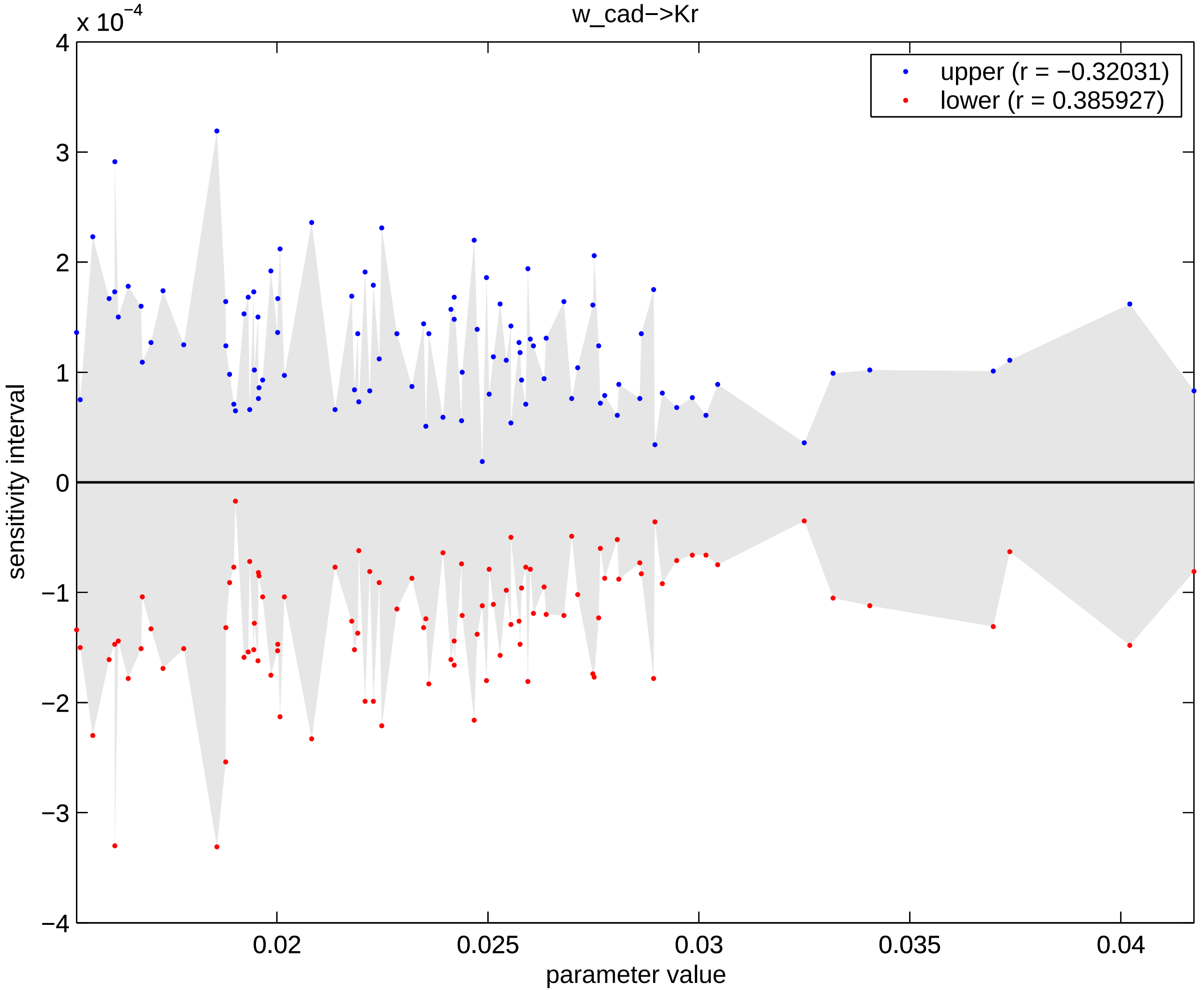
w_cad−>Kr
x 10^−4
upper (r = −0.32031)
lower (r = 0.385927)
sensitivity interval
parameter value
4
3
2
1
0
−1
−2
−3
−4
0.02
0.025
0.03
0.035
0.04

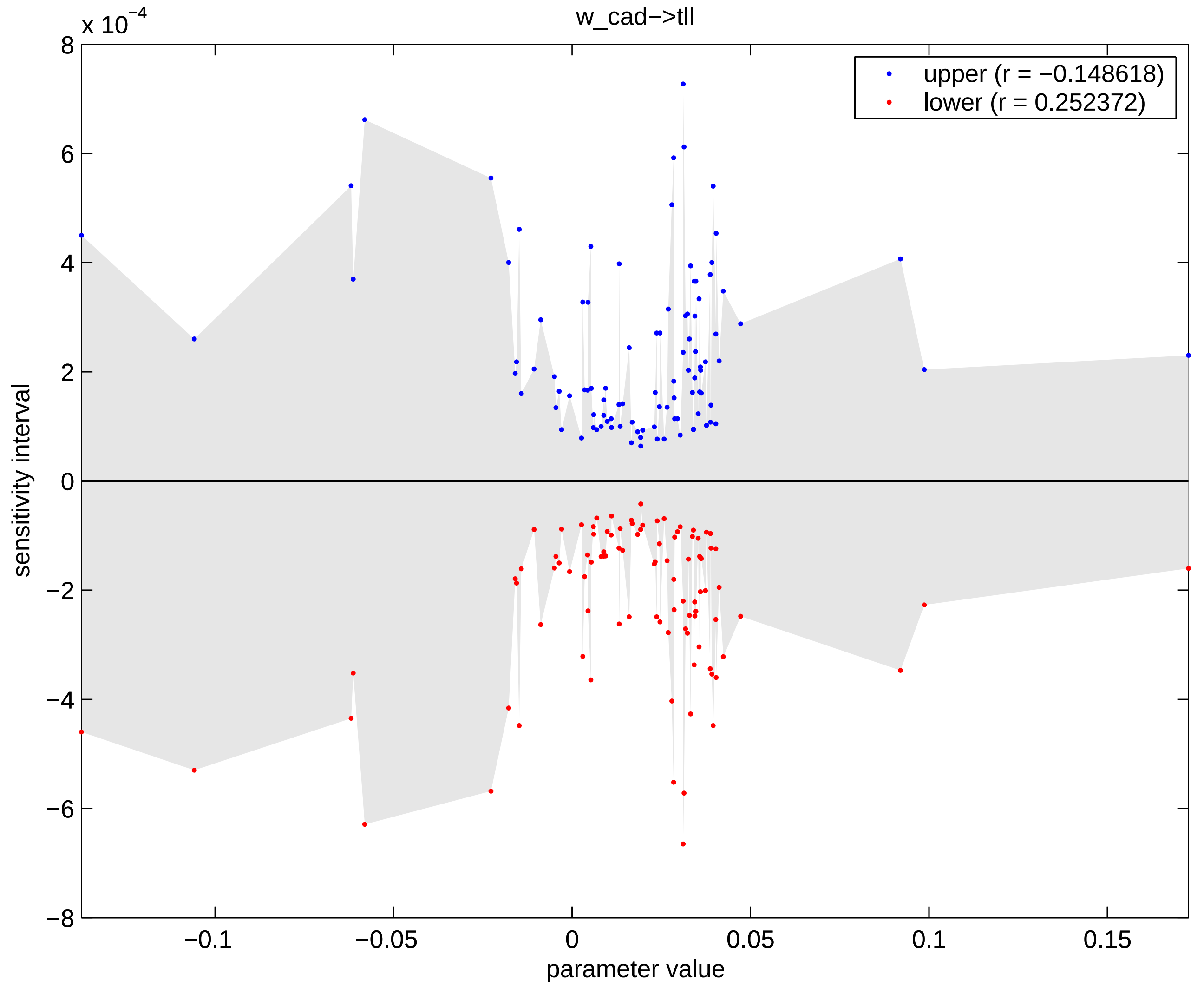
w_cad−>tll
x 10^−4
8
6
4
2
0
−2
−4
−6
−8
sensitivity interval
upper (r = −0.148618)
lower (r = 0.252372)
−0.1
−0.05
0
0.05
0.1
0.15
parameter value

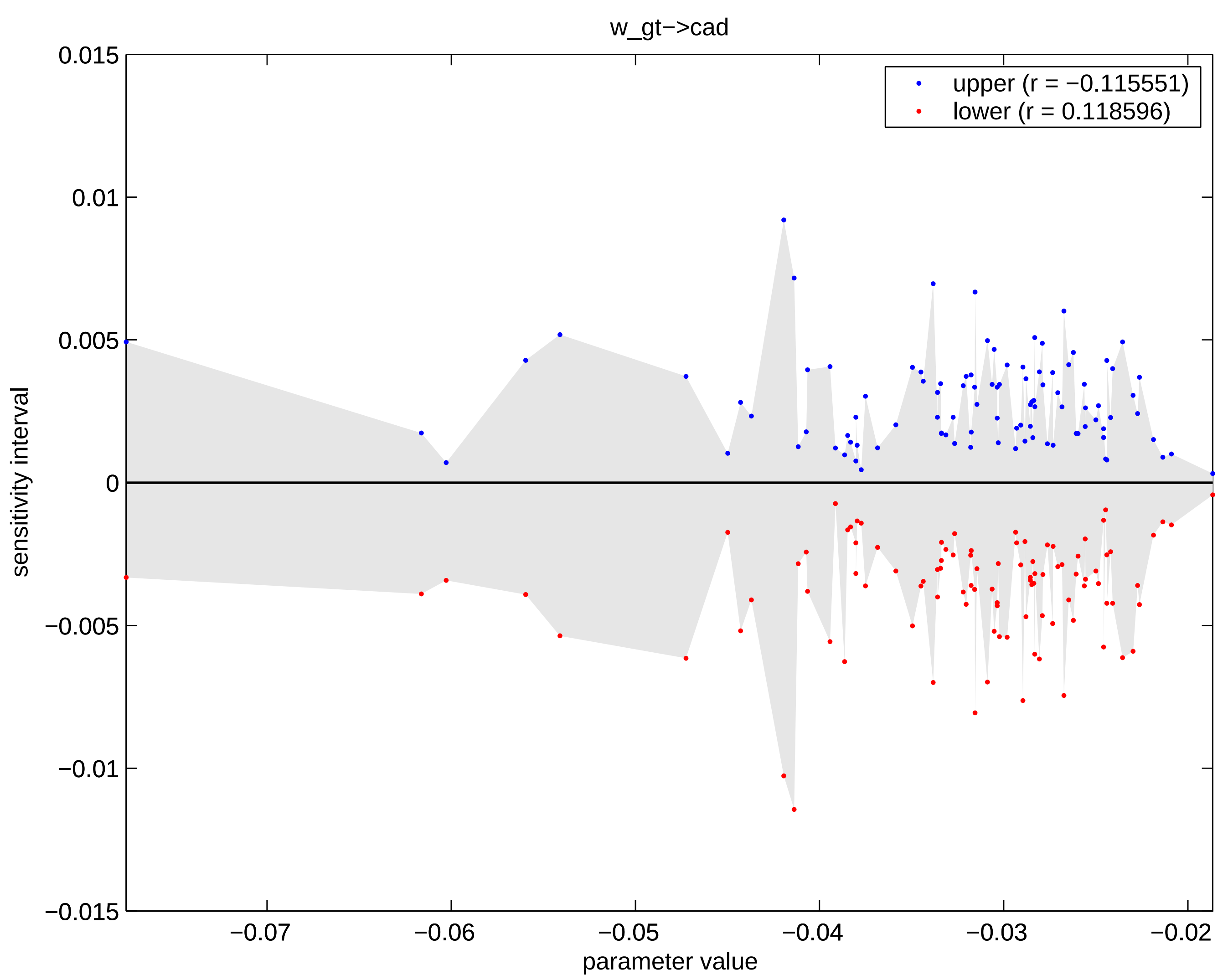

w_gt−>cad
upper (r = −0.115551)
lower (r = 0.118596)
0.015
0.01
0.005
0
−0.005
−0.01
−0.015
sensitivity interval
−0.07
−0.06
−0.05
−0.04
−0.03
−0.02
parameter value

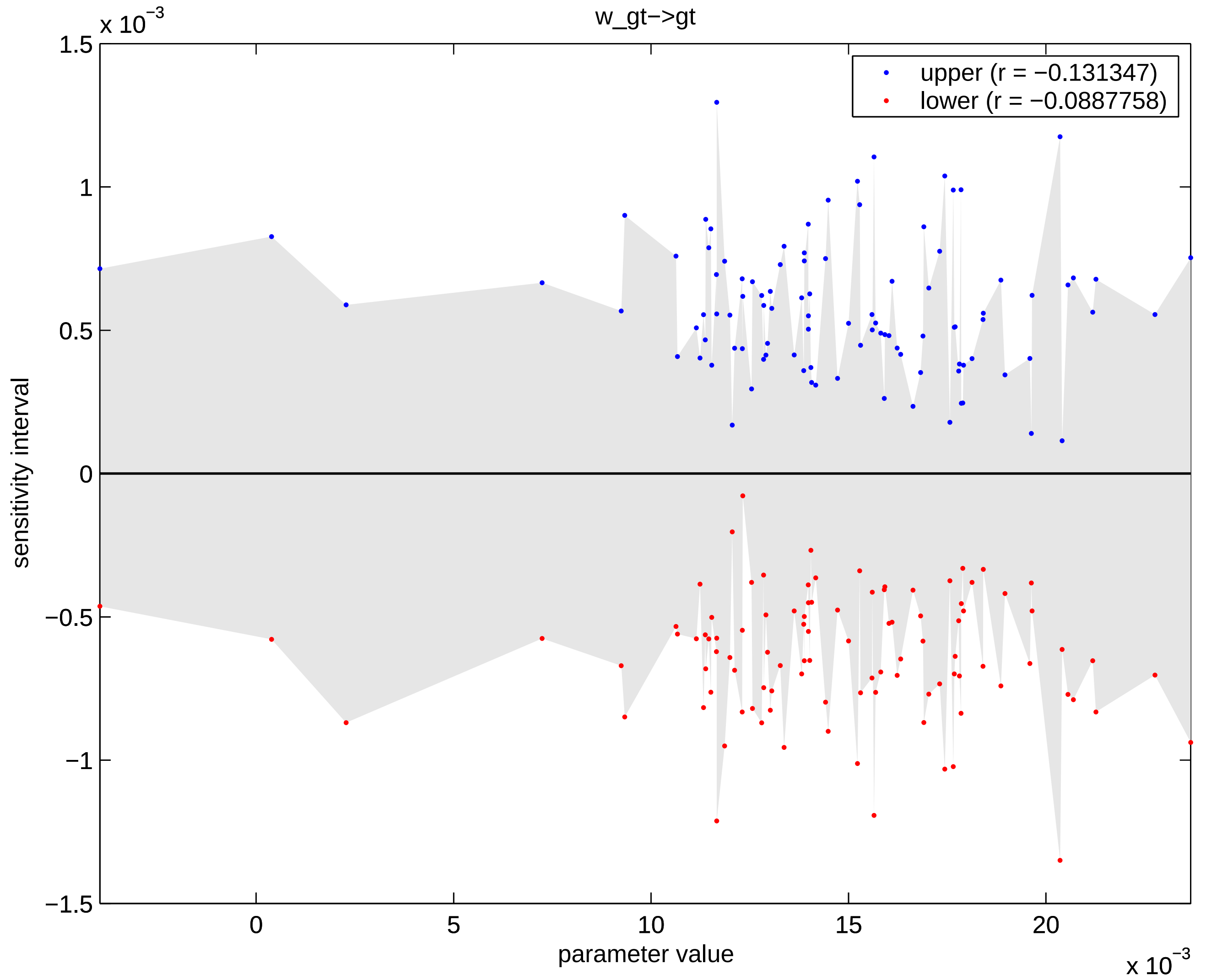

w_gt−>gt
x 10^−3
1.5
1
0.5
0
−0.5
−1
−1.5
upper (r = −0.131347)
lower (r = −0.0887758)
sensitivity interval
0
5
10
15
20
parameter value
x 10^−3

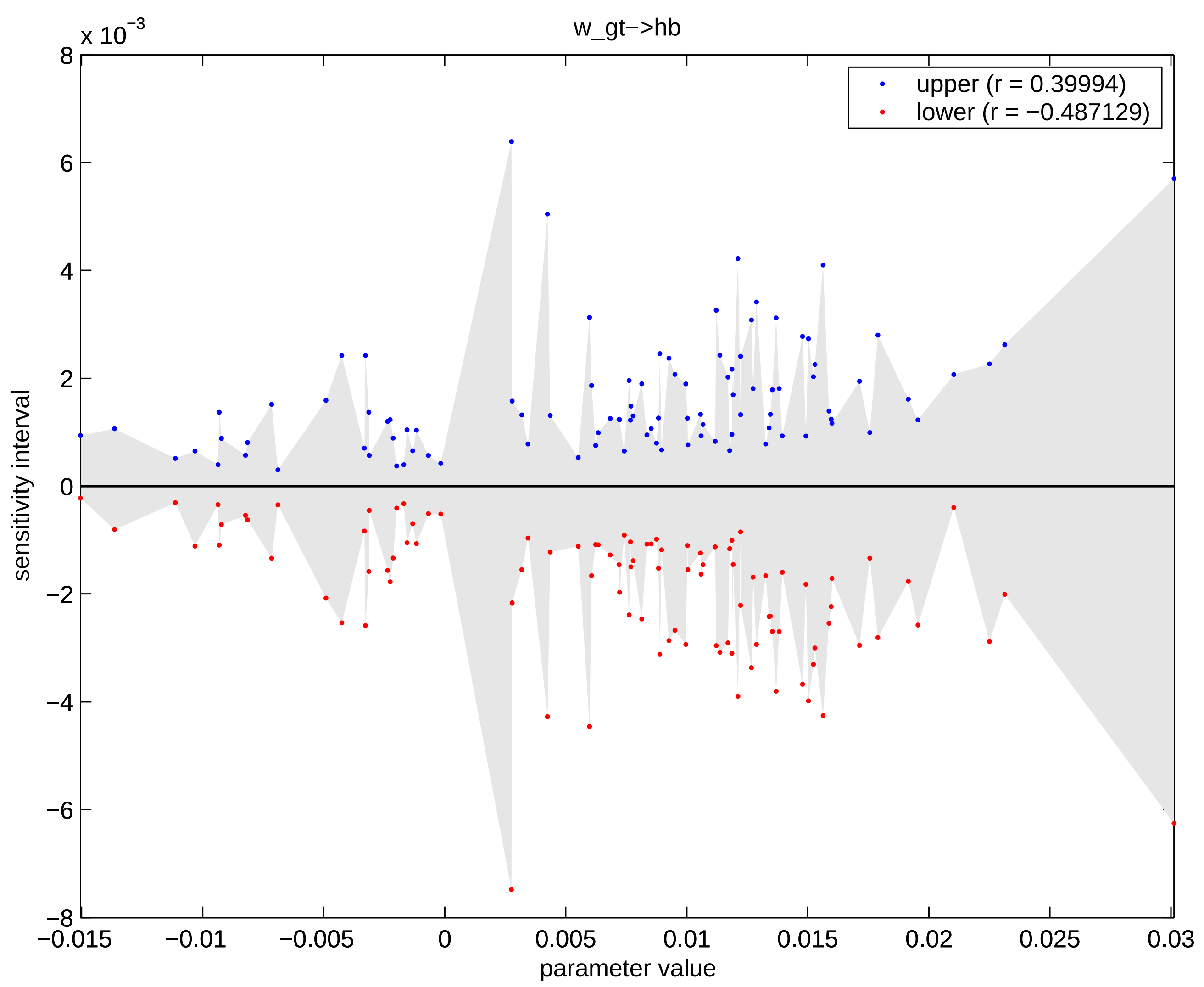

w_gt->hb
x 10^-3
upper (r = 0.39994)
lower (r = −0.487129)
sensitivity interval
parameter value
8
6
4
2
0
−2
−4
−6
−8
−0.015
−0.01
−0.005
0
0.005
0.01
0.015
0.02
0.025
0.03

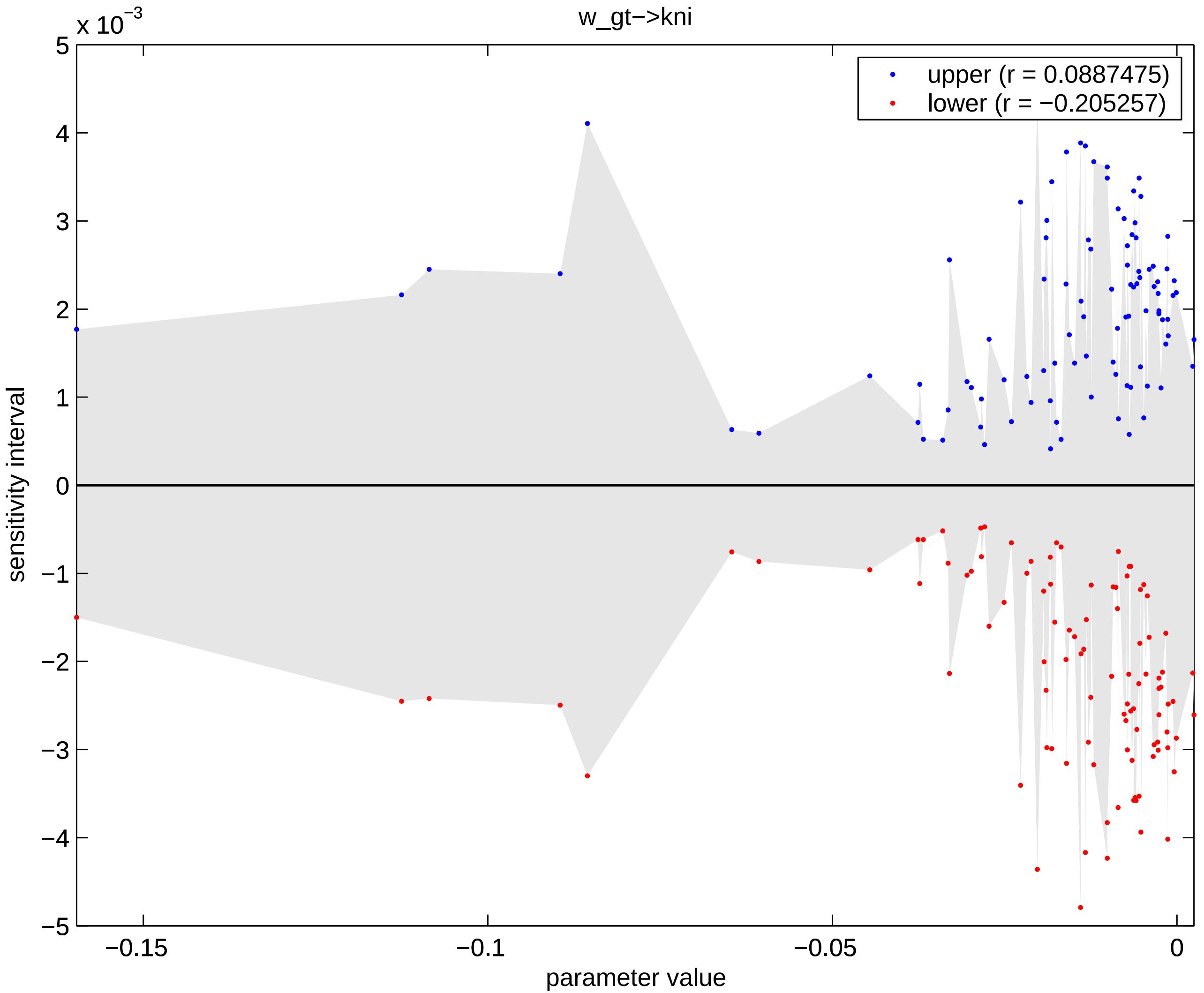
w_gt−>kni
x 10^−3
upper (r = 0.0887475)
lower (r = −0.205257)
sensitivity interval
parameter value
5
4
3
2
1
0
−1
−2
−3
−4
−5
−0.15
−0.1
−0.05
0

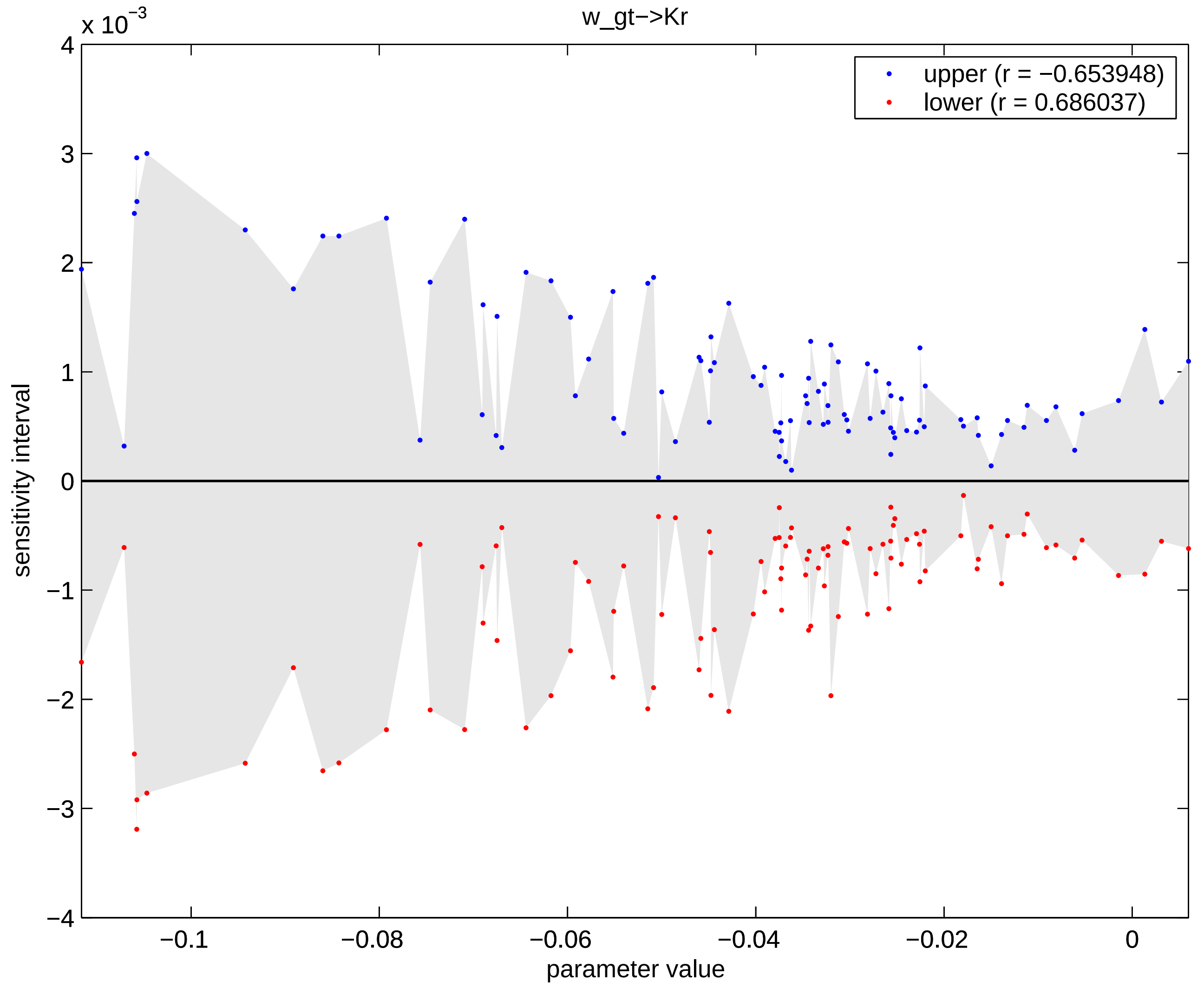
w_gt−>Kr
x 10^−3
upper (r = −0.653948)
lower (r = 0.686037)
sensitivity interval
parameter value

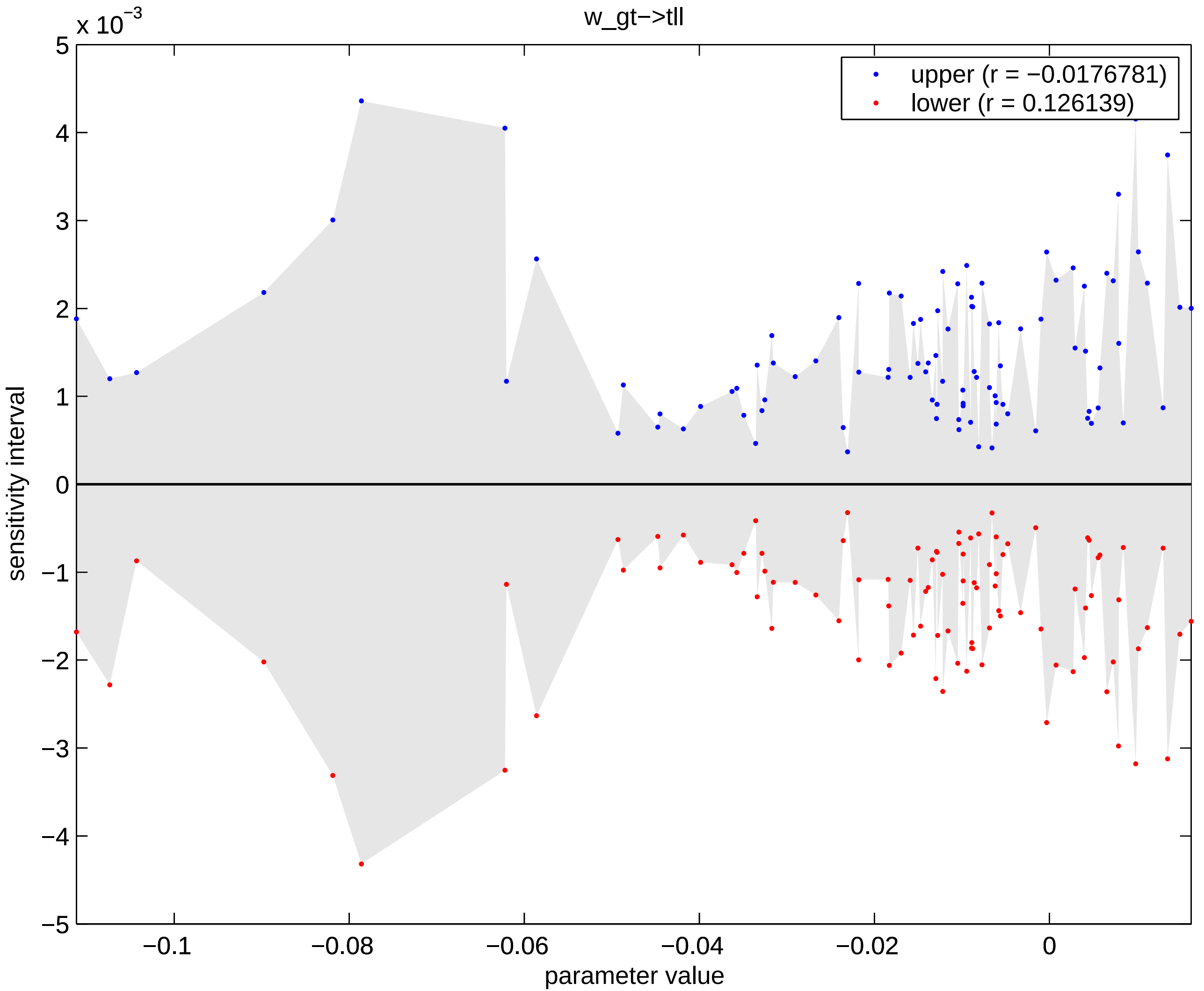

w_gt−>tll
x 10^−3
5
4
3
2
1
0
−1
−2
−3
−4
−5
sensitivity interval
upper (r = −0.0176781)
lower (r = 0.126139)
−0.1
−0.08
−0.06
−0.04
−0.02
0
parameter value

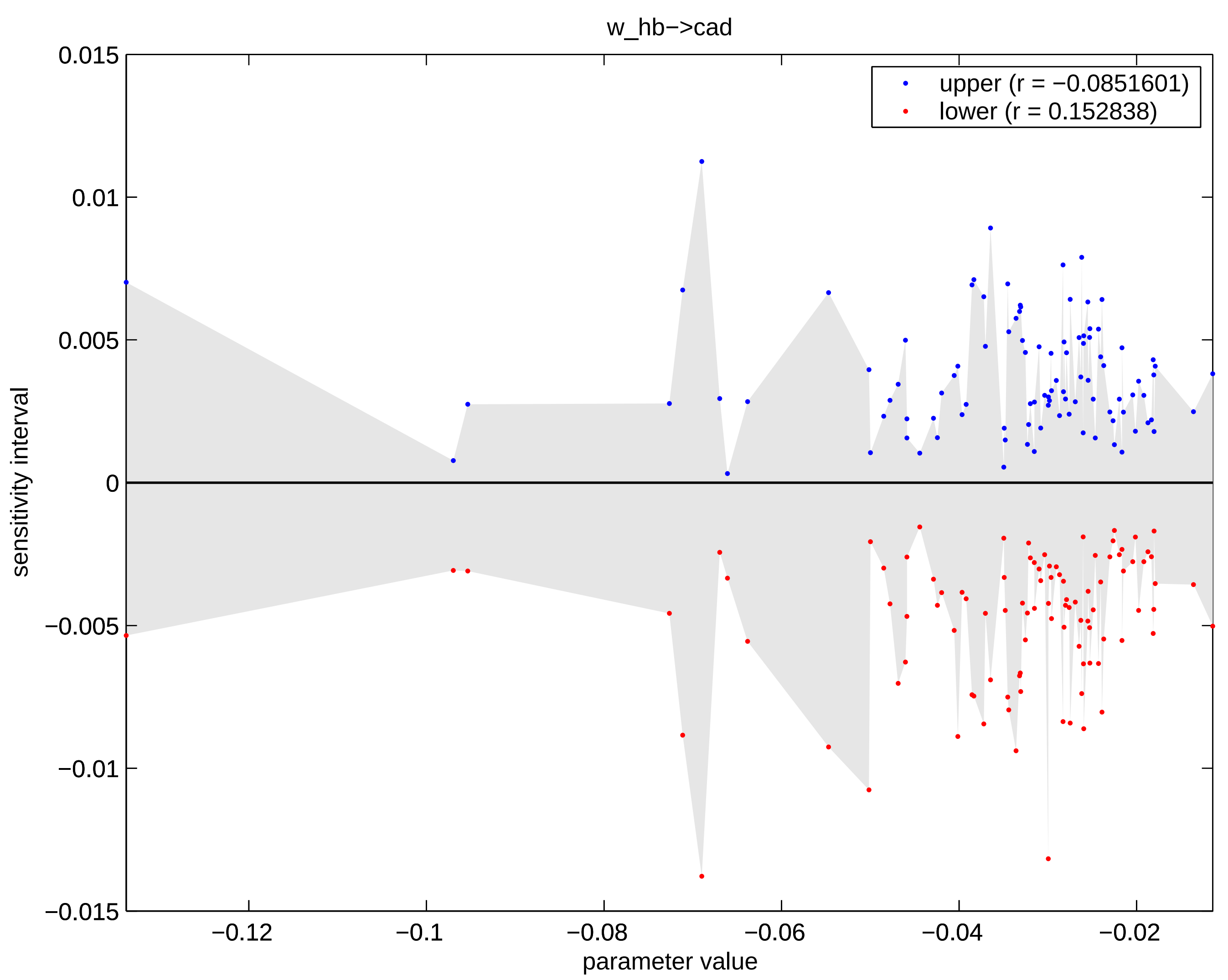

w_hb−>cad
upper (r = −0.0851601)
lower (r = 0.152838)
0.015
0.01
0.005
0
−0.005
−0.01
−0.015
sensitivity interval
−0.12
−0.1
−0.08
−0.06
−0.04
−0.02
parameter value

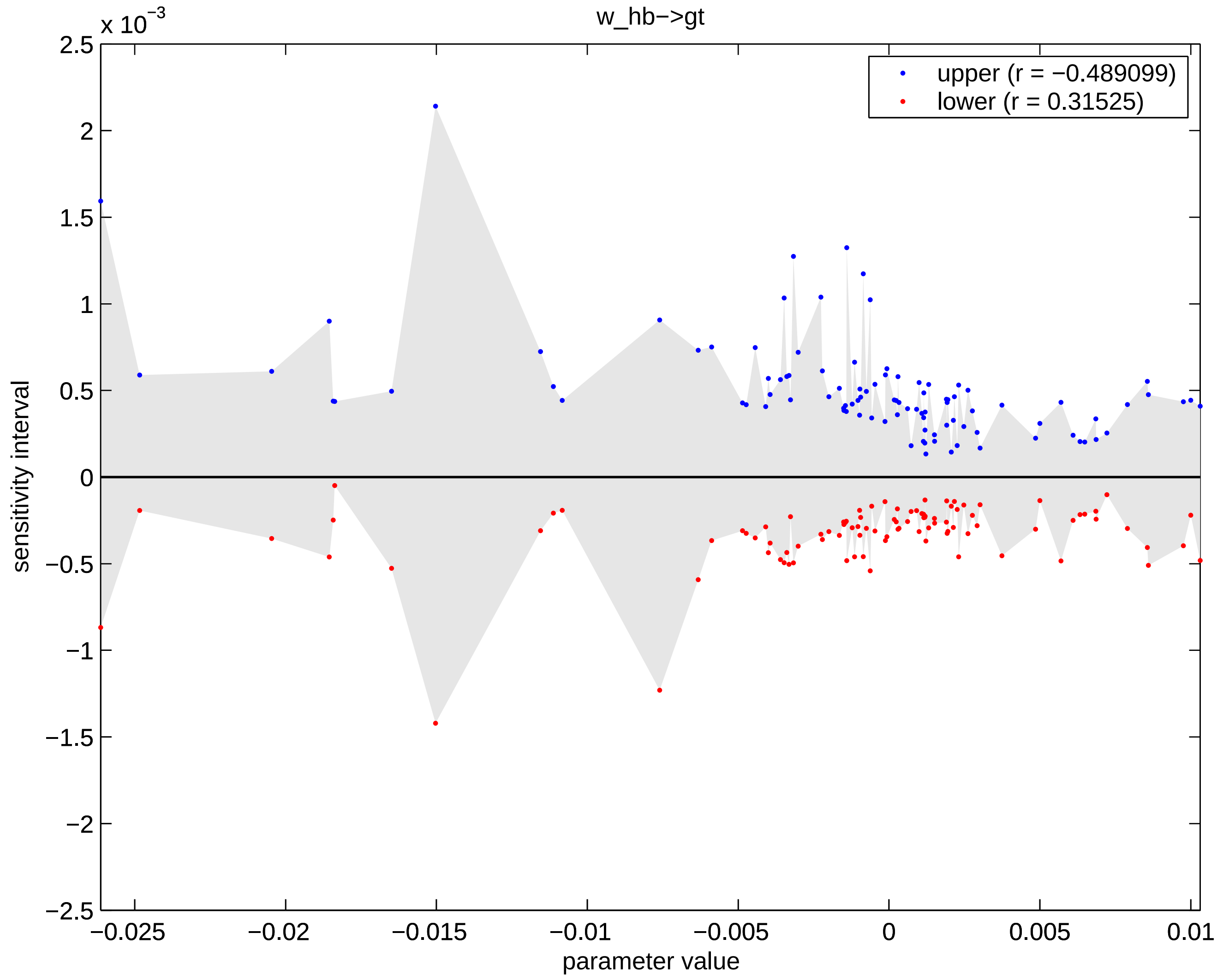

w_hb−>gt
x 10^−3
2.5
2
1.5
1
0.5
0
−0.5
−1
−1.5
−2
−2.5
sensitivity interval
−0.025
−0.02
−0.015
−0.01
−0.005
0
0.005
0.01
parameter value
upper (r = −0.489099)
lower (r = 0.31525)

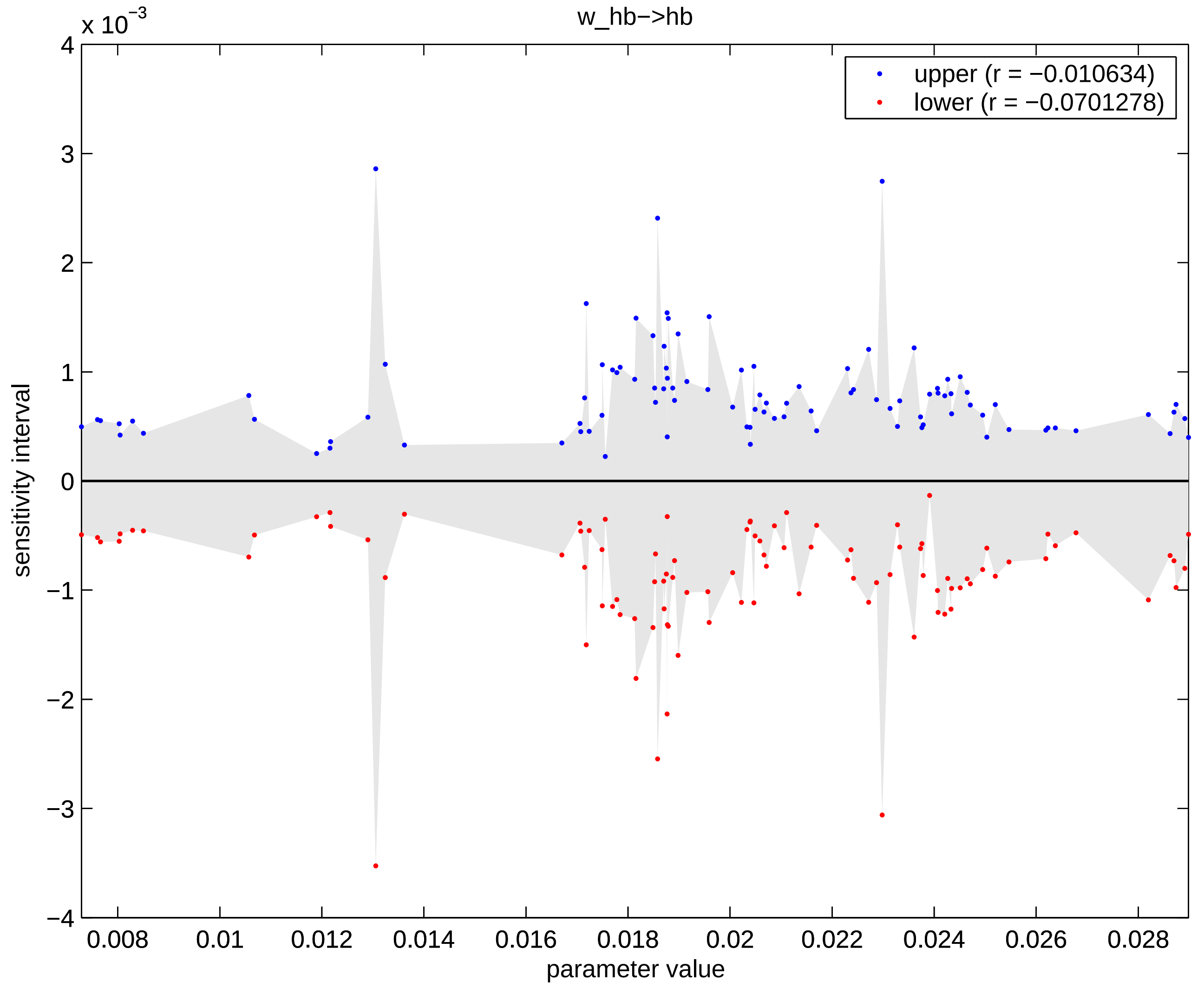
w_hb−>hb
x 10^−3
upper (r = −0.010634)
lower (r = −0.0701278)
4
3
2
1
0
−1
−2
−3
−4
sensitivity interval
0.008
0.01
0.012
0.014
0.016
0.018
0.02
0.022
0.024
0.026
0.028
parameter value

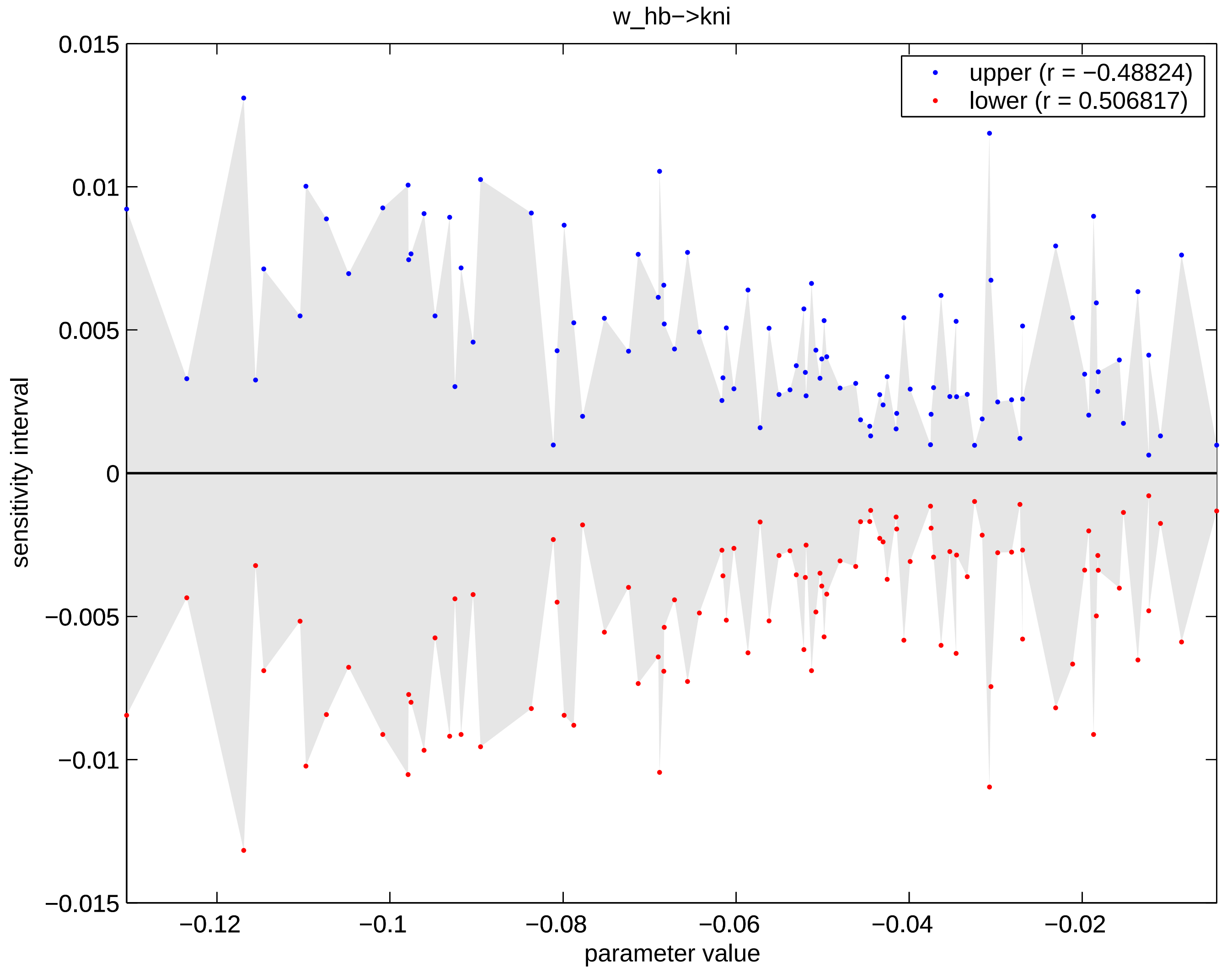

w_hb−>kni
upper (r = −0.48824)
lower (r = 0.506817)
0.015
0.01
0.005
0
−0.005
−0.01
−0.015
−0.12
−0.1
−0.08
−0.06
−0.04
−0.02
sensitivity interval
parameter value

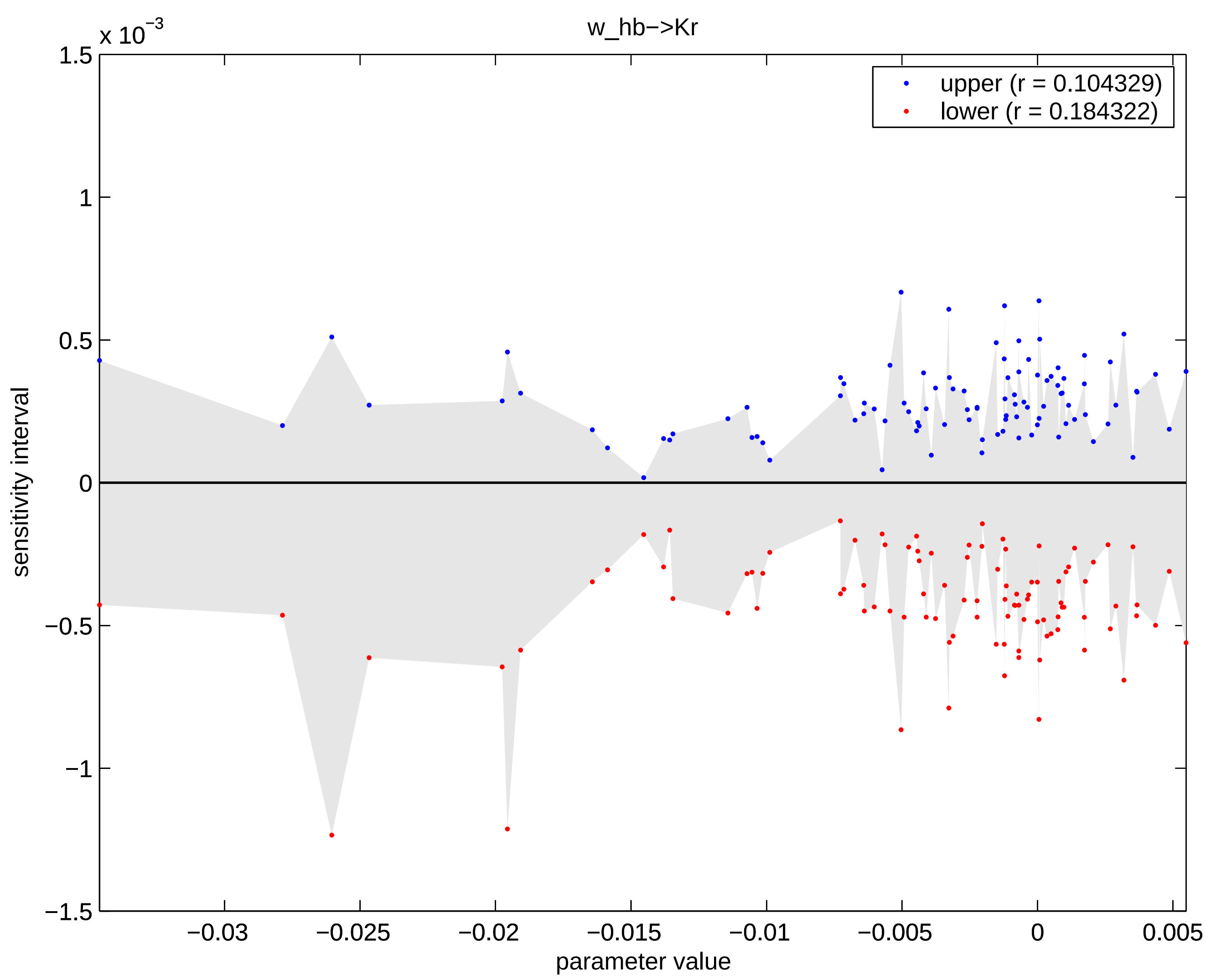

w_hb−>Kr
x 10^−3
upper (r = 0.104329)
lower (r = 0.184322)
sensitivity interval
parameter value
1.5
1
0.5
0
−0.5
−1
−1.5
−0.03
−0.025
−0.02
−0.015
−0.01
−0.005
0
0.005

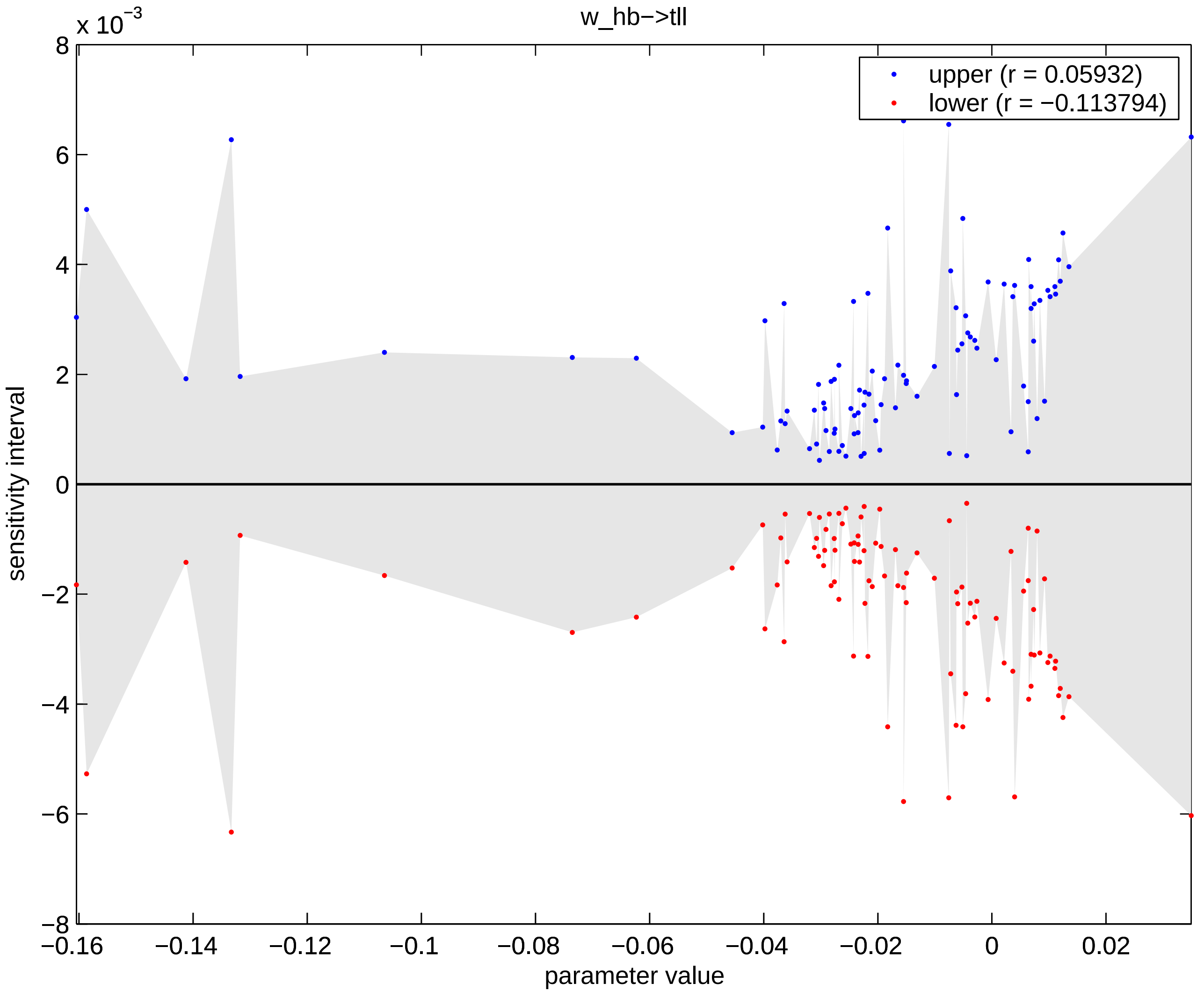

w_hb−>tll
x 10^−3
upper (r = 0.05932)
lower (r = −0.113794)
sensitivity interval
parameter value
8
6
4
2
0
−2
−4
−6
−8
−0.16
−0.14
−0.12
−0.1
−0.08
−0.06
−0.04
−0.02
0
0.02

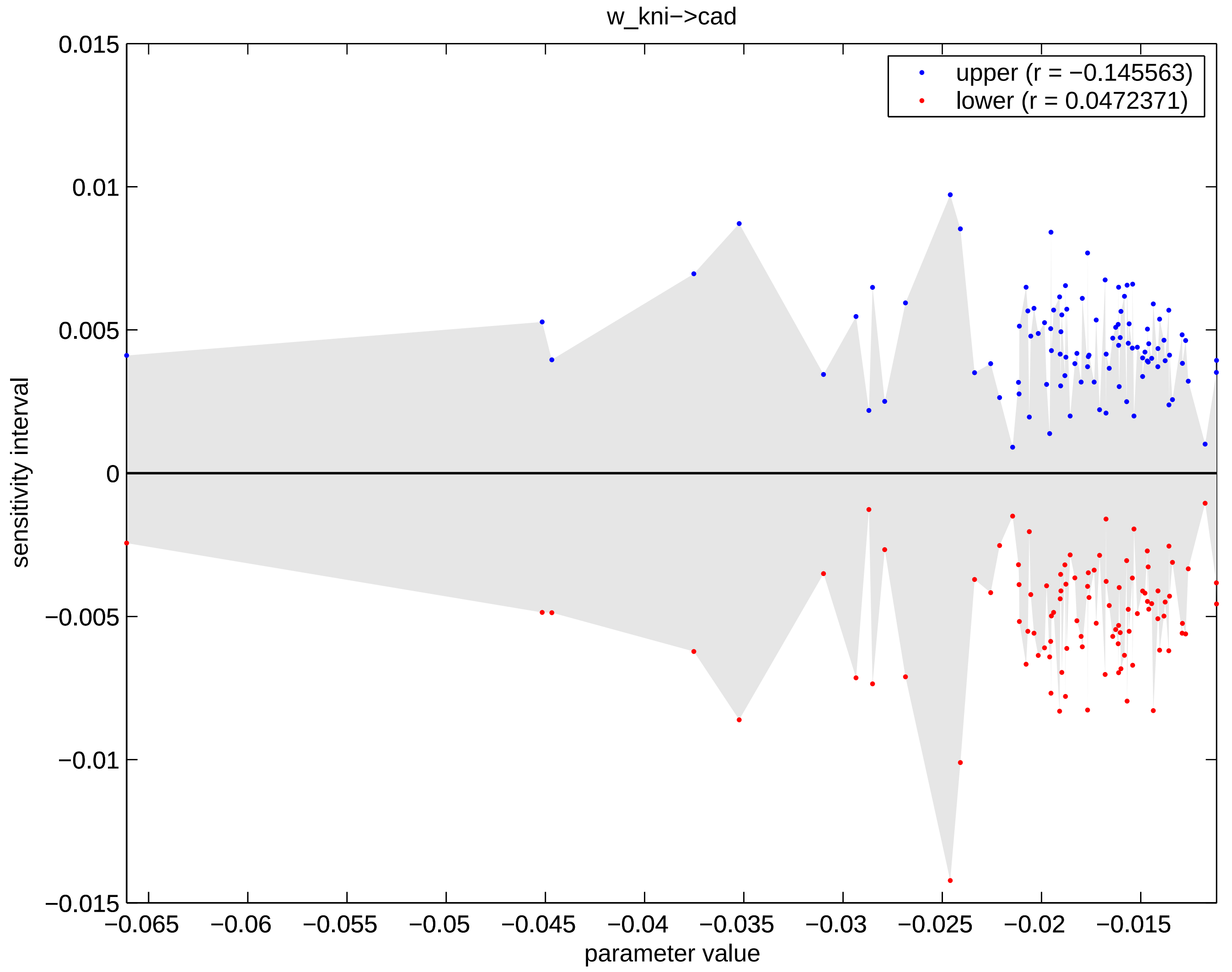
w_kni−>cad
upper (r = −0.145563)
lower (r = 0.0472371)
0.015
0.01
0.005
0
−0.005
−0.01
−0.015
−0.065
−0.06
−0.055
−0.05
−0.045
−0.04
−0.035
−0.03
−0.025
−0.02
−0.015
sensitivity interval
parameter value

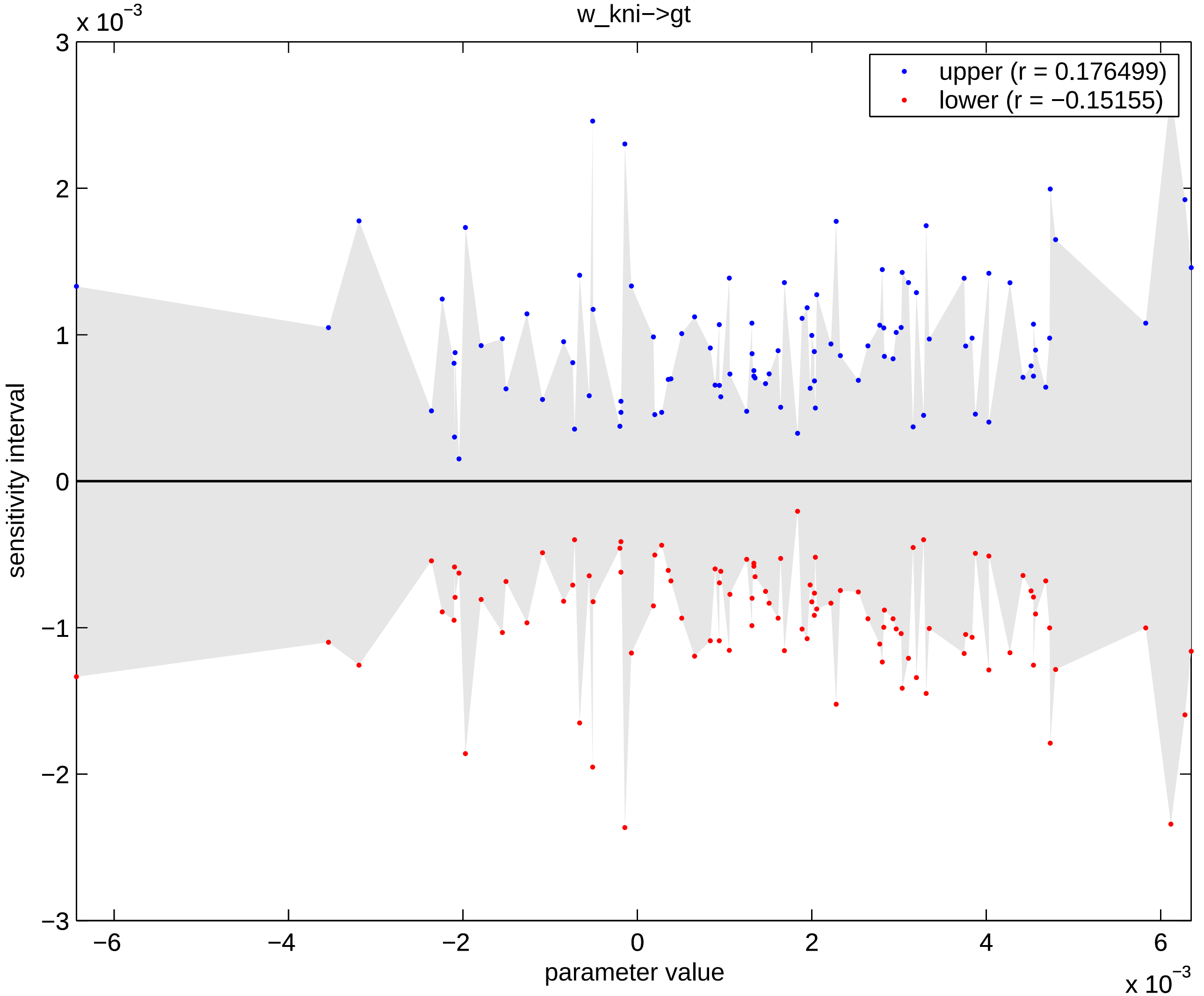
w_kni−>gt
x 10^−3
upper (r = 0.176499)
lower (r = −0.15155)
sensitivity interval
parameter value
x 10^−3
3
2
1
0
−1
−2
−3
−6
−4
−2
0
2
4
6

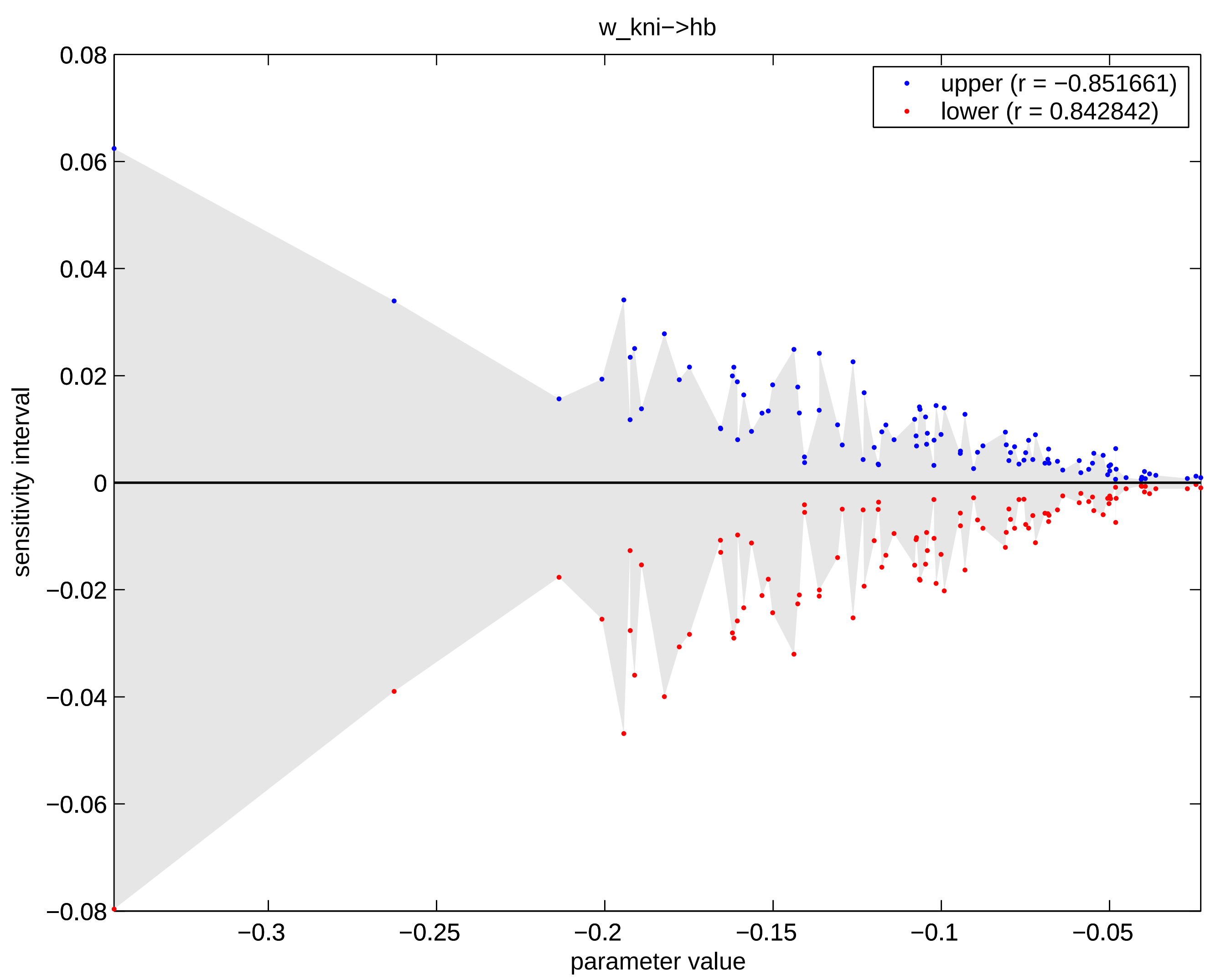

w_kni−>hb
upper (r = −0.851661)
lower (r = 0.842842)
sensitivity interval
parameter value
0.08
0.06
0.04
0.02
0
−0.02
−0.04
−0.06
−0.08
−0.3
−0.25
−0.2
−0.15
−0.1
−0.05

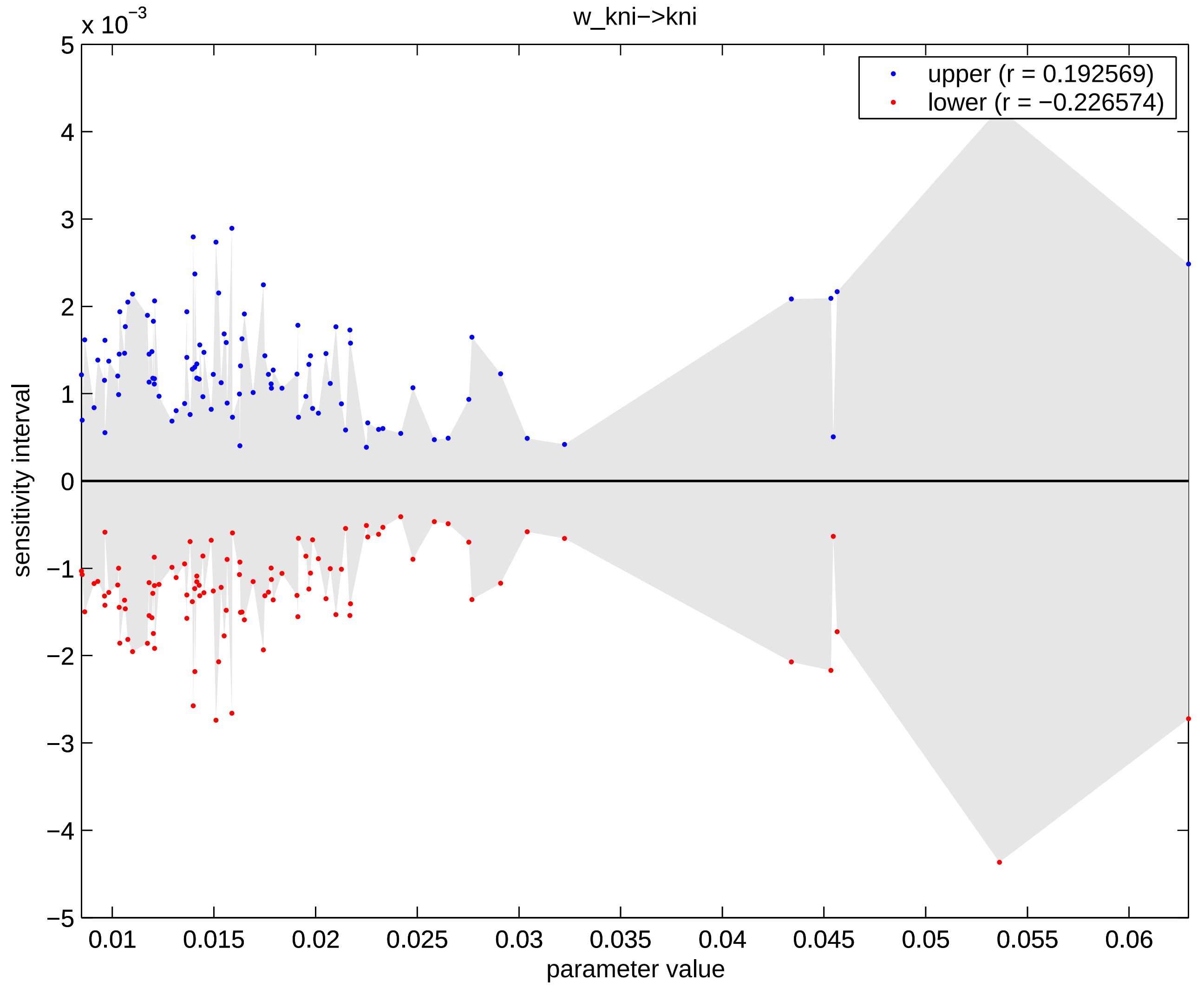

w_kni−>kni
x 10^−3
upper (r = 0.192569)
lower (r = −0.226574)
sensitivity interval
parameter value
5
4
3
2
1
0
−1
−2
−3
−4
−5
0.01
0.015
0.02
0.025
0.03
0.035
0.04
0.045
0.05
0.055
0.06

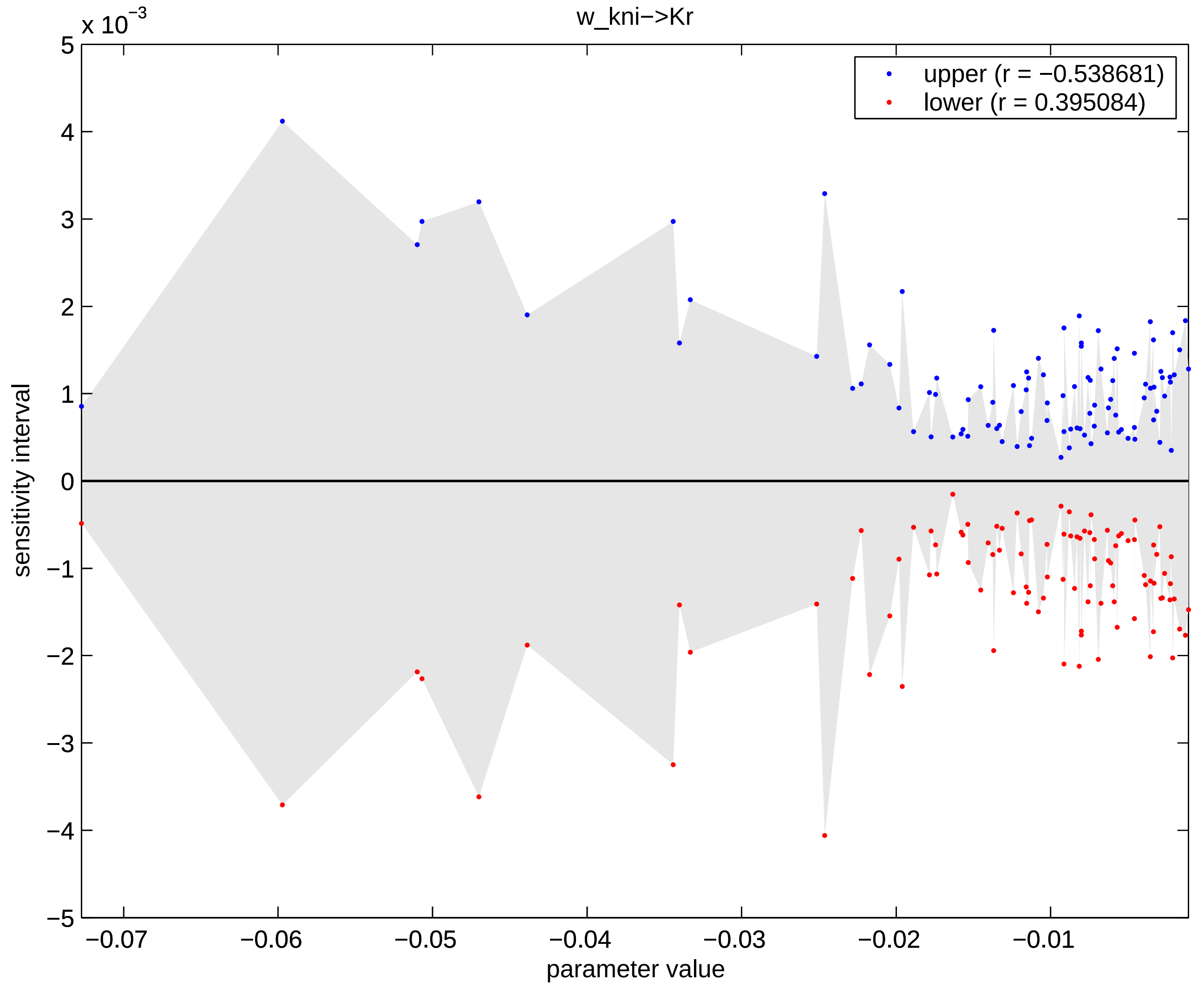

w_kni−>Kr
x 10^−3
upper (r = −0.538681)
lower (r = 0.395084)
sensitivity interval
parameter value
5
4
3
2
1
0
−1
−2
−3
−4
−5
−0.07
−0.06
−0.05
−0.04
−0.03
−0.02
−0.01

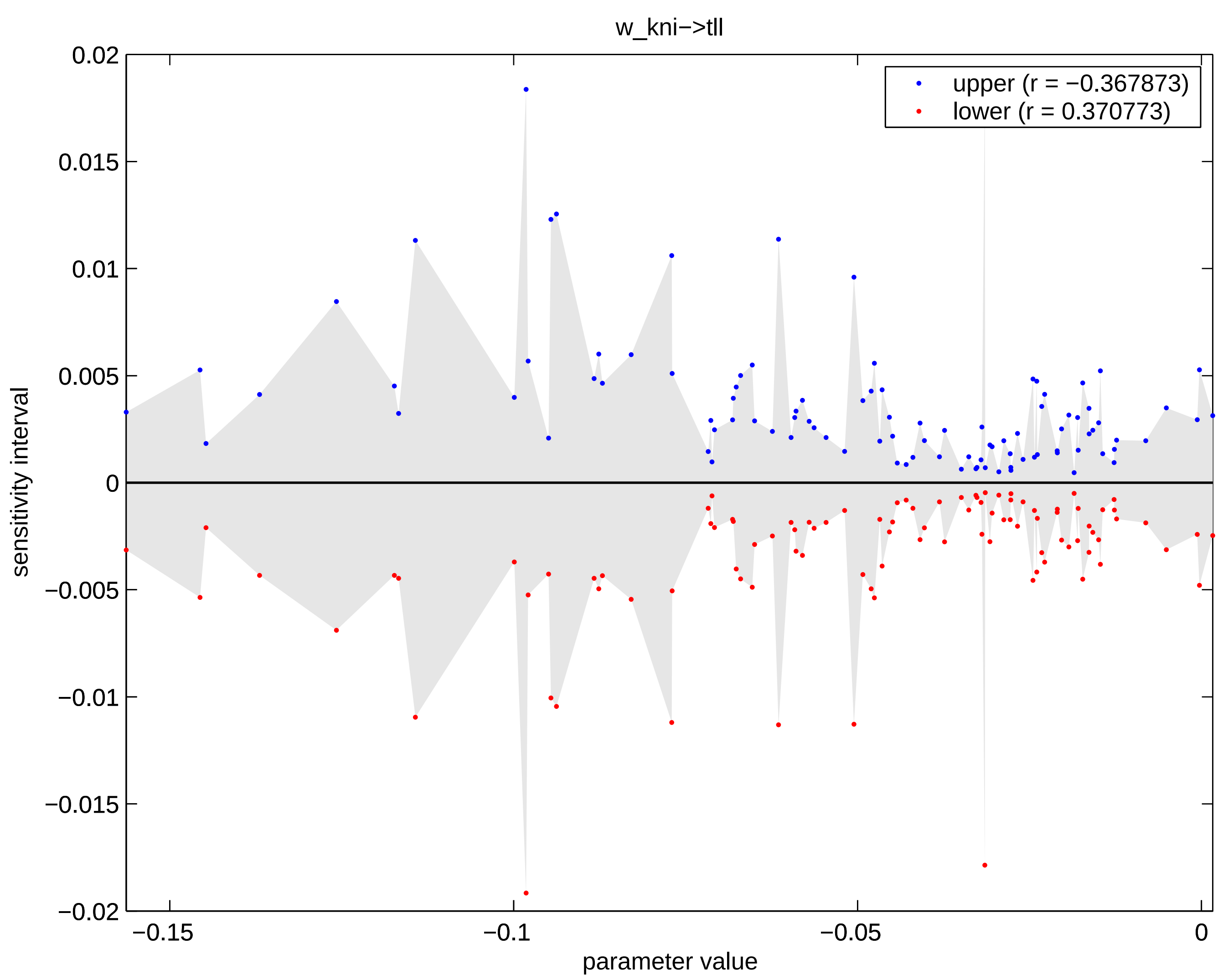
w_kni−>tll
upper (r = −0.367873)
lower (r = 0.370773)
sensitivity interval
parameter value
0.02
0.015
0.01
0.005
0
−0.005
−0.01
−0.015
−0.02
−0.15
−0.1
−0.05
0

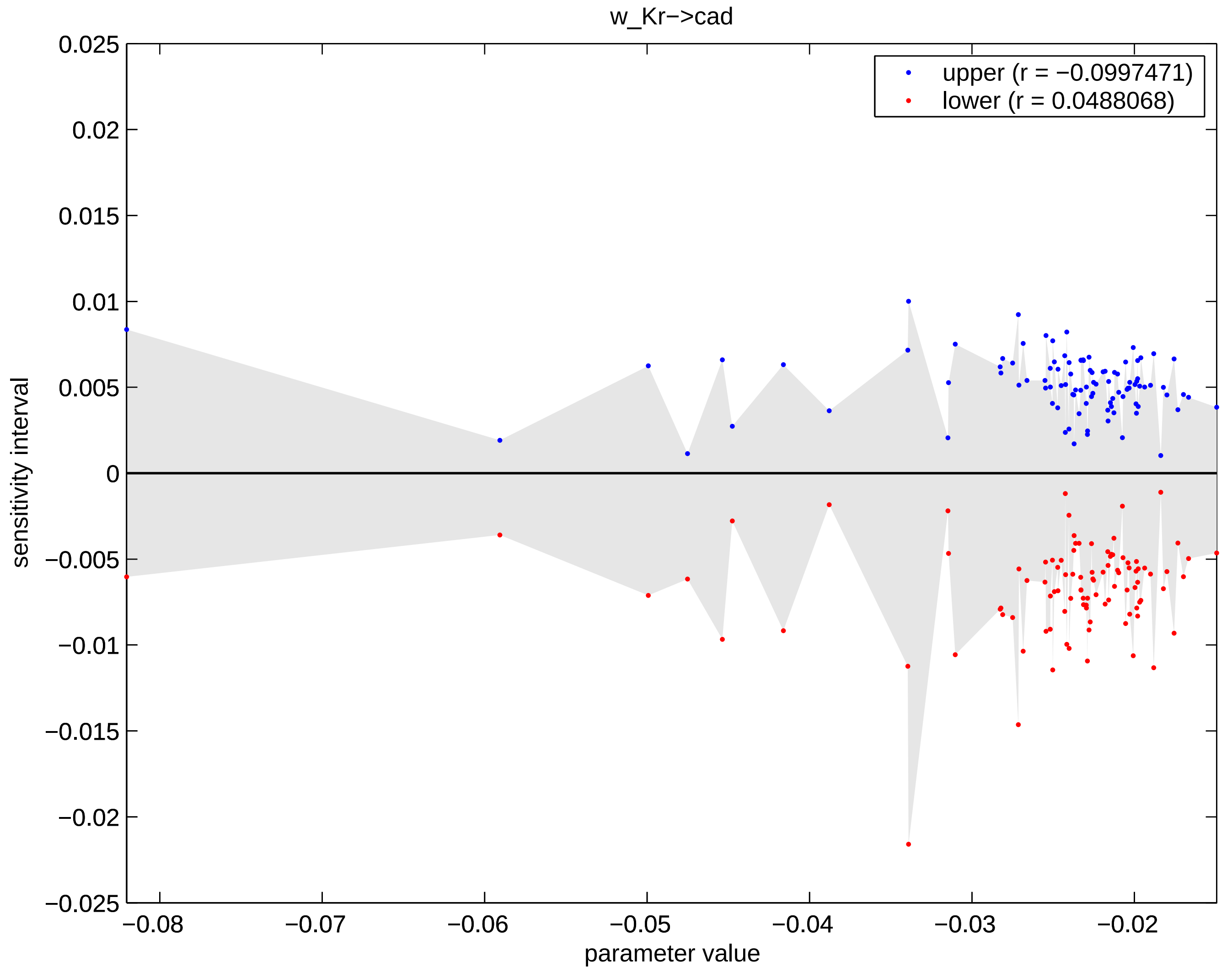
w_Kr−>cad
upper (r = −0.0997471)
lower (r = 0.0488068)
sensitivity interval
parameter value
0.025
0.02
0.015
0.01
0.005
0
−0.005
−0.01
−0.015
−0.02
−0.025
−0.08
−0.07
−0.06
−0.05
−0.04
−0.03
−0.02

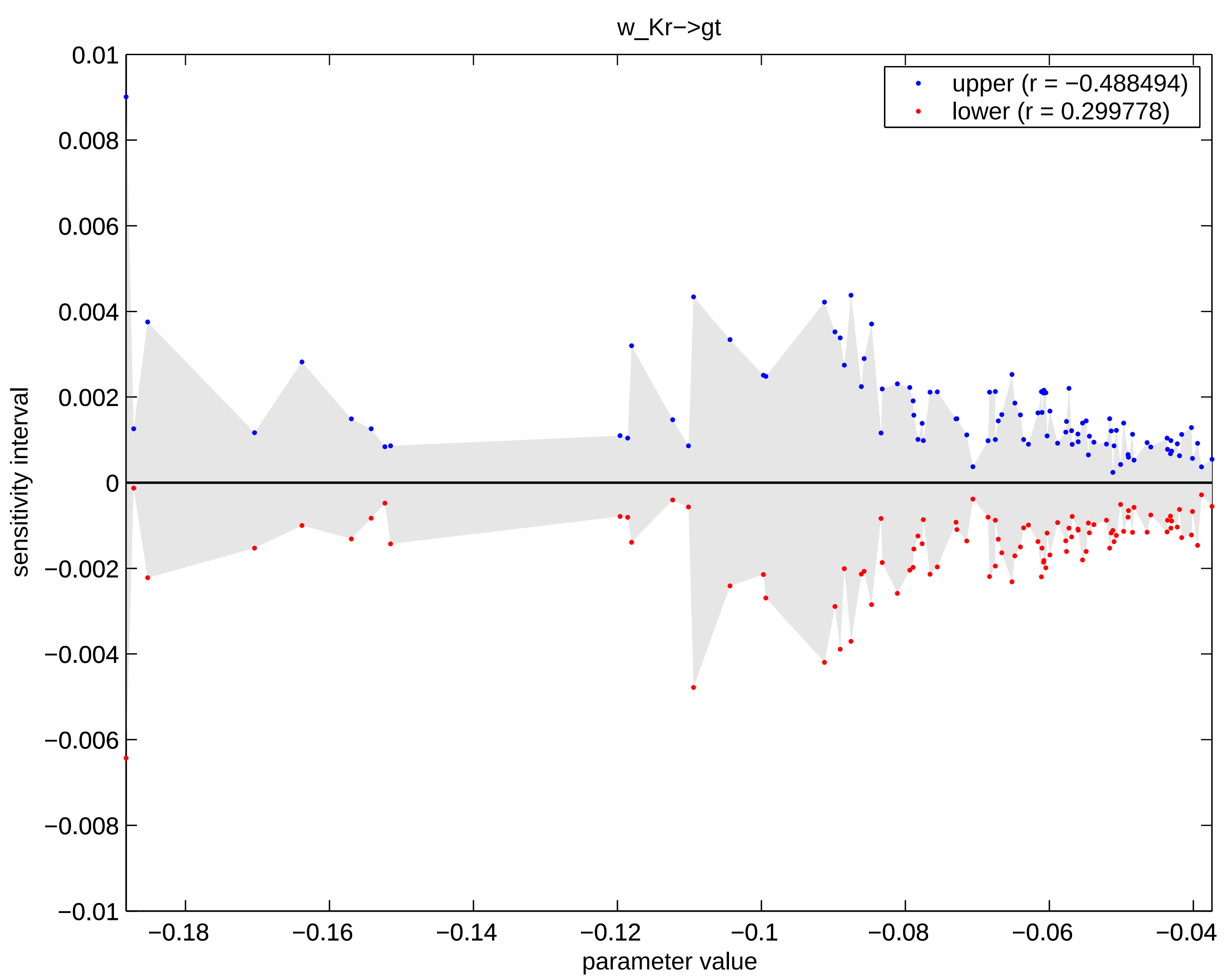

w_Kr−>gt
upper (r = −0.488494)
lower (r = 0.299778)
sensitivity interval
parameter value
0.01
0.008
0.006
0.004
0.002
0
−0.002
−0.004
−0.006
−0.008
−0.01
−0.18
−0.16
−0.14
−0.12
−0.1
−0.08
−0.06
−0.04

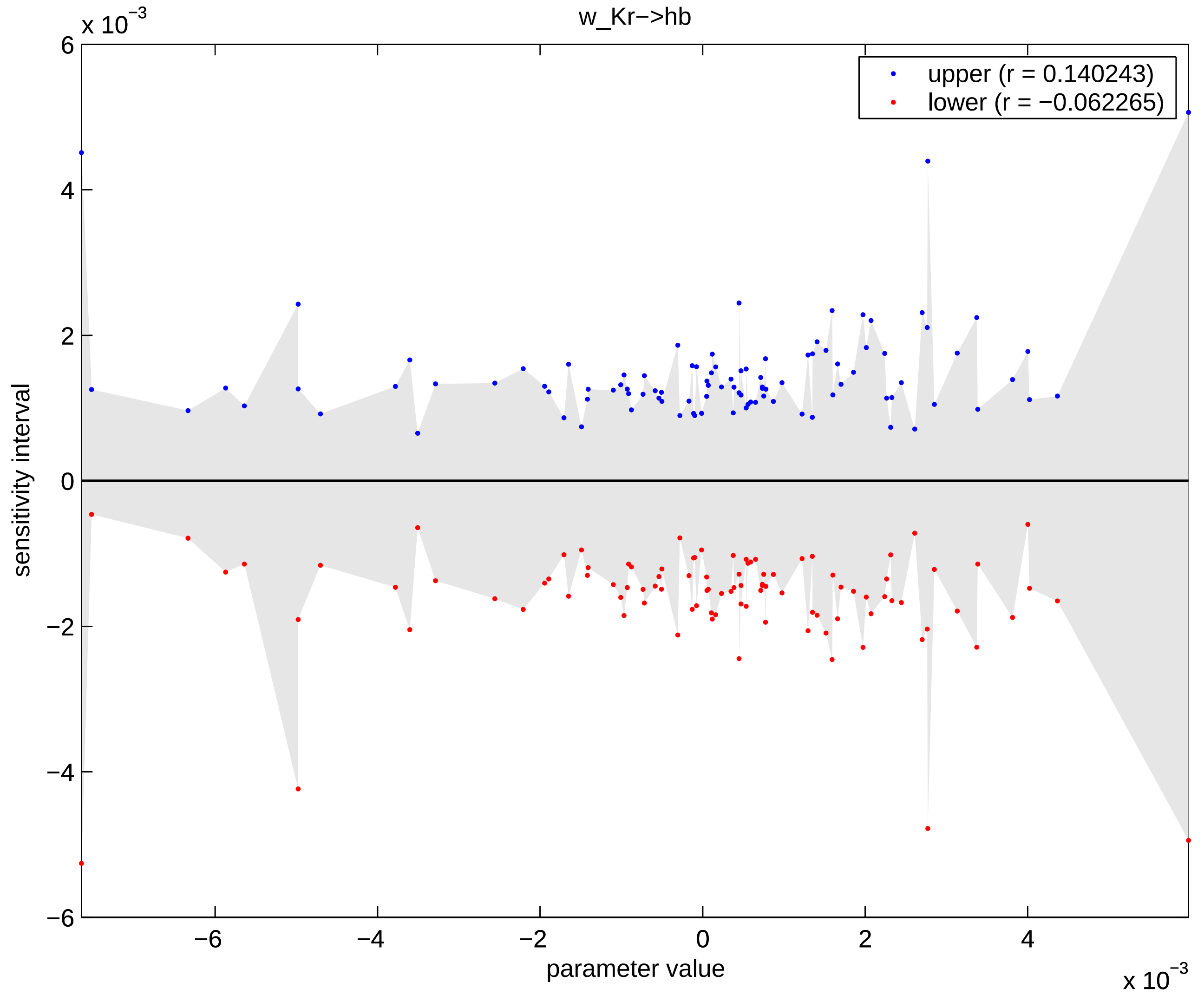

w_Kr−>hb
x 10^−3
upper (r = 0.140243)
lower (r = −0.062265)
6
4
2
0
−2
−4
−6
sensitivity interval
−6
−4
−2
0
2
4
parameter value
x 10^−3

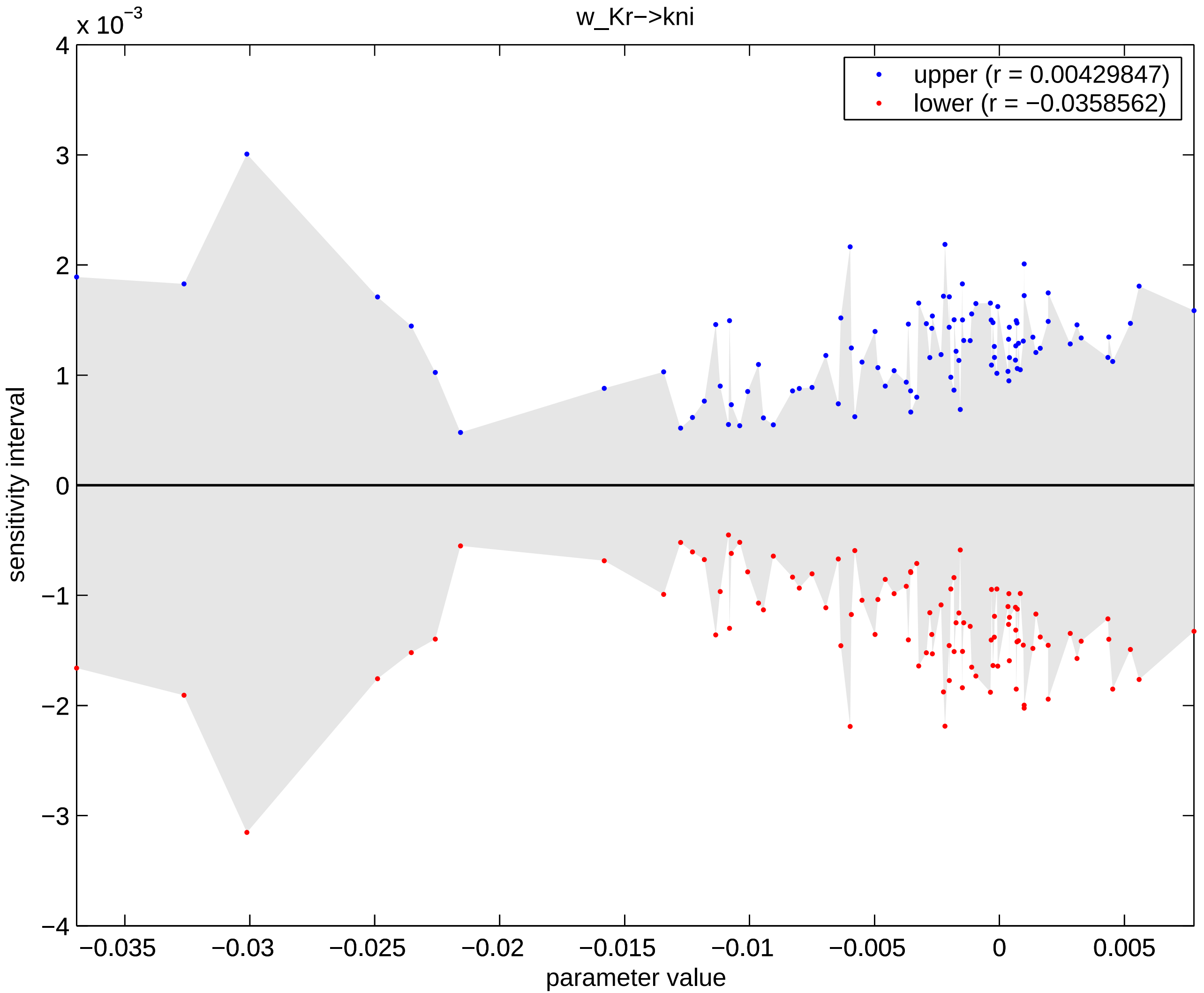

w_Kr−>kni
x 10^−3
upper (r = 0.00429847)
lower (r = −0.0358562)
sensitivity interval
parameter value
4
3
2
1
0
−1
−2
−3
−4
−0.035
−0.03
−0.025
−0.02
−0.015
−0.01
−0.005
0
0.005

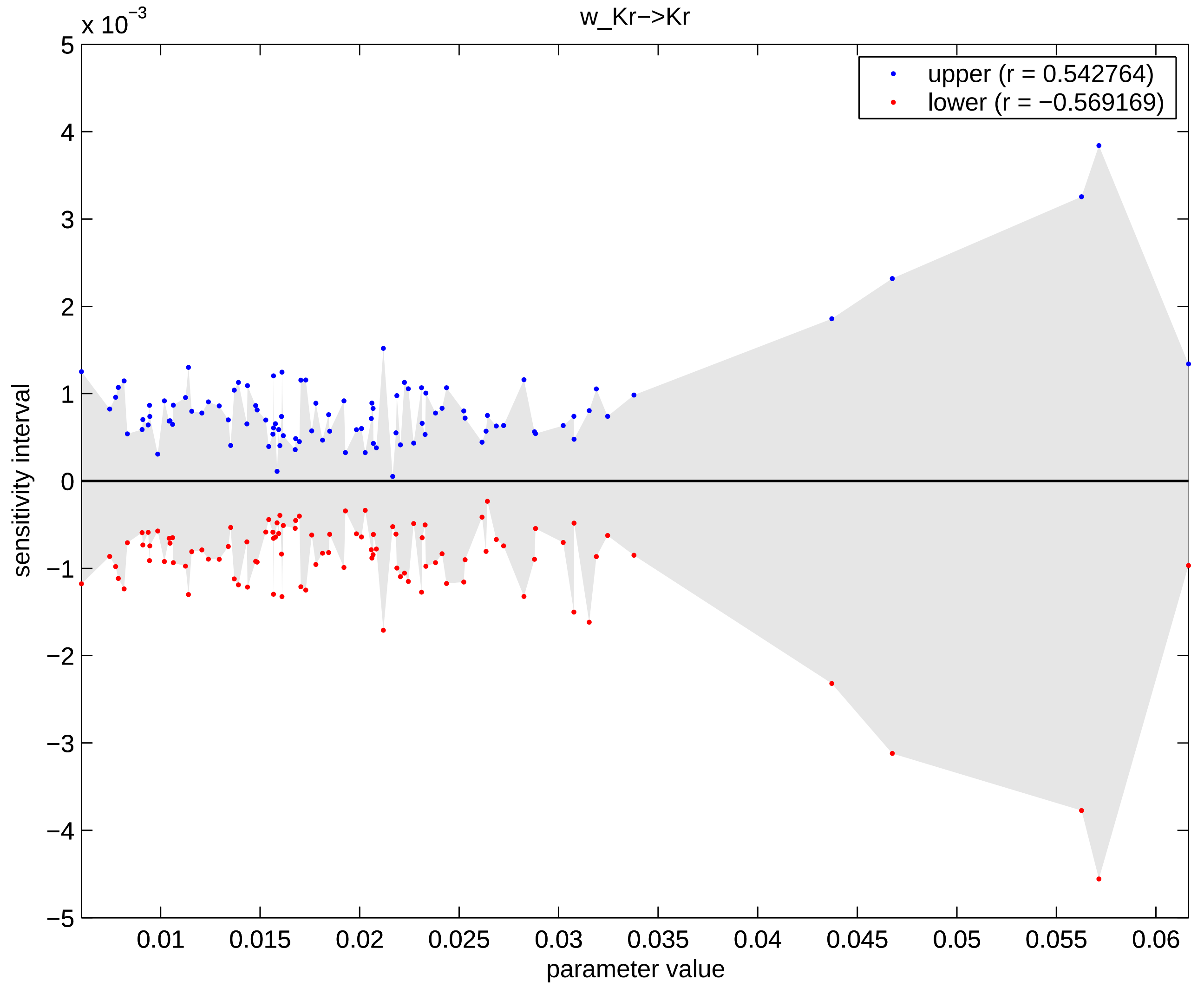

w_Kr−>Kr
x 10^−3
upper (r = 0.542764)
lower (r = −0.569169)
sensitivity interval
parameter value
5
4
3
2
1
0
−1
−2
−3
−4
−5
0.01
0.015
0.02
0.025
0.03
0.035
0.04
0.045
0.05
0.055
0.06

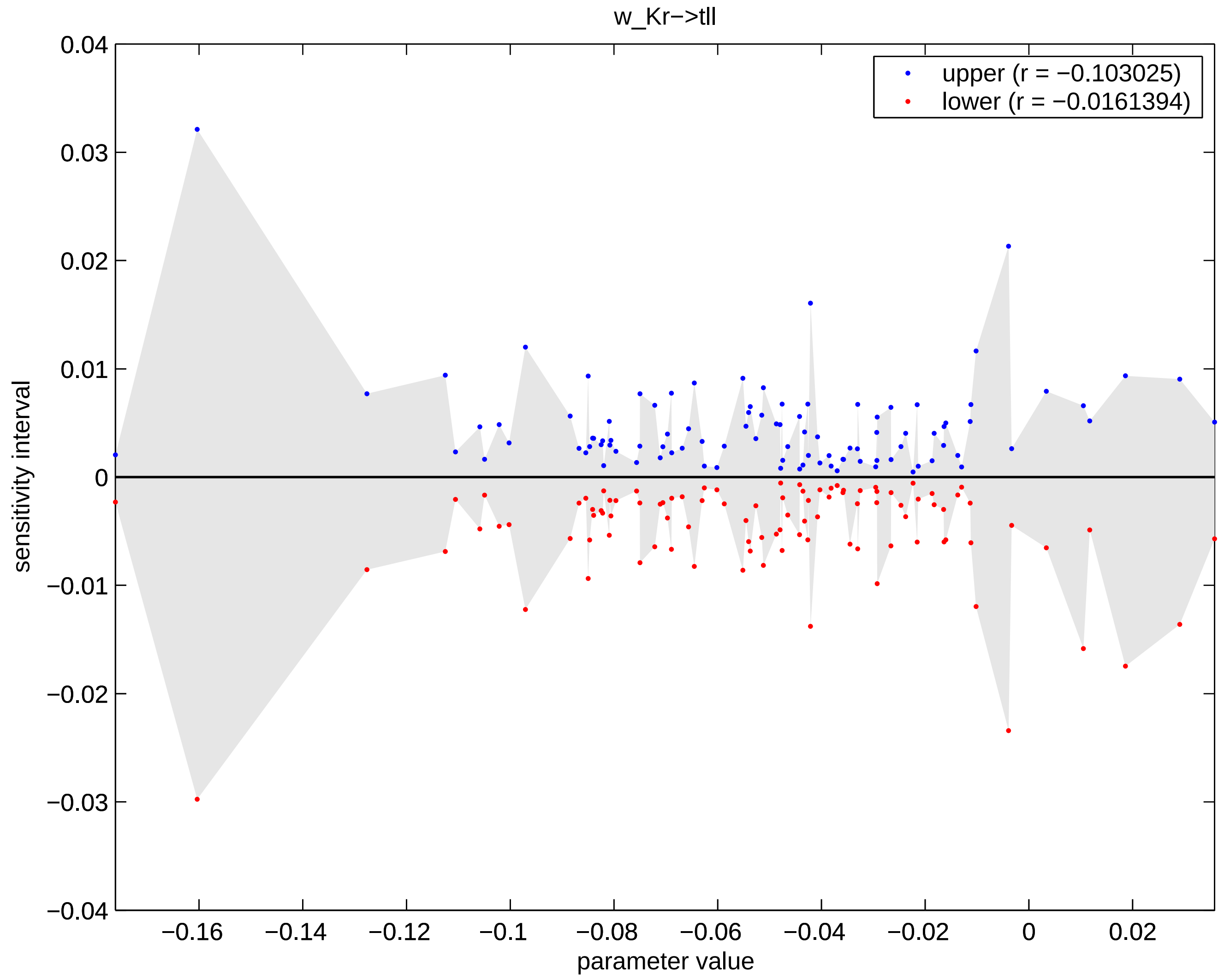

w_Kr−>tll
upper (r = −0.103025)
lower (r = −0.0161394)
sensitivity interval
parameter value
0.04
0.03
0.02
0.01
0
−0.01
−0.02
−0.03
−0.04
−0.16
−0.14
−0.12
−0.1
−0.08
−0.06
−0.04
−0.02
0
0.02

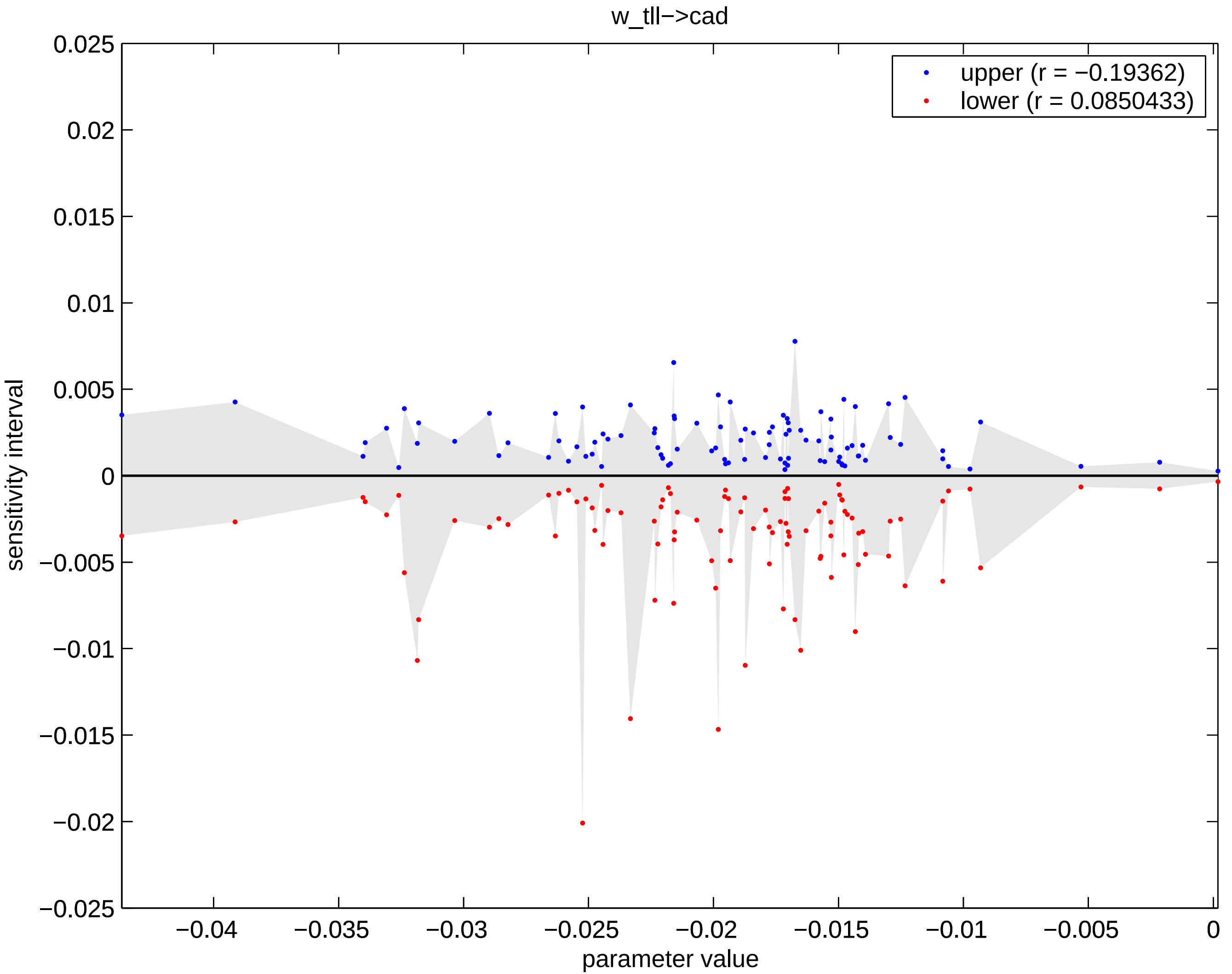
w_tll−>cad
upper (r = −0.19362)
lower (r = 0.0850433)
sensitivity interval
parameter value
0.025
0.02
0.015
0.01
0.005
0
−0.005
−0.01
−0.015
−0.02
−0.025
−0.04
−0.035
−0.03
−0.025
−0.02
−0.015
−0.01
−0.005
0

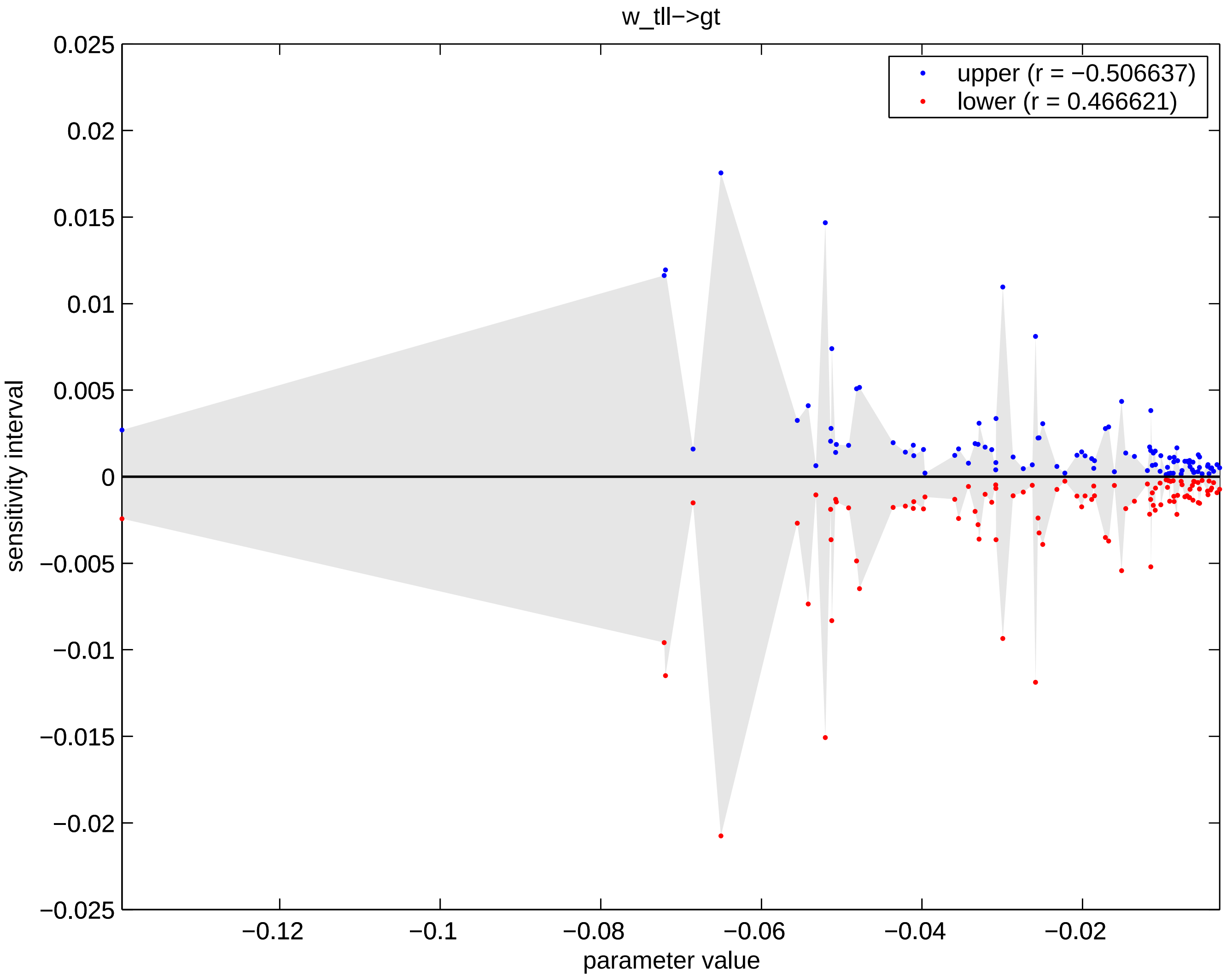

w_tll->gt
upper (r = −0.506637)
lower (r = 0.466621)
0.025
0.02
0.015
0.01
0.005
0
−0.005
−0.01
−0.015
−0.02
−0.025
−0.12
−0.1
−0.08
−0.06
−0.04
−0.02
sensitivity interval
parameter value

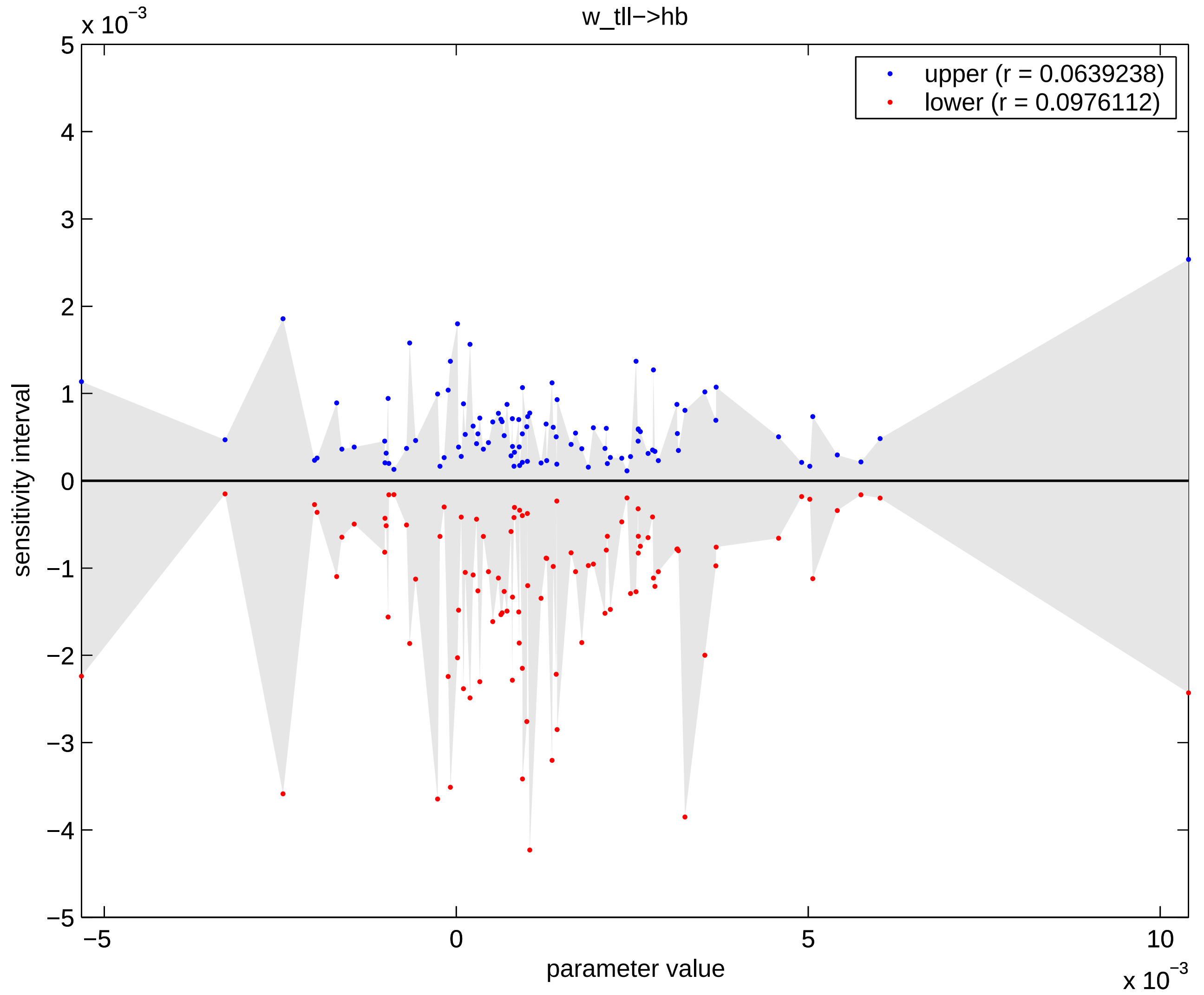

w_tll->hb
x 10^-3
upper (r = 0.0639238)
lower (r = 0.0976112)
sensitivity interval
parameter value
x 10^-3
5
4
3
2
1
0
−1
−2
−3
−4
−5
−5
0
5
10

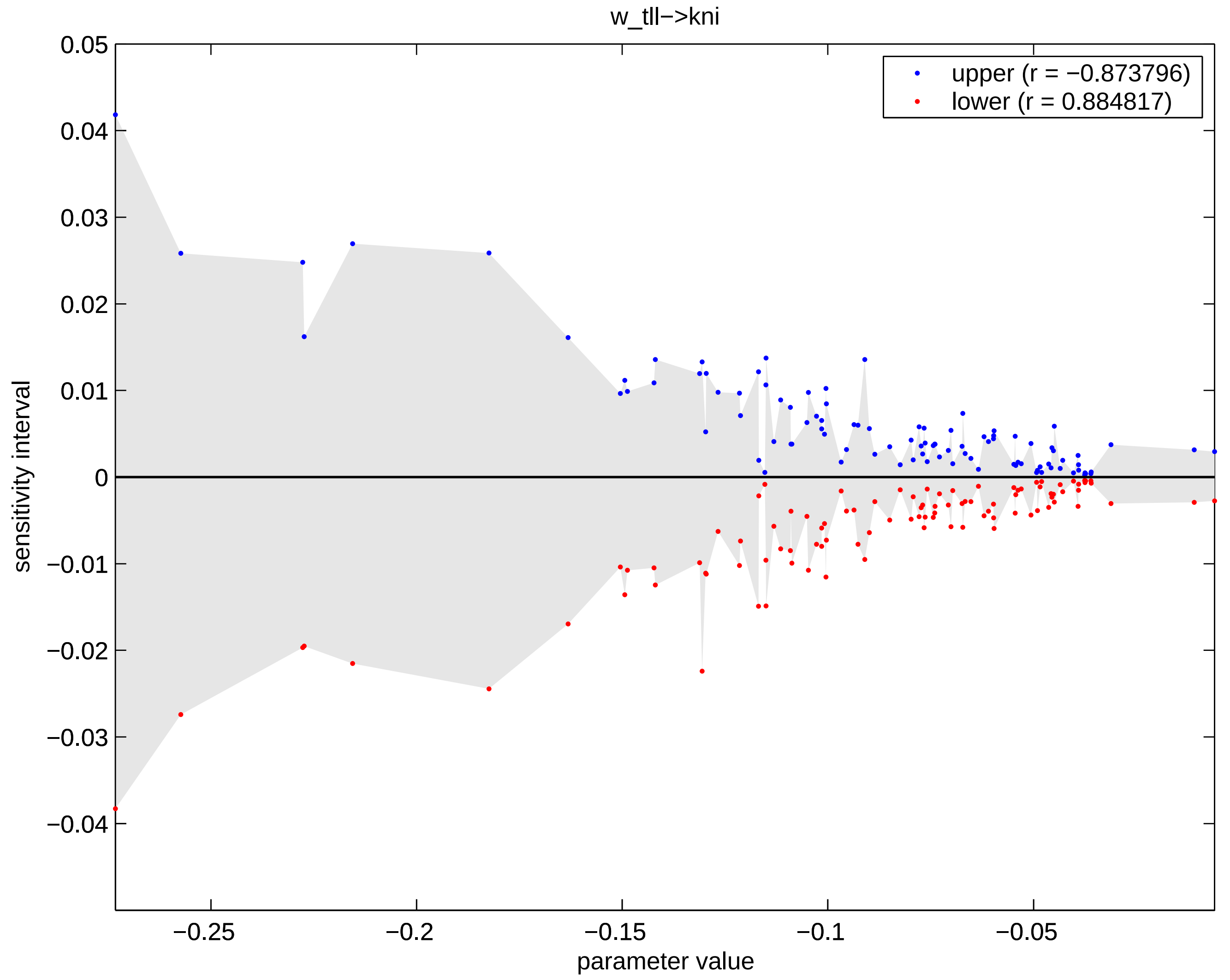

w_tll−>kni
upper (r = −0.873796)
lower (r = 0.884817)
sensitivity interval
parameter value
0.05
0.04
0.03
0.02
0.01
0
−0.01
−0.02
−0.03
−0.04
−0.25
−0.2
−0.15
−0.1
−0.05

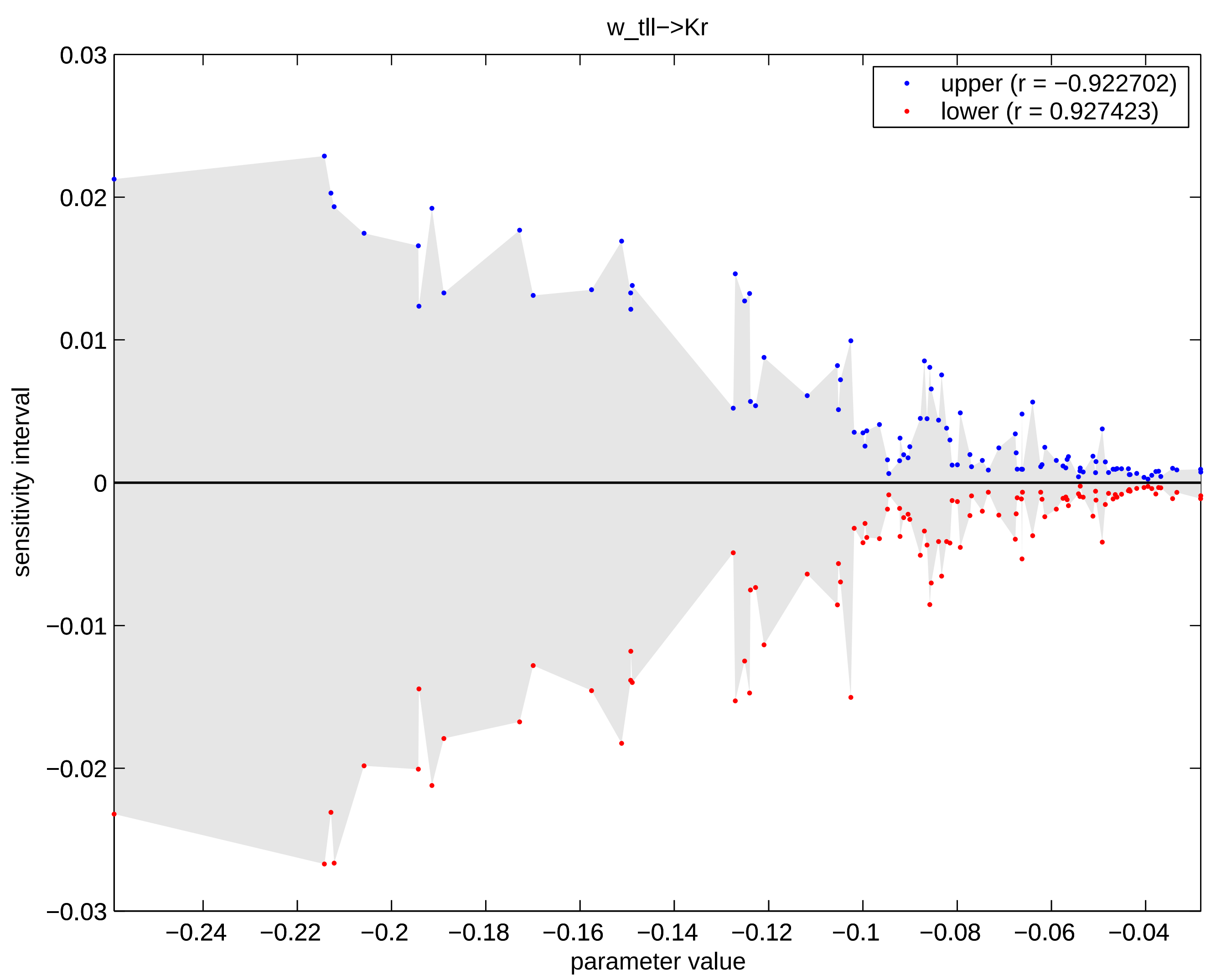

w_tll−>Kr
upper (r = −0.922702)
lower (r = 0.927423)
0.03
0.02
0.01
0
−0.01
−0.02
−0.03
sensitivity interval
−0.24
−0.22
−0.2
−0.18
−0.16
−0.14
−0.12
−0.1
−0.08
−0.06
−0.04
parameter value

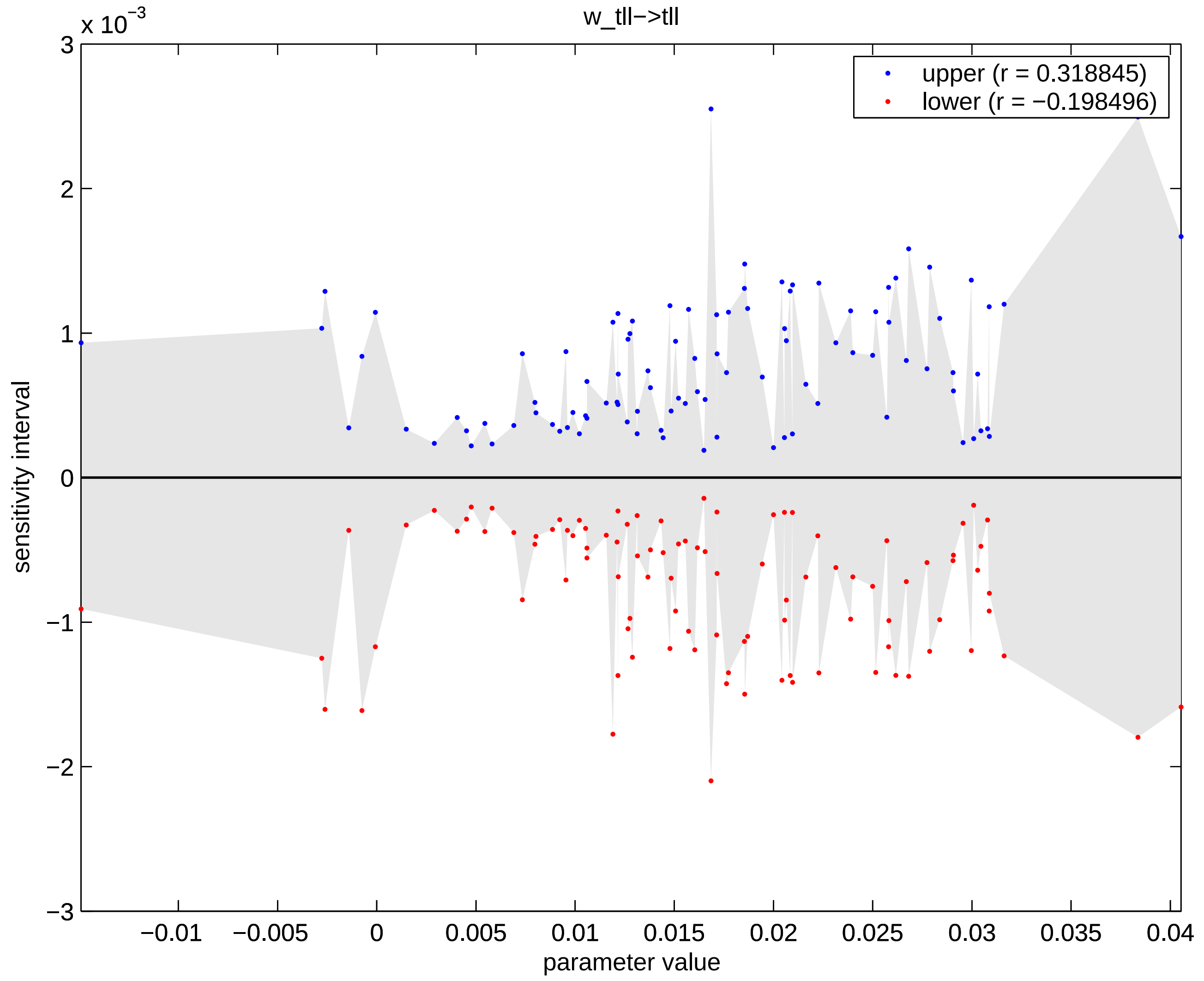

w_tll−>tll
x 10^−3
upper (r = 0.318845)
lower (r = −0.198496)
sensitivity interval
parameter value
3
2
1
0
−1
−2
−3
−0.01
−0.005
0
0.005
0.01
0.015
0.02
0.025
0.03
0.035
0.04
